# Toward Chiral Recognition by Design: Uncovering the Self-Enantioresolving Properties of Chiral Amine Derivatives

**DOI:** 10.1021/jacs.5c01251

**Published:** 2025-05-23

**Authors:** Anka Hagelschuer, Damián Padín, Vanda Dašková, Ben L. Feringa

**Affiliations:** Stratingh Institute for Chemistry, University of Groningen, Nijenborgh 4, 9747 AG Groningen, The Netherlands

## Abstract

The study of chiral recognition phenomena is key for understanding biological processes, designing bioactive compounds, and for asymmetric catalysis and chiral analysis. In addition, phenomena related to the self-recognition of enantiomers are highly relevant in emergence-of-homochirality research and supramolecular chemistry. However, the design of molecules exhibiting chiral self-recognition remains challenging, and its observation is mainly based on serendipity. Here, we report a comprehensive study of the self-enantiorecognition properties of chiral amine-derived building blocks frequently encountered in organic synthesis. Through a structure−activity relationship study, multiple families of chiral amine derivatives, featuring self-complementary hydrogen-bond donor and acceptor groups, have been found to exhibit self-induced diastereomeric anisochronism (SIDA) by NMR analysis, a rather unexplored form of self-recognition of enantiomers. Our study suggests that the self-enantiorecognition properties of many common building blocks in asymmetric synthesis might have remained inadvertently unnoticed. We have also rationalized the origins of their SIDA effect and demonstrated their potential as an in situ probe for the determination of enantiomeric purity, the analysis of supramolecular interactions, and the study of reaction mechanisms. We anticipate that the principles outlined here will contribute to fostering the use of the SIDA effect in fundamental stereochemical studies, asymmetric synthesis, catalysis, and supramolecular chemistry.

## Graphical abstract

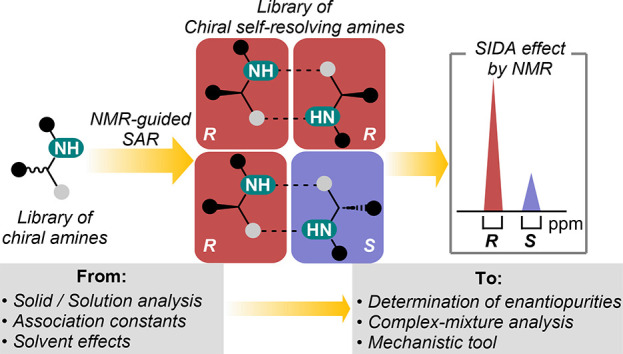

## Introduction

From D-sugars and L-amino acids to DNA and proteins, the single-handedness of biological molecules is key for molecular recognition and information transfer processes in living organisms.^1^ As a consequence, the Life Sciences industry heavily relies on the design, preparation, and analysis of optically active compounds, including pharmaceuticals, agrochemicals, fragrances, and new materials, capable of selectively interacting with biological systems.^2^ Although the underlying fundamental principles that govern enantiorecognition and chirality transfer mechanisms are not yet fully understood,^3^ the formation of transient diastereomeric complexes between the enantiomers of a chiral molecule (guest, e.g., a drug) and a chiral selector (host, e.g., an enzyme) through three points of attractive/repulsive interactions is, in most cases, envisioned to be the basis of these phenomena (Figure 1a).^4^ Ultimately, the distinct binding interactions between the two enantiomers and the chiral selector are responsible for the enantiorecognition. Importantly, this mechanism is central not only in biological systems but also in catalysis, the separation of enantiomers, and the determination of enantiomeric purities by chromatographic or spectroscopic techniques.^5^

The self-recognition of enantiomers represents an intriguing case of enantiorecognition via formation of homo- and heterochiral complexes (Figure 1b).^6^ This phenomenon has proven to be the cornerstone of many hypotheses related to the emergence of prebiotic homochirality^1,7^ and, namely, in asymmetric autocatalysis,^8^ the spontaneous fractionation of enantiomers,^9^ conglomerate/racemate formation in crystallizations,^7b,10^ the Horeau effect,^11^ and the observation of nonlinear effects in synthesis and catalysis.^12^ Unlike other forms of enantiorecognition, this mechanism does not require the presence of an external chiral selector; instead, the formation of noncovalent interactions between enantiomers leads to diastereomeric supramolecular associates that can exhibit different physicochemical properties (e.g., solubility, melting/boiling points, polarity).

An important, but often overlooked, form of self-recognition of enantiomers is the self-induced diastereomeric anisochronism effect (SIDA effect) in NMR spectroscopy.^13,14^ Arising from the dynamic (and fast) equilibration between enantiomers and their diastereomeric homo- and heterochiral associates in solution, the SIDA effect gives rise to two sets of peaks in the NMR spectra of certain scalemic mixtures (0% < e.e. <100%), whose relative ratio matches the enantiomeric composition (Figure 1c). That is, unlike other NMR techniques that require the use of achiral^15^ or chiral derivatizing/solvating agents or lanthanide shift reagents,^16^ the SIDA effect enables the direct read-out of the enantiomeric ratio (e.r.) by a simple NMR experiment in the absence of external chiral sources or additives. Each set of peaks relates to the weighted average chemical shifts (δ_obs_) of all species where each enantiomer is involved (*R* + *RR* + *RS* vs *S* + *SS* + *RS*), reflecting the distinct time-averaged local environments for each associate. As a result, racemic and enantiopure compounds show only one set of peaks but different chemical shifts.^17^ Despite the potential paradigm shift that the SIDA effect can represent for asymmetric synthesis and the study of chiral recognition phenomena, its observation has been considered a serendipitous finding and case-dependent.^18^ An understanding of the stereochemical features that make a compound “SIDA-active” is, therefore, still lacking, leaving the SIDA effect as an anecdotal phenomenon and precluding further applications in synthesis and supramolecular chemistry. In an effort to better understand this effect and leverage its benefits, our group has recently demonstrated that, by applying supramolecular principles, self-recognition of enantiomers can be achieved by design and the SIDA effect can be used as a convenient analytical tool to accelerate reaction optimization in asymmetric catalysis.^19^ Critical to our design was the introduction of self-complementary functional groups (a hydrogen-bond donor and an acceptor) next to a stereogenic center, which promoted the formation of dimeric complexes. Although we initially focused on the design of a new family of SIDA-active compounds, we envisioned that these features could actually be present in more common building blocks for organic synthesis than previously anticipated. As the SIDA effect relies on the formation of supramolecular complexes anchored through relatively weak intermolecular interactions, the choice of solvent, temperature, and concentration is critical for its observation. Given the prevalent use of medium-/high-polarity solvents such as CDCl_3_, DMSO-*d*_6_, or MeOD-*d*_4_ in NMR spectroscopy, many SIDA-active compounds might remain unnoticed if the solvent−solute interactions are too strong to enable intermolecular associations. Thus, we embarked on a structure−activity relationship (SAR) study to systematically decipher the stereochemical features that make a compound SIDA-active. Specifically, we focused our study on uncovering the self-recognition properties of chiral amine derivatives, being among the most important targets in asymmetric synthesis and present in >40% of industrially relevant pharmaceuticals, fine chemicals, and agrochemicals.^20^ Herein, we report the results of our SAR study and the discovery of the previously unnoticed self-resolving properties of numerous common building blocks in organic synthesis. In addition, we shed light on the origin of the SIDA effect and discuss some previously unexplored applications that can lead to a paradigm shift in the interpretation of chiral information in asymmetric synthesis, catalysis, and supramolecular chiral recognition processes.

**Figure 1:**
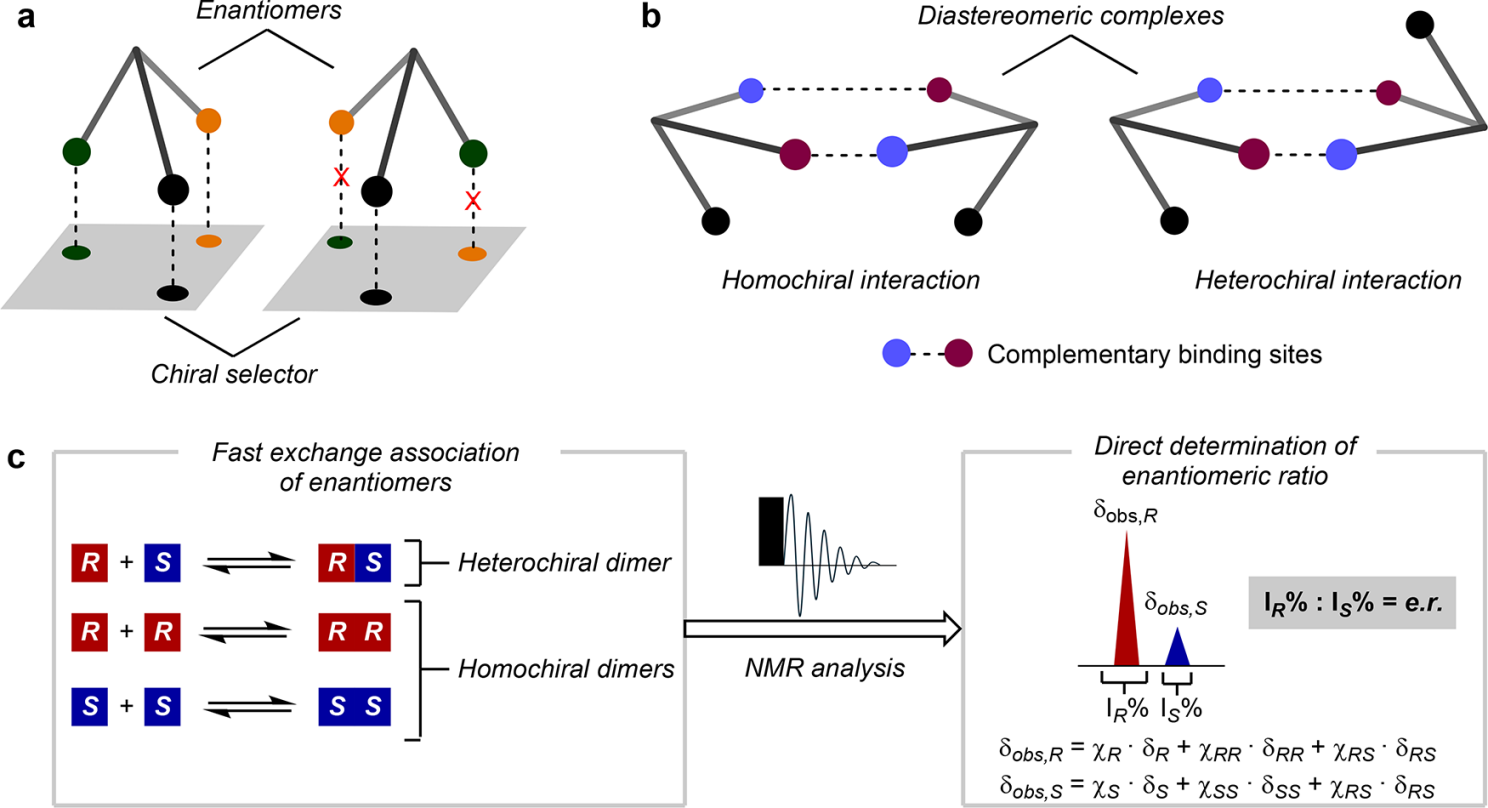
Chiral recognition mechanisms. (a) Three-point interaction model for recognition of enantiomers. (b) Self-recognition of enantiomers through formation of transient homo- and heterochiral associates. (c) Origin of the SIDA effect by NMR and its application to the direct determination of enantiomeric ratios (e.r.). δ_obs,*R*_/δ_obs,*S*_, observed NMR chemical shift of enantiomer *R* or *S*, respectively; δ_*R*_/δ_*S*_/δ_*RR*_/δ*s*_*S*_/δ_*RS*_, NMR chemical shift of each monomeric or dimeric species; *I*_*R*_%/*I*_*S*_%, relative percentage integral/area of the NMR signal; and χ_*R*_/χ_*S*_/χ_*RR*_/χ_*SS*_/χ_*RS*_, molar fraction for each monomeric or dimeric species.

## Results and Discussion

### SAR Study

To relate the molecular structure of a given compound to the presence or absence of SIDA effect by NMR, we prepared a library of scalemic amine derivatives (0% < e.e. < 100%) using standard asymmetric synthesis methods or by a combination of commercially available enantiopure amines of opposite configurations (Figure 2a), and we analyzed them by NMR spectroscopy under a variety of conditions. This library of scalemic amines with known enantiomeric purities was designed to (1) cover a broad chemical space featuring some of the most explored motives in organic synthesis, such as α-amino phosphonates (**1**), α-amino amides (**2**), α-amino esters (**3**) and 1-phenethylamines (**4**); (2) combine self-complementary hydrogen-bond donors (HBDs) and hydrogen-bond acceptors (HBAs) in close proximity to a stereogenic center, but preventing intramolecular self-association; and (3) include as many known examples from the literature as possible for the sake of comparison. Importantly, the amine groups were functionalized to display known HBD properties, including (thio)­ureas (**a−** **d**), phosphoramidates (**e**), phosphinamides (**f**), and amides (**g** and **h**). By confronting each HBD with its corresponding HBA, we could map combinations of functional groups that can lead to SIDA-active and inactive compounds (Figure 2b). Occasionally, the low solubility of certain scalemic derivatives hampered their evaluation as potential SIDA-active compounds under certain conditions. In addition, special care was taken to prevent spontaneous fractionation of enantiomers by preferential solubilization of homochiral aggregates,^9b,19,21^ which could lead to a misinterpretation of the results.

**Figure 2:**
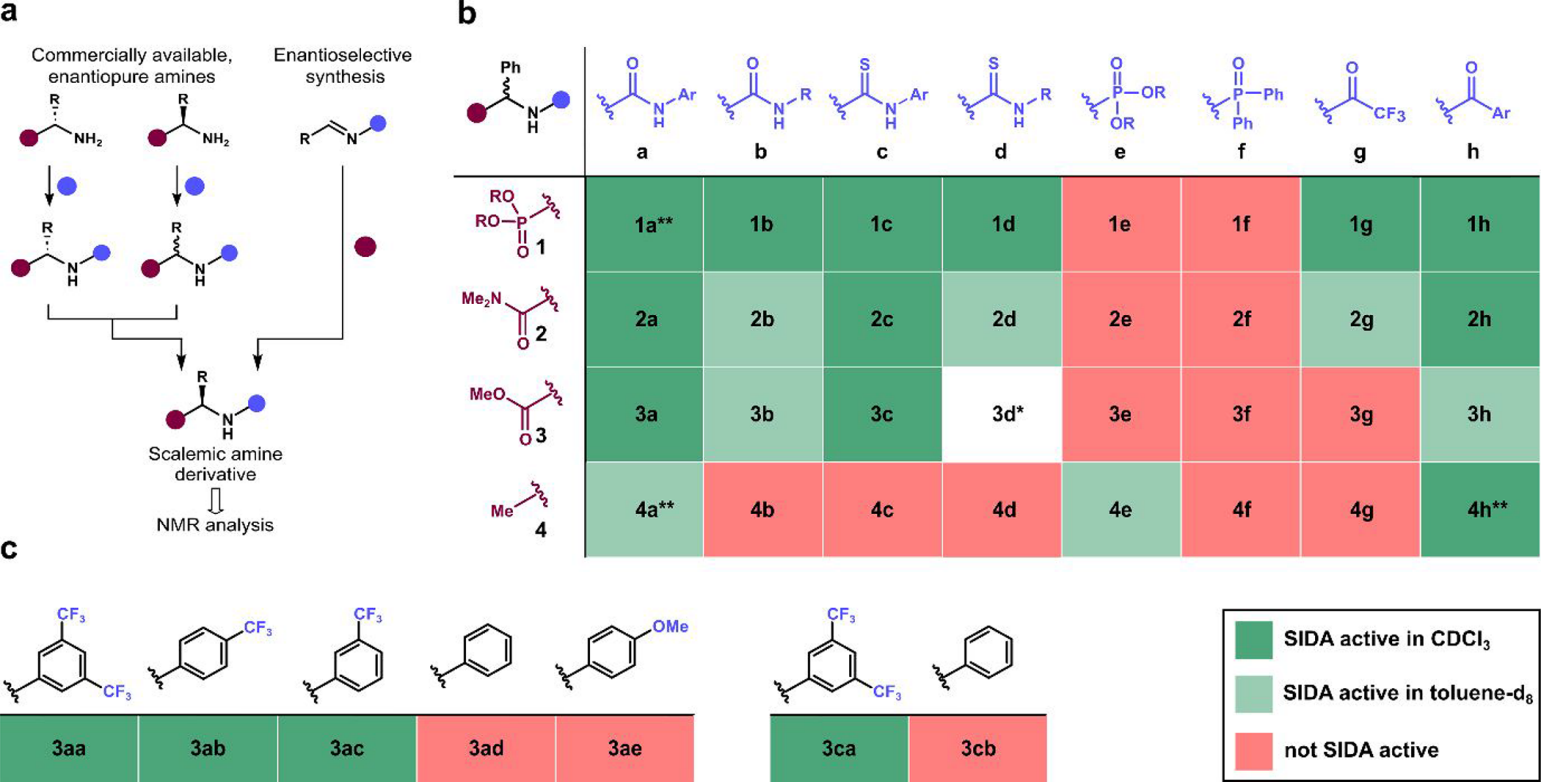
SAR approach. (a) Preparation of scalemic amines. (b) SAR table. NMR analysis was performed with ∼10 mg of scalemic substrate in CDCl_3_ or toluene-*d*_8_ (0.6 mL) at 25 °C. (c) Influence of electron-donating and electron-withdrawing substituents on the SIDA effect of **3a**/**3c** derivatives. *Compound **3d** could not be isolated. **Compounds whose SIDA effect has been reported (see main text).

By inspecting the first row of the SAR table (Figure 2b), we observed that α-amino phosphonates, featuring one of the strongest HBAs (HBA constant of phosphonate, β = 8.9),^22^ often led to SIDA-active compounds by functionalization of the amino groups with a wide range of HBDs, including aromatic and aliphatic (thio)­ureas (**1a−d**), trifluoroacetamides (**1g**), and aromatic amides (**1h**), in CDCl_3_ at 25 °C. Under these conditions, the enantiomeric purity of these derivatives could be easily determined by ^1^H-NMR, ^31^P-NMR, and ^19^F-NMR (when applicable), being evidence of a marked SIDA effect. In contrast, phosphoramidate **1e** and phosphinamide **1f** did not show a SIDA effect under a variety of conditions. Analogous results were obtained with α-amino amides (**2a−** **h**), where the amide moiety is also known to be an excellent HBA (β = 8.3).^23,24^ The generality of the SIDA effect diminished with α-amino esters (**3a−3h**), bearing a weaker HBA group (β = 5.2). Of particular note is the dependence of these α-amino esters on the substitution pattern (**3a** and **3c**, Figure 2c). While α-amino esters functionalized with electron-poor urea HBD groups, such as **3aa** to **3ac**, were SIDA-active, compounds **3ad** and **3ae**, possessing electron-rich aryl moieties, showed no SIDA activity in CDCl_3_. We attributed this to the increased HBD ability of ureas bearing electron-withdrawing groups, which might enhance their affinity for the ester group. Occasionally, HBD groups can also act as HBAs, such as amide groups in peptides. This was the case of the known 1-phenethylamine derivatives **4a** and **4h**,^25^ bearing urea and amide groups capable of undergoing self-association in CDCl_3_ at 25 °C. Curiously, their thiourea analogues **4b** and **4d** did not show SIDA effect, most likely due to the lower proton acceptor properties of the S atom compared to the O atom. Especially noteworthy is the case of phosphoramidates, which were previously studied in our group due to their ability to form homo- and heterochiral associates and undergo remarkable spontaneous fractionation of enantiomers in water.^9c^ Their SIDA effect remained unnoticed to us since they were exclusively analyzed in CDCl_3_ by NMR. However, when switching the solvent to toluene-*d*_*8*_, the SIDA effect became evident for phosphoramidate **4e**, both by ^1^H-NMR and by ^31^P-NMR (Figure 3a). Based on this result, we further explored the use of phosphoramidates as general self-resolving groups for chiral amines (Figure 3b). Notably, the SIDA effect could be observed in a variety of substituted 1-phenethylamines functionalized with the diisopropylphosphoramide group (**4e**, **5−7**), even in the presence of a competing HBD (−OH group in **7**). Conversely, no SIDA effect with the *p*-Br-aryl derivative **8** was observed under different conditions (vide infra for a rationalization), nor in phosphoramidate derivatives **9** and **10**.

This SAR analysis pointed out that many common organic building blocks, including α-amino phosphonates, α-amino amides, α-amino esters, and 1-phenethylamines, can display the SIDA effect under certain conditions. It also suggests that the SIDA effect might be more general than anticipated and that the combination of good HBDs and HBAs in the proximity of a stereogenic center often leads to SIDA-active compounds.

**Figure 3:**
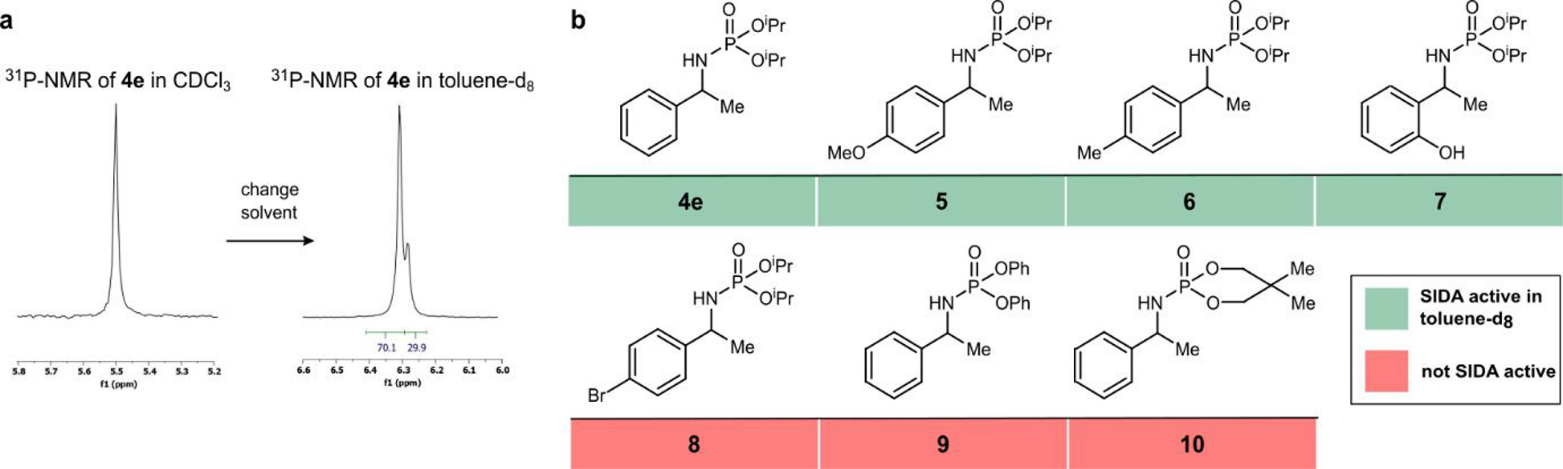
SAR analysis of the phosphoramidates. (a) Influence of substitution pattern on the SIDA effect. NMR analysis was performed with ∼10 mg of scalemic substrate in toluene-*d*_8_ (0.6 mL) at 25 °C. (b) ^31^P-NMR spectrum of **4e** (70:30 e.r.) in CDCl_3_ (left) and toluene-*d*_8_ (right) at 25 °C.

### Unveiling the Origin of the SIDA Effect: In Solution vs Solid-State Analysis

As stated before, the SIDA effect is a natural consequence of the intermolecular association of enantiomers in solution that leads to homochiral and heterochiral complexes. But what is the nature of those associates? Dimers or higher-order aggregates? Why can subtle variations of the structure sometimes lead to a SIDA-inactive compound? Earlier work on the SIDA effect had seldom addressed these questions, and evidence of association is, in most cases, only supported by X-ray diffraction analysis of compounds in the crystalline state. While solid-state analysis can offer valuable information on the nature of the intermolecular interactions and the distinct 3D-arrangement of the homo- and heterochiral complexes, the nature of the aggregates in solution might significantly differ from that in the solid state. In our previous study,^19^ we discovered that the combination of diffusion-ordered spectroscopy (DOSY) and solid-state analysis can provide a reliable description of the nature of the supramolecular complexes involved, allowing the estimation of the molecular weight of the aggregates in solution, and, hence, their composition.^26^

To gain insights into the origins of the SIDA effect and further expand the repertoire of SIDA-active compounds, we performed a comparative study between the reported solid-state data of compounds related to those from Figure 2 and their speciation in solution using DOSY NMR analysis (Figure 4). As a calibration point, we started our investigation with Jacobsen’s chiral α-thioureidoamide **11**, a well-known enantioselective organocatalyst for a variety of transformations.^27^ Given its similarity to compounds **2a−** **d**, we hypothesized that **11** should also be SIDA-active. Indeed, an 85:15 mixture of *R*/*S* enantiomers of **11** in CDCl_3_ showed two sets of peaks in ^1^H-NMR whose relative integration matched the expected enantiomeric ratio (85:15, see the Supporting Information). Importantly, the self-association of these α-thioureidoamides was extensively investigated by Jacobsen and coworkers, both in solution and in the solid state, observing a strong tendency to form dimeric complexes.^27b^ We further examined compound **11** by DOSY NMR under the conditions where it showed the SIDA effect (in CDCl_3_) and where the SIDA effect disappears (in DMSO-*d*_*6*_). On the one hand, in CDCl_3_, a diffusion coefficient of *D* = 5.8 × 10^−10^ m^2^/s was measured, which corresponds to an estimated molecular weight of *M*_W_(estimated) = 1296 g/mol, suggesting the formation of a dimeric species (*M*_W_(expected) = 1163 g/mol)_._ On the other hand, in DMSO-*d*_*6*_, a diffusion coefficient of *D* = 1.8 × 10^−10^ m^2^/s was obtained, corresponding to an *M*_W_(estimated) of 713 g/mol, in good agreement with the presence of the monomeric species (*M*_W_(expected) = 581 g/mol). These observations indicate that the origin of the SIDA effect in α-thioureidoamides is most likely due to the formation of dimers in solution.

We continued our comparative study with alanine derivative **12**, whose remarkable SIDA activity in C_6_D_6_ was observed by Trapp and coworkers.^18^ The reported X-ray diffraction analysis of this compound revealed linear polymeric structures. However, as pointed out by the authors, the structure in solution can deviate considerably from that in the solid state. Indeed, DOSY NMR analysis of a 50 mM solution of **12** in toluene-*d*_*8*_ at 25 °C (where the compound also showed SIDA effect) featured two sets of signals that pointed to the coexistence of monomeric and dimeric species, with a tendency toward the monomeric form. By a fivefold increase of the concentration, the equilibrium could be shifted toward the dimeric form (see the Supporting Information). No other higher-order aggregate could be detected.

**Figure 4:**
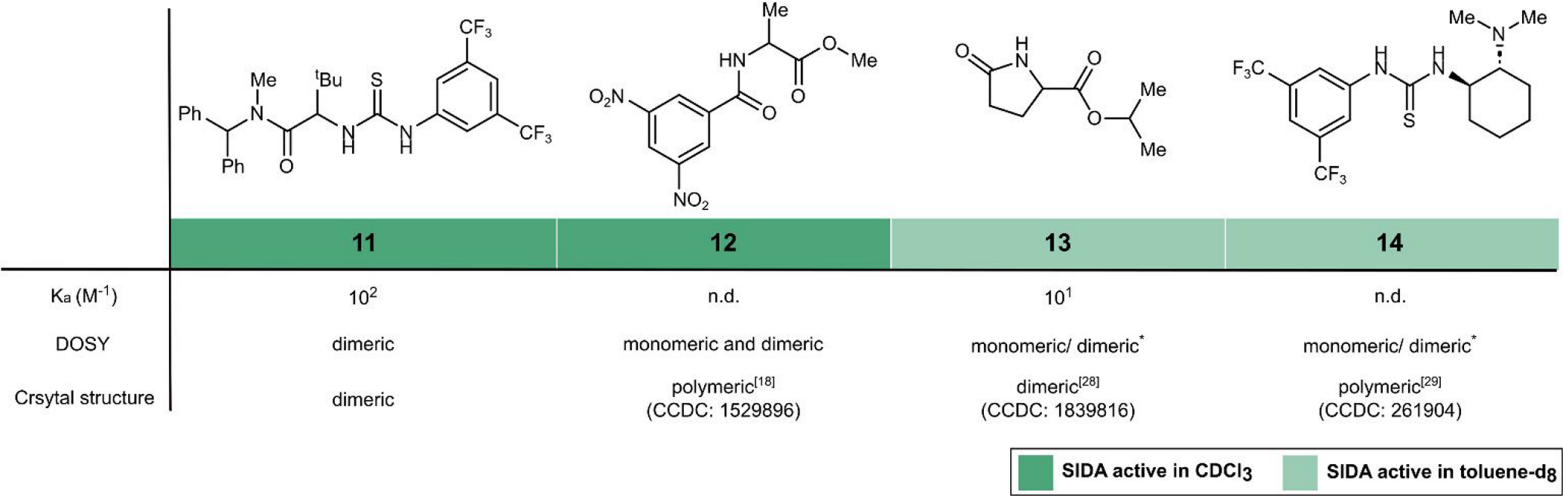
Overview of the comparative structural study by DOSY NMR and reported solid-state data. *Concentration-dependent.

Further evidence supporting a preference for dimeric association in SIDA-active compounds was found with lactam **13** and Takemoto’s catalyst **14**, compounds closely related to those explored in our SAR study. While, to the best of our knowledge, the SIDA activity of these well-known compounds has never been reported, **13** and **14** showed noticeable SIDA effect in toluene-*d*_*8*_ (see the Supporting Information). The solid-state structure of **13** clearly indicated the preference for dimeric species.^28^ DOSY NMR analysis revealed a concentration-dependent shift between monomeric and dimeric species (see the Supporting Information). Analogous results were obtained with **14**.^29^ Once again, no other higher-order aggregates were observed in solution for these compounds.

One of the most puzzling aspects of the SIDA effect is why, occasionally, subtle modifications of the structure can “turn off” the SIDA effect. This is the case for phosphoramidates **4e** (SIDA-active) (Figures 2 and 3) and **8** (SIDA-inactive) (Figure 3). In our previous report, we also noticed that, while **4e** led to the spontaneous fractionation of enantiomers in water, **8** did not show this phenomenon.^9c^ The only difference between both structures is a Br atom in an apparently innocent position. However, by comparing the solid-state structures of **4e** and **8**, we observed that the introduction of the Br atom disrupts the self-complementary hydrogen-bond association of the phosphoramidate group. The racemic crystal structure of **4e** (racemate, CCDC: 1040052) consisted of dimers in which two phosphoramidates are linked in an antiparallel arrangement through H-bonds between the N−H group of one molecule and the P=O group of its partner (Figure 5a, *d*(N−H···O=P) = 2.850 Å). In contrast, the X-ray structure of racemic **8** (conglomerate, CCDC: 2059102) showed a noncovalent halogen bond between the Br atom and the P=O moiety (Figure 5b, *d*(Br···O=P) = 3.336 Å). This intermolecular halogen bond seems to affect the relative disposition of the N−H and P=O groups, preventing an antiparallel arrangement and the formation of dimeric entities. Although evidence for such a subtle effect in solution could not be confirmed, it is conceivable that the introduction of certain functionalities can indeed compete with the required dimerization and, hence, the observation of the SIDA effect. A similar conclusion could be drawn when comparing the X-ray structures of phosphoramidate **4e** and phosphinamide **4f** (Figure 5c, CCDC: 265442, SIDA-inactive).

**Figure 5:**
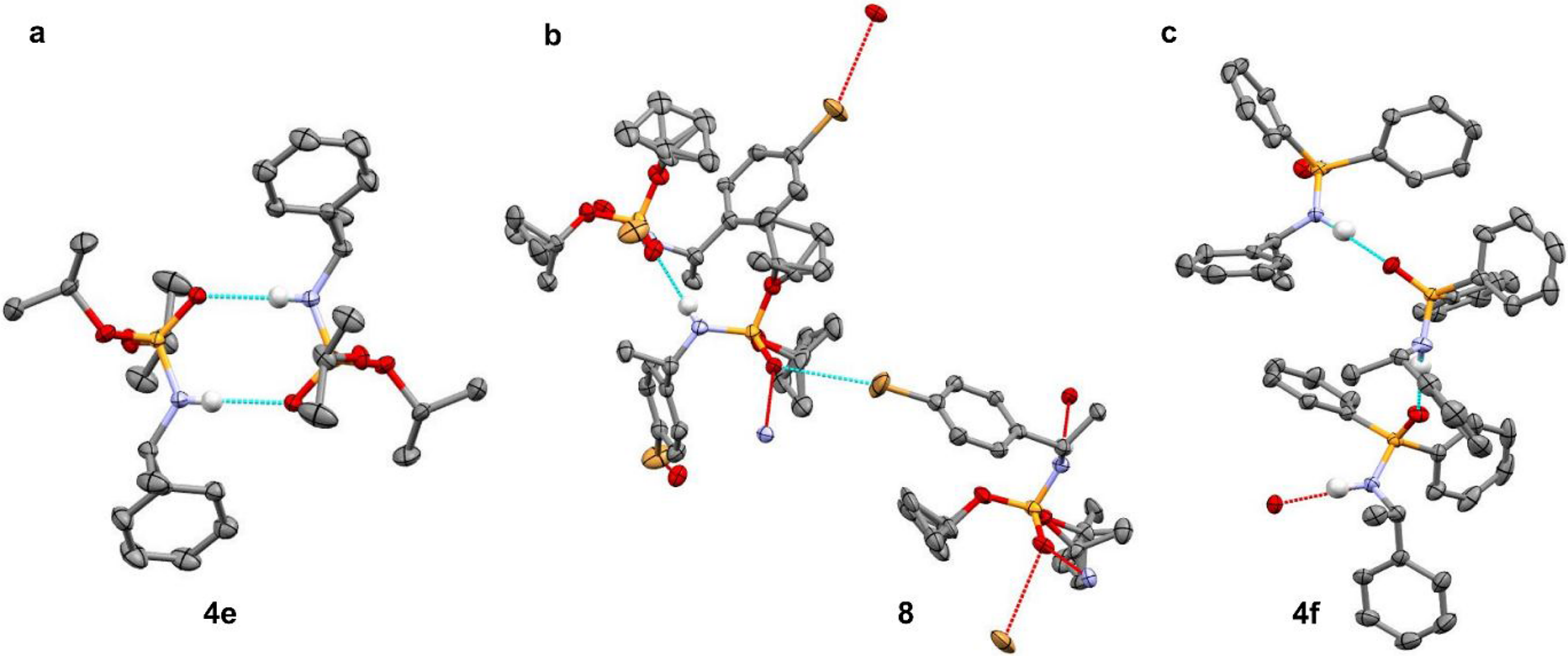
Crystal structure analysis of the phosphoramidates. (a) Dimeric structure of **4e** in the solid state, with hydrogen bonds highlighted (dashed blue lines). (b) Polymeric structure of **8** in the solid state with the hydrogen bonds highlighted (dashed blue lines) and halogen bonds (dashed red lines). (c) Polymeric structure of **4f** in the solid state, with the hydrogen bonds highlighted (dashed blue lines). Hydrogen atoms are omitted for clarity.

**Figure 6:**
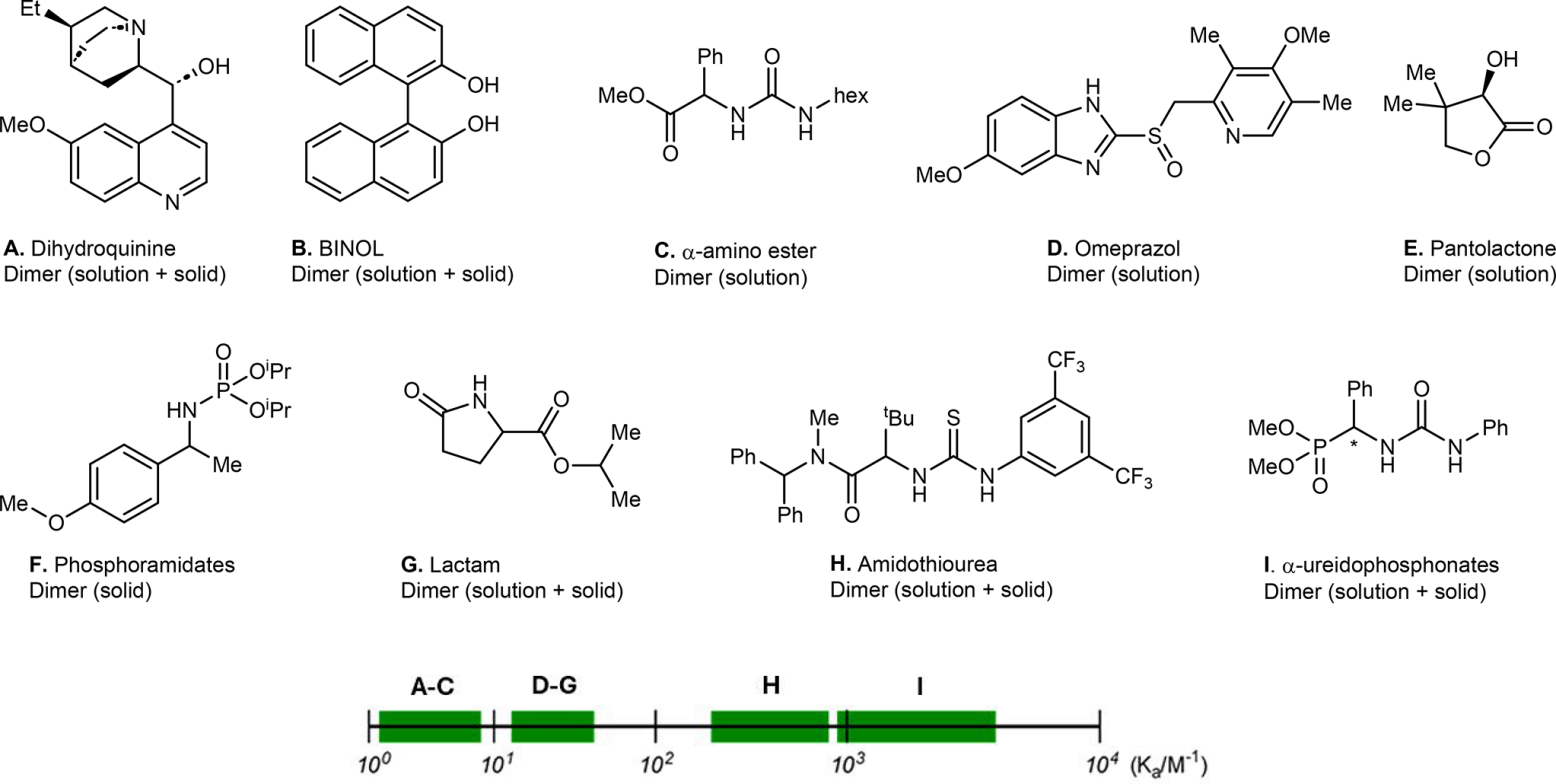
List of association constants (*K*_a_) for the SIDA-active compounds.

### Does the SIDA Effect Require a High Association Constant?

By definition, the SIDA effect is a manifestation of self-association, but how strong does this association need to be? Clearly, the solvent and concentration are critical to promoting solute−solute interactions, yet a strong self-association might not be required. To shed some light on these parameters, we collected all values for association constants (*K*_a_) available in the literature for SIDA-active compounds (Figure 6, structures **A**,^30^ **B**,^31^ **D**,^32^ **E**,^33^ **H**,^27b^ **I** ^19^) and also measured those for some selected compounds included in our SAR study (Figure 6, structures **C**, **F**, **G**; see the Supporting Information for details).

For the collection of SIDA-active compounds depicted in Figure 6, the wide range of association constants, spanning from 10^0^ to 10^3^ M^−1^, indicates that the strength of the association does not play an important role. This also applies not only to amine derivatives but also to compounds such as BINOL **B** or pantolactone **E**.

### Critical Role of the Solvent

As mentioned earlier, the SIDA effect is strongly solvent-dependent, and as a consequence, the choice of solvent is of paramount importance. It has been suggested that the use of low-polarity solvents, such as toluene-*d*_*8*_, C_6_D_6_, or CCl_4_, favors solute−solute interactions and, hence, the observation of the SIDA effect. However, the solubility of many potentially SIDA-active compounds might be compromised in these solvents. Moreover, we found some discrepancies when trying to relate the solvent polarity (using the dielectric constant ε) to the SIDA effect for some compounds, such as α-amino ester **12** (Figure 7a). By using no-D NMR spectroscopy and solvent suppression routines,^34^ we observed that **12** did not show a SIDA effect in 1,4-dioxane (ε = 2.21) or diethyl ether (ε = 4.2), which are less polar than CHCl_3_ (ε = 4.89) or DCE (ε = 10.65), where **12** proved to be SIDA-active. We anticipated that the appearance of the SIDA effect might be more affected by the presence or absence of HBDs and HBAs in the solvent than the polarity. To examine this in detail, we used noncovalent interaction parameters α and β, which are related to the H-bond donor and acceptor sites, respectively.^35^ With these values, a generalized functional group interaction profile (FGIP) can be derived,^22,36^ illustrating the free energy landscape for the pairwise functional group interactions between two solutes (α_solute_ and β_solute_) in a solvent (α_solvent_ and β_solvent_) that can be partitioned into four quadrants (Figure 7b). Applied to the SIDA effect, the top-right quadrant (highlighted in green) delimits the region where solute−solute interactions are favored, and hence, the SIDA effect is possible. In the other quadrants, highlighted in red, solute−solvent (top left and bottom right) and solvent−solvent (bottom left) interactions are favored, indicating that the SIDA effect is unlikely.

**Figure 7:**
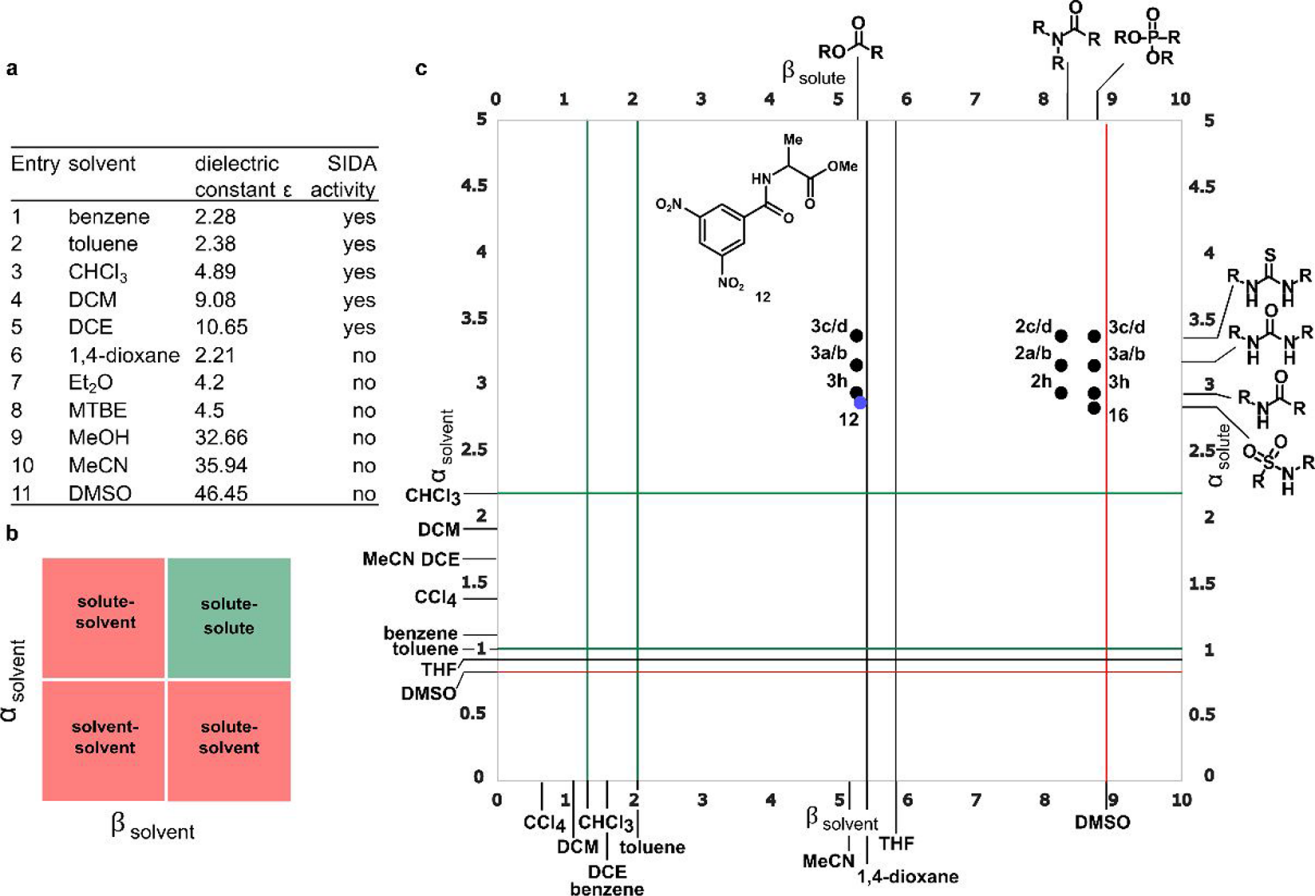
Influence of solvent polarity and its HBD/HBA properties on the SIDA effect. (a) Solvent polarity vs. SIDA effect for **12**. (b) General FGIP interpretation. (c) FGIP with α and β values for commonly used solvents and functional groups. Black dots, studied amine derivatives; blue dot, **12**. α and β values were calculated for THF and 1,4-dioxane (black lines), CHCl_3_/toluene (green lines), and DMSO (red lines).

By representing the α and β values of the most common solvents against the α and β values of SIDA-active compounds (Figure 7c), we can intuitively identify which solvents might favor solute−solute interactions. Those compounds lying on the top-right quadrant delimited by a specific solvent are expected to show a SIDA effect. This is, for instance, the case of α-amino phosphonates **1a**/**b**/**c**/**d**/**h** and **16**, α-amino amide **2a**/**b**/**c**/**d**/**h**, and α-amino esters **3a**/**b**/**h** and **12**, in CHCl_3_, toluene, benzene, or DCM as solvents. On the contrary, these compounds lie on the top-left quadrant delimited by DMSO, indicating that solvent−solute interactions are favored and the SIDA effect is unlikely in this solvent. For α-amino ester derivatives **3a**/**b**/**h** and **12**, we can also observe that, given the lower HBA properties of the ester group, solvents such as THF or 1,4-dioxane can outcompete solute−solute interactions, indicating that the SIDA effect is unlikely in these solvents. This is in perfect agreement with the experimental results and suggests that FGIPs can provide good guidance when selecting a solvent to study chiral self-recognition phenomena. However, it is important to note that factors such as concentration and temperature can also play an important role since high concentrations and low temperatures usually favor solute−solute interactions. Importantly, for the purpose of studying intermolecular association by NMR, the use of nondeuterated solvents together with solvent suppression techniques seemed to represent a viable alternative to traditionally used deuterated solvents, resulting in a considerable cost reduction.

### Accurate Determination of Enantiomeric Purities

A key advantage of the SIDA effect is the direct determination of the enantiomeric purities by NMR without requiring any external chiral source or specialized equipment. In many cases, the accuracy of this approach is comparable to that of traditional chiral chromatographic techniques. A number of examples showcasing this high level of accuracy can be found in the literature, especially when a good splitting of the signals ensures unequivocal integration of the peaks (the peaks are well resolved). However, in cases with poor splitting of the signals, integration might be challenging, particularly at low enantiomeric purities, where the peaks tend to merge. In Figure 9, we highlight two cases showing both situations. Compound **2a** (Figure 8a) exhibited a good splitting of the signals, by both ^1^H-NMR and ^19^F-NMR, and the determination of the enantiomeric ratio can be performed for a broad range of enantiomeric purities with high accuracy. In contrast, compound **5** showed poor splitting of the signals by ^1^H-NMR and ^31^P-NMR (Figure 8b,c). However, by applying quantitative Global Spectral Deconvolution (qGSD), included in software packages such as MestReNova®, high-quality integration analysis could also be obtained, with results comparable to chiral HPLC analysis, except for enantiomeric ratios below 60:40, where both sets of peaks merged.^37^

**Figure 8:**
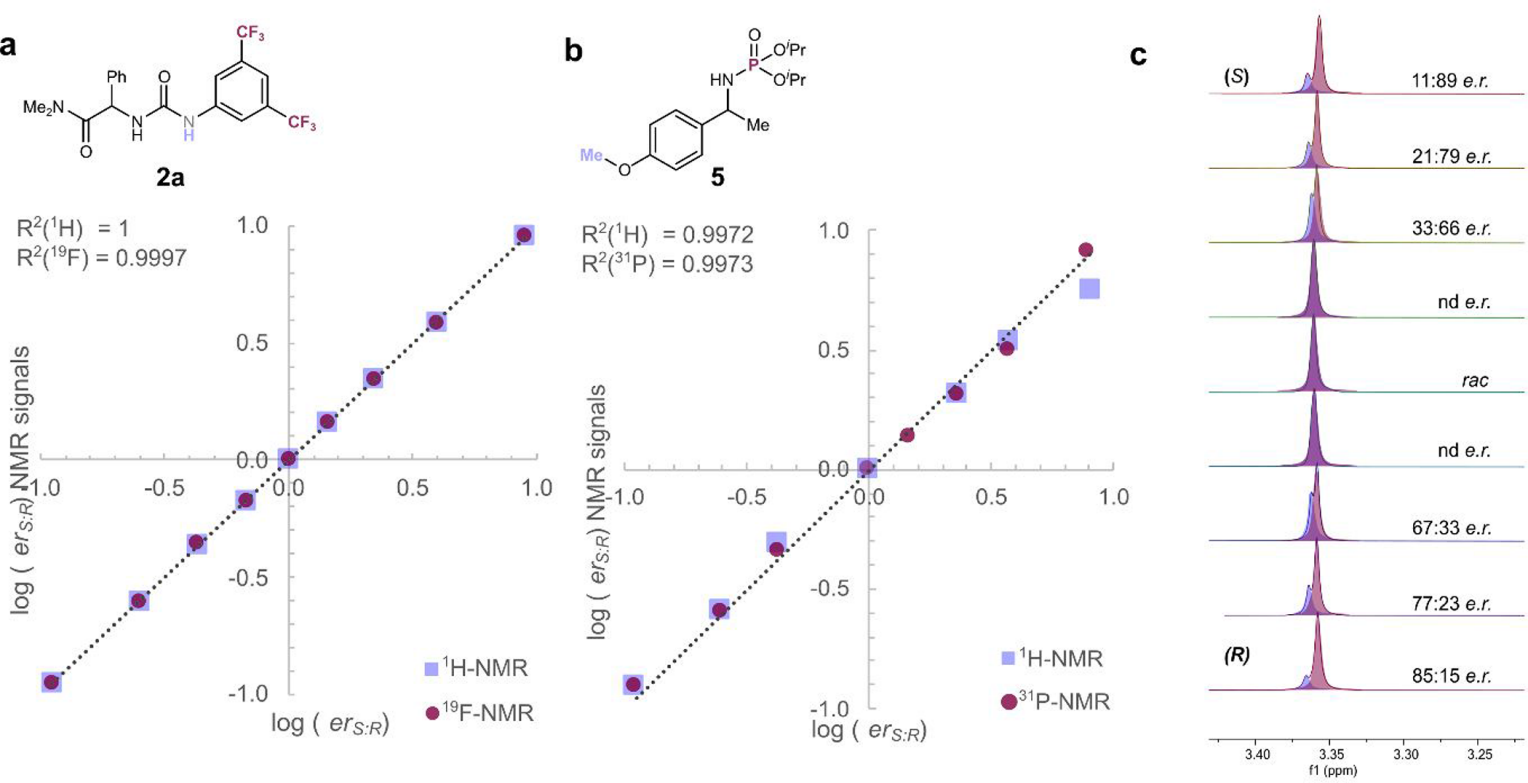
Accuracy of the SIDA effect for the determination of enantiomeric purities. (a) Comparison of the enantiomeric ratios of **2a** determined by ^1^H-NMR/^19^F-NMR vs chiral HPLC analysis. (b) Comparison of the enantiomeric ratios of **5** determined by ^1^H-NMR/^31^P-NMR vs chiral HPLC analysis. (c) ^31^P-NMR spectra of **5** with different enantiomeric purities (qGSD was applied). Monitored nuclei are highlighted in light blue (^1^H-NMR) and maroon (^19^F-NMR and ^31^P-NMR).

A remarkable example was found with α-amino phosphonate **15** (Figure 9a), an analogue of the plant antiviral agent Dufulin.^38^ We discovered that this compound, closely related to the α-amino phosphonates explored in our SAR study (Figure 2), also showed a SIDA effect in toluene-*d*_8_. The remarkable splitting of the ^31^P-NMR signals enabled us to determine enantiomeric ratios as low as 51:49 (Figure 9b), matching the results obtained by chiral HPLC analysis (Figure 9c).

At this point, we envisaged that the SIDA effect would become particularly useful in the analysis of complex reaction mixtures. In principle, simple NMR analysis of a crude mixture would allow the simultaneous determination of conversions and enantiomeric purities in asymmetric transformations. If so, the SIDA effect can significantly accelerate optimization of the reaction conditions. Although we have already developed a basic application of this concept for a rationally designed system,^19^ we speculated whether it could be extended to other known asymmetric reactions, thus generalizing the principle. To illustrate this, we selected two known enantioselective transformations leading to potentially SIDA-active compounds (Figure 10). On the one hand, we discovered that α-sulfonamidophosphonates such as **16**, a well-explored family of α-aminophosphonate derivatives that crystallize as dimers,^39^ showed the SIDA effect in toluene-*d*_*8*_. Once again, their self-enantiorecognition properties probably went unnoticed as they have been exclusively characterized in CDCl_3_ by NMR, where they do not show a SIDA effect. The enantioselectivity and conversion for the enantioselective hydrophosphonylation reaction toward α-sulfonamidophosphonates could be easily monitored by ^1^H-NMR and ^31^P-NMR analysis of the crude mixture, without requiring deuterated toluene (Figure 10a−d).

**Figure 9:**
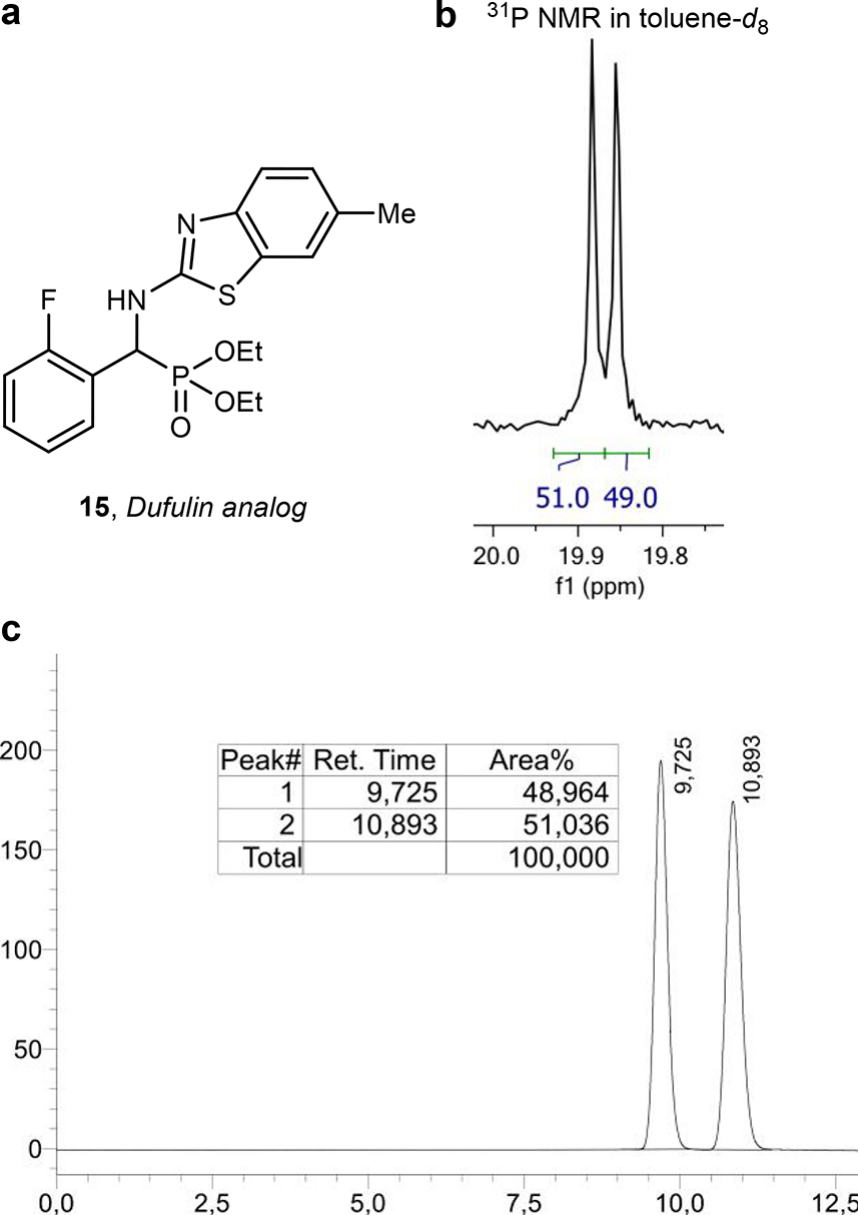
(a) Structure of **15**. (b) ^31^P-NMR of **15** in toluene-*d*_8_. (c) Chiral HPLC chromatogram of **15**.

**Figure 10:**
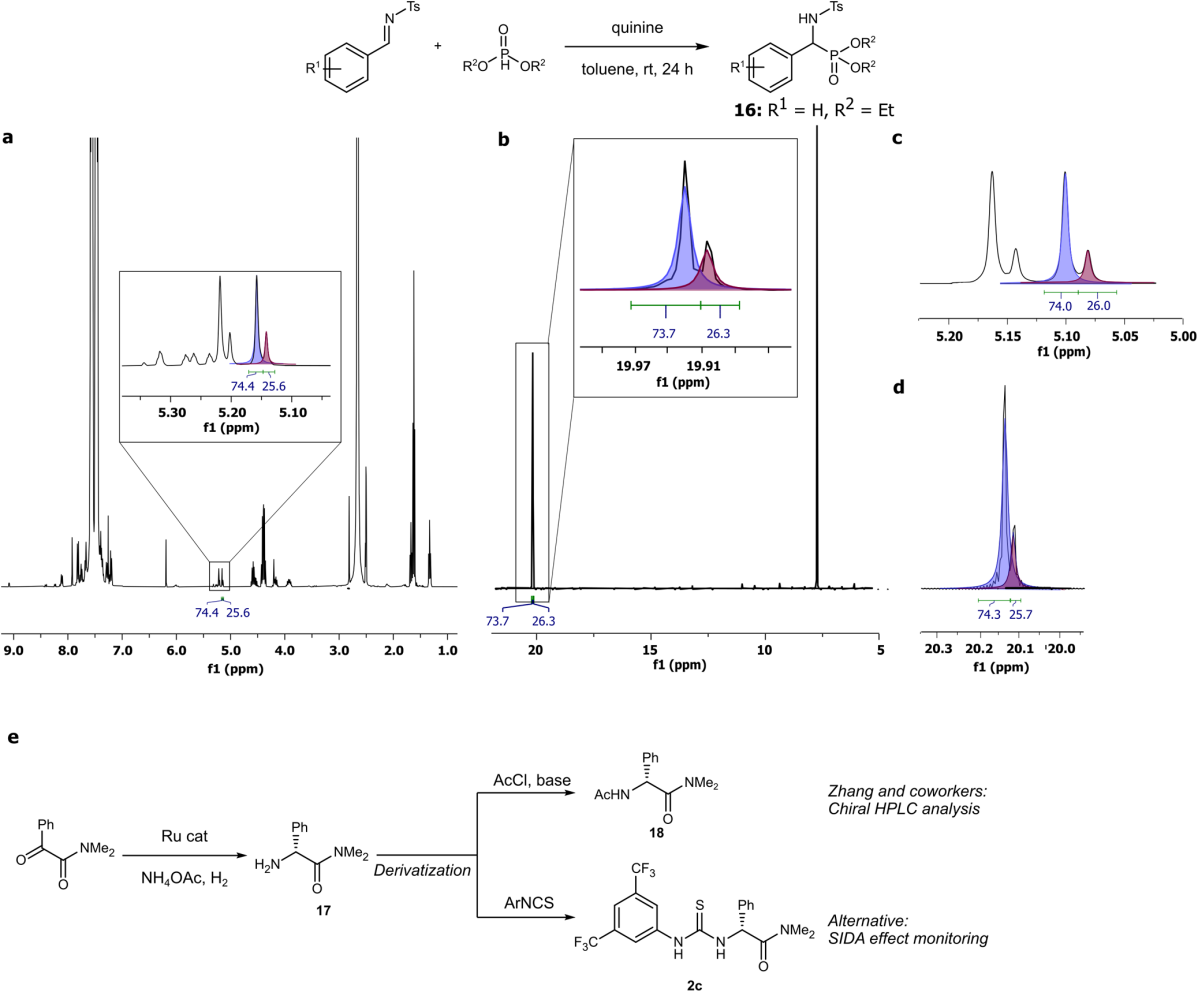
Application of the SIDA effect in analysis of the enantiomeric composition in complex mixtures. (a, b) Determination of the enantiomeric purity of **16** in the crude mixture by ^1^H-NMR and ^31^P-NMR, respectively, using nondeuterated toluene and peak deconvolution. (c, d) ^1^H-NMR and ^31^P-NMR spectra, respectively, of pure **16** in toluene-*d*_8._ (e) Determination of enantiomeric purity via derivatization into a SIDA-active compound as an alternative to chiral HPLC analysis.

Notably, the SIDA effect was also present in a variety of α-sulfonamidophosphonate derivatives (see the Supporting Information). On the other hand, we could also apply this method to the enantioselective synthesis of amino acid derivatives via asymmetric reductive amination recently reported by Zhang and coworkers (Figure 10e).^40^ The α-amino amides obtained by this method required in situ derivatization for the analysis of the enantiomeric purity by chiral HPLC. Alternatively, we found that the enantioselectivity of this transformation could be easily monitored by NMR through in situ derivatization with an isothiocyanate, leading to compounds such as **2c**, which showed a SIDA effect in several solvents (see Figure 7). Overall, when compared to other analytical protocols, the SIDA effect benefits from a faster determination of enantiomeric purities (<5 min) and significantly lower amounts of solvents (<1 mL).

### SIDA Effect as a Mechanistic Tool

The nonlinear effect test (NLE test) has been routinely used in asymmetric catalysis to study the nature of the catalytically active species (monomeric or higher order). In this test, the presence of (+)-NLE or (−)-NLE when using a scalemic version of the catalyst is often regarded as proof of its aggregation. However, several authors have recently raised some concerns on this generalization, since NLE can occasionally be observed in the absence of aggregates,^41^ as well as linear relationships between catalyst and product enantiopurities can be detected in spite of catalyst aggregation.^42^ In this regard, we envisioned the use of SIDA effect detection as more direct evidence of catalyst aggregation. Given that the SIDA effect can only take place by formation of homo- and heterochiral associates (most likely dimers), it can potentially be used as a probe to identify off-cycle, high-order species derived from the catalyst. In fact, as shown earlier, we could identify several chiral amine derivatives that have been widely used as organocatalysts in asymmetric synthesis, but whose SIDA effect had not been reported (Jacobsen’s thiourea catalyst **11** and Takemoto’s catalyst **14**). To these newly described examples of SIDA-active compounds, we can also add dihydroquinine (**A**, Figure 6), a well-known organocatalyst whose aggregation in solution has been documented.^13,30^ These catalysts have shown NLE in certain transformations as a result of the formation of homochiral and heterochiral off-cycle associates. Curiously, a simple ^1^H-NMR analysis of a scalemic mixture of these catalysts can provide analogous information. It is important to note that, however, the detection of the SIDA effect might not be as generally applicable as the NLE test, nor does it provide evidence of the formation of high-order species in the enantio-determining transition state of an asymmetric reaction. Still, the simplicity and inexpensive nature of the SIDA effect test can be useful to quickly identify high-order species in organocatalytic reactions. The fact that many well-known chiral organocatalysts feature HBDs and HBAs in close proximity to stereogenic centers in their structures suggests that the SIDA effect test might be applicable in other systems alike.

## Conclusions

We have performed a SAR study on chiral amine derivatives to uncover the stereochemical parameters that lead to the observation of the SIDA effect by NMR. By cross-checking new and previous data, we revealed which combinations of functional groups, featuring HBDs and HBAs, are prone to give SIDA-active compounds. A combination of solid-state analysis and DOSY NMR studies allowed us to delve into the origins of the SIDA effect, observing that dimeric homo- and heterochiral complexes are the most likely form of association, although higher-order aggregates cannot be discarded in cases not included in this study. In addition, we confirmed that the presence of self-complementary HBD and HBA groups in close proximity to a stereogenic center can often lead to SIDA-active compounds, with this being dependent on the strength of the intermolecular interaction. As a rule of thumb, a good HBA (e.g., phosphonates or amides) can be recognized by a wider range of HBDs (e.g., (thio)­ureas, amides, or sulfonamides). In relation to this, as the SIDA effect is a manifestation of intermolecular interactions between chiral molecules, the nature of the solvent plays a critical role. While previous studies pointed to the polarity of the solvent as an important aspect to consider when analyzing potential SIDA-active compounds by NMR, we observed that the presence/absence of certain HBD/HBA groups in the solvent is more critical. In this regard, the use of FGIPs can be helpful in the identification of a suitable solvent. Moreover, we have demonstrated that the SIDA effect can be a time- and cost-effective analytical tool for the determination of enantiomeric purities (<1 mL of solvent, possibility of using non-D-NMR, <5 min analysis time), even in complex mixtures, making it suitable for future applications in high-throughput experimentation and complementing other well-established techniques. We have also identified the SIDA effect as a potential mechanistic probe to study catalyst aggregation in a series of organocatalysts, thus serving as a complementary approach to the standard NLE test.

Overall, in this study, we have identified >35 amine derivatives showing SIDA effect by NMR, representing an approximate 40% increase in the total number of SIDA-active compounds reported since the discovery of this effect in 1969. This fact suggests that the phenomenon might be more common than previously anticipated. Finally, this work provides a basis to address chiral self-recognition processes by design, as well as opening new avenues for the analysis of scalemic mixtures in asymmetric synthesis and the study of intermolecular interactions in supramolecular chemistry.

## Supplementary Material



## Notes

Experimental procedures, characterization of new compounds, structural data, NMR experiments, and HPLC chromatograms (PDF)

## †

Department of Chemistry, University of Cambridge, Cambridge CB2 1EW, U.K

## Abbreviations

DOSY: diffusion-ordered spectroscopy
FGIP: functional group interaction profile
HBA: hydrogen-bond acceptor
HBD: hydrogen-bond donor
qGSD: quantitative global spectra deconvolution
NLE: nonlinear effect
SAR: structure−activity relationship
SIDA: self-induced diastereomeric anisochronism
