# Supplementary material for: Toward Chiral Recognition by Design: Uncovering the Self-Enantioresolving Properties of Chiral Amine Derivatives
Source: J Am Chem Soc. 2025 May 23;147(22):18662–73. doi: 10.1021/jacs.5c01251 (PMC12147147; doi:10.1021/jacs.5c01251)
Supplement: Supplementary file 1 [file ja5c01251_si_001.pdf]

Supporting Information

Towards Chiral Recognition by Design. Uncovering the Self-Enantioresolving Properties of Chiral Amine Derivatives.

Anka Hagelschuer, Damián Padín,*† Vanda Dašková, Ben L. Feringa*

Stratingh Institute for Chemistry, University of Groningen, Nijenborgh 4, 9747 AG Groningen, The Netherlands.

Corresponding author e-mail: dp721@cam.ac.uk

Corresponding author e-mail: b.l.feringa@rug.nl

1. Table of Contents

2. General experimental procedures S3
3. Dimerization studies S4
3.1. Solution studies - 2D-DOSY NMR in $CDCl_3$/toluene-d_8 and DMSO-d_6 ..... S4
3.2 Solid state analysis S11
3.3 Association constants S14
4. Studies on the accuracy of the SIDA effect compared to chiral HPLC S23
4.1 Comparison NMR vs HPLC S23
4.2 Peak deconvolution S26
5. Solvent study S27
5.1 NMR spectra using solvent suppression S27
5.2 Functional group interaction profile S34
6. Synthetic procedures S35
6.1 Compounds prepared following literature procedures S35
6.2 Synthesis and characterization of α-amino phosphonates S35
General Procedure **A**: S35
6.3 Synthesis and characterization of α-amino amides S38
General Procedure **B** for the synthesis of α-amino amine **S2**: S38
General procedure **C** for the synthesis of α-amino amide **2a – 2h**: S38
6.4 Synthesis and characterization of α-amino ester 3a – 3h S43
General procedure **D** for the synthesis of α-amino esters S43
6.5 Synthesis and characterization of 1-phenethylamines 4a – d and 4f – 4h S48
General procedure **E** for the synthesis of 1-phenethylamines **4a – d** and **4f – 4h** S48
6.6 Synthesis and characterization of 11-15, S3 ..... S53
6.7 Synthesis and characterization of α-amino phosphonates 16, S4-S7 ..... S57
General procedure **F** for the synthesis of α-amino phosphonates S57
7. *e.r.* determination by NMR and/or HPLC S60
8. Overview of all compounds tested for SIDA activity S100
9. Additional compounds tested for SIDA activity S101
10. NMR spectra S102
11. References S189

2. General experimental procedures

All reagents were purchased from commercials sources and used without purification. Oven-dried or heat gun-dried glassware was used for all experiments carried out under N_2 atmosphere. THF, diethyl ether, toluene and DCM were obtained dried by a MBRAUN solvent purification system (SPS). When specified, reactions were monitored by analytical thin layer chromatography on silica-coated aluminum plates (silica gel 60 F254 Merck) and components were visualized by UV light and $KMnO_4$ staining (1.5 g $KMnO_4$, 10 g K_2CO_3, 1.25 mL 10% NaOH, 200 mL H_2O). Flash column chromatography was performed on silica gel 60 (Merck, 230-400 mesh) or by automated column chromatography (Biotage® Selekt System with Biotage® Sfär Silica cartridges).

^{1}H-NMR, ^{13}C-NMR, ^{19}F-NMR and ^{31}P-NMR measurements were recorded on a Varian AMX400, Varian Oxford AS 500 MHz and Bruker Innova 600 MHz spectrometers. Chemical shift values are reported in ppm with the residual solvent resonances as the internal standards. Coupling constants (*J*) are given in Hertz (Hz). Multiplicities are reported as follows: s = singlet, d = doublet, t = triplet, q = quartet, p = pentet, m = multiplet, or as a combination of them.

MestReNova® software was used for the analysis of NMR spectra and the default quantitative Global Spectral Deconvolution (qGSD) included in this software package (based on generalized Lorentzian function) was applied for quantification of enantiomeric excess by NMR for those compounds showing partially overlapping peaks.

For the indicated cases, enantiomeric ratios were determined by NMR using the SIDA effect in the indicated solvent. Alternatively, and for selected cases, enantiomeric excesses were determined by HPLC analysis using a Shimadzu LC-10ADVP HPLC equipped with a Shimadzu SPDM10AVP diode array detector using Chiralpak columns with the indicated mixtures of HPLC-grade *n*-heptane and 2-propanol as eluents and a column temperature of 40 °C. Sample injections were made using a HP 6890 Series Auto sample Injector.

Mass spectra were recorded on an AEI-MS-902 mass spectrometer (EI+) or a LTQ Orbitrap XL (ESI+, ESI-, APCI+).

3. Dimerization studies

3.1. Solution studies - 2D-DOSY NMR in $CDCl_3$/toluene-d_8 and DMSO-d_6

2D-DOSY NMR shows the influence of the solvent on the dimer-monomer equilibrium of SIDA active compounds, resulting in different diffusion coefficients. The values for the diffusion coefficients (D) were obtained by 2D-DOSY NMR using a 600 MHz instrument at 25 °C in the indicated solvents, using a scalemic mixture of the indicated compound (Figures S1-S13). The values obtained for the diffusion coefficients were related to the estimated molecular weight (M_w) using the Stokes-Einstein Gierer-Wirtz estimation.[1] The estimated molecular weight was obtained by using the MestReNova® software to process the DOSY NMR spectra.

DOSY NMR experiments were performed by dissolving ~10 mg of the indicated scalemic compound (~80:20 *e.r.*) in the indicated solvent (0.6 mL).

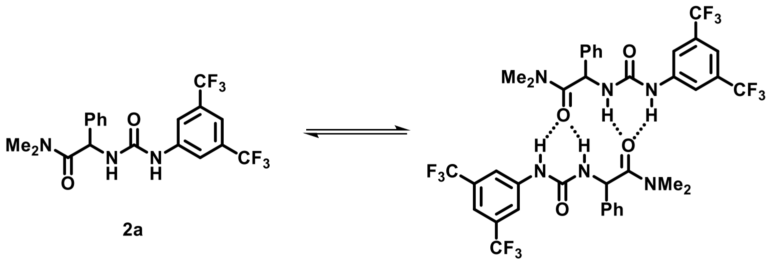

Molecular Weight: 433.35 g/mol

Molecular Weight: 866.7 g/mol

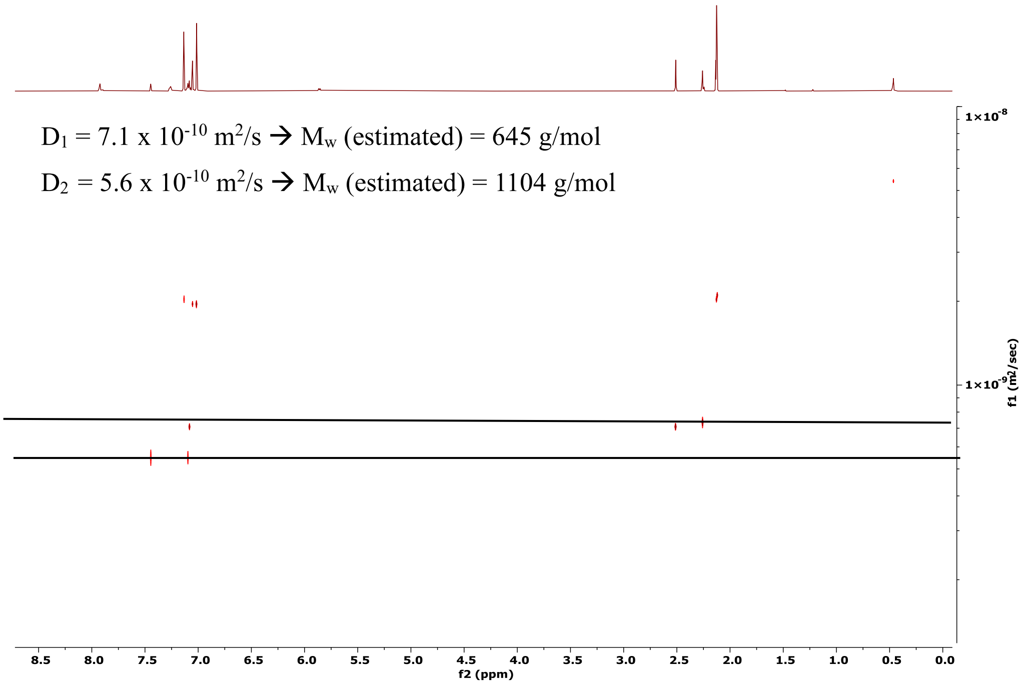

Figure S1: 2D-DOSY NMR in toluene-d_8 of scalemic **2a**.

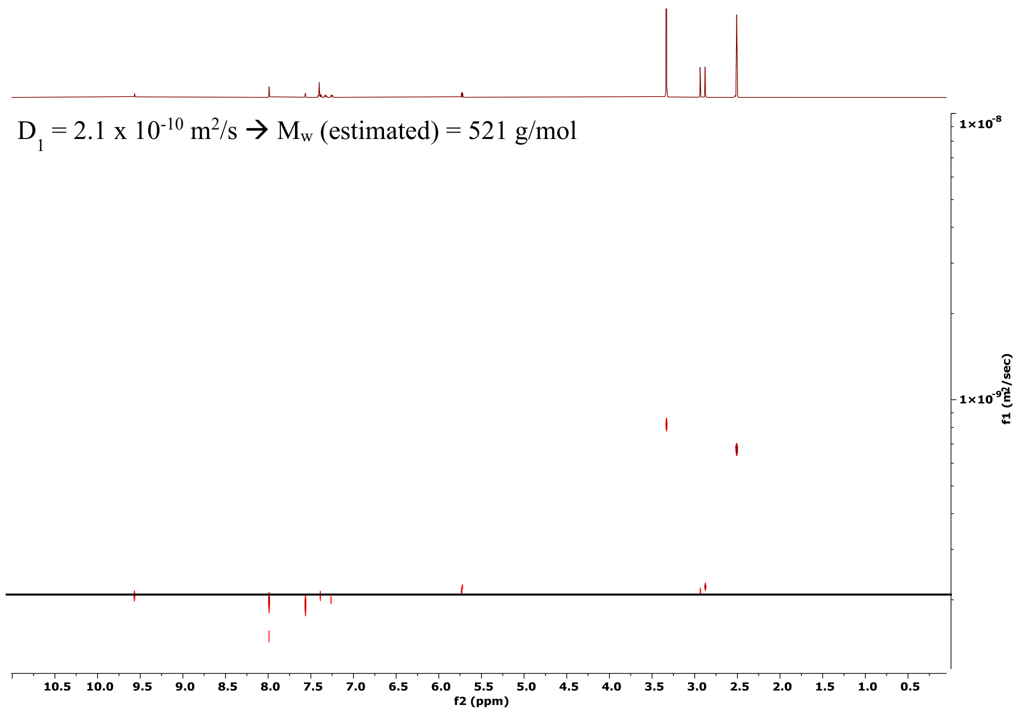

Figure S2: 2D-DOSY NMR in DMSO-d_6 of scalemic **2a**.

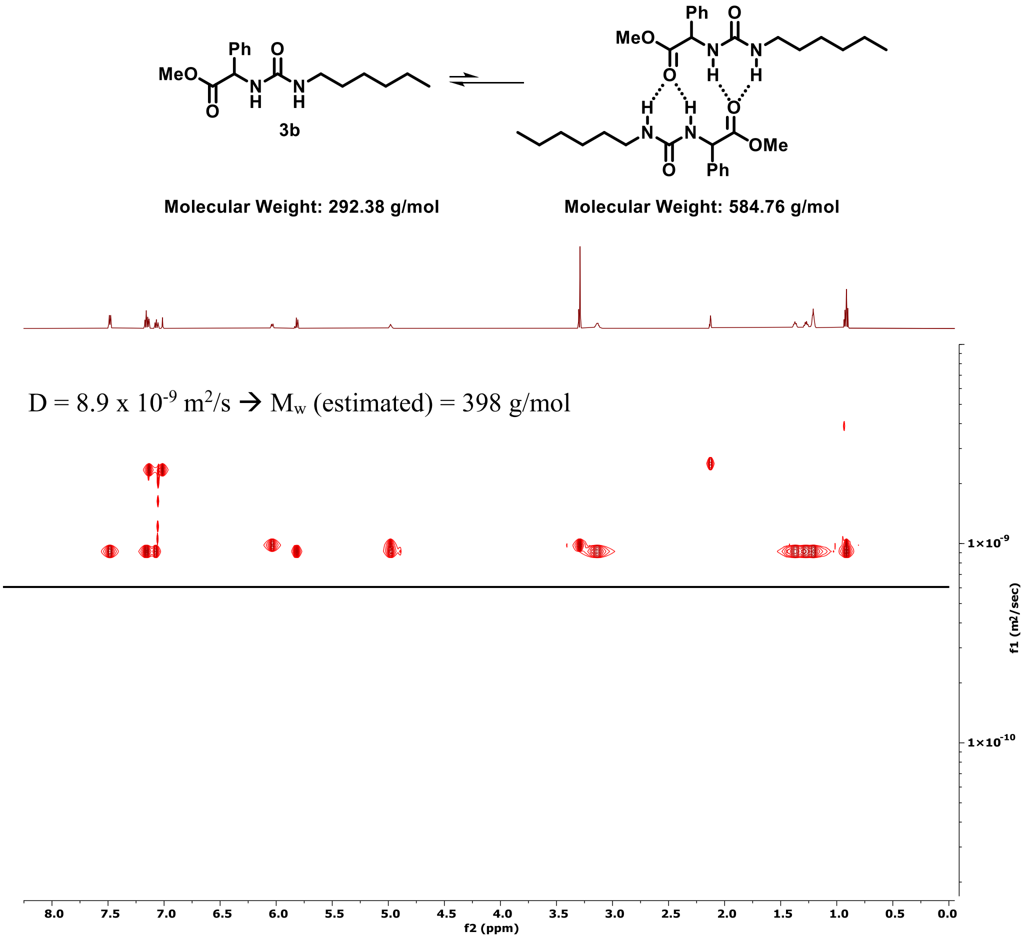

Figure S3: 2D-DOSY NMR in toluene-d_8 of scalemic **3b**.

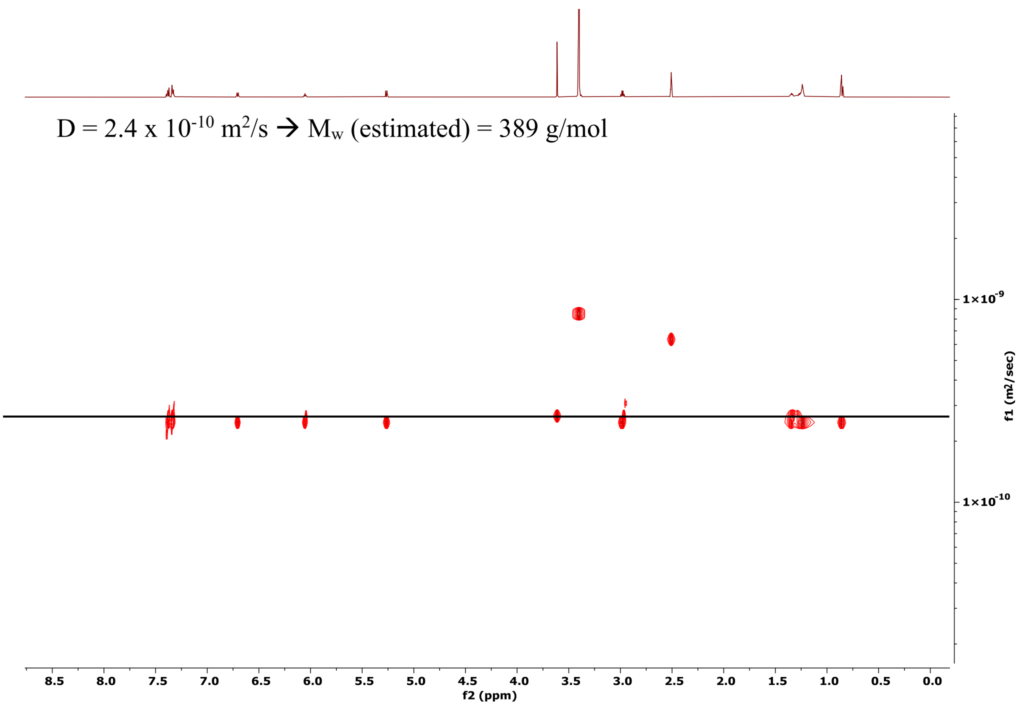

Figure S4: 2D-DOSY NMR in DMSO-d_6 of scalemic **3b**.

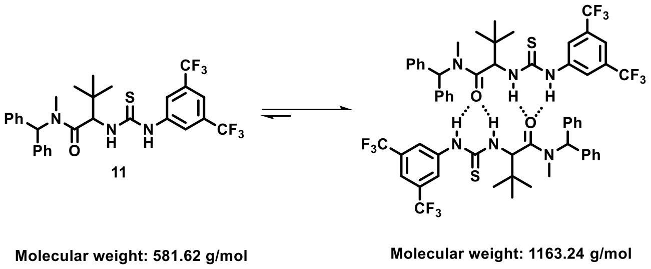

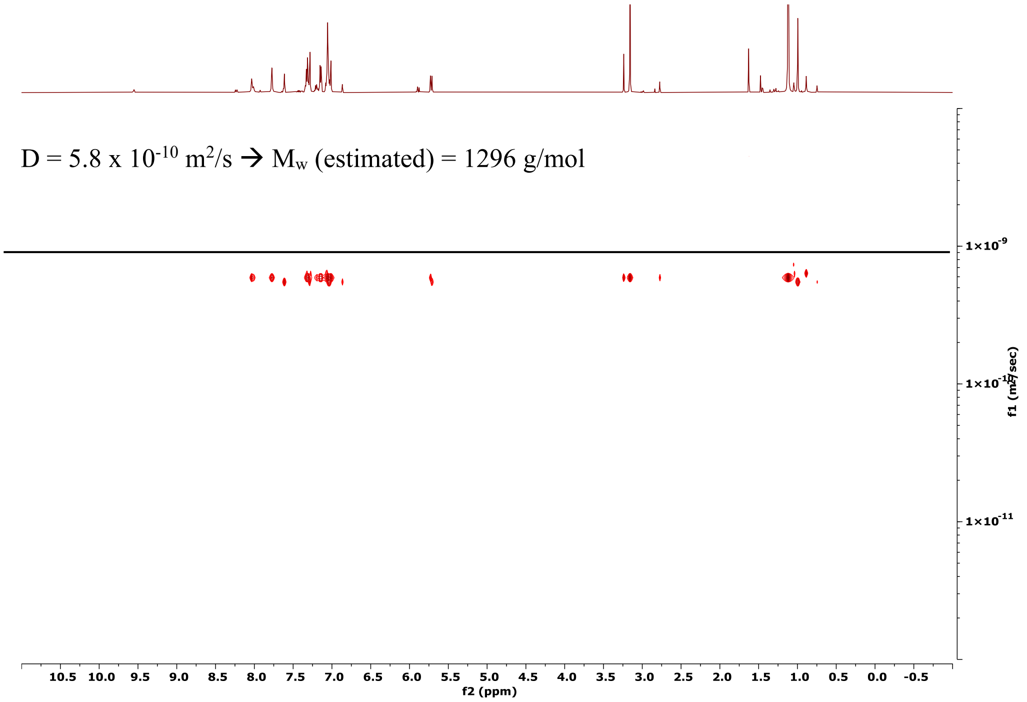

Figure S5: 2D DOSY NMR in $CDCl_3$ of scalemic **11**.

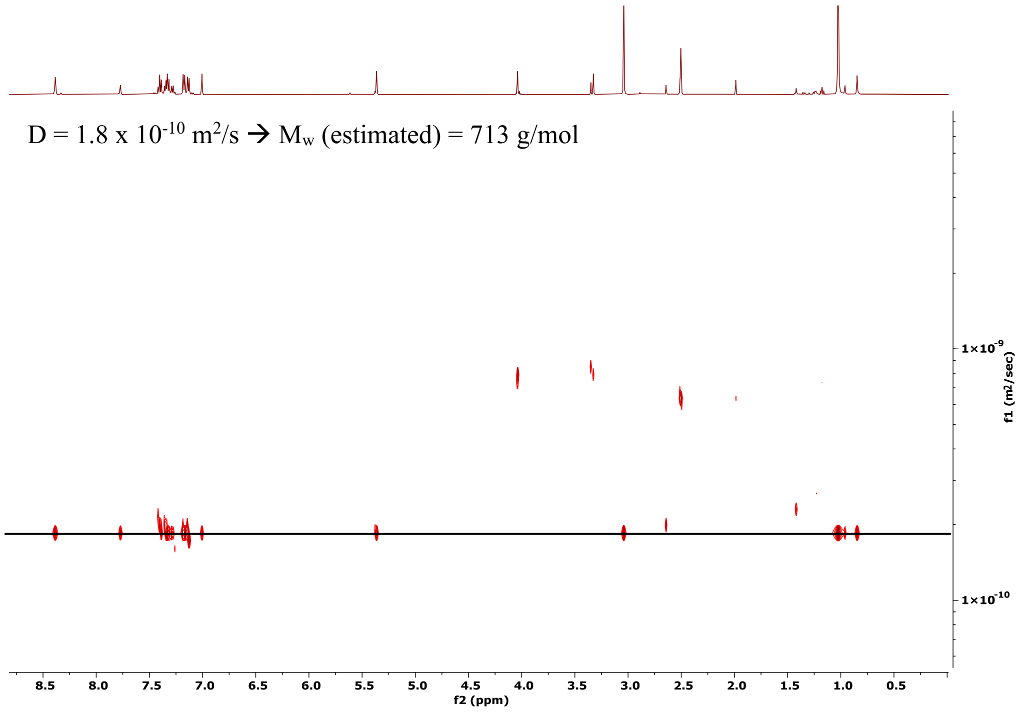

Figure S6: 2D DOSY NMR in DMSO-d$_6$ of scalemic **11**.

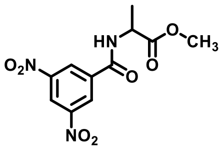

12

Molecular Weight: 297.22 g/mol

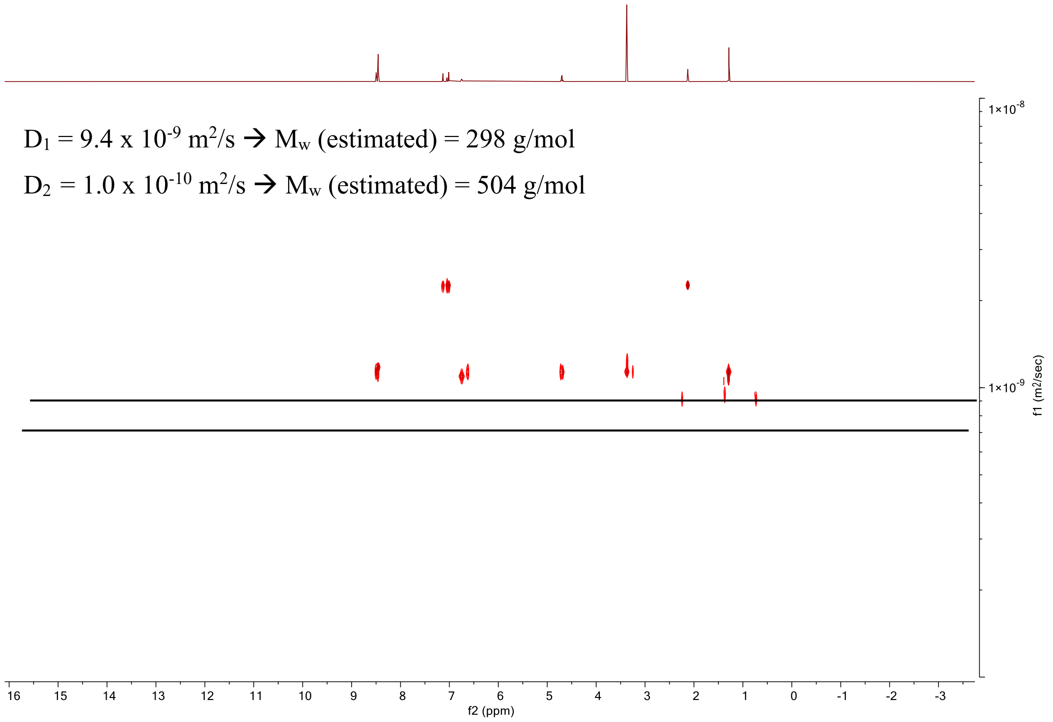

Figure S7: 2D-DOSY NMR in toluene-d$_8$ of scalemic **12** (50 mM).

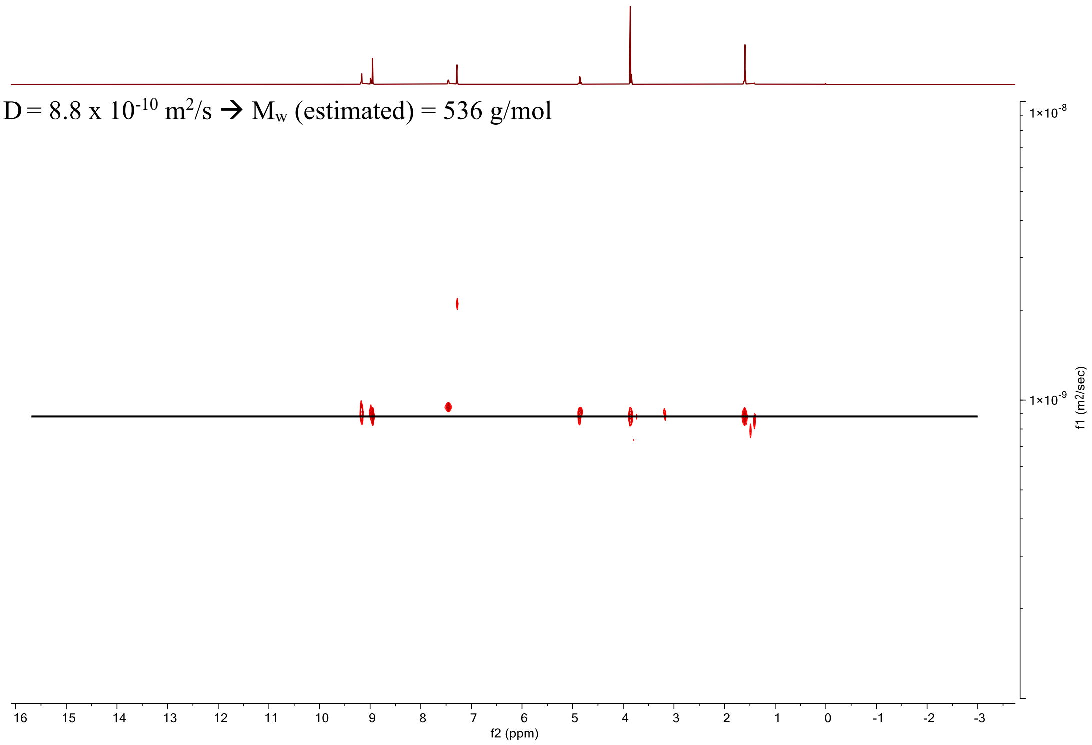

Figure S8: 2D-DOSY NMR in $CDCl_3$ of scalemic **12** (250 mM). **NOTE**: For solubility reasons, this spectrum was recorded in $CDCl_3$.

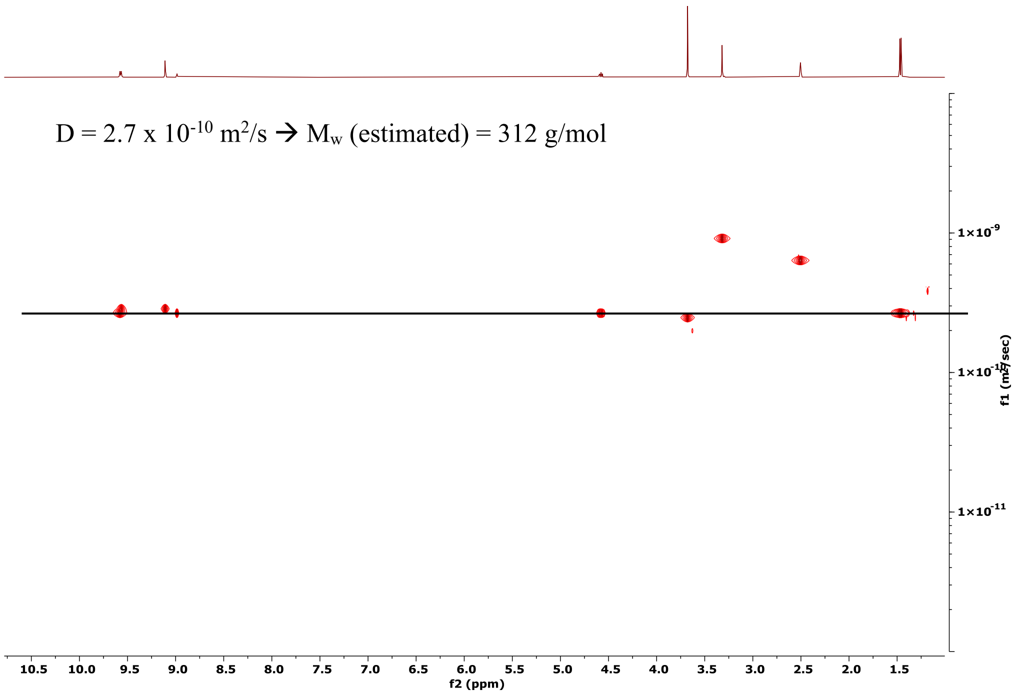

Figure S9: 2D-DOSY NMR in DMSO-d_6 of scalemic **12**.

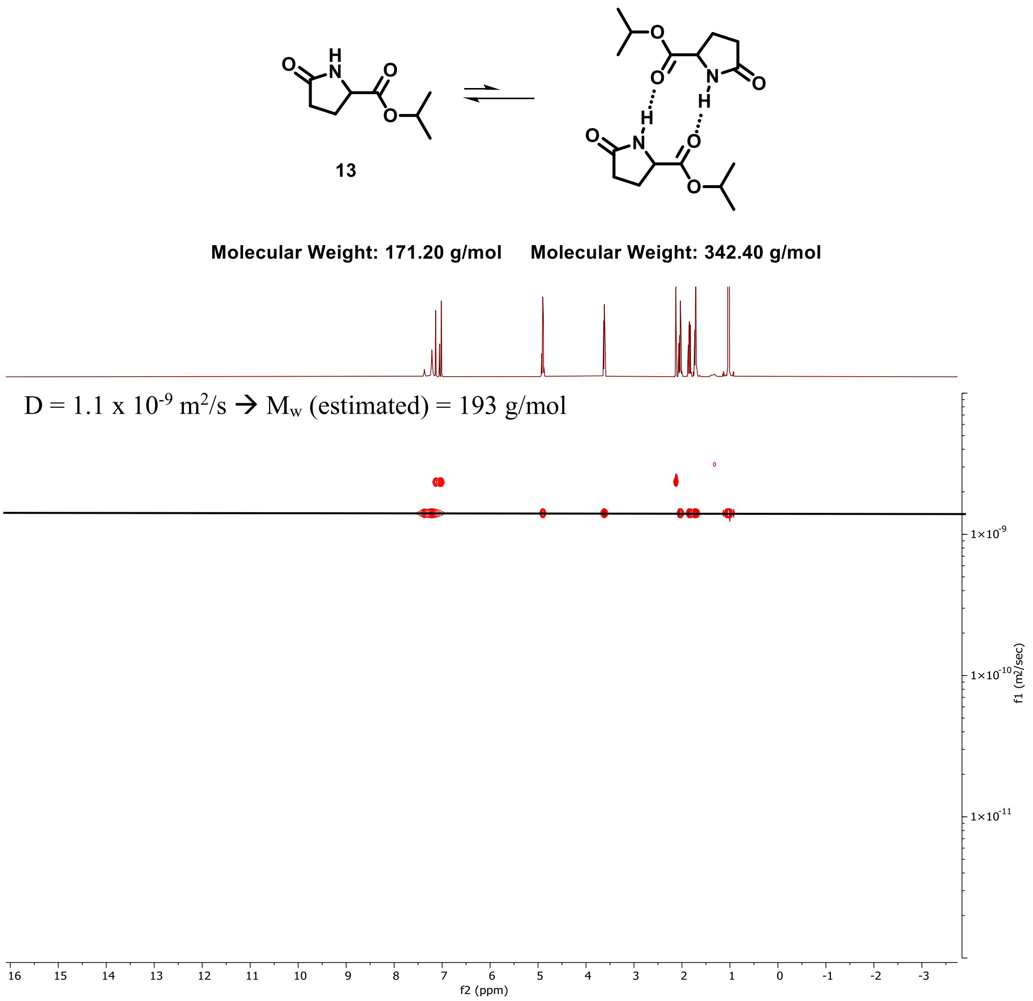

Figure S10: 2D-DOSY NMR in toluene-d_8 of scalemic **13** (100 mM).

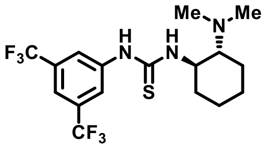

14
Molecular Weight: 413.43 g/mol

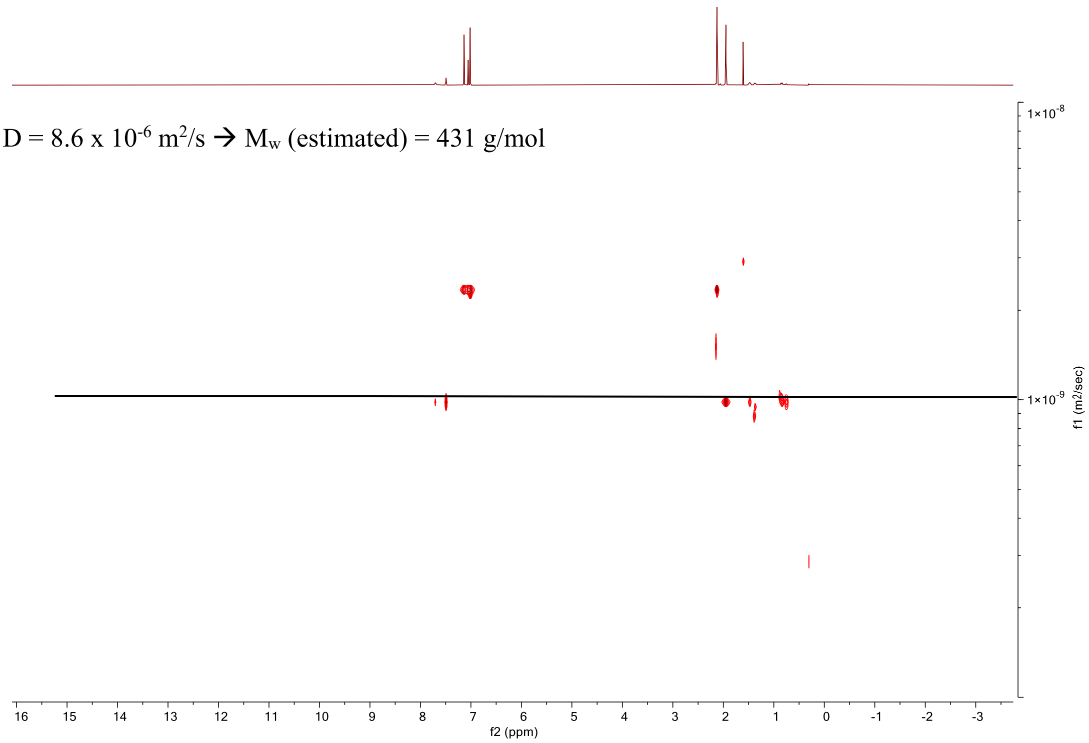

Figure S11: 2D-DOSY NMR in toluene-d_8 of scalemic **14** (50 mM).

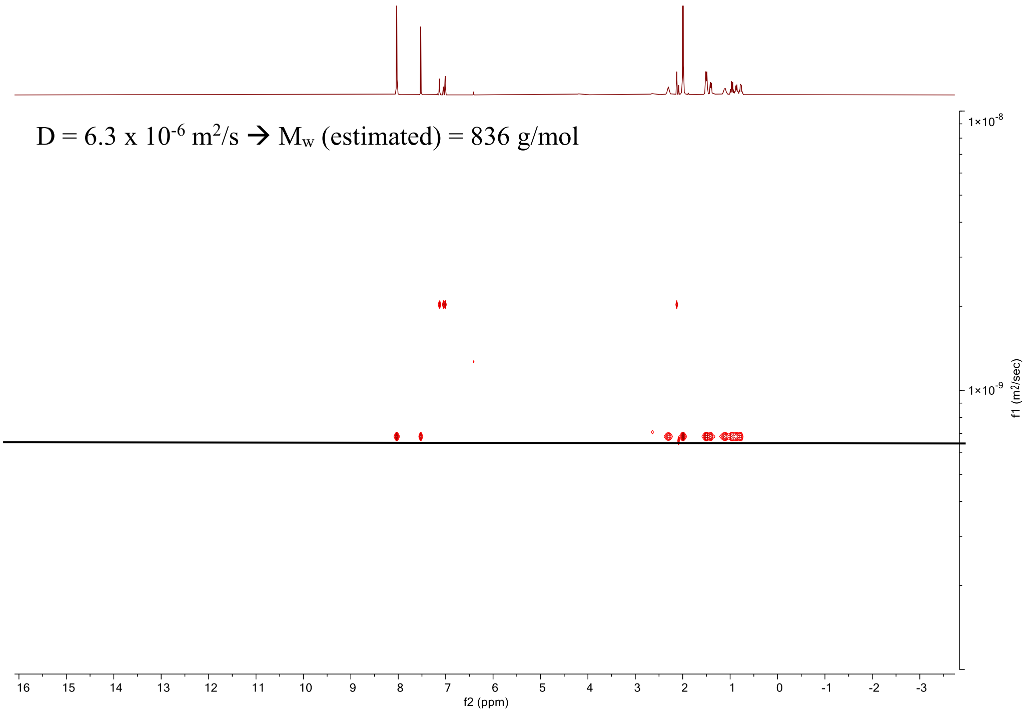

Figure S12: 2D-DOSY NMR in toluene-d_8 of scalemic **14** (200 mM).

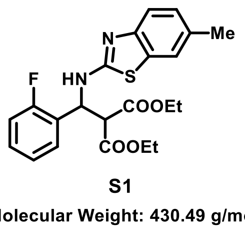

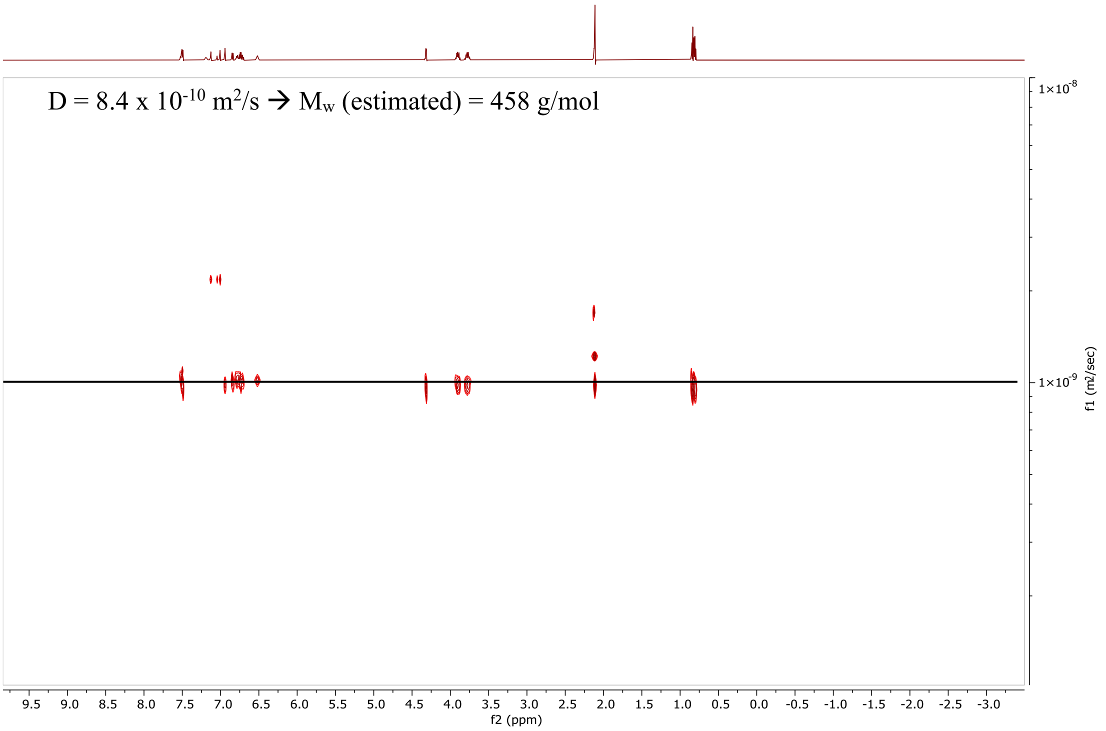

Figure S13: 2D-DOSY NMR in toluene-d_8 of scalemic **S1** (100 mM).

3.2 Solid state analysis

Crystal structure analysis was performed to study aggregation in the solid state and the nature of the intermolecular interactions. While a dimeric crystal structure might be indicative of a SIDA active compound, this might not be always the case.

The crystal structure of **4e** (CCDC: 1040052), SIDA active in toluene-d_8, was published previously by our group.[2]

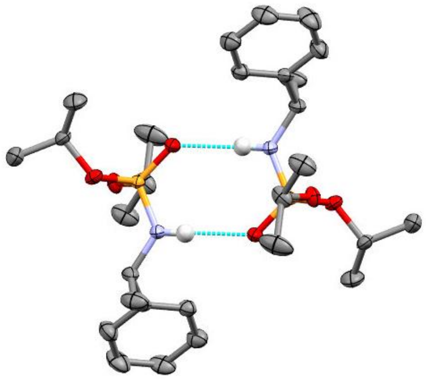

Figure S14: Structure of the H-bonded dimer of rac-**4e** d(N–H···O═P) = 2.850 Å. SIDA activity was observed.

The crystal structure of **4f** (CCDC: 265442), SIDA inactive, was published previously.[3]

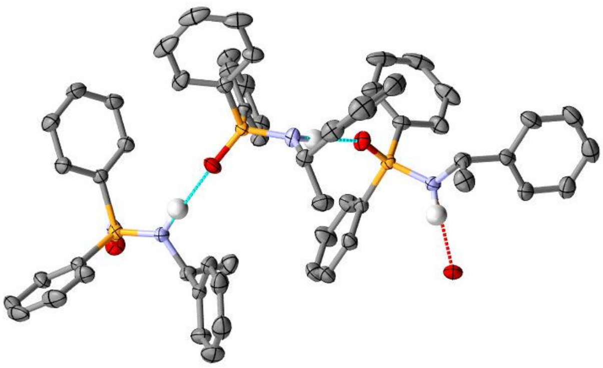

Figure S15: Structure of the H-bonded aggregate of **4f**. SIDA inactive.

The crystal structure of **8** (CCDC: 2059102), SIDA inactive, was published previously by our group.[2]

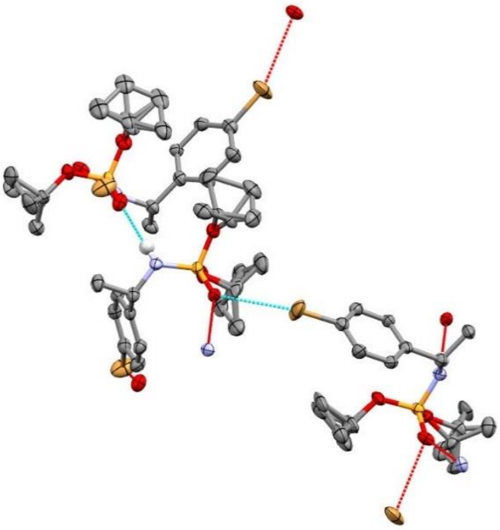

Figure S16: Crystal structure of the H-bonded aggregate of **8**. SIDA inactive.

The crystal structure of **12** (CCDC: 1529896), SIDA active in $CDCl_3$, toluene-d_8 and benzene-d_6, was published previously.[4]

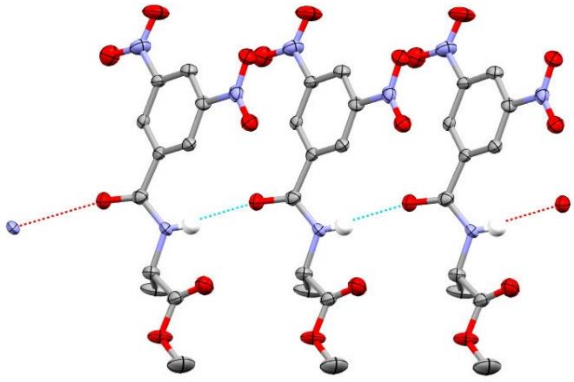

Figure S17: Crystal structure of the H-bonded polymer of **12**. SIDA active.

The crystal structures of **13** (CCDC: 1839816), SIDA active in toluene-d_8, was published previously.[5]

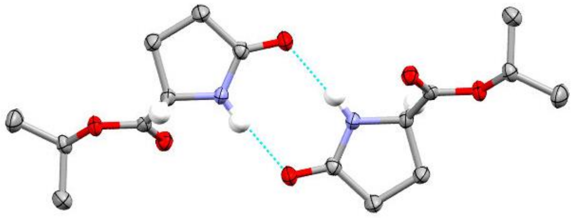

Figure S18: Crystal structure of the H-bonded dimer of **13**. d(N–H···O═C) = 2.888 Å. SIDA active.

The crystal structure of **15** (CCDC: 1524472), SIDA active in toluene-d_8, was published previously.[6]

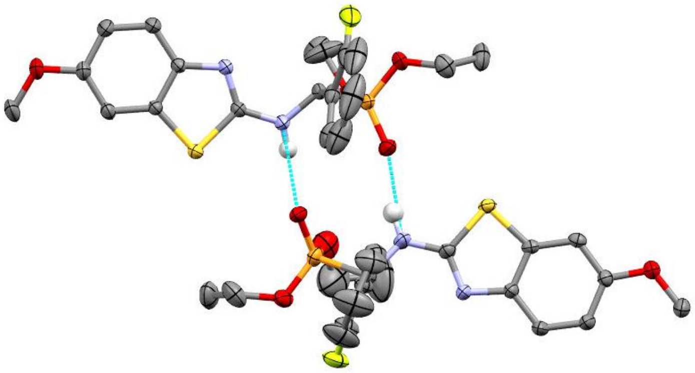

Figure S19: Structure of the H-bonded dimer of **16**. d(N–H···O═P) = 1.938 Å. SIDA active.

The crystal structure of **16,** SIDA active in toluene-d_8, was published previously.[7]

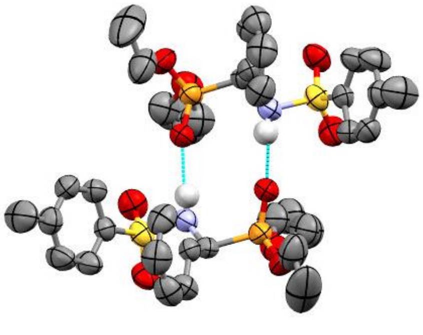

Figure S20: Structure of the H-bonded dimer of **16**. d(N–H···O═P) = 2.871 Å. SIDA active.

The crystal structure of **S3** (CCDC: 781779), SIDA inactive, was published previously.[8]

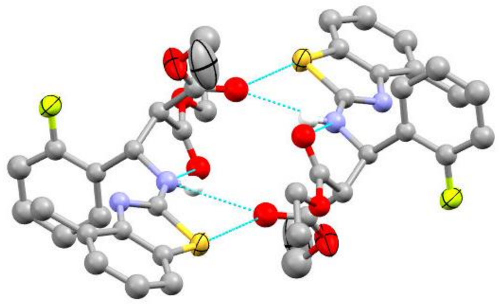

Figure S21: Crystal structure of the H-bonded aggregate of **S3**. d(N–H···O═C) 2.265 Å. SIDA inactive.

3.3 Association constants

NMR titrations in toluene-d_8 were performed to determine the association constant (K_a) of the enantiopure or racemic compounds **3b**, **5b**, and **12** by following the chemical shift of a distinct signal over different concentrations.[9] The binding isotherm was obtained through a non-linear regression analysis using platform http://app.supramolecular.org/bindfit/.[10]

According to literature, the relationship between the association constant K_a and the chemical shift of a distinct signal can be described as follows:[9] The association constant K_a for a monomer (M) – dimer (D) equilibrium is defined as:

$$2\,\mathrm{M} \rightleftarrows \mathrm{D}$$

$$\mathrm{K_a} = \frac{[\mathrm{D}]}{[\mathrm{M}]^2} \quad (1)$$

With the initial concentration $[M]_0$ being the sum of the monomer (M) and dimer (D) species.

$$[M]_0 = [M] + 2\,[D] \quad (2)$$

The observed chemical shift (δ_{obs}), a linear combination of the chemical shift of the monomeric (δ_M) and dimeric species (δ_D) which is directly proportional to the molar fraction $2[D]/[M]_0$ or $(1 - [M]/[M]_0)$ of the dimer can be described as follows:

$$\delta_{\mathrm{obs}} = \delta_{\mathrm{M}} + (\delta_{\mathrm{D}} - \delta_{\mathrm{M}})\left(\frac{2[\mathrm{D}]}{[\mathrm{M}]_0}\right) \quad (3)$$

$$\delta_{\mathrm{obs}} = \delta_{\mathrm{M}} + (\delta_{\mathrm{D}} - \delta_{\mathrm{M}})\left(1 - \frac{[\mathrm{M}]}{[\mathrm{M}]_0}\right) \quad (4)$$

Expressing equations (3 and (4 as [D] and [M] respectively and combining it with equation (1 gives allows us to relate K_a with the chemical shift.

$$\mathrm{K_a} = \frac{(\delta_{\mathrm{D}} - \delta_{\mathrm{M}}) - (\delta_{\mathrm{obs}} - \delta_{\mathrm{M}})}{2(\delta_{\mathrm{D}} - \delta_{\mathrm{obs}})^2[\mathrm{M}]_0} \quad (5)$$

Hence, the values of δ_M and δ_D must be known to compute K_a. For large values of K_a, the extrapolation to accurate values of δ_D is easier than to δ_M, as for the latter one, the NMR measurement must be performed at very high dilutions, which makes the sensitivity of the instrument a potentially limiting factor. Nowadays, association constants as high as 10^5 M^{-1} are obtained by NMR titrations.[10]

Procedure: Samples of various concentrations (1 mM – 800 mM for **3b**; 0.5 mM – 500 mM for **12**; 0.5 mM – 200 mM for **5b**) of the racemic and enantiopure compound were prepared in toluene-*d*$_8$. The N-H signal (highlighted in red) was monitored by ^{1}H-NMR (500 MHz, 25 °C) and all the measurements were independently performed twice. Data for the isotherms and fitting analysis can be obtained via the following links (open access).

Note: For compound **3b**, peak broadening was observed for concentrations < 1 mM, preventing the accurate determination of the chemical shift.

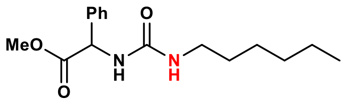

3b

<u>a) enantiopure **3b**</u>**:** K_a (enantiopure **3b**) = 8.9 ± 0.2 M^{-1}

Measurement 1: K_a^1 = 9.1 M^{-1} ± 3.67 %
http://app.supramolecular.org/bindfit/view/c1226ec0-5f7d-4250-aff5-8e72d90145fb

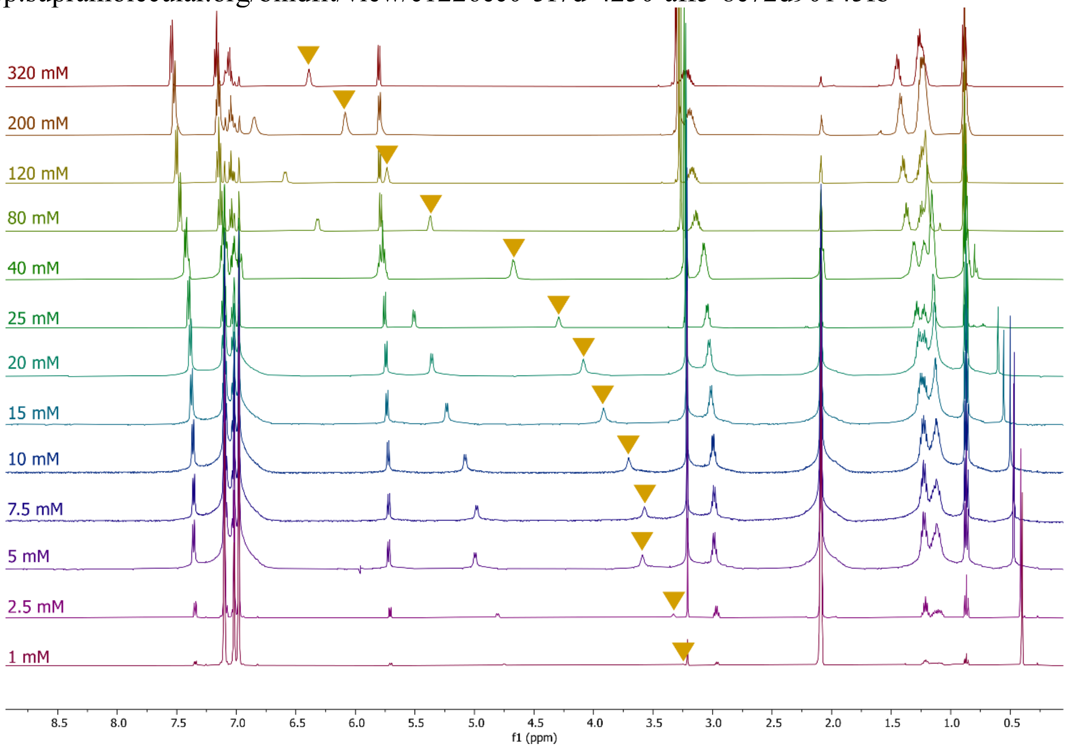

Scheme S1: NMR titration of enantiopure **3b** in toluene-*d*$_8$. Studied peak is indicated by a triangle.

Measurement 2: K_a^2 = 8.7 M^{-1} ± 4.01 %
http://app.supramolecular.org/bindfit/view/26f9eedf-b956-4951-adb1-76ae12dda733

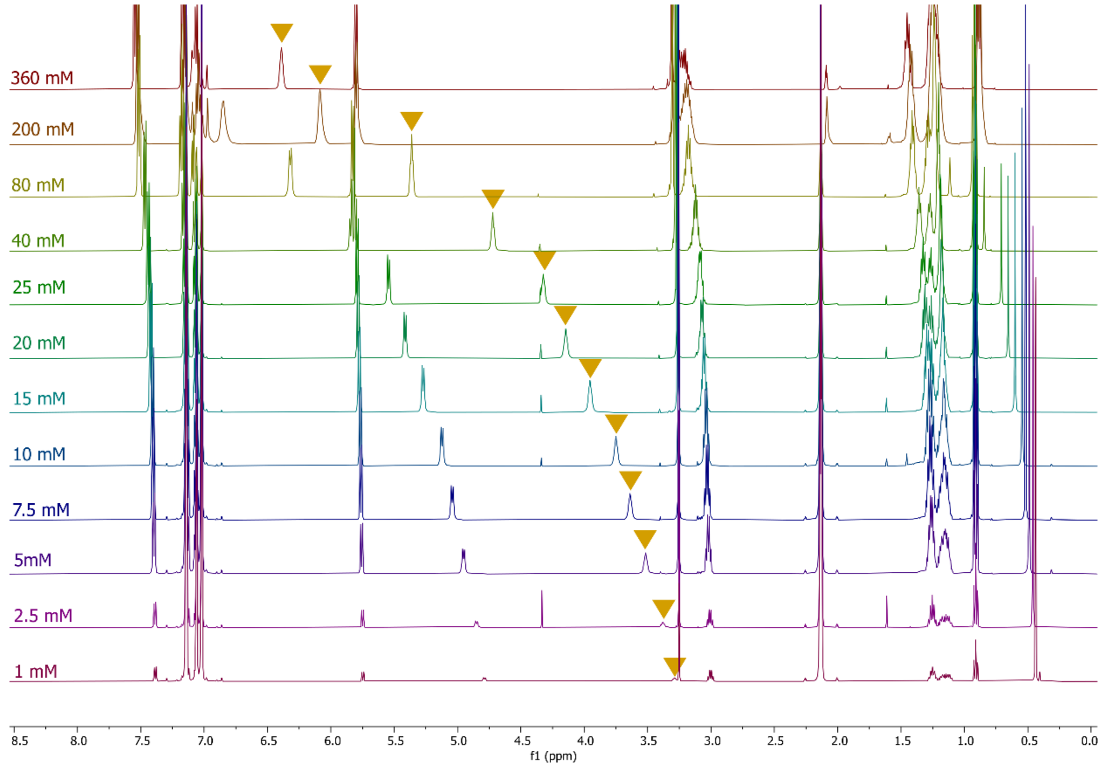

Scheme S2: NMR titration of enantiopure **3b** in toluene-d_8. Studied peak is indicated by a triangle.

<u>b) racemic **3b**:</u> K_a (racemic **3b**) = 8.8 ± 0.2 M^{-1}

Measurement 1: K_a^1 = 8.6 M^{-1} ± 4.43 %

http://app.supramolecular.org/bindfit/view/bcd264c9-07ef-4a44-a419-b89e521f9ded

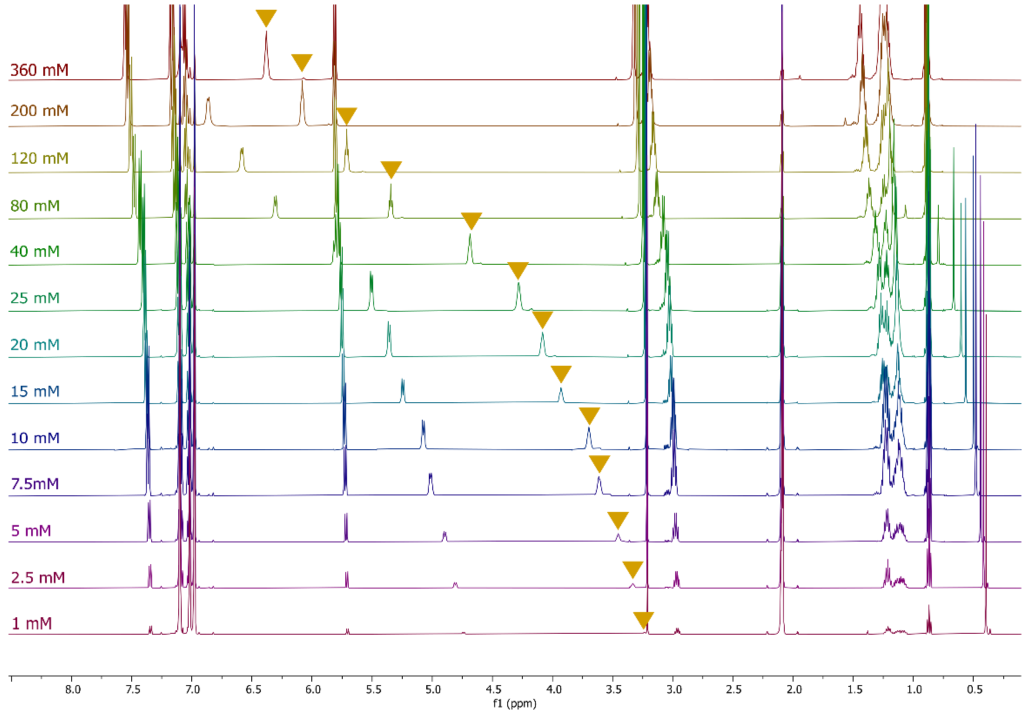

Scheme S3: NMR titration of racemic **3b** in toluene-d_8. Studied peak is indicated by a triangle.

Measurement 2: K_a^2 = 9.0 M^{-1} ± 3.66 %

http://app.supramolecular.org/bindfit/view/5731db65-f087-4cf3-8c73-a757bbdbad71

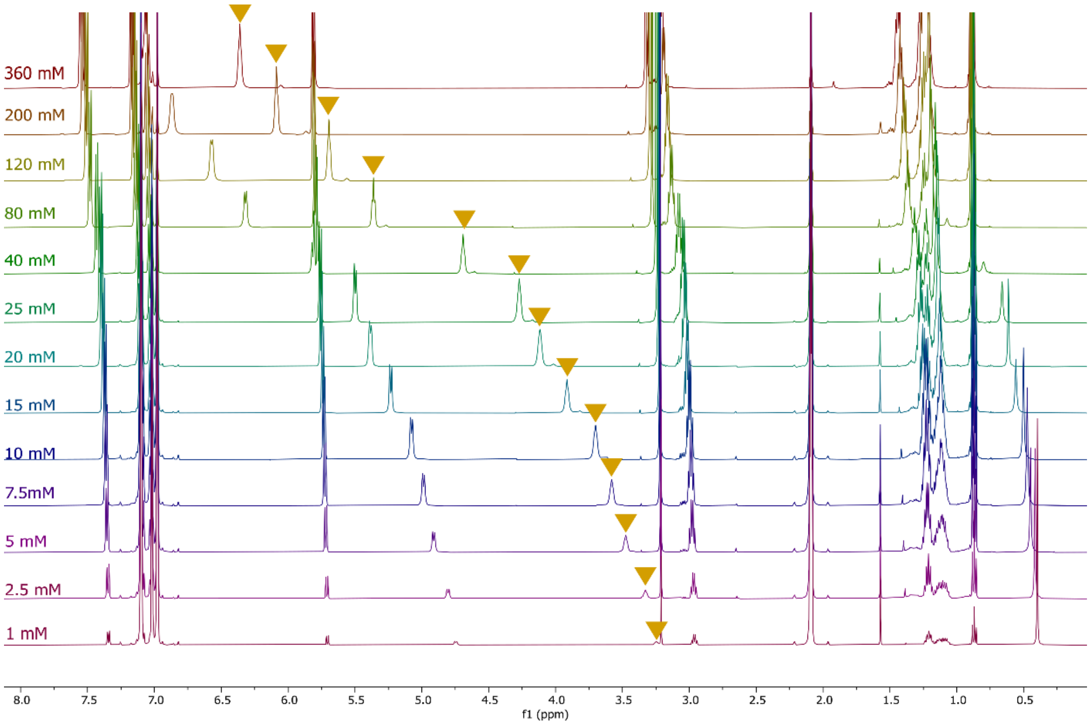

Scheme S4: NMR titration of racemic **3b** in toluene-d_8. Studied peak is indicated by a triangle.

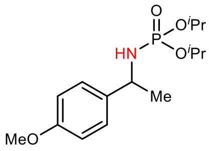

5b

<u>a) enantiopure **5b:**</u> K_a (enantiopure **5b**) = 20.4 M^{-1}

Measurement 1: $K_a^1 = 20.4\ M^{-1} \pm 0.96\ \%$
http://app.supramolecular.org/bindfit/view/d73cc8e6-0f06-4caf-9d6b-3dd1b5c5cc89

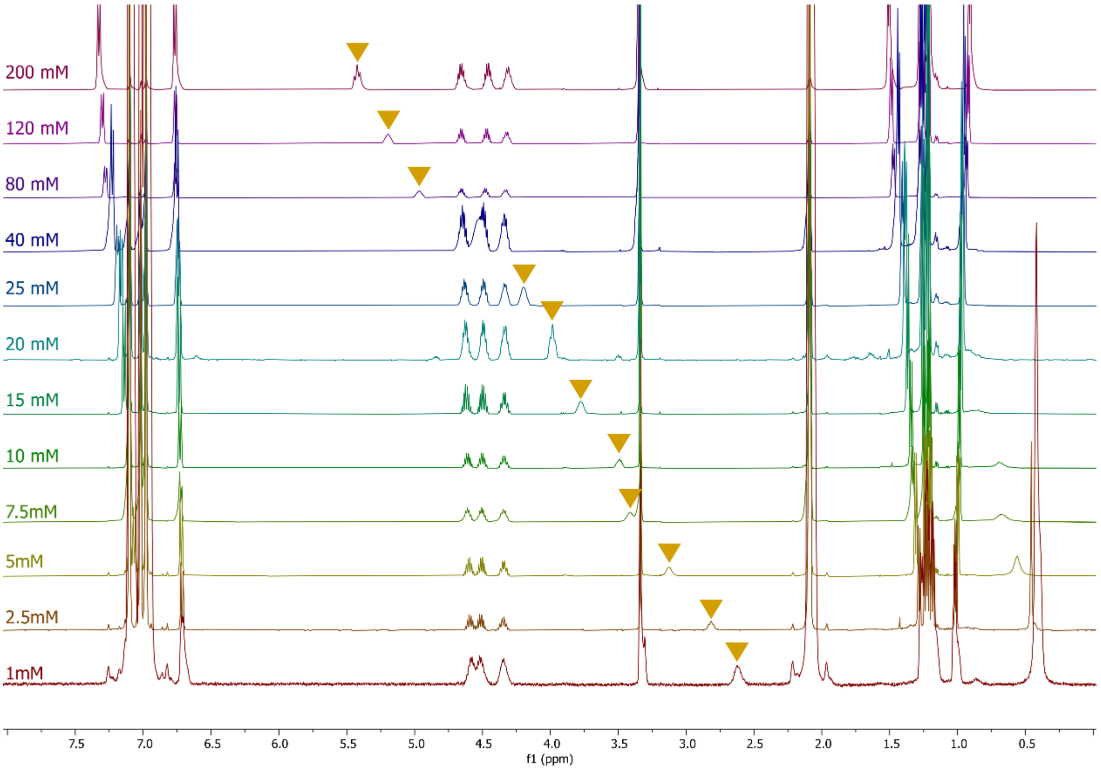

Scheme S5: NMR titration of enantiopure **5b** in toluene-d_8. Studied peak is indicated by a triangle.

Measurement 2: $K_a^2 = 20.4\ M^{-1} \pm 0.66\ \%$
http://app.supramolecular.org/bindfit/view/47c73dc8-01a7-4a0b-be1b-4f78b1277aa7

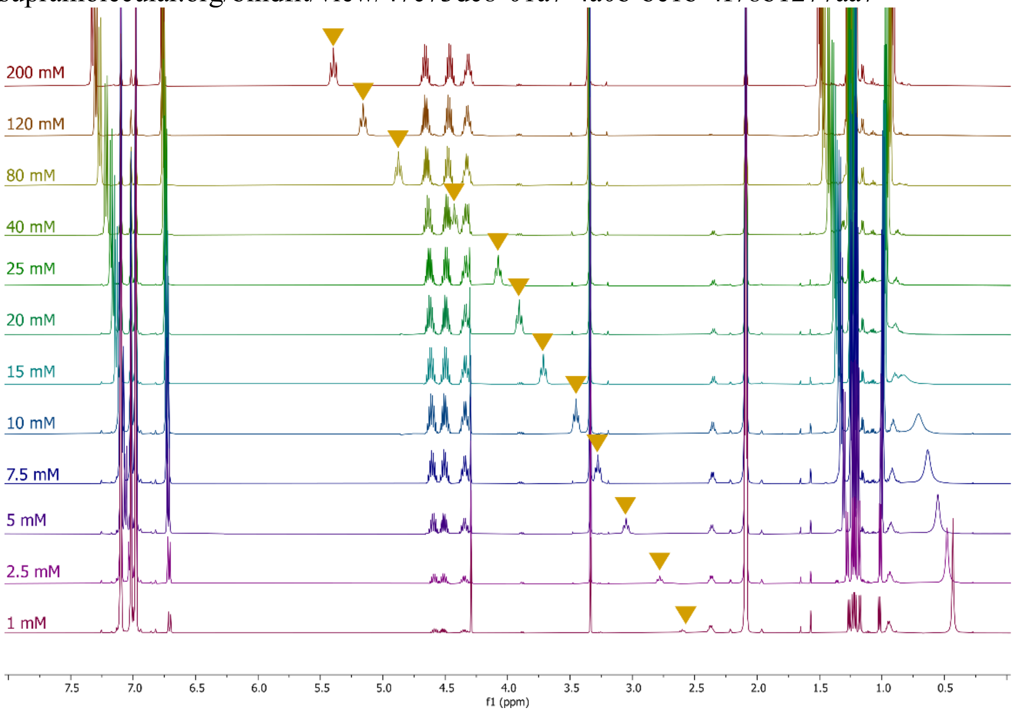

Scheme S6: NMR titration of enantiopure **5b** in toluene-d_8. Studied peak is indicated by a triangle.

<u>b) racemic **5b:**</u> K_a (racemic **5b**) = $20.4 \pm 0.4\ M^{-1}$

Measurement 1: $K_a^1 = 20.8\ M^{-1} \pm 0.86\ \%$
http://app.supramolecular.org/bindfit/view/d73cc8e6-0f06-4caf-9d6b-3dd1b5c5cc89

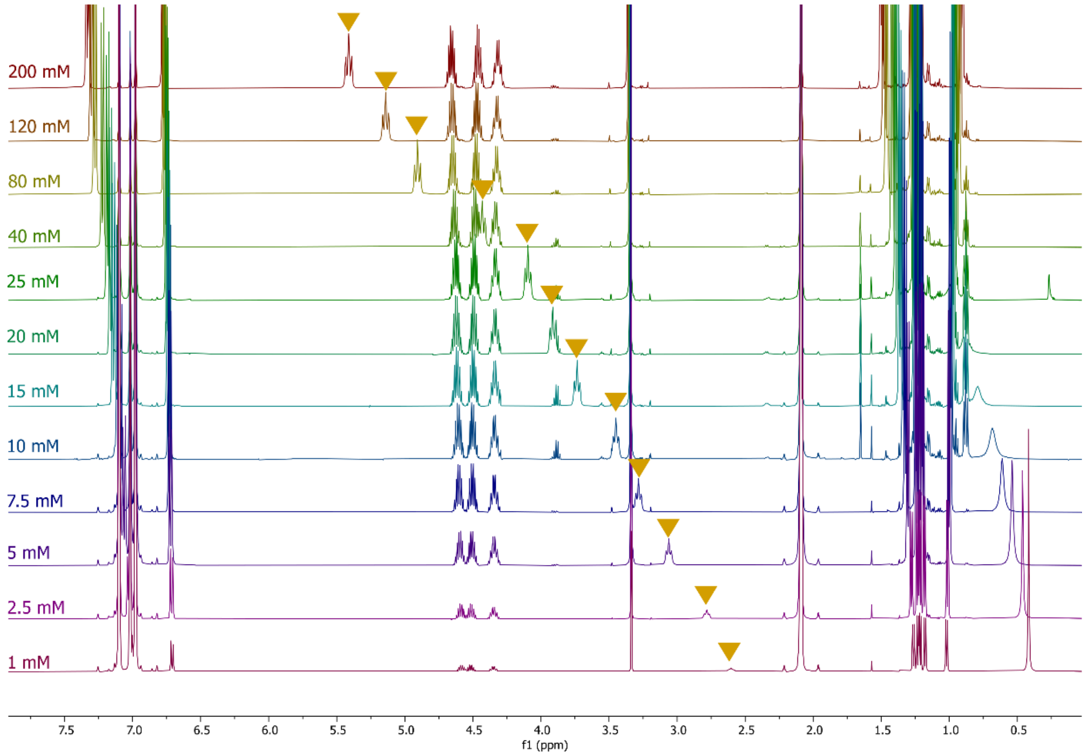

Scheme S7: NMR titration of racemic **5b** in toluene-d_8. Studied peak is indicated by a triangle.

Measurement 2: $K_a^2 = 20.1\ M^{-1} \pm 0.74\ \%$
http://app.supramolecular.org/bindfit/view/34a97745-2b47-4f43-8892-13547d6f994a

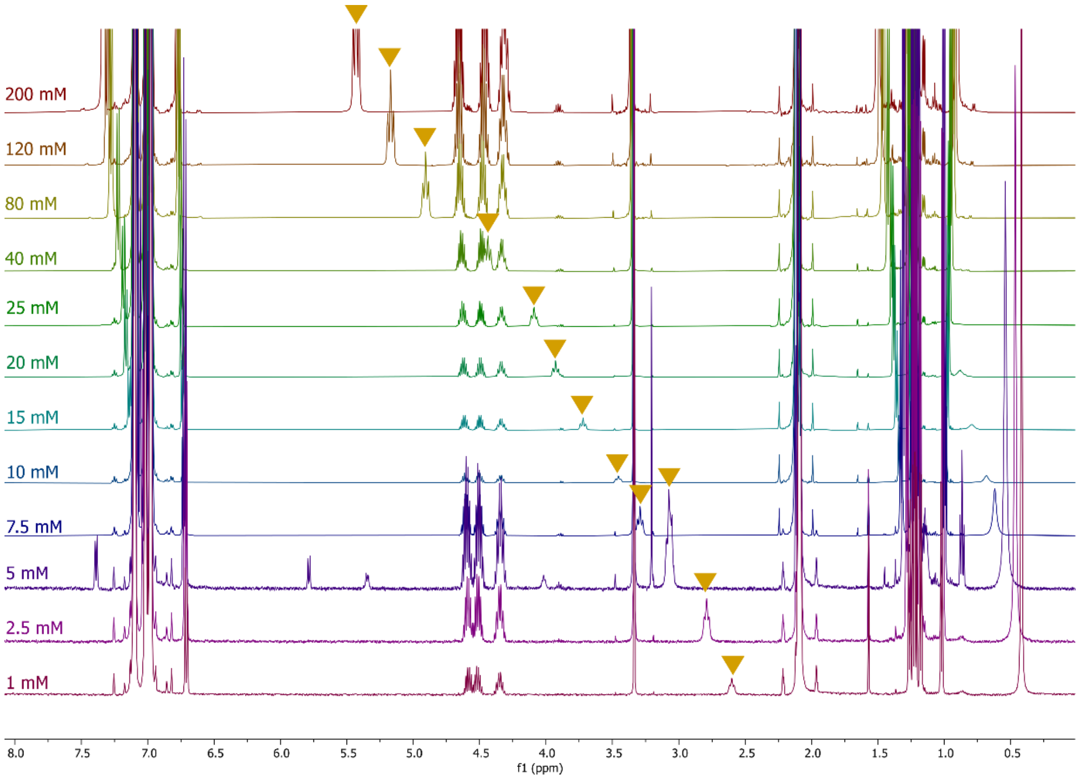

Scheme S8: NMR titration of racemic **5b** in toluene-d_8. Studied peak is indicated by a triangle.

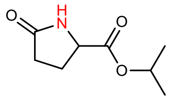

13

<u>a) enantiopure **13:**</u> K_a (enantiopure **13a**) = 14.9 ± 0.3 M^{-1}

Measurement 1: K_a^1 = 15.2 M^{-1} ± 2.0 %
http://app.supramolecular.org/bindfit/view/600bd0a6-987c-45ff-80e2-351a75bce06b

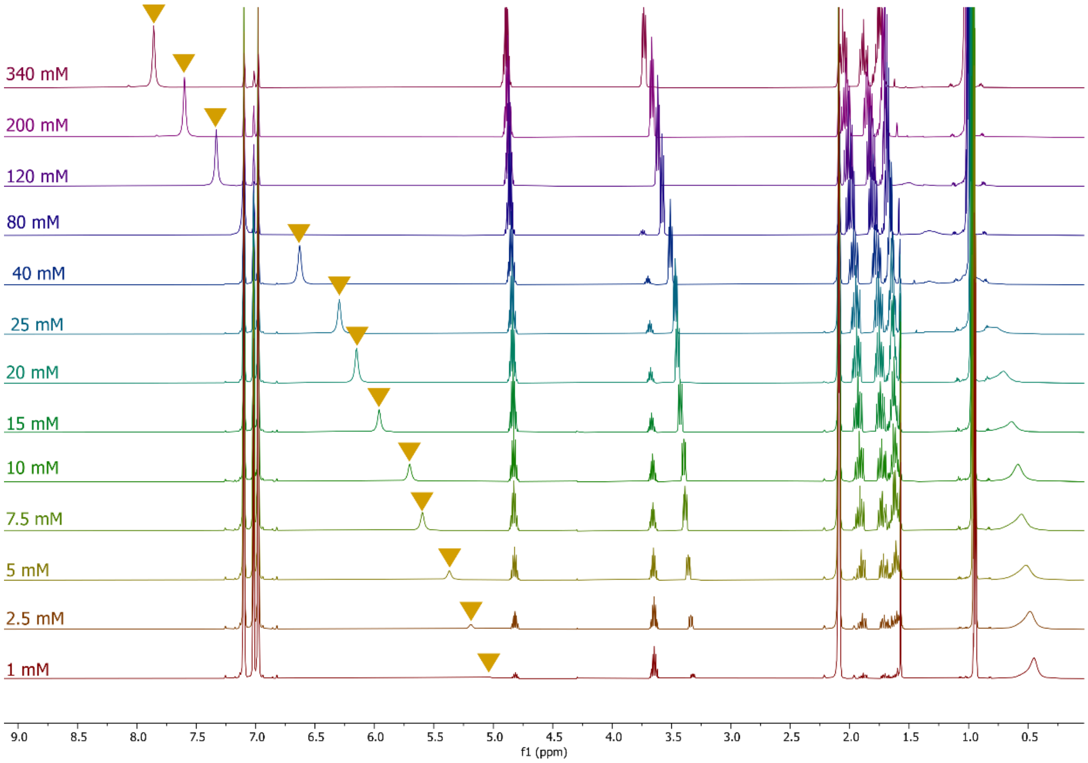

Scheme S9: NMR titration of enantiopure **13** in toluene-d_8. Studied peak is indicated by a triangle.

Measurement 2: K_a^2 = 14.6 M^{-1} ± 2.2 %
http://app.supramolecular.org/bindfit/view/73a88f55-0729-4523-9c8b-a2e80fc6f49e

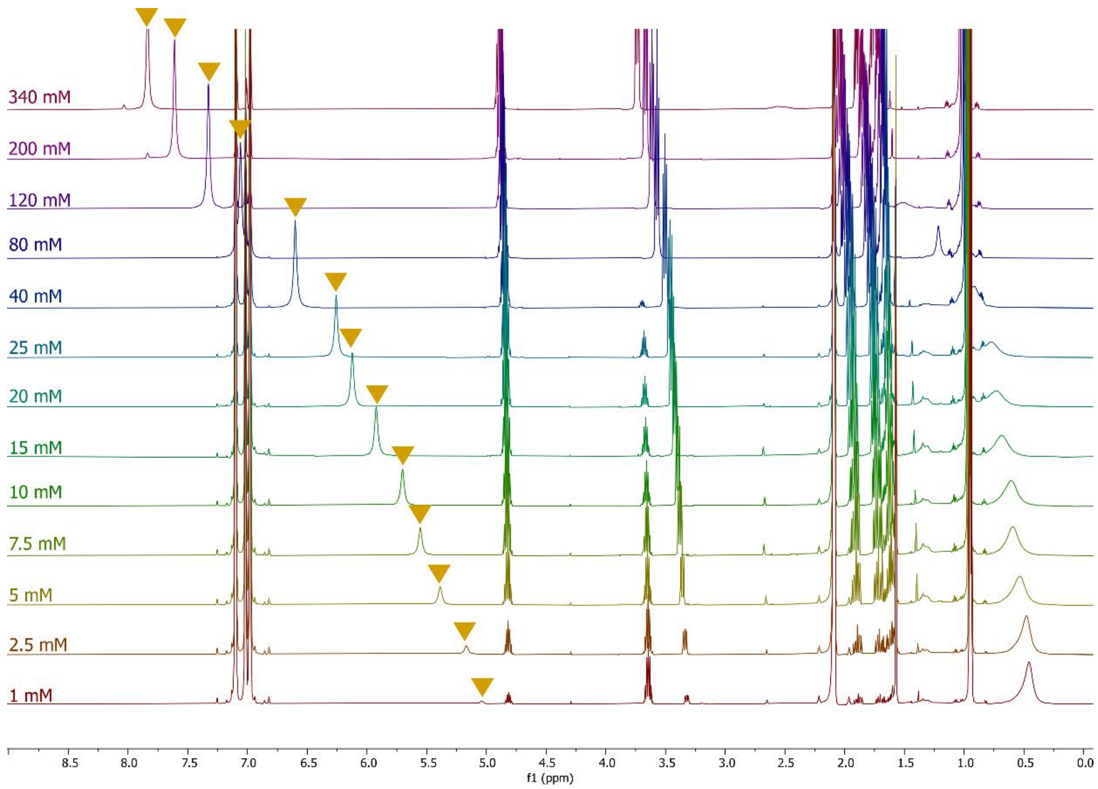

Scheme S10: NMR titration of enantiopure **13** in toluene-d_8. Studied peak is indicated by a triangle.

<u>b) racemic **13**:</u> K_a (racemic **13**) = 18.5 ± 0.6 M^{-1}

Measurement 1: K_a^1 = 17.9 M^{-1} ± 4.1 %

http://app.supramolecular.org/bindfit/view/1828eafa-bb44-4b0d-bfe3-6a502616d9bf

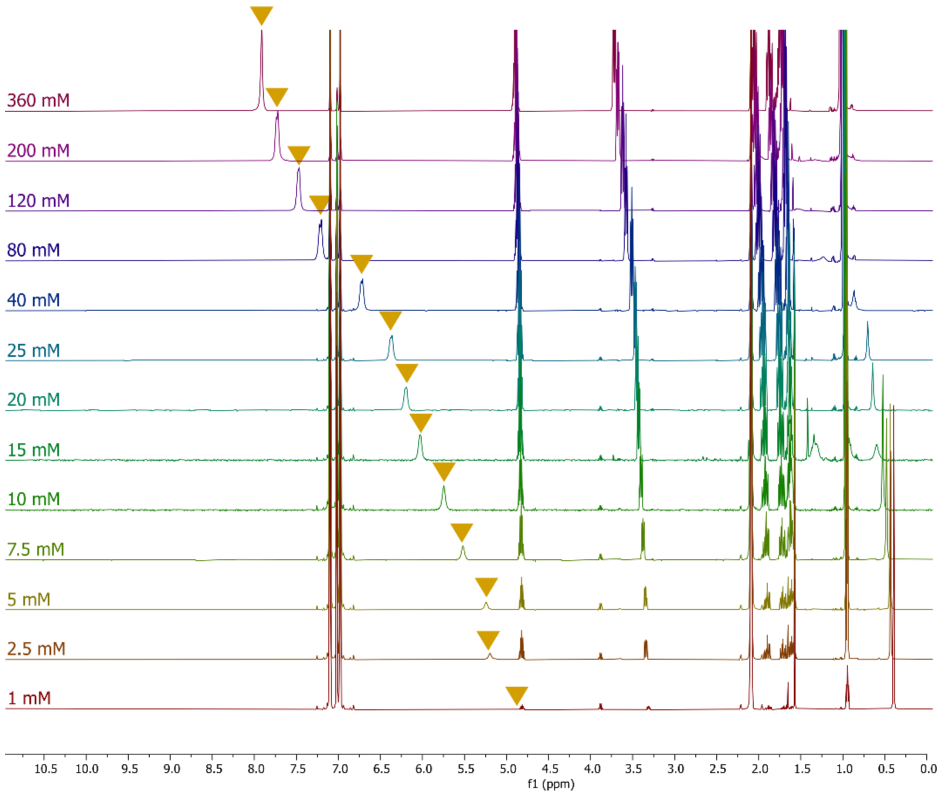

Scheme S11: NMR titration of racemic **13** in toluene-d_8. Studied peak is indicated by a triangle.

Measurement 2: K_a^2 = 19.1 M^{-1} ± 1.7 %
http://app.supramolecular.org/bindfit/view/559d788f-a874-42c3-ae31-ab67892e66e1

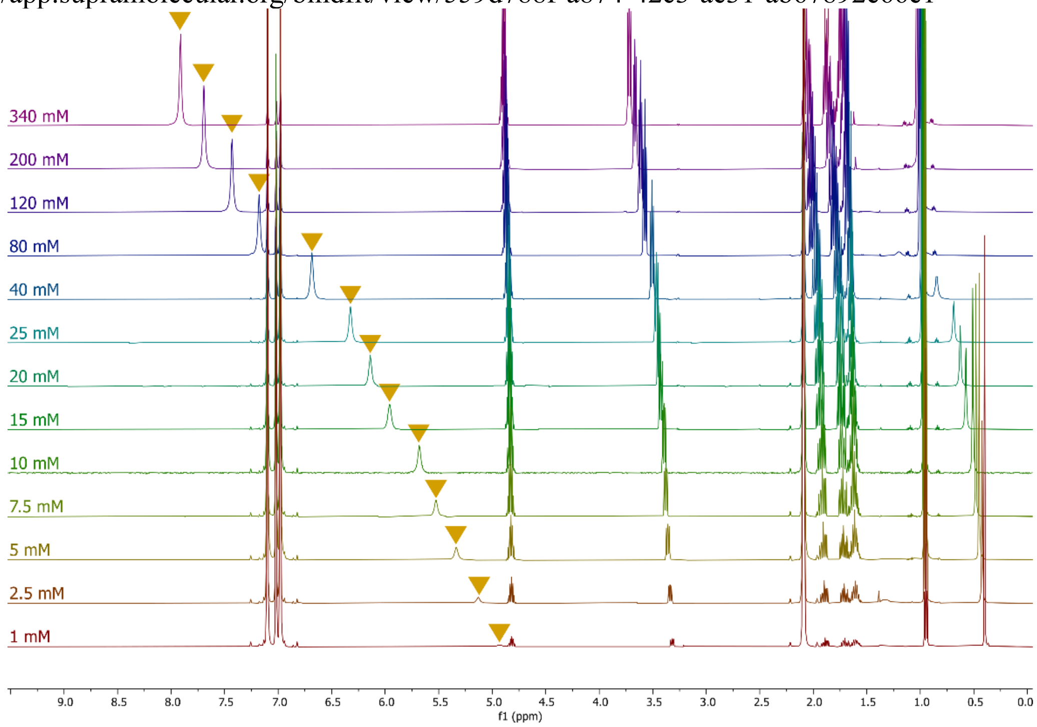

Scheme S12: NMR titration of racemic **13** in toluene-*d*$_8$. Studied peak is indicated by a triangle.

The association constant K_a allows the comparison of the degree of dimerization in racemic and enantiopure solutions.[11] According to equation (1), the concentration of a dimer in an enantiopure solution (e.g. the *R*-enantiomer) is given by

$$[\text{dimer}] = [R_2] = K_{RR}[R]^2 = K_{RR}[\text{monomer}]^2. \tag{6}$$

The concentration C' of a racemic solution (*R,S*) is defined as

$$C' = [S] + [\mathrm{R}] + 2[S_2] + 2[RS] = 2[R] + 4[S_2] + 2[RS] \tag{7}$$

with a concentration of the dimer in racemic solution expressed as

$$\begin{aligned}[\text{dimer}] &= [S_2] + [R_2] + [RS] \\ &= K_{\mathrm{RR}}([S]^2 + [R]^2) + K_{RS}[R][S] \\ &= \frac{1}{4}(2K_{RR} + K_{RS})([S] + [R])^2 + \frac{1}{4}(2K_{RR} - K_{RS})([S] - [R])^2 \\ &= \frac{1}{4}(2K_{RR} + K_{RS})[\text{monomer}]^2.\end{aligned} \tag{8}$$

Comparing equations (6) and (8), and provided that $K_{RS} = 2K_{RR}$ holds, the degree of dimerization in enantiopure solution can be regarded the same as the one in racemic solution, hence, no stereospecificity is observed.

4. Studies on the accuracy of the SIDA effect compared to chiral HPLC

4.1 Comparison NMR vs HPLC

The enantiomeric purity of the compound determines the magnitude of the splitting of the signals in SIDA active compounds, as reported previously.[12] Hence, we tested a range of enantiomeric ratios of **2a** and **5** by ^{1}H-NMR(Scheme S13 for **2a** and Scheme S15 for **5**), ^{19}F-NMR (only for **2a,** Scheme S14) or ^{31}P-NMR (only for **5,** Scheme S16) and chiral HPLC (Table S1 for **2a** and Table S2 for **5**). The difference in the chemical shift of racemic **2a** and scalemic **2a**, as well as the appearance of two sets of signals for scalemic 2a whose relative ratio matched the enantiomeric composition, clearly indicated a marked SIDA effect. The same applies for **5**. The measurement of the enantiomeric ratio of **2a** by NMR correlated well with the enantiomeric ratio obtained by chiral HPLC analysis (Table S1) (Column conditions: Chiralcel OX-H Lux Cellulose-4, *n*-heptane/^{i}PrOH 85:15, 40 °C, 1 mL/min, λ_{abs} = 248 nm)

For **5,** poor peak splitting of the signals was observed. For enantiomeric purities ≤60:40, peak splitting was negligible, especially in ^{1}H-NMR, and only one set of peaks was observed. The enantiomeric ratios of **5** were determined by peak deconvolution, as described in section 4.2 Peak deconvolution. Still, the enantiomeric ratio obtained by NMR correlated well with the enantiomeric ratio obtained by chiral HPLC (Table S2).

The enantiomeric ratios were obtained by mixing the required amounts of the (*S*)- and (*R*)-enantiomers. In total, 10 mg of the corresponding compound were dissolved in 0.6 mL of $CDCl_3$ (**2a**) or toluene-d_8 (**5b**).

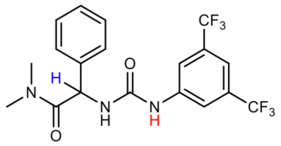

2a

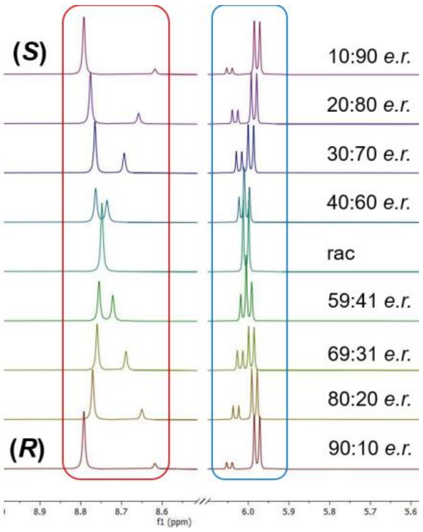

Scheme S13: Tracing the N-H signal (red) and C-H signal (blue) of **2a** by ^{1}H-NMR (400 MHz, $CDCl_3$, 25°C) with different enantiomeric ratios.

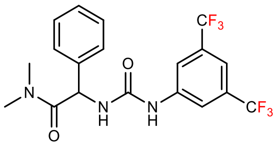

2a

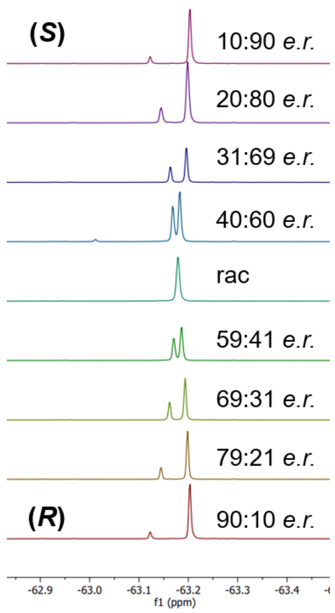

Scheme S14: Tracing the F-signal (red) of **2a** by ^{19}F-NMR (376 MHz, CDCl3, 25 °C) with different enantiomeric ratios.

entry	*e.r.* by ^{1}H-NMR	*e.r.* by ^{19}F-NMR	*e.r.* by chiral HPLC
**1**	10:90	10:90	10:90
**2**	20:80	20:80	20:80
**3**	30:70	30:70	30:70
**4**	40:60	40:60	40:60
**5**	rac	rac	rac
**6**	59:41	59:41	59:41
**7**	69:31	69:31	69:31
**8**	80:20	79:21	80:20
**9**	90:10	90:10	90:10

Table S1: Comparison of the enantiomeric ratios obtained by ^{1}H-NMR and ^{19}F-NMR in $CDCl_3$ and the enantiomeric ratios obtained by chiral HPLC of **2a**.

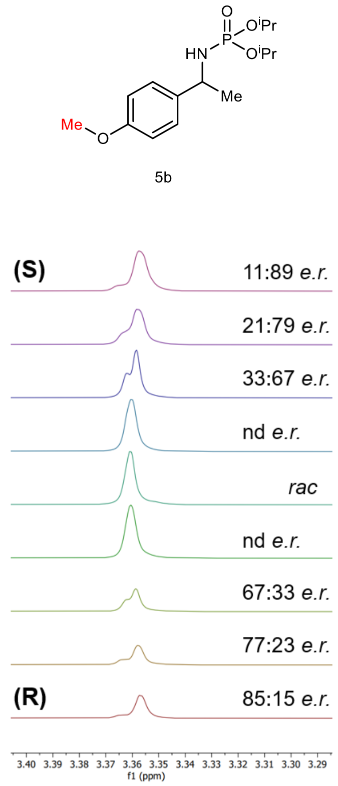

Scheme S15: Tracing the CH_3 signal (red) of **5** by ^{1}H-NMR (400 MHz, 25°C, toluene-d_8) with different enantiomeric ratios.

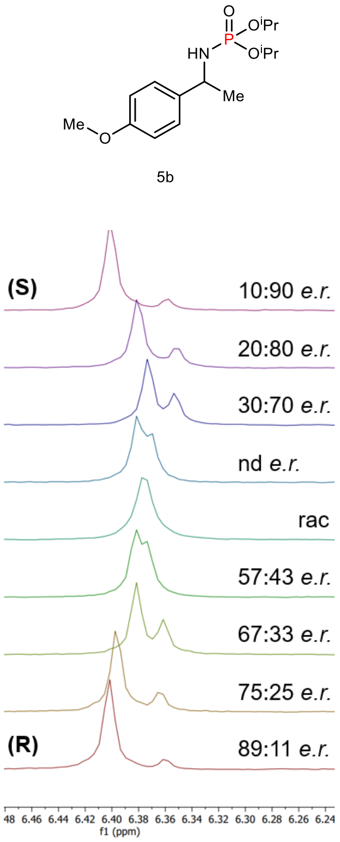

Scheme S16: Tracing the ^{31}P- signal (162 MHz, 25°C, toluene-d_8, red) of **5** with different enantiomeric ratios.

entry	*e.r.* by ^{1}H-NMR	*e.r.* by ^{31}P-NMR	*e.r.* by chiral HPLC
**1**	11:89	10:90	10:90
**2**	21:79	20:80	20:80
**3**	33:67	30:70	30:70
**4**	-	-	41:59
**5**	rac	rac	rac
**6**	-	57:43	60:40
**7**	67:33	67:33	70:30
**8**	77:23	75:25	79:21
**9**	85:15	89:11	89:11

Table S2: Comparison of the enantiomeric ratios obtained by ^{1}H-NMR and ^{31}P-NMR in toluene-d_8 and the enantiomeric ratios obtained by chiral HPLC of **5**.

4.2 Peak deconvolution

For compounds with poor peak resolution, such as **5**, peak deconvolution was performed (Scheme S17). It allows the resolution of overlapping peaks and the determination of the underlying peak area, which can be used to calculate the enantiomeric ratio. MestReNova v15.0.0. software offers a tool for Global Spectral Deconvolution (GSD - Global Spectral Deconvolution (GSD) - Mestrelab Resources), which will automatically deconvolute the selected peaks. The refinement level was set to Ref. 2 (5 fitting cycles).

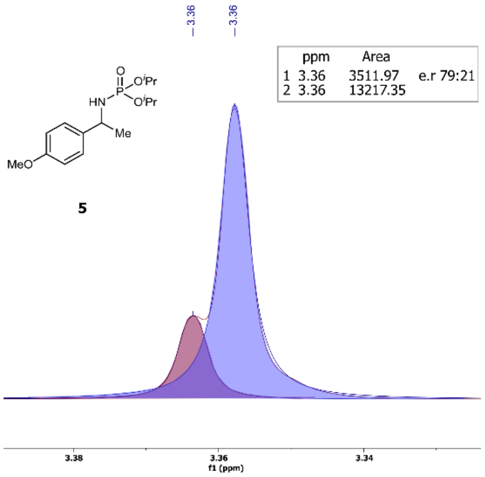

Scheme S17: a) ^{1}H-NMR spectrum of **5**. Peak deconvolution was performed, the area for the (*S*)-enantiomer is labelled in purple and the one for the (*R*)-enantiomer is labelled in blue. b) peak area obtained by peak deconvolution.

The relative area (e.g. for the *S*-enantiomer) was calculated as follows:

$$(S) = \left(\frac{\text{area }(S)}{\text{area }(S) + \text{area }(R)}\right) * 100 \tag{9}$$

5. Solvent study

5.1 NMR spectra using solvent suppression

A systematic solvent study was performed using ~10 mg of **12** or **1h** (*e.r.* 90:10) in 0.6 mL of the indicated solvent. The spectra were recorded using a 400 MHz NMR instrument with a solvent suppression routine: Varian NMR WET1D (water suppression enhanced through T_1 effects) solvent suppression sequences were used to suppress the solvent peaks. More information on the WET1D solvent suppression sequence can be found under reference[13] By using a capillary filled with a deuterated solvent, the spectrum can be locked onto a specific signal.

As highlighted in the paper, the SIDA effect for compound **12** was observed in benzene, *m*-xylene, toluene, $CDCl_3$, DCM, and DCE, whereas no splitting of the peaks was observed in 1,4-dioxane, Et_2O, MTBE, THF, MeOH-d_6, MeCN, and DMSO-d_6 (Figure S22-32). No suitable NMR spectra could be obtained for compound **12** in xylene or THF, due to troublesome solvents suppression in combination with this specific compound. Therefore, **1h** was used to investigate the SIDA effect in these two solvents (Figure S33 and S34). Moreover, we observed an overlap of the aromatic signals of **12** with the signals of toluene and benzene, making it hard to determine the enantiomeric ratio by NMR. Consequently, we used the signal of the methoxy group to analyze the SIDA effect in these solvents.

NOTE: The signals used to determine the *e.r.* are highlighted in red (aromatic signal) and blue (methoxy group).

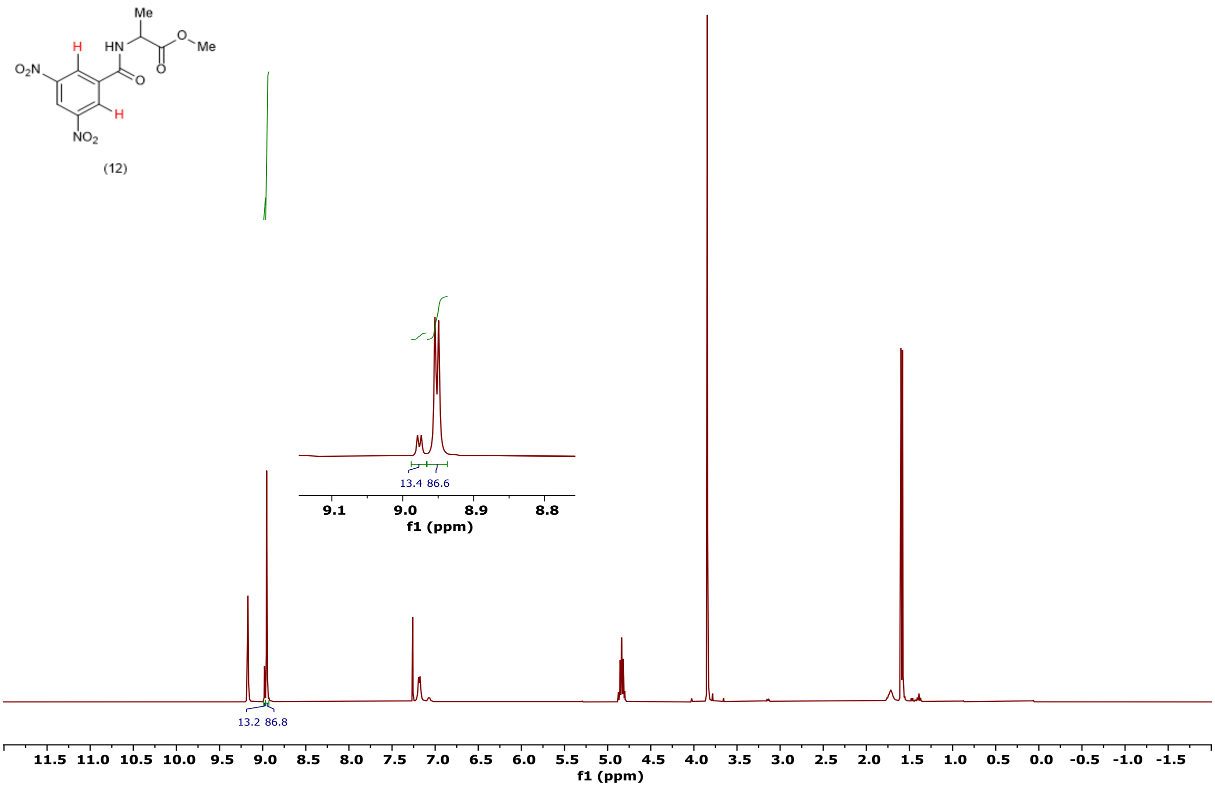

Figure S22: ^{1}H-NMR spectrum of **12** (*e.r.* ~ 90:10) in $CHCl_3$.

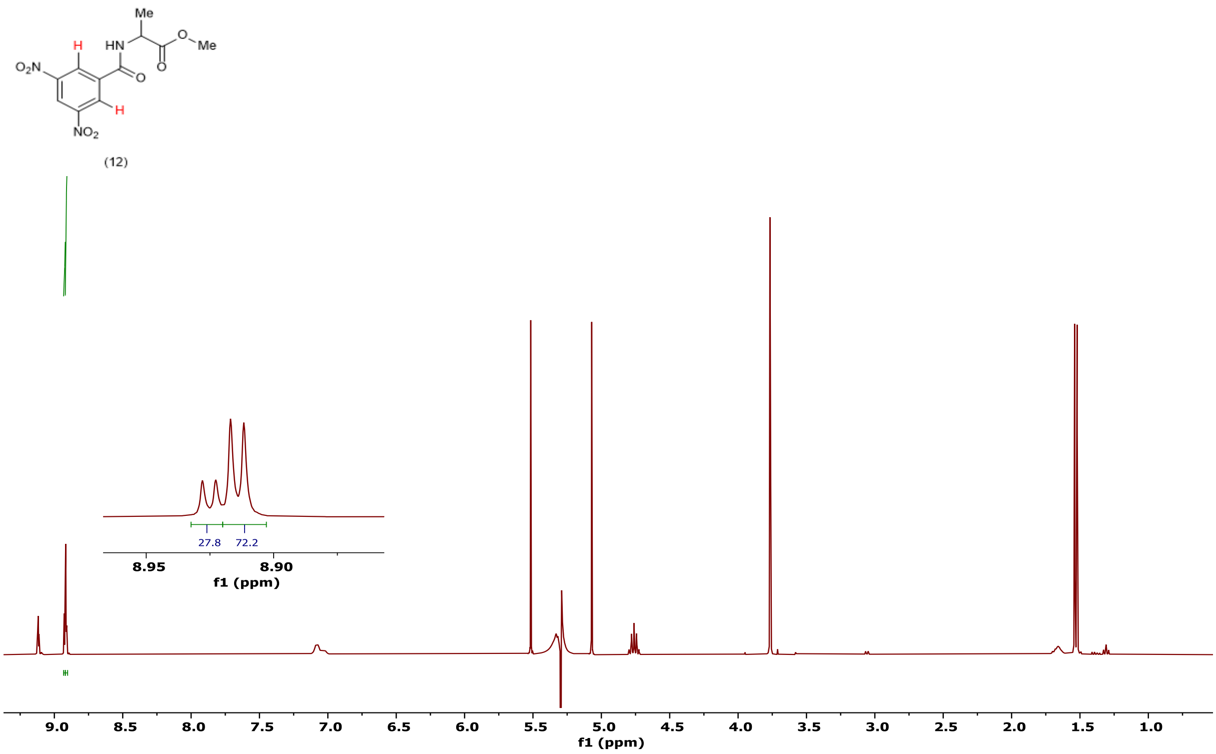

Figure S23: ^{1}H-NMR spectrum of **12** (*e.r.* 70:30) in DCM.

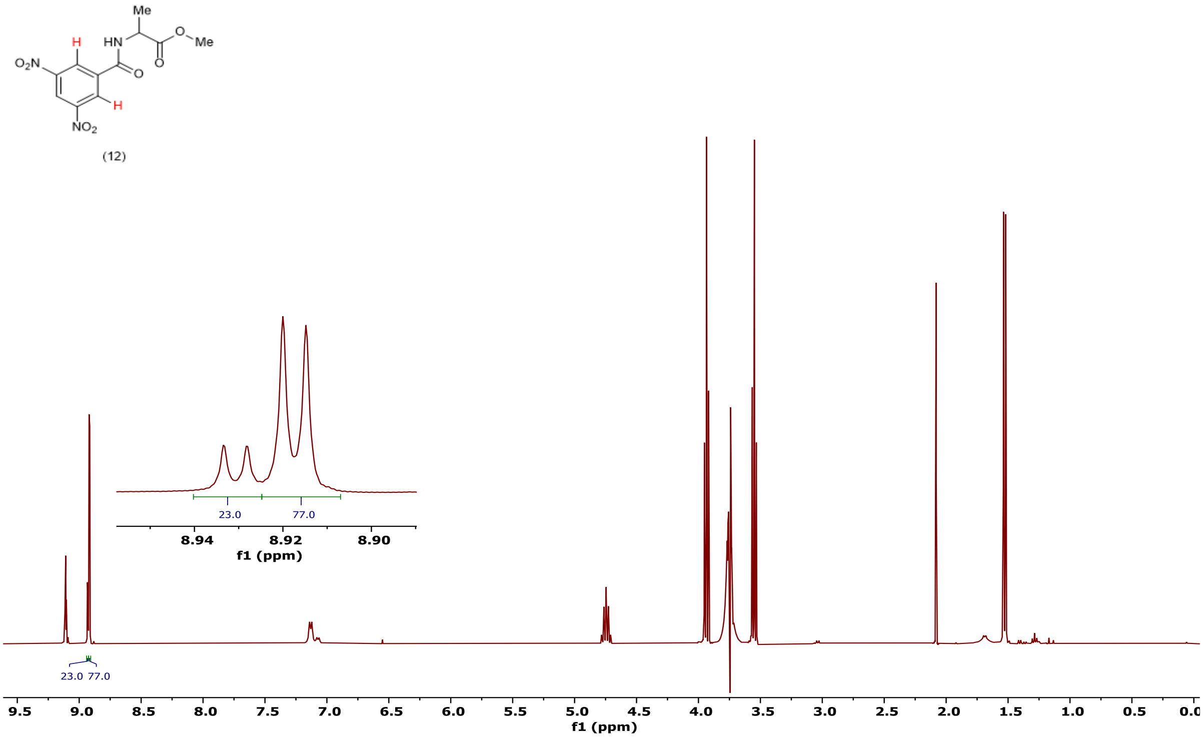

Figure S24: ^{1}H-NMR spectrum of **12** (*e.r.* ~80:20) in DCE.

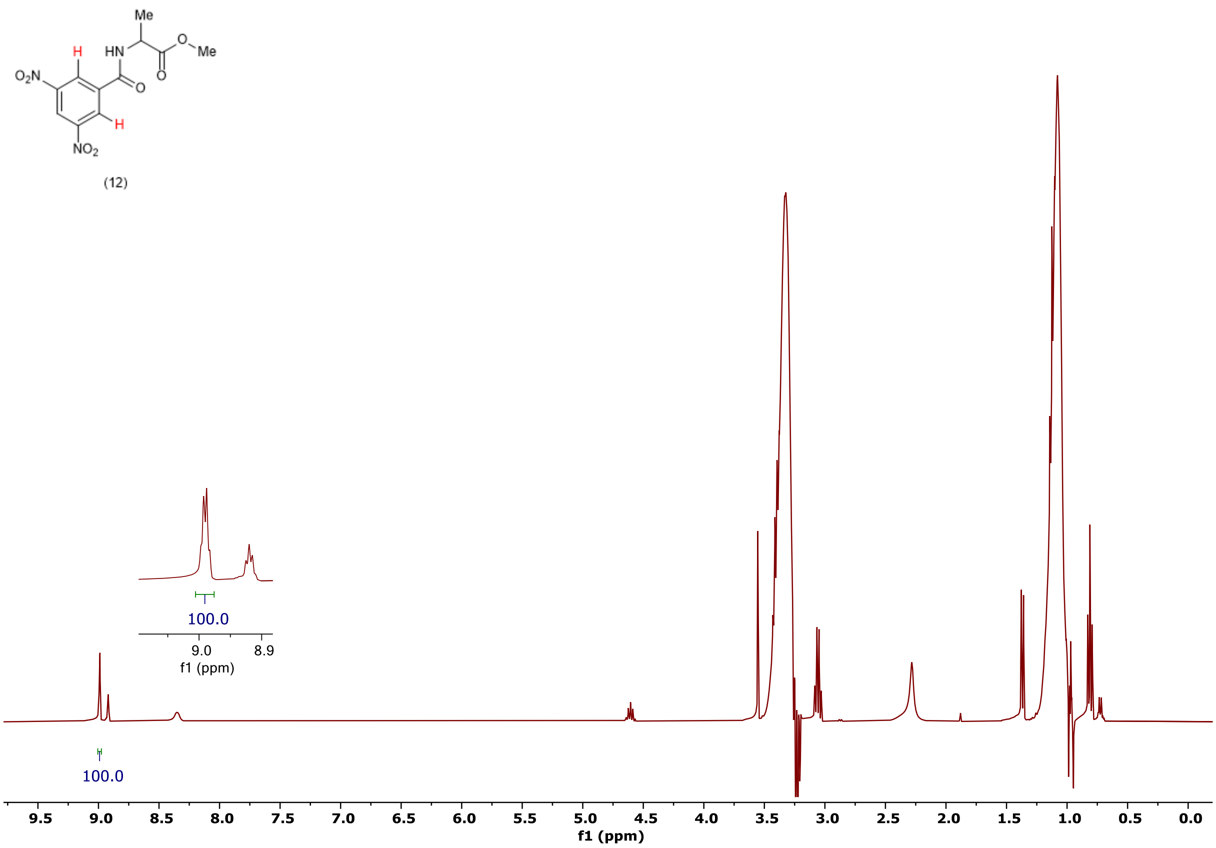

Figure S25: ^{1}H-NMR spectrum of **12** (*e.r.* ~ 80:20) in 1,4-dioxane.

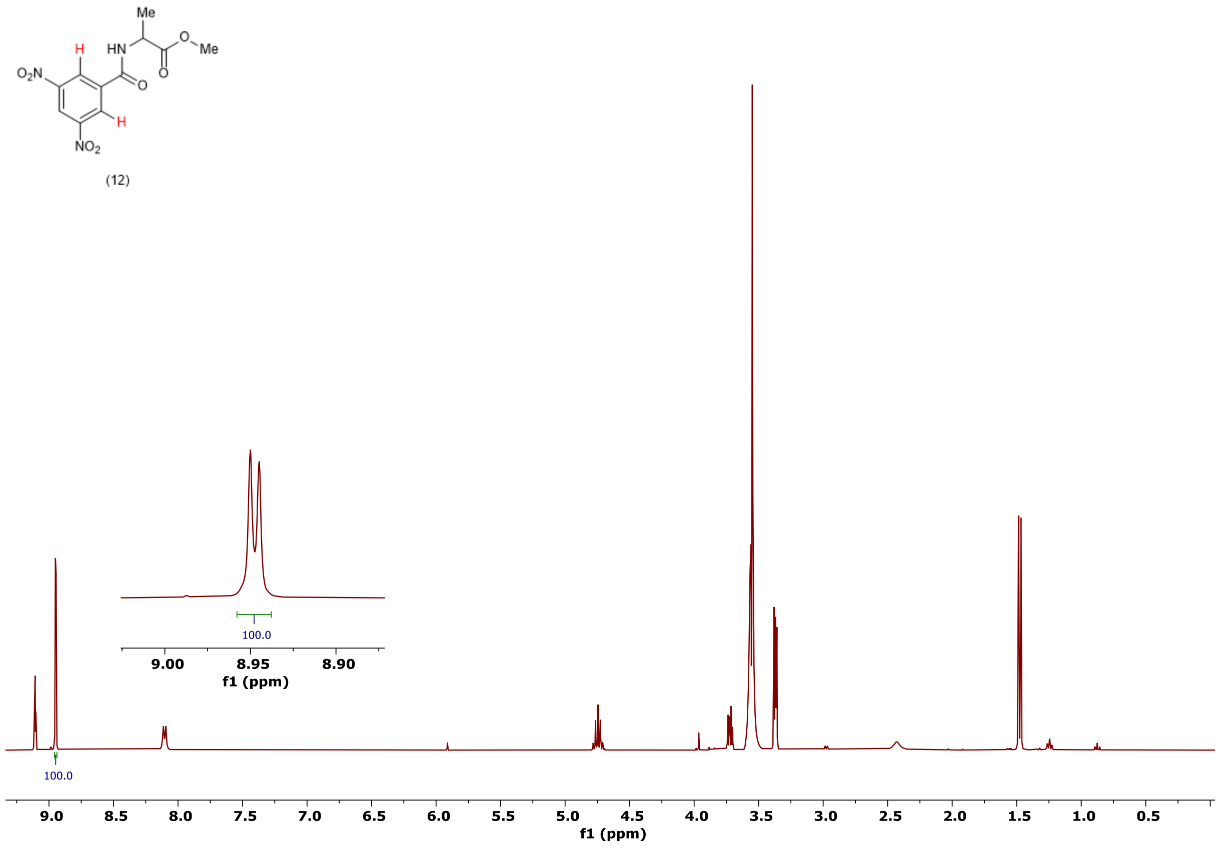

Figure S26: ^{1}H-NMR spectrum of **12** (*e.r.* ~80:20) in Et_2O.

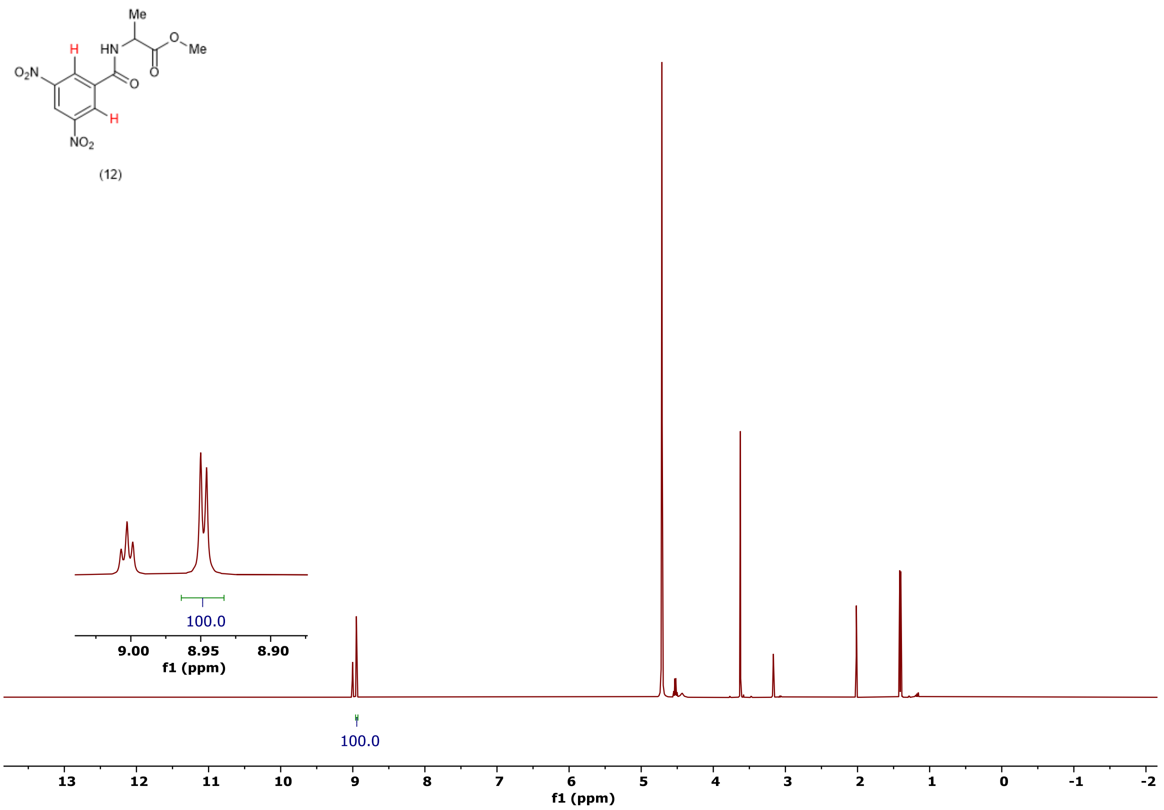

Figure S27: 1H-NMR spectrum of **12** (*e.r.* ~80:20) in MTBE

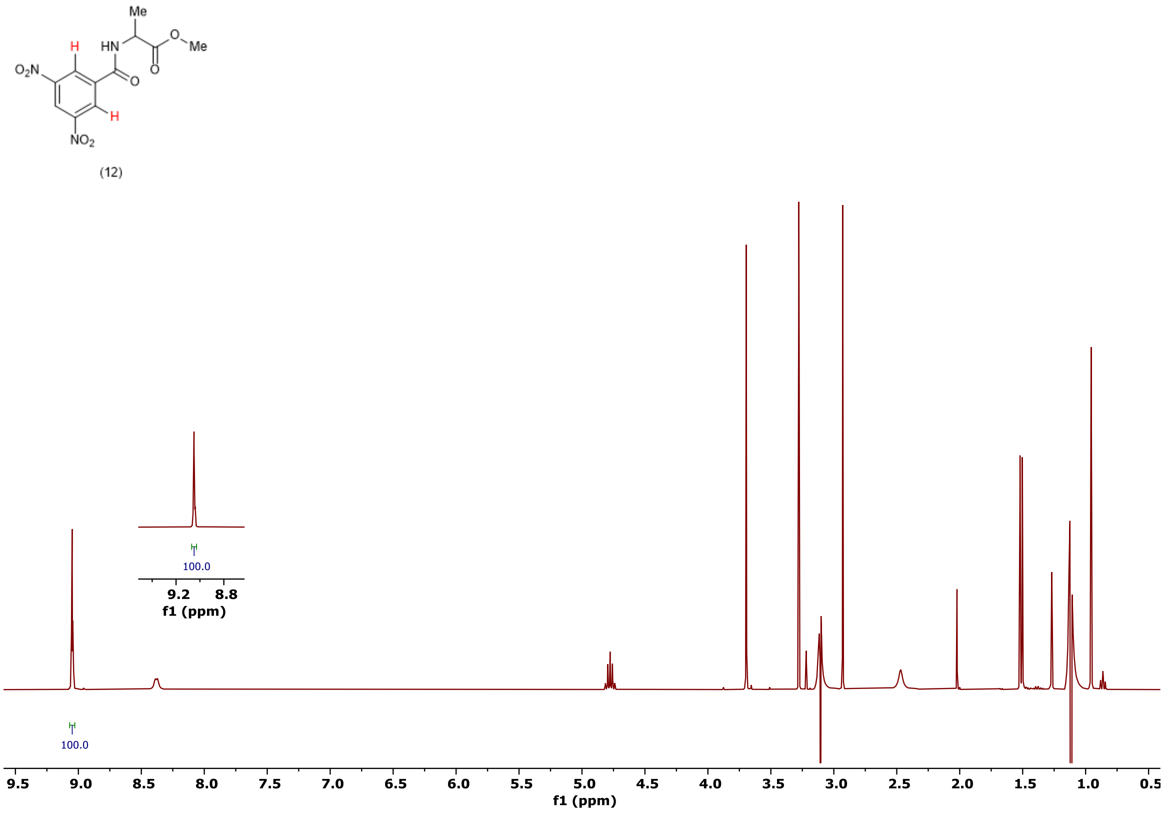

Figure S28: ^{1}H-NMR spectrum of **12** (*e.r.* ~80:20) in MeOH-d_6. The MeOH was contaminated with water (peak at 4.71 ppm).

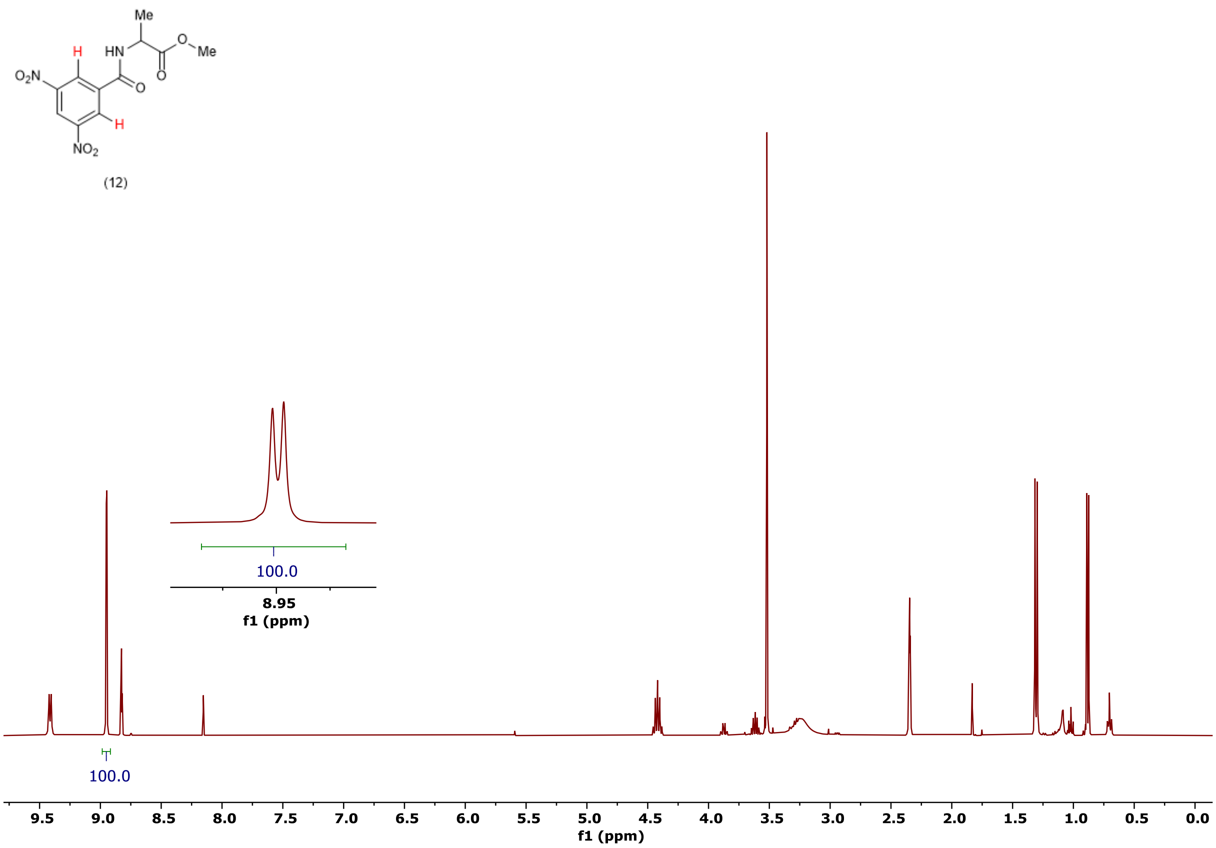

Figure S29: ^{1}H-NMR spectrum of **12** (*e.r.* ~ 80:20) in MeCN.

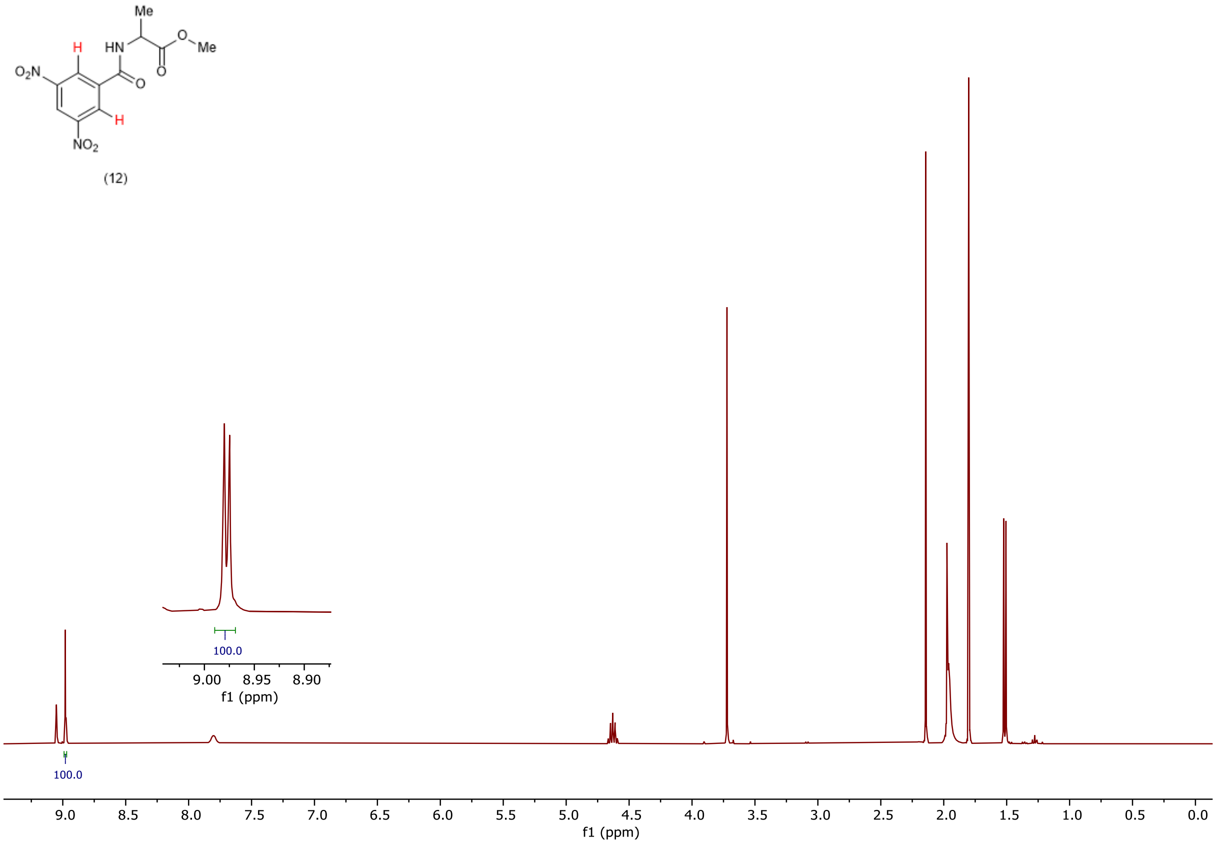

Figure S30: ^{1}H-NMR spectrum of **12** (*e.r.* ~80:20) in DMSO-d_6.

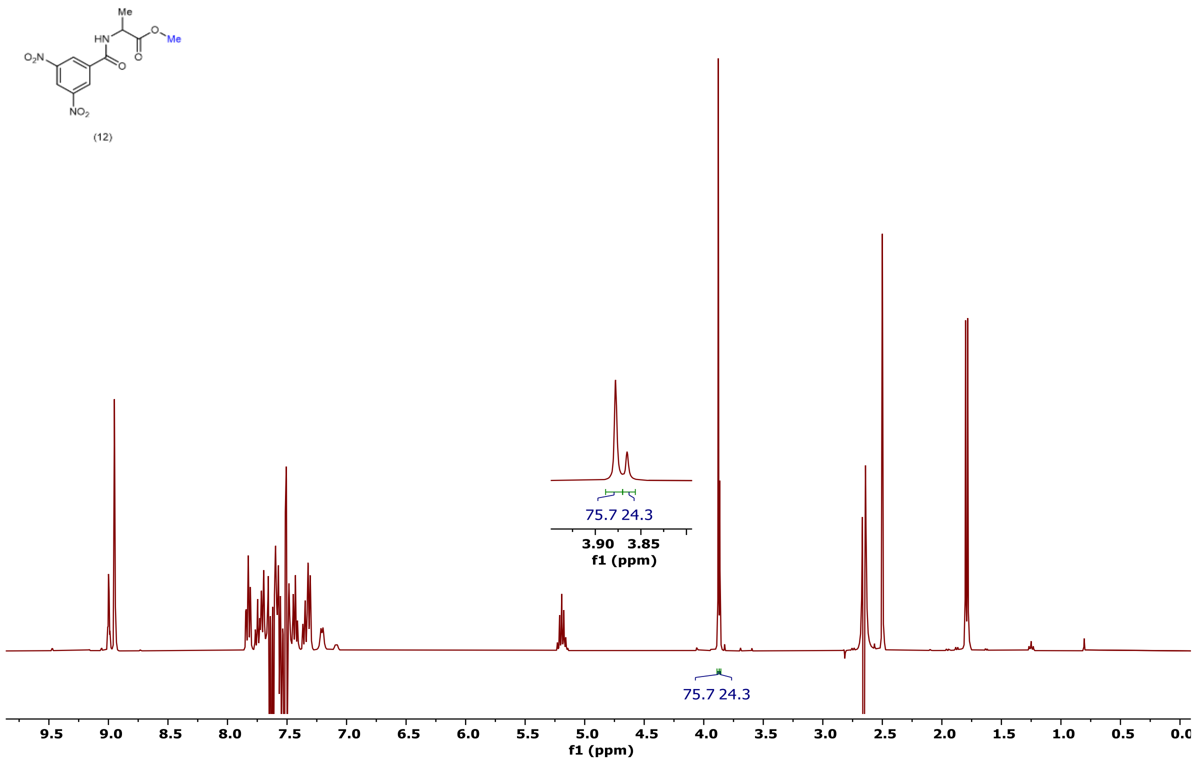

Figure S31: ^{1}H-NMR spectrum of **12** (*e.r.* ~75:25) in toluene.

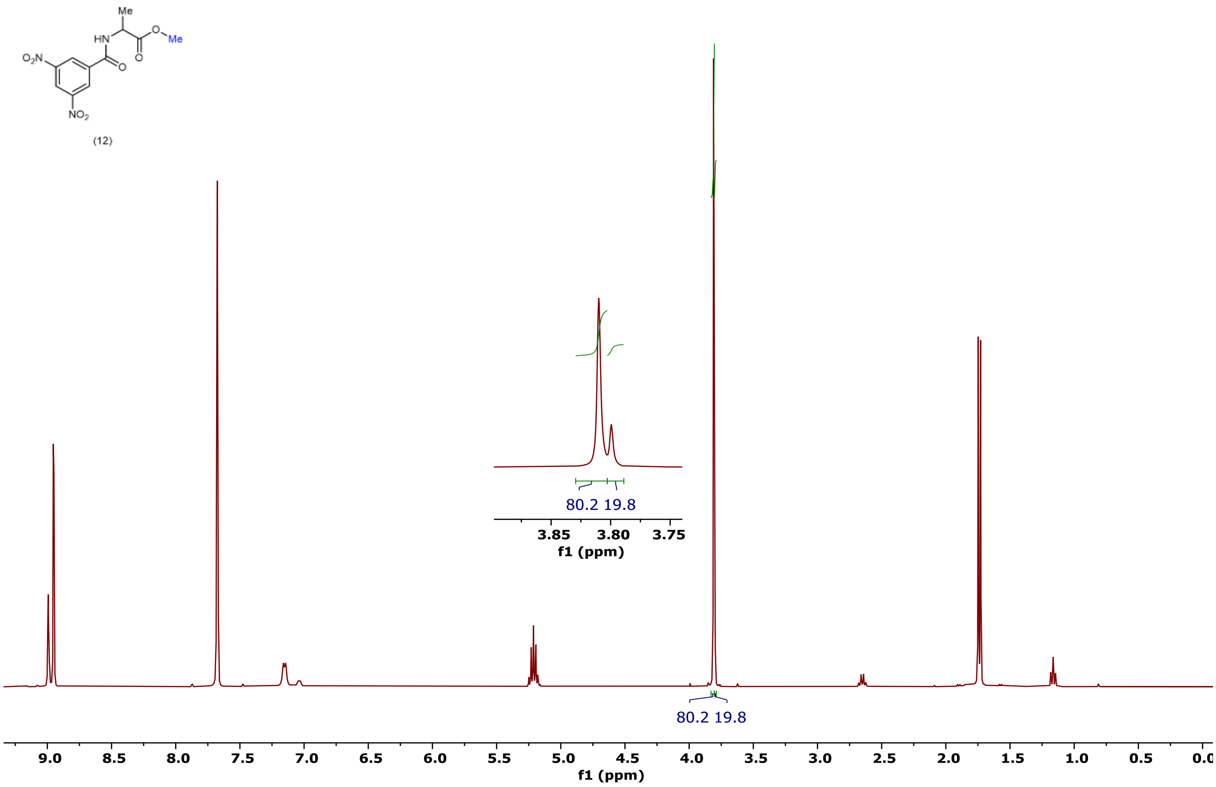

Figure S32: ^{1}H-NMR spectrum of **12** (*e.r.* ~80:20) in toluene-d_8.

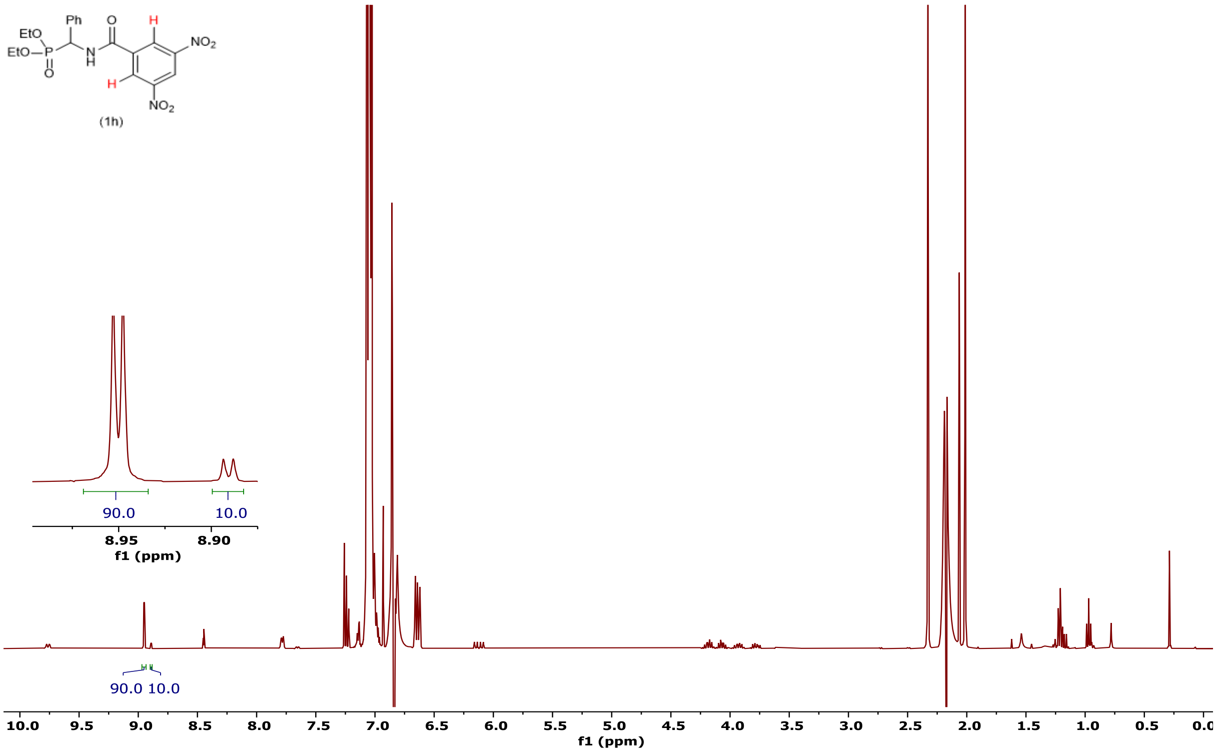

Figure S33: ^{1}H-NMR spectrum of **1h** (*e.r.* 90:10) in xylene.

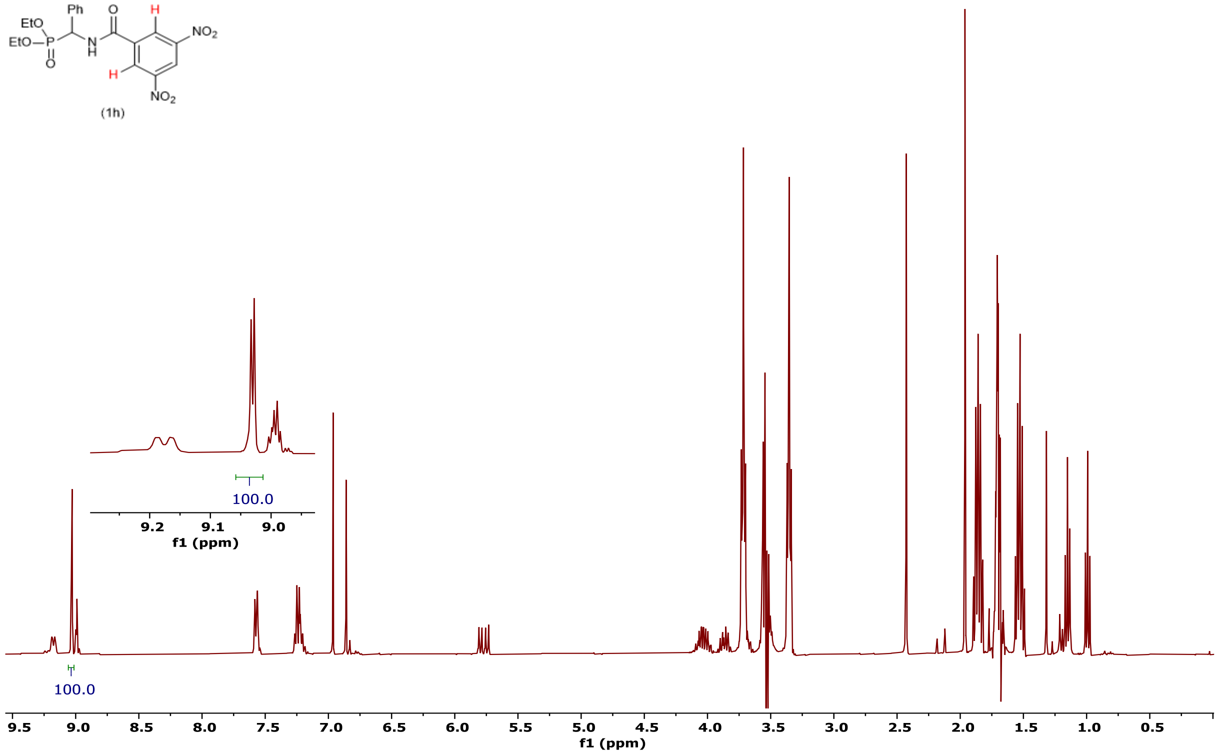

Figure S34: ^{1}H-NMR spectrum of **1h** (*e.r.* 90:10) in THF.

5.2 Functional group interaction profile

Functional Group Interaction Profiles (FGIPs) were built following Hunter´s method using the reported non-covalent interaction parameters α and β for each functional group.[14]

Scheme S18 shows an example of the application of the Functional Group Interaction Profile (FGIP). In solvents such as DMSO, in which the compounds to be analyzed are found in the upper left quadrant (red), no SIDA activity was observed, whereas in solvents such as toluene, in which all compounds are found in the upper right quadrant (green), SIDA activity was observed.

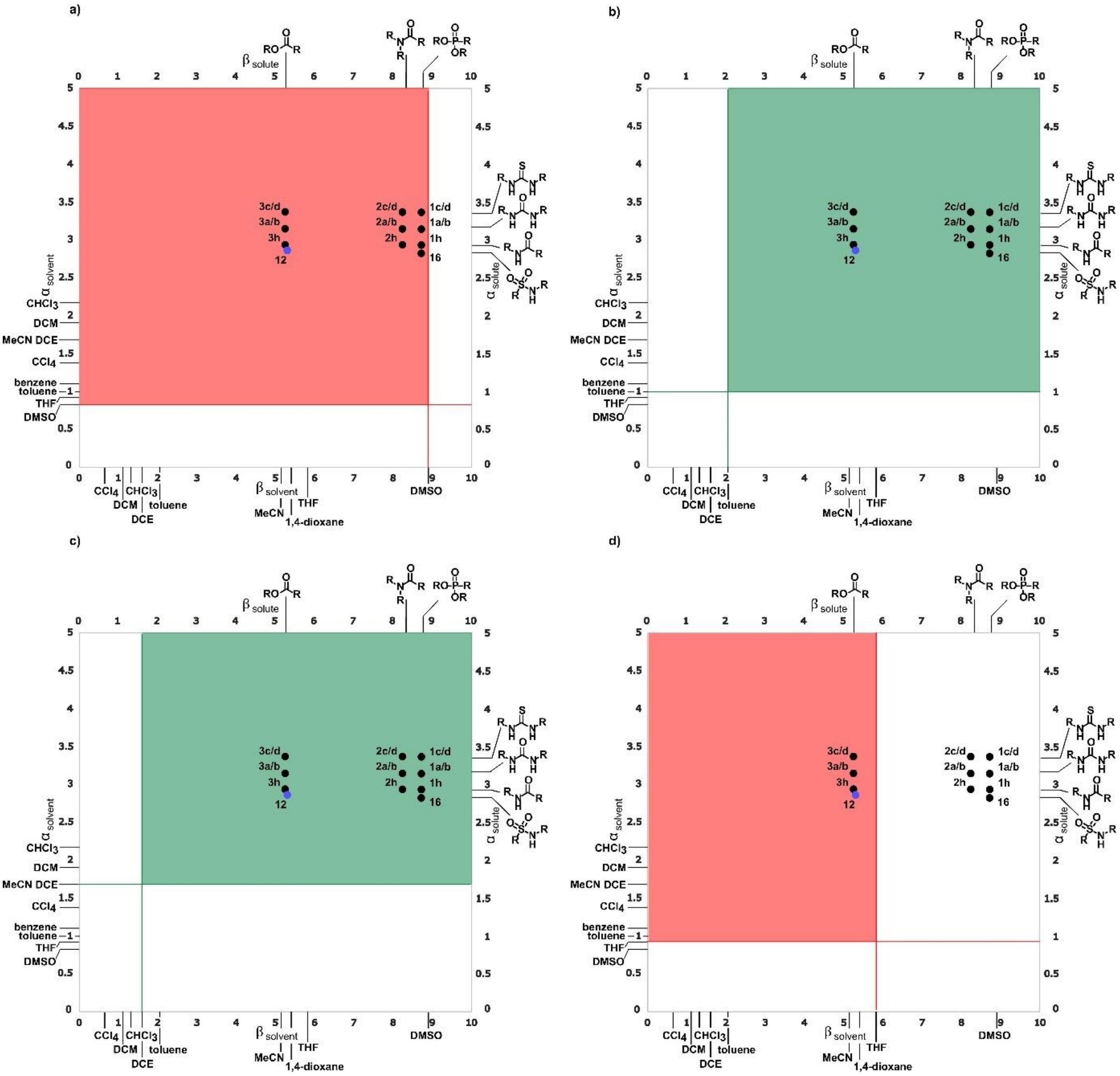

Scheme S18: Functional group interaction profile of commonly used solvents and functional groups. a) DMSO, b) toluene, c) DCE, d) THF.

6. Synthetic procedures

6.1 Compounds prepared following literature procedures

The α-ureidophosphonate **1a**,[15] phosphoramidates **4e**, **5 - 10** were prepared following literature procedures.[2]

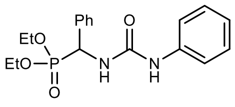
1a

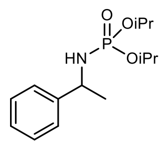
(*S*) and (*R*)-**4e**

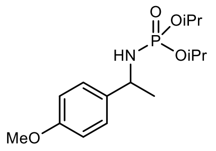
(*S*) and (*R*)-**5**

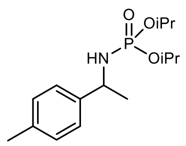
(*S*) and (*R*)-**6**

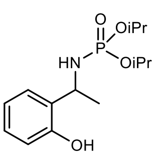
(*S*) and (*R*)-**7**

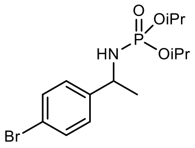
(*S*) and (*R*)-**8**

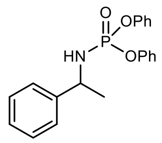
(*S*) and (*R*)-**9**

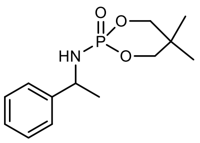
(*S*) and (*R*)-**10**

6.2 Synthesis and characterization of α-amino phosphonates

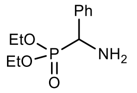
S1

The α-aminophosphonate **S1** used for the synthesis of **1a-1h** was prepared following a literature procedure.[15]

General Procedure **A**:

The amine **S1** (1 equiv.) and a base (Et_3N or pyridine) (1.05 equiv.) were dissolved in DCM (0.2 M) and the solution was cooled to 0 °C. The corresponding electrophile (isocyanate, thioisocyanate, etc.) (1.1 equiv.) was added and the mixture was allowed to warm up to room temperature and stirred overnight. After full conversion of the starting material (TLC monitoring), the reaction was quenched with 0.5 M aq. HCl and diluted with DCM. The layers were separated, and the organic layer was washed with aq. $NaHCO_3$, dried over Na_2SO_4, filtered and concentrated under reduced pressure. The crude product was purified by column chromatography on silica gel.

Diethyl (phenyl(3-phenylureido)methyl)phosphonate (1a)

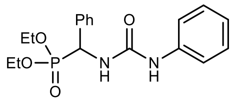

Prepared according to literature procedure.[15]
SIDA active in $CDCl_3$.

Diethyl ((3-hexylureido)(phenyl)methyl)phosphonate (1b)

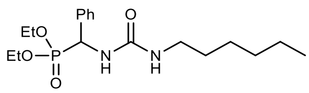

Prepared according to general procedure **A** using α-aminophosphonate **S1** (160 mg, 0.572 mmol, 1.00 equiv.), Et_3N (0.088 mL, 0.63 mmol, 1.1 equiv.) and hexyl isocyanate (73 mg, 0.572 mmol, 1.00 equiv.) in DCM (2.9 mL, 0.2 M). Purification by column chromatography (hexane:EtOAc 8:2) afforded product **1b** as a white solid (179 mg, 0.483 mmol, 85 %). **^{1}H NMR** (400 MHz, $CDCl_3$) δ 7.42 (dt, *J* = 7.8, 1.8

Hz, 2H), 7.24 (dq, *J* = 8.1, 6.3 Hz, 3H), 6.05 (s, 1H), 5.55 – 5.36 (m, 1H), 4.16 (pt, *J* = 7.2, 3.6 Hz, 2H), 3.84 (dp, *J* = 10.2, 7.1 Hz, 1H), 3.72 – 3.58 (m, 1H), 3.13 – 2.96 (m, 3H), 1.29 (t, *J* = 7.0 Hz, 3H), 1.23 – 1.15 (m, 8H), 1.06 (t, *J* = 7.1 Hz, 2H), 0.88 – 0.73 (m, 4H). **^{13}C NMR** (101 MHz, $CDCl_3$) δ 157.7, 136.2, 128.5 (d, *J* = 2.1 Hz), 128.0 (d, *J* = 6.0 Hz), 127.8, 63.7 (d, *J* = 7.2 Hz), 63.3 (d, *J* = 7.4 Hz), 53.4, 40.8, 40.2, 31.5 (d, *J* = 4.8 Hz), 30.2, 26.5 (d, *J* = 2.0 Hz), 22.5 (d, *J* = 2.1 Hz), 16.4 (d, *J* = 5.7 Hz), 16.1 (d, *J* = 5.7 Hz), 14.0. **^{31}P NMR** (162 MHz, $CDCl_3$) δ 23.53, 23.4. **HRMS** (ESI+, *m/z*) calculated for $C_{18}H_{31}N_2O_4PH^+$ $[M+H]^+$: 317.2094, found: 371.2090.
SIDA active in $CDCl_3$.

Diethyl ((3-(3,5-bis(trifluoromethyl)phenyl)thioureido)(phenyl)methyl)phosphonate (1c)

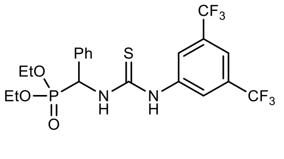

Prepared according to general procedure **A** using α-aminophosphonate **S1** (220 mg, 0.904 mmol, 1.00 equiv.), Et_3N (0.252 mL, 1.809 mmol, 2.00 equiv.) and bis-(3,5-trifluoromethyl)phenyl thioisocyanate (0.165 mL, 0.904 mmol, 1.00 equiv.) in DCM (4.5 mL, 0.2 M). Purification by column chromatography (hexane/EtOAc) afforded product **1c** as a white solid (186 mg, 0.362 mmol, 40%). **^{1}H NMR** (600 MHz, $CDCl_3$) δ 9.94 (s, 1H), 8.91 (s, 1H), 8.02 (s, 2H), 7.56 (dd, *J* = 13.4, 4.8 Hz, 3H), 7.40 (dt, *J* = 12.2, 5.8 Hz, 2H), 7.34 (d, *J* = 7.7 Hz, 1H), 6.70 – 6.52 (m, 1H), 4.43 (d, *J* = 8.5 Hz, 1H), 4.34 (ddt, *J* = 10.0, 7.0, 5.0 Hz, 1H), 4.03 – 3.94 (m, 1H), 3.84 – 3.77 (m, 1H), 1.46 (q, *J* = 6.5 Hz, 3H), 1.17 – 1.12 (m, 3H). **^{31}P NMR** (243 MHz, $CDCl_3$) 22.13, 22.10. **^{19}F NMR** (565 MHz, $CDCl_3$) δ -62.99. **^{13}C NMR** (151 MHz, $CDCl_3$) δ 181.26 (d, *J* = 12.3 Hz), 141.01, 134.08, 131.55 (q, *J* = 33.7 Hz), 128.92 (d, *J* = 2.1 Hz), 128.78, 128.22 (t, *J* = 5.2 Hz), 124.95 (q, *J* = 273.0 Hz), 117.35, 64.77 (dd), 64.35 (dd), 53.92, 52.91, 16.48 (t, *J* = 5.1 Hz), 15.91 (d, *J* = 6.0 Hz).**HRMS** (ESI+, *m/z*) calculated for $C_{20}H_{21}F_6N_2O_3PSH^+$ $[M+H]^+$: 515.0987, found: 515.0987.
SIDA active in $CDCl_3$.

Diethyl ((3-butylthioureido)(phenyl)methyl)phosphonate (1d)

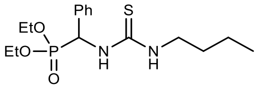

Prepared according to general procedure **A** using α-aminophosphonate **S1** (160 mg, 0.572 mmol, 1.00 equiv.), Et_3N (0.088 mL, 0.63 mmol, 1.1 equiv.) and butyl isothiocyanate (66 mg, 0.572 mmol, 1.00 equiv.) in DCM (2.9 mL, 0.2 M). Product **1d** was obtained as a white solid (185 mg, 0.516 mmol, 90%) without further purification. **^{1}H NMR** (400 MHz, toluene-*d_8*): 7.78 – 7.62 (m, 2H), 7.17 (t, *J* = 7.6 Hz, 2H), 4.35 – 4.21 (m, 1H), 4.13 (dtd, *J* = 14.2, 7.1, 2.2 Hz, 1H), 3.79 – 3.41 (m, 4H), 2.58 – 2.50 (m, 2H), 1.16 (t, *J* = 7.1 Hz, 3H), 1.00 – 0.85 (m, 5H), 0.76 (dt, *J* = 13.1, 7.1 Hz, 6H), 0.61 – 0.53 (m, 3H). **^{13}C NMR** (101 MHz, $CDCl_3$) δ 135.3, 128.6 (d, *J* = 2.0 Hz), 128.4 (d, *J* = 6.1 Hz), 128.1 (d, *J* = 2.6 Hz), 64.0 (d, *J* = 7.0 Hz), 63.6 (d, *J* = 7.5 Hz), 44.7, 31.2, 20.1, 16.4 (d, *J* = 5.9 Hz), 16.1 (d, *J* = 5.9 Hz), 13.8. **^{31}P NMR** (162 MHz, $CDCl_3$) δ 22.84.
NOTE: Not satisfactory HRMS could be obtained for this compound.
SIDA active in $CDCl_3$.

Diethyl ((diethoxyphosphoryl)(phenyl)methyl)phosphoramidate (1e)

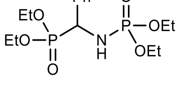

Prepared according to general procedure **A** using α-aminophosphonate **S1** (182 mg, 0.750 mmol, 1.00 equiv.), pyridine (0.066 mL, 0.825 mmol, 1.1 equiv.) and diethyl phosphorochloridate (0.12 mL, 0.825 mmol, 1.00 equiv.) in DCM (7.5 mL, 0.1 M). Purification by column chromatography (hexane/^{i}PrOH 9:1) afforded product **1e** as a colorless oil (200 mg, 0.527 mmol, 70%). **^{1}H NMR** (400 MHz, $CDCl_3$) δ 7.41 – 7.34 (m, 2H), 7.34 – 7.20 (m, 3H), 4.53 – 4.37 (m, 1H), 4.27 – 4.16 (m, 2H), 4.09 (ttd, *J* = 7.1, 5.4, 2.0 Hz, 2H), 4.02 – 3.79 (m, 3H), 3.69 – 3.53 (m, 2H), 1.38 – 1.18 (m, 10H), 1.02 (dtd, *J* = 23.5, 7.1, 1.5 Hz, 6H). **^{13}C NMR** (151 MHz, $CDCl_3$) δ 136.7, 128.5 (d, *J* = 2.3 Hz), 128.1 (d, *J* = 2.9 Hz), 127.9 (d, *J* = 5.9 Hz), 63.4 (t, *J* = 7.1 Hz), 63.1 (d, *J* = 7.1 Hz), 62.5 (d, *J* = 5.3 Hz), 62.5 (d, *J* = 4.9 Hz), 53.6, 52.6, 16.4 (d, *J* = 6.0 Hz), 16.1 (d, *J* = 5.7 Hz), 16.1 (dd, *J* = 7.2, 2.4 Hz), 15.7 (d, *J* = 7.6 Hz).. **^{31}P NMR** (162 MHz, $CDCl_3$) δ 22.3, 22.1, 6.8, 6.5, -13.2. **HRMS** (ESI+, *m/z*) calculated for $C_{15}H_{27}NO_6P_2$ $[M+H]^+$: 380.1382, found: 380.1386.
SIDA inactive

Diethyl (((diphenylphosphoryl)amino)(phenyl)methyl)phosphonate (1f)

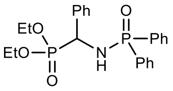

Prepared according to general procedure **A** using α-aminophosphonate **S1** (220mg, 0.904 mmol, 1.00 equiv.), DIPEA (0.173 mL, 0.995 mmol, 1.10 equiv.) and diphenylphosphonic chloride (224 mg, 0.950 mmol, 1.05 equiv.) in DCM (4.5 mL, 0.2 M). Product **1f** was obtained as a white solid (120 mg, 0.516 mmol, 28%) without further purification. **^{1}H NMR** (400 MHz, $CDCl_3$) δ 7.68 – 7.59 (m, 1H), 7.44 (d, *J* = 8.1 Hz, 2H), 7.41 – 7.20 (m, 3H), 7.14 (s, 1H), 4.25 (d, *J* = 17.2 Hz, 1H), 4.09 – 4.00 (m, 2H), 4.00 – 3.91 (m, 1H), 3.91 – 3.77 (m, 1H), 3.70 – 3.51 (m, 1H), 1.53 (dd, *J* = 12.2, 7.0 Hz, 3H), 1.43 (d, *J* = 6.7 Hz, 2H), 1.26 (t, *J* = 7.2 Hz, 4H), 1.16 (t, *J* = 7.1 Hz, 3H). **^{31}P NMR** (162 MHz, $CDCl_3$) δ 24.60. **^{13}C NMR** (151 MHz, $CDCl_3$) δ 131.2, 128.5 (d, *J* = 2.4 Hz), 128.1, 127.9, 63.0 (d, *J* = 23.2 Hz), 53.6, 16.4 (d, *J* = 5.5 Hz), 16.3 (d, *J* = 5.7 Hz). **HRMS** (ESI+, *m/z*) calculated for $C_{23}H_{27}NO_4P_2$ $[M+H]^+$: 443.1428, found: 443.1430.
SIDA inactive
Note: Aromatic signals overlap with $CDCl_3$ peak.

Diethyl (phenyl(2,2,2-trifluoroacetamido)methyl)phosphonate (1g)

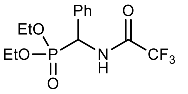

Prepared according to general procedure **A** using α-aminophosphonate **S1** (184 mg, 0.760 mmol, 1.00 equiv.), Et_3N (0.120 mL, 0.863 mmol, 1.14 equiv.) and trifluoroacetic anhydride (0.12 mL, 0.798 mmol, 1.05 equiv.) in DCM (7.5 mL, 0.1 M). Purification by column chromatography (Et_2O/DCM 8:2) afforded product **1g** as a white solid (214 mg, 0.631 mmol, 83%). **^{1}H NMR** (400 MHz, $CDCl_3$): 9.14 (s, 1H), 7.59 – 7.48 (m, 2H), 7.39 – 7.27 (m, 3H), 5.57 – 5.43 (m, 1H), 4.16 (p, *J* = 7.3 Hz, 2H), 3.92 (dp, *J* = 10.3, 7.2 Hz, 1H), 3.79 – 3.62 (m, 1H), 1.39 – 1.18 (m, 3H), 1.09 (t, *J* = 7.1 Hz, 3H).**^{13}C NMR** (151 MHz, DMSO) δ 156.7 (qd, *J* = 37.1, 7.1 Hz), 134.2, 129.0 (d, *J* = 5.8 Hz), 128.9, 128.7, 116.3 (q, *J* = 287.8 Hz), 63.2 (dd, *J* = 10.6, 6.8 Hz), 51.5, 50.5, 16.6 (d, *J* = 4.8 Hz), 16.4 (d, *J* = 5.3 Hz). **^{31}P NMR** (162 MHz, $CDCl_3$) δ 18.8. **^{19}F NMR** (376 MHz, $CDCl_3$) δ -69.3, -75.0. **HRMS** (ESI+, *m/z*) calculated for $C_{13}H_{17}F_3NO_4P$ $[M+H]^+$: 340.0920, found: 340.0916.
Data in accordance with literature.[16]
SIDA active in $CDCl_3$.

Diethyl ((3,5-dinitrobenzamido)(phenyl)methyl)phosphonate (1h)

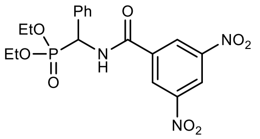

Prepared according to general procedure **A** using α-aminophosphonate **S1** (239 mg, 0.983 mmol, 1.00 equiv.), Et_3N (0.151 mL, 1.081 mmol, 1.10 equiv.) and 3,5-dinitrobenzoyl chloride (249 mg, 1.081 mmol, 1.10 equiv.) in DCM (9.8 mL, 0.1 M). Purification by column chromatography (hexane/EtOAc 6:4) afforded product **1h** as a white foam (273 mg, 0.624 mmol, 64%). **^{1}H NMR** (400 MHz, $CDCl_3$) δ 9.78 (q, *J* = 10.8 Hz, 1H), 9.21 (d, *J* = 2.1 Hz, 2H), 9.10 – 9.03 (m, 1H), 7.71 (d, *J* = 7.3 Hz, 2H), 7.35 – 7.21 (m, 2H), 5.97 – 5.85 (m, 1H), 4.13 (dqt, *J* = 14.3, 7.2, 2.6 Hz, 2H), 4.00 (dt, *J* = 9.7, 7.1 Hz, 1H), 3.75 (dtd, *J* = 16.6, 12.2, 4.8 Hz, 1H), 1.23 (t, *J* = 7.2 Hz, 3H), 1.09 (t, *J* = 7.0 Hz, 3H).**^{13}C NMR** (151 MHz, DMSO) δ 163.13 (d, *J* = 7.6 Hz), 148.56, 136.54, 135.46, 129.07, 129.03, 128.79, 128.68, 128.43 (d, *J* = 2.7 Hz), 121.71, 63.10 (dd, *J* = 20.7, 6.5 Hz), 51.72, 50.69, 16.80 (d, *J* = 4.9 Hz), 16.52 (d, *J* = 5.4 Hz). **^{31}P NMR** (162 MHz, $CDCl_3$) δ 19.64, 19.02 **HRMS** (ESI+, *m/z*) calculated for $C_{18}H_{20}N_3O_8P$ $[M+H]^+$: 340.0920, found: 340.0919.

SIDA active in $CDCl_3$.

6.3 Synthesis and characterization of α-amino amides

General Procedure **B** for the synthesis of α-amino amine **S2**:

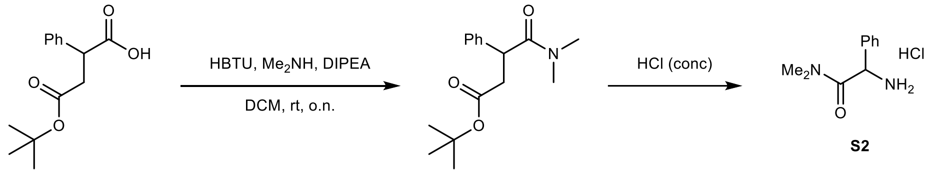

The HCl salt of α-amino amine **S2** used for the synthesis of **2a-2h** was synthesized enantiopure. An oven-dried 50-mL round-bottomed flask was charged with Boc-D/L-phenylglycine (1.00 equiv.), and HBTU (1.10 equiv.). Anhydrous DCM (0.5 M), dimethylamine solution (1.20 equiv.) and *N*,*N*-diisopropylethylamine (2.00 equiv.) were added sequentially via a syringe. The flask was capped with a plastic stopper, and the reaction mixture was stirred at room temperature overnight. The resulting mixture was diluted with Et_2O (100 mL), washed with aq. 1 N HCl (2x), sat. $NaHCO_3$, water (2x) and brine. The organic phase was dried over Na_2SO_4 and concentrated under reduced pressure to give a pale yellow solid. The product was pure enough for further reactions. The product was dissolved in DCM and 2 mL of concentrated HCl were added. After 1h, the solvents were removed under remove pressure to give the HCl salt of α-amino amine **S2**.

General procedure **C** for the synthesis of α-amino amide **2a – 2h**:

To a stirred solution of the amine **S2** (1.00 equiv.) and Et_3N (1.50 equiv.) in anhydrous DCM (0.2 M) at 0 °C, the corresponding electrophile (isocyanate, thioisocyanate) (1.10 equiv.) was added dropwise. The reaction mixture was allowed to warm up to room temperature overnight and was quenched with aq. HCl (1 M) and extracted with DCM (3x). The organic phase was dried over Na_2SO_4 and concentrated under reduced pressure. The product was purified by column chromatography on silica gel or by crystallization.

(*S*)-2-(3-(3,5-bis(trifluoromethyl)phenyl)ureido)-*N*,*N*-dimethyl-2-phenylacetamide ((*S*)-2a)

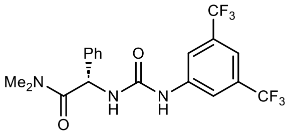

Prepared according to general procedure **C** using α-amino amine salt (*S*)-**S2** (400 mg, 1.863 mmol, 1.00 equiv.), Et_3N (0.389 mL, 2.795 mmol, 1.50 equiv.) and 3,5-bis(trifluoromethyl)phenyl isocyanate (0.354 mL, 2.049 mmol, 1.10 equiv.) in DCM (9.3 mL, 0.2 M). Crystallization from Et_2O/DCM afforded product **(*S*)-2a** as a white solid (712 mg, 1.643 mmol, 88%). **^{1}H NMR** (400 MHz, $CDCl_3$) δ 8.69 (s, 1H), 7.70 (d, *J* = 1.6 Hz, 2H), 7.31 (s, 4H), 7.25 – 7.19 (m, 2H), 7.04 (s, 1H), 5.93 (s, 1H), 3.05 (d, *J* = 14.4 Hz, 6H). **^{13}C NMR** (101 MHz, $CDCl_3$) δ 172.7, 154.6, 140.9, 135.9, 131.6 (q, *J* = 33.1 Hz), 129.4, 128.7, 127.7, 123.2 (q, *J* = 272.8 Hz), 117.7, 115.0, 54.9, 37.4, 36.4. **^{19}F NMR** (376 MHz, $CDCl_3$) δ - 63.2. **HRMS** (ESI-, *m/z*) calculated for $C_{19}H_{16}F_6N_3O_2$ $[M-H]^-$: 432.1152, found: 432.1146.
SIDA active in $CDCl_3$.

(*R*)-2-(3-(3,5-bis(trifluoromethyl)phenyl)ureido)-*N*,*N*-dimethyl-2-phenylacetamide ((*R*)-2a)

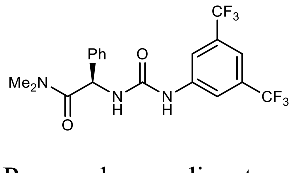

Prepared according to general procedure **C** using α-amino amine salt (*R*)-**S2** (400 mg, 1.863 mmol, 1.00 equiv.), Et_3N (0.389 mL, 2.795 mmol, 1.50 equiv.) and 3,5-bis(trifluoromethyl)phenyl isocyanate (0.354 mL, 2.049 mmol, 1.10 equiv.) in DCM (9.3 mL, 0.2 M). Crystallization from Et_2O/DCM afforded the product **(*R*)-2a** as a white solid (697 mg, 1.608 mmol, 86%). **^{1}H NMR** (400 MHz, $CDCl_3$) δ 8.74 (s, 1H), 7.72 (d, *J* = 1.5 Hz, 2H), 7.35 – 7.27 (m, 5H), 7.25 – 7.20 (m, 2H), 5.94 (s, 1H), 3.05 (d, *J* = 10.0 Hz, 6H).**^{13}C NMR** (101 MHz, $CDCl_3$) δ 172.7, 154.6, 140.9, 135.9, 131.6 (q, *J* = 33.1 Hz), 129.4, 127.7, 123.2 (d, *J* = 272.8 Hz), 117.7, 115.0, 54.9, 37.4, 36.4. **^{19}F NMR** (376 MHz, $CDCl_3$) δ -63.21, -63.17. **HRMS** (ESI-, *m/z*) calculated for $C_{19}H_{16}F_6N_3O_2$ $[M-H]^-$: 434.1298, found: 434.1298.
SIDA active in $CDCl_3$.

(*S*)-2-(3-hexylureido)-*N*,*N*-dimethyl-2-phenylacetamide ((*S*)-2b)

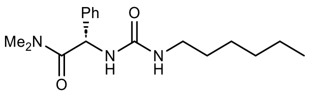

Prepared according to general procedure **C** using α-amino amine salt (*S*)-**S2** (200 mg, 0.932 mmol, 1.00 equiv.), Et_3N (0.0.194 mL, 1.397 mmol, 1.5 equiv.) and hexyl isocyanate (0.130 mg, 1.025 mmol, 1.10 equiv.) in DCM (4.7 mL, 0.2 M). Purification by column chromatography (hexane/ EtOAc 7:3) afforded product **(*S*)-2b** as a white solid (68 mg, 0.223 mmol, 24%). **^{1}H NMR** (600 MHz, $CDCl_3$) δ 7.43 – 7.30 (m, 5H), 6.04 (d, *J* = 7.7 Hz, 1H), 5.83 (dd, *J* = 7.7, 3.5 Hz, 1H), 4.61 (s, 1H), 3.22 – 3.09 (m, 2H), 3.00 (s, 3H), 2.94 (s, 3H), 1.47 (s, 2H), 1.33 – 1.25 (m, 6H), 0.91 – 0.86 (m, 3H). **^{13}C NMR** (101 MHz, $CDCl_3$) δ 171.3, 157.4, 138.5, 128.9, 127.9, 127.7, 54.5, 40.5, 37.0, 36.0, 31.5, 30.2, 26.5, 22.5, 14.0. **HRMS** (ESI+, *m/z*) calculated for $C_{17}H_{27}N_3O_2Na^+$ $[M+Na]^+$: 328.1995, found: 328.1990.
SIDA active in toluene-d_8.

(*R*)-2-(3-hexylureido)-*N*,*N*-dimethyl-2-phenylacetamide ((*R*)-2b)

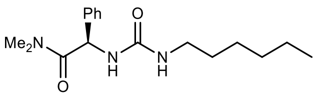

Prepared according to general procedure **C** using α-amino amine salt (*R*)-**S2** (200 mg, 0.932 mmol, 1.00 equiv.), Et_3N (0.194 mL, 1.397 mmol, 1.5 equiv.) and hexyl isocyanate (0.130 mg, 1.025 mmol, 1.10 equiv.) in DCM (4.7 mL, 0.2 M). Purification by column chromatography (hexane/ EtOAc 7:3) afforded product **(*R*)-2b** as a white solid (71 mg, 0.232 mmol, 25%). **^{1}H NMR** (600 MHz, $CDCl_3$) δ 7.43 – 7.38 (m, 2H), 7.38 – 7.29 (m, 3H), 6.09 (d, *J* = 7.7 Hz, 1H), 5.84 (dd, *J* = 7.7, 4.0 Hz, 1H), 4.68 (s, 1H), 3.22 – 3.09 (m, 2H), 3.00 (s, 3H), 2.94 (d, *J* = 0.8 Hz, 3H), 1.51 – 1.42 (m, 2H), 1.34 – 1.24 (m,

6H), 0.92 – 0.86 (m, 3H). **^{13}C NMR** (101 MHz, $CDCl_3$) 171.5, 157.6, 138.5, 128.9, 127.9, 127.7, 54.4, 40.4, 37.1, 36.0, 31.5, 30.2, 26.6, 22.5, 14.0.
NOTE: Not satisfactory HRMS could be obtained for this compound.
SIDA active in toluene-d_8.

2-(3-(3,5-bis(trifluoromethyl)phenyl)thioureido)-*N*,*N*-dimethyl-2-phenylacetamide (2c)

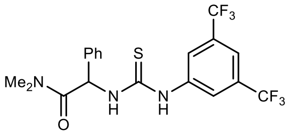

Prepared according to general procedure **C** using α-amino amine salt **S2** (107 mg, 0.500 mmol, 1.00 equiv.), Et_3N (0.104 mL, 0.750 mmol, 1.50 equiv.) and 3,5-bis(trifluoromethyl)phenyl isothiocyanate (0.1 mL, 0.550 mmol, 1.10 equiv.) in DCM (2.5 mL, 0.2 M). Purification by column chromatography (hexane/ EtOAc 6:4) afforded product **2c** as a white solid (136 mg, 0.303 mmol, 61 %). **^{1}H NMR** (400 MHz, $CDCl_3$) δ 9.68 – 9.62 (m, 1H), 8.63 – 8.53 (m, 1H), 8.07 (s, 2H), 7.49 (d, *J* = 12.7 Hz, 1H), 7.43 – 7.22 (m, 4H), 7.22 – 7.15 (m, 2H), 6.58 (d, *J* = 7.4 Hz, 1H), 3.12 (d, *J* = 6.8 Hz, 3H), 3.02 (dd, *J* = 6.9, 1.2 Hz, 3H). **^{13}C NMR** (151 MHz, $CDCl_3$) δ 180.9, 172.5, 140.5, 140.4, 134.7, 131.2 (q, *J* = 33.5 Hz), 129.4, 128.8, 127.9, 123.2 (q, *J* = 272.7 Hz), 122.7, 117.5, 58.8, 37.4, 36.4.**^{19}F NMR** (376 MHz, $CDCl_3$) δ -63.1, -63.1. **HRMS** (ESI-, *m/z*) calculated for $C_{19}H_{16}F_6N_3SO$ [M-H]$^-$: 448.0913, found: 448.0922. Data in accordance with literature[17]
SIDA active in $CDCl_3$.

(*S*)-2-(3-butylthioureido)-*N*,*N*-dimethyl-2-phenylacetamide ((*S*)-2d)

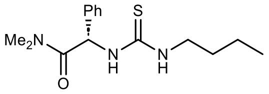

Prepared according to general procedure **C** using α-amino amine salt ***(S)*-S2** (315 mg, 1.467 mmol, 1.00 equiv.), Et_3N (0.306 mL, 2.201 mmol, 1.5 equiv.) and butyl isothiocyanate (0.187 mL, 1.614 mmol, 1.10 equiv.) in DCM (7.3 mL, 0.2 M). Crystallization from Et_2O/DCM afforded the product **(*S*)-2d** as a white solid (0.096 mg, 0.327 mmol, 22%). **^{1}H NMR** (^{1}H NMR (400 MHz, $CDCl_3$) δ 7.39 (s, 2H), 7.34 – 7.28 (m, 3H), 6.56 (d, *J* = 7.5 Hz, 1H), 3.42 (s, 1H), 3.33 (s, 1H), 3.00 (s, 3H), 2.98 (s, 3H), 1.58 – 1.47 (m, 2H), 1.36 (h, *J* = 7.3 Hz, 2H), 0.91 (t, *J* = 7.3 Hz, 3H). **^{13}C NMR** (101 MHz, $CDCl_3$) δ 177.2, 160.8, 136.7, 129.0, 128.3, 128.0, 77.3, 77.0, 76.7, 58.5, 37.2, 36.1, 30.9, 20.1, 13.8. **HRMS** (ESI+, *m/z*) calculated for $C_{15}H_{23}N_3OSNa^+$ [M+Na]$^+$: 316.1451, found: 316.1454.
SIDA active in toluene-d_8.

(*R*)-2-(3-butylthioureido)-*N*,*N*-dimethyl-2-phenylacetamide ((*R*)-2d)

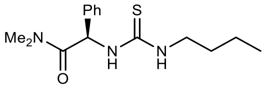

Prepared according to general procedure **C** using α-amino amine salt (*R*)-**S2** (305 mg, 1.412 mmol, 1.00 equiv.), Et_3N (0.296 mL, 2.131 mmol, 1.5 equiv.) and butyl isothiocyanate (0.173 mL, 1.492 mmol, 1.10 equiv.) in DCM (7.1 mL, 0.2 M). Crystallization from Et_2O/DCM afforded product **(*R*)-2d** as a white solid (0.098 mg, 0.334 mmol, 24%). **^{1}H NMR** (400 MHz, $CDCl_3$) δ 7.45 (s, 2H), 7.32 (td, *J* = 4.1, 2.4 Hz, 3H), 6.53 (d, *J* = 7.5 Hz, 1H), 6.18 (s, 1H), 3.33 (s, 2H), 2.98 (s, 3H), 2.98 (s, 3H), 1.61 – 1.49 (m, 2H), 1.44 – 1.29 (m, 2H), 0.91 (t, *J* = 7.3 Hz, 3H). **^{13}C NMR** (101 MHz, $CDCl_3$) δ 177.2, 160.8, 136.7, 129.0, 128.3, 128.0, 77.3, 77.0, 76.7, 58.5, 37.2, 36.1, 30.9, 20.1, 13.8. **HRMS** (ESI+, *m/z*) calculated for $C_{15}H_{23}N_3OSNa^+$ [M+Na]$^+$: 316.1454, found: 316.1454.
SIDA active in toluene-d_8.

Diethyl (*S*)-(2-(dimethylamino)-2-oxo-1-phenylethyl)phosphoramidate ((*S*)-2e)

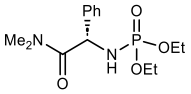

Prepared according to general procedure **C** using α-amino amine salt **(*S*)-S2** (315 mg, 1.467 mmol, 1.00 equiv.), Et_3N (0.296 mL, 2.131 mmol, 1.5 equiv.) and diethyl phosphorochloridate (0.223 mL, 1.541 mmol, 1.05 equiv.) in DCM (7.3 mL, 0.2 M). Purification by column chromatography (hexane/^{i}PrOH 9:1 to 8:2) afforded the product **(*S*)-2e** as a white solid (0.103 mg, 0.327 mmol, 22%). **^{1}H NMR** (400 MHz, $CDCl_3$) 7.41 – 7.26 (m, 5H), 5.13 (t, *J* = 7.9 Hz, 1H), 4.48 (dd, *J* = 12.2, 8.3 Hz, 1H), 3.96 (dp, *J* = 10.1, 7.1 Hz, 1H), 3.86 – 3.67 (m, 3H), 2.97 (s, 3H), 2.86 (s, 3H), 1.15 (dtd, *J* = 20.2, 7.1, 0.9 Hz, 6H). **^{13}C NMR** (101 MHz, $CDCl_3$) δ 170.3 (d, *J* = 9.0 Hz), 139.3, 128.9, 128.1, 127.6, 62.3 (d, *J* = 5.2 Hz), 62.1 (d, *J* = 5.4 Hz), 56.1 (d, *J* = 4.1 Hz), 36.9, 36.2, 16.0 (d, *J* = 1.6 Hz), 15.9. **^{31}P NMR** (162 MHz, $CDCl_3$) δ 6.74. **HRMS** (ESI+, *m/z*) calculated for $C_{14}H_{23}N_2O_4PNa^+$ $[M+Na]^+$: 337.1288, found: 337.1286.
SIDA inactive

Diethyl (*R*)-(2-(dimethylamino)-2-oxo-1-phenylethyl)phosphoramidate ((*R*)-2e)

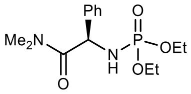

Prepared according to general procedure **C** using α-amino amine salt **(*R*)-S2** (315 mg, 1.467 mmol, 1.00 equiv.), Et_3N (0.296 mL, 2.131 mmol, 1.50 equiv.) and diethyl phosphorochloridate (0.223 mL, 1.541 mmol, 1.05 equiv.) in DCM (7.3 mL, 0.2 M). Purification by column chromatography (hexane/^{i}PrOH 9:1 to 8:2) afforded the product **(*R*)-2e** as a white solid (0.121 mg, 0.385 mmol, 26%). ^{1}H NMR (400 MHz, $CDCl_3$) δ 7.4 – 7.2 (m, 5H), 5.1 (t, *J* = 7.9 Hz, 1H), 4.5 (dd, *J* = 12.2, 8.3 Hz, 1H), 4.0 (dp, *J* = 10.1, 7.1 Hz, 1H), 3.9 – 3.7 (m, 3H), 3.0 (s, 3H), 2.9 (s, 3H), 1.2 (dtd, *J* = 20.3, 7.1, 0.9 Hz, 6H). **C NMR** (151 MHz, $CDCl_3$) δ 170.4 (d, *J* = 9.1 Hz), 139.2 (d, *J* = 2.7 Hz), 128.9, 128.1, 127.6, 65.2 (t, *J* = 3.1 Hz), 62.2 (dd, *J* = 25.1, 5.3 Hz), 56.1 (d, *J* = 4.1 Hz), 36.9, 36.2, 16.0 (d, *J* = 2.6 Hz), 16.0 (d, *J* = 2.4 Hz). **^{31}P NMR** (162 MHz, $CDCl_3$) δ 6.74**.** **HRMS** (ESI+, *m/z*) calculated for $C_{14}H_{23}N_2O_4PNa^+$ $[M+Na]^+$: 337.1288, found: 337.1285.
SIDA inactive

(*S*)- 2-((diphenylphosphoryl)amino)-*N*,*N*-dimethyl-2-phenylacetamide ((*S*)-2f)

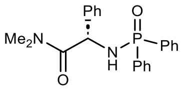

Prepared according to general procedure **C** using α-amino amine salt **(S)-S2** (407 mg, 1.891 mmol, 1.00 equiv.), Et_3N (0.494 mL, 2.837 mmol, 1.50 equiv.) and diphenylphosphonic chloride (0.361 mL, 1.891mmol, 1.00 equiv.) in DCM (9.5 mL, 0.2 M). Product **(*S*)-2f** was obtained as a white solid (0.124 mg, 0.327 mmol, 17%) and was pure enough for further analysis. **^{1}H NMR** (400 MHz, $CDCl_3$) δ 7.81 (dd, *J* = 12.28, 7.33 Hz, 3H), 7.64 (dd, *J* = 12.31, 7.61 Hz, 2H), 7.53 – 7.34 (m, 5H), 7.20 (p, *J* = 4.02 Hz, 5H), 5.19 (dd, *J* = 10.67, 8.36 Hz, 1H), 4.95 (t, *J* = 9.09 Hz, 1H), 2.96 (s, 3H), 2.74 (s, 3H). **^{13}C NMR** (101 MHz, $CDCl_3$) δ 170.7, 132.4, 132.4, 131.9, 131.7, 131.6, 131.5, 131.5, 128.7, 128.5, 128.4, 128.2, 128.0, 127.8, 127.7, 54.5, 36.8, 36.2. **^{31}P NMR** (162 MHz, $CDCl_3$) δ 23.59. **HRMS** (APCl+, *m/z*) calculated for $C_{22}H_{23}N_2O_2PH$ [M+H]: 379.1570, found: 379.1561.
SIDA inactive

(*R*)-2-((diphenylphosphoryl)amino)-*N*,*N*-dimethyl-2-phenylacetamide ((*R*)-2f)

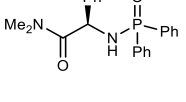

Prepared according to general procedure **C** using α-amino amine salt **(*R*)-S2** (364 mg, 1.695 mmol, 1.00 equiv.), Et_3N (0.443 mL, 2.543 mmol, 1.50 equiv.) and diphenylphosphonic chloride (0.324 mL, 1.695 mmol, 1.00 equiv.) in DCM (8.5 mL, 0.2 M). Product **(*R*)-2f** was obtained as a white solid (0.118 mg, 0.312 mmol, 18%) and was pure enough for further analysis. **^{1}H NMR** (400 MHz, $CDCl_3$) δ 7.81 (dd, *J* = 12.3, 7.3 Hz, 3H), 7.64 (dd, *J* = 12.3, 7.6 Hz, 2H), 7.53 – 7.34 (m, 5H), 7.20 (p, *J* = 4.0 Hz, 5H), 5.19 (dd, *J* = 10.7, 8.4 Hz, 1H), 4.95 (t, *J* = 9.1 Hz, 1H), 2.96 (s, 3H), 2.74 (s, 3H). **^{13}C NMR** (101 MHz, $CDCl_3$) δ 170.7, 132.4, 132.4, 131.9, 131.7, 131.6, 131.5, 131.5, 128.7, 128.5, 128.4, 128.2, 128.0, 127.8, 127.7, 54.5, 36.8, 36.2. **^{31}P NMR** (162 MHz, $CDCl_3$) δ 23.56. **HRMS** (ESI+, *m/z*) calculated for $C_{22}H_{23}N_2O_2PH^+$ $[M+H]^+$: 379.1570, found: 379.1566.
SIDA inactive

(*S*)-*N*-(2-(dimethylamino)-2-oxo-1-phenylethyl)-2,2,2-trifluoroacetamide ((*S*)-2g)

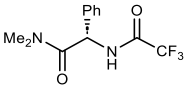

Prepared according to general procedure **C** using α-amino amine salt **(*S*)-S2** (186 mg, 0.870 mmol, 1.00 equiv.), Et_3N (0.241 mL, 1.740 mmol, 2.00 equiv.) and trifluoroacetic anhydride (0.135 mL, 0.957 mmol, 1.10 equiv.) in DCM (5.7 mL, 0.2 M). Purification by column chromatography (hexane/Et_2O 1:1 to 1:2) afforded product **(*S*)-2g** as a pale yellow solid (286 mg, 1.043 mmol, 91%). **^{1}H NMR** (400 MHz, $CDCl_3$) δ 8.15 – 8.08 (m, 1H), 7.44 – 7.31 (m, 5H), 5.73 (d, *J* = 6.9 Hz, 1H), 3.00 (d, *J* = 1.9 Hz, 3H), 2.86 (d, *J* = 1.5 Hz, 3H). **^{13}C NMR** (151 MHz, $CDCl_3$) δ 168.1, 156.0 (q, *J* = 37.6 Hz), 135.4, 129.3, 129.0, 128.0, 115.7 (q, *J* = 287.6 Hz), 54.6, 36.8, 36.1. **^{19}F NMR** (376 MHz, $CDCl_3$) δ -75.90. **HRMS** (ESI+, *m/z*) calculated for $C_{12}H_{13}F_3N_2O_2Na^+$ $[M+Na]^+$: 297.0818, found: 297.0821.
SIDA active in toluene-*d₈*.

(*R*)-*N*-(2-(dimethylamino)-2-oxo-1-phenylethyl)-2,2,2-trifluoroacetamide ((*R*)-2g)

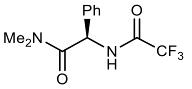

Prepared according to general procedure **C** using α-amino amine salt **(*R*)-S2** (251 mg, 1.170 mmol, 1.00 equiv.), Et_3N (0.325 mL, 2.340 mmol, 2.00 eq.) and trifluoroacetic anhydride (0.182 mL, 1.287 mmol, 1.1 eq.) in DCM (7.7 mL, 0.2 M). Purification by column chromatography (hexane/Et_2O 1:1 to 1:2) afforded product **(*R*)-2g** as a pale yellow solid (297 mg, 1.083 mmol, 93%). **^{1}H NMR** (400 MHz, $CDCl_3$) δ 8.14 (s, 1H), 7.43 – 7.36 (m, 2H), 7.36 – 7.30 (m, 3H), 5.73 (d, *J* = 7.0 Hz, 1H), 2.98 (dd, *J* = 5.6, 2.3 Hz, 3H), 2.85 (dd, *J* = 4.3, 1.9 Hz, 3H). **^{13}C NMR** (151 MHz, $CDCl_3$) δ 168.1, 156.0 (q, *J* = 37.6 Hz), 135.4, 129.3, 129.0, 128.0, 115.7 (q, *J* = 287.6 Hz), 54.6, 36.8, 36.1. **^{19}F NMR** (376 MHz, $CDCl_3$) δ -75.90. **HRMS** (ESI+, *m/z*) calculated for $C_{12}H_{13}F_3N_2O_2Na^+$ $[M+Na]^+$: 297.0818, found: 297.0819.
SIDA active in toluene-*d₈*.

(*S*)-N-(2-(dimethylamino)-2-oxo-1-phenylethyl)-3,5-dinitrobenzamide ((*S*)-2h)

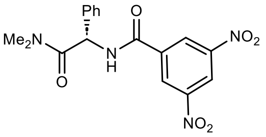

Prepared according to general procedure **C** using α-amino amine salt **(*S*)-S2** (250 mg, 0.870 mmol, 1.00 equiv.), Et_3N (0.244 mL, 1.755 mmol, 1.5 eq.) and 3,5-dinitrobenzoyl chloride (297 mg, 1.287 mmol, 1.10 equiv.) in DCM (7.7 mL, 0.2 M). Purification by column chromatography (hexane/EtOAc 1:9 to 2:8) afforded product **(*S*)-2h** as a pale yellow solid (341 mg, 0.916 mmol, 78 %). **^{1}H NMR** (600 MHz, $CDCl_3$) δ 9.14 (t, *J* = 2.1 Hz, 1H), 8.95 (t, *J* = 2.1 Hz, 2H), 8.57 (d, *J* = 9.8 Hz, 1H), 7.55 – 7.50 (m, 2H), 7.46 – 7.41 (m, 2H), 7.41 – 7.35 (m, 1H), 6.07 (dt, *J* = 5.3, 2.2 Hz, 1H), 3.08 – 3.04 (m, 3H),

2.99 – 2.96 (m, 3H). **^{13}C NMR** (151 MHz, $CDCl_3$) δ 169.3, 161.3, 148.5, 137.4, 136.4, 129.3, 128.8, 128.2, 127.4, 127.4, 121.1, 55.2, 37.0, 36.2. **HRMS** (ESI+, *m/z*) calculated for $C_{17}H_{16}N_4O_6H^+$ $[M+H]^+$: 373.1143 found: 373.1142.
SIDA active in $CDCl_3$

(*R*)-N-(2-(dimethylamino)-2-oxo-1-phenylethyl)-3,5-dinitrobenzamide ((*R*)-2h)

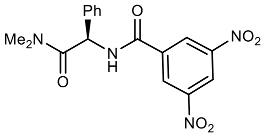

Prepared according to general procedure **C** using α-amino amine salt **(*R*)-S2** (186 mg, 0.870 mmol, 1.00 equiv.), Et_3N (0.183 mL, 1.315 mmol, 1.50 equiv.) and 3,5-dinitrobenzoyl chloride (222 mg, 0.965 mmol, 1.10 equiv.) in DCM (5.7 mL, 0.2 M). Purification by column chromatography (hexane/EtOAc 1:9 to 2:8) afforded product **(*R*)-2h** as a pale yellow solid (275 mg, 0.739 mmol, 84 %). **^{1}H NMR** (400 MHz, $CDCl_3$) δ 9.13 (t, *J* = 2.1 Hz, 1H), 8.93 (d, *J* = 2.1 Hz, 2H), 8.39 (d, *J* = 6.9 Hz, 1H), 7.53 – 7.46 (m, 2H), 7.45 – 7.31 (m, 3H), 6.02 (d, *J* = 7.0 Hz, 1H), 3.04 (s, 3H), 2.94 (s, 3H). **^{13}C NMR** (151 MHz, $CDCl_3$) δ 169.3, 161.3, 148.5, 137.4, 136.4, 129.3, 128.8, 128.2, 127.4, 127.4, 121.1, 55.2, 37.0, 36.2. **HRMS** (ESI+, *m/z*) calculated for $C_{17}H_{16}N_4O_6H^+$ $[M+H]^+$: 373.1143 found: 373.1144.
SIDA active in $CDCl_3$

6.4 Synthesis and characterization of α-amino ester 3a – 3h

General procedure **D** for the synthesis of α-amino esters

To a stirred solution of (*R*)- or (*S*)-phenylglycine methyl ester (1 equiv.) and Et_3N (if applicable) in anhydrous DCM (0.2 M) at 0 ˚C, the electrophile (isocyanate, isothiocyanate, etc.) (1.00 to 1.10 equiv.) was added dropwise, and the mixture was allowed to warm up to room temperature overnight. The reaction mixture was diluted with DCM and washed with aq. HCl (3 M), sat. aq. $NaHCO_3$, and brine (only applicable if Et_3N was used). The organic layer was dried over Na_2SO_4 and all volatiles were removed under reduced pressure. Purification by column chromatography afforded the desired product.

Methyl 2-(3-(3,5-bis(trifluoromethyl)phenyl)ureido)-2-phenylacetate (3aa)

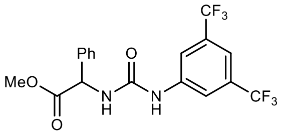

Prepared according to general procedure **D** using scalemic phenylglycine methyl ester (*R/S* = 80:20) (300 mg, 1.816 mmol, 1.00 equiv.) and 3,5-bis(trifluoromethyl)phenyl isocyanate (0.314 mL, 1.816 mmol, 1.00 equiv.) in DCM (9.1 mL, 0.2 M). Product **3aa** (328 mg, 0.780 mmol, 43%) was pure enough for further analysis. **^{1}H NMR** (400 MHz, $CDCl_3$) δ 7.82 – 7.75 (m, 2H), 7.47 (d, *J* = 10.1 Hz, 1H), 7.41 – 7.30 (m, 5H), 7.08 (s, 1H), 5.95 (t, *J* = 6.7 Hz, 1H), 5.57 (d, *J* = 6.7 Hz, 1H), 3.79 (d, *J* = 4.6 Hz, 3H). **^{13}C NMR** (101 MHz, DSMO) δ 171.79, 154.52, 142.33, 137.19, 131.18 (q, *J* = 32.4 Hz), 129.31, 128.84, 127.81, 123.72 (q, *J* = 272.9 Hz), 117.80, 114.51, 57.15, 52.90. **^{19}F NMR** (376 MHz, DMSO) δ -61.8.
SIDA active in $CDCl_3$.

(*S*)-Methyl 2-phenyl-2-(3-(3-(trifluoromethyl)phenyl)ureido)acetate ((*S*)-3ab)

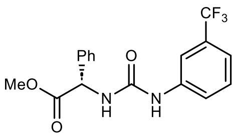

Prepared according to general procedure **D** using (*S*)-phenylglycine methyl ester (189 mg, 1.142 mmol, 1.00 equiv.) and 3-(trifluoromethyl)phenyl isocyanate (0.16 mL, 1.142 mmol, 1.00 equiv.) in DCM (5.7 mL, 0.2 M). Product **(*S*)-3ab** (382mg, 1.084 mmol, 95 %) was pure enough for further analysis. **^{1}H NMR** (400 MHz, $CDCl_3$) δ 7.57 (d, *J* = 12.4 Hz, 2H), 7.39 – 7.18 (m, 8H), 6.48 (d, *J* = 7.0 Hz, 1H), 5.58 (d, *J* = 7.0 Hz, 1H), 3.72 (s, 3H). ^{13}C NMR (101 MHz, $CDCl_3$) δ 172.9, 154.6, 139.0, 136.4, 131.2 (d, *J* = 32.4 Hz), 129.4, 129.1, 128.7, 127.1, 125.2, 122.6, 119.7 (d, *J* = 3.9 Hz), 116.2 (d, *J* = 4.0 Hz), 57.3, 53.0. ^{19}F NMR (376 MHz, $CDCl_3$) δ -62.8. **HRMS** (ESI+, *m/z*) calculated for $C_{17}H_{15}F_3N_2O_3H^+$ $[M+H]^+$: 353.1108, found: 353.1107.
SIDA active in $CDCl_3$

(*R*)-Methyl 2-phenyl-2-(3-(3-(trifluoromethyl)phenyl)ureido)acetate ((*R*)-3ab)

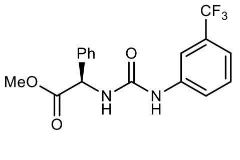

Prepared according to general procedure **D** using (*R*)-phenylglycine methyl ester (189 mg, 1.142 mmol, 1.00 equiv.) and 3-(trifluoromethyl)phenyl isocyanate (0.16 mL, 1.142 mmol, 1.00 equiv.) in DCM (5.7 mL, 0.2 M). Product **(*R*)-3ab** (375 mg, 1.064 mmol, 93 %) was pure enough for further analysis. **^{1}H NMR** (400 MHz, $CDCl_3$) δ 7.58 (d, *J* = 12.5 Hz, 1H), 7.46 – 7.30 (m, 7H), 7.25 – 7.13 (m, 2H), 6.19 (d, *J* = 7.0 Hz, 1H), 5.58 (d, *J* = 7.0 Hz, 1H), 3.75 (s, 3H). **^{13}C NMR** (101 MHz, $CDCl_3$) δ 172.8, 154.7, 138.9, 136.3, 131.3 (d, *J* = 32.4 Hz), 129.5, 129.1, 128.7, 127.1, 125.2, 122.7, 119.8 (d, *J* = 3.9 Hz), 116.4 (d, *J* = 4.0 Hz), 57.3, 53.0. **^{19}F NMR** (376 MHz, $CDCl_3$) δ -62.8. **HRMS** (ESI+, *m/z*) calculated for $C_{17}H_{15}F_3N_2O_3H^+$ $[M+H]^+$: 353.1108, found: 353.1105.
SIDA active in $CDCl_3$

(*S*)-Methyl 2-phenyl-2-(3-(4-(trifluoromethyl)phenyl)ureido)acetate ((*S*)-3ac)

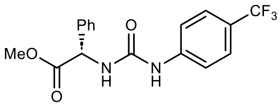

Prepared according to general procedure **D** using (*S*)-phenylglycine methyl ester (189 mg, 1.142 mmol, 1.00 equiv.) and 4-(trifluoromethyl)phenyl isocyanate (0.16 mL, 1.142 mmol, 1.00 equiv.) in DCM (5.7 mL, 0.2 M). Purification by column chromatography (hexane/ EtOAc 8/2) afforded product **(*S*)-3ac** as a white solid (345 mg, 0.979 mmol, 87 %). **^{1}H NMR** (400 MHz, $CDCl_3$) δ 7.47 (d, *J* = 8.5 Hz, 2H), 7.38 (d, *J* = 12.6 Hz, 7H), 6.90 (s, 1H), 6.00 (s, 1H), 5.57 (s, 1H), 3.76 (s, 3H). **^{13}C NMR** (101 MHz, $CDCl_3$) δ 172.8, 154.7, 141.6, 136.2, 129.1, 128.7, 127.1, 126.2 (d, *J* = 3.8 Hz), 125.5, 125.0, 118.9, 77.3, 77.0, 76.7, 57.3, 53.0. **^{19}F NMR** (376 MHz, $CDCl_3$) δ -62.1. **HRMS** (ESI+, *m/z*) calculated for $C_{17}H_{15}F_3N_2O_3H^+$ $[M+H]^+$: 353.1108, found: 353.1104.
SIDA active in $CDCl_3$

(*R*)-Methyl 2-phenyl-2-(3-(4-(trifluoromethyl)phenyl)ureido)acetate ((*R*)-3ac)

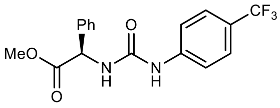

Prepared according to general procedure **D** using (*R*)-phenylglycine methyl ester (189 mg, 1.142 mmol, 1.00 eqiv.) and 4-(trifluoromethyl)phenyl isocyanate (0.16 mL, 1.142 mmol, 1.00 equiv.) in DCM (5.7 mL, 0.2 M). Purification by column chromatography (hexane/ EtOAc 8/2) afforded product **(*R*)-3ac** as a white solid (356 mg, 1.010 mmol, 90 %). **^{1}H NMR** (400 MHz, $CDCl_3$) δ 7.48 (d, *J* = 8.5 Hz, 2H), 7.38 (d, *J* = 15.3 Hz, 7H), 6.79 (s, 1H), 5.93 (d, *J* = 7.0 Hz, 1H), 5.57 (d, *J* = 6.9 Hz, 1H), 3.76 (s, 3H). **^{13}C NMR** (101 MHz, $CDCl_3$) δ 173.0, 154.5, 141.7, 136.2, 129.1, 128.7, 127.1, 126.1 (q, *J* = 3.9

Hz), 125.0, 122.8, 118.8, 57.3, 53.1. **^{19}F NMR** (376 MHz, $CDCl_3$) δ -62.0. **HRMS** (ESI+, *m/z*) calculated for $C_{17}H_{15}F_3N_2O_3H^+$ $[M+H]^+$: 353.1108, found: 353.1105.
SIDA active in $CDCl_3$

Methyl 2-phenyl-2-(3-phenylureido)acetate (3ad)

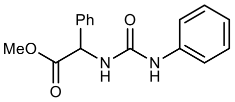

Prepared according to general procedure **D** using scalemic phenylglycine methyl ester (*R/S* = 8/2) (280 mg, 1.695 mmol, 1.00 equiv.) and phenyl isocyanate (0.184 mL, 1.695 mmol, 1.00 equiv.) in DCM (8.5 mL, 0.2 M). Purification by column chromatography (hexane/ EtOAc 7/3) afforded product **3ad** as a white solid (395 mg, 1.389 mmol, 82 %). **^{1}H NMR** (400 MHz, $CDCl_3$) δ 7.41 – 7.28 (m, 9H), 7.09 (dq, *J* = 8.6, 2.8 Hz, 1H), 6.42 (d, *J* = 6.6 Hz, 1H), 5.79 (d, *J* = 6.9 Hz, 1H), 5.58 (d, *J* = 7.1 Hz, 1H), 3.74 (s, 3H). **^{13}C NMR** (101 MHz, DMSO) δ 172.2, 154.7, 140.3, 137.6, 129.3, 129.2, 128.8, 127.7, 121.9, 118.0, 57.1, 52.8. **HRMS** (ESI+, *m/z*) calculated for $C_{16}H_{16}N_2O_3Na^+$ $[M+Na]^+$: 307.1053, found: 307.1051.
SIDA inactive

Methyl 2-(3-(4-methoxyphenyl)ureido)-2-phenylacetate (3ae)

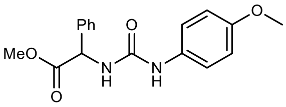

Prepared according to general procedure **D** using scalemic phenylglycine methyl ester (*R/S* = 8/2) (280 mg, 1.695 mmol, 1.00 equiv.) and 4-methoxyphenyl isocyanate (0.220 mL, 1.695 mmol, 1.00 equiv.) in DCM (8.5 mL, 0.2 M). Purification by column chromatography (hexane/ EtOAc 7/3) afforded product **3ae** as a white solid (289 mg, 0.919 mmol, 54 %). **^{1}H NMR** (400 MHz, $CDCl_3$) δ 7.37 – 7.28 (m, 5H), 7.25 – 7.16 (m, 2H), 6.91 – 6.82 (m, 2H), 6.27 (s, 1H), 5.69 (d, *J* = 7.4 Hz, 1H), 5.56 (d, *J* = 7.3 Hz, 1H), 3.79 (s, 3H), 3.72 (s, 3H). **^{13}C NMR** (101 MHz, $CDCl_3$) δ 172.4, 156.8, 155.5, 137.0, 130.8, 128.9, 128.4, 127.2, 124.0, 114.5, 57.1, 55.5, 52.8. **HRMS** (ESI+, *m/z*) calculated for $C_{17}H_{18}N_2O_4Na^+$ $[M+Na]^+$: 337.1159, found: 337.1158.
SIDA inactive

Methyl (*S*)-2-(3-hexylureido)-2-phenylacetate ((*S*)-3b)

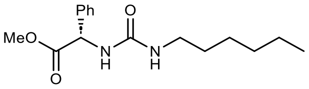

Prepared according to general procedure **D** using (*S*)-phenylglycine methyl ester (700 mg, 4.237 mmol, 1.00 equiv.) and hexyl isocyanate (0.617 mL, 4.327 mmol, 1.00 equiv.) in DCM (18.2 mL, 0.2 M). Purification by column chromatography (hexane/ EtOAc 6/4) afforded product **(*S*)-3b** as a white solid (1010 mg, 3.454 mmol, 82 %). **^{1}H NMR** (400 MHz, $CDCl_3$) δ 7.40 – 7.27 (m, 5H), 5.90 (d, *J* = 6.0 Hz, 1H), 5.49 (d, *J* = 7.3 Hz, 1H), 5.08 (s, 1H), 3.68 (s, 3H), 3.19 – 3.04 (m, 2H), 1.41 (p, *J* = 6.7 Hz, 2H), 1.32 – 1.15 (m, 6H), 0.96 – 0.78 (m, 3H). **^{13}C NMR** (101 MHz, $CDCl_3$) δ 172.5, 156.9, 137.4, 128.9, 128.4, 127.2, 57.2, 52.7, 40.7, 31.5, 30.0, 26.5, 22.6, 14.0.
SIDA active in toluene-*d_8*.

Methyl (*R*)-2-(3-hexylureido)-2-phenylacetate ((*R*)-3b)

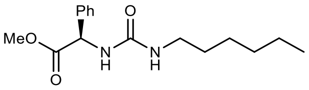

Prepared according to general procedure **D** using (*R*)-phenylglycine methyl ester (600 mg, 3.632 mmol, 1.00 equiv.) and hexyl isocyanate (0.529 mL, 3.632 mmol, 1.00 equiv.) in DCM (18.2 mL, 0.2 M). Purification by column chromatography (hexane/ EtOAc 6/4) afforded product **(*R*)-3b** as a white solid (889 mg, 3.039 mmol, 84 %). **^{1}H NMR** (400 MHz, $CDCl_3$) δ 7.39 – 7.24 (m, 5H), 5.77 (d, *J* = 7.3 Hz, 1H), 5.50 (d, *J* = 7.3 Hz, 1H), 4.93 (t, *J* = 5.6 Hz, 1H), 3.69 (s, 3H), 3.10 (td, *J* = 7.2, 5.6 Hz, 2H), 1.41 (q, *J* = 7.3 Hz, 2H), 1.33 – 1.18 (m, 6H), 0.90 – 0.82 (m, 3H). **^{13}C NMR** (101 MHz, $CDCl_3$) δ 1 172.5, 156.9, 137.4, 128.9, 128.4, 127.2, 57.2, 52.7, 40.7, 31.5, 30.0, 26.5, 22.6, 14.0.
SIDA active in toluene-d_8.

Methyl 2-phenyl-2-(3-phenylthioureido)acetate (3c)

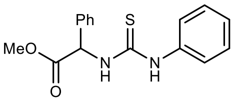

Prepared according to general procedure **D** using racemic phenylglycine methyl ester (*R/S* = 75:25) (386 mg, 2.337 mmol, 1.00 equiv.) and phenyl isothiocyanate (0.280 mL, 2.337 mmol, 1.00 equiv.) in DCM (11.7 mL, 0.2 M). Purification by column chromatography (hexane/ EtOAc 7:3) afforded the product **3c** as a white solid (889 mg, 3.039 mmol, 84 %). **^{1}H NMR** (400 MHz, $CDCl_3$) $^{\delta}$ 8.07 (s, 1H), 7.54 – 7.43 (m, 3H), 7.40 – 7.27 (m, 7H), 7.12 – 7.08 (m, 1H), 6.20 (d, *J* = 6.6 Hz, 1H), 3.75 (s, 3H). **^{13}C NMR** (101 MHz, $CDCl_3$) δ 180.0, 171.4, 135.9, 135.9, 130.3, 129.0, 128.7, 127.4, 124.8, 61.4, 53.0. **HRMS** (ESI+, *m/z*) calculated for $C_{16}H_{17}N_2O_2SH^+$ $[M+H]^+$: 301.1005, found: 301.1002.
SIDA inactive

Methyl 2-((diethoxyphosphoryl)amino)-2-phenylacetate (3e)

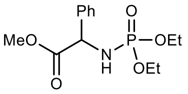

Prepared according to general procedure **D** using racemic phenylglycine methyl ester (*R/S* = 90:10) (403 mg, 2.000 mmol, 1.00 equiv.), diethyl phosphorchloridate (0.347 mL, 2.000 mmol, 1.00 equiv.), and Et_3N (0.612 mL, 4.400 mmol, 2.20 eq.) in DCM (10 mL, 0.2 M). Purification by column chromatography (Et_2O to Et_2O/DCM 8:2) afforded product **3e** as a white solid (537 mg, 1.782 mmol, 89 %). **^{1}H NMR** (600 MHz, $CDCl_3$) δ 7.41 – 7.32 (m, 5H), 4.93 (t, *J* = 8.8 Hz, 1H), 3.99 (m, 4H), 3.83 – 3.76 (m, 1H), 3.73 (d, *J* = 0.9 Hz, 3H), 1.27 (t, *J* = 7.1 Hz, 3H), 1.14 (t, *J* = 7.1 Hz, 3H). **^{13}C NMR** (151 MHz, $CDCl_3$) δ 172.2, 172.1, 138.3, 138.3, 128.8, 128.4, 126.9, 77.2, 77.0, 76.8, 62.6, 62.5, 62.5, 58.0, 57.9, 52.9, 16.1, 16.1, 15.9, 15.9. ^{31}P NMR (243 MHz, $CDCl_3$) δ 6.2. **HRMS** (ESI+, *m/z*) calculated for $C_{13}H_{21}N_1O_5P^+$ $[M+H]^+$: 302.1152, found: 302.1149.
SIDA inactive

Methyl-(*S*)-2-((diphenylphosphoryl)amino)-2-phenylacetate ((*S*)-3f)

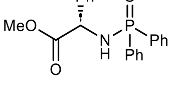

Prepared according to general procedure **D** using (*S*)-phenylglycine methyl ester (350 mg, 2.200 mmol, 1.00 equiv.), diphenylphosphonic chloride (0.404 mL, 2.2 mmol, 1.00 eq.), and Et_3N (0.324 mL, 2.420 mmol, 1.10 equiv.) in DCM (11 mL, 0.2 M). Purification by column chromatography (100 % EtOAc) afforded product **(*S*)-3f** as a white solid (490 mg, 1.341 mmol, 61 %). **^{1}H NMR** (400 MHz, $CDCl_3$) δ

7.91 – 7.82 (m, 2H), 7.77 – 7.70 (m, 3H), 7.56 – 7.49 (m, 1H), 7.49 – 7.39 (m, 4H), 7.39 – 7.27 (m, 5H), 4.95 (t, *J* = 9.6 Hz, 1H), 4.29 (s, 1H), 3.69 (s, 3H). **^{13}C NMR** (101 MHz, $CDCl_3$) δ 172.6, 138.2, 132.4, 132.3, 131.9, 131.8, 128.7, 128.7, 128.5, 128.4, 128.3, 128.2, 127.0, 56.8, 52.9. **^{31}P NMR** (162 MHz, $cdcl_3$) δ 23.70. Data in agreement with literature.[18]
SIDA inactive

Methyl-(*R*)-2-((diphenylphosphoryl)amino)-2-phenylacetate ((*R*)-3f)

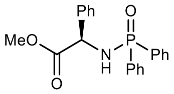

Prepared according to general procedure **D** using (*R*)-phenylglycine methyl ester (300 mg, 1.816 mmol, 1.00 equiv.), diphenylphosphonic chloride (0.347 mL, 1.816 mmol, 1.00 equiv.), and Et_3N (0.348 mL, 1.998 mmol, 1.10 equiv.) in DCM (11 mL, 0.2 M). Purification by column chromatography (100 % EtOAc) afforded product **(*R*)-3f** as a white solid (391 mg, 1.070 mmol, 59 %). **^{1}H NMR** (400 MHz, $CDCl_3$) δ 7.9 (ddt, *J* = 12.1, 7.0, 1.4 Hz, 2H), 7.7 (ddt, *J* = 12.3, 7.1, 1.4 Hz, 2H), 7.6 – 7.5 (m, 1H), 7.4 (tdd, *J* = 7.3, 3.6, 2.3 Hz, 4H), 7.4 – 7.3 (m, 6H), 4.9 (t, *J* = 10.1 Hz, 1H), 4.3 (dd, *J* = 9.5, 6.1 Hz, 1H), 3.7 (s, 3H). **^{13}C NMR** (101 MHz, $CDCl_3$) δ 170.9, 132.4, 132.3, 132.1, 131.9, 131.8, 128.7, 128.7, 128.5, 128.4, 128.3, 128.2, 127.0, 56.8, 52.9. **^{31}P NMR** (162 MHz, $CDCl_3$) δ 23.70. Data in agreement with literature.[18]
SIDA inactive

Methyl (*S*)-2-phenyl-2-(2,2,2-trifluoroacetamido)acetate ((*S*)-3g)

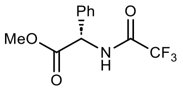

Prepared according to general procedure **D** using (*S*)-phenylglycine methyl ester (300 mg, 1.816 mmol, 1.00 equiv.), trifluoroacetic anhydride (283 mg, 1.816 mmol, 1.0 equiv.), and Et_3N (0.287 mL, 1.998 mmol, 1.10 equiv.) in DCM (11 mL, 0.2 M). Purification by column chromatography (hexanes/EtOAc 8:2) afforded product **(*S*)-3g** as a white solid (210 mg, 0.804 mmol, 44 %). %). **^{1}H NMR** (400 MHz, $CDCl_3$) δ 7.46 – 7.31 (m, 6H), 5.56 (d, *J* = 7.0 Hz, 1H), 3.78 (s, 3H). **^{13}C NMR** (101 MHz, $CDCl_3$) δ 180.3, 170.0, 134.6, 129.3, 129.2, 127.2, 56.5, 53.4. **^{19}F NMR** (376 MHz, $CDCl_3$) δ -75.78. Data in agreement with literature.[19]
SIDA inactive

Methyl (*R*)-2-phenyl-2-(2,2,2-trifluoroacetamido)acetate ((*R*)-3g)

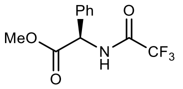

Prepared according to general procedure **D** using (*R*)-phenylglycine methyl ester (300 mg, 1.816 mmol, 1.00 equiv.), trifluoroacetic anhydride (283 mg, 1.816 mmol, 1.00 equiv.), and Et_3N (0.287 mL, 1.998 mmol, 1.10 equiv.) in DCM (11 mL, 0.2 M). Purification by column chromatography (hexanes/EtOAc 8:2) afforded product **(*R*)-3g** as a white solid (163 mg, 0.624 mmol, 34 %). %). **^{1}H NMR** (400 MHz, $CDCl_3$) δ 7.49 – 7.32 (m, 6H), 5.56 (d, *J* = 7.0 Hz, 1H), 3.78 (s, 3H). **^{13}C NMR** (101 MHz, $CDCl_3$)^{13}C NMR (101 MHz, $CDCl_3$) δ 180.3, 170.0, 134.6, 129.3, 129.2, 127.2, 56.5, 53.4. **^{19}F NMR** (376 MHz, $CDCl_3$) δ -75.78. Data in agreement with literature.[19]
SIDA inactive

Methyl (*S*)-2-(3,5-dinitrobenzamido)-2-phenylacetate ((*S*)-3h)

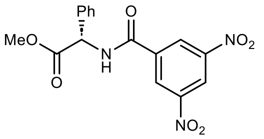

Prepared according to general procedure **D** using (*S*)-phenylglycine methyl ester (300 mg, 1.816 mmol, 1.00 equiv.), 3,5-dinitrobenzoyl chloride (419 mg, 1.816 mmol, 1.00 equiv.), and Et_3N (0.287 mL, 1.998 mmol, 1.10 equiv.) in DCM (11 mL, 0.2 M). Purification by column chromatography (hexanes/EtOAc 7:3) afforded the product **(*S*)-3h** as a white solid (80 mg, 0.223 mmol, 12 %). **^{1}H NMR** (400 MHz, $CDCl_3$) δ 9.38 – 9.25 (m, 1H), 9.19 (t, *J* = 2.1 Hz, 2H), 8.97 (d, *J* = 2.1 Hz, 3H), 7.44 (td, *J* = 5.9, 2.4 Hz, 6H), 7.44 – 7.31 (m, 4H), 7.20 – 7.05 (m, 1H), 5.78 (d, *J* = 6.8 Hz, 2H), 4.23 (s, 1H), 3.82 (s, 5H). **^{13}C NMR** (151 MHz, $CDCl_3$) δ 171.0, 162.0, 148.7, 137.1, 135.5, 129.3, 129.2, 127.5, 127.4, 121.5, 77.2, 77.0, 76.8, 57.3, 53.3. NMR data in agreement with literature.[20]
SIDA active in toluene-*d*8.

Methyl (*R*)-2-(3,5-dinitrobenzamido)-2-phenylacetate ((*R*)-3h)

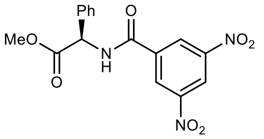

Prepared according to general procedure **D** using (*R*)-phenylglycine methyl ester (300 mg, 1.816 mmol, 1.00 equiv.), 3,5-dinitrobenzoyl chloride (419 mg, 1.816 mmol, 1.00 equiv.), and Et_3N (0.287 mL, 1.998 mmol, 1.10 equiv.) in DCM (11 mL, 0.2 M). Purification by column chromatography (hexanes/EtOAc 7:3) afforded product **(*R*)-3h** as a white solid (163 mg, 0.624 mmol, 34 %). **^{1}H NMR** (400 MHz, $CDCl_3$) δ 9.19 (t, *J* = 2.1 Hz, 1H), 8.97 (d, *J* = 2.1 Hz, 2H), 7.48 – 7.38 (m, 5H), 7.35 (d, *J* = 7.0 Hz, 1H), 5.78 (d, *J* = 6.8 Hz, 1H), 3.82 (s, 3H). **^{13}C NMR** (151 MHz, $CDCl_3$) δ 171.0, 162.0, 148.7, 137.6, 137.1, 135.5, 129.3, 129.2, 127.5, 127.4, 121.5, 88.9, 77.2, 77.0, 76.8, 57.3, 53.3. NMR data in agreement with literature.[20]
SIDA active in toluene-*d*8.

6.5 Synthesis and characterization of 1-phenethylamines 4a – d and 4f – 4h

General procedure **E** for the synthesis of 1-phenethylamines **4a – d** and **4f – 4h**

To a stirred solution of (*R*)- or (*S*)-α-methylbenzylamine (1.00 equiv.) and base (if applicable) in anhydrous DCM (0.2 M) at 0 °C, the electrophile (1.00 to 1.10 equiv.) was added dropwise, and the mixture was allowed to warm to rt overnight. The reaction mixture was diluted with DCM and washed with aq. HCl (3 M), sat. aq. $NaHCO_3$, and brine (only applicable if base was used). The organic layer was dried over Na_2SO_4, and all volatiles were removed under reduced pressure. Purification by column chromatography afforded the desired product.

1-(3,5-bis(trifluoromethyl)phenyl)-3-(1-phenylethyl)urea (*rac*-4a)

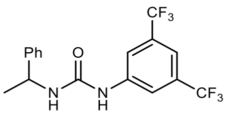

Prepared according to general procedure **E** using (rac)-α-methylbenzylamine (242 mg, 2.000 mmol, 1.00 equiv.) and 3,5-bis(trifluoromethyl)phenyl isocyanate (535 mg, 2.100 mmol, 1.05 equiv.) in DCM (10 mL, 0.2 M). Crystallization from DCM/pentane afforded product **(rac)-4a** as a white solid (731 mg, 1.943 mmol, 97 %). **^{1}H NMR** (400 MHz, $CDCl_3$) δ 7.76 (d, *J* = 1.5 Hz, 2H), 7.47 (s, 1H), 7.42 – 7.26 (m, 5H), 6.56 (s, 1H), 4.94 (q, *J* = 6.9 Hz, 1H), 1.53 (d, *J* = 6.8 Hz, 3H). **^{13}C NMR** (101 MHz, $CDCl_3$)

δ 155.5, 143.5, 140.2, 132.0 (q, J = 33.2 Hz), 128.7, 127.4, 125.3, 124.4, 121.7, 118.4, 115.8, 50.3, 22.8. **^{19}F NMR** (376 MHz, $CDCl_3$) δ -63.26. NMR data in agreement with literature.[21]
SIDA active in toluene-d_8.

(*R*)-1-(3,5-bis(trifluoromethyl)phenyl)-3-(1-phenylethyl)urea ((*R*)-4a)

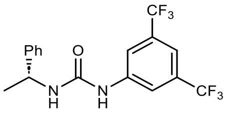

Prepared according to general procedure **E** using (*R*)-α-methylbenzylamine (242 mg, 2.000 mmol, 1.00 equiv.) and 3,5-bis(trifluoromethyl)phenyl isocyanate (535 mg, 2.100 mmol, 1.05 equiv.) in DCM (10 mL, 0.2 M). Crystallization from DCM/pentane afforded product **(*R*)-4a** as a white solid (743 mg, 1.975 mmol, 98 %). **^{1}H NMR** (400 MHz, $CDCl_3$) δ 7.78 – 7.73 (m, 2H), 7.47 (s, 1H), 7.41 – 7.27 (m, 5H), 6.61 (s, 1H), 4.93 (d, J = 6.9 Hz, 1H), 1.52 (d, J = 6.8 Hz, 3H). **^{13}C NMR** (101 MHz, $CDCl_3$) δ 155.5, 143.5, 140.2, 132.0 (q, J = 33.2 Hz), 128.7, 127.4, 125.3, 124.4, 121.7, 118.4, 115.8, 50.3, 22.8. **^{19}F NMR** (376 MHz, $CDCl_3$) δ -63.26. NMR data in agreement with literature.[21]
SIDA active in toluene-d_8.

(*S*)-1-hexyl-3-(1-phenylethyl)urea ((*S*)-4b)

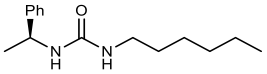

Prepared according to general procedure **E** using (*S*)-α-methylbenzylamine (242 mg, 2.000 mmol, 1.00 equiv.) and hexyl isocyanate (0.306 mL, 2.100 mmol, 1.05 equiv.) in DCM (10 mL, 0.2 M). Purification by column chromatography (hexane/ EtOAc 7:3 to 1:1) afforded product **(*S*)-4b** as a white solid (470 mg, 1.892 mmol, 95 %). **^{1}H NMR** (400 MHz, $CDCl_3$) 7.35 – 7.17 (m, 5H), 5.20 (s, 1H), 4.77 (q, J = 6.6 Hz, 1H), 4.11 (q, J = 7.1 Hz, 1H), 3.07 (tdd, J = 16.0, 11.9, 6.5 Hz, 2H), 2.03 (s, 1H), 1.39 (tdd, J = 9.9, 7.9, 4.8 Hz, 4H), 1.27 – 1.17 (m, 6H), 0.85 (t, J = 6.9 Hz, 3H). **^{13}C NMR** (101 MHz, $CDCl_3$) 157.6, 144.1, 128.8, 127.4, 125.9, 50.5, 40.5, 31.5, 30.0, 26.4, 23.5, 22.5, 14.0. **HRMS** (ESI+, *m/z*) calculated for $C_{15}H_{25}N_2O^+$ $[M+H]^+$: 249.1961, found: 249.1960.
SIDA inactive

(*R*)-1-hexyl-3-(1-phenylethyl)urea ((*R*)-4b)

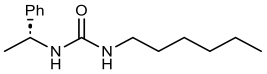

Prepared according to general procedure **E** using (*R*)-α-methylbenzylamine (242 mg, 2.000 mmol, 1.00 equiv.) and hexyl isocyanate (0.306 mL, 2.100 mmol, 1.05 equiv.) in DCM (10 mL, 0.2 M). Purification by column chromatography (hexane/ EtOAc 7:3 to 1:1) afforded product **(*R*)-4b** as a white solid (482 mg, 1.941 mmol, 97 %). **^{1}H NMR** (600 MHz, $CDCl_3$) δ 7.37 (d, J = 6.2 Hz, 4H), 7.30 (s, 1H), 4.77 (p, J = 7.7 Hz, 1H), 4.59 – 4.55 (m, 1H), 4.17 (s, 1H), 3.13 (dh, J = 26.5, 6.6 Hz, 2H), 1.51 – 1.46 (m, 3H), 1.40 (p, J = 7.6 Hz, 2H), 1.24 (ddq, J = 28.9, 15.1, 7.6 Hz, 6H), 0.88 (dq, J = 6.8, 2.4 Hz, 3H). ^{13}**C NMR** (151 MHz, $CDCl_3$) δ 157.6, 144.1, 128.8, 127.4, 125.9, 50.6, 40.5, 31.5, 30.0, 26.4, 23.5, 22.5, 14.0. **HRMS** (ESI+, *m/z*) calculated for $C_{15}H_{25}N_2O^+$ $[M+H]^+$: 249.1961, found: 249.1962.
SIDA inactive

(*S*)-1-(3,5-bis(trifluoromethyl)phenyl)-3-(1-phenylethyl)thiourea ((*S*)-4c)

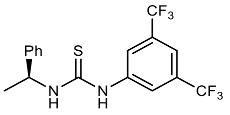

Prepared according to general procedure **E** using (*S*)-α-methylbenzylamine (242 mg, 2.000 mmol, 1.00 equiv.) and 3,5-bis(trifluoromethyl)phenyl isothiocyanate (0.383 mL, 2.100 mmol, 1.05 equiv.) in DCM (10 mL, 0.2 M). Purification by column chromatography (hexane/ EtOAc 3:2) afforded product **(*S*)-4c** as a white solid (741 mg, 1.889 mmol, 94 %). **^{1}H NMR** (600 MHz, $CDCl_3$) δ 8.23 (s, 1H), 7.68 (s, 3H), 7.45 – 7.31 (m, 5H), 6.63 (s, 1H), 5.46 (s, 1H), 1.62 (d, *J* = 6.9 Hz, 3H). **^{19}F NMR** (565 MHz, $CDCl_3$) δ -63.13. **HRMS** (ESI+, *m/z*) calculated for $C_{17}H_{25}F_6N_2S^+$ $[M+H]^+$: 393.0855, found: 393.0851. NMR data in agreement with literature.[22]
SIDA inactive

(*R*)-1-(3,5-bis(trifluoromethyl)phenyl)-3-(1-phenylethyl)thiourea ((*R*)-4c)

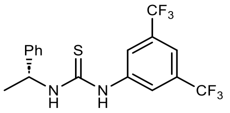

Prepared according to general procedure **E** using (*R*)-α-methylbenzylamine (242 mg, 2.000 mmol, 1.00 equiv.) and 3,5-bis(trifluoromethyl)phenyl isothiocyanate (0.383 mL, 2.100 mmol, 1.05 equiv.) in DCM (10 mL, 0.2 M). Purification by column chromatography (hexane/ EtOAc 3:2) afforded product **(*R*)-4c** as a white solid (775 mg, 1.975 mmol, 99 %). **^{1}H NMR** (600 MHz, $CDCl_3$) δ 7.81 (s, 1H), 7.68 (d, *J* = 3.7 Hz, 3H), 7.50 – 7.32 (m, 5H), 6.57 (s, 1H), 5.42 (s, 1H), 1.63 (d, *J* = 6.9 Hz, 3H). **^{19}F NMR** (565 MHz, $CDCl_3$) δ -63.11. **HRMS** (ESI+, *m/z*) calculated for $C_{17}H_{25}F_6N_2S^+$ $[M+H]^+$: 393.0855, found: 393.0852. NMR data in agreement with literature.[22]
SIDA inactive

(*S*)-1-Butyl-3-(1-phenylethyl)thiourea ((*S*)-4d)

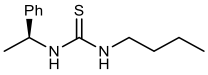

Prepared according to general procedure **E** using (*S*)-α-methylbenzylamine (242 mg, 2.000 mmol, 1.00 equiv.) and butyl isothiocyanate (0.243 mL, 2.100 mmol, 1.05 equiv.) in DCM (10 mL, 0.2 M). Purification by column chromatography (hexane/ EtOAc 6:4) afforded product **(*S*)-4d** as an orange oil (0.437 mg, 1.852 mmol, 92 %). **^{1}H NMR** (600 MHz, $CDCl_3$) δ 7.44 – 7.30 (m, 5H), 6.21 (s, 1H), 5.43 (s, 1H), 4.82 (s, 1H), 3.46 (s, 1H), 3.41 – 3.22 (m, 1H), 1.54 (s, 3H), 1.39 (q, *J* = 8.3 Hz, 2H), 1.13 (s, 2H), 0.83 (t, *J* = 7.3 Hz, 3H). **^{13}C NMR** (151 MHz, $CDCl_3$) δ 181.0, 142.0, 129.3, 128.1, 125.8, 60.4, 54.0, 30.8, 19.7, 13.6. **HRMS** (ESI+, *m/z*) calculated for $C_{13}H_{20}N_2SH^+$ $[M+H]^+$: 237.1420, found: 237.1414.
SIDA inactive

(*R*)-1-Butyl-3-(1-phenylethyl)thiourea ((*R*)-4d)

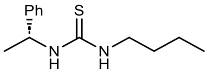

Prepared according to general procedure **E** using (*R*)-α-methylbenzylamine (242 mg, 2.000 mmol, 1.00 equiv.) and butyl isothiocyanate (0.243 mL, 2.100 mmol, 1.05 equiv.) in DCM (10 mL, 0.2 M). Purification by column chromatography (hexane/ EtOAc 6:4) afforded product **(*R*)-4d** as an orange oil (0.429 mg, 1.815 mmol, 91 %). **^{1}H NMR** (600 MHz, $CDCl_3$) δ 7.42 – 7.30 (m, 5H), 6.26 (s, 1H), 5.46 (s, 1H), 4.84 (s, 1H), 3.45 (s, 1H), 3.37 (s, 1H), 1.57 – 1.51 (m, 3H), 1.39 (dt, *J* = 15.3, 7.9 Hz, 2H), 1.18

– 1.06 (m, 2H), 0.83 (t, *J* = 7.4 Hz, 3H). **^{13}C NMR** (151 MHz, $CDCl_3$) δ 180.9, 142.0, 129.2, 128.1, 125.8, 53.9, 30.8, 19.7, 13.6. **HRMS** (ESI+, *m/z*) calculated for $C_{13}H_{20}N_2SH^+$ $[M+H]^+$: 237.1420, found: 237.1419.
SIDA inactive

Diisopropyl (*S*)-(1-phenylethyl)phosphoramidate ((*S*)-4e)

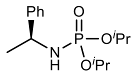

Prepared according to general procedure **F** from (*S*)-(-)-1-phenylethylamine (7.60 mL, 58.7 mmol, 1.50 equiv.) and diisopropyl phosphonate (6.50 mL, 39.1 mmol, 1.00 equiv.). Product **(*S*)-4e** was obtained as a colorless oil (9.35 g, 32.8 mmol, 84%). Product **(*S*)-4e** was obtained as a colorless oil (8.95 g, 31.4 mmol, 80%). **^{1}H NMR** (400 MHz, $CDCl_3$) δ 7.33 – 7.26 (m, 4H), 7.25 – 7.18 (m, 1H), 4.55 (dq, J = 7.4, 6.1 Hz, 1H), 4.45 (dq, J = 7.4, 6.1 Hz, 1H), 4.37 – 4.24 (m, 1H), 3.00 (t, J = 9.6 Hz, 1H), 1.45 (d, J = 6.8 Hz, 3H), 1.30 (d, J = 6.2 Hz, 3H), 1.27 (d, J = 6.2 Hz, 3H), 1.23 (d, J = 6.1 Hz, 3H), 1.02 (d, J = 6.2 Hz, 3H); **^{13}C NMR** (151 MHz, $CDCl_3$) δ 145.2 (d, J = 5.3 Hz), 128.3, 126.8, 125.7, 70.6 (t, J = 5.2 Hz), 51.3, 25.3 (d, J = 5.9 Hz), 23.7 (d, J = 5.4 Hz), 23.7 (d, J = 4.5 Hz), 23.6 (d, J = 4.2 Hz), 23.3 (d, J = 5.6 Hz); **^{31}P NMR** (162 MHz, $CDCl_3$) δ 2.53. Data in accordance with literature.[2]
SIDA active in toluene-*d₈*.

Diisopropyl (R)-(1-phenylethyl)phosphoramidate ((*R*)-4e)

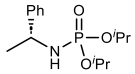

Prepared according to general procedure **F** from (*R*)-(-)-1-phenylethylamine (7.60 mL, 58.7 mmol, 1.50 equiv.) and diisopropyl phosphonate (6.50 mL, 39.1 mmol, 1.00 equiv.). Product **(*R*)-4e** was obtained as a colorless oil (8.95 g, 31.4 mmol, 80%). **^{1}H NMR** (400 MHz, $CDCl_3$) δ 7.34 – 7.27 (m, 4H), 7.26 – 7.18 (m, 1H), 4.57 (dp, J = 7.6, 6.2 Hz, 1H), 4.46 (dp, J = 7.4, 6.1 Hz, 1H), 4.37 – 4.26 (m, 1H), 2.93 (t, J = 9.6 Hz, 1H), 1.46 (d, J = 6.8, 0.8 Hz, 3H), 1.31 (d, J = 6.2 Hz, 3H), 1.28 (d, J = 6.2 Hz, 3H), 1.24 (d, J = 6.2 Hz, 3H), 1.03 (d, J = 6.2 Hz, 3H); **^{13}C NMR** (101 MHz, $CDCl_3$) δ 145.4 (d, J = 5.1 Hz), 128.5, 127.1, 125.9, 70.9 – 70.8 (m), 51.5, 25.6 (d, J = 5.9 Hz), 24.0 (d, J = 4.0 Hz), 23.9, 23.9 (d, J = 6.5 Hz), 23.6 (d, J = 5.7 Hz); **^{31}P NMR** (162 MHz, $CDCl_3$) δ 2.50. Data in accordance with literature.[2]
SIDA active in toluene-*d₈*.

***P,P*-diphenyl-*N*-(1-phenylethyl)phosphinic amide (4f)**

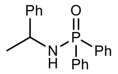

Prepared according to general procedure **E** using α-methylbenzylamine (909 mg, 7.500 mmol, 1.00 equiv.) diphenylphosphonic chloride (1.5 mL, 8.000 mmol, 1.05 eq.), and DIPEA (1.5 mL, 8.750 mmol, 1.15 equiv.) in DCM (20 mL, 0.125 M). Purification by column chromatography (100% EtoAc) afforded product **4f** as white solid (1.986 g, 6.124 mmol, 82 %). **^{1}H NMR** (400 MHz, $CDCl_3$) δ 7.86 (dddt, *J* = 34.2, 12.0, 6.8, 1.5 Hz, 4H), 7.54 – 7.19 (m, 11H), 4.39 (tq, *J* = 9.5, 6.7 Hz, 1H), 3.20 (d, *J* = 7.9 Hz, 1H), 1.57 (d, *J* = 6.8 Hz, 3H). **^{31}P NMR** (162 MHz, $CDCl_3$) δ 22.52. **^{13}C NMR** (151 MHz, DMSO) δ 146.5 (d, *J* = 5.5 Hz), 135.0, 134.7, 134.2, 133.9, 132.3, 132.3, 132.2, 132.2, 131.9 (d, *J* = 2.6 Hz), 131.8 (d, *J* = 2.6 Hz), 128.9, 128.8, 128.8, 128.7, 128.5, 126.8, 126.5, 50.7, 26.4 (d, *J* = 4.3 Hz). **HRMS** (ESI+, *m/z*) calculated for $C_{20}H_{21}NOP^+$ $[M+H]^+$: 322.1355, found: 322.1353.

Data in accordance with literature.[23]
SIDA inactive

(*S*)-2,2,2-trifluoro-*N*-(1-phenylethyl)acetamide ((*S*)-4g)

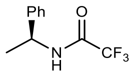

Prepared according to general procedure E using (*S*)-α-methylbenzylamine (242 mg, 2.000 mmol, 1.00 equiv.), trifluoroacetic anhydride (0.353 mL, 2.500 mmol, 1.25 equiv.), and pyridine (0.402 mL, 5.000 mmol, 2.5 eq.) in DCM (3.6 mL, 0.55 M). Product **(*S*)-4g** was obtained as a white solid (428 mg, 1.971 mmol, 98 %). **^{1}H NMR** (600 MHz, $CDCl_3$) δ 7.45 – 7.39 (m, 2H), 7.36 (td, *J* = 6.8, 1.6 Hz, 3H), 6.44 (s, 1H), 5.18 (p, *J* = 7.1 Hz, 1H), 1.62 (d, *J* = 6.9 Hz, 3H). **^{13}C NMR** (101 MHz, $CDCl_3$) δ 150.16, 140.80, 128.22, 126.17, 49.77, 21.02. **^{19}F NMR** (565 MHz, $CDCl_3$) δ -75.88. NMR data is in agreement with literature.[24]
SIDA inactive

(*R*)-2,2,2-trifluoro-*N*-(1-phenylethyl)acetamide ((*R*)-4g)

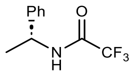

Prepared according to general procedure **E** using (*R*)-α-methylbenzylamine (242 mg, 2.000 mmol, 1.00 equiv.), trifluoroacetic anhydride (0.353 mL, 2.500 mmol, 1.25 equiv.), and pyridine (0.402 mL, 5.000 mmol, 2.5 eq.) in DCM (3.6 mL, 0.55 M). Product **(*R*)-4g** was obtained as a white solid (424 mg, 1.952 mmol, 98 %). **^{1}H NMR** (600 MHz, $CDCl_3$) δ 7.41 (tq, *J* = 7.8, 1.5 Hz, 2H), 7.35 (td, *J* = 7.1, 1.1 Hz, 3H), 6.47 (s, 1H), 5.18 (p, *J* = 7.1 Hz, 1H), 1.62 (d, *J* = 6.9 Hz, 3H). **^{13}C NMR** (101 MHz, $CDCl_3$) δ 161.1, 140.8, 136.8, 129.0, 128.2, 126.2, 49.8, 21.0. **^{19}F NMR** (565 MHz, $CDCl_3$) δ -75.90. NMR data is in agreement with literature.[24]
SIDA inactive

(*S*)-3,5-dinitro-*N*-(1-phenylethyl)benzamide ((*S*)-4h)

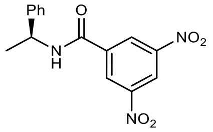

Prepared according to general procedure **E** using (*S*)-α-methylbenzylamine (242 mg, 2.000 mmol, 1.00 equiv.), 3,5-dinitrobenzoyl chloride (461 mg, 2.000 mmol, 1.0 equiv.), and Et_3N (0.313 mL, 2.250 mmol, 1.13 equiv.) in DCM (10 mL, 0.2 M). Crystallization from $CHCl_3$/hexane afforded product **(*S*)-4h** as a white solid (512 mg, 1.624 mmol, 81 %). **^{1}H NMR** (400 MHz, $CDCl_3$) δ 9.12 (t, *J* = 2.1 Hz, 1H), 8.93 (d, *J* = 2.0 Hz, 2H), 7.37 (m, 5H), 6.81 (d, *J* = 7.7 Hz, 1H), 5.32 (p, *J* = 7.1 Hz, 1H), 1.66 (d, *J* = 6.9 Hz, 3H). **^{13}C NMR** (101 MHz, $CDCl_3$) δ 161.9, 148.6, 141.9, 137.8, 128.9, 128.0, 127.2, 126.3, 121.1, 50.3, 21.4. Data in agreement with literature.[25]
SIDA active in $CDCl_3$

3,5-dinitro-N-(1-phenylethyl)benzamide (*rac*-4h)

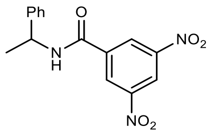

Prepared according to general procedure **E** using *rac*-α-methylbenzylamine (242 mg, 2.000 mmol, 1.00 equiv.), 3,5-dinitrobenzoyl chloride (461 mg, 2.000 mmol, 1.0 equiv.), and Et_3N (0.313 mL, 2.250 mmol, 1.13 equiv.) in DCM (10 mL, 0.2 M). Crystallization from $CHCl_3$/hexane afforded product ***rac*-4h** as a white solid (527 mg, 1.672 mmol, 84 %). **^{1}H NMR** (400 MHz, $CDCl_3$) δ 9.15 – 9.10 (m, 1H), 8.96 – 8.91 (m, 2H), 7.35 – 7.24 (m, 3H), 6.76 (d, *J* = 8.1 Hz, 1H), 5.33 (p, *J* = 7.2 Hz, 1H), 1.66 (d, *J* = 6.9 Hz, 3H). **^{13}C NMR** (101 MHz, DMSO) δ 161.8, 148.6, 144.5, 137.3, 128.8, 128.1, 127.3, 126.6, 121.3, 49.7, 22.4. Data in agreement with literature.[25]
SIDA active in $CDCl_3$

6.6 Synthesis and characterization of 11-15, S3

(*S*)-*N*-benzhydryl-2-(3-(3,5-bis(trifluoromethyl)phenyl)thioureido)-N,3,3-trimethylbutanamide ((*S*)-11)

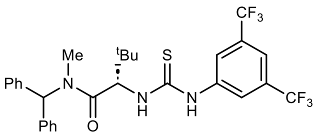

A 100-mL round-bottomed flask containing (*S*)-2-amino-N-benzhydryl-N,3,3-trimethylbutanamide (620 mg, 2.000 mmol, 1.00 equiv.) was charged with anhydrous DCM (6.3 mL, 0.32 M). 3,5-bis(trifluoromethyl)phenyl isothiocyanate (542 mg, 2.000 mmol, 1.00 equiv.) was added in one portion via syringe. The flask was capped with a plastic stopper, and the reaction mixture was stirred for 2 h at rt. Purification by column chromatography (hexanes to hexanes/ Et_2O 1:1) afforded product **(*S*)-11** as a white solid (1087 mg, 1.869 mmol, 93 %). **^{1}H NMR** (600 MHz, $CDCl_3$) δ 8.33 (s, 1H), 8.09 (d, *J* = 9.1 Hz, 1H), 7.67 (s, 2H), 7.58 (s, 1H), 7.38 – 7.29 (m, 3H), 7.16 – 7.12 (m, 2H), 7.04 – 7.00 (m, 3H), 6.92 (dt, *J* = 13.7, 7.1 Hz, 3H), 5.68 (d, *J* = 9.2 Hz, 1H), 3.12 (s, 3H), 1.15 (s, 9H). **^{13}C NMR** (151 MHz, $CDCl_3$) δ 182.3, 174.1, 139.6, 138.8, 137.2, 131.6 (q, *J* = 33.6 Hz), 129.6, 128.7, 128.3, 128.0, 127.3, 127.1, 126.1, 123.0 (q, *J* = 273.0 Hz), 119.1, 62.3, 36.4, 33.9, 27.4. **^{19}F NMR** (565 MHz, $CDCl_3$) δ -62.88. **HRMS** (ESI+, *m/z*) calculated for $C_{29}H_{29}F_9N_3OSNa^+$ $[M+Na]^+$: 605.1861, found: 605.1861.
Data in accordance with literature. [26]
SIDA active in $CDCl_3$

(*R*)-*N*-benzhydryl-2-(3-(3,5-bis(trifluoromethyl)phenyl)thioureido)-N,3,3-trimethylbutanamide ((*R*)-11)

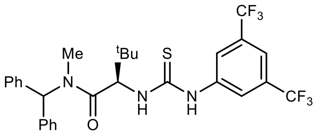

A 100-mL round-bottomed flask containing (*R*)-2-amino-N-benzhydryl-N,3,3-trimethylbutanamide (376 mg, 1.210 mmol, 1.00 equiv.) was charged with anhydrous DCM (3.8 mL, 0.32 M). 3,5-bis(trifluoromethyl)phenyl isothiocyanate (328 mg, 1.210 mmol, 1.00 equiv.) was added in one portion via syringe. The flask was capped with a plastic stopper, and the reaction mixture was stirred for 2 h at rt. Purification by column chromatography (hexanes to hexanes/ Et_2O 1:1) afforded product **(*R*)-11** as a white solid (664 mg, 1.142 mmol, 94 %). **^{13}C NMR** (151 MHz, $CDCl_3$) δ 182.31, 174.07, 139.59, 138.84, 137.18, 131.79 (t, *J* = 33.8 Hz), 129.64, 129.43, 128.65, 128.31, 128.05, 127.34, 127.09, 126.13, 122.97 (q, *J* = 272.8 Hz), 119.12, 62.27, 62.11, 36.36, 33.88, 27.41. **^{13}C NMR** (151 MHz, $CDCl_3$) δ 182.3, 174.1, 139.6, 138.8, 137.2, 131.8 (t, *J* = 33.8 Hz), 129.6, 129.4, 128.6, 128.3, 128.0, 127.3, 127.1, 126.1, 123.0 (q, *J* = 272.8 Hz), 119.1, 62.3, 62.1, 36.4, 33.9, 27.4. **^{19}F NMR** (565 MHz, $CDCl_3$) δ -

62.88. **HRMS** (ESI+, *m/z*) calculated for $C_{29}H_{29}F_9N_3OSNa^+$ $[M+Na]^+$: 605.1861, found: 605.1859. Data in accordance with literature. [26]
SIDA active in $CDCl_3$

Methyl (3,5-dinitrobenzoyl)-*D*-alaninate ((*D)*-12)

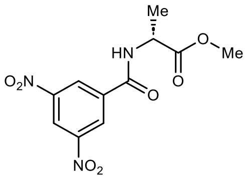

Prepared according to a literature procedure.[4] The alanine methyl ester hydrochloride (614 mg, 4.400 mmol, 1.10 equiv.) was suspended in anhydrous DCM and Et_3N (1.1 mL, 8.000 mmol, 2.00 equiv.) was added. The reaction mixture was cooled to 0 °C. Subsequently, 3,5-dinitrobenzoyl chloride (922 mg, 4.000 mmol, 1.00 equiv.) was added in small portions and the reaction mixture was stirred overnight during which it was slowly allowed to reach room temperature. After diluting with DCM, the mixture was washed with hydrochloric acid (3 M), $NaHCO_3$ solution (saturated aqueous) and brine. The organic layer was dried over Na_2SO_4 and all volatiles were removed under reduced pressure. Product **(*D*)-12** was obtained as a white solid (952 mg, 3.203 mmol, 81 %). **^{1}H NMR** (400 MHz, $CDCl_3$) δ 9.17 (t, *J* = 2.1 Hz, 1H), 8.96 (d, *J* = 2.1 Hz, 2H), 7.18 (d, *J* = 7.2 Hz, 1H), 4.83 (m, 1H), 3.84 (s, 3H), 1.59 (d, 3H). **^{13}C NMR** (151 MHz, $CDCl_3$) δ 173.3, 162.1, 148.7, 137.2, 127.3, 121.3, 53.0, 49.1, 18.4. Data in agreement with literature.[4]
SIDA active in $CDCl_3$

Methyl (3,5-dinitrobenzoyl)-*L*-alaninate ((*L*)-12

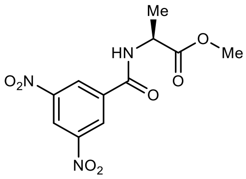

Prepared according to a literature procedure.[4] The alanine methyl ester hydrochloride (614 mg, 4.400 mmol, 1.10 equiv.) was suspended in anhydrous DCM and Et_3N (1.1 mL, 8.000 mmol, 2.00 eqiv.) was added. The reaction mixture was cooled to 0 °C. Subsequently, 3,5-dinitrobenzoyl chloride (922 mg, 4.000 mmol, 1.00 equiv.) was added in small portions and the reaction mixture was stirred overnight during which it was slowly allowed to reach room temperature. After diluting with DCM, the mixture was washed with aq. HCl (3 M), $NaHCO_3$ solution (saturated aqueous) and brine. The organic layer was dried over Na_2SO_4 and all volatiles were removed under reduced pressure. Product **(*L*)-12** was obtained as a white solid (952 mg, 3.166 mmol, 80 %). **^{1}H NMR** (400 MHz, $CDCl_3$) δ 9.19 (t, *J* = 2.1 Hz, 1H), 8.97 (d, *J* = 2.1 Hz, 2H), 7.00 (d, *J* = 7.1 Hz, 1H), 4.89 – 4.78 (m, 1H), 3.84 (s, 3H), 1.60 (d, *J* = 7.1 Hz, 3H). **^{13}C NMR** (151 MHz, $CDCl_3$) δ 173.3, 162.2, 148.7, 137.4, 121.3, 53.0, 49.1, 18.4. Data in agreement with literature.[4]
SIDA active in $CDCl_3$

Isopropyl (*S*)-5-oxopyrrolidine-2-carboxylate ((*S*)-13)

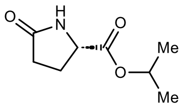

Prepared according to a literature procedure.[5] To a solution of *D*-Pyroglutamic acid (1.00 g, 7.745 mmol 1.00 equiv.) in ^{i}PrOH (3.3 mL) was added $SOCl_2$ (0.67 mL, 9.294 mmol, 1.20 equiv.) dropwise at 0 °C, then the solution was stirred for 3 days at room temperature. The volatile residuals were evaporated, and the remaining oil was dissolved in EtOAc and washed with aq. $NaHCO_3$. The aq. phase was washed with EtOAc and the combined organic phase was washed with brine (2 × 5 mL), dried over Na_2SO_4, filtered and concentrated in vacuo to give product **(*S*)-13** as a white solid (1.02 g, 5.958 mmol, 77 %). **^{1}H NMR** (400 MHz, $CDCl_3$) δ 5.83 (s, 1H), 5.07 (p, *J* = 6.2 Hz, 1H), 4.20 (dd, *J* = 8.5, 5.3 Hz, 1H), 2.55 – 2.42 (m, 1H), 2.36 (dt, *J* = 16.7, 8.3 Hz, 2H), 2.28 – 2.15 (m, 1H), 1.27 (d, *J* = 6.3 Hz, 6H). **^{13}C NMR** (101 MHz, $CDCl_3$) δ 177.5, 171.4, 69.4, 55.4, 29.2, 24.8, 21.7. Data in agreement with literature.[27] SIDA active in toluene-*d*$_8$.

Isopropyl (*R*)-5-oxopyrrolidine-2-carboxylate ((*R*)-13)

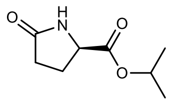

Prepared according to a literature procedure.[5] To a solution of *L*-Pyroglutamic acid (1.00 g, 7.745 mmol 1.00 equiv.) in ^{i}PrOH (3.3 mL) was added $SOCl_2$ (0.67 mL, 9.294 mmol, 1.20 equiv.) dropwise at 0 °C, then the solution was stirred for 3 days at room temperature. The volatile residuals were evaporated, and the remaining oil was dissolved in EtOAc and washed with $NaHCO_3$. The aq. phase was washed with EtOAc and the combined organic phase was washed with brine (2 × 5 mL), dried over Na_2SO_4, filtered and concentrated in vacuo to give product **(*R*)-13** as a white solid (1.09 g, 6.367 mmol, 82 %). **^{1}H NMR** (400 MHz, $CDCl_3$) δ 5.80 (s, 1H), 5.07 (p, *J* = 6.2 Hz, 1H), 4.20 (dd, *J* = 8.5, 5.3 Hz, 1H), 2.55 – 2.41 (m, 1H), 2.37 (ddd, *J* = 9.4, 7.9, 5.9 Hz, 2H), 2.28 – 2.15 (m, 1H), 1.27 (d, *J* = 6.3 Hz, 6H). **^{13}C NMR** (101 MHz, $CDCl_3$) δ 171.4, 156.0, 69.4, 55.4, 29.2, 24.8, 21.7. Data in agreement with literature.[27] SIDA active in toluene-*d*$_8$.

1-(3,5-bis(trifluoromethyl)phenyl)-3-((1*S*,2*S*)-2-(dimethylamino)cyclohexyl)thiourea ((*S*)-14)

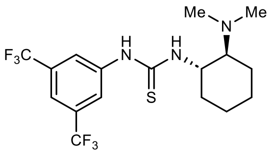

To a stirred solution of (1*S*,2*S*)-N1,N1-dimethylcyclohexane-1,2-diamine (284 mg, 2.000 mmol, 1.00 equiv.) in DCM (5 mL, 0.4 M), was added 3,5-bis(trifluoromethyl)phenyl isothiocyanate (542 mg, 2.000 mmol, 1.00 equiv.) at room temperature. After stirring at room temperature overnight, the mixture was concentrated in vacuo. The residue was purified by column chromatography on silica gel (DCM:MeOH 95:5 to 90:10) to afford product **(*S*)-14** a white foam (670 mg, 1.612 mmol, 81 %). **^{1}H NMR** (600 MHz, $CDCl_3$) δ 7.93 (s, 2H), 7.63 (s, 1H), 7.29 (s, 2H), 4.14 (s, 1H), 2.65 (s, 1H), 2.42 (s, 7H), 1.97 (d, *J* = 12.4 Hz, 1H), 1.90 (d, *J* = 13.1 Hz, 1H), 1.78 (d, *J* = 13.1 Hz, 1H), 1.50 – 1.09 (m, 4H). **^{13}C NMR** (151 MHz, $CDCl_3$) δ 179.9, 140.0 ,132.1, 125.8, 123.9, 123.0, 122.1, 120.3, 118.1, 67.1, 55.9, 40.0, 32.7, 24.6, 24.4, 21.9. **^{19}F NMR** (565 MHz, $CDCl_3$) δ -63.01. Data in agreement with literature.[28] SIDA active in toluene-*d*$_8$.

1-(3,5-bis(trifluoromethyl)phenyl)-3-((1*R*,2*R*)-2-(dimethylamino)cyclohexyl)thiourea ((*R*)-14)

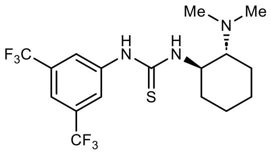

To a stirred solution of (1*R*,2*R*)-N1,N1 -dimethylcyclohexane-1,2-diamine (284 mg, 2.000 mmol, 1.00 equiv.) in DCM (5 mL, 0.4 M), was added 3,5-bis(trifluoromethyl)phenyl isothiocyanate (542 mg, 2.000 mmol, 1.00 equiv.) at room temperature. After stirring at room temperature overnight, the mixture was concentrated in vacuo. The residue was purified by column chromatography on silica gel (DCM:MeOH 95:5 to 90:10) to afford product **(*R*)-14** as a white foam (780 mg, 1.887 mmol, 94 %). ^{1}**H NMR** (400 MHz, $CDCl_3$) δ 7.82 (s, 2H), 7.62 (s, 1H), 3.72 (s, 1H), 2.44 (td, *J* = 11.0, 3.7 Hz, 1H), 2.40 – 2.17 (m, 6H), 1.93 (d, *J* = 12.0 Hz, 1H), 1.85 (d, *J* = 11.9 Hz, 1H), 1.76 (d, *J* = 12.0 Hz, 1H), 1.24 (dtd, *J* = 24.9, 12.7, 9.4 Hz, 4H). ^{13}**C NMR** (151 MHz, $CDCl_3$) δ 140.0, 132.5, 125.7, 123.9, 123.3, 122.1, 120.3, 118.3, 66.8, 56.5, 40.1, 32.9, 24.8, 24.5, 21.5. ^{19}**F NMR** (376 MHz, $CDCl_3$) δ -63.07. Data in agreement with literature.[28]
SIDA active in toluene-*d*$_8$.

Diethyl ((2-fluorophenyl)((5-methylbenzo[d]thiazol-2-yl)amino)methyl)phosphonate (15)

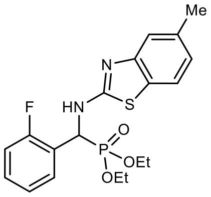

Prepared according to literature[6] using the corresponding imine (405 mg, 1.500 mmol, 1.00 equiv.), diethyl phosphite (0.290 mL, 2.250 mmol, 1.50 equiv.), K_2CO_3 (311 mg, 2.250 mmol, 1.50 equiv.), and quinine (10 mol %) in DCM (4.5 mL, 0.33 M). Crystallization from Et_2O/ hexanes afforded product **15** as yellow solid (489 mg, 1.197 mmol, 80 %). For the racemic version, the corresponding imine (200 mg, 0.740 mmol, 1.00 equiv.), Et_3N (0.15 mL, 1.100 mmol, 1.50 equiv.) and diethyl phosphite (0.14 mL, 1.100 mmol, 1.50 equiv.) were dissolved in DCM (2.2 mL, 0.33 M) and stirred for 3 days. Crystallization from Et_2O/ hexanes afforded product **15** as yellow solid (217 mg, 0.531 mmol, 72 %) ^{1}**H NMR** (600 MHz, $CDCl_3$) δ 7.61 (tt, *J* = 7.6, 2.0 Hz, 1H), 7.45 (d, *J* = 8.2 Hz, 1H), 7.36 (s, 1H), 7.34 – 7.29 (m, 1H), 7.18 – 7.06 (m, 3H), 6.73 (d, *J* = 8.3 Hz, 1H), 5.81 (dd, *J* = 22.6, 8.7 Hz, 1H), 4.31 – 4.18 (m, 2H), 4.03 (dp, *J* = 10.1, 7.2 Hz, 1H), 3.85 (ddq, *J* = 10.1, 8.5, 7.1 Hz, 1H), 2.38 (s, 3H), 1.33 (t, *J* = 7.1 Hz, 3H), 1.15 (t, *J* = 7.1 Hz, 3H). ^{13}**C NMR** (101 MHz, $CDCl_3$) δ 164.9 (d, *J* = 12.1 Hz), 149.9, 131.6, 131.2, 129.8 (d, *J* = 2.8 Hz), 129.8 (d, *J* = 2.9 Hz), 129.3 (t, *J* = 3.7 Hz), 126.9, 124.4 (t, *J* = 3.1 Hz), 123.0 (d, *J* = 14.1 Hz), 120.7, 119.1, 115.4 (dd, *J* = 21.8, 2.2 Hz), 77.3, 77.0, 76.7, 63.6 (d, *J* = 7.1 Hz), 50.1 (d, *J* = 3.2 Hz), 48.5, 21.2, 16.4 (d, *J* = 5.9 Hz), 16.1 (d, *J* = 5.8 Hz). ^{31}**P NMR** (162 MHz, DMSO) δ 20.05. ^{31}**P NMR** (162 MHz, toluene-d$_8$, racemic **15**) δ 19.85, ^{19}**F NMR** (565 MHz, $CDCl_3$) δ -116.70. Data in agreement with literature.[6]
SIDA active in toluene-*d*$_8$.

Diethyl 2-((2-fluorophenyl)((5-methylbenzo[I]thiazol-2-yl)amino)methyl)malonate (S3)

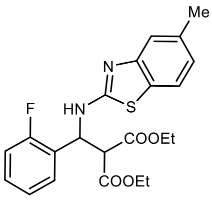

Prepared according to literature[8] using the corresponding imine (405 mg, 1.500 mmol, 1.00 equiv.), diethyl malonate (288 mg, 1.800 mmol, 1.20 equiv.), and quinine (10 mol %) in DCM (15 mL, 0.1 M). Crystallization from Et_2O/ hexanes afforded product **S3** as a yellow solid (525 mg, 1.120 mmol, 81 %). **^{1}H NMR** (600 MHz, $CDCl_3$) δ 7.48 – 7.41 (m, 2H), 7.39 – 7.36 (m, 1H), 7.28 (s, 1H), 7.13 – 7.06 (m, 3H), 6.82 (d, *J* = 9.8 Hz, 1H), 5.91 (dd, *J* = 9.8, 5.3 Hz, 1H), 4.25 – 4.07 (m, 5H), 2.39 (s, 3H), 1.17 (dt, *J* = 20.1, 7.1 Hz, 6H). **^{13}C NMR** (101 MHz, $CDCl_3$) δ 168.0, 166.6, 165.2, 161.5, 159.1, 150.0, 131.6, 130.9, 129.8 (d, *J* = 8.4 Hz), 128.6 (d, *J* = 3.7 Hz), 127.0, 124.3 (d, *J* = 3.4 Hz), 120.8, 119.0, 115.6 (d, *J* = 21.5 Hz), 62.2, 61.8, 55.5 (d, *J* = 2.5 Hz), 53.4 (d, *J* = 1.8 Hz), 21.2, 13.9, 13.9. **^{19}F NMR** (376 MHz, toluene-*d₈*.) δ -117.92. Data in agreement with literature.[8]
SIDA inactive

6.7 Synthesis and characterization of α-amino phosphonates 16, S4-S7

General procedure **F** for the synthesis of α-amino phosphonates

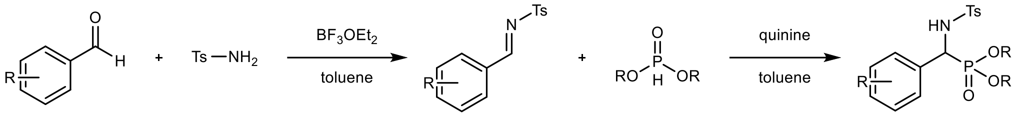

The imine was synthesized following a literature procedure.[29] To a stirred solution of p-toluenesulfonamide (1.00 equiv.) in toluene (0.2 M), the corresponding aldehyde (1.00 equiv.) and catalytic amount of BF_3OEt_2 (10 mol %) were added. The mixture was heated to reflux and stirred for 12 h. Purification by crystallization afforded the desired imine. The desired α-amino phosphonate was synthesized using a literature procedure.[30] To a stirred solution of the imine (1.00 equiv.) in toluene (0.1 M), the corresponding dialkyl or diaryl phosphite (2.00 equiv.) and catalytic amounts of quinine (10 mol %) were added. The mixture was stirred for 24 - 72 h and purification by crystallization or column chromatography afforded the desired product.

Diethyl (((4-methylphenyl)sulfonamido)(phenyl)methyl)phosphonate (16)

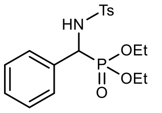

Prepared according to general procedure **F** using the corresponding imine (778 mg, 3.000 mmol, 1.00 equiv.), diethyl phosphite (0.773 mL, 6.000 mmol, 2.00 equiv.), and quinine (10 mol %) in toluene (30 mL, 0.1 M). Crystallization from toluene/ hexanes afforded product **16** as a white solid (1.02 g, 2.566 mmol, 86 %). **^{1}H NMR** (400 MHz, $CDCl_3$) δ 7.50 – 7.43 (m, 2H), 7.19 – 7.08 (m, 5H), 7.02 (dd, *J* = 7.8, 5.1 Hz, 2H), 5.85 (s, 1H), 4.74 (d, *J* = 23.8 Hz, 1H), 4.22 – 4.06 (m, 2H), 3.86 (dp, *J* = 10.3, 7.2 Hz, 1H), 3.67 – 3.52 (m, 1H), 2.30 (s, 3H), 1.31 (td, *J* = 7.1, 3.2 Hz, 3H), 1.03 (td, *J* = 7.1, 1.6 Hz, 3H). **^{13}C NMR** (101 MHz, $CDCl_3$) δ 142.7, 137.8, 133.6, 129.0 (d, *J* = 5.8 Hz), 128.2, 128.2 (d, *J* = 1.6 Hz), 128.1 (d, *J* = 2.2 Hz), 127.7 (d, *J* = 3.0 Hz), 127.0 (d, *J* = 1.8 Hz), 64.1 (d, *J* = 7.1 Hz), 63.6 (d, *J* = 7.4 Hz), 56.0 (d, *J* = 8.1 Hz), 54.5 (d, *J* = 7.4 Hz), 21.3, 16.4 (d, *J* = 5.9 Hz), 16.0 (d, *J* = 5.5 Hz). **^{31}P NMR** (162 MHz, toluene-*d₈*.) δ 20.14, 20.12. Data in agreement with literature.[31]
SIDA active in toluene-*d₈*.

Diethyl ((4-methoxyphenyl)((4-methylphenyl)sulfonamido)methyl)phosphonate (S4)

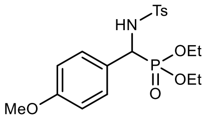

Prepared according to general procedure **F** using the corresponding imine (579 mg, 2.000 mmol, 1.00 equiv.), diethyl phosphite (0.515 mL, 4.000 mmol, 2.00 equiv.), and quinine (10 mol %) in xylene (20 mL, 0.1 M). Crystallization from toluene/ hexanes afforded product **S4** as a white solid (689 mg, 1.612 mmol, 81 %). **^{1}H NMR** (400 MHz, $CDCl_3$) δ 7.46 (dd, *J* = 8.1, 6.1 Hz, 2H), 7.15 – 7.07 (m, 2H), 6.98 (dd, *J* = 12.6, 8.0 Hz, 2H), 6.60 (t, *J* = 8.6 Hz, 2H), 4.72 (dd, *J* = 23.4, 6.2 Hz, 1H), 4.29 – 4.20 (m, 1H), 4.20 – 4.10 (m, 1H), 3.87 (dp, *J* = 10.2, 7.2 Hz, 1H), 3.72 (d, *J* = 2.2 Hz, 3H), 3.69 – 3.54 (m, 1H), 2.27 (s, 3H), 1.34 (t, *J* = 7.2 Hz, 3H), 1.05 (q, *J* = 7.1 Hz, 3H). **^{13}C NMR** (101 MHz, $CDCl_3$) δ 159.3 (d, *J* = 2.7 Hz), 142.6, 138.0 (d, *J* = 1.8 Hz), 129.5, 128.9, 127.1 (d, *J* = 2.4 Hz), 125.6, 113.5 (d, *J* = 2.2 Hz), 64.0 (d, *J* = 7.2 Hz), 63.5 (d, *J* = 7.0 Hz), 55.2, 21.3, 21.3, 16.4 (d, *J* = 6.1 Hz), 16.1 (d, *J* = 5.6 Hz). **^{31}P NMR** (162 MHz, $CDCl_3$) δ 19.78, 19.76. Data in agreement with literature.[32]
SIDA active in toluene-*d*$_8$.

Diethyl (((4-methylphenyl)sulfonamido)(4-(trifluoromethyl)phenyl)methyl)phosphonate (S5)

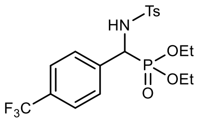

Prepared according to general procedure **F** using the corresponding imine (655 mg, 2.000 mmol, 1.00 equiv.), diethyl phosphite (0.515 mL, 4.000 mmol, 2.00 equiv.), and quinine (10 mol %) in toluene (20 mL, 0.1 M). Crystallization from toluene/ hexanes afforded product **S5** as a white solid (674 mg, 1.448 mmol, 72 %). **^{1}H NMR** (400 MHz, $CDCl_3$) δ 7.44 – 7.38 (m, 2H), 7.34 (d, *J* = 8.2 Hz, 2H), 7.25 (s, 2H), 6.99 (d, *J* = 8.0 Hz, 2H), 4.81 (d, *J* = 24.2 Hz, 1H), 4.27 – 4.11 (m, 2H), 3.94 (dp, *J* = 10.2, 7.2 Hz, 1H), 3.73 (ddt, *J* = 15.8, 10.1, 7.1 Hz, 1H), 2.28 (s, 3H), 1.34 (t, *J* = 7.1 Hz, 3H), 1.09 (t, *J* = 7.1 Hz, 3H). **^{13}C NMR** (101 MHz, $CDCl_3$) δ 143.2, 137.6 (d, *J* = 14.0 Hz), 129.0, 128.6 (d, *J* = 5.8 Hz), 127.0, 124.9, 64.6 (d, *J* = 6.8 Hz), 63.6 (d, *J* = 7.1 Hz), 55.8, 54.3, 21.1, 16.4 (d, *J* = 5.9 Hz), 16.1 (d, *J* = 5.6 Hz). **^{31}P NMR** (162 MHz, $CDCl_3$) δ 18.56. **^{19}F NMR** (376 MHz, $CDCl_3$) δ -62.80. **HRMS** (ESI+, *m/z*) calculated for $C_{19}H_{23}F_3NO_5PSNa^+$ $[M+Na]^+$: 488.0879, found: 488.0874.
SIDA inactive

Diisopropyl (((4-methylphenyl)sulfonamido)(phenyl)methyl)phosphonate (S6)

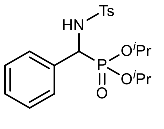

Prepared according to general procedure **F** using the corresponding imine (400 mg, 2.542 mmol, 1.00 eq.), diisopropyl phosphite (0.518 mL, 3.085 mmol, 2.00 eq.), and quinine (20 mol %) in toluene (15.4 mL, 0.1 M). Crystallization from toluene/ hexanes afforded product **S6** as a white solid (399 mg, 0.938 mmol, 61 %). **^{1}H NMR** (400 MHz, $CDCl_3$) δ 7.42 (s, 2H), 7.17 – 7.06 (m, 5H), 7.00 (d, *J* = 7.9 Hz, 2H), 5.57 (t, *J* = 7.9 Hz, 1H), 4.78 – 4.71 (m, 1H), 4.71 – 4.61 (m, 1H), 4.35 (dq, *J* = 12.6, 6.3 Hz, 1H), 2.29 (s, 3H), 1.31 (dd, *J* = 9.5, 6.2 Hz, 6H), 1.19 (d, *J* = 6.2 Hz, 3H), 0.80 (d, *J* = 6.2 Hz, 3H). **^{13}C NMR** (101 MHz, $CDCl_3$) δ 142.5, 138.1, 133.9, 128.8, 128.5 (d, *J* = 6.0 Hz), 127.7 (dd, *J* = 42.3, 2.6 Hz), 127.0, 77.4, 77.0, 76.7, 72.8 (d, *J* = 7.1 Hz), 72.5 (d, *J* = 7.2 Hz), 56.7, 55.1, 24.3 (d, *J* = 3.3 Hz), 24.1 (d, *J* = 3.1 Hz), 23.8 (d, *J* = 5.6 Hz), 22.8 (d, *J* = 6.1 Hz), 21.3. **^{31}P NMR** (162 MHz, $CDCl_3$) δ 17.95, 17.93. Data in agreement with lietarture.[31]
SIDA active in $CDCl_3$.

Diphenyl (((4-methylphenyl)sulfonamido)(phenyl)methyl)phosphonate (S7)

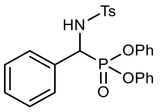

Prepared according to general procedure **F** using the corresponding imine (400 mg, 2.542 mmol, 1.00 eq.), diphenyl phosphite (0.591 mL, 3.085 mmol, 2.00 eq.), and quinine (20 mol %) in toluene (15.4 mL, 0.1 M). Crystallization from toluene/ hexanes afforded the desired product **S7** as a white solid (369 mg, 0.748 mmol, 49 %). **^{1}H NMR** (400 MHz, $CDCl_3$) δ 7.47 (dd, *J* = 8.3, 3.9 Hz, 2H), 7.33 – 7.28 (m, 2H), 7.17 (s, 11H), 6.97 (s, 2H), 6.77 – 6.70 (m, 2H), 5.96 (dd, *J* = 9.6, 5.7 Hz, 1H), 5.24 – 5.11 (m, 1H), 2.27 (s, 3H). **^{13}C NMR** (101 MHz, $CDCl_3$) δ 143.2, 132.5, 129.7, 129.5, 129.2, 128.5, 127.0, 126.4, 125.5, 125.3, 120.7, 120.2, 55.1 21.3. **^{31}P NMR** (162 MHz, $CDCl_3$) δ 12.13, 12.08. Data in agreement with literature.[31]
SIDA active in $CDCl_3$.

7. *e.r.* determination by NMR and/or HPLC

^{1}H-NMR (600 MHz, $CDCl_3$) of **2a** (*e.r.* 10:90, *S/R*)

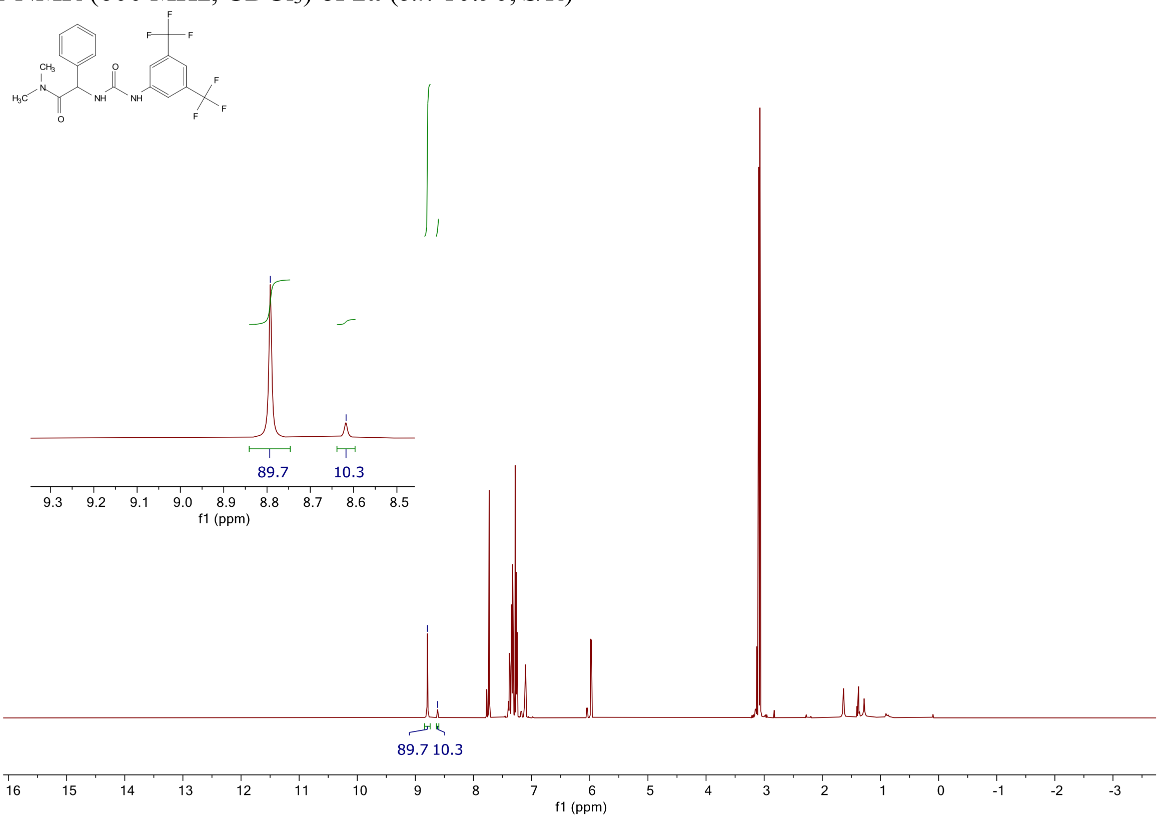

^{19}F-NMR (376 MHz, $CDCl_3$) of **2a** (*e.r.* 10:90)

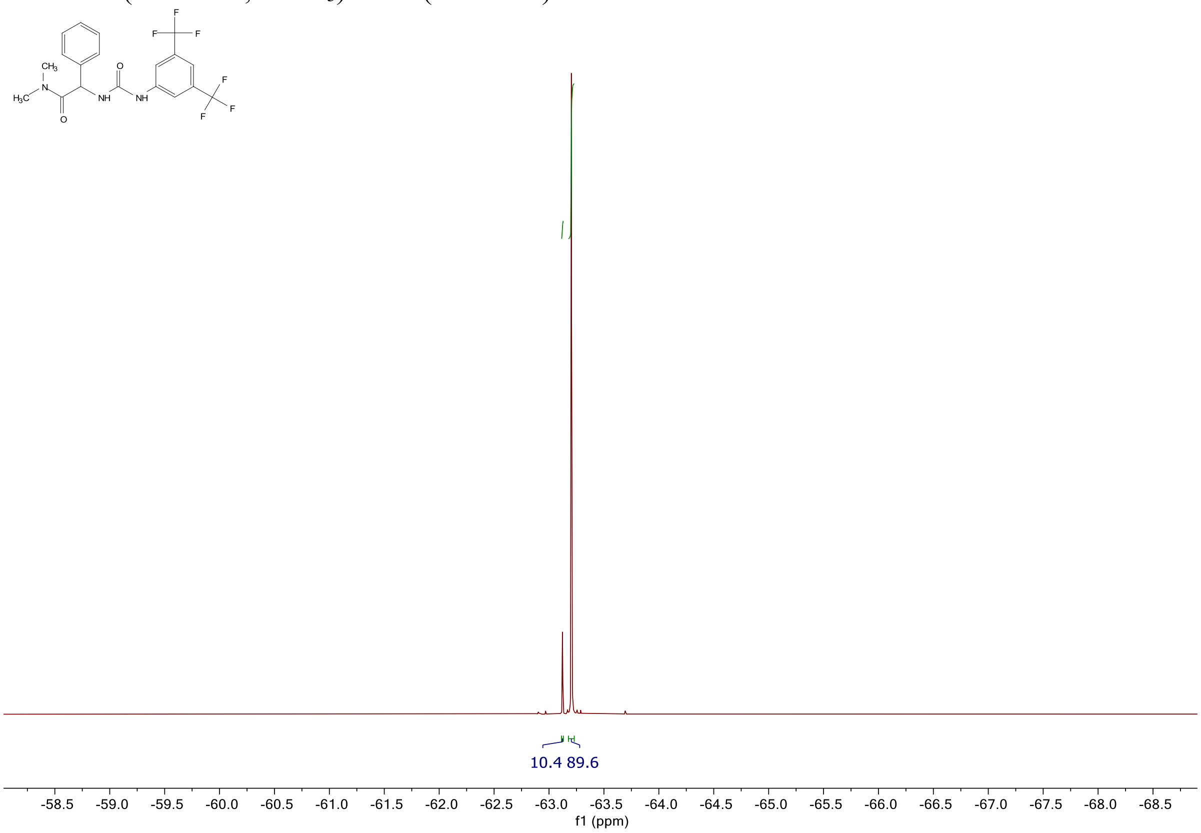

HPLC chromatogram of scalemic **2a** (*e.r.* 10:90)

Conditions: Chiralcel OX-H Lux Cellulose-4, *n*-heptane/^{i}PrOH 85-15, 40 ºC, 1 mL/min, λ_{abs} = 235 nm

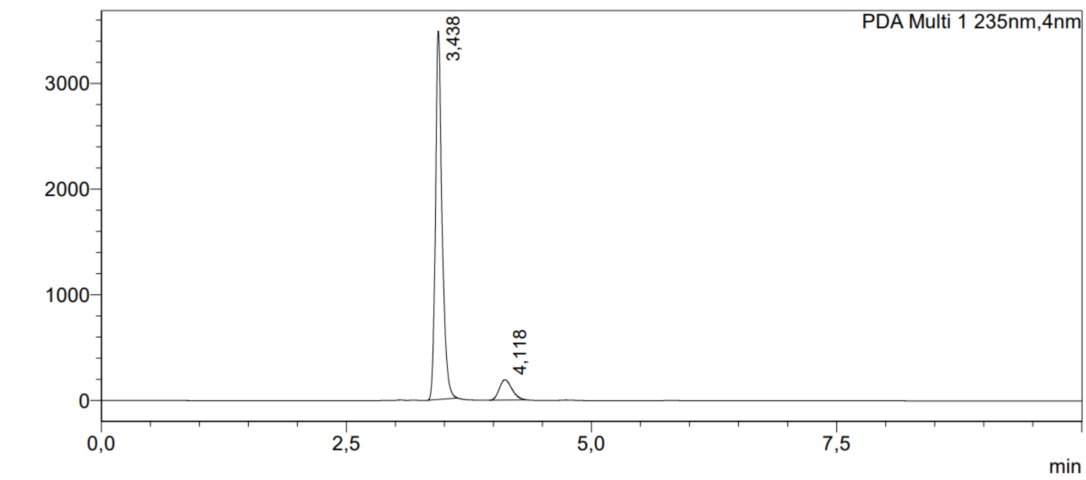

<Peak Table>

PDA Ch1 235nm

Peak#	Ret. Time	Area	Area%
1	3,438	16317355	90,412
2	4,118	1730501	9,588
Total		18047856	100,000

^{1}H-NMR (600 MHz, $CDCl_3$) of **2a** (*e.r.* 20:80)

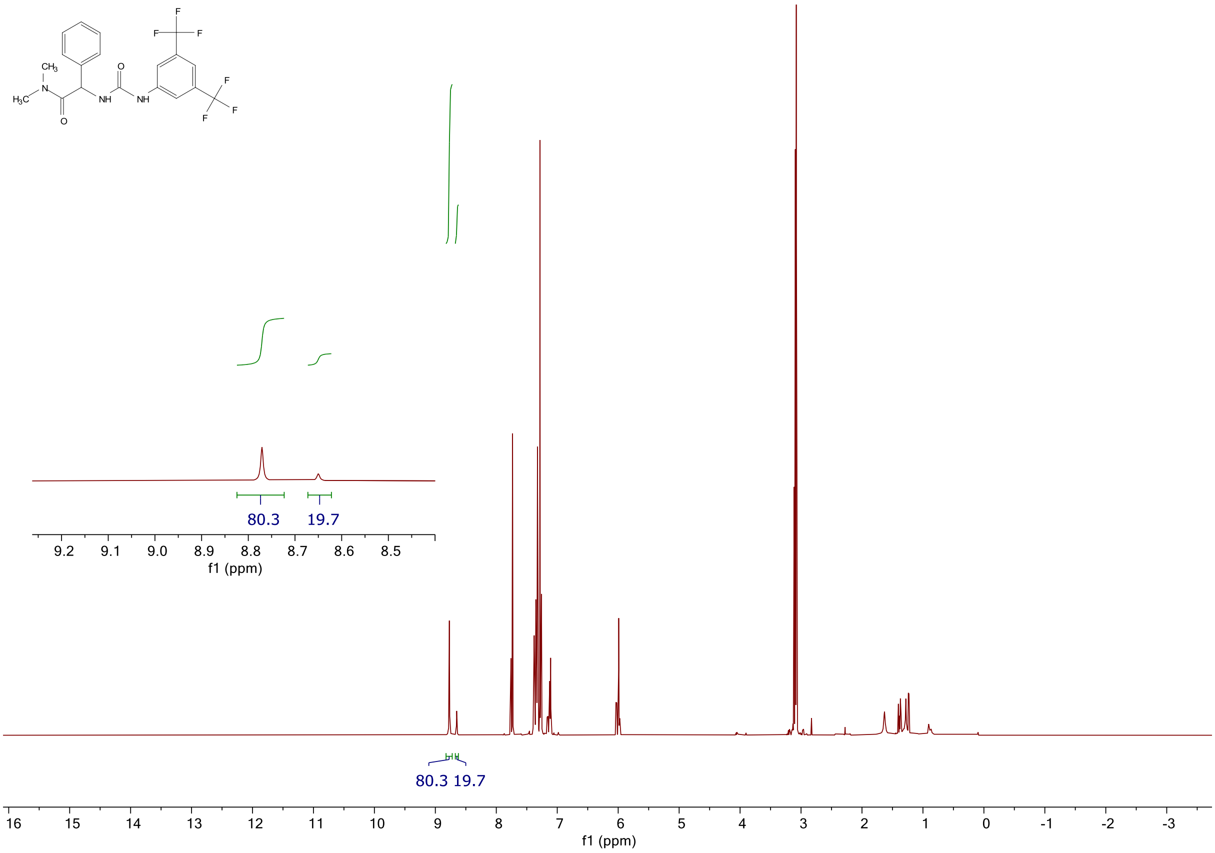

^{19}F-NMR (376 MHz, $CDCl_3$) of **2a** (*e.r.* 20:80)

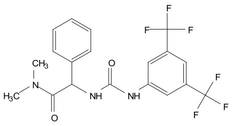

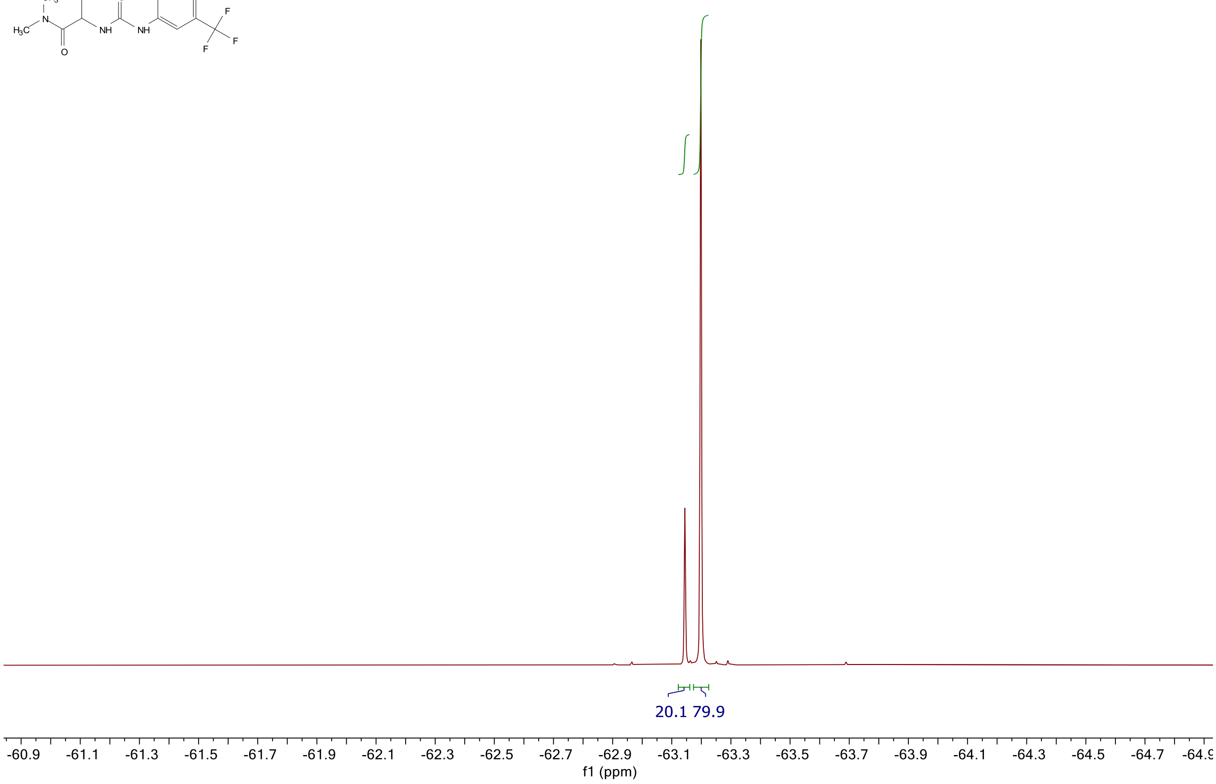

HPLC chromatogram of scalemic **2a** (*e.r.* 20:80)

Conditions: Chiralcel OX-H Lux Cellulose-4, *n*-heptane/^{i}PrOH 85-15, 40 ºC, 1 mL/min, λ_{abs} = 225 nm

<Chromatogram>

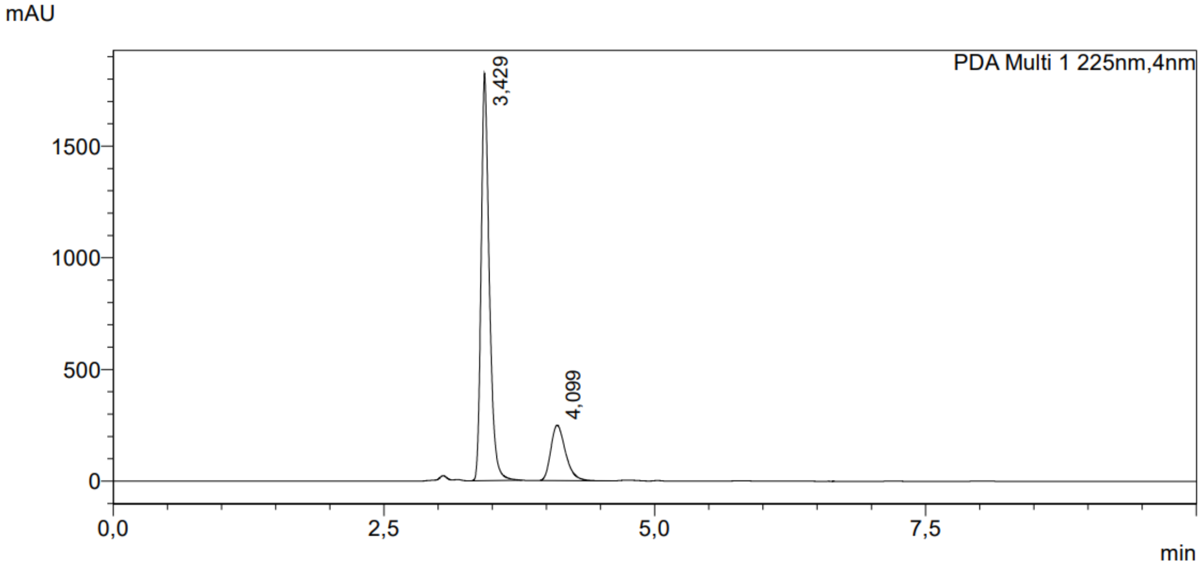

<Peak Table>

PDA Ch1 225nm

Peak#	Ret. Time	Area	Area%
1	3,429	9552072	80,359
2	4,099	2334651	19,641
Total		11886723	100,000

^{1}H-NMR (600 MHz, $CDCl_3$) of **2a** (*e.r.* 30:70)

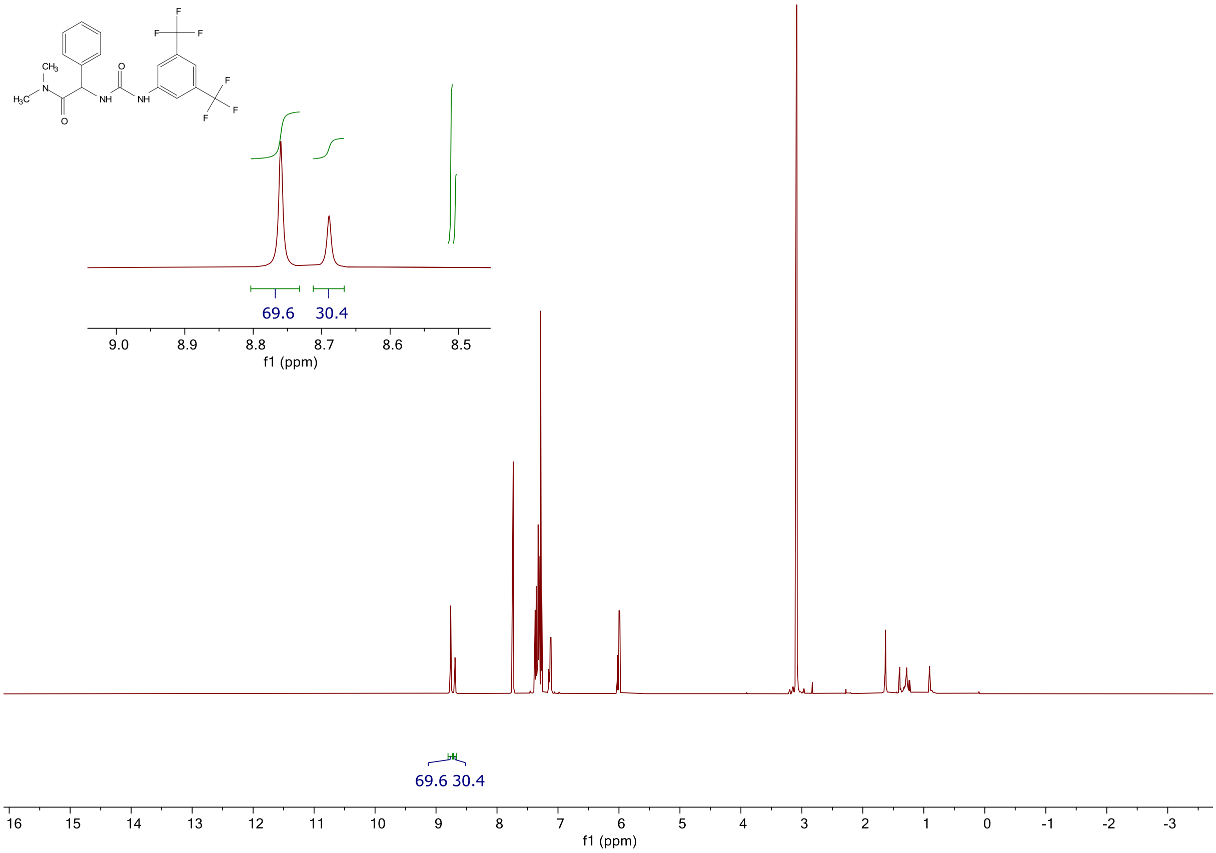

^{19}F-NMR (376 MHz, $CDCl_3$) of **2a** (*e.r.* 30:70)

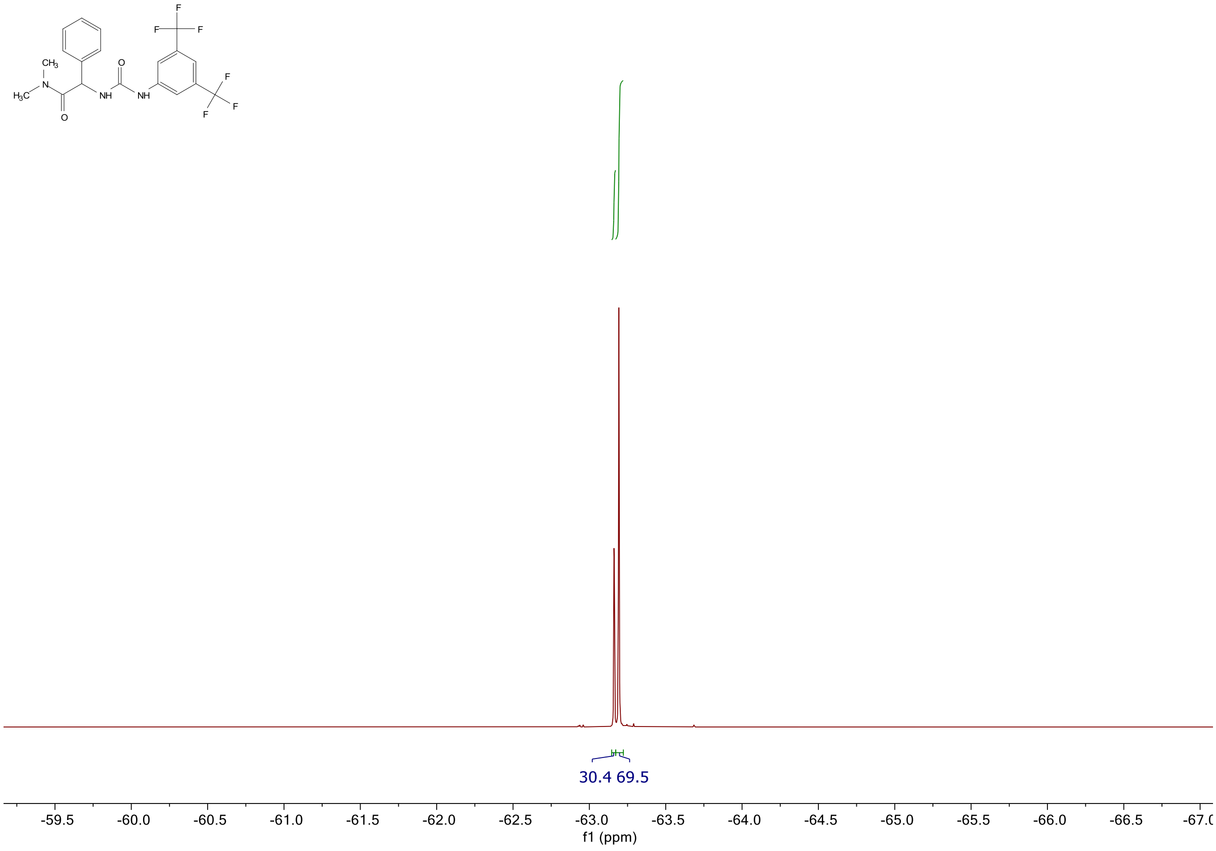

HPLC chromatogram of scalemic **2a** (*e.r.* 30:70)
Conditions: Chiralcel OX-H Lux Cellulose-4, *n*-heptane/^{i}PrOH 85-15, 40 ºC, 1 mL/min, λ_{abs} = 226 nm

<Chromatogram>

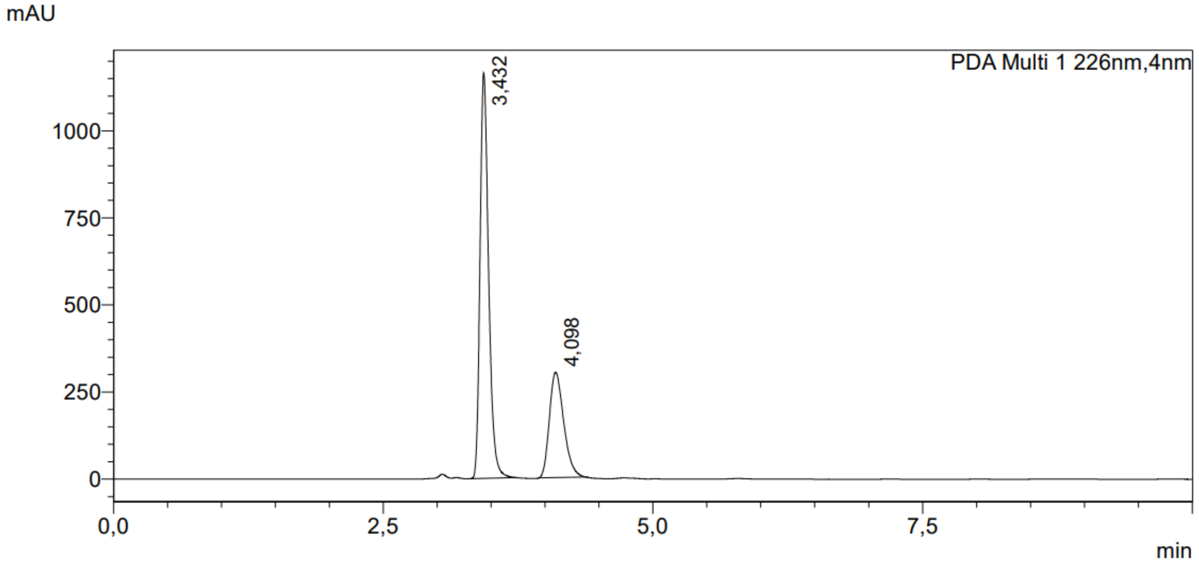

<Peak Table>

PDA Ch1 226nm

Peak#	Ret. Time	Area%
1	3,432	69,775
2	4,098	30,225
Total		100,000

^{1}H-NMR (600 MHz, $CDCl_3$) of **2a** (*e.r.* 40:60)

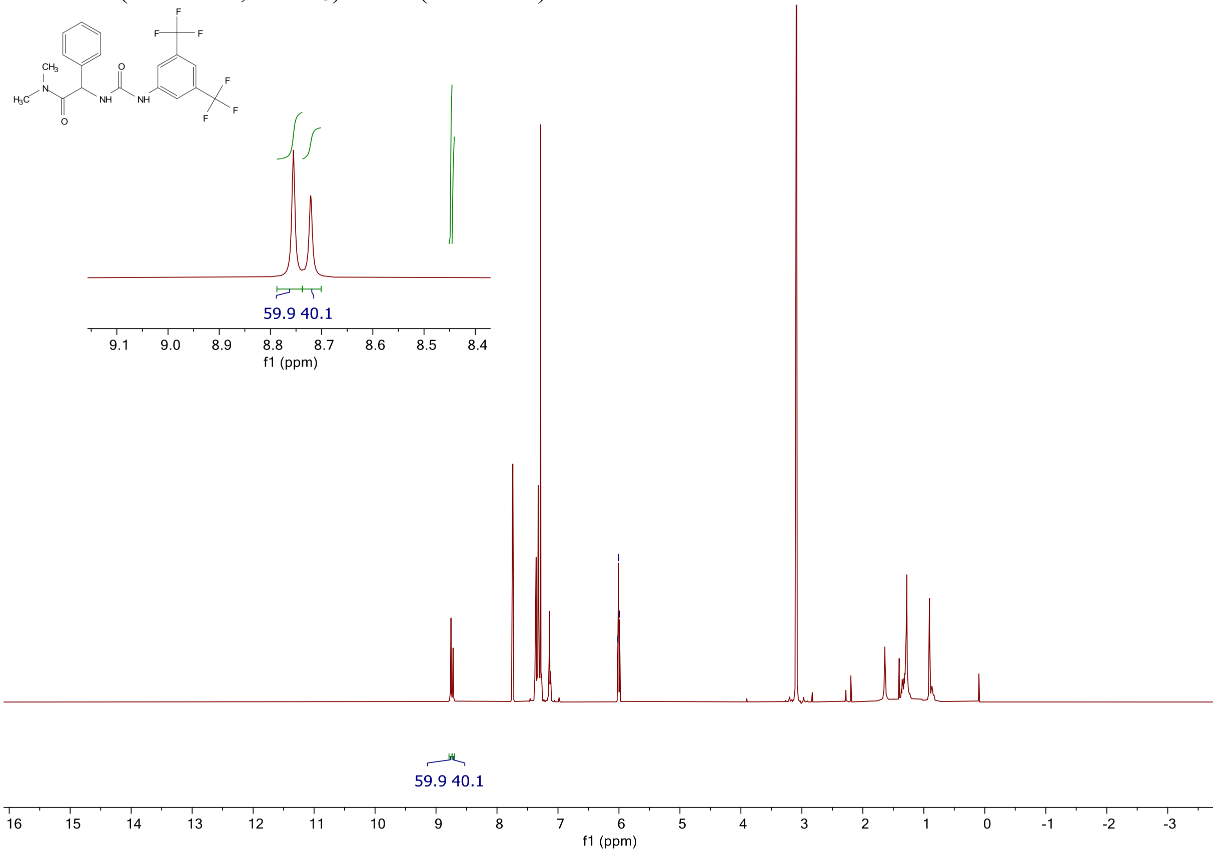

^{19}F-NMR (376 MHz, $CDCl_3$) of **2a** (*e.r.* 40:60)

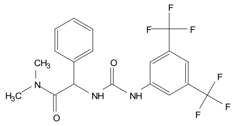

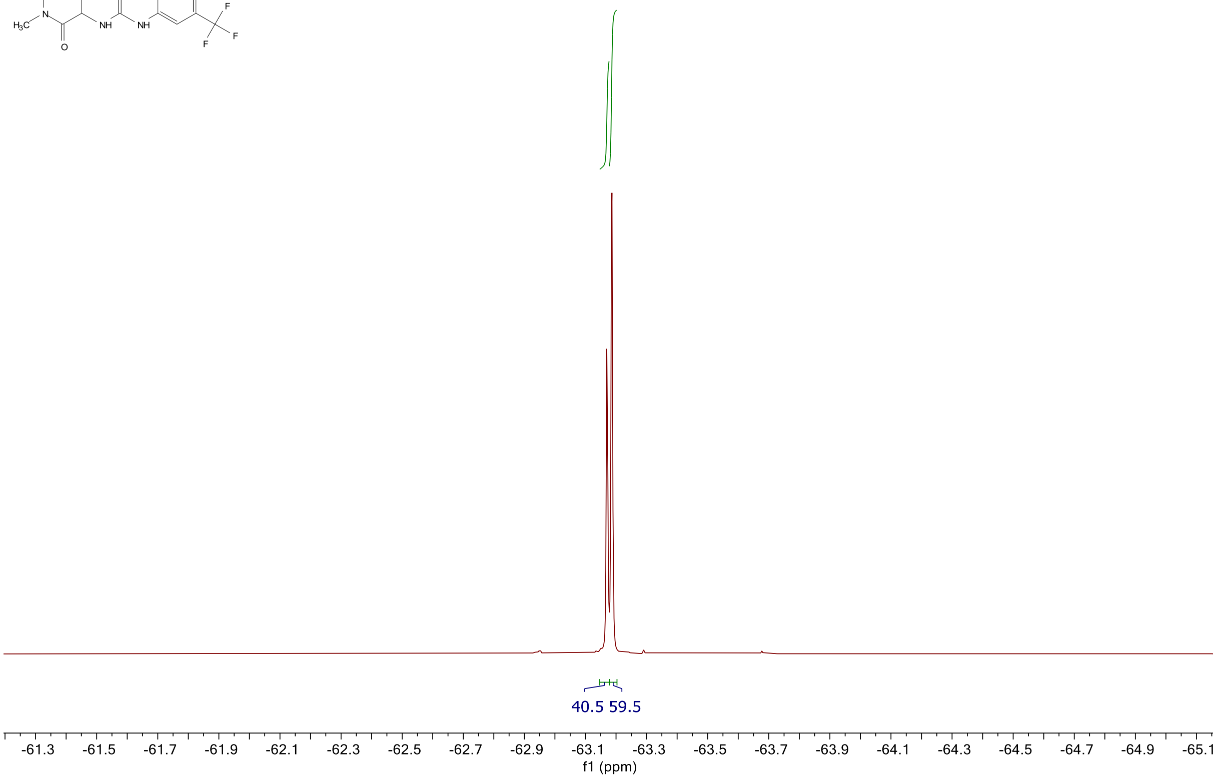

HPLC chromatogram of scalemic **2a** (*e.r.* 40:60)
Conditions: Chiralcel OX-H Lux Cellulose-4, *n*-heptane/^{i}PrOH 85-15, 40 ºC, 1 mL/min, λ_{abs} = 227 nm

<Chromatogram>

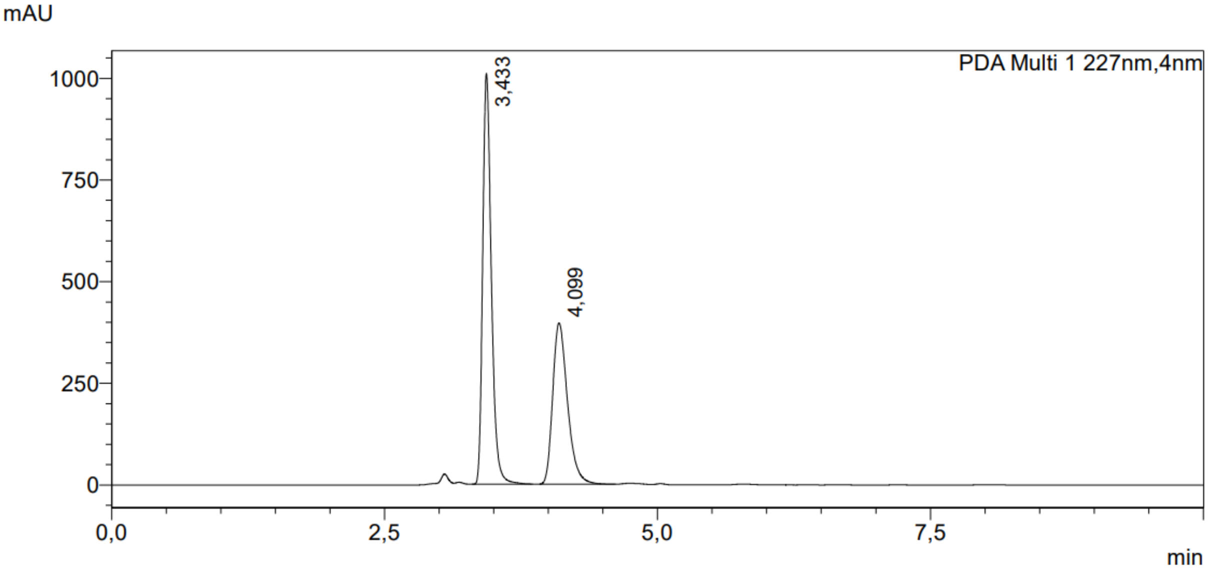

<Peak Table>

PDA Ch1 227nm

Peak#	Ret. Time	Area%
1	3,433	59,760
2	4,099	40,240
Total		100,000

^{1}H-NMR (600 MHz, $CDCl_3$) of **2a** (*rac*)

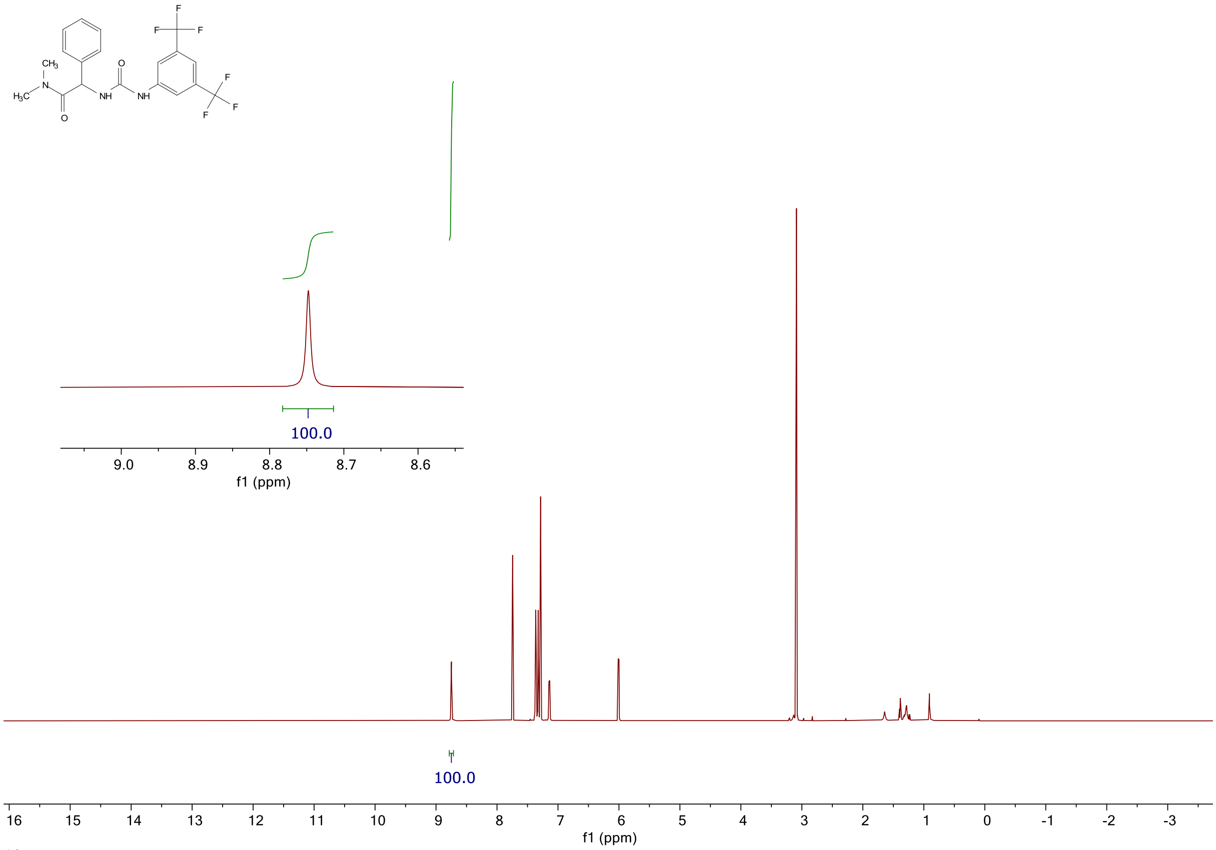

^{19}F-NMR (376 MHz, $CDCl_3$) of **2a** (*rac*)

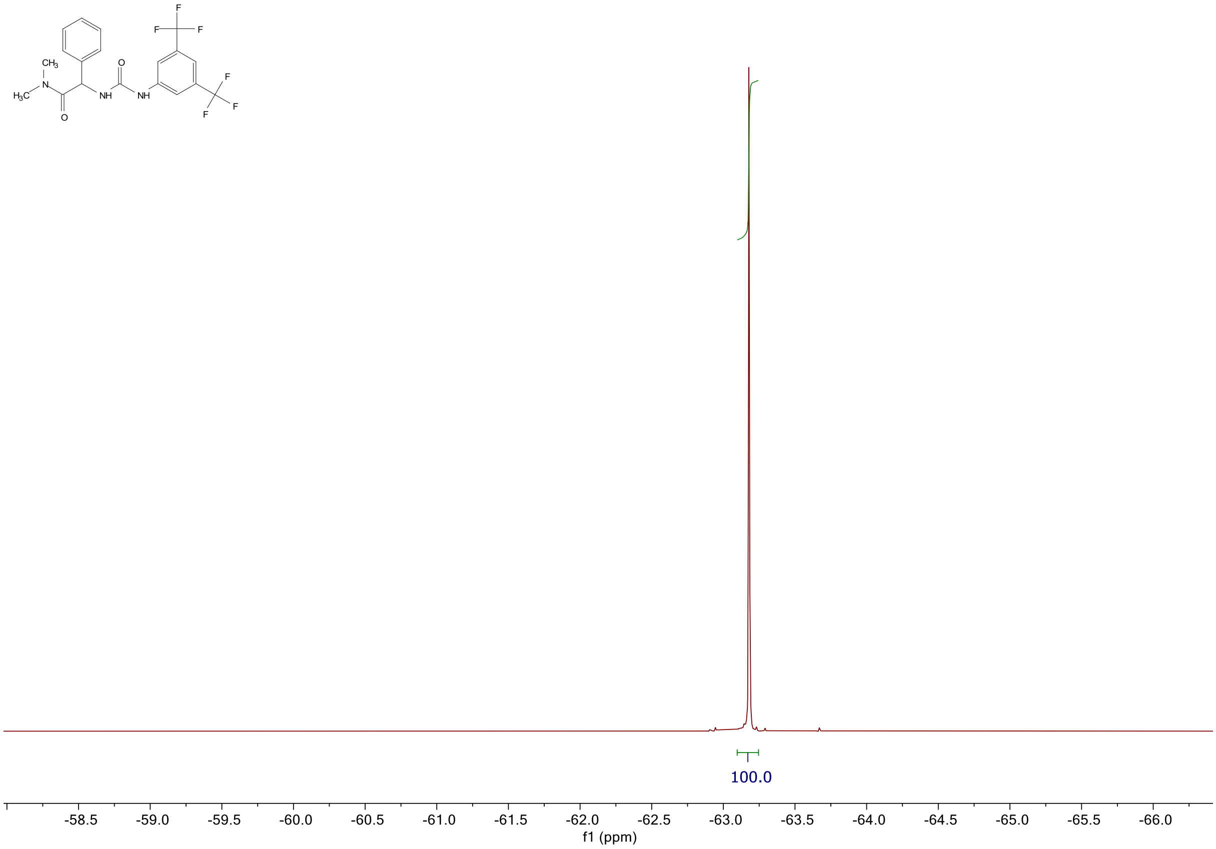

HPLC chromatogram of scalemic **2a** (*rac*)
Conditions: Chiralcel OX-H Lux Cellulose-4, *n*-heptane/^{i}PrOH 85-15, 40 ºC, 1 mL/min, λ_{abs} = 222 nm

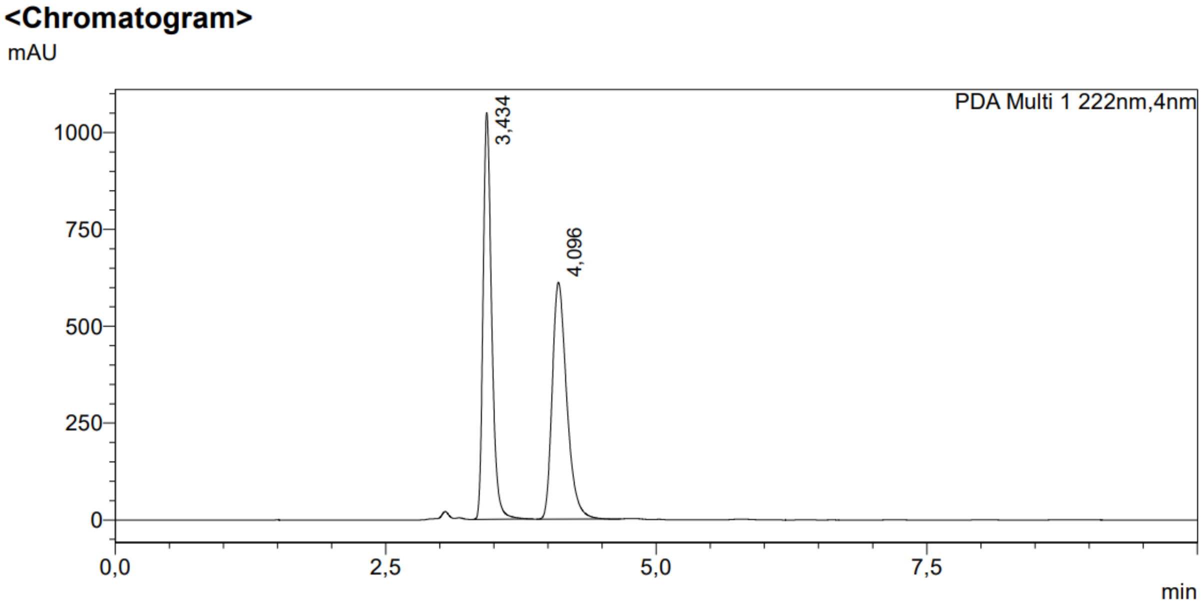

<Peak Table>

PDA Ch1 222nm

Peak#	Ret. Time	Name	Area%
1	3,434		50,338
2	4,096		49,662
Total			100,000

^{1}H-NMR (600 MHz, $CDCl_3$) of **2a** (*e.r.* 60:40)

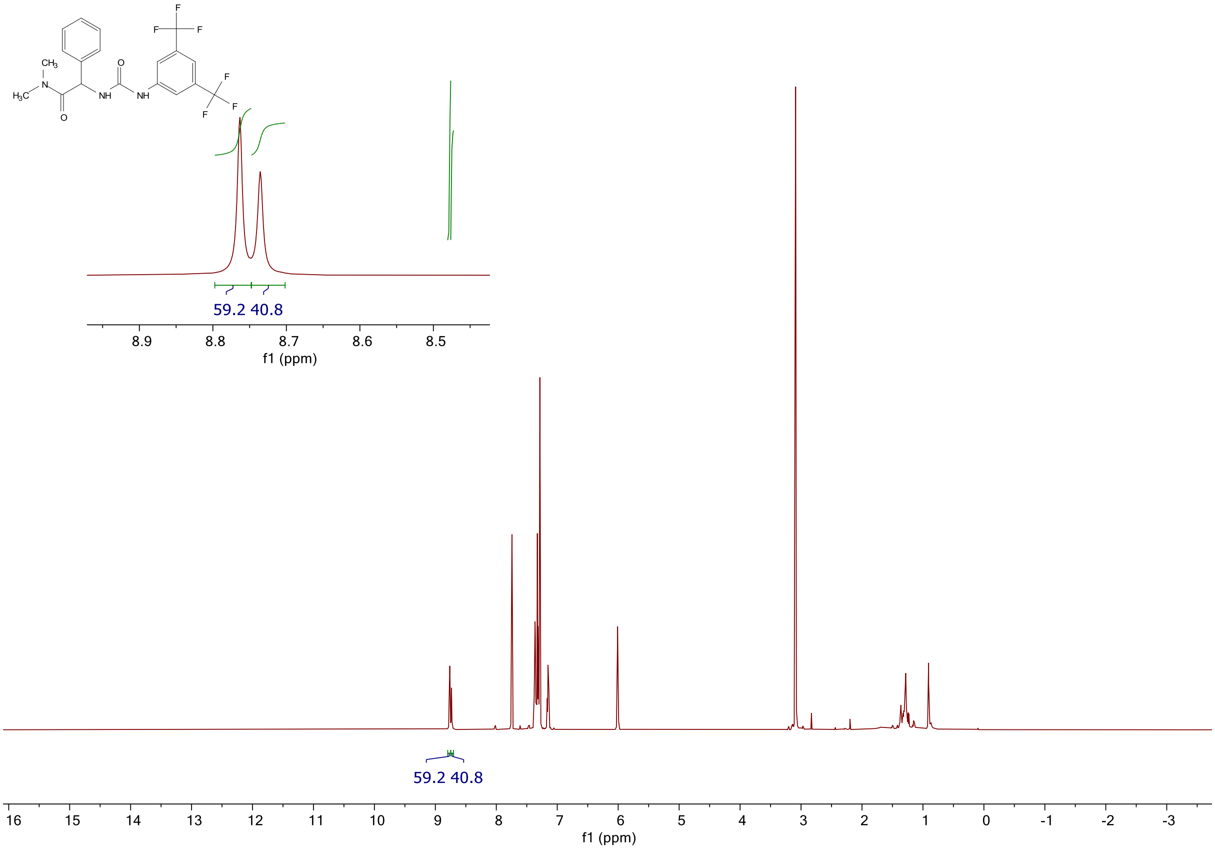

^{19}F-NMR (376 MHz, $CDCl_3$) of **2a** (*e.r.* 60:40)

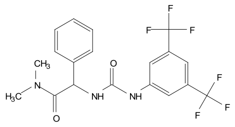

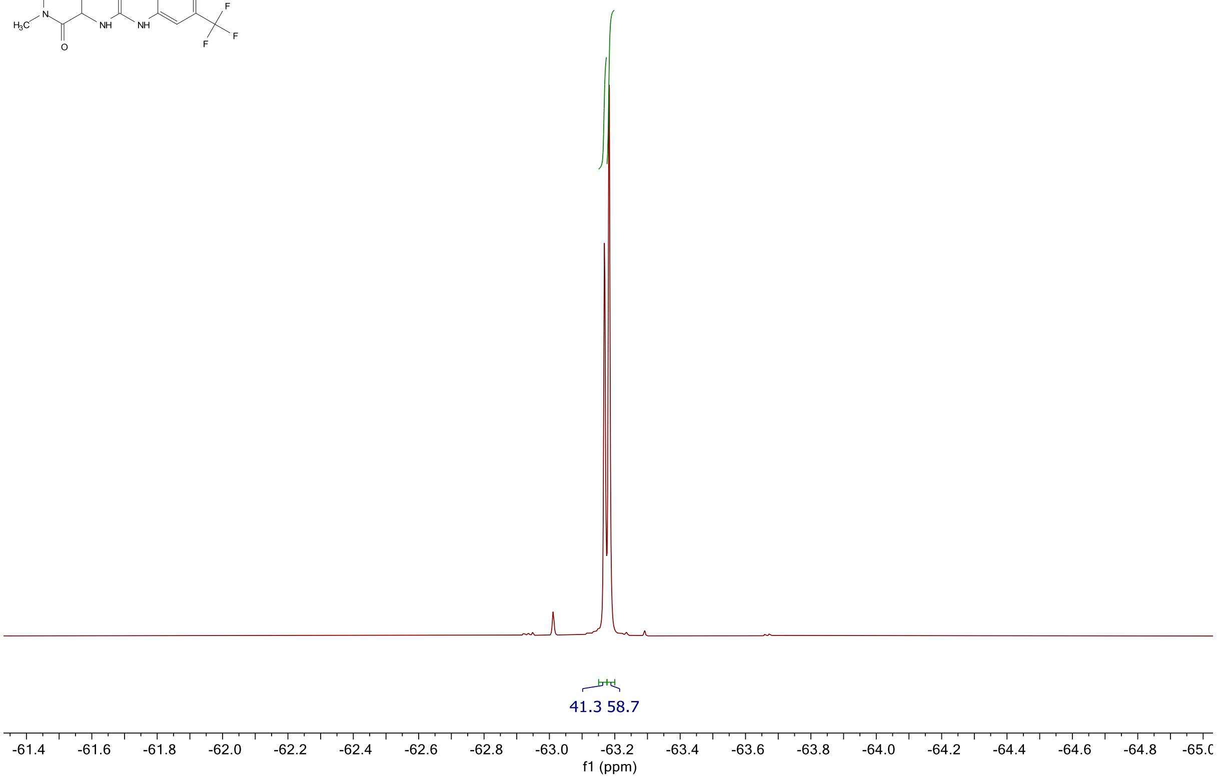

HPLC chromatogram of scalemic **2a** (*e.r.* 60:40)
Conditions: Chiralcel OX-H Lux Cellulose-4, *n*-heptane/^{i}PrOH 85-15, 40 ºC, 1 mL/min, λ_{abs} = 248 nm

<Chromatogram>

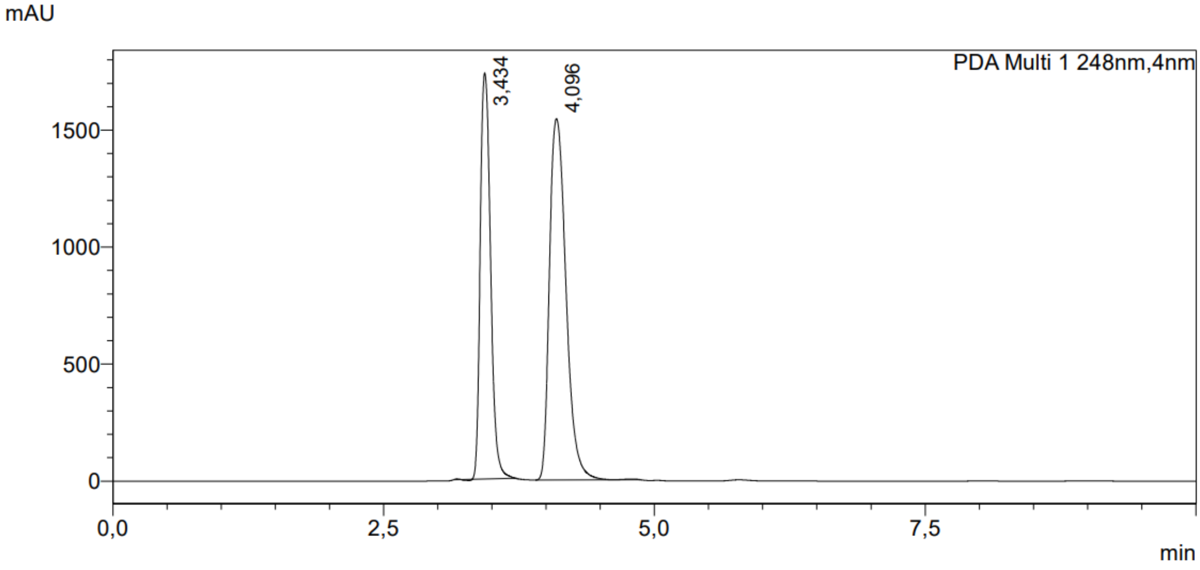

<Peak Table>

PDA Ch1 248nm

Peak#	Ret. Time	Area%
1	3,434	41,166
2	4,096	58,834
Total		100,000

^{1}H-NMR (600 MHz, $CDCl_3$) of **2a** (*e.r.* 70:30)

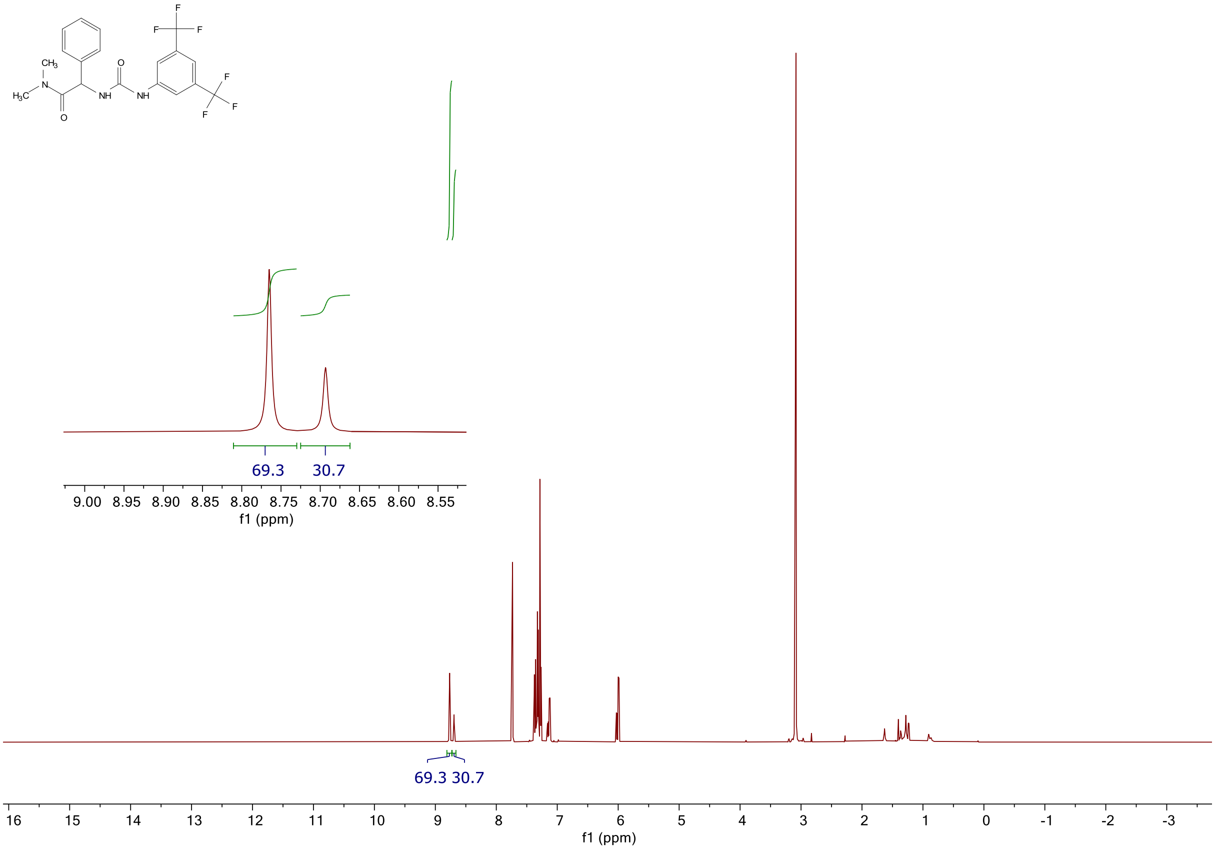

^{19}F-NMR (376 MHz, $CDCl_3$) of **2a** (*e.r.* 70:30)

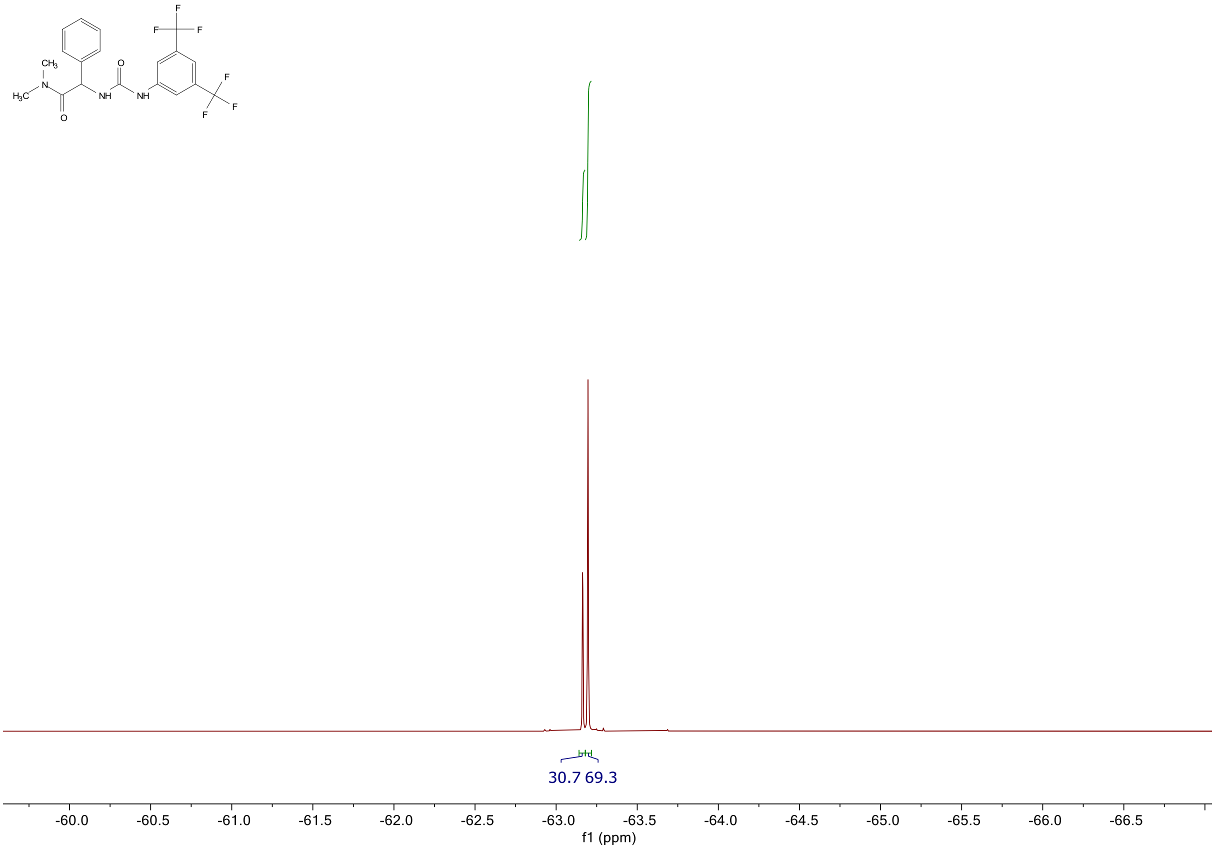

HPLC chromatogram of scalemic **2a** (*e.r.* 70:30)
Conditions: Chiralcel OX-H Lux Cellulose-4, *n*-heptane/^{i}PrOH 85-15, 40 ºC, 1 mL/min, λ_{abs} = 221 nm

<Chromatogram>

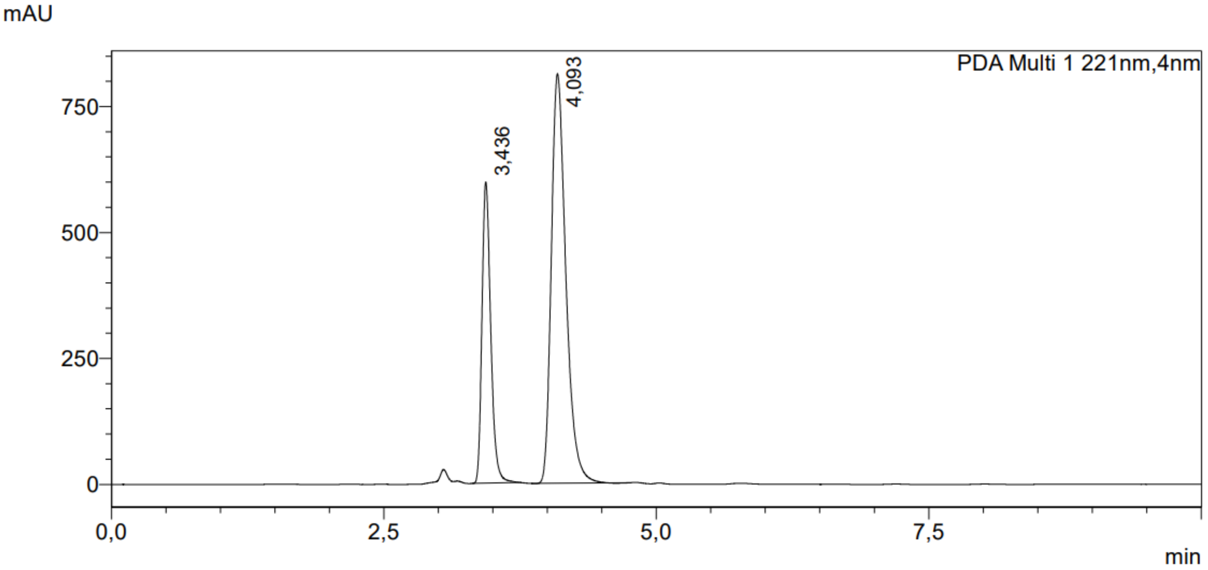

<Peak Table>

PDA Ch1 221nm

Peak#	Ret. Time	Area%
1	3,436	30,720
2	4,093	69,280
Total		100,000

^{1}H-NMR (600 MHz, $CDCl_3$) of **2a** (*e.r.* 80:20)

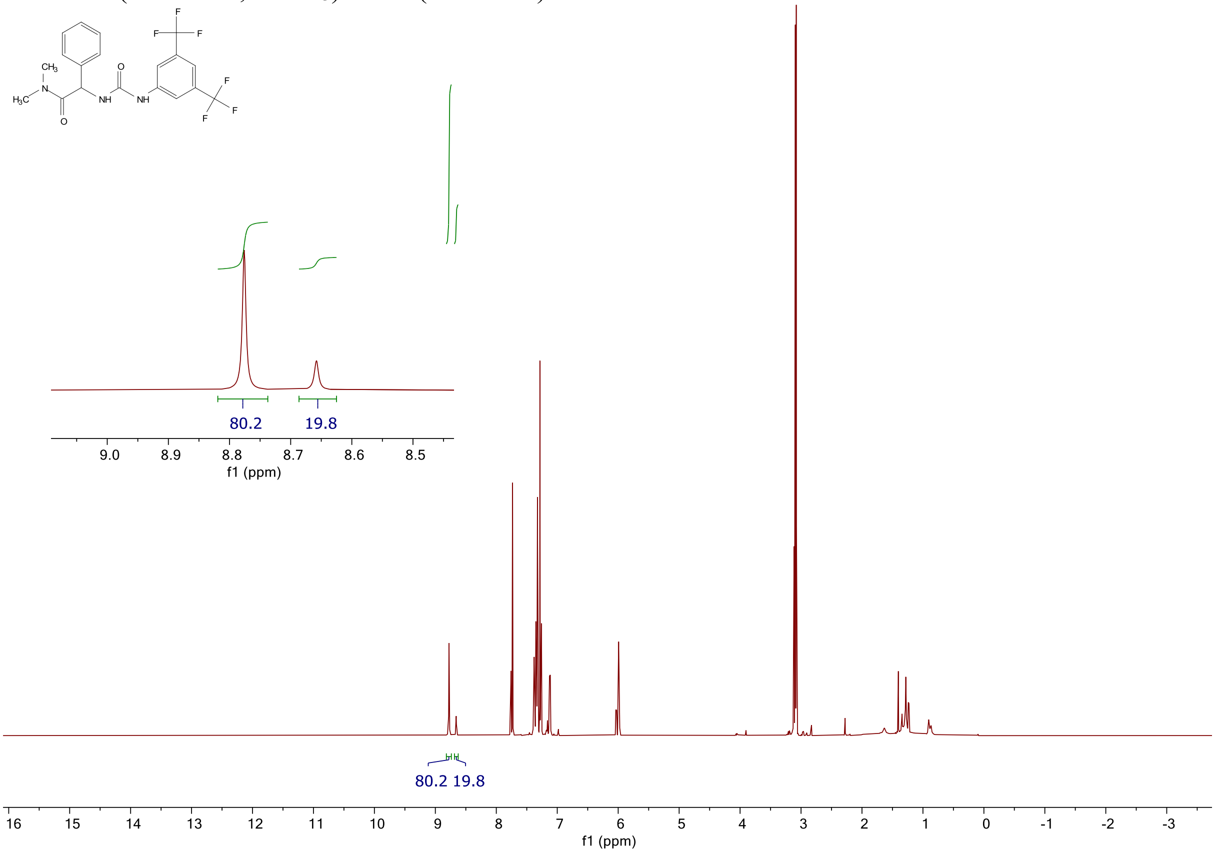

^{19}F-NMR (376 MHz, $CDCl_3$) of **2a** (*e.r.* 80:20)

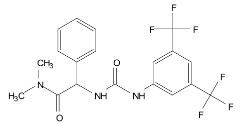

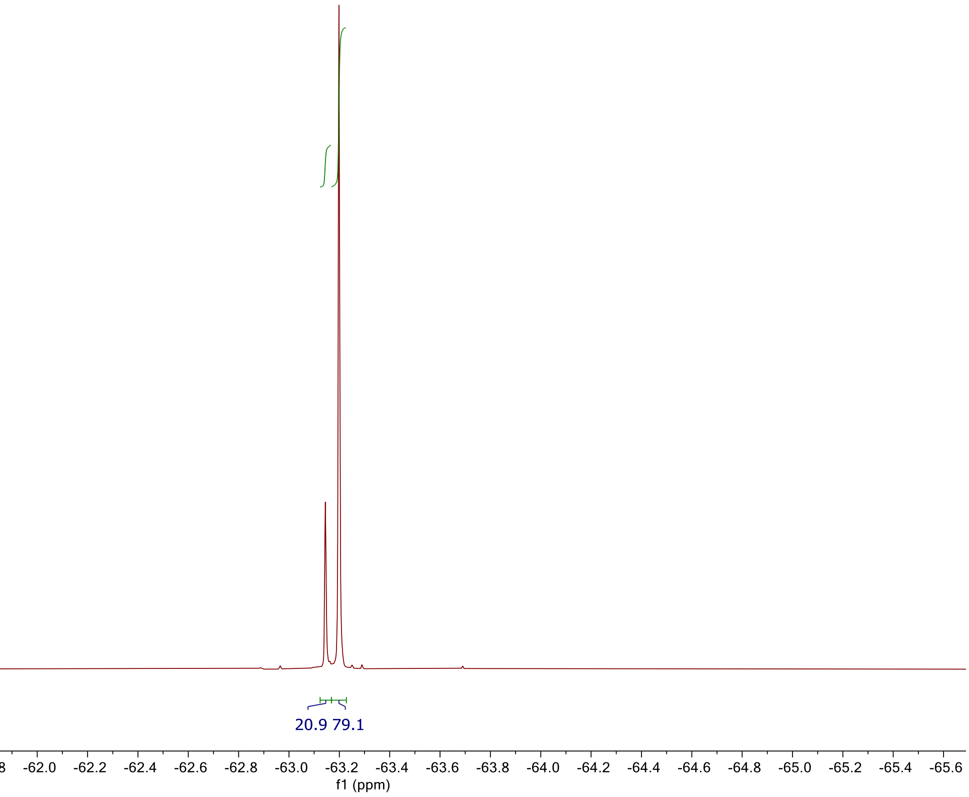

HPLC chromatogram of scalemic **2a** (*e.r.* 80:20)

Conditions: Chiralcel OX-H Lux Cellulose-4, *n*-heptane/^{i}PrOH 85-15, 40 ºC, 1 mL/min, λ_{abs} = 224 nm

<Chromatogram>

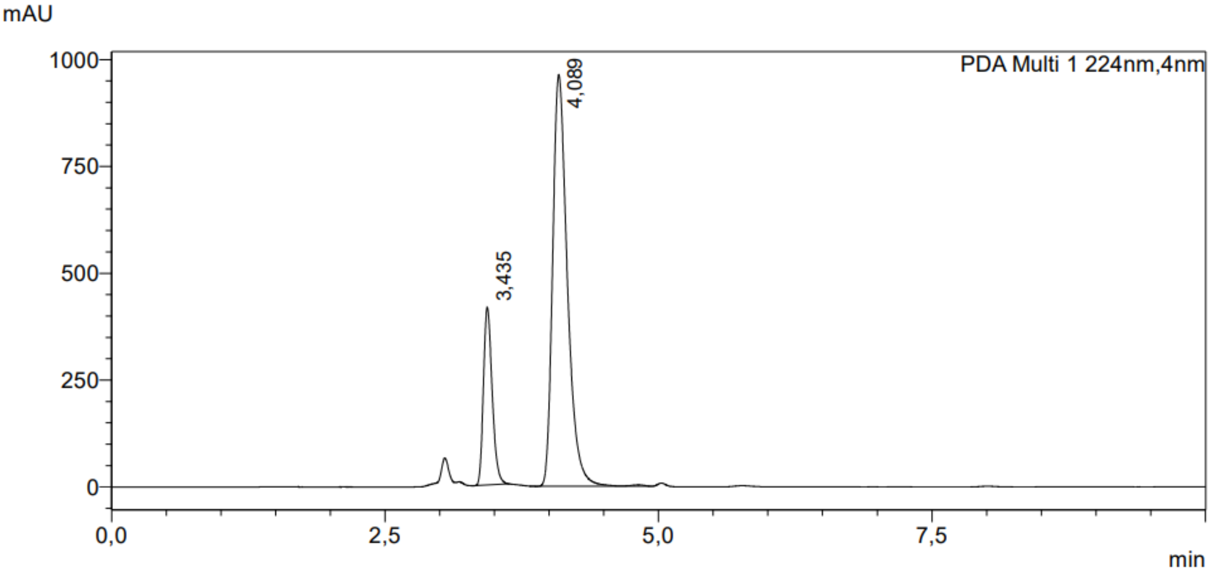

<Peak Table>

PDA Ch1 224nm

Peak#	Ret. Time	Area%
1	3,435	20,465
2	4,089	79,535
Total		100,000

^{1}H-NMR (600 MHz, $CDCl_3$) of **2a** (*e.r.* 90:10)

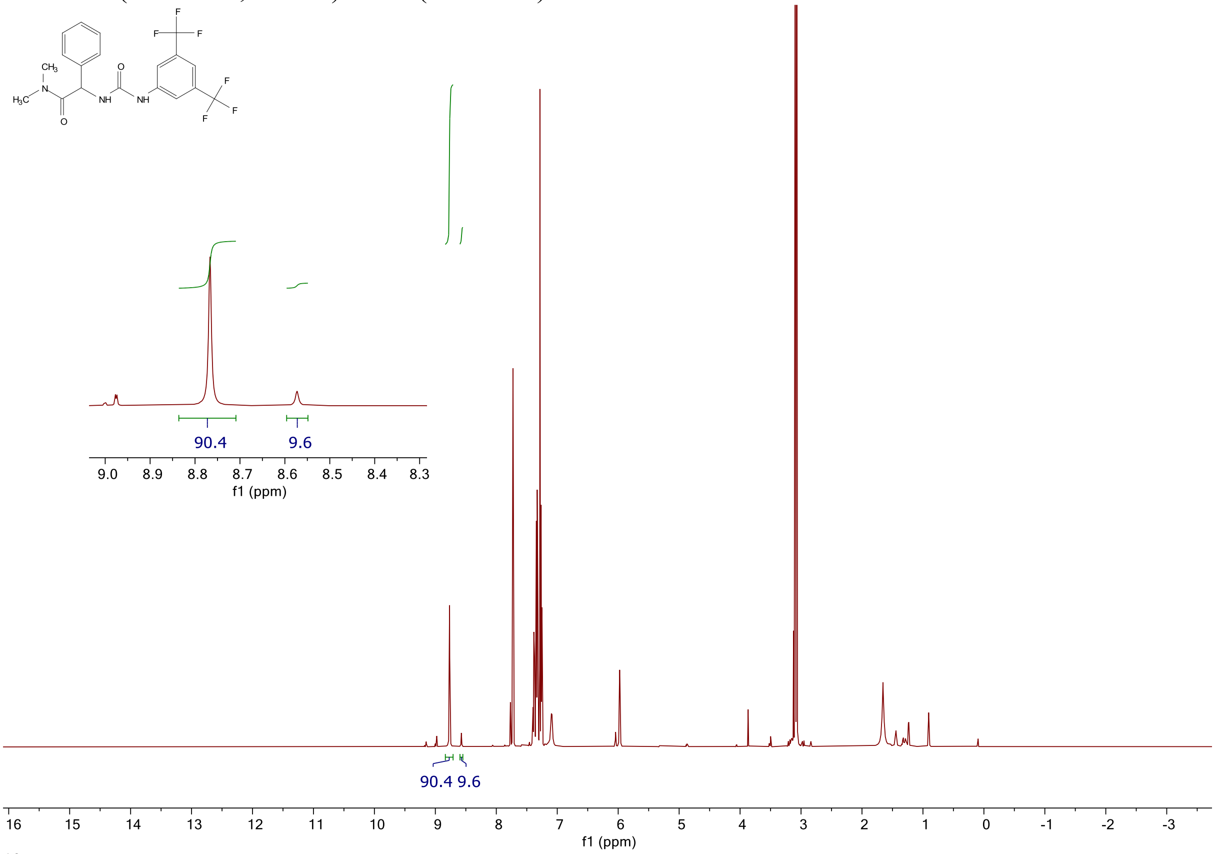

^{19}F-NMR (376 MHz, $CDCl_3$) of **2a** (*e.r.* 90:10)

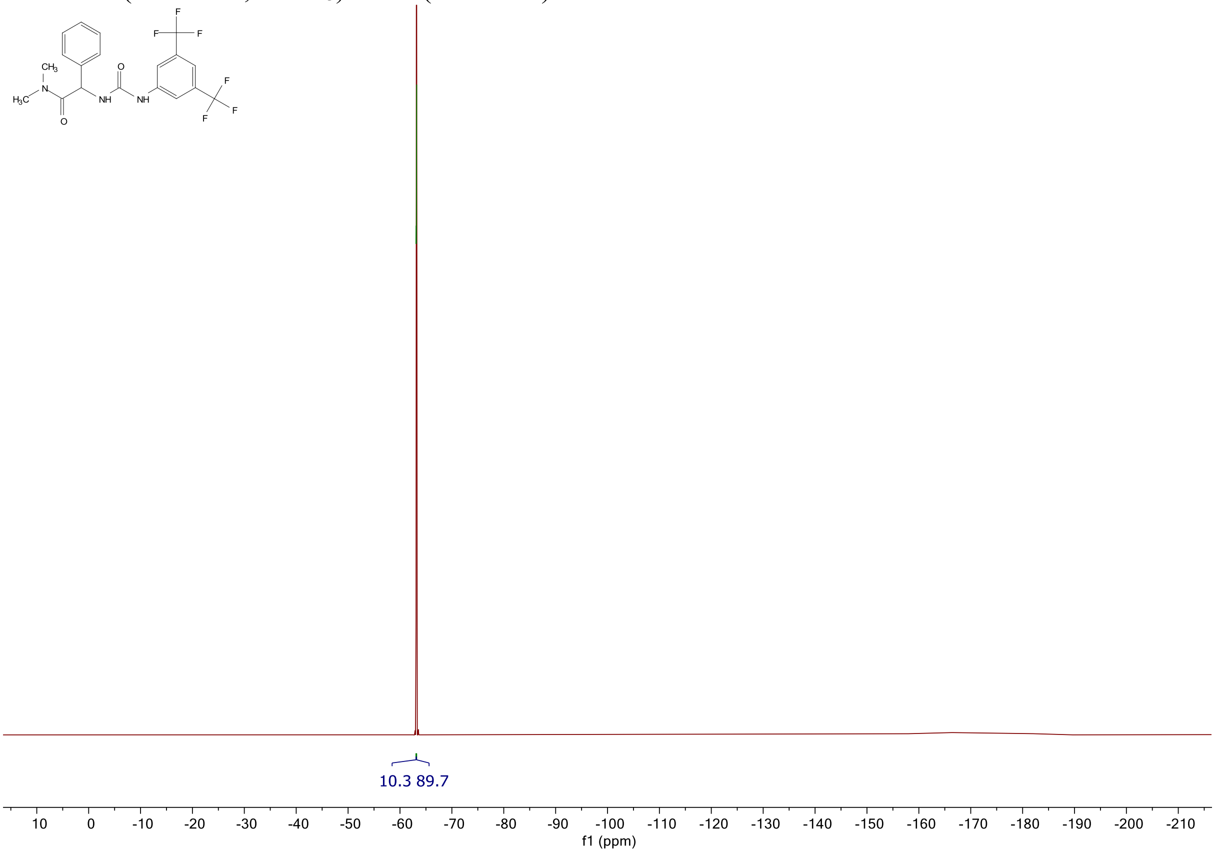

HPLC chromatogram of scalemic **2a** (*e.r.* 90:10)

Conditions: Chiralcel OX-H Lux Cellulose-4, *n*-heptane/^{i}PrOH 85-15, 40 ºC, 1 mL/min, λ_{abs} = 221 nm

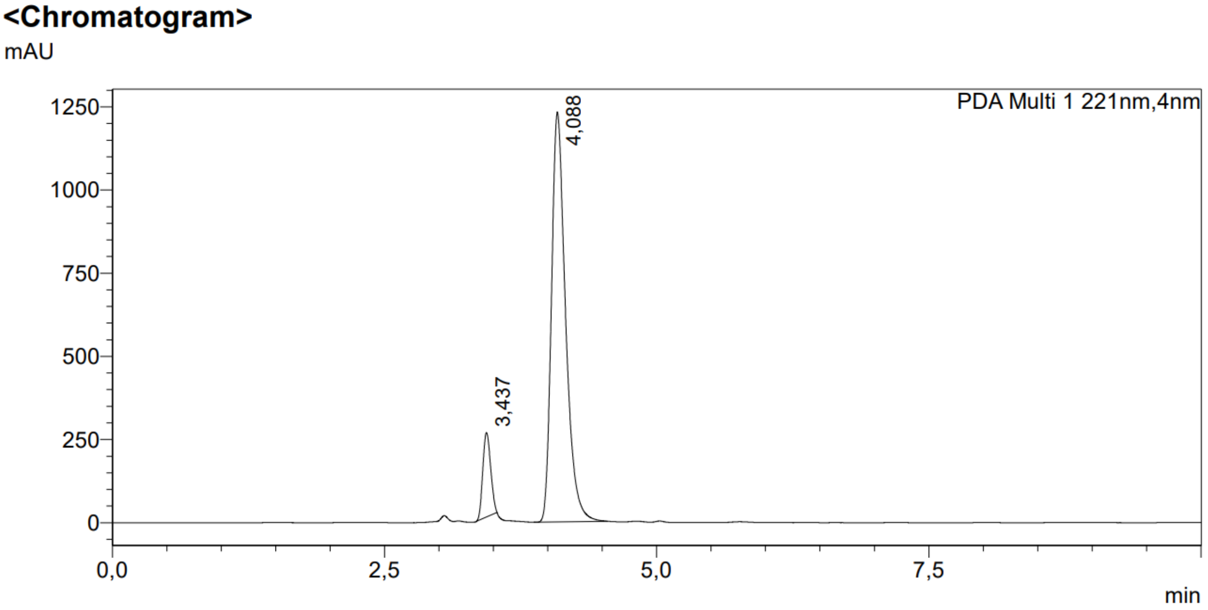

<Peak Table>

PDA Ch1 221nm

Peak#	Area%	Ret. Time
1	10,329	3,437
2	89,671	4,088
Total	100,000	

The enantiomeric ratio of **5** was calculated using qGSD.

^{1}H-NMR (400 MHz, toluene-d_8) of **5** (*e.r.* 10:90)

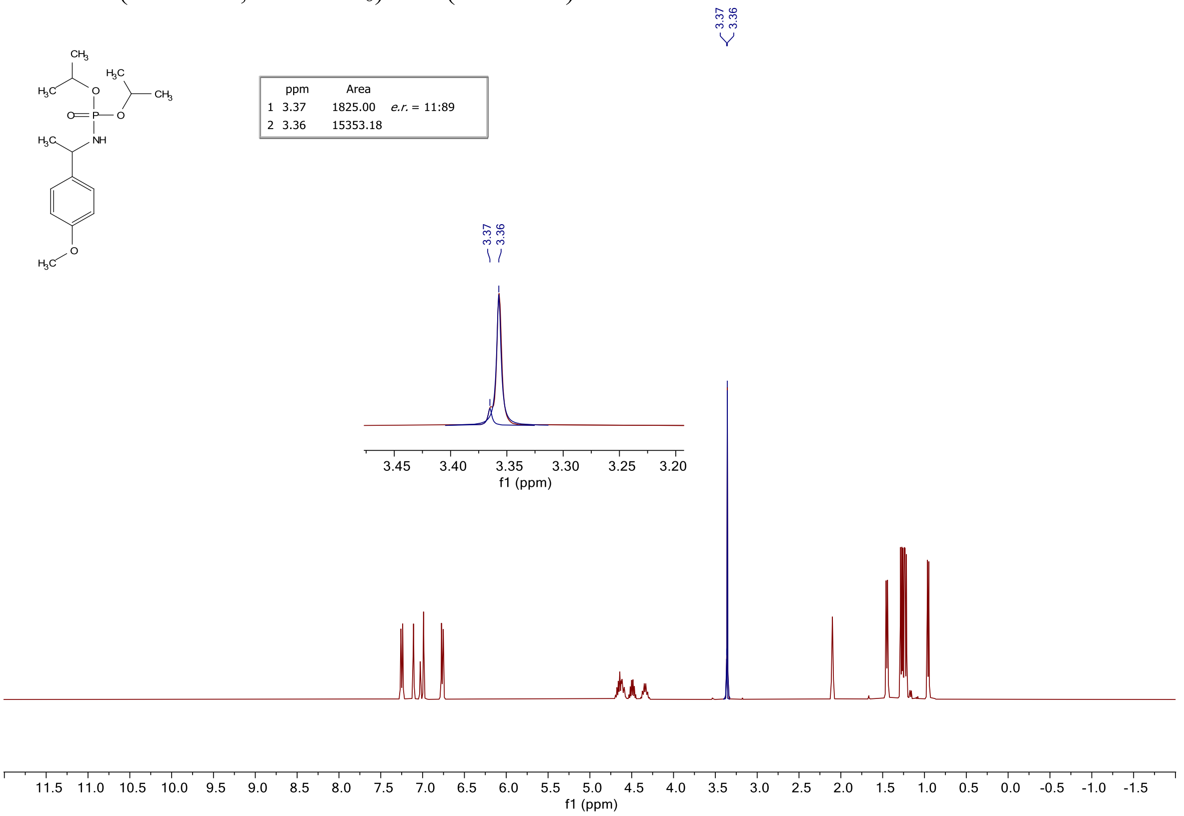

^{31}P-NMR (162 MHz, toluene-d_8) of **5** (*e.r.* 10:90)

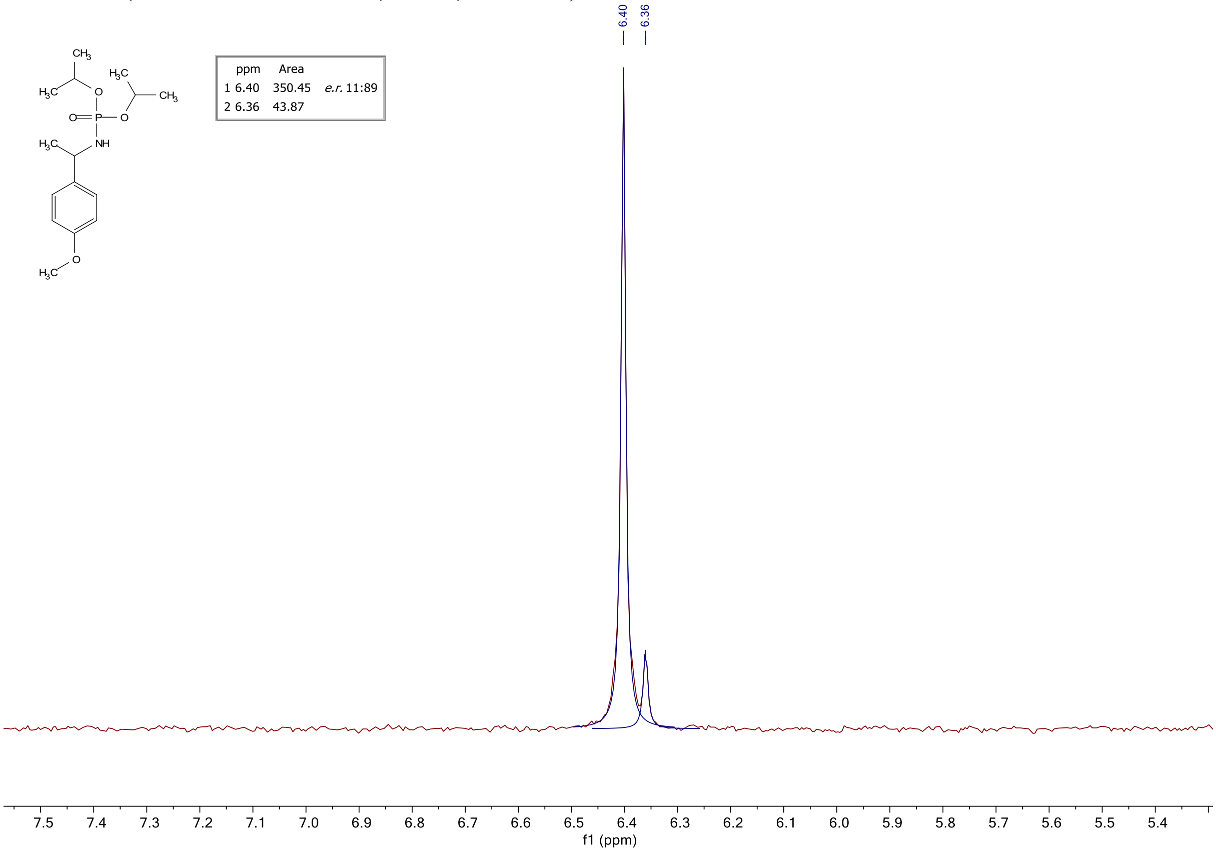

HPLC chromatogram of scalemic **5** (*e.r.* 10:90)

Conditions: Chiralpak AD-H, *n*-heptane/^{i}PrOH 93-7 40 ºC, 1 mL/min, λ_{abs} = 254 nm

<Chromatogram>

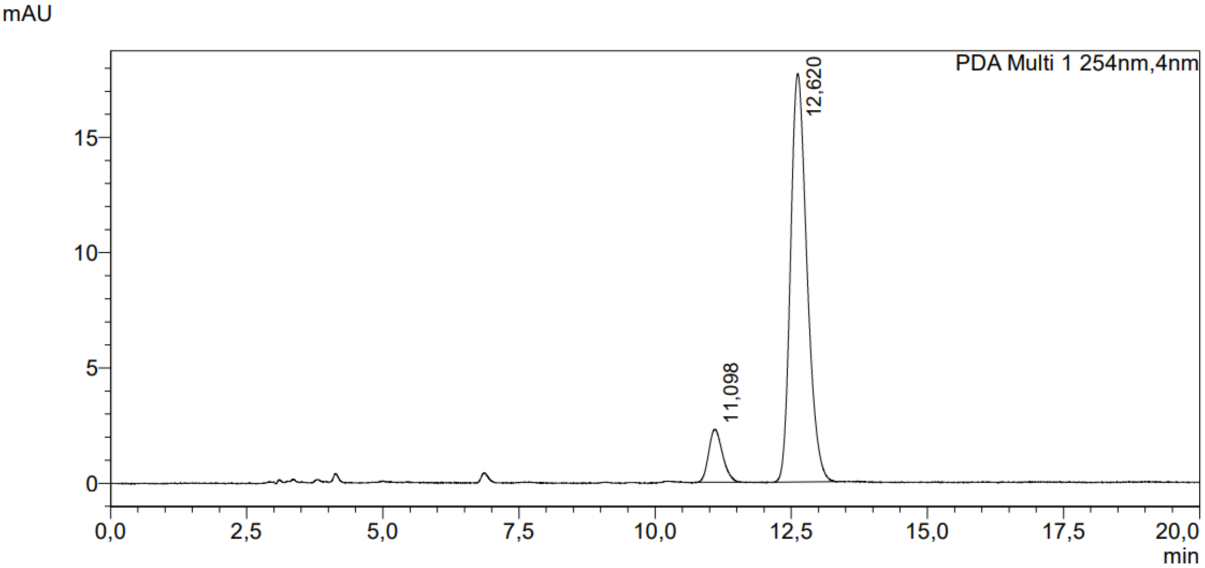

<Peak Table>

PDA Ch1 254nm

Peak#	Ret. Time	Area%
1	11,098	9,838
2	12,620	90,162
Total		100,000

^{1}H-NMR (400 MHz, toluene-d_8) of **5** (*e.r.* 20:80)

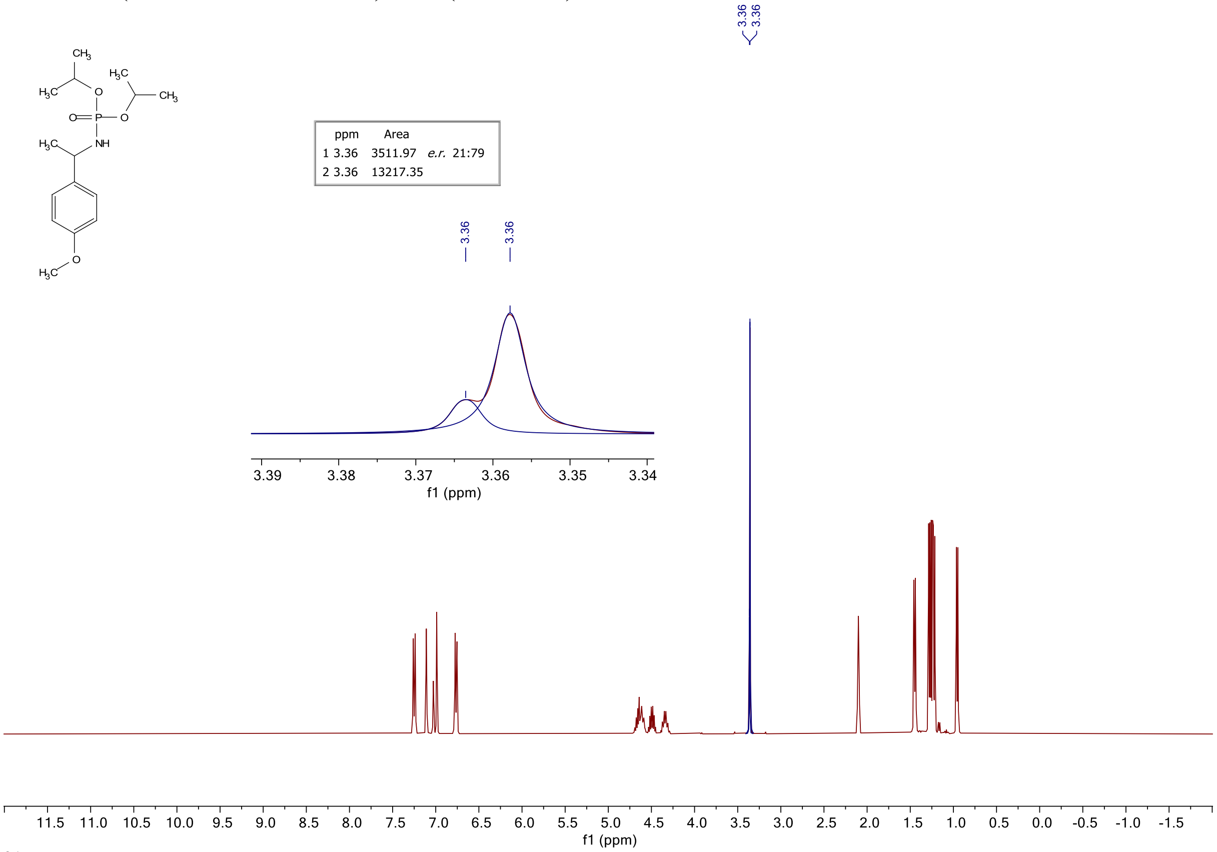

^{31}P-NMR (162 MHz, toluene-d_8) of **5** (*e.r.* 20:80)

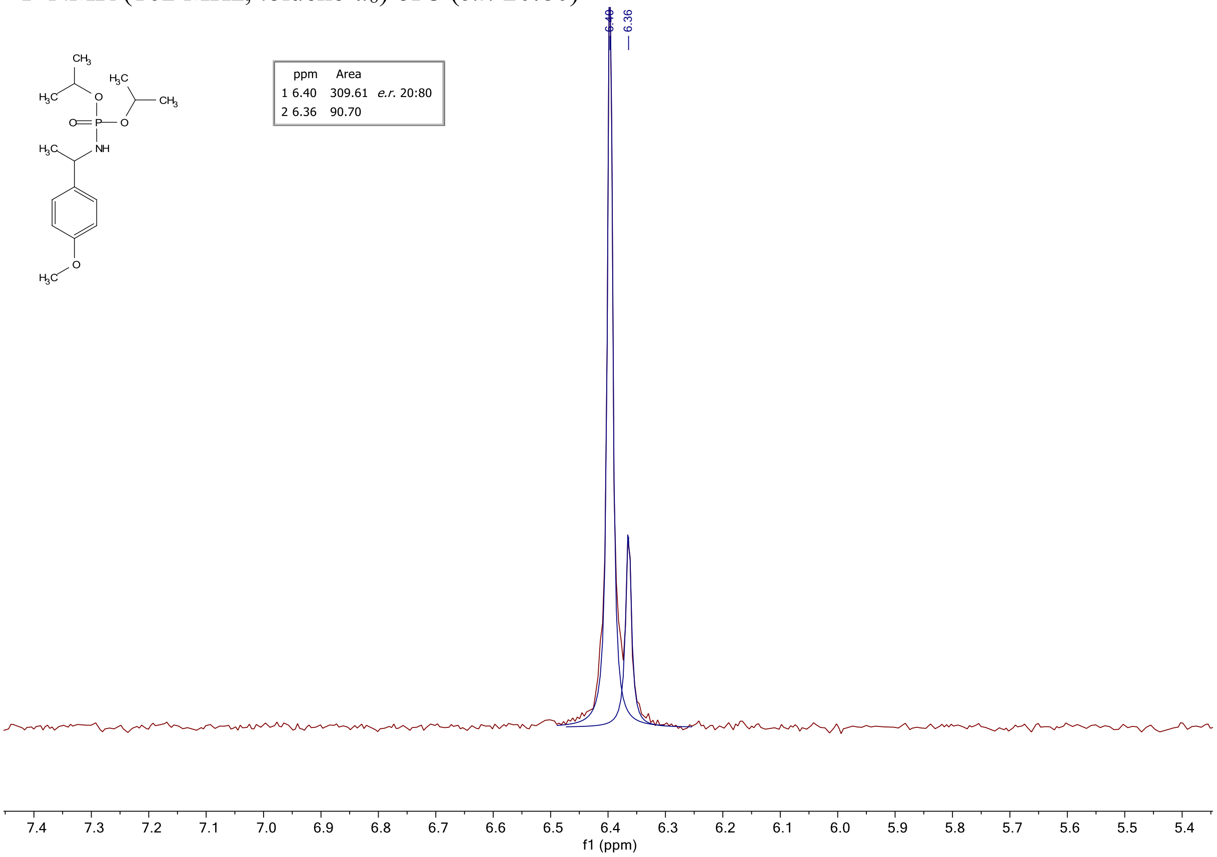

HPLC chromatogram of scalemic **5** (*e.r.* 20:80)
Conditions: Chiralpak AD-H, *n*-heptane/^{i}PrOH 93-7 40 °C, 1 mL/min, λ_{abs} = 254 nm

<Chromatogram>

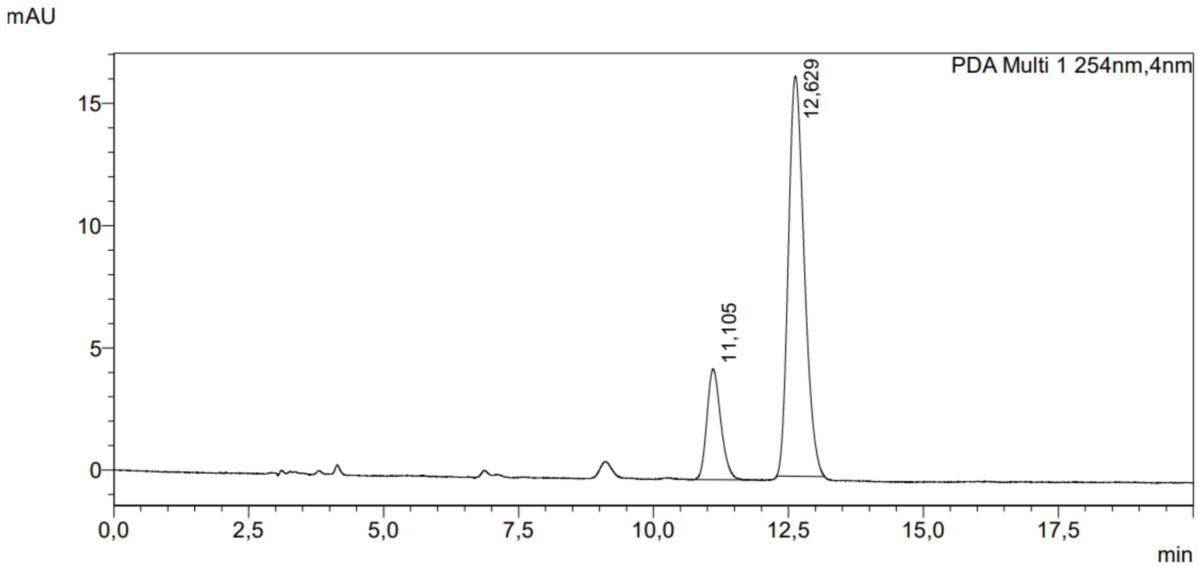

<Peak Table>

PDA Ch1 254nm

Peak#	Ret. Time	Area	Area%	Height%
1	11,105	81188	19,523	21,655
2	12,629	334679	80,477	78,345
Total		415867	100,000	100,000

^{1}H-NMR (400 MHz, toluene-d_8) of **5** (*e.r.* 30:70)

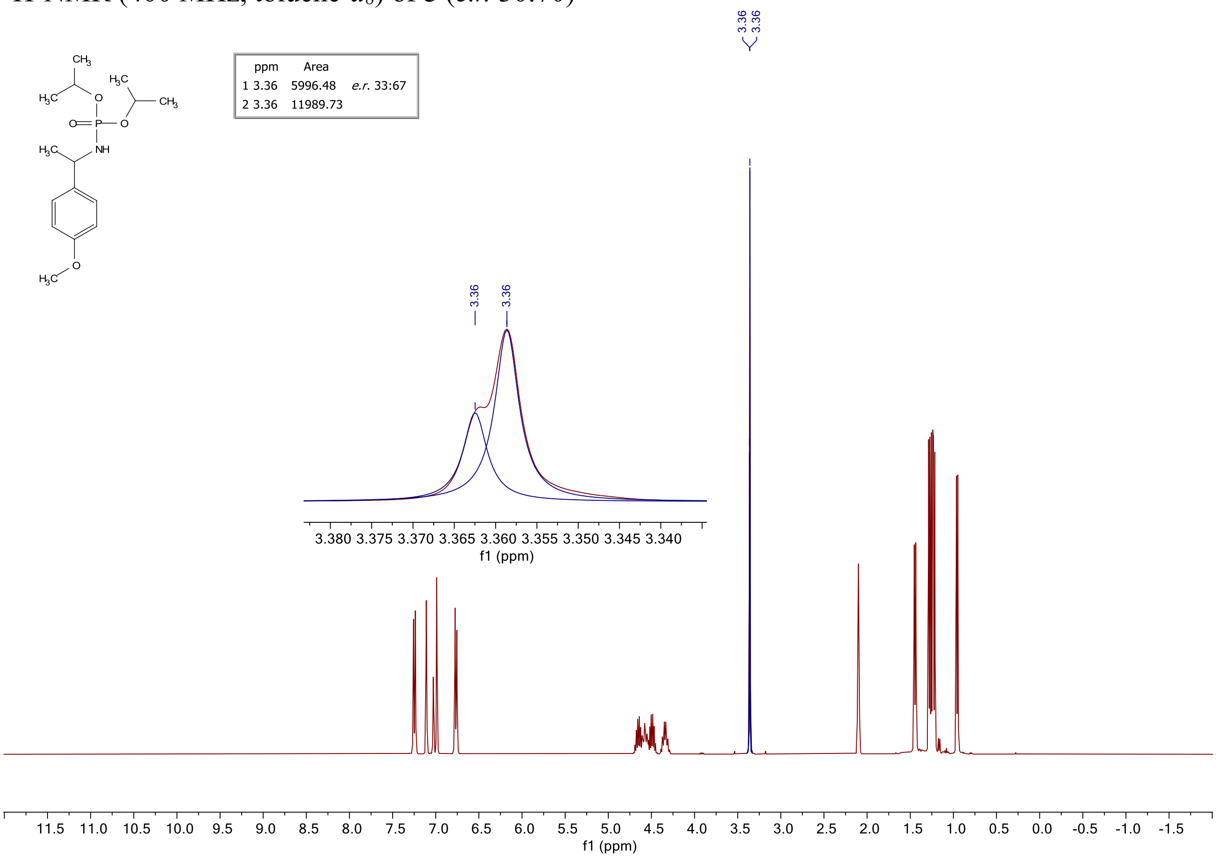

^{31}P-NMR (162 MHz, toluene-d_8) of **5** (*e.r.* 30:70)

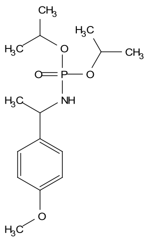

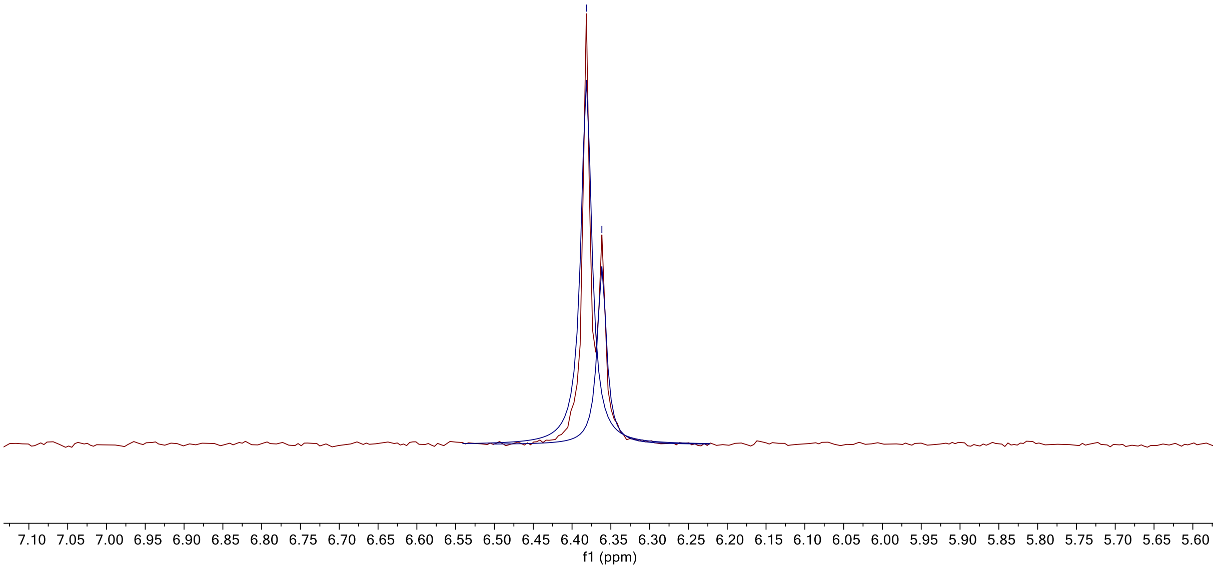

HPLC chromatogram of scalemic **5** (*e.r.* 30:70)
Conditions: Chiralpak AD-H, *n*-heptane/^{i}PrOH 93-7 40 ºC, 1 mL/min, λ_{abs} = 254 nm

<Chromatogram>

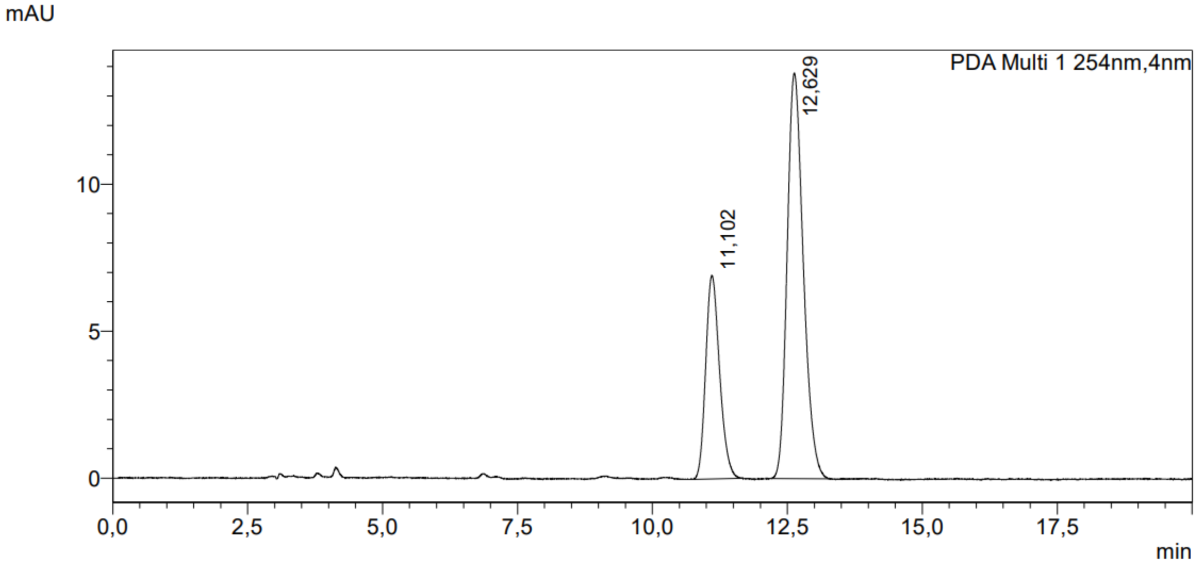

<Peak Table>

PDA Ch1 254nm

Peak#	Ret. Time	Area%
1	11,102	30,176
2	12,629	69,824
Total		100,000

^{1}H-NMR (400 MHz, toluene-d_8) of **5** (*e.r.* 40:60)

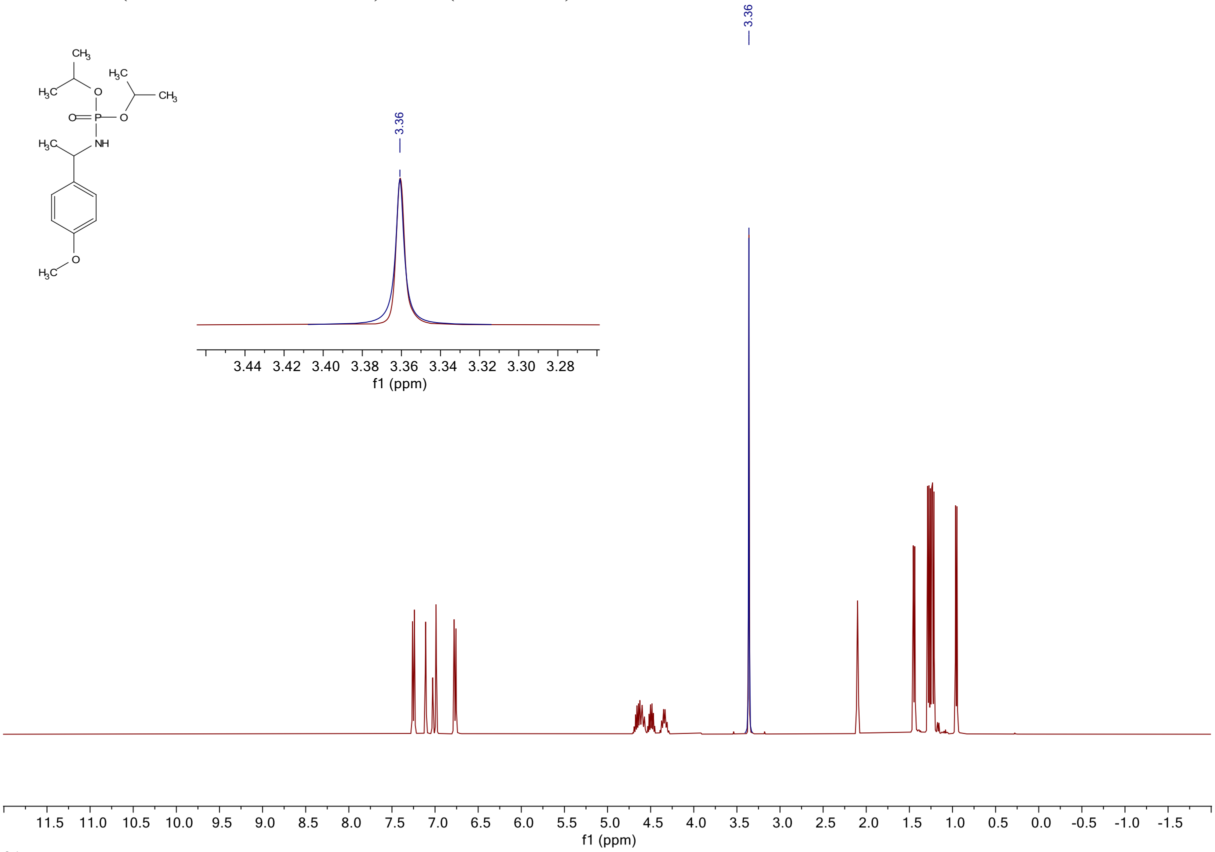

^{31}P-NMR (162 MHz, toluene-d_8) of **5** (*e.r.* 40:60)

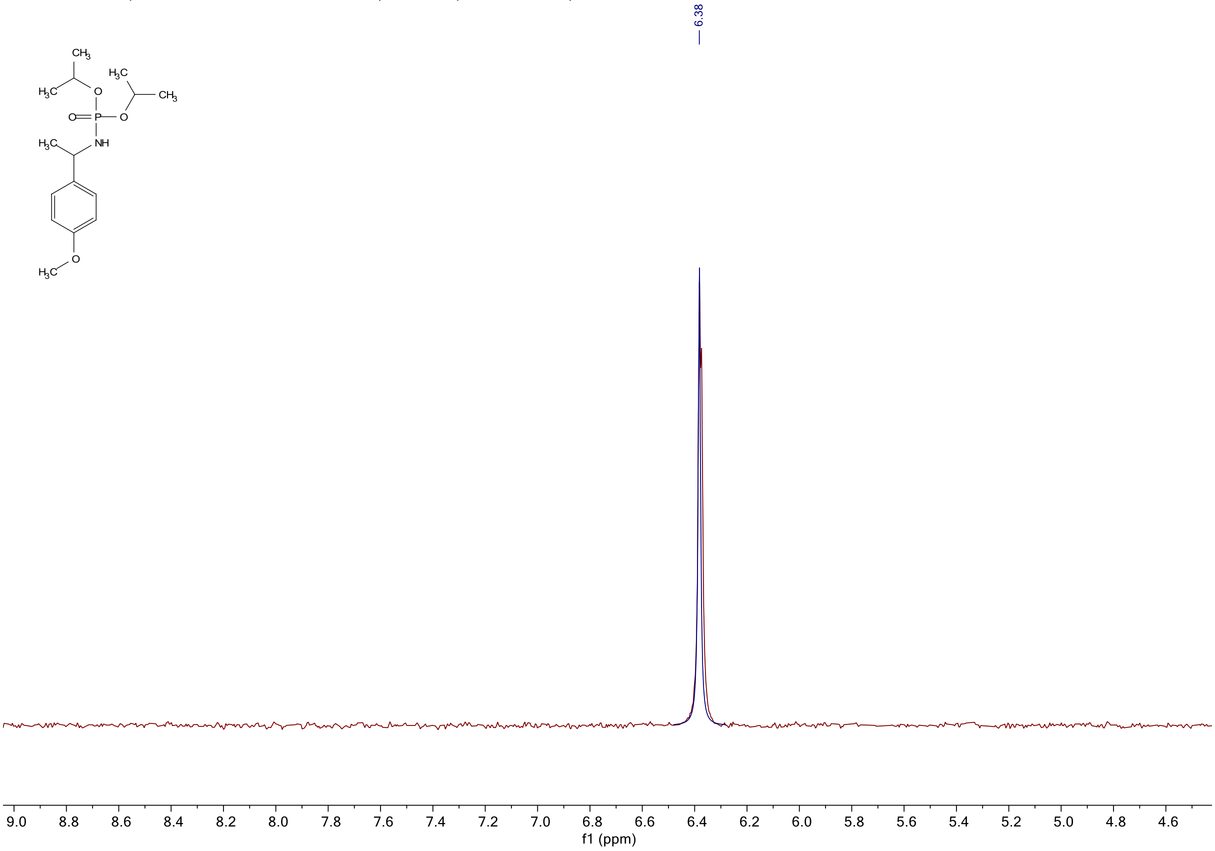

HPLC chromatogram of scalemic **5** (*e.r.* 40:60)
Conditions: Chiralpak AD-H, *n*-heptane/^{i}PrOH 93-7 40 °C, 1 mL/min, λ_{abs} = 254 nm

<Chromatogram>

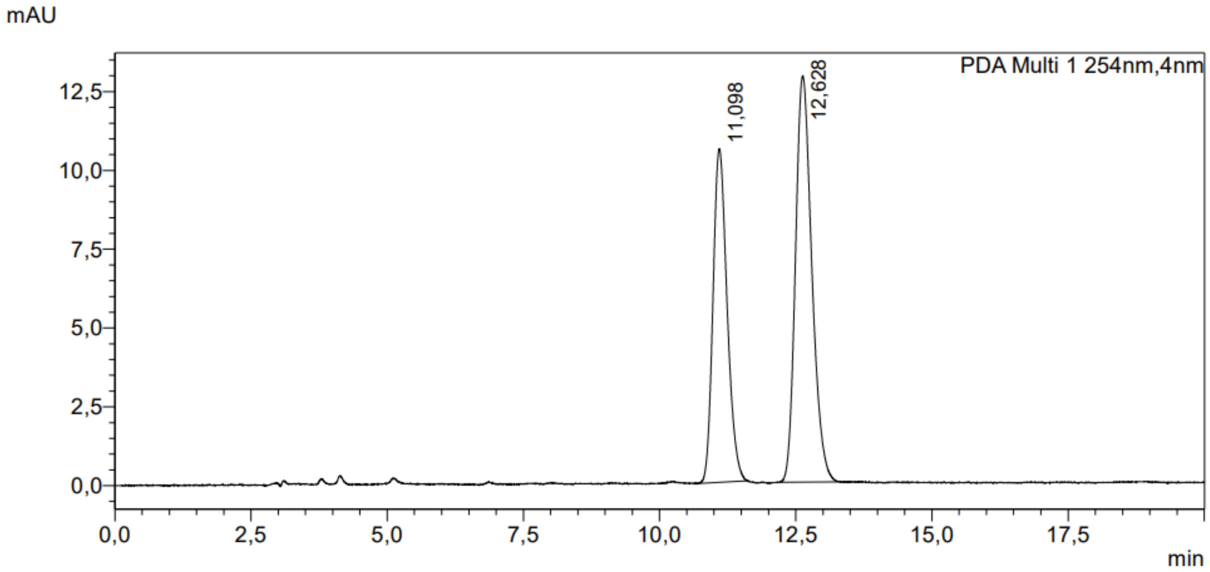

<Peak Table>

PDA Ch1 254nm

Peak#	Ret. Time	Area%
1	11,098	41,401
2	12,628	58,599
Total		100,000

^{1}H-NMR (400 MHz, toluene-d_8) of **5** (*rac*)

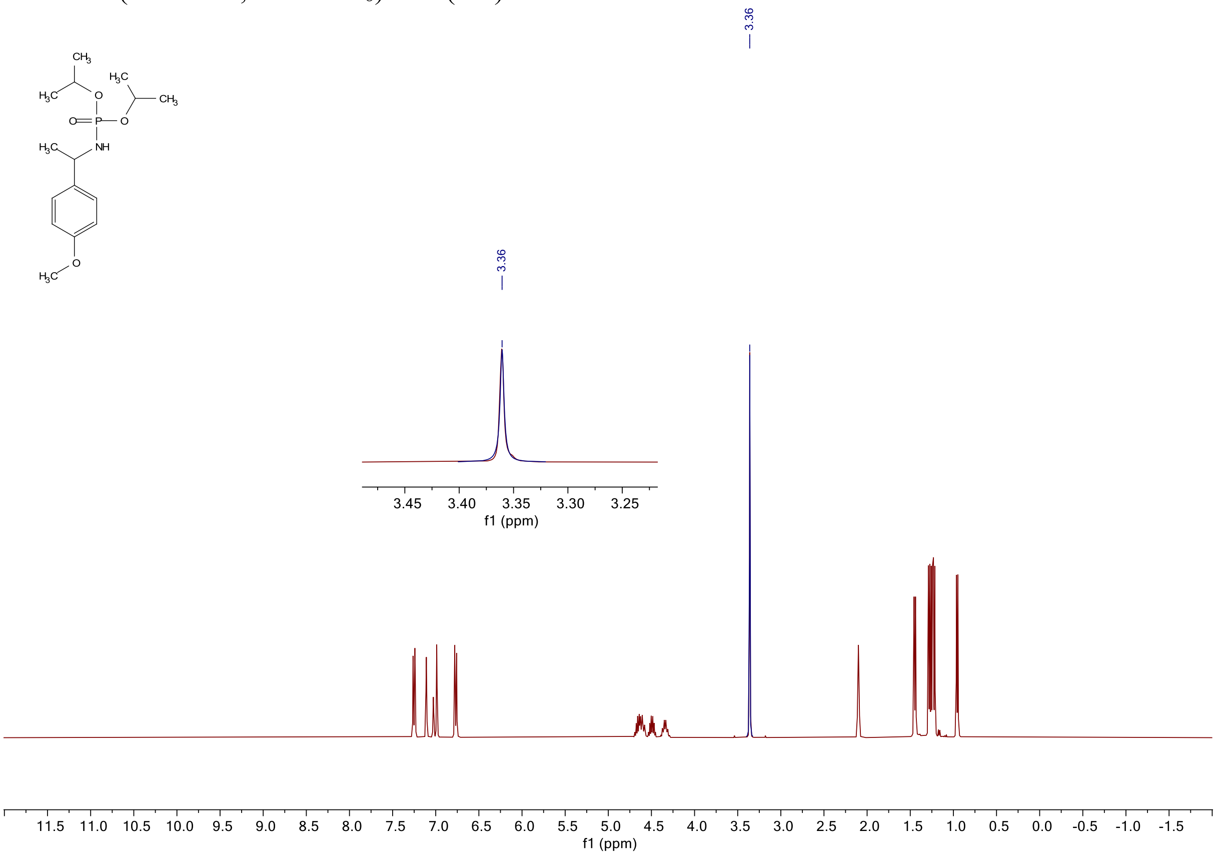

^{31}P-NMR (162 MHz, toluene-d_8) of **5** (*rac*)

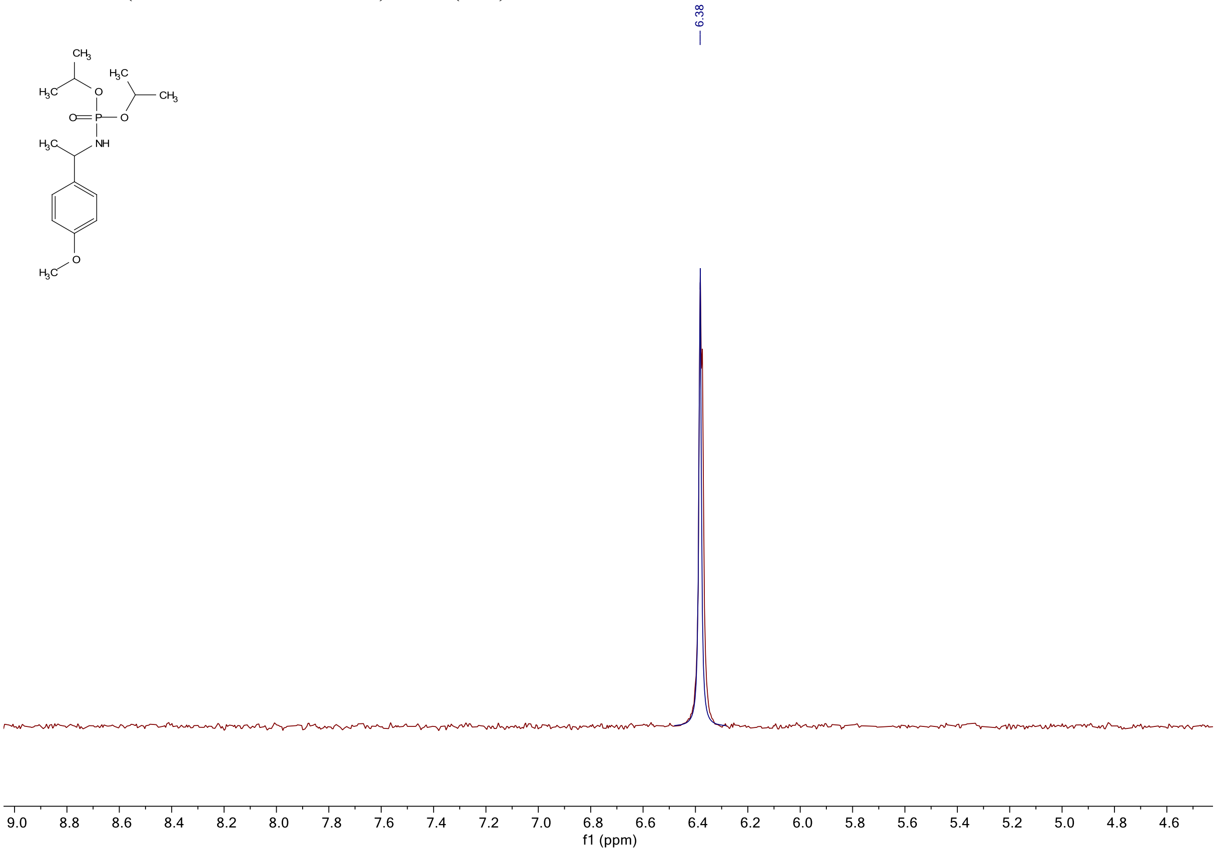

HPLC chromatogram of racemic **5**

Conditions: Chiralpak AD-H, *n*-heptane/^{i}PrOH 93-7 40 ºC, 1 mL/min, λ_{abs} = 254 nm

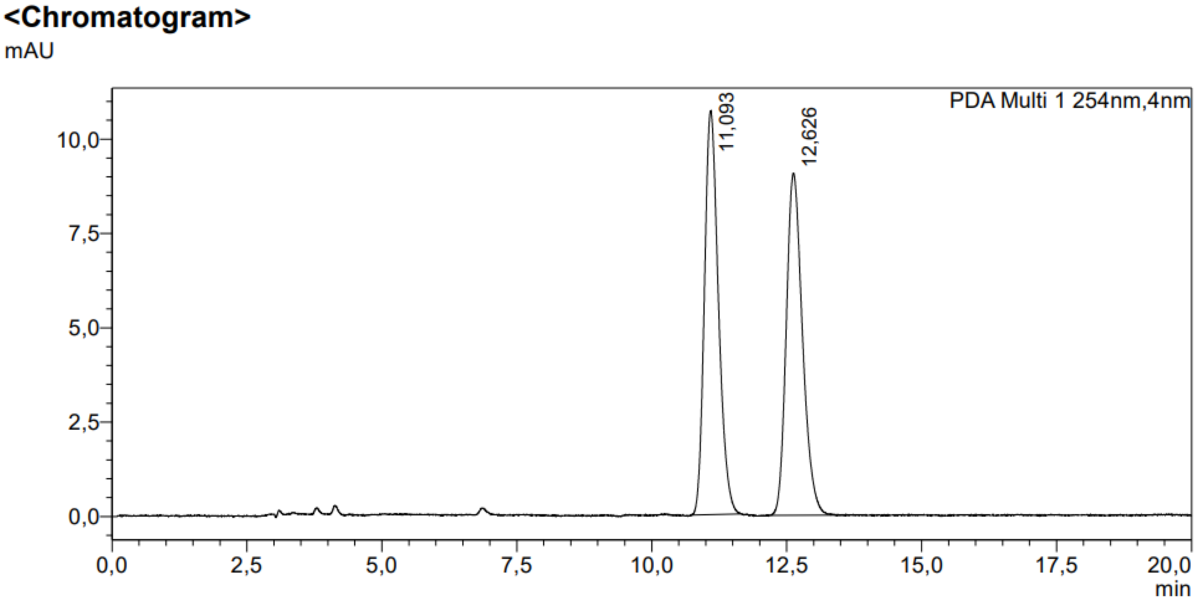

<Peak Table>

PDA Ch1 254nm

Peak#	Ret. Time	Area%
1	11,093	50,363
2	12,626	49,637
Total		100,000

^{1}H-NMR (400 MHz, toluene-d_8) of **5** (*e.r.* 60:40)

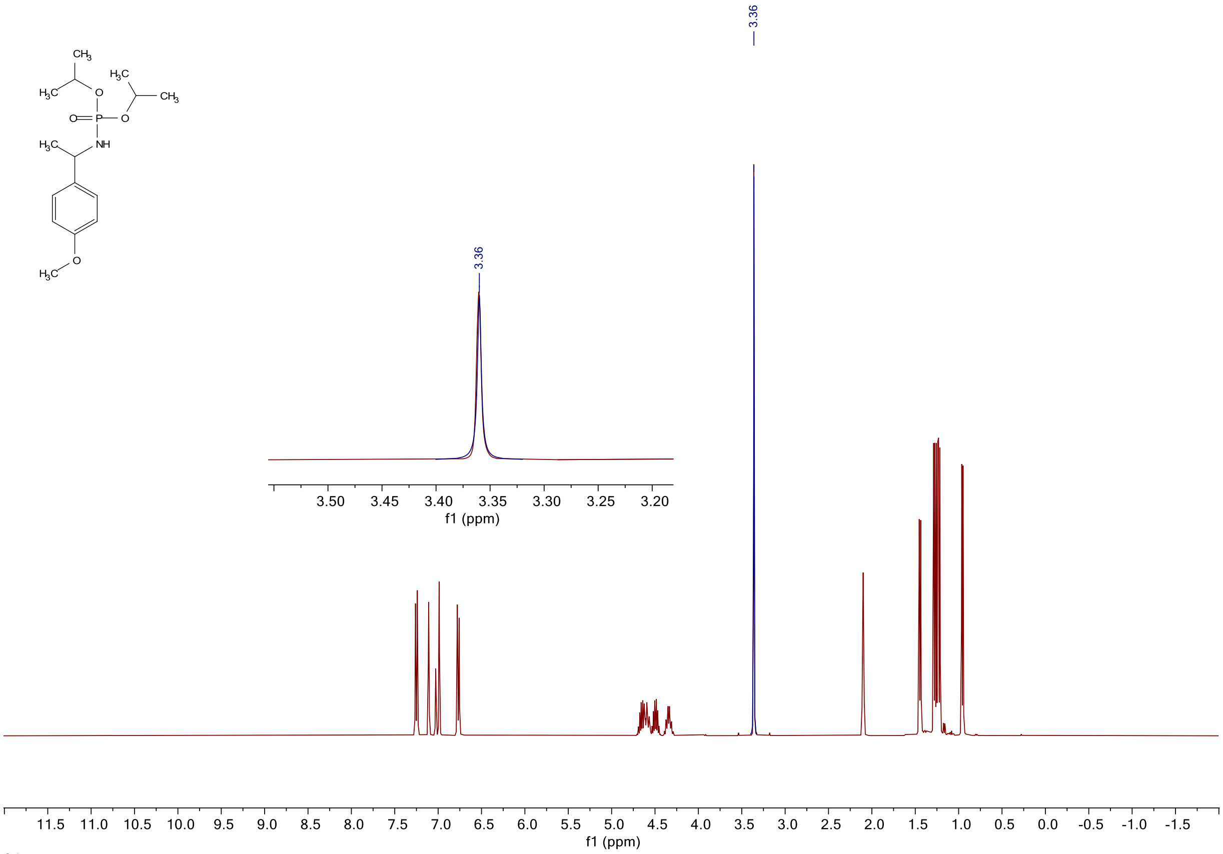

^{31}P-NMR (162 MHz, toluene-d_8) of **5** (*e.r.* 60:40)

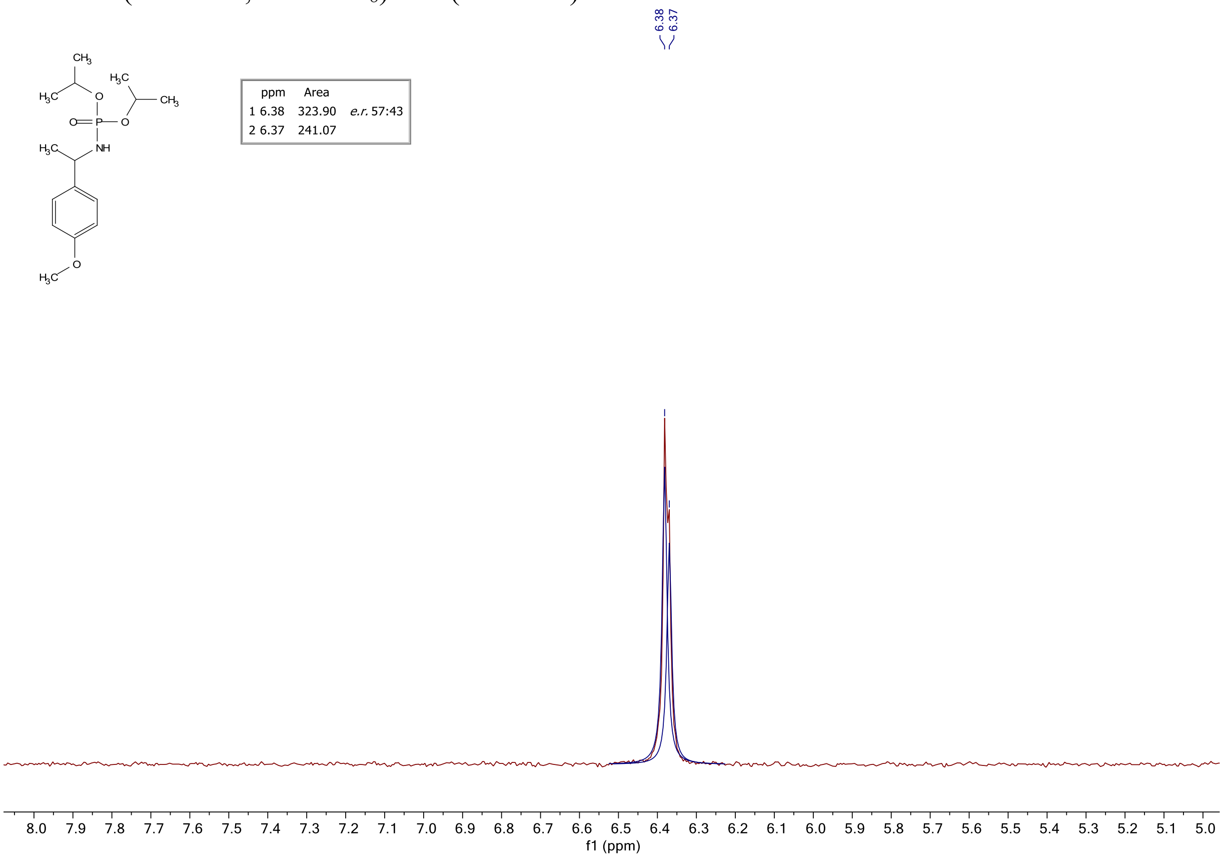

HPLC chromatogram of scalemic **5** (*e.r.* 60:40)
Conditions: Chiralpak AD-H, *n*-heptane/^{i}PrOH 93-7 40 °C, 1 mL/min, λ_{abs} = 254 nm

<Chromatogram>

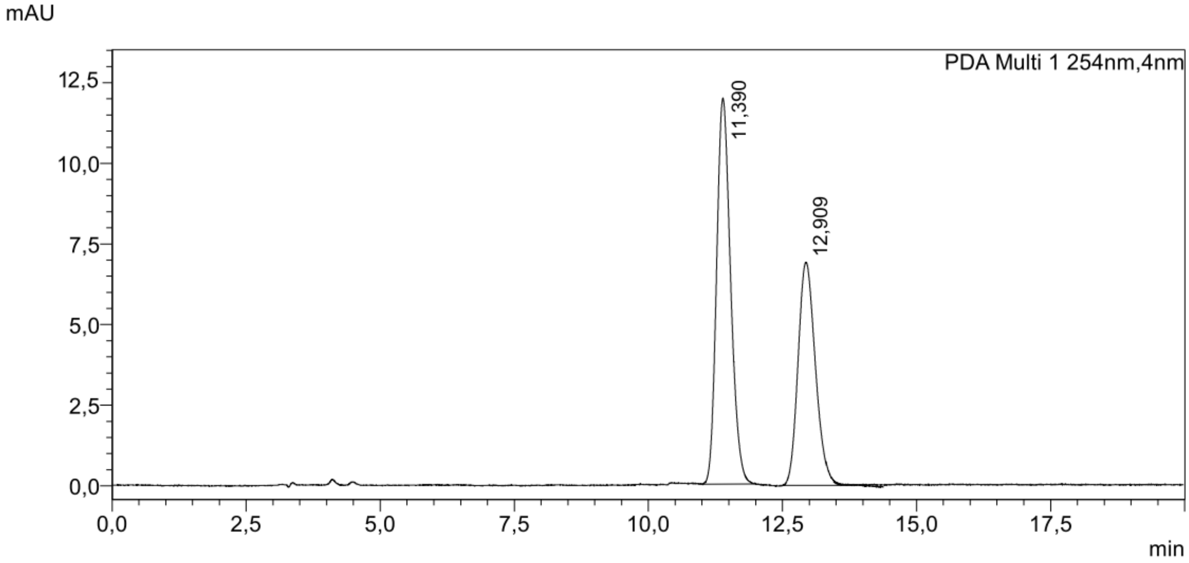

<Peak Table>
PDA Ch1 254nm

Peak#	Ret. Time	Area%
1	11,390	59,839
2	12,909	40,161
Total		100,000

^{1}H-NMR (400 MHz, toluene-d_8) of **5** (*e.r.* 70:30)

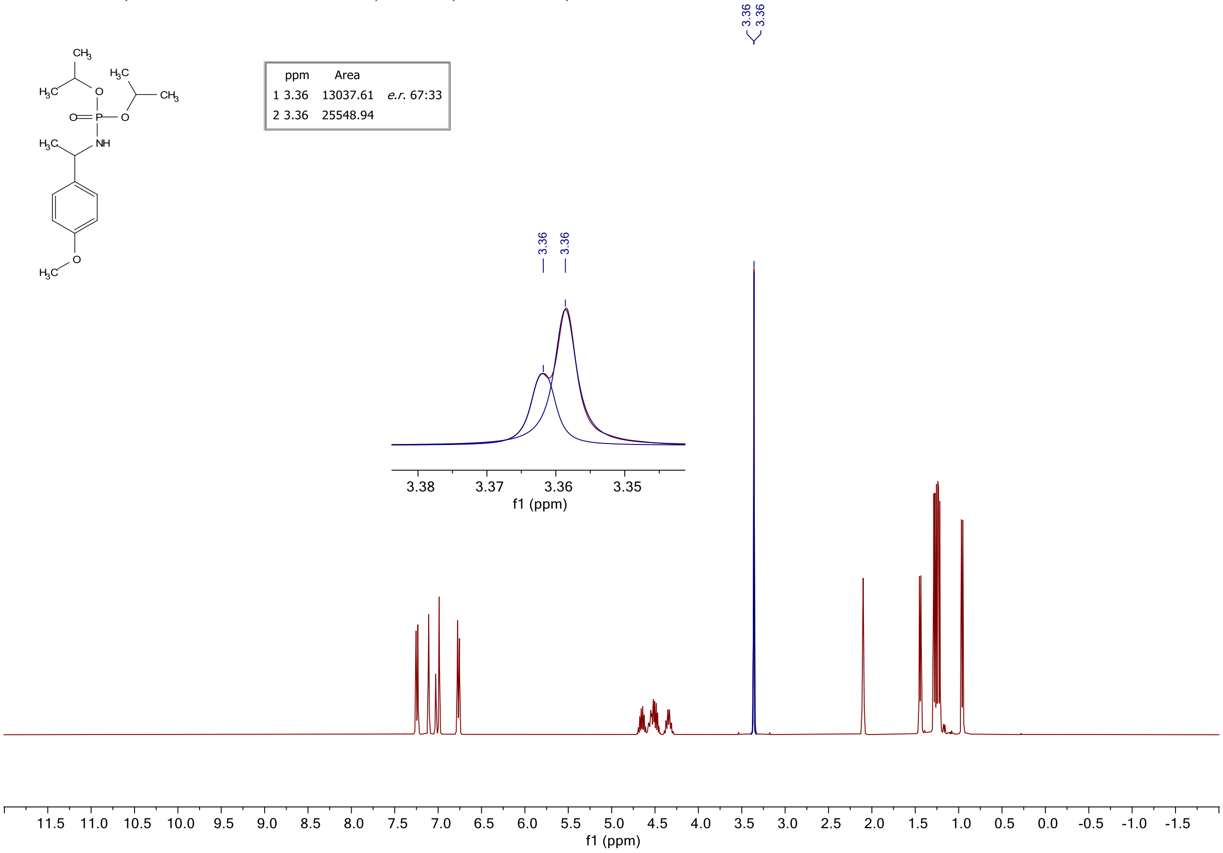

^{31}P-NMR (162 MHz, toluene-d_8) of **5** (*e.r.* 70:30)

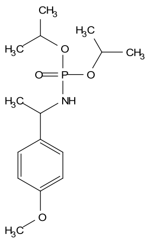

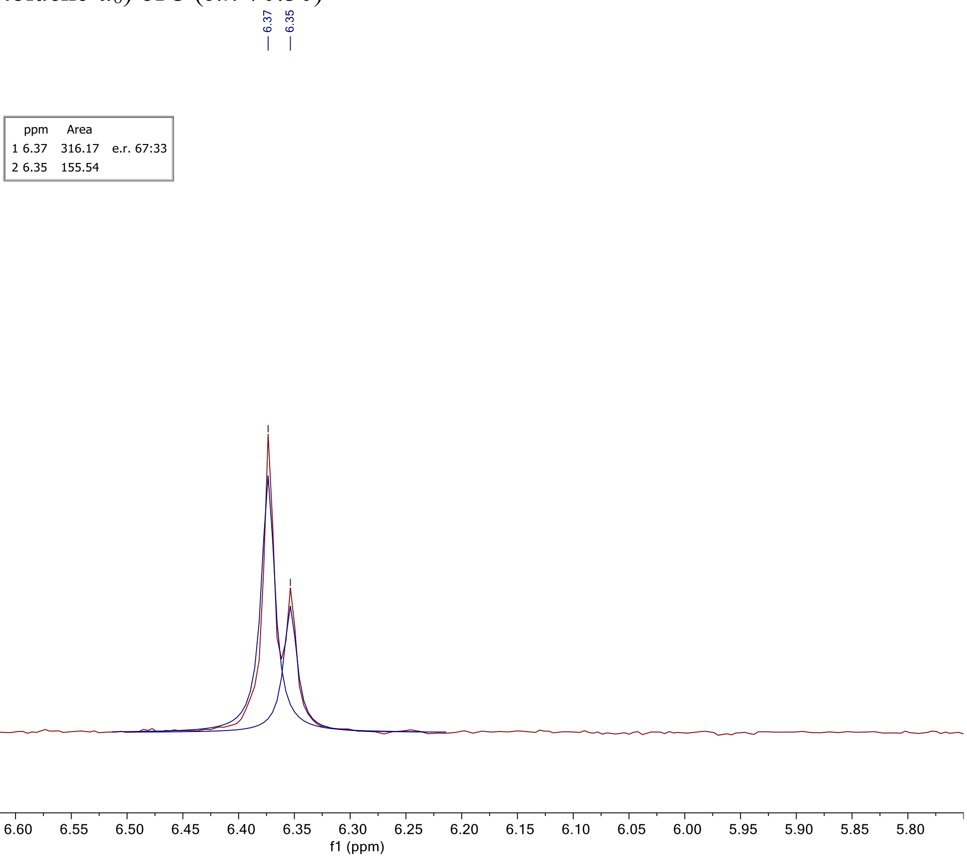

HPLC chromatogram of scalemic **5** (*e.r.* 70:30)

Conditions: Chiralpak AD-H, *n*-heptane/^{i}PrOH 93-7 40 °C, 1 mL/min, λ_{abs} = 254 nm

<Chromatogram>

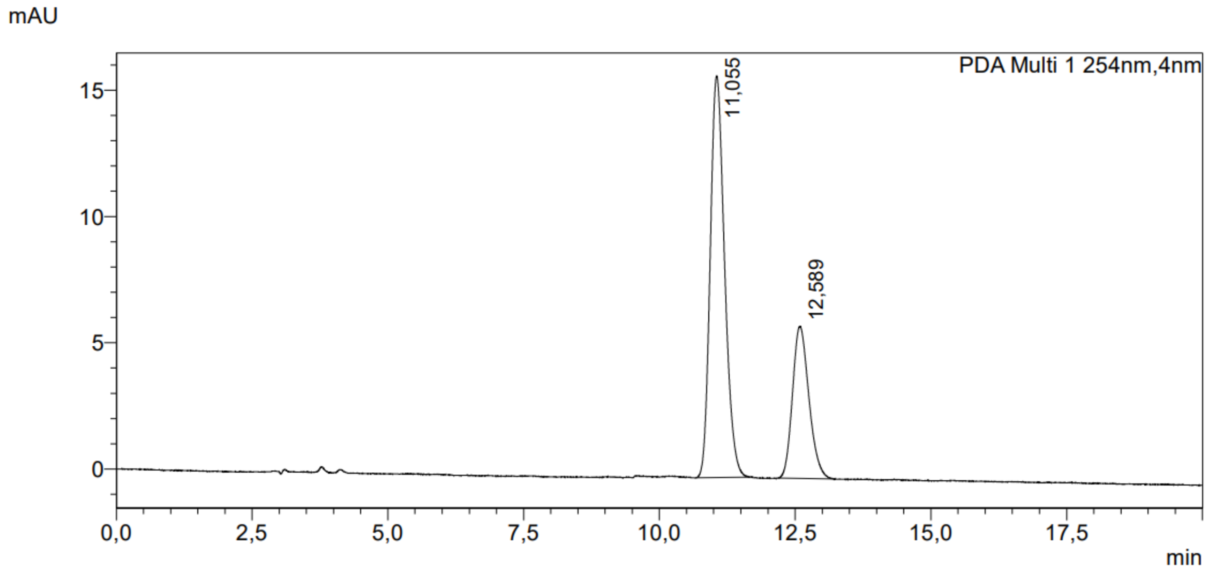

<Peak Table>

PDA Ch1 254nm

Peak#	Ret. Time	Area%
1	11,055	69,786
2	12,589	30,214
Total		100,000

^{1}H-NMR (400 MHz, toluene-d_8) of **5** (*e.r.* 80:20)

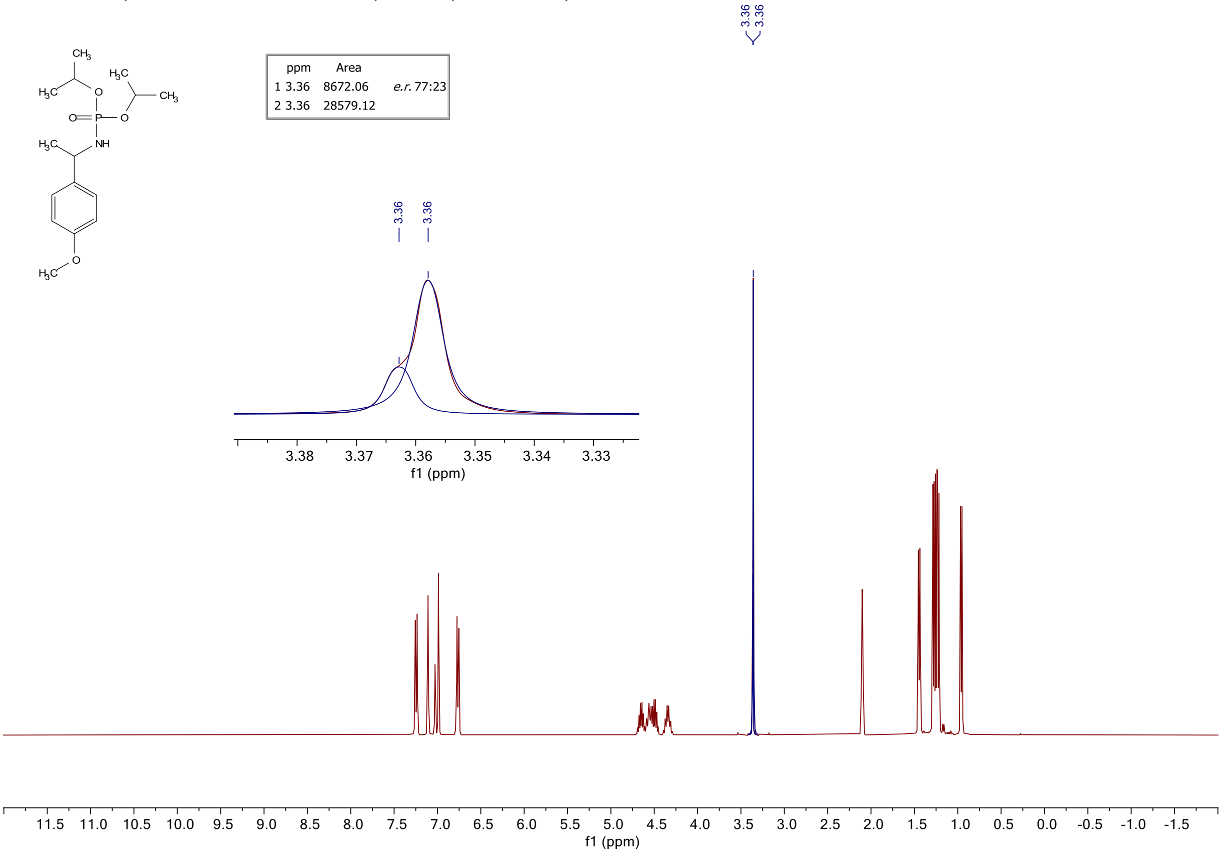

^{31}P-NMR (162 MHz, toluene-d_8) of **5** (*e.r.* 80:20)

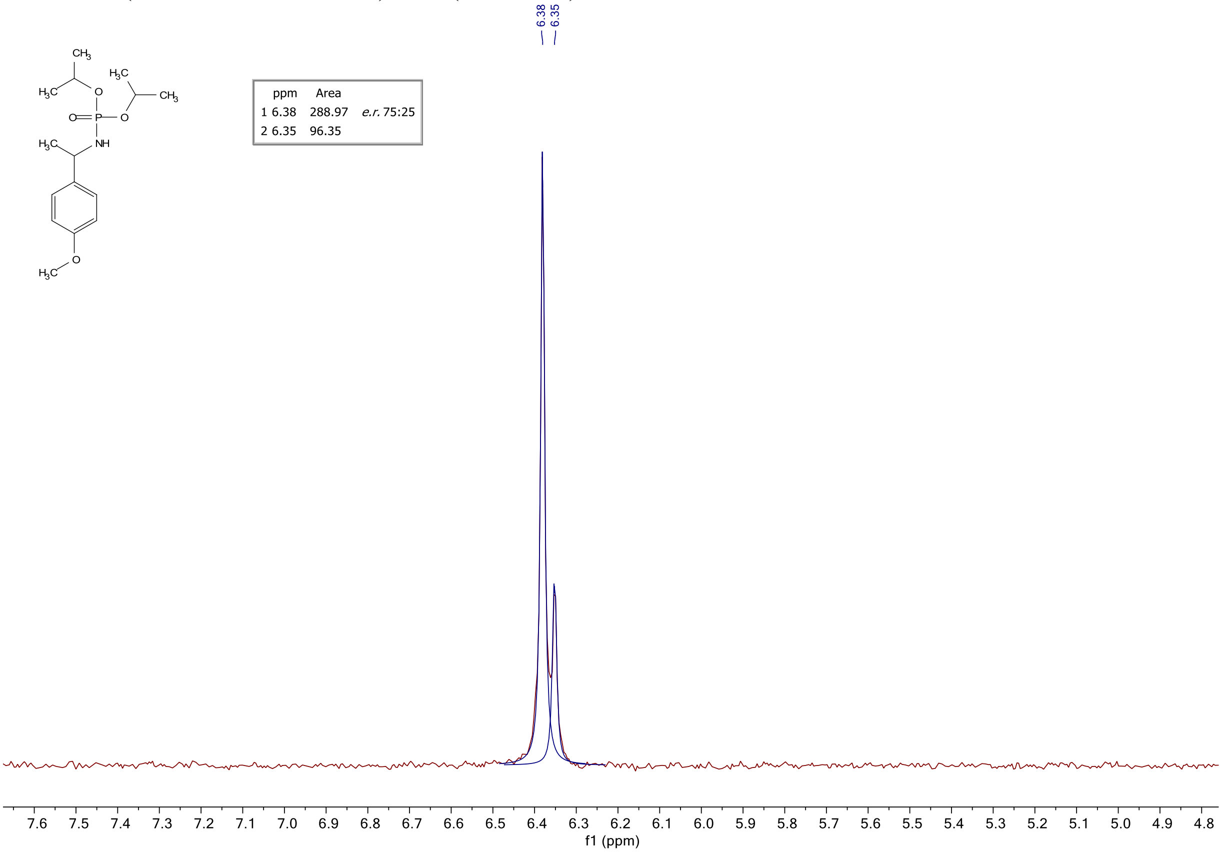

HPLC chromatogram of scalemic **5** (*e.r.* 80:20)

Conditions: Chiralpak AD-H, *n*-heptane/^{i}PrOH 93-7 40 °C, 1 mL/min, λ_{abs} = 254 nm

<Chromatogram>

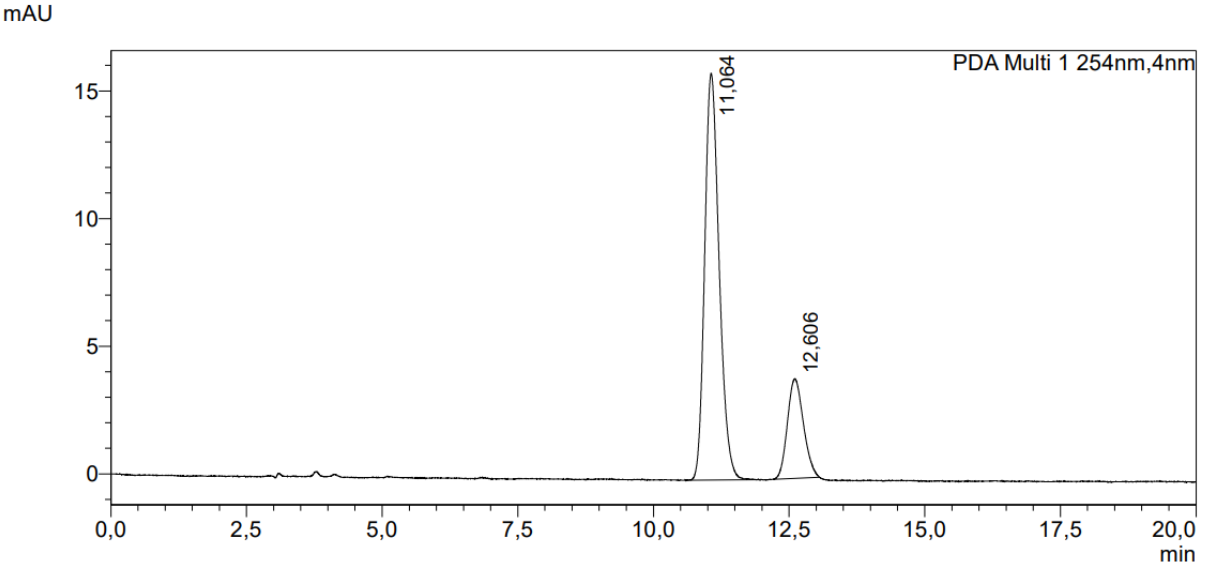

<Peak Table>

PDA Ch1 254nm

Peak#	Ret. Time	Area%
1	11,064	78,709
2	12,606	21,291
Total		100,000

^{1}H-NMR (400 MHz, toluene-d_8) of **5** (*e.r.* 90:10)

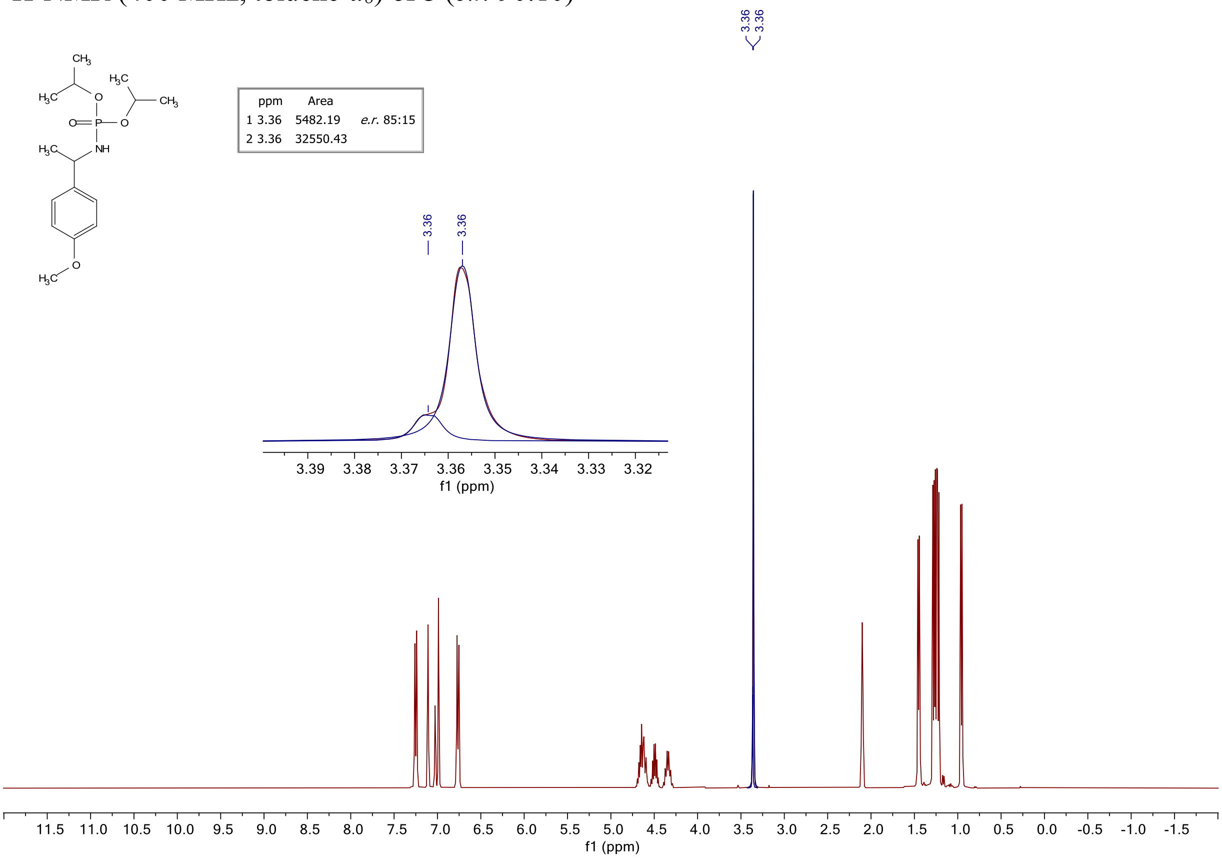

^{31}P-NMR (162 MHz, toluene-d_8) of **5** (*e.r.* 90:10)

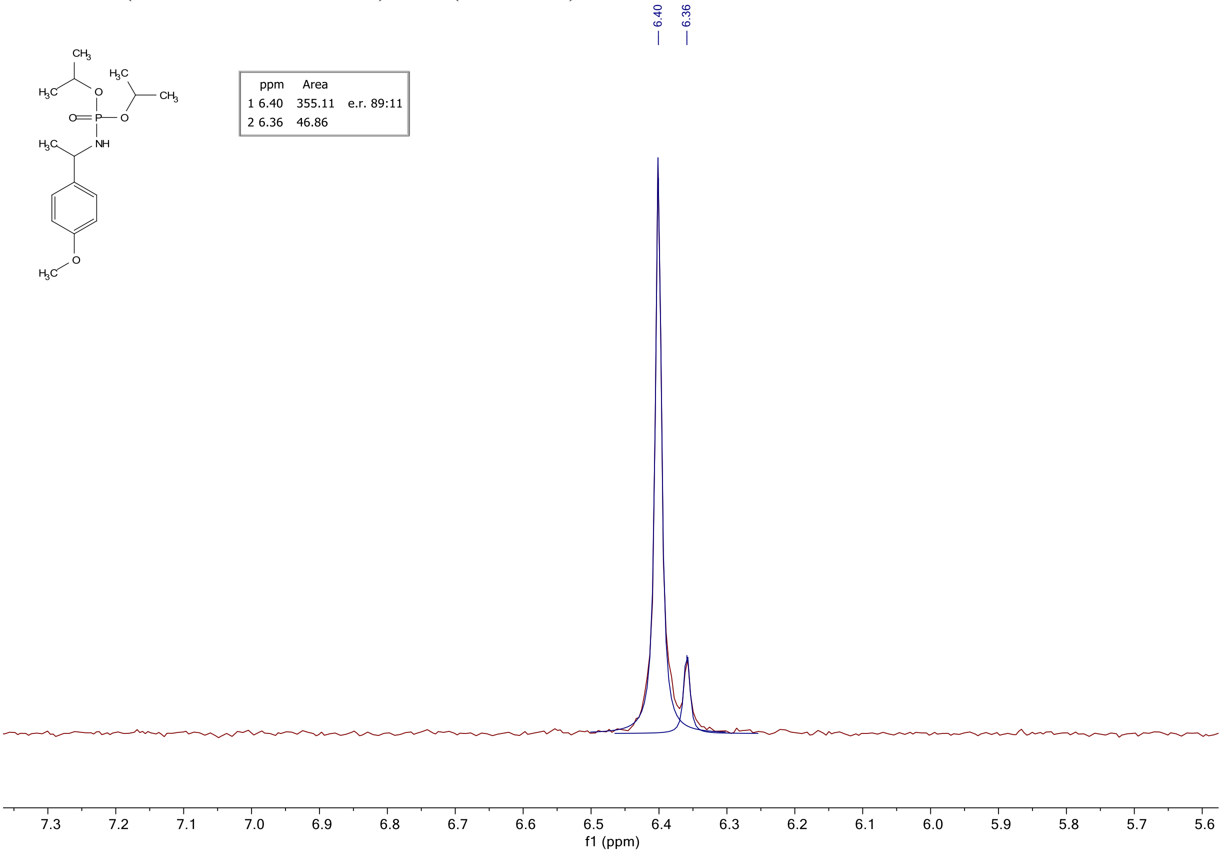

HPLC chromatogram of scalemic **5** (*e.r.* 90:10)
Conditions: Chiralpak AD-H, *n*-heptane/^{i}PrOH 93-7 40 ºC, 1 mL/min, λ_{abs} = 254 nm

<Chromatogram>

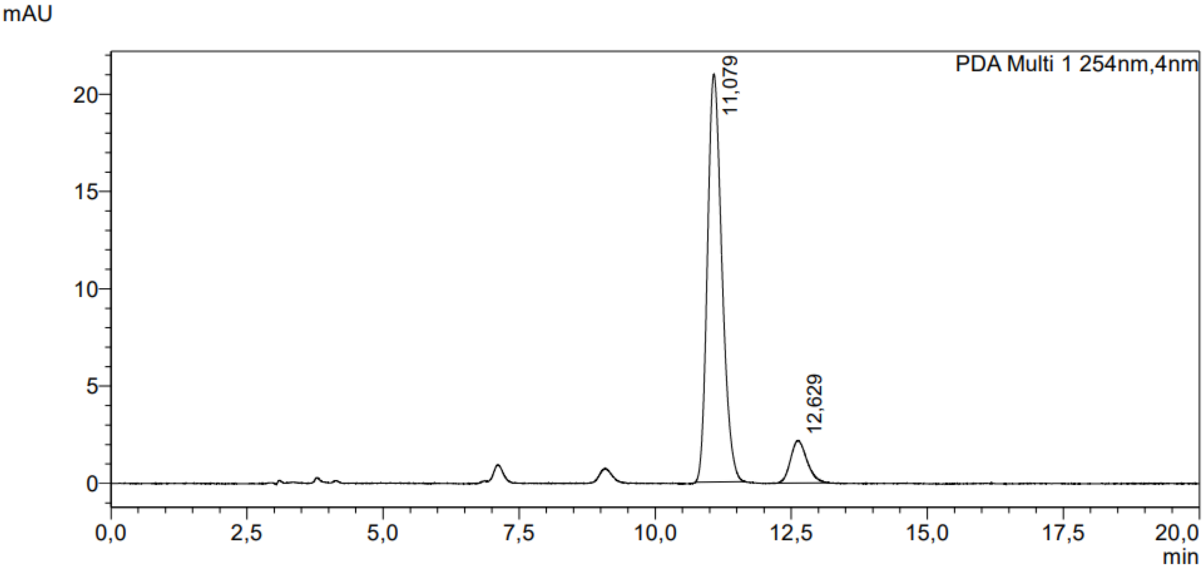

<Peak Table>

PDA Ch1 254nm

Peak#	Ret. Time	Area%
1	11,079	89,446
2	12,629	10,554
Total		100,000

^{31}P-NMR (162 MHz, $CDCl_3$) of **1b**

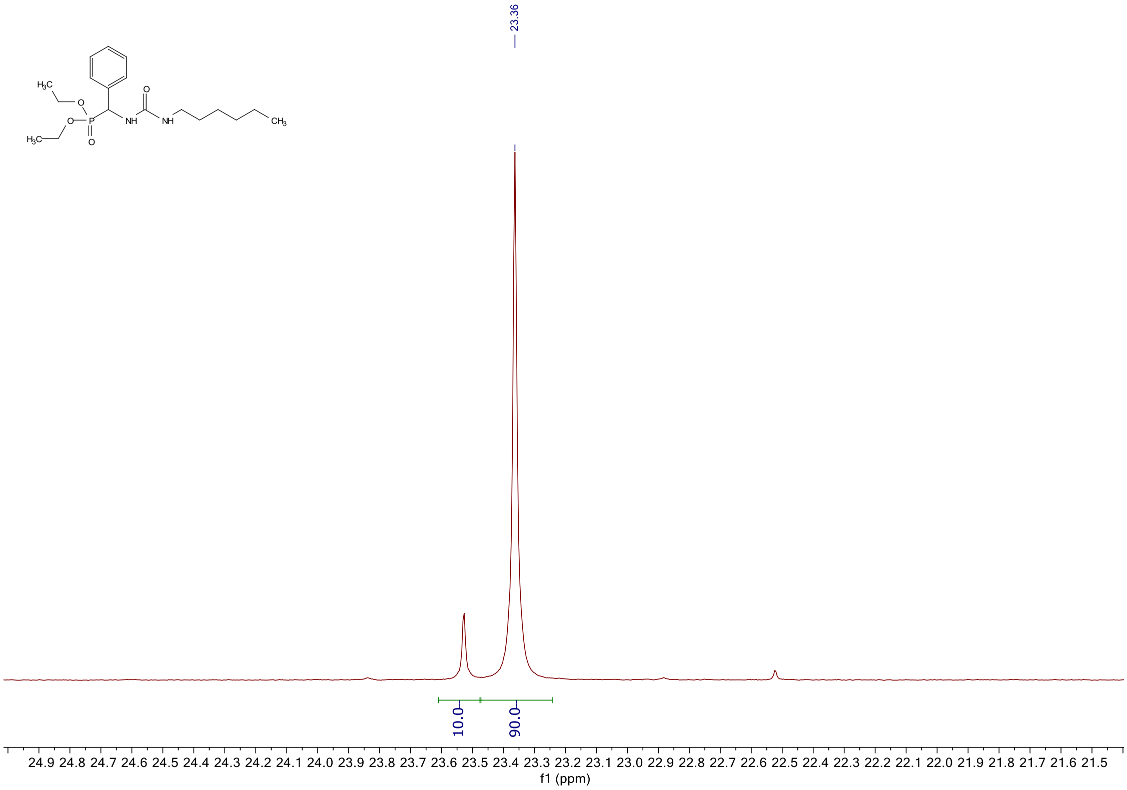

^{31}P-NMR (162 MHz, $CDCl_3$) of **1d**

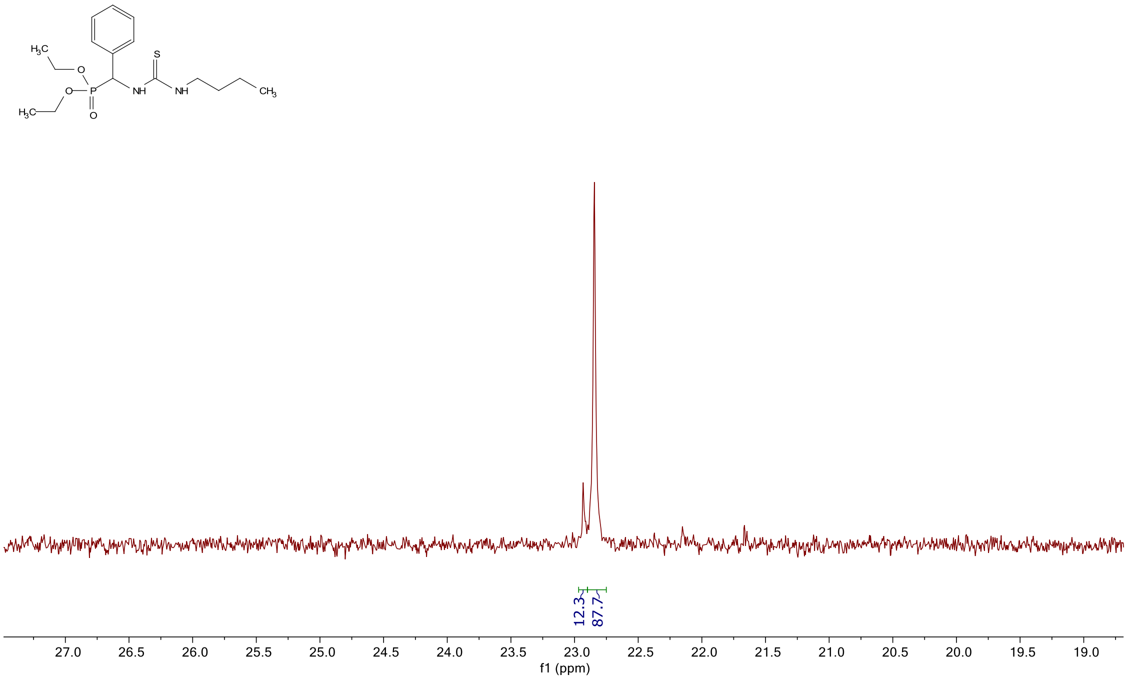

^{31}P-NMR (162 MHz, $CDCl_3$) of **1g**

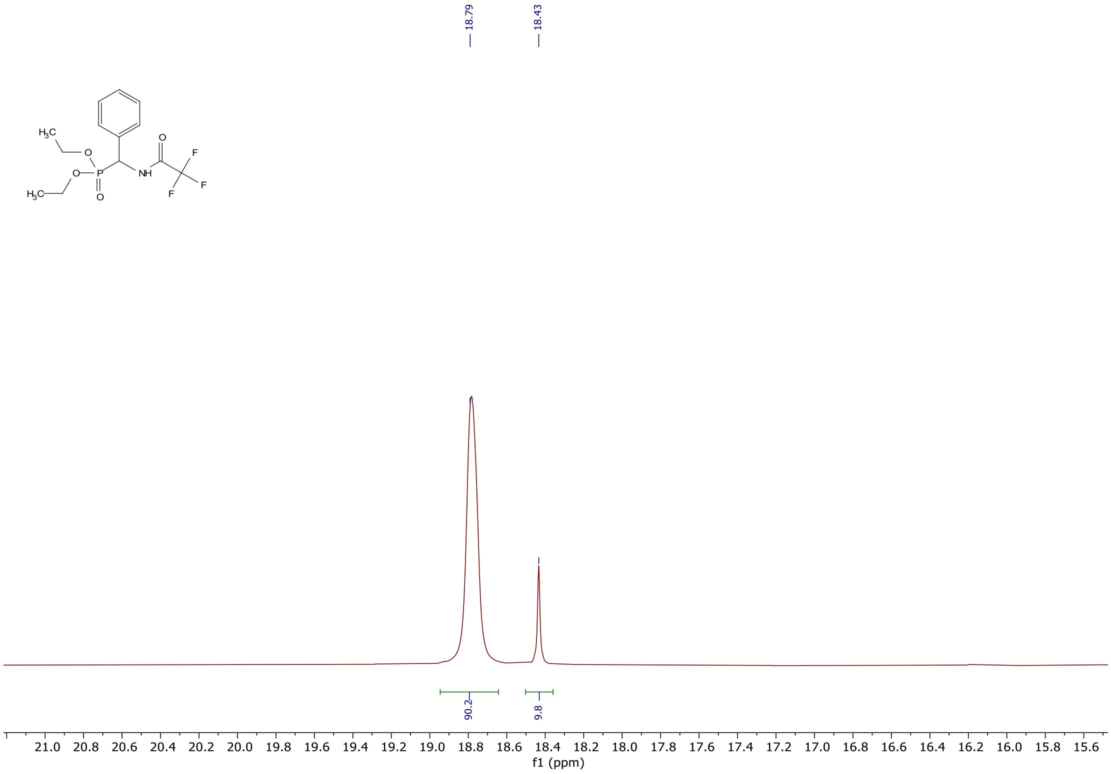

^{1}H-NMR (400 MHz, $CDCl_3$) of **1h**

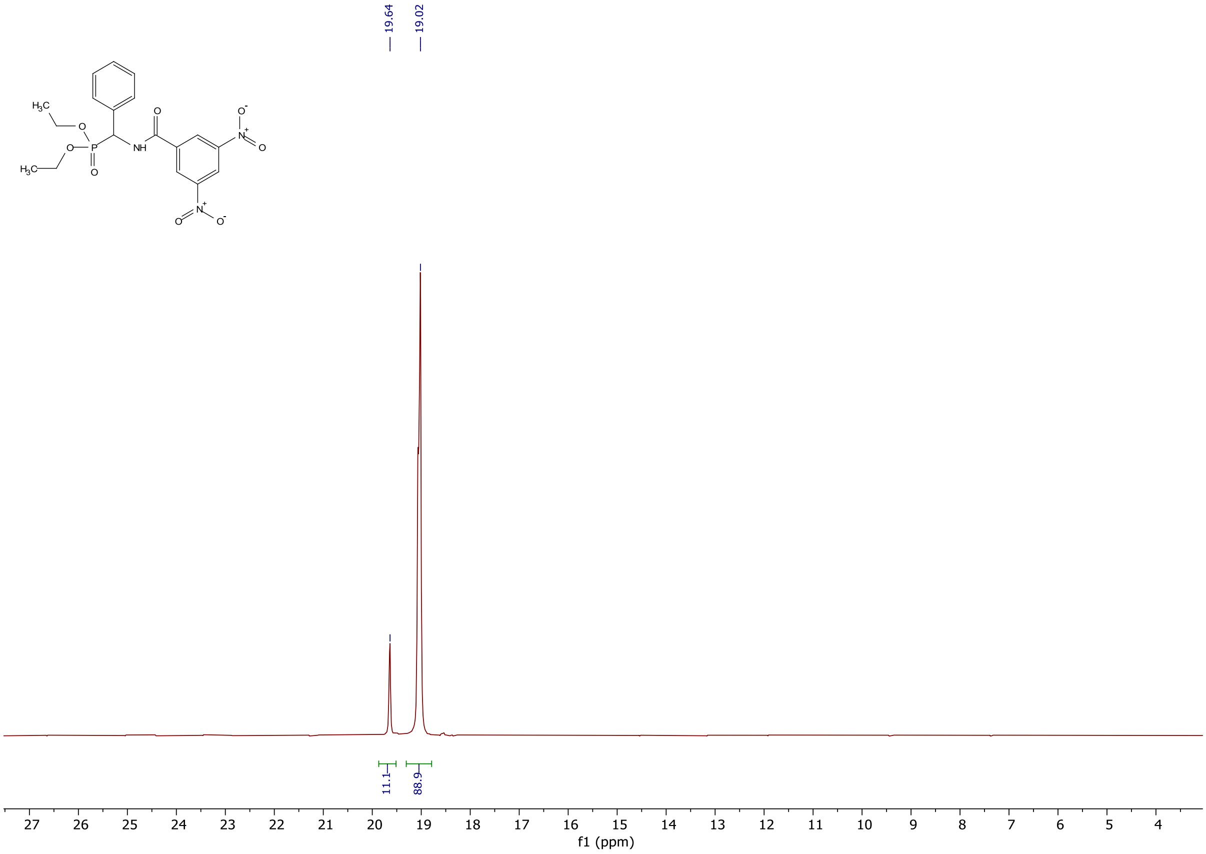

^{1}H-NMR (400 MHz, $CDCl_3$) of **2a**

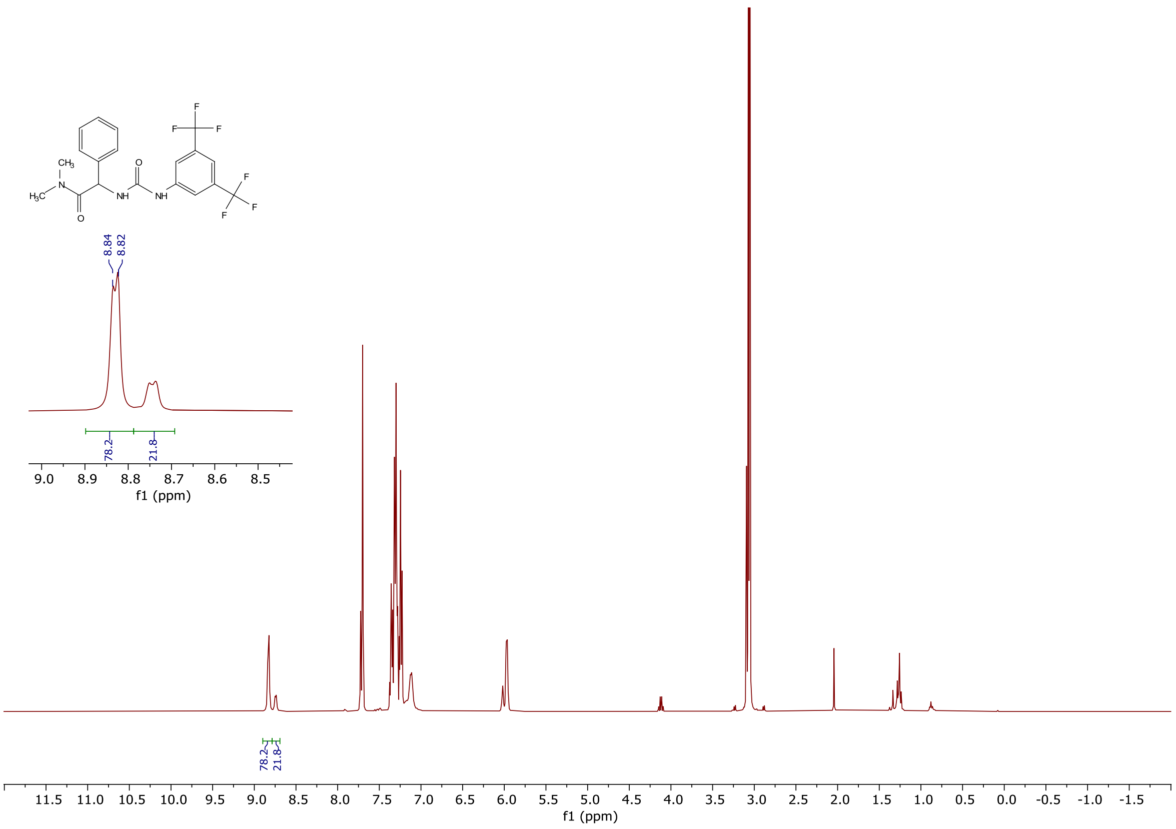

^{1}H-NMR (400 MHz, $CDCl_3$) of **2b**

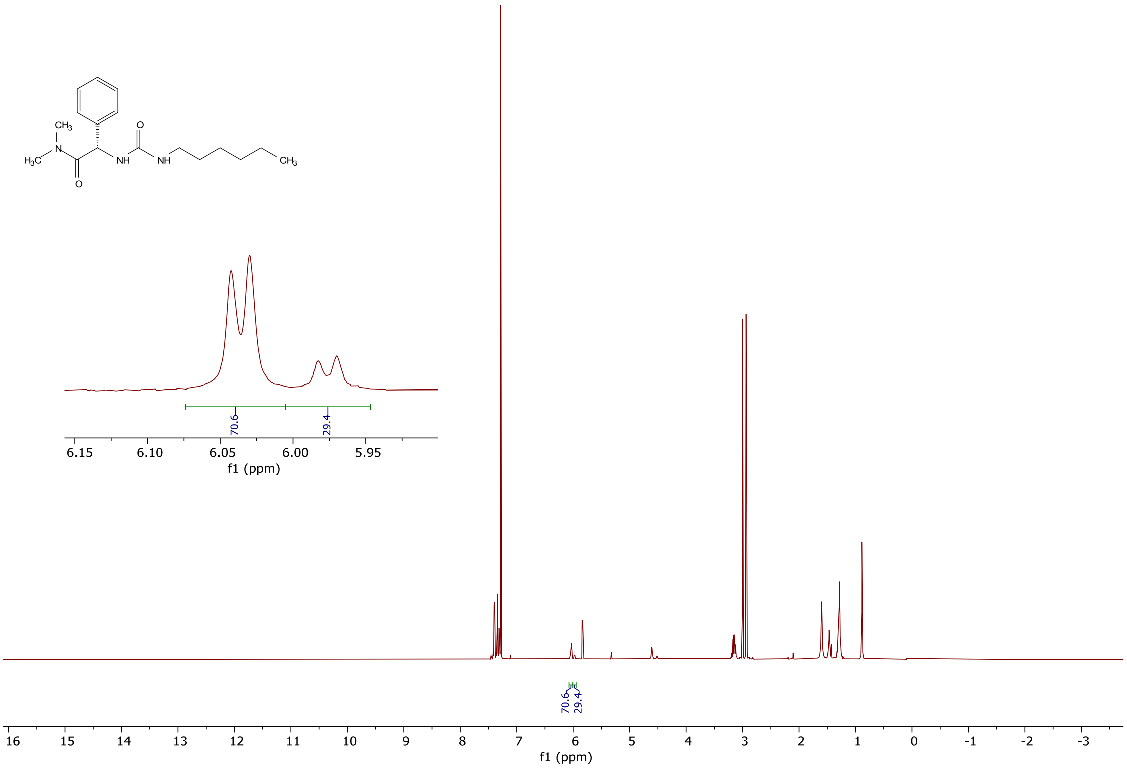

^{1}H-NMR (400 MHz, $CDCl_3$) of **2c**

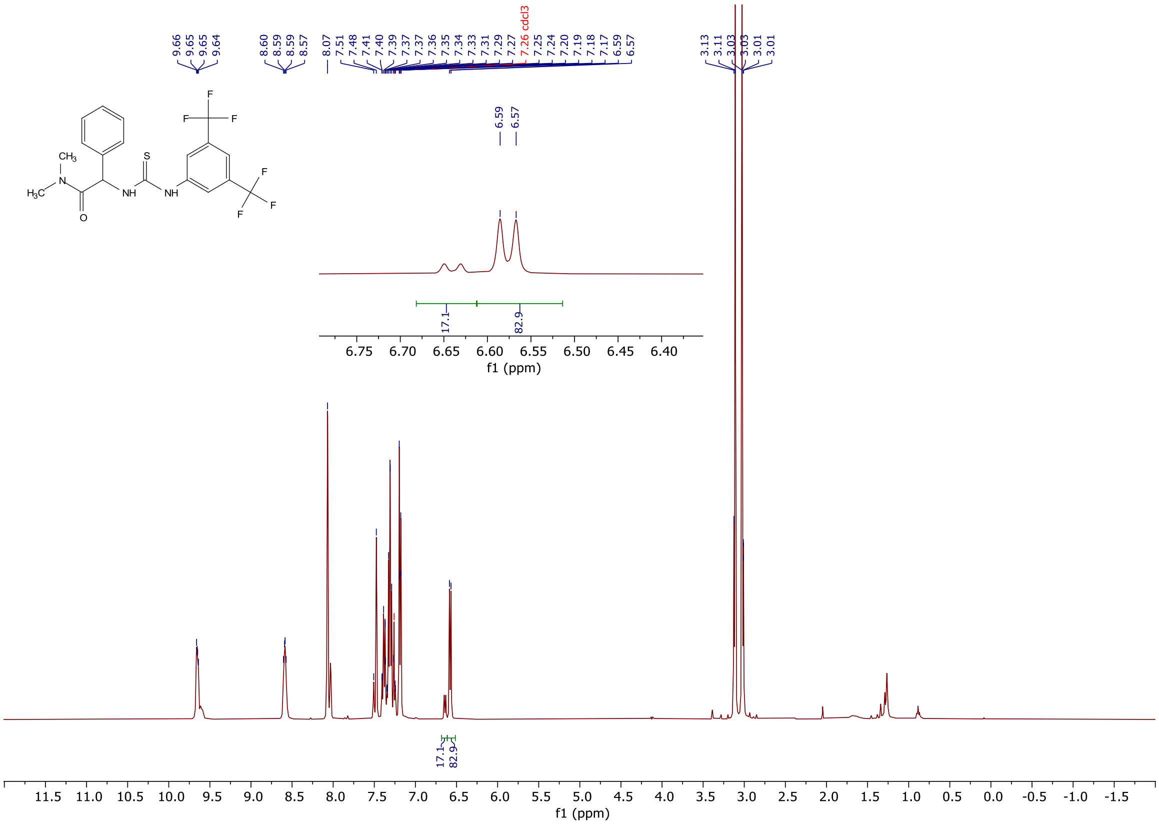

^{1}H-NMR (400 MHz, toluene-d_8.) of **2d**

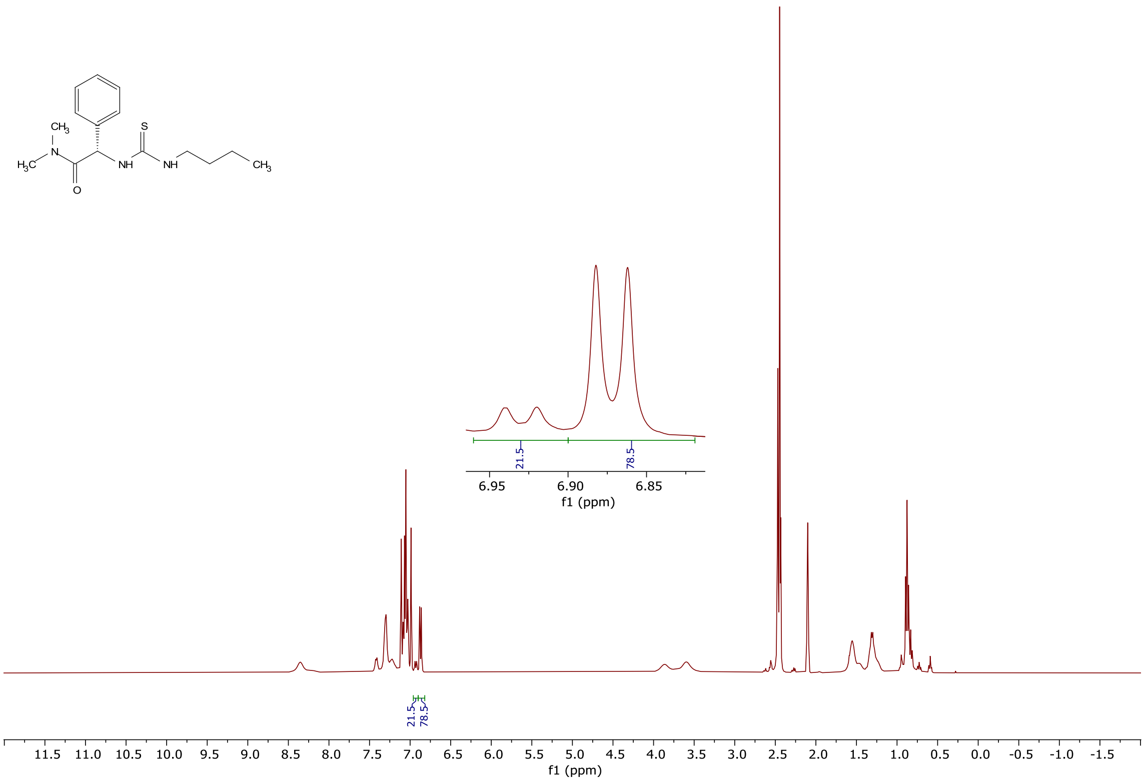

^{19}F-NMR (565 MHz, toluene-d_8) of **2g**

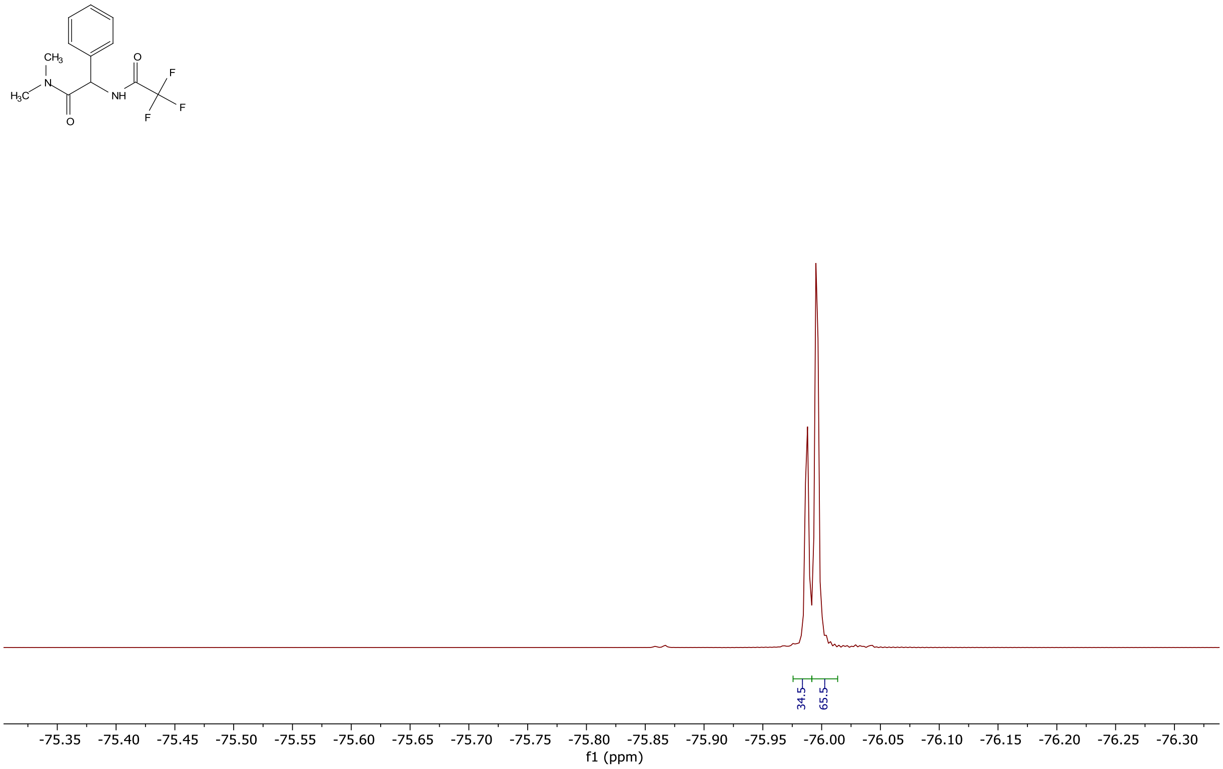

^{1}H-NMR (400 MHz, $CDCl_3$) of **2h**

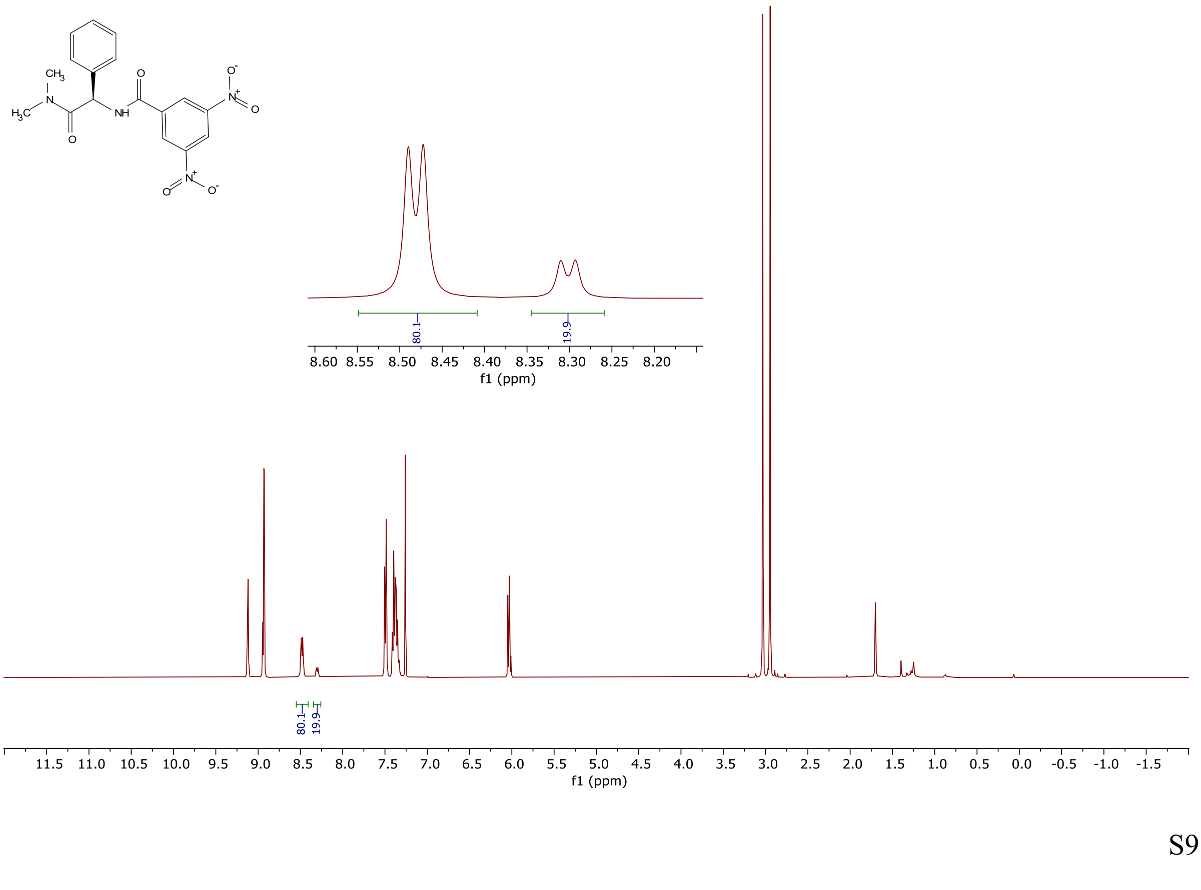

^{1}H-NMR (600 MHz, $CDCl_3$) of **3aa**

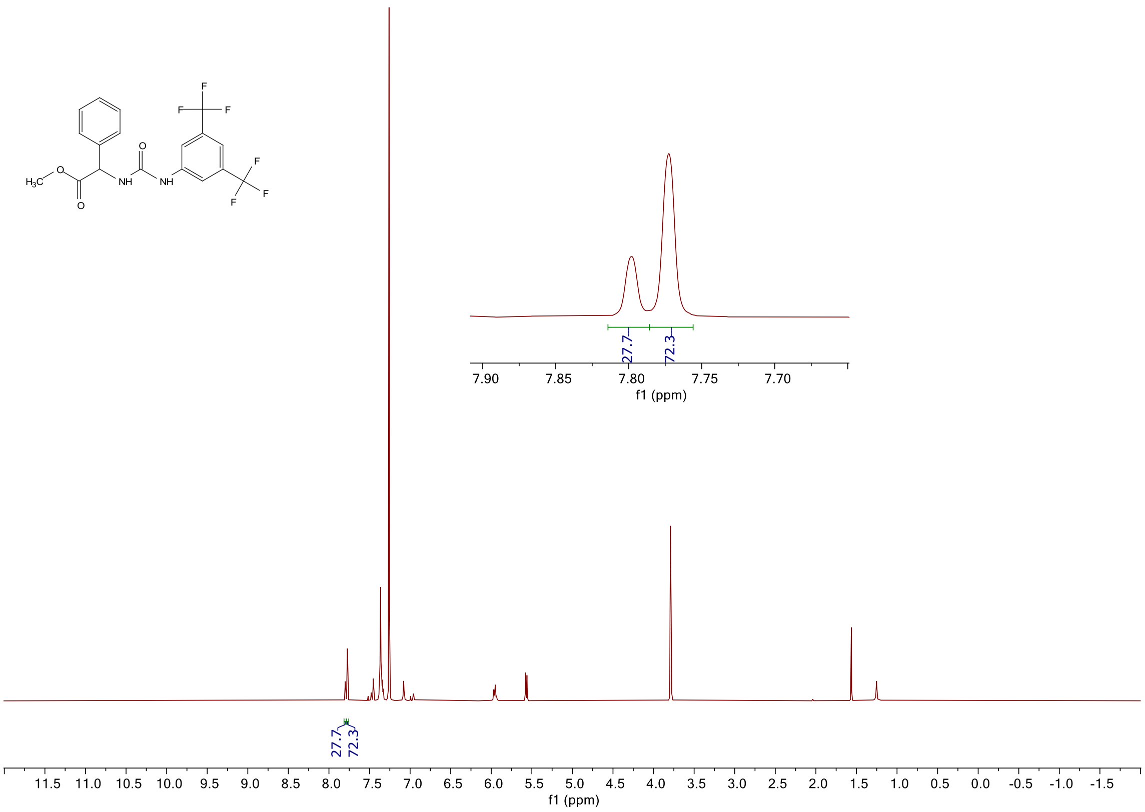

^{1}H-NMR (600 MHz, $CDCl_3$) of **3ab**

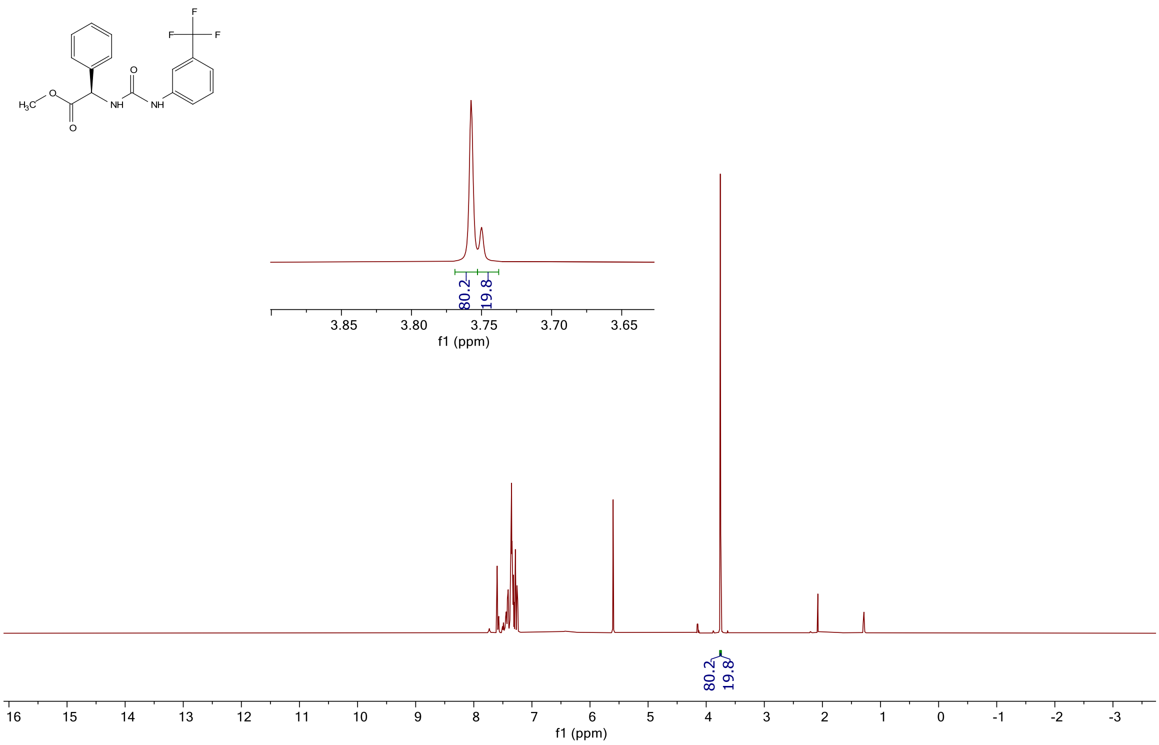

^{19}F-NMR (565 MHz, toluene-d_8) of **3ac**

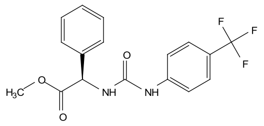

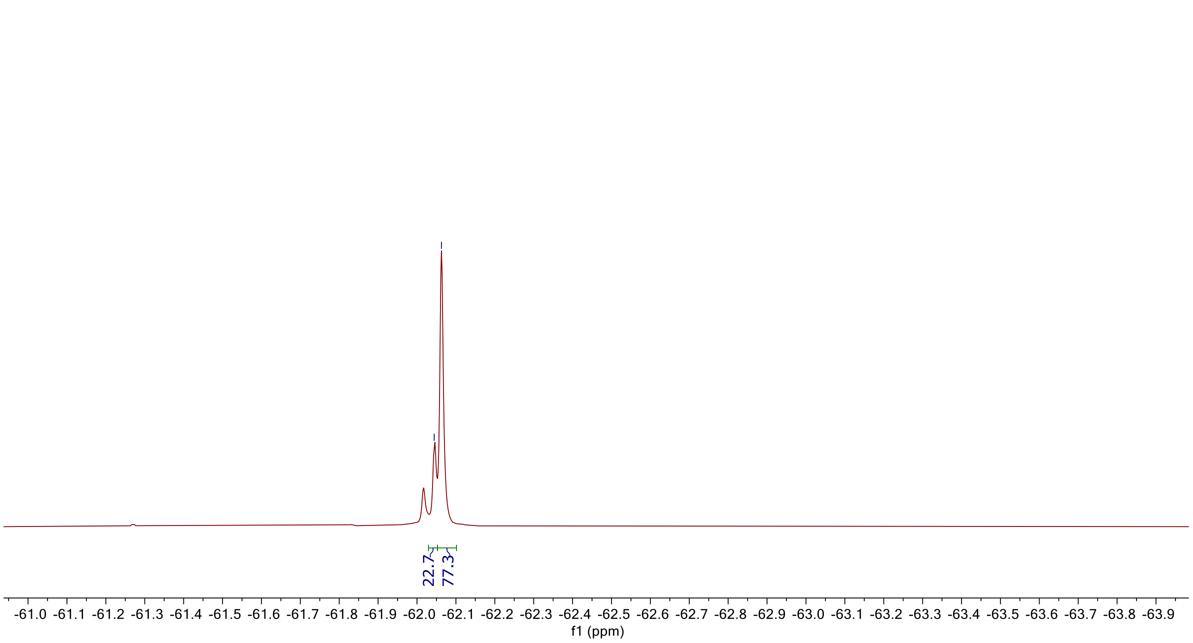

^{1}H-NMR (400 MHz, toluene-d_8) of **3b**

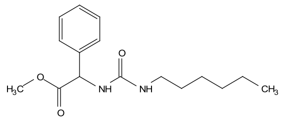

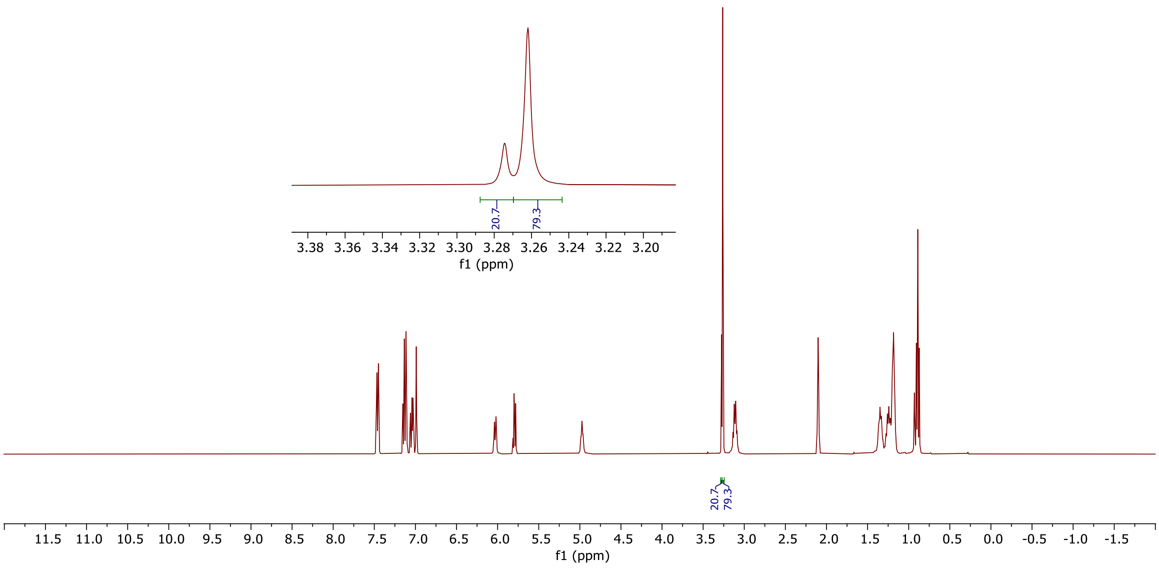

^{1}H-NMR (400 MHz, toluene-d_8) of **3h**

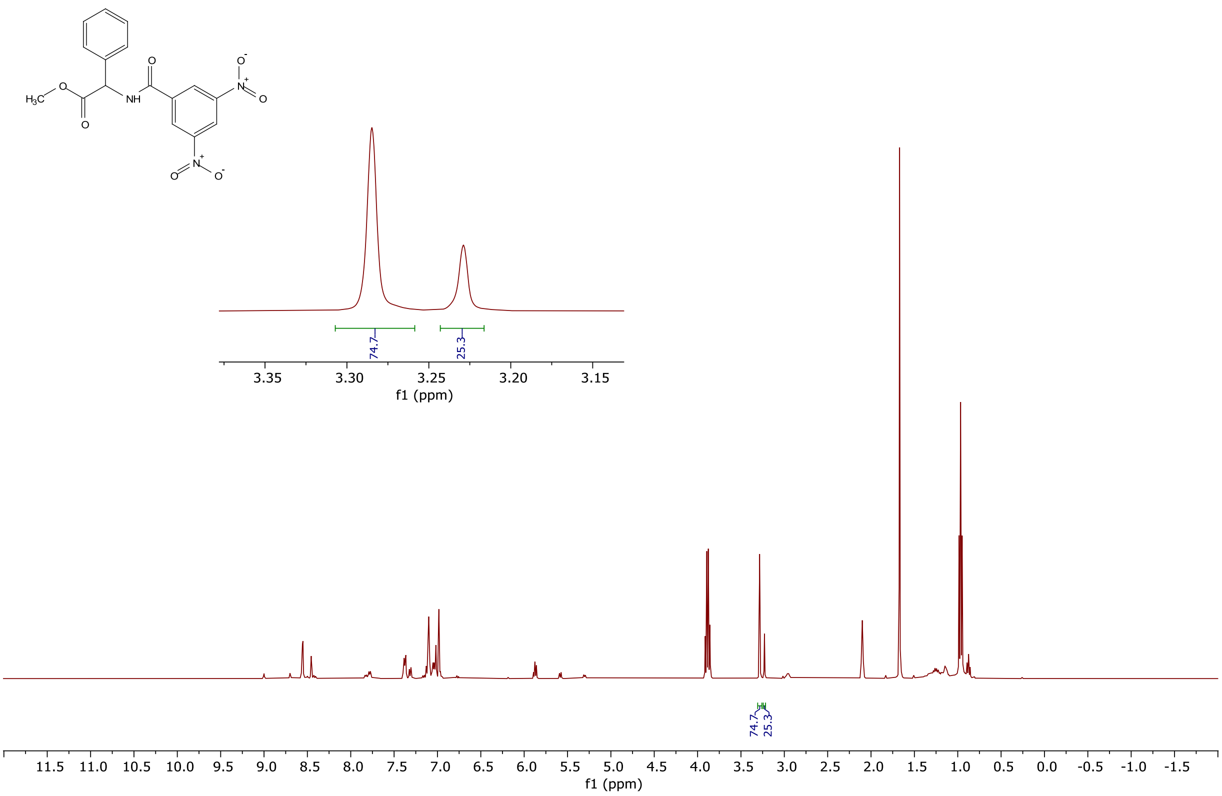

^{31}P-NMR (162 MHz, toluene-d_8) of **4e**

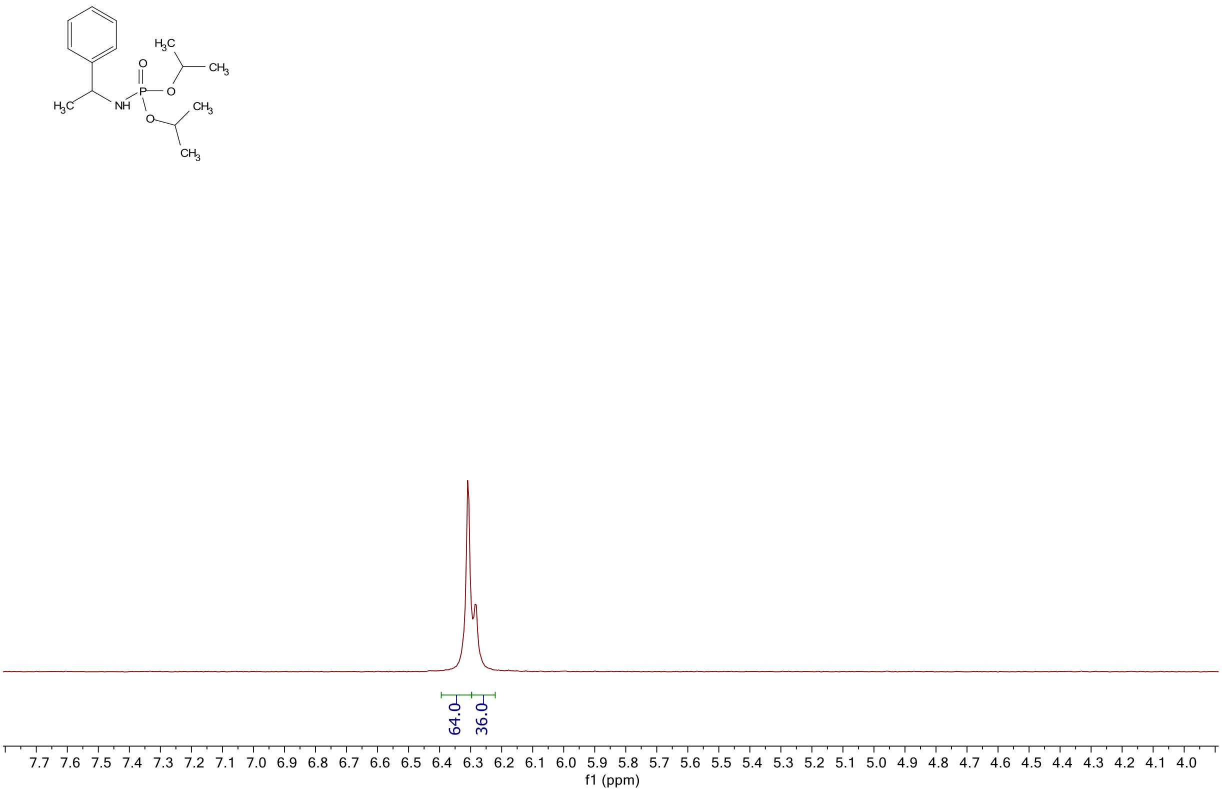

^{1}H-NMR (600 MHz, $CDCl_3$) of **11**

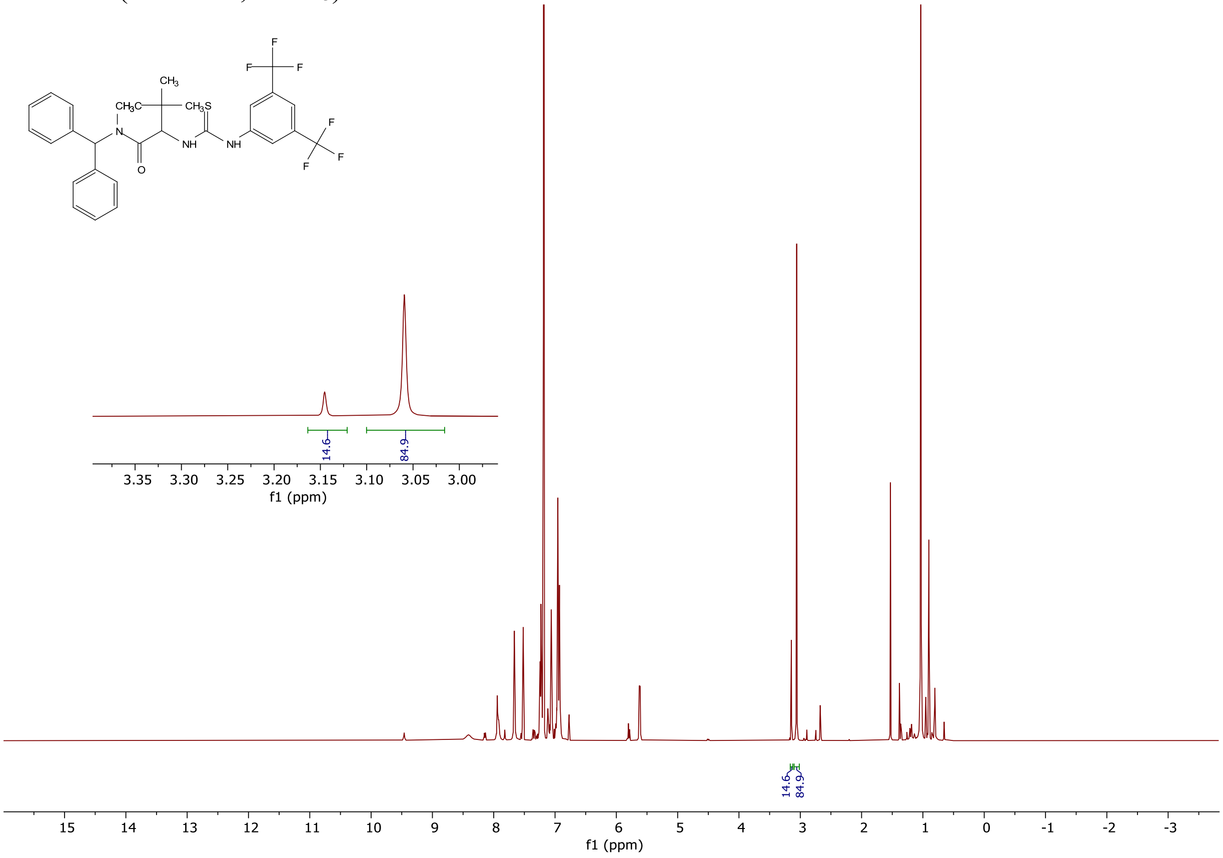

^{1}H NMR (600 MHz, C_6D_6) of **13**

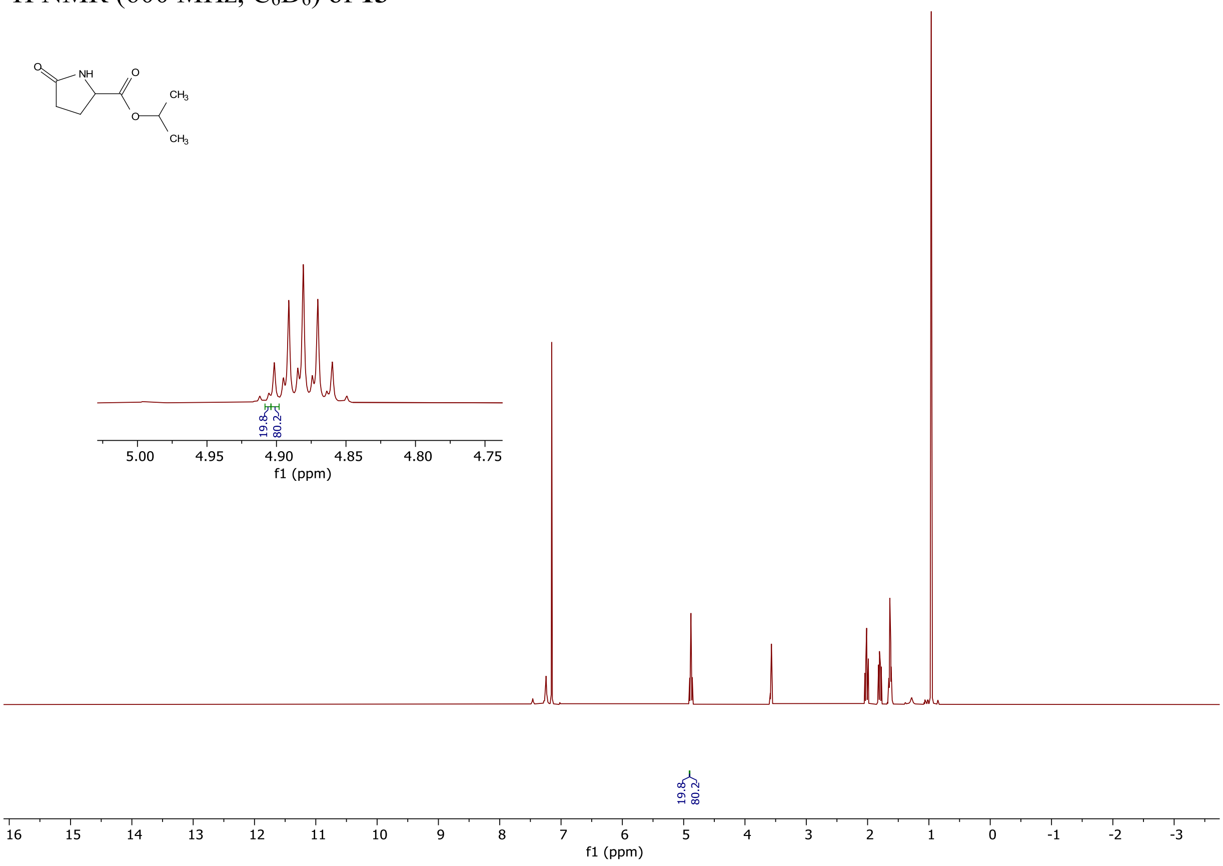

^{1}H-NMR (600 MHz, toluene-d_8) of **14**

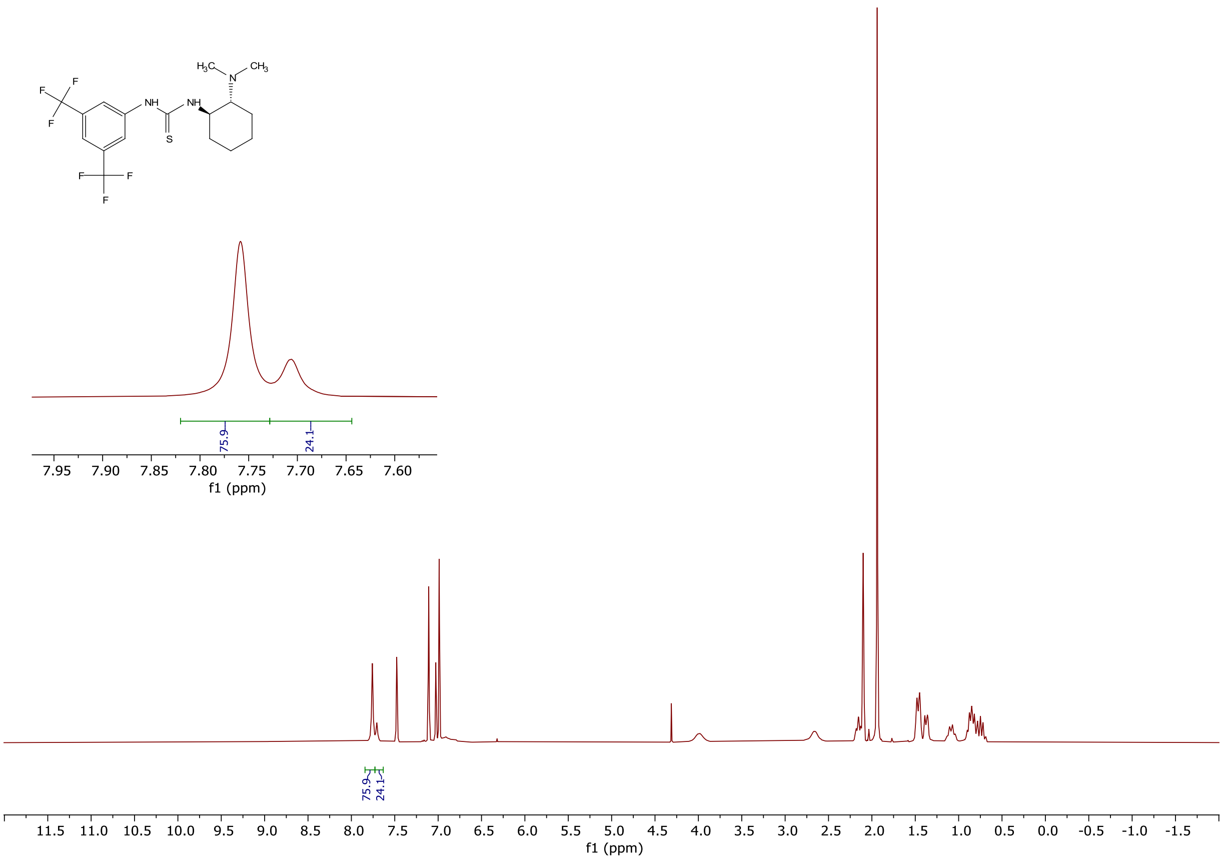

^{31}P-NMR (162 MHz, toluene-d_8) of **15**

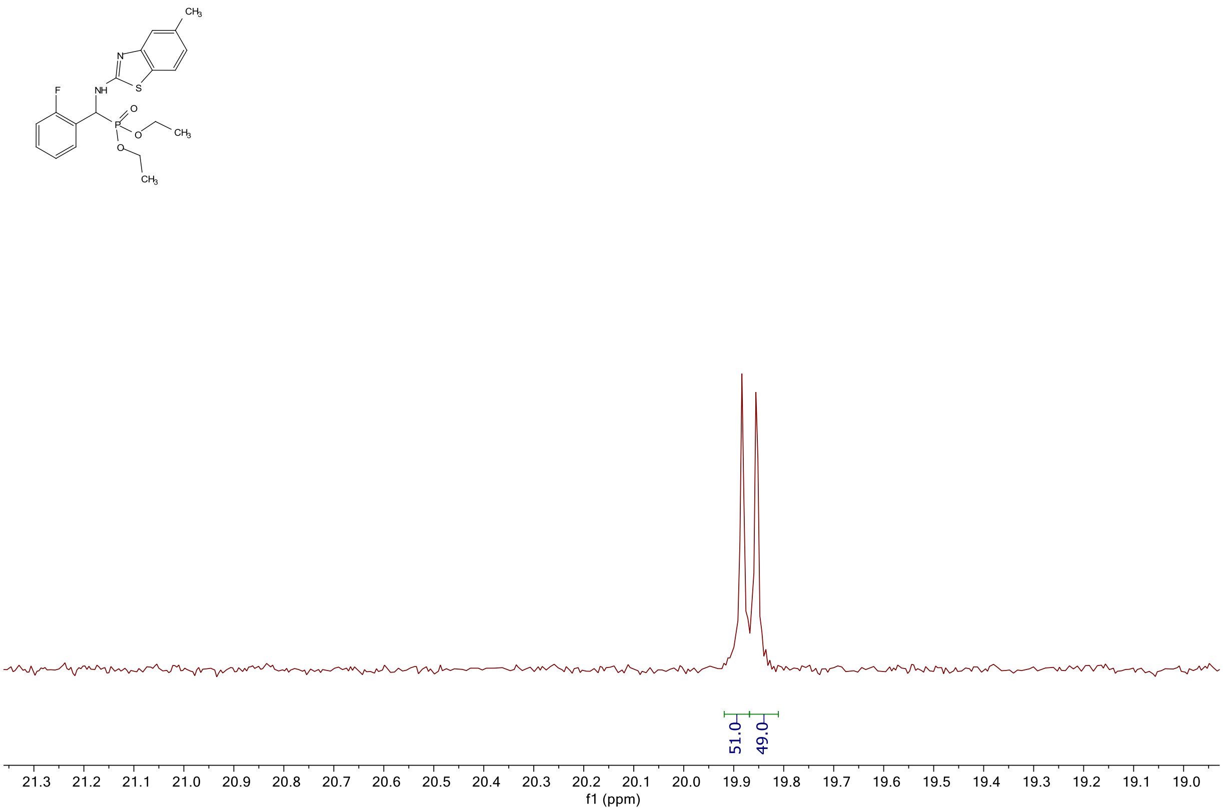

^{31}P-NMR (162 MHz, toluene-d_8) of *rac*-**15**

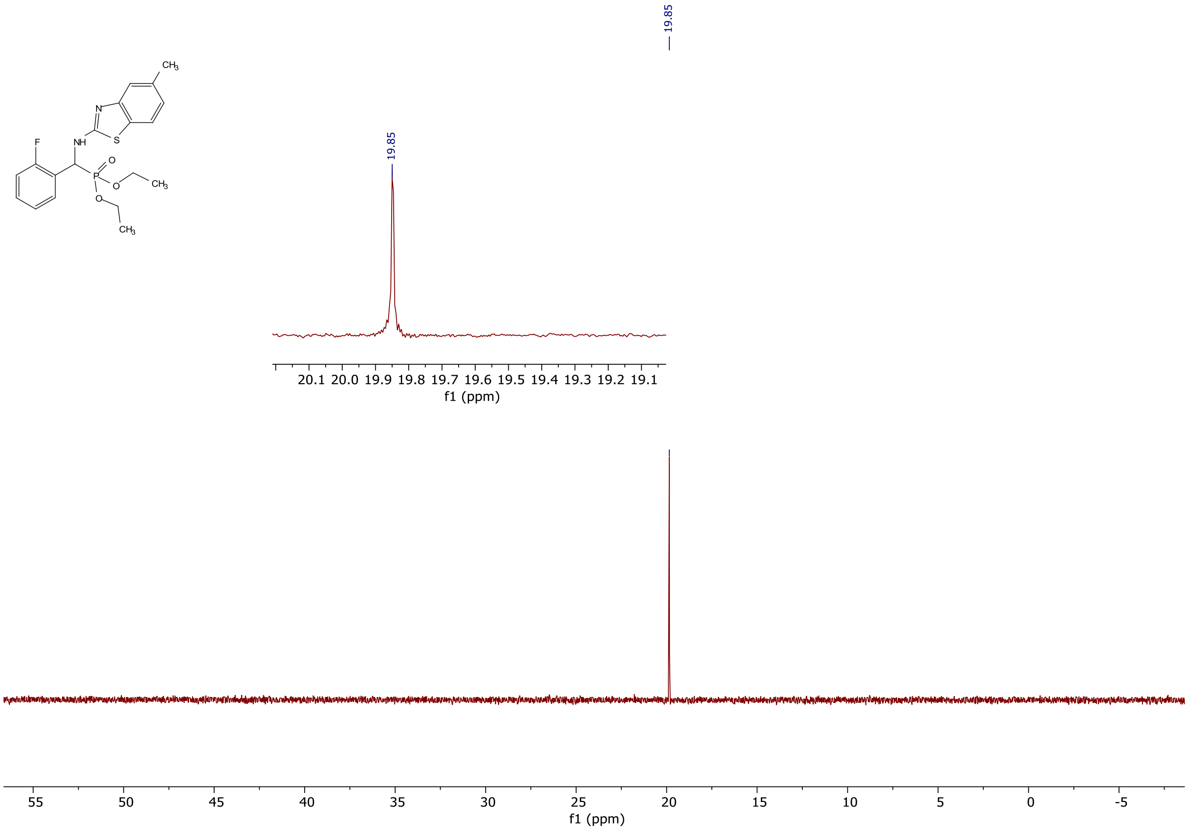

^{31}P-NMR (162 MHz, toluene-d_8) of **16**

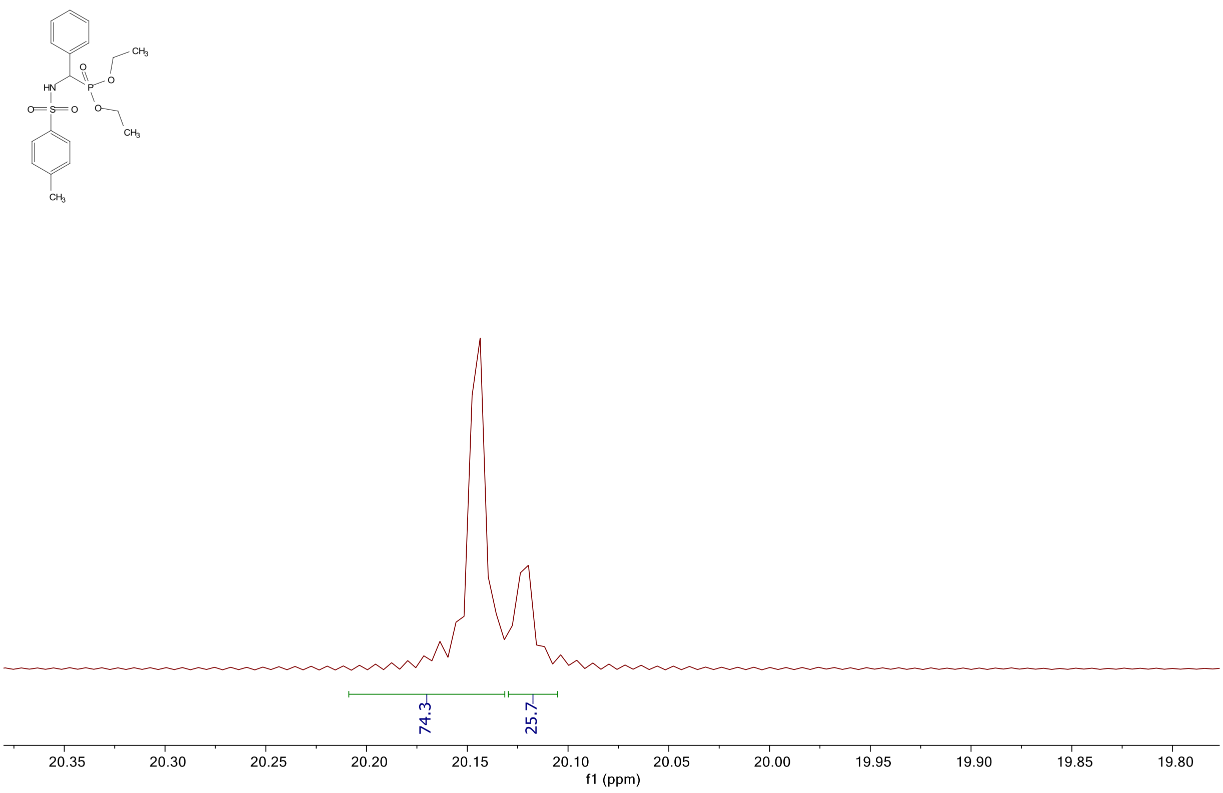

^{1}H-NMR (600 MHz, toluene-d_8) of **S4**

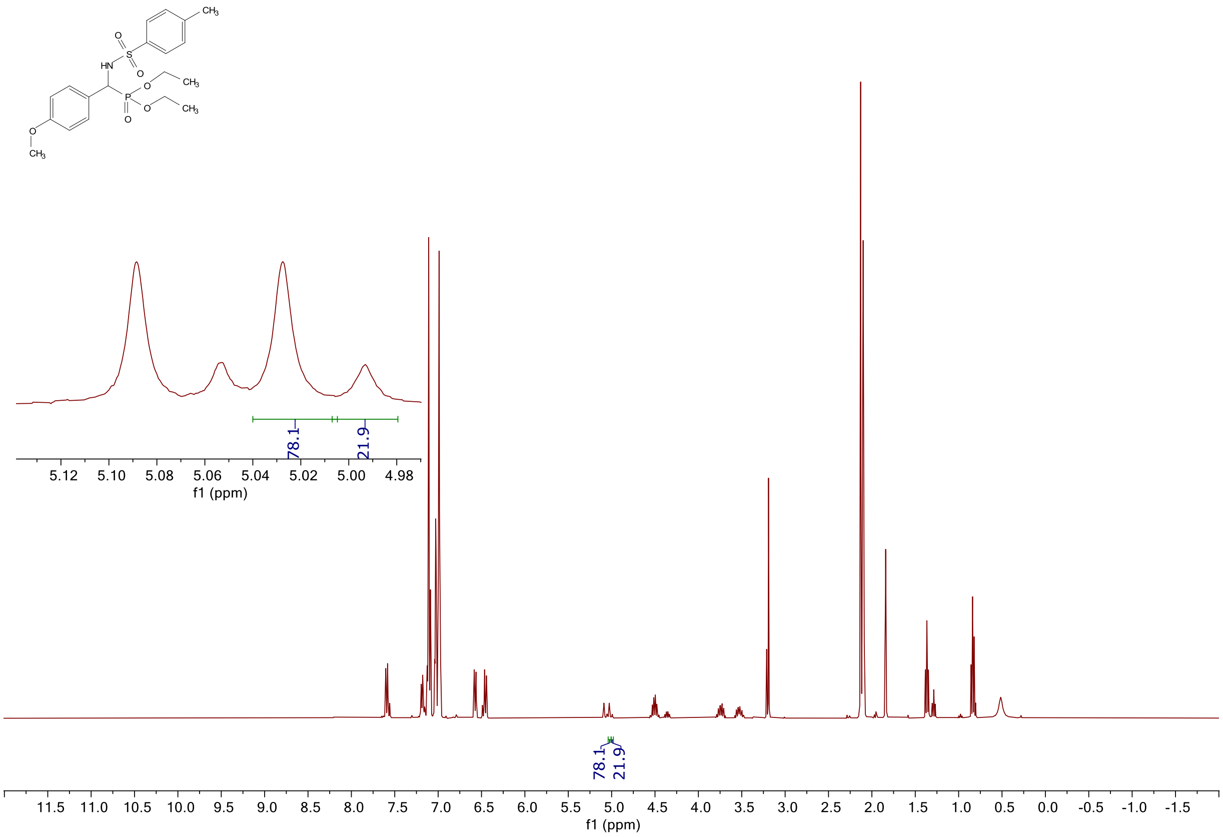

^{31}P-NMR (162 MHz, $CDCl_3$) of **S6**

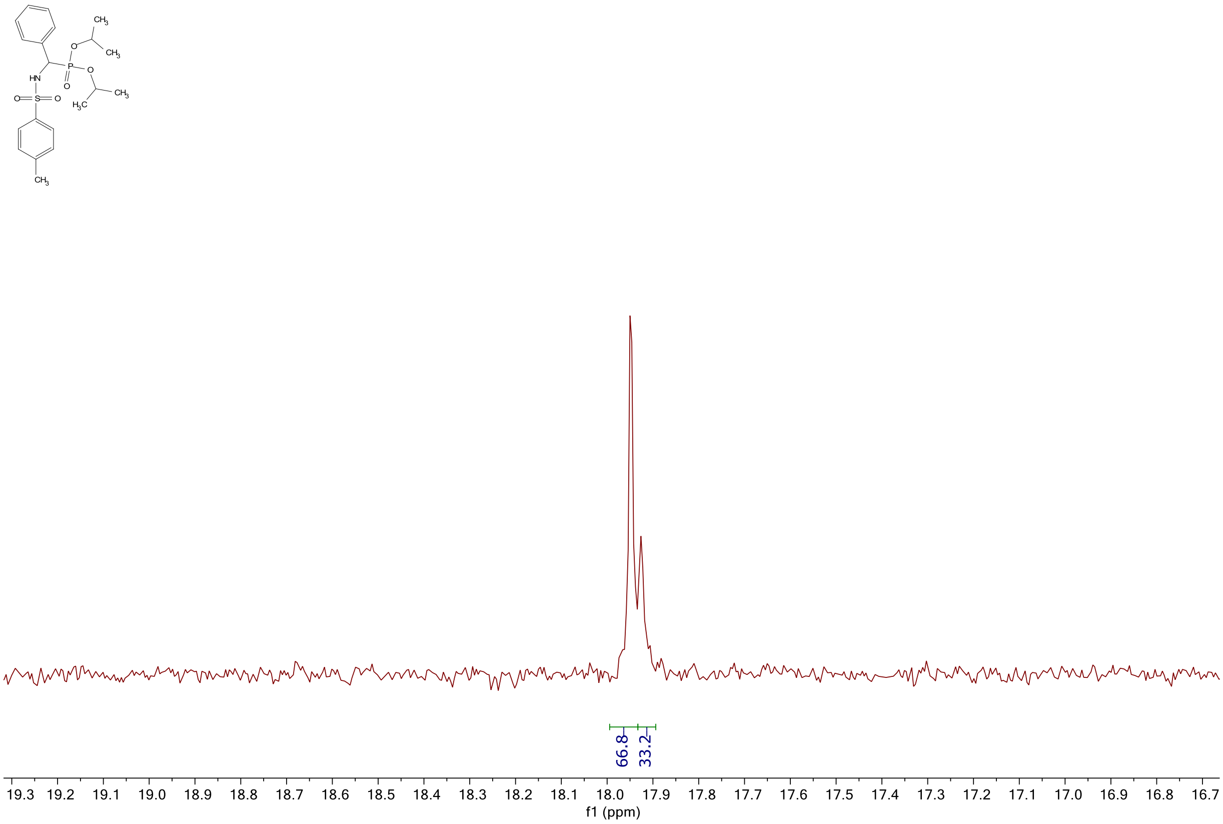

^{31}P-NMR (162 MHz, $CDCl_3$) of **S7**

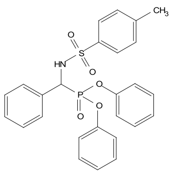

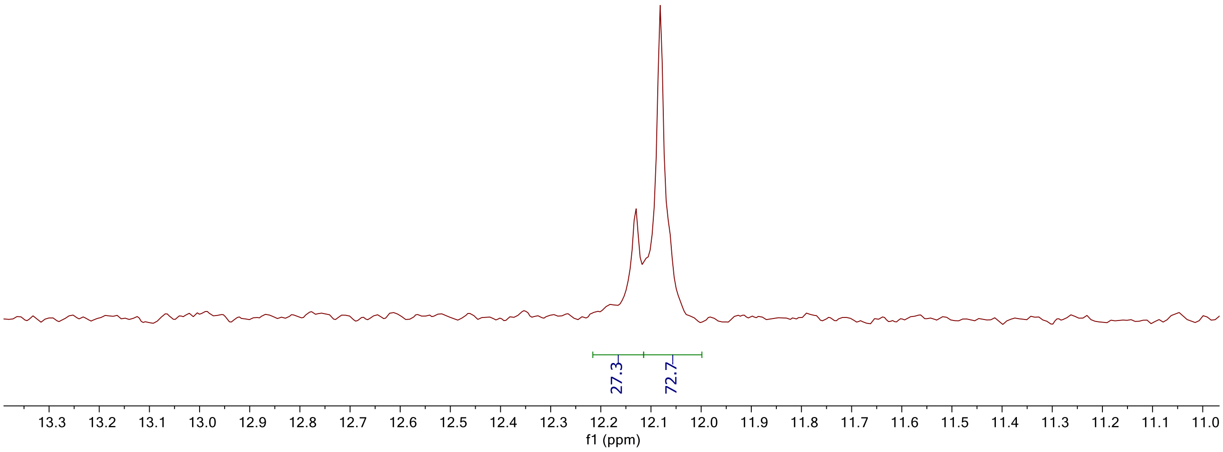

8. Overview of all compounds tested for SIDA activity

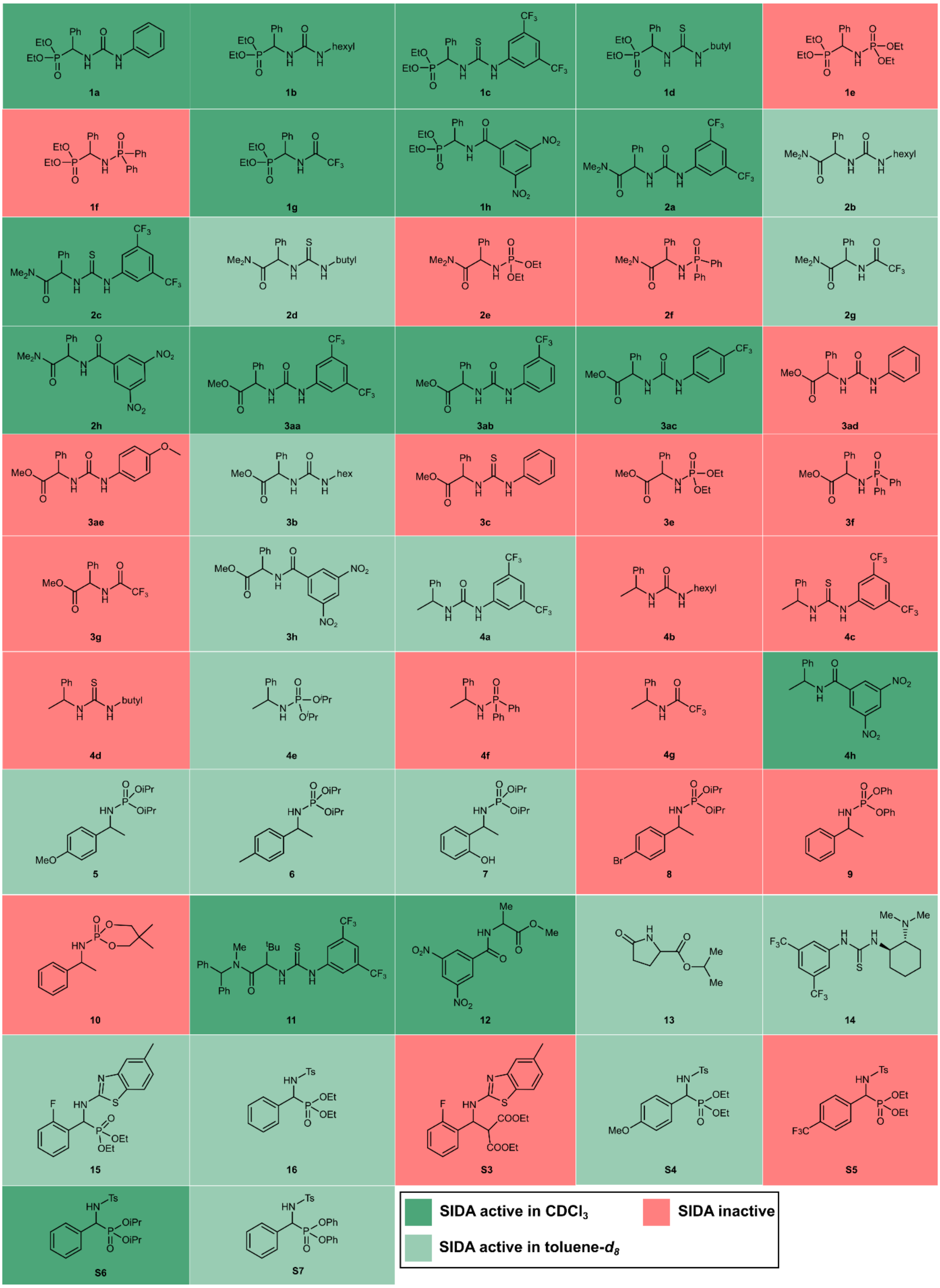

9. Additional compounds tested for SIDA activity

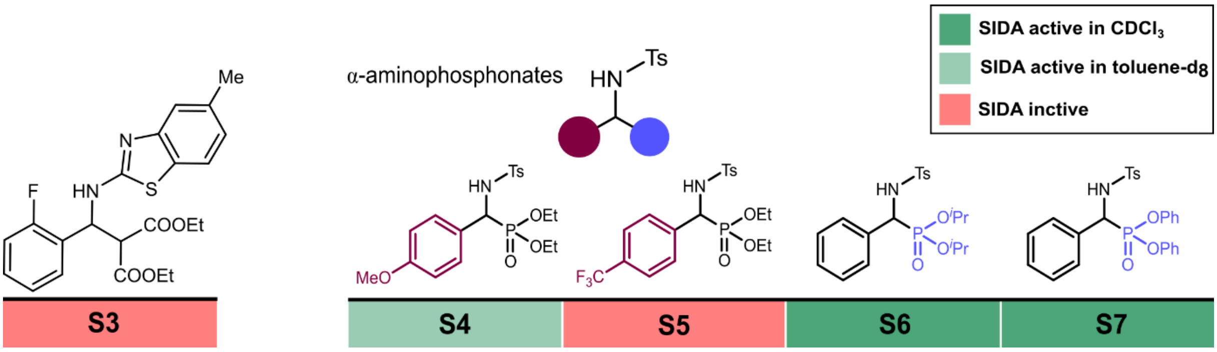

10.NMR spectra

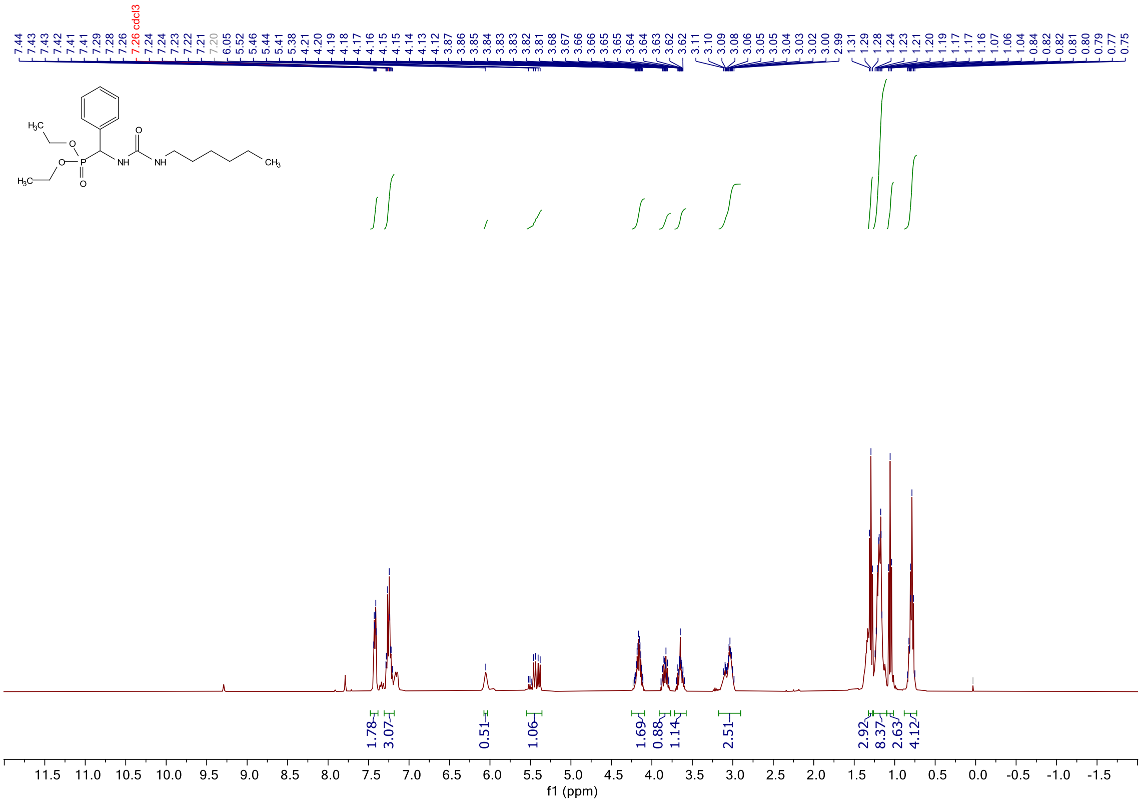

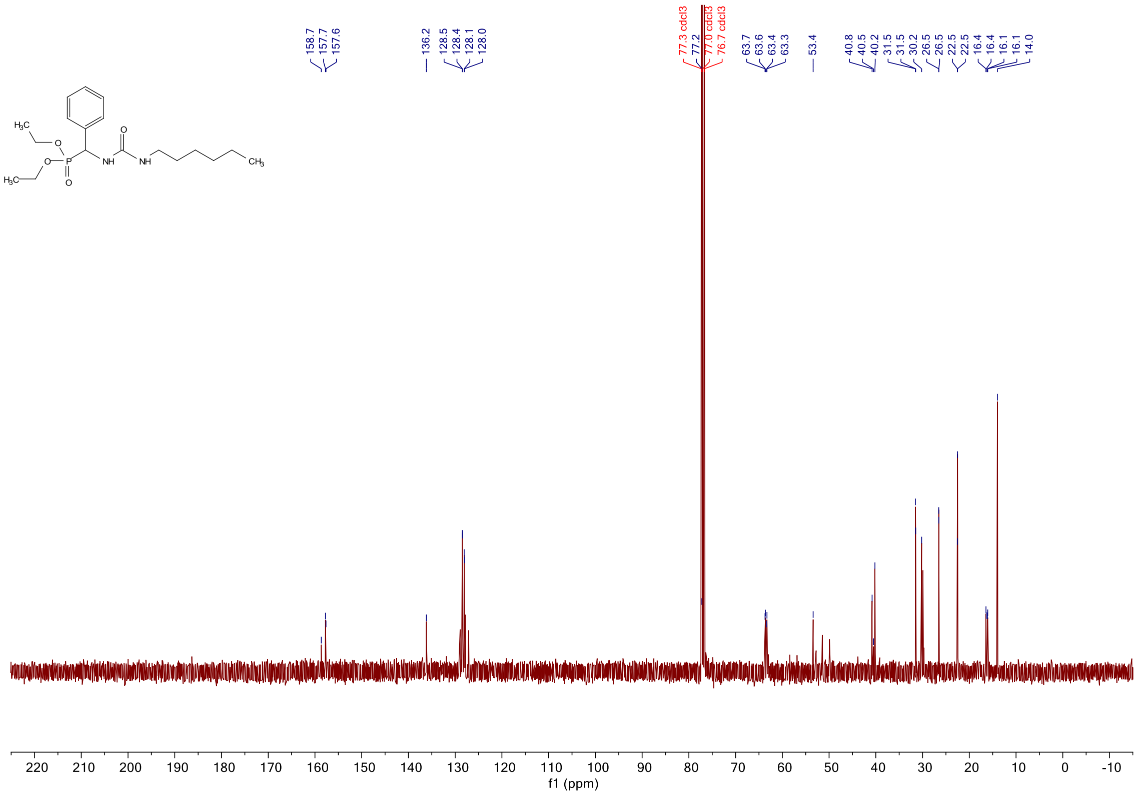

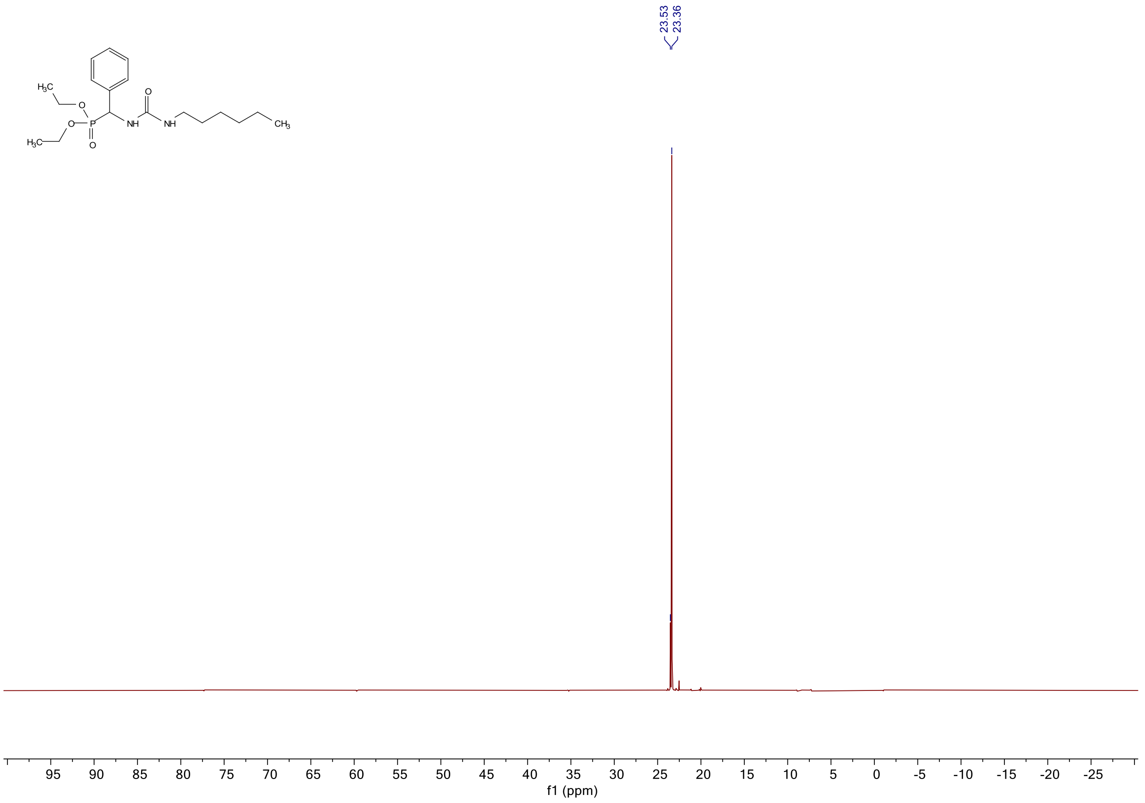
23.53
23.36
H3C
O
P
O
NH
NH
O
CH3
f1 (ppm)

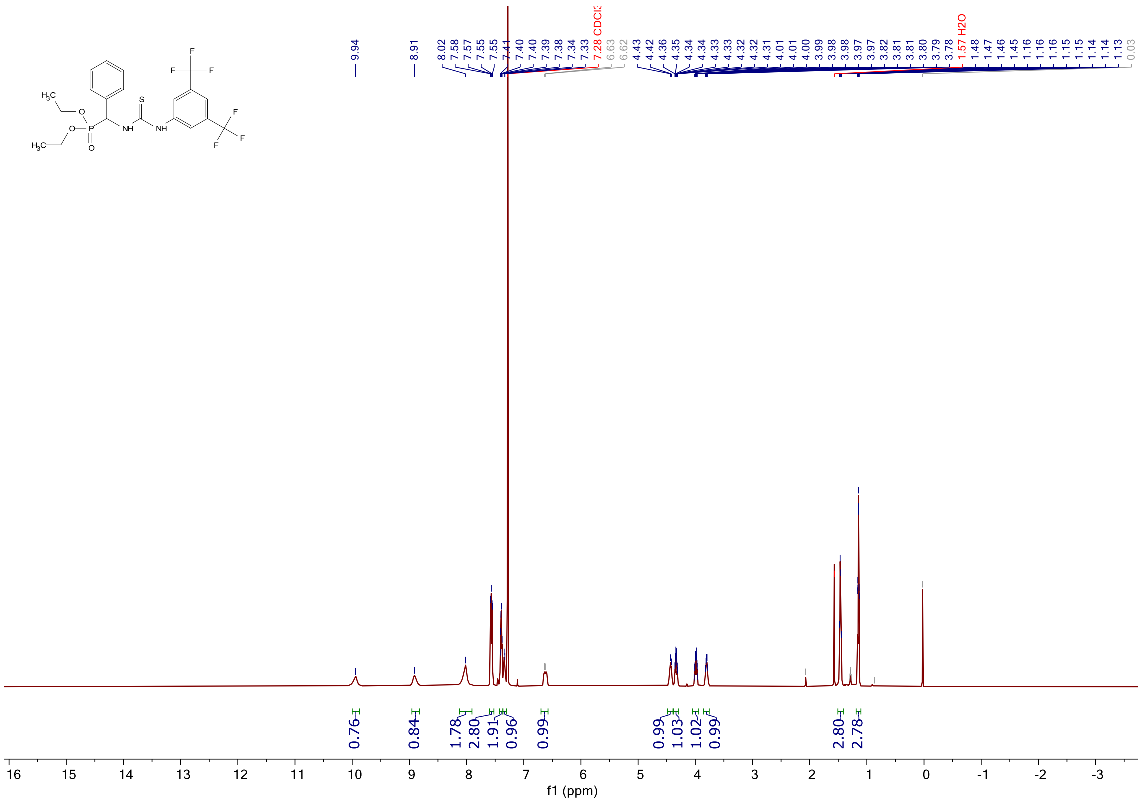
9.94
8.91
8.02
7.58
7.57
7.55
7.55
7.41
7.40
7.40
7.39
7.38
7.34
7.33
7.28 CDCl3
6.63
6.62
4.43
4.42
4.36
4.35
4.34
4.34
4.33
4.33
4.32
4.32
4.31
4.01
4.01
4.00
3.99
3.98
3.98
3.97
3.82
3.81
3.81
3.80
3.79
3.78
1.57 H2O
1.48
1.47
1.46
1.45
1.16
1.16
1.16
1.15
1.14
1.14
1.13
0.03
0.76
0.84
1.78
2.80
1.91
0.96
0.99
0.99
1.03
1.02
0.99
2.80
2.78
H3C
O
P
O
NH
S
NH
F
f1 (ppm)

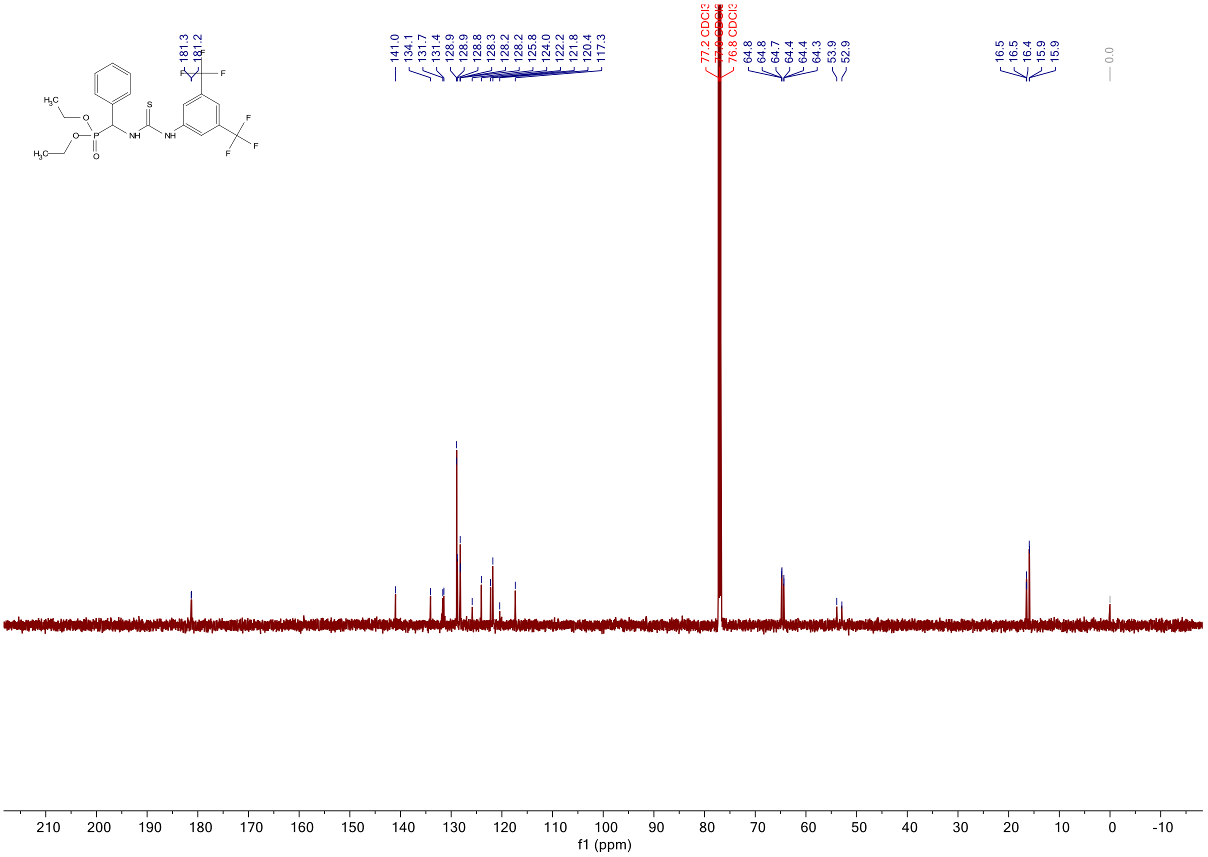
181.3
181.2
141.0
134.1
131.7
131.4
128.9
128.9
128.8
128.3
128.2
128.2
125.8
124.0
122.2
121.8
120.4
117.3
77.2 CDCl3
76.8 CDCl3
64.8
64.8
64.7
64.4
64.4
64.3
53.9
52.9
16.5
16.5
16.4
15.9
15.9
0.0
f1 (ppm)

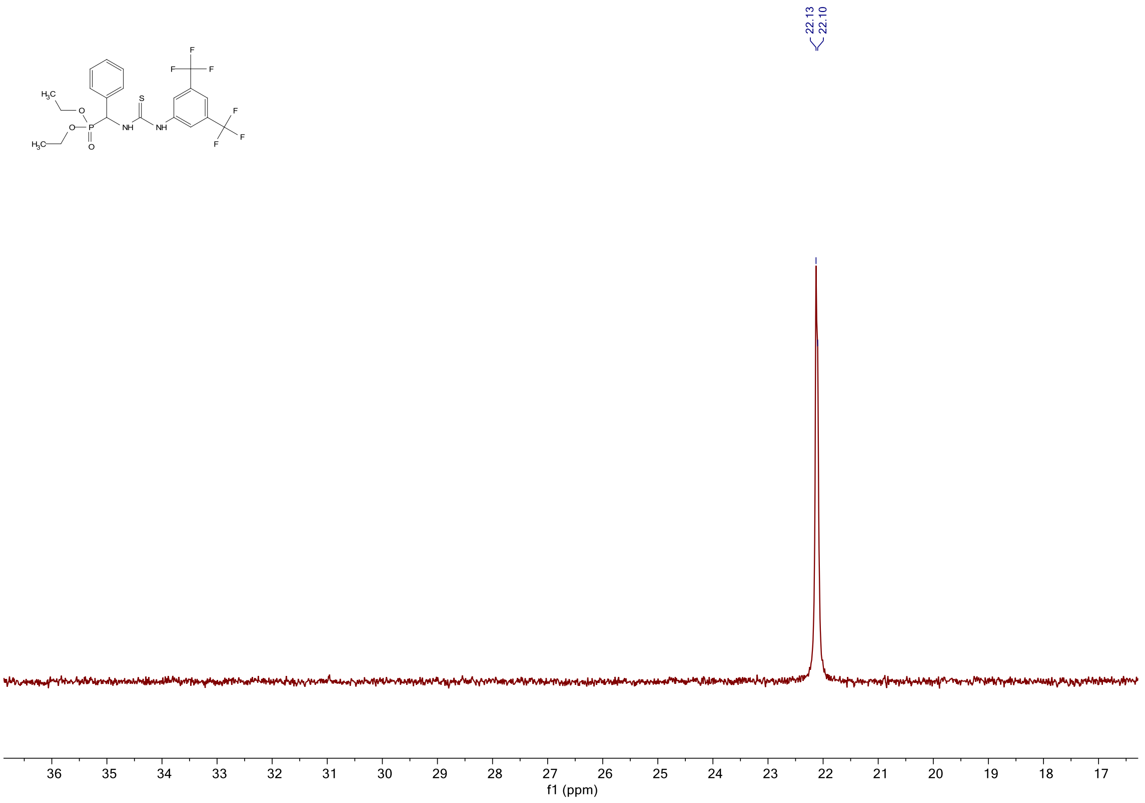
22.13
22.10
f1 (ppm)

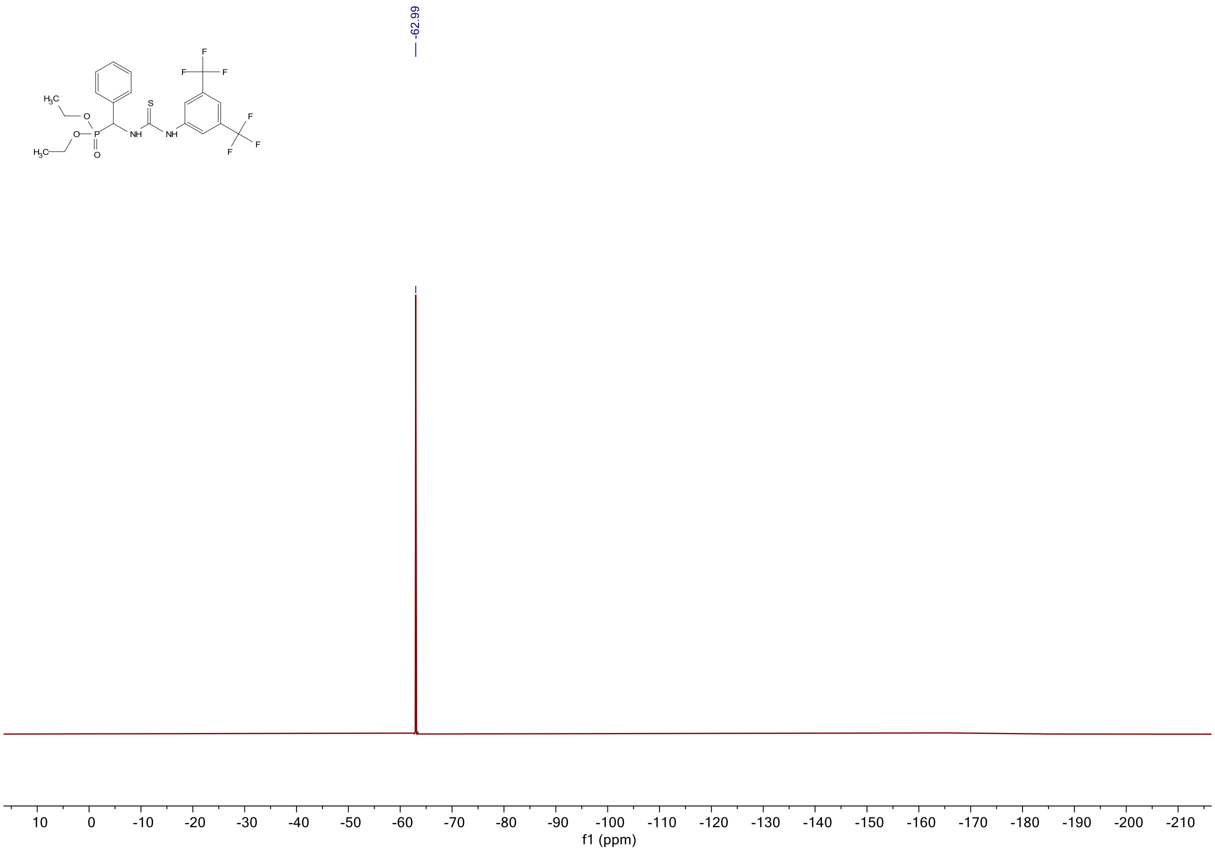

-62.99
H3C
O
O
P
O
H3C
NH
S
NH
F
F
F
F
F
F
f1 (ppm)

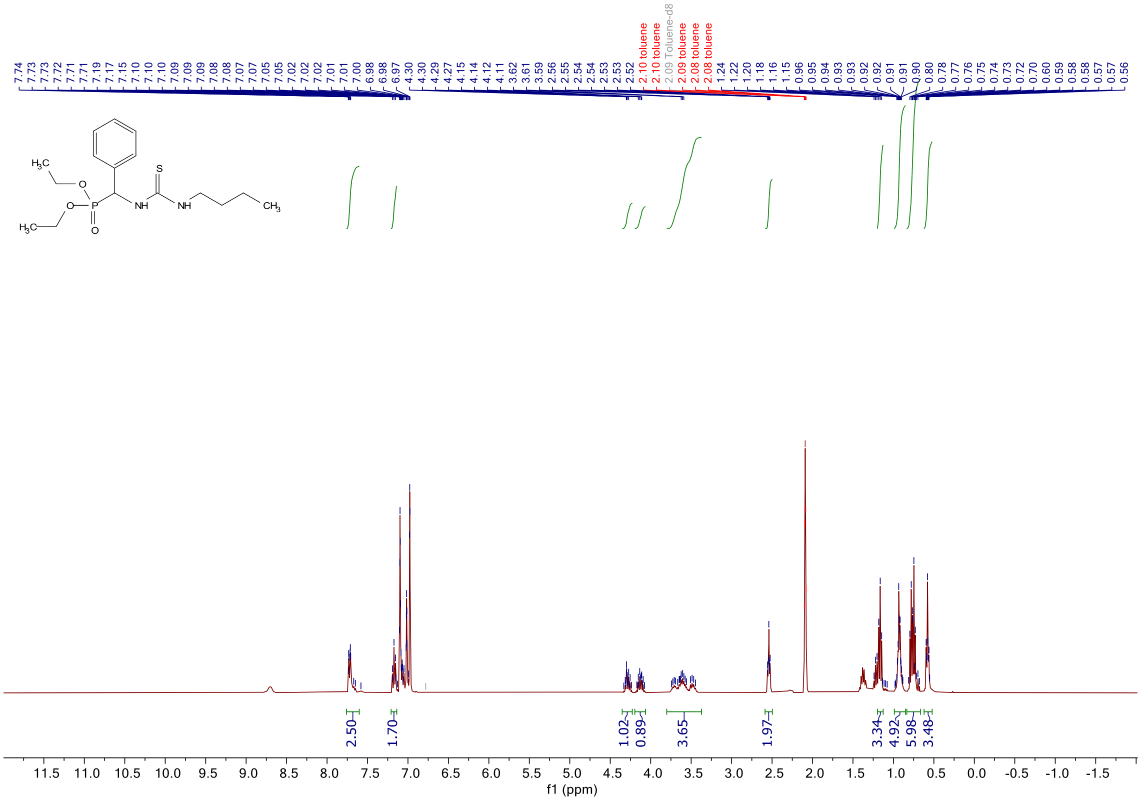

7.74 7.73 7.73 7.72 7.71 7.71 7.19 7.17 7.15 7.10 7.10 7.10 7.09 7.09 7.09 7.08 7.08 7.07 7.07 7.05 7.05 7.02 7.02 7.02 7.01 7.00 6.98 6.98 6.97 4.30 4.30 4.29 4.27 4.15 4.14 4.12 4.11 3.62 3.61 3.59 2.56 2.55 2.54 2.54 2.53 2.53 2.52
2.10 toluene
2.10 toluene
2.09 Toluene-d8
2.09 toluene
2.08 toluene
2.08 toluene
1.24 1.22 1.20 1.18 1.16 1.15 0.96 0.95 0.94 0.93 0.93 0.92 0.92 0.91 0.90 0.80 0.78 0.77 0.76 0.75 0.74 0.73 0.72 0.70 0.60 0.59 0.58 0.58 0.57 0.56
H3C
O
O
P
O
H3C
NH
S
NH
CH3
2.50
1.70
1.02
0.89
3.65
1.97
3.34
4.92
5.98
3.48
f1 (ppm)

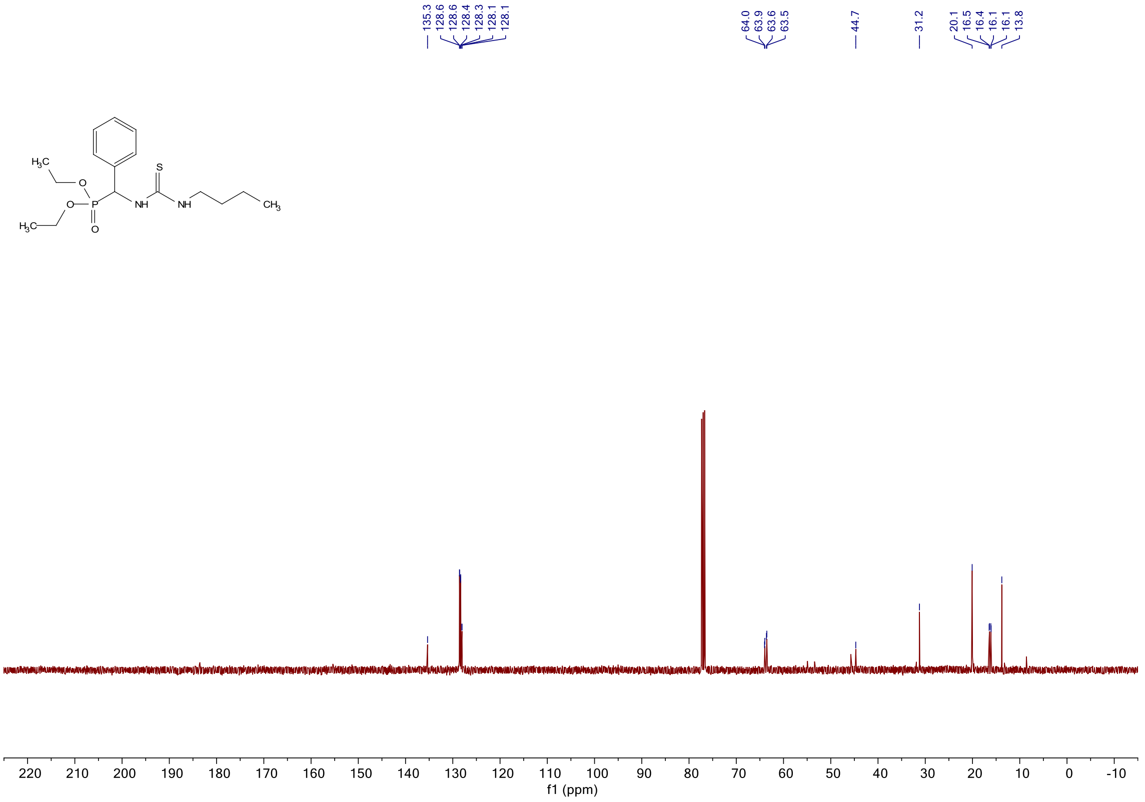
135.3
128.6
128.6
128.4
128.3
128.1
128.1
64.0
63.9
63.6
63.5
44.7
31.2
20.1
16.5
16.4
16.1
16.1
13.8
f1 (ppm)

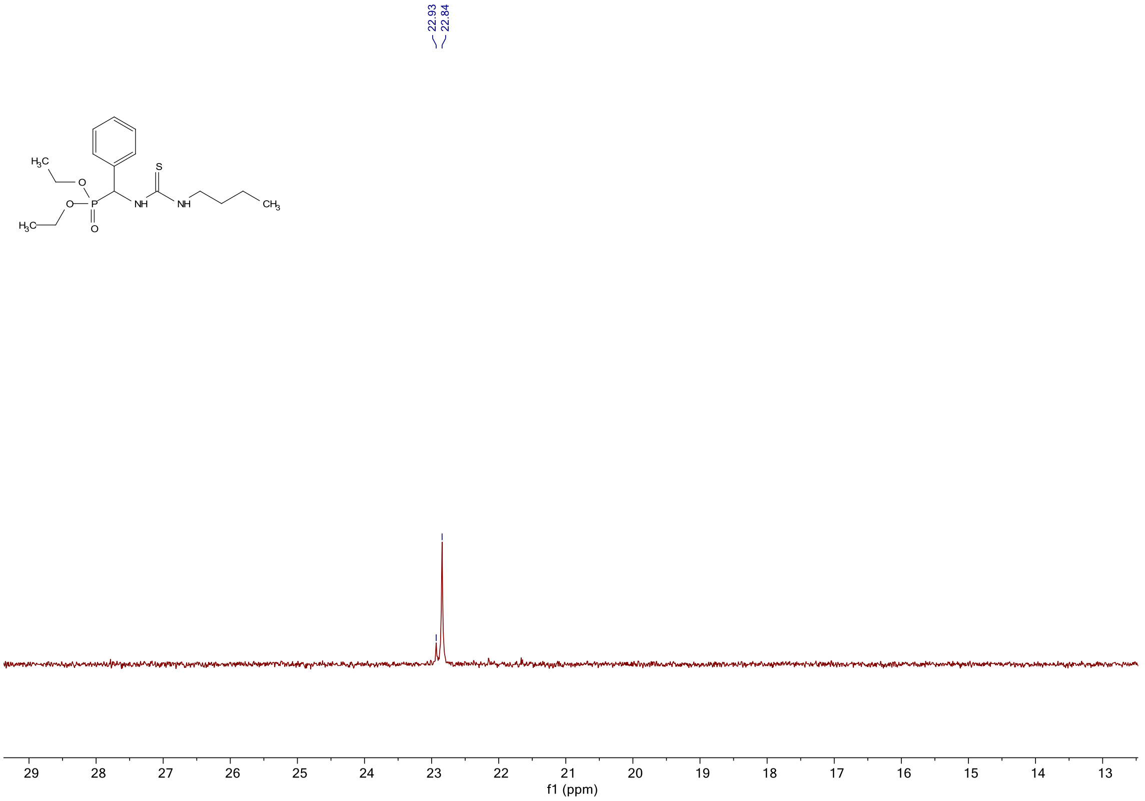
22.93
22.84
f1 (ppm)

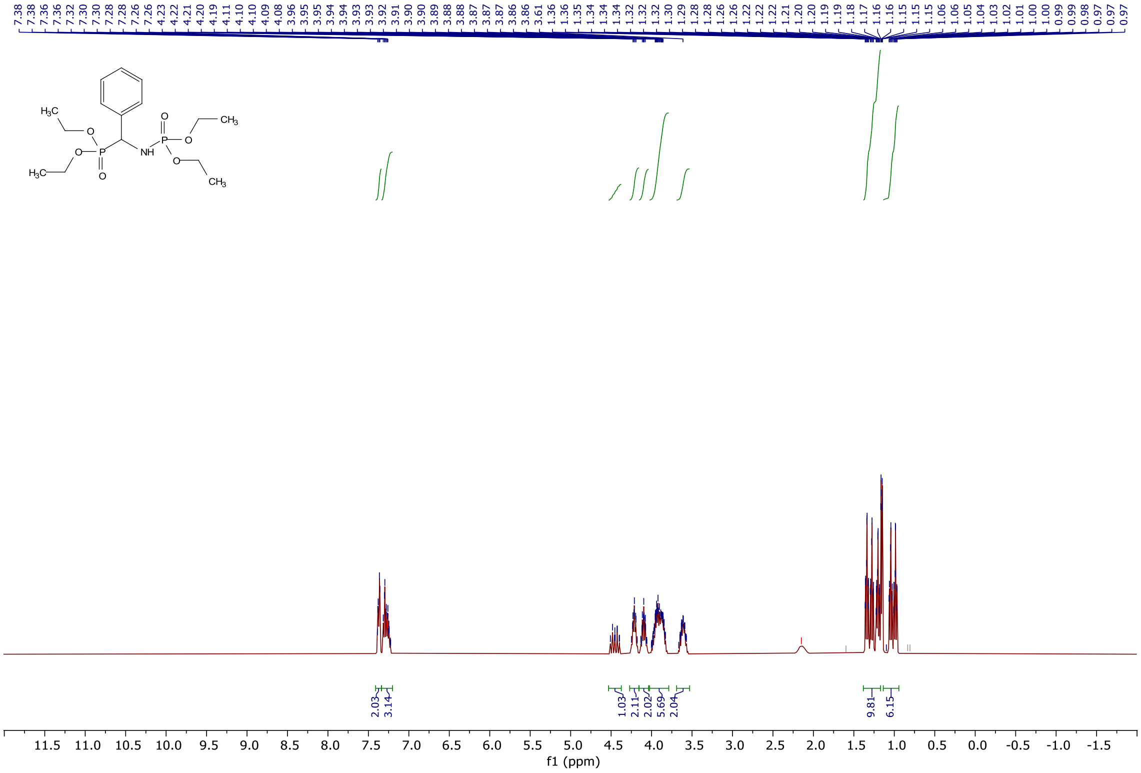
7.38 7.38 7.36 7.36 7.32 7.30 7.30 7.28 7.28 7.26 7.26 4.23 4.22 4.21 4.20 4.19 4.11 4.10 4.10 4.09 4.08 3.96 3.95 3.95 3.94 3.94 3.93 3.93 3.92 3.91 3.90 3.90 3.89 3.88 3.88 3.87 3.87 3.86 3.61 1.36 1.35 1.34 1.34 1.34 1.32 1.32 1.32 1.32 1.30 1.29 1.28 1.28 1.26 1.26 1.22 1.22 1.22 1.21 1.20 1.20 1.19 1.19 1.18 1.17 1.16 1.16 1.15 1.15 1.15 1.06 1.05 1.04 1.03 1.02 1.01 1.00 1.00 0.99 0.98 0.97 0.97
2.03
3.14
1.03
2.11
2.02
5.69
2.04
9.81
6.15
f1 (ppm)

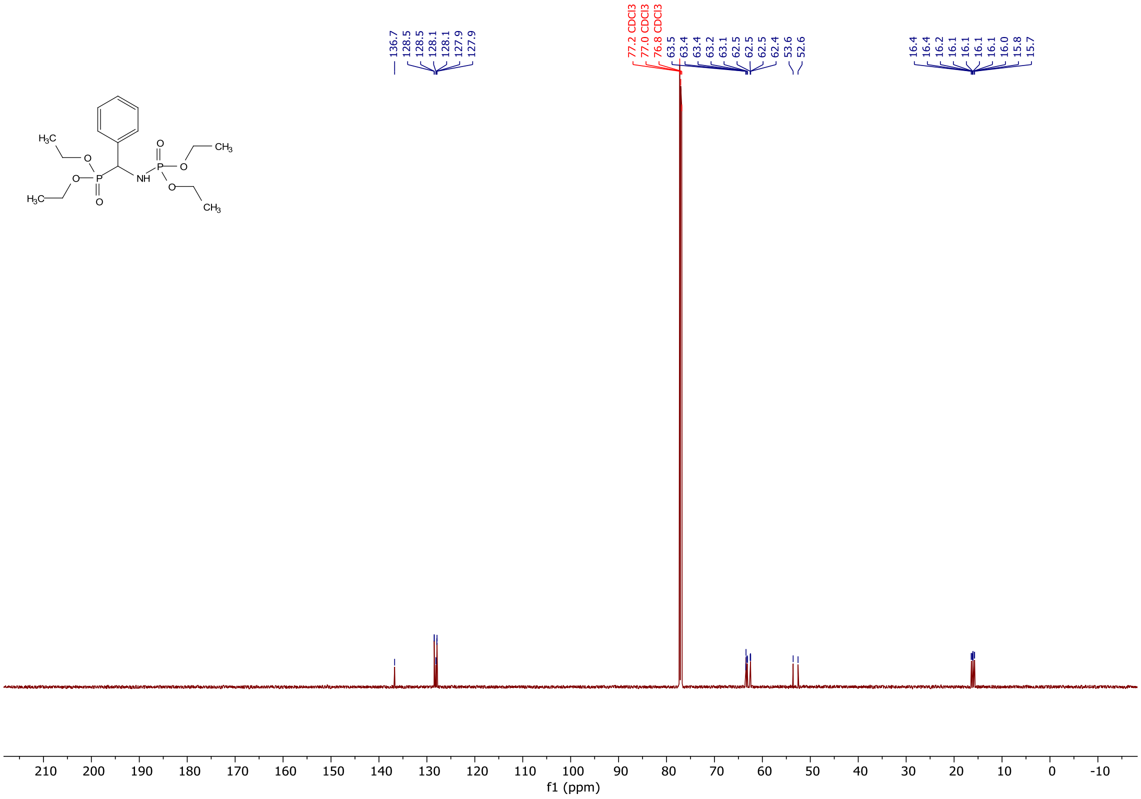
136.7 128.5 128.5 128.1 128.1 127.9 127.9
77.2 CDCl3
77.0 CDCl3
76.8 CDCl3
63.5 63.4 63.4 63.2 63.1 62.5 62.5 62.4 53.6 52.6
16.4 16.4 16.2 16.1 16.1 16.1 16.1 16.0 15.8 15.7
f1 (ppm)

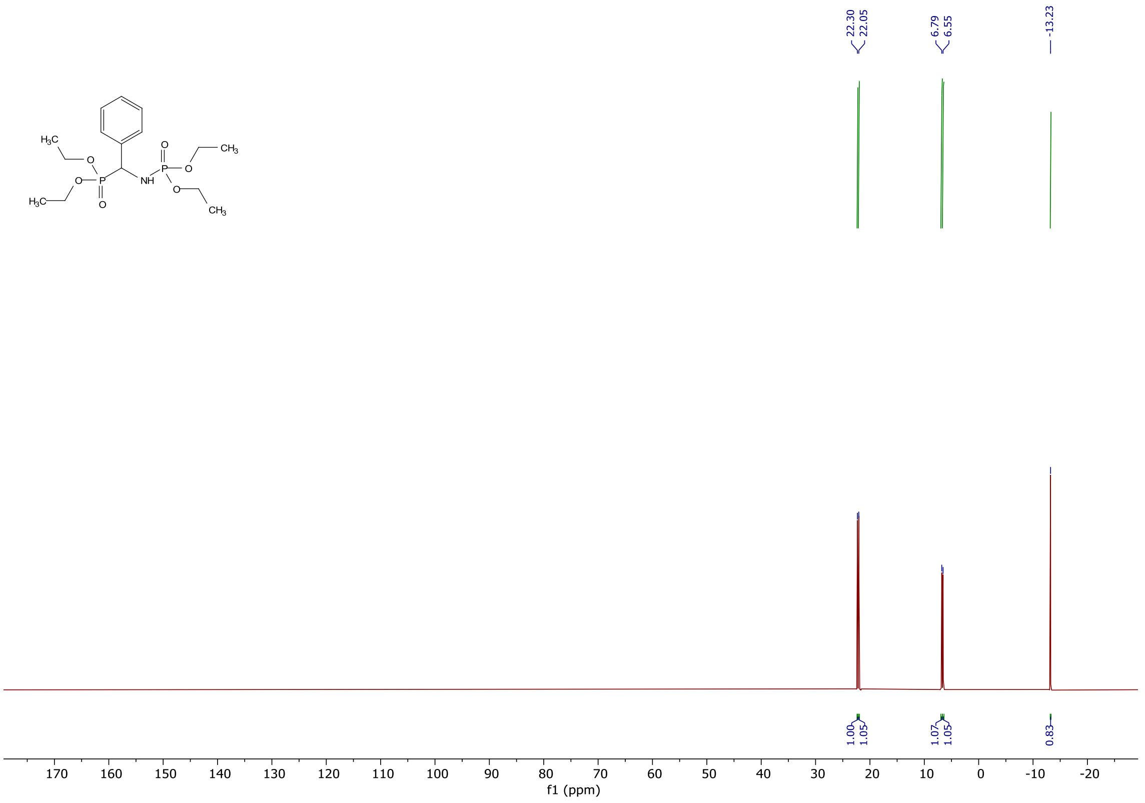

22.30
22.05
6.79
6.55
-13.23
1.00
1.05
1.07
1.05
0.83
170 160 150 140 130 120 110 100 90 80 70 60 50 40 30 20 10 0 -10 -20
f1 (ppm)

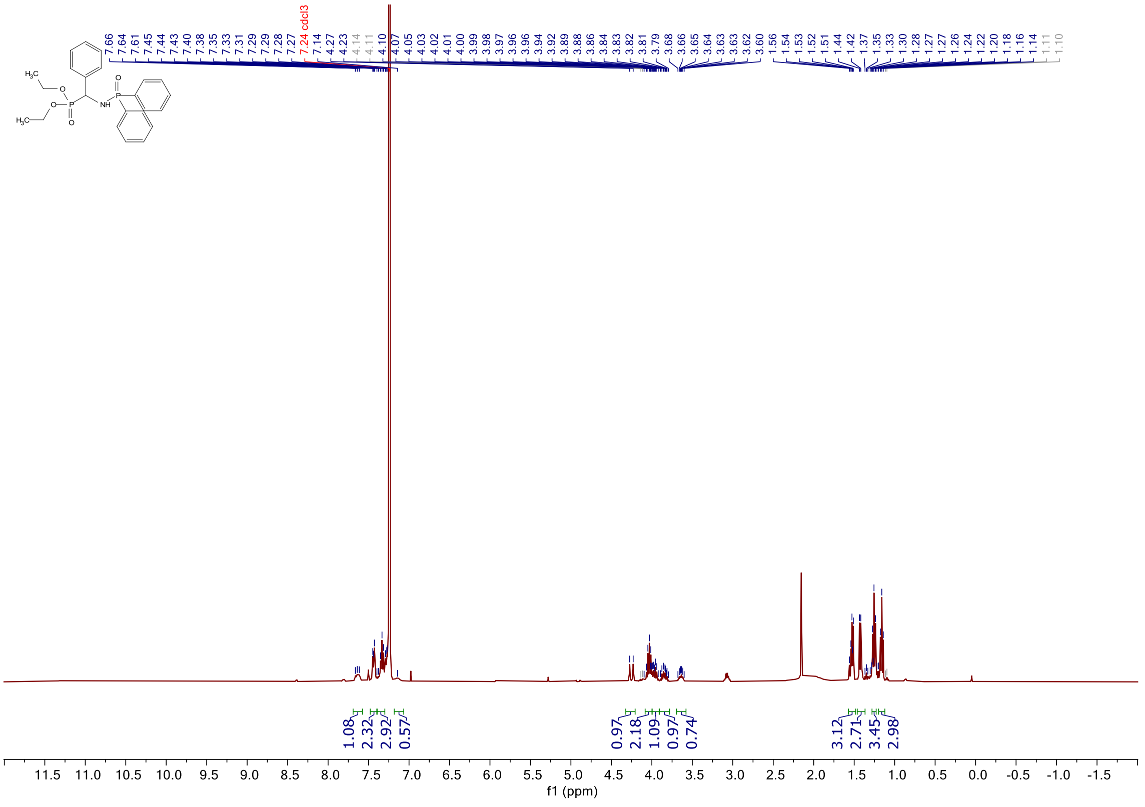

7.66 7.64 7.61 7.45 7.44 7.43 7.40 7.38 7.35 7.33 7.31 7.29 7.28 7.27
7.24 cdcl3
7.14 4.27 4.23 4.14 4.11 4.10 4.07 4.05 4.03 4.02 4.01 4.00 3.99 3.98 3.97 3.96 3.96 3.94 3.92 3.89 3.88 3.86 3.84 3.83 3.82 3.81 3.79 3.68 3.66 3.65 3.64 3.63 3.63 3.62 3.60 1.56 1.54 1.53 1.52 1.51 1.44 1.42 1.37 1.35 1.33 1.30 1.28 1.27 1.26 1.24 1.22 1.20 1.18 1.16 1.14 1.11 1.10
1.08 2.32 2.92 0.57 0.97 2.18 1.09 0.97 0.74 3.12 2.71 3.45 2.98
11.5 11.0 10.5 10.0 9.5 9.0 8.5 8.0 7.5 7.0 6.5 6.0 5.5 5.0 4.5 4.0 3.5 3.0 2.5 2.0 1.5 1.0 0.5 0.0 -0.5 -1.0 -1.5
f1 (ppm)

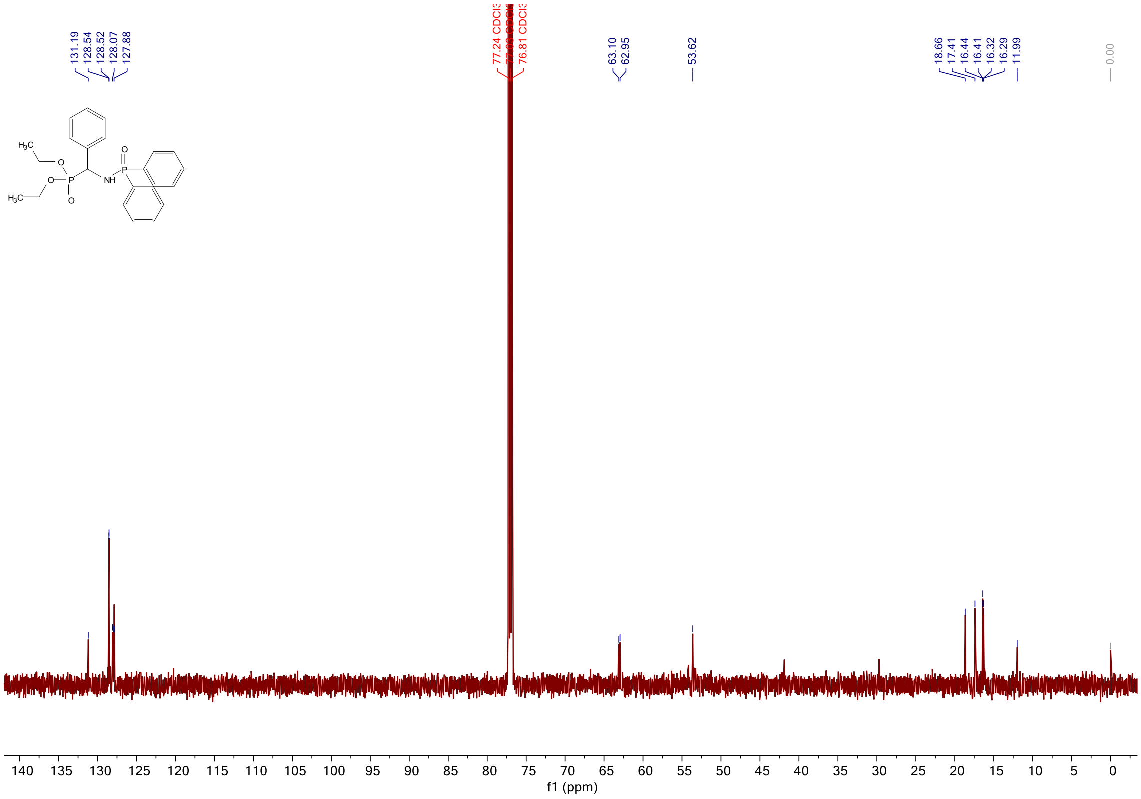
131.19
128.54
128.52
128.07
127.88
77.24 CDCl3
76.81 CDCl3
63.10
62.95
53.62
18.66
17.41
16.44
16.41
16.32
16.29
11.99
0.00
f1 (ppm)

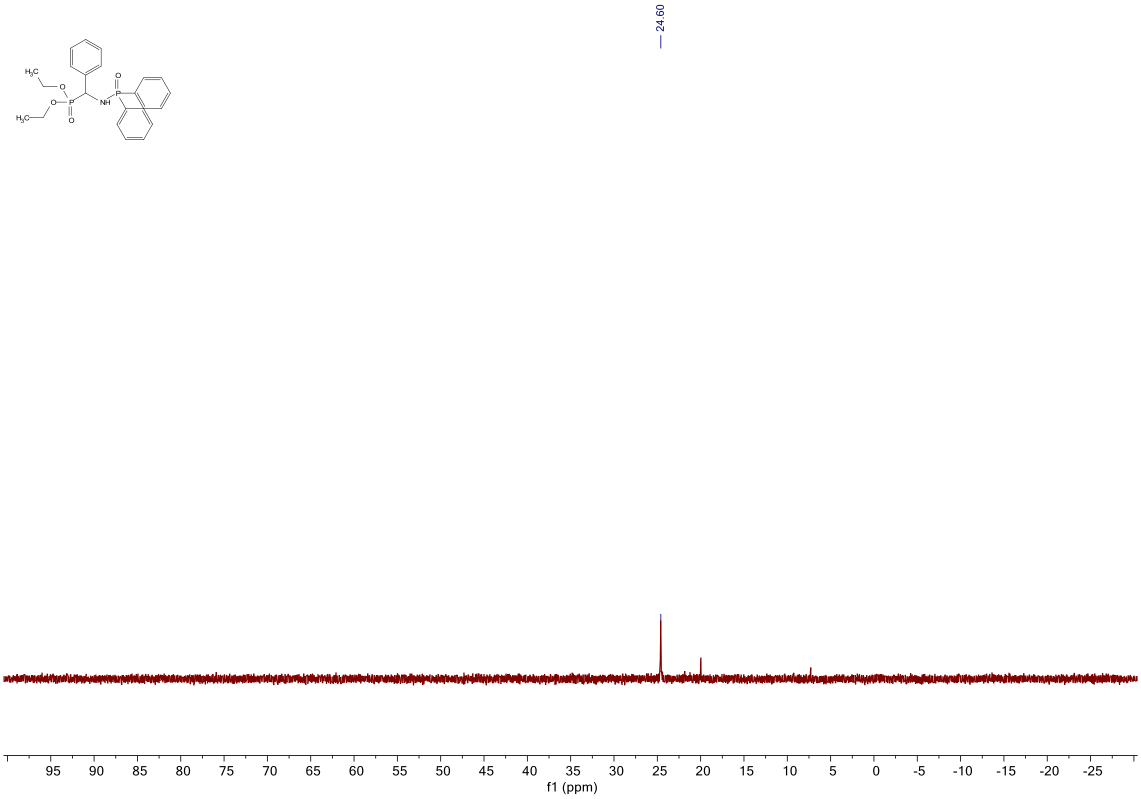
24.60
f1 (ppm)

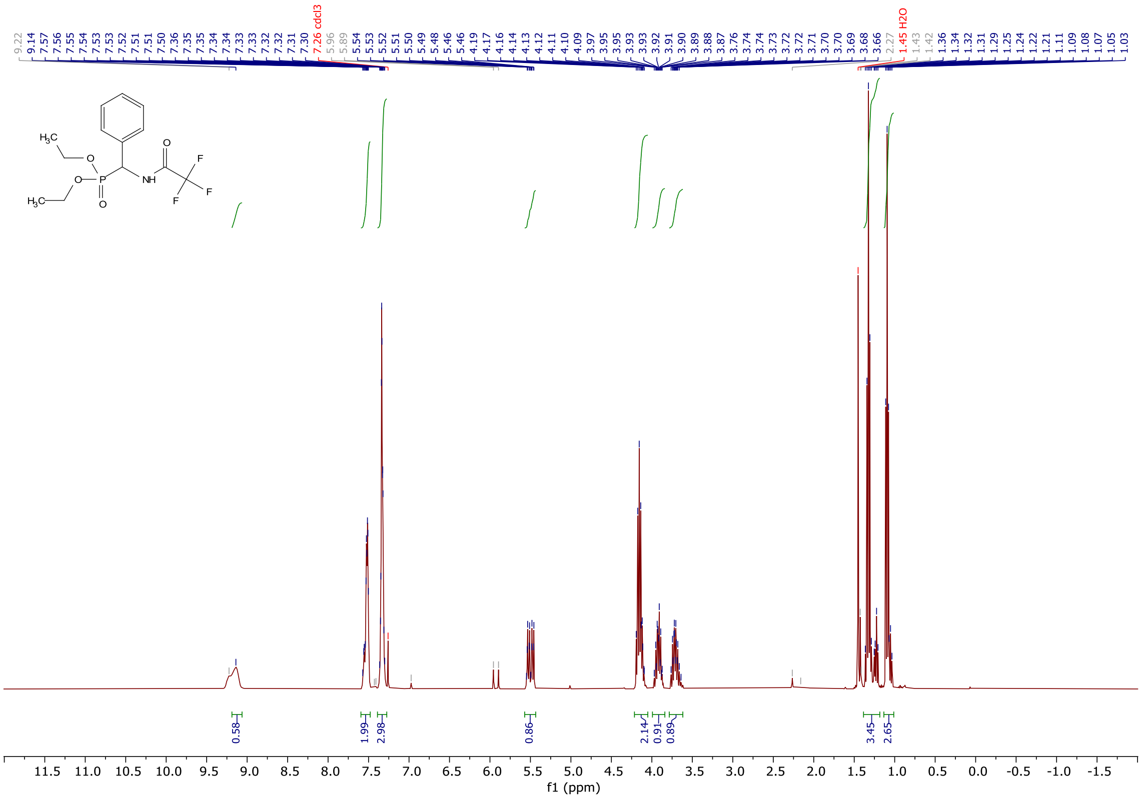

9.22 9.14 7.57 7.56 7.55 7.54 7.53 7.53 7.52 7.51 7.51 7.50 7.36 7.35 7.35 7.34 7.34 7.33 7.33 7.32 7.32 7.31 7.30 7.26 cdcl3 5.96 5.89 5.54 5.53 5.52 5.51 5.50 5.49 5.48 5.46 5.46 4.19 4.17 4.16 4.14 4.13 4.12 4.11 4.10 4.09 3.97 3.95 3.95 3.93 3.93 3.92 3.91 3.90 3.89 3.88 3.87 3.76 3.74 3.74 3.73 3.72 3.72 3.71 3.70 3.69 3.68 3.66 2.27 1.45 H2O 1.43 1.42 1.36 1.34 1.32 1.31 1.29 1.25 1.24 1.22 1.21 1.11 1.09 1.08 1.07 1.05 1.03
0.58 1.99 2.98 0.86 2.14 0.91 0.89 3.45 2.65
11.5 11.0 10.5 10.0 9.5 9.0 8.5 8.0 7.5 7.0 6.5 6.0 5.5 5.0 4.5 4.0 3.5 3.0 2.5 2.0 1.5 1.0 0.5 0.0 -0.5 -1.0 -1.5
f1 (ppm)

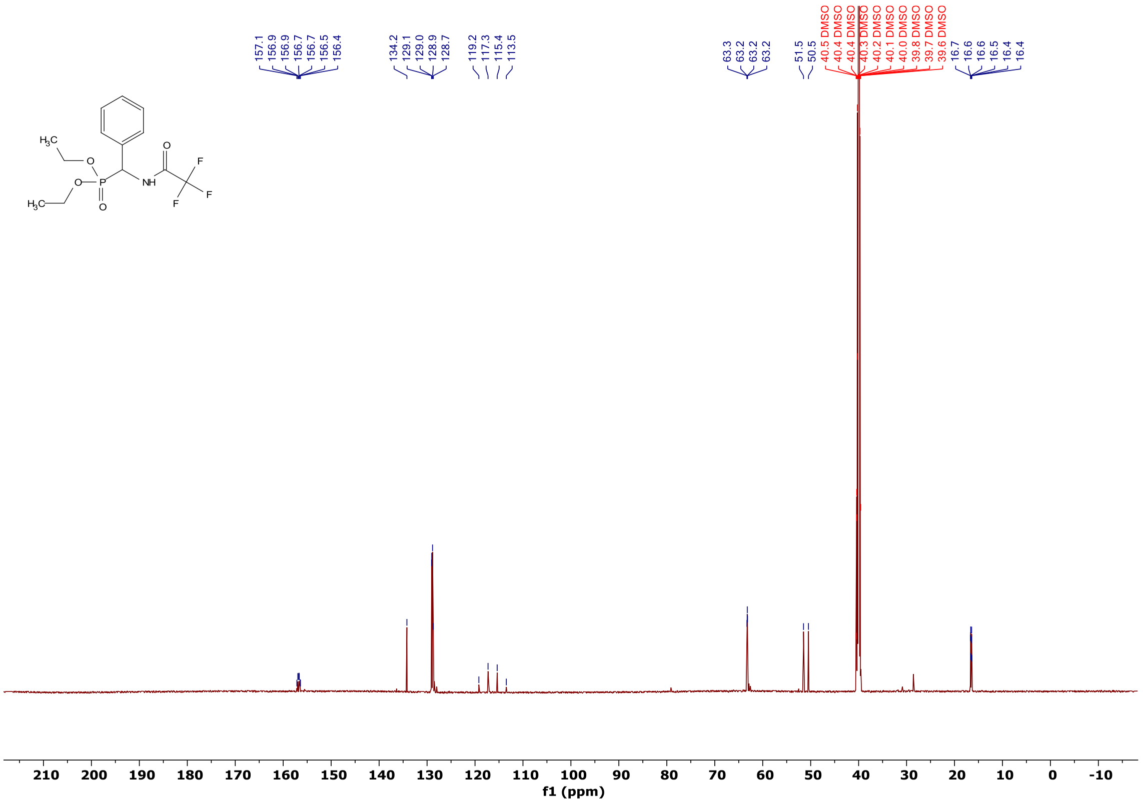

157.1 156.9 156.9 156.7 156.7 156.5 156.4 134.2 129.1 129.0 128.9 128.7 119.2 117.3 115.4 113.5 63.3 63.2 63.2 51.5 50.5 40.5 DMSO 40.4 DMSO 40.4 DMSO 40.3 DMSO 40.3 DMSO 40.2 DMSO 40.1 DMSO 40.0 DMSO 39.8 DMSO 39.7 DMSO 39.6 DMSO 16.7 16.6 16.6 16.5 16.4 16.4
210 200 190 180 170 160 150 140 130 120 110 100 90 80 70 60 50 40 30 20 10 0 -10
f1 (ppm)

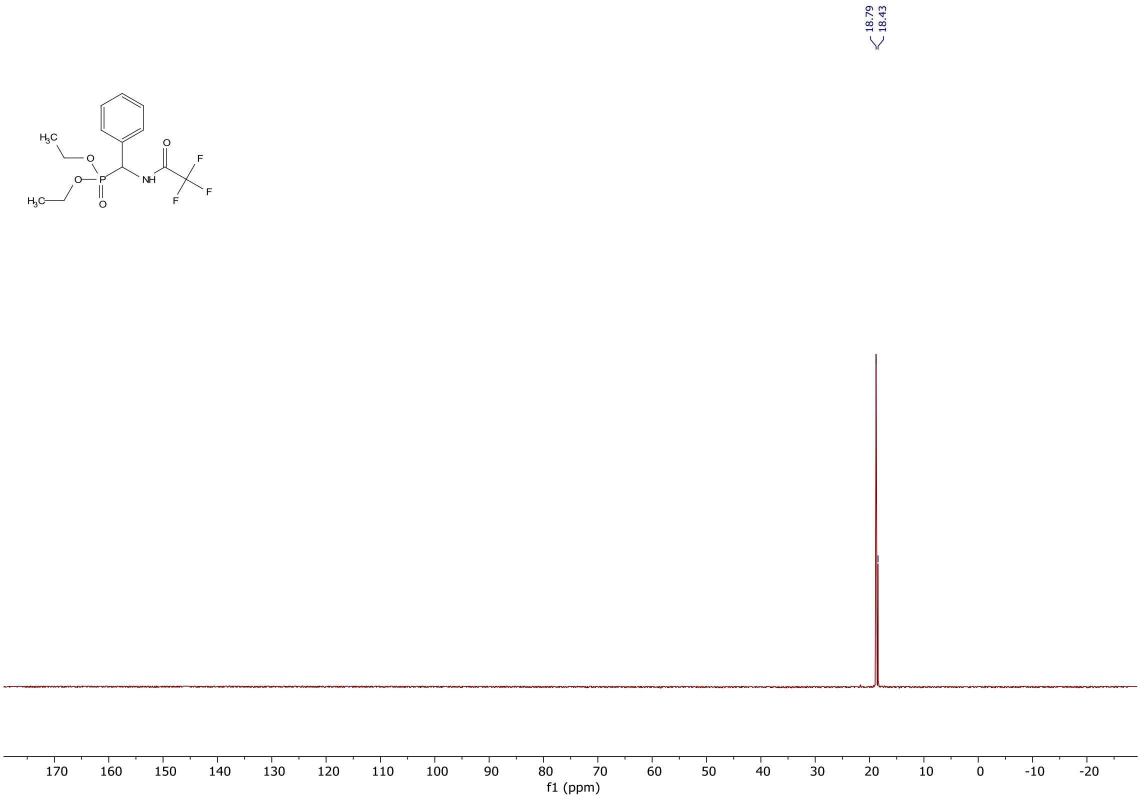
18.79
18.43
H3C
O
O
P
O
H3C
NH
O
F
F
F
170 160 150 140 130 120 110 100 90 80 70 60 50 40 30 20 10 0 -10 -20
f1 (ppm)

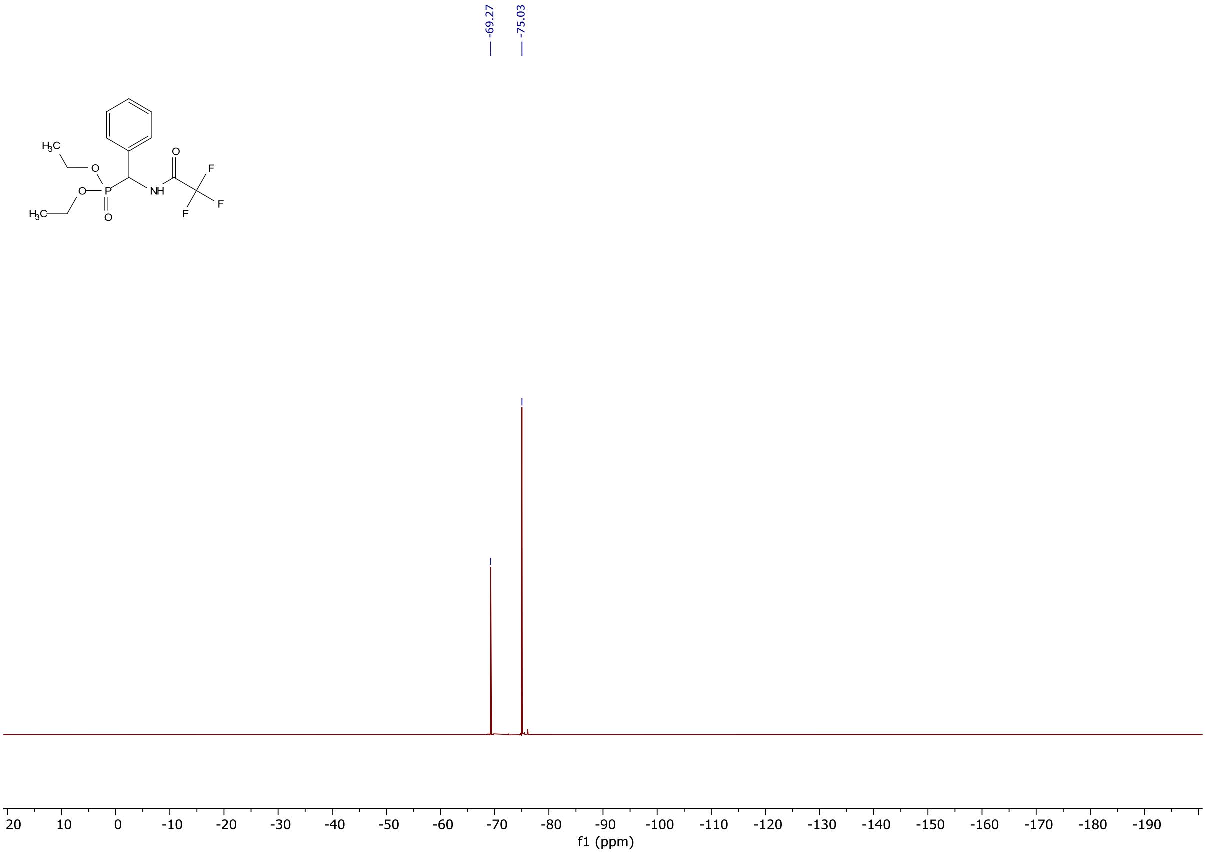
-69.27
-75.03
H3C
O
O
P
O
H3C
NH
O
F
F
F
20 10 0 -10 -20 -30 -40 -50 -60 -70 -80 -90 -100 -110 -120 -130 -140 -150 -160 -170 -180 -190
f1 (ppm)

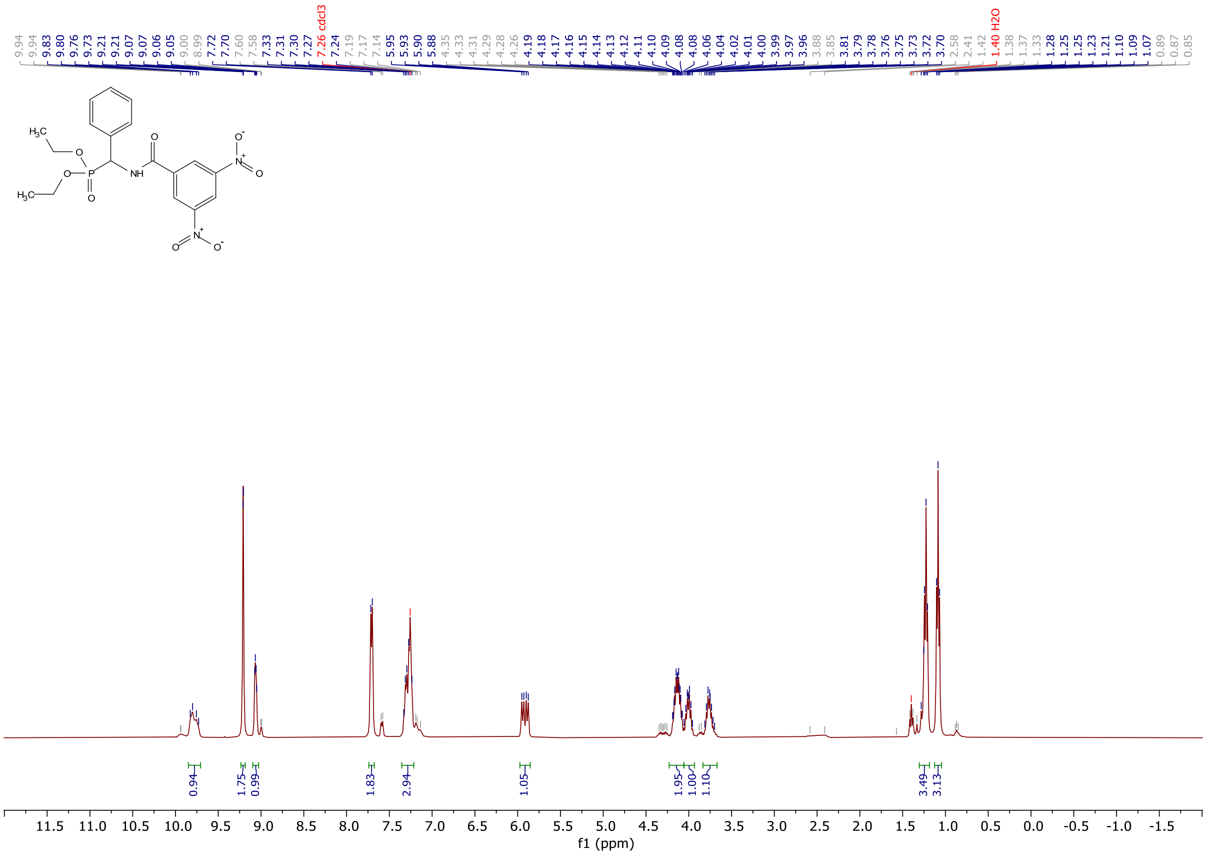

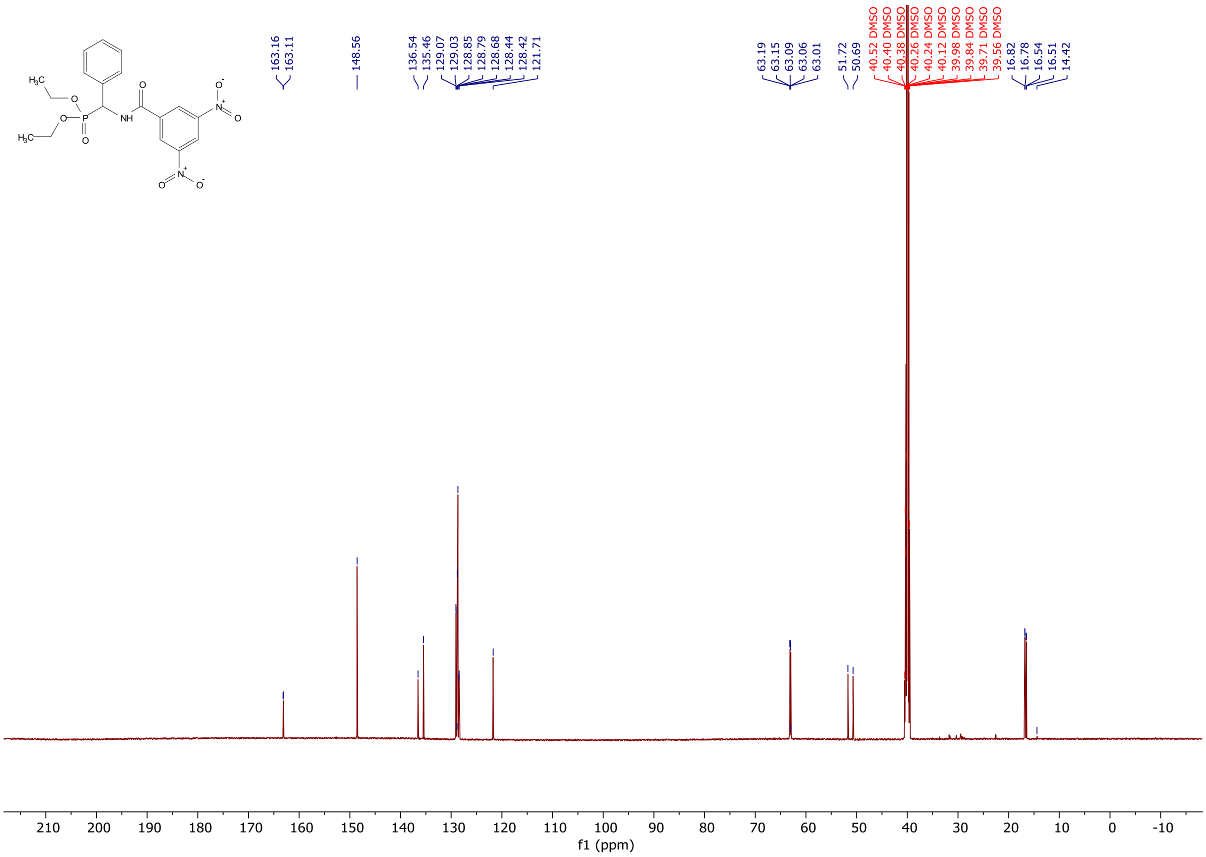

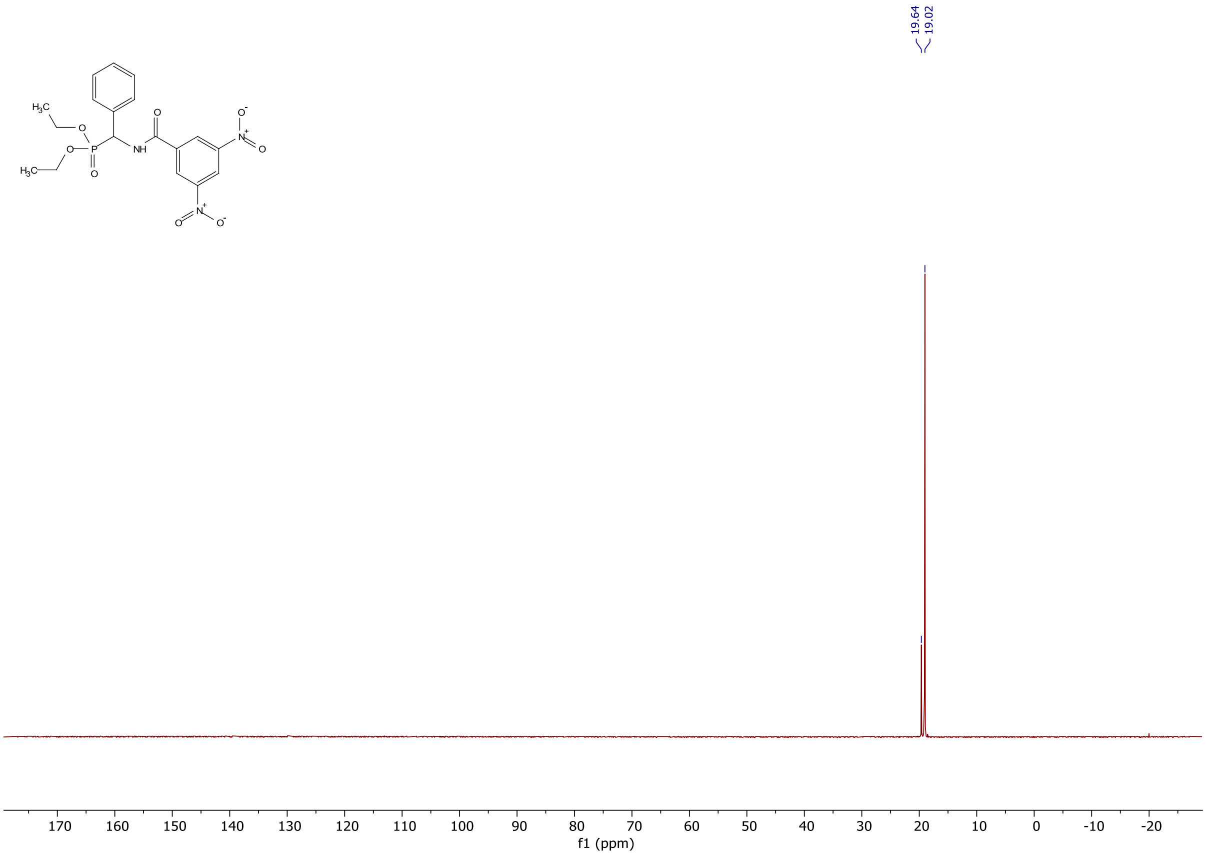
19.64
19.02
H3C
O
P
O
H3C
O
NH
O
N+
O-
O
N+
O
O-
170 160 150 140 130 120 110 100 90 80 70 60 50 40 30 20 10 0 -10 -20
f1 (ppm)

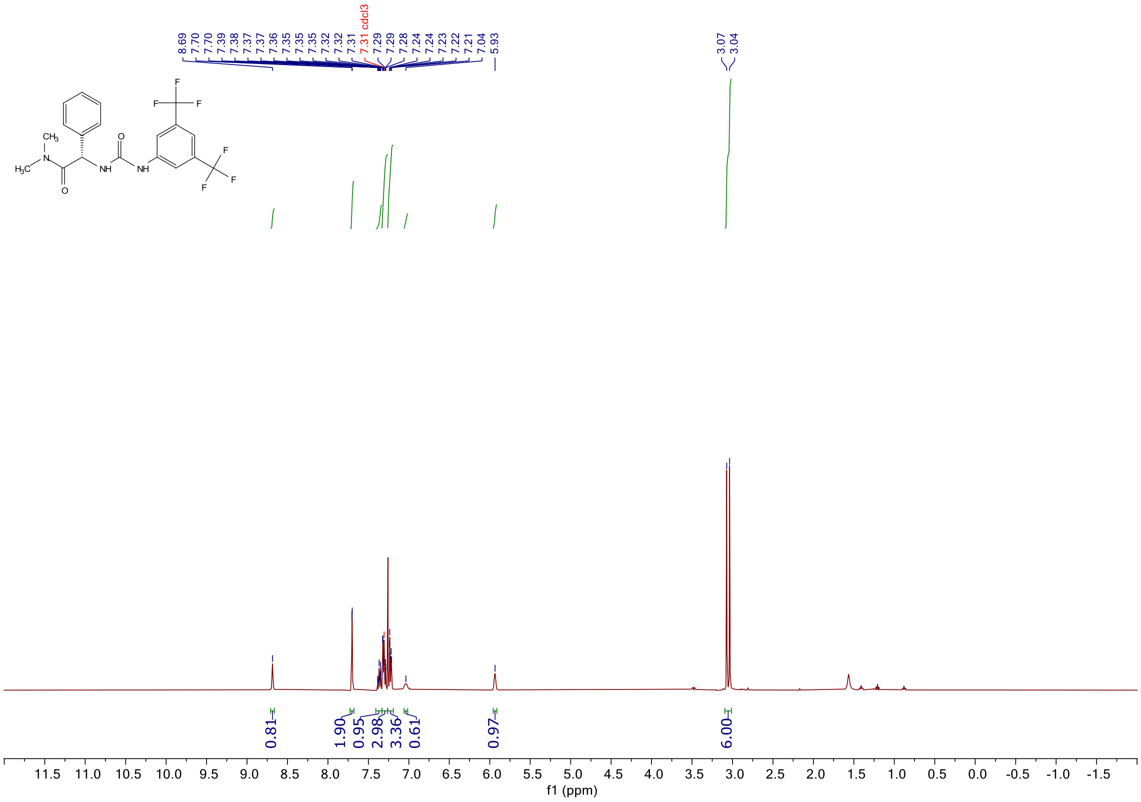
8.69 7.70 7.70 7.39 7.38 7.37 7.36 7.35 7.35 7.35 7.32 7.31
7.31 cdcl3
7.29 7.29 7.28 7.24 7.24 7.23 7.22 7.21 7.04 5.93
3.07
3.04
CH3
H3C
N
O
NH
O
NH
F
F
F
F
F
F
0.81
1.90
0.95
2.98
3.36
0.61
0.97
6.00
11.5 11.0 10.5 10.0 9.5 9.0 8.5 8.0 7.5 7.0 6.5 6.0 5.5 5.0 4.5 4.0 3.5 3.0 2.5 2.0 1.5 1.0 0.5 0.0 -0.5 -1.0 -1.5
f1 (ppm)

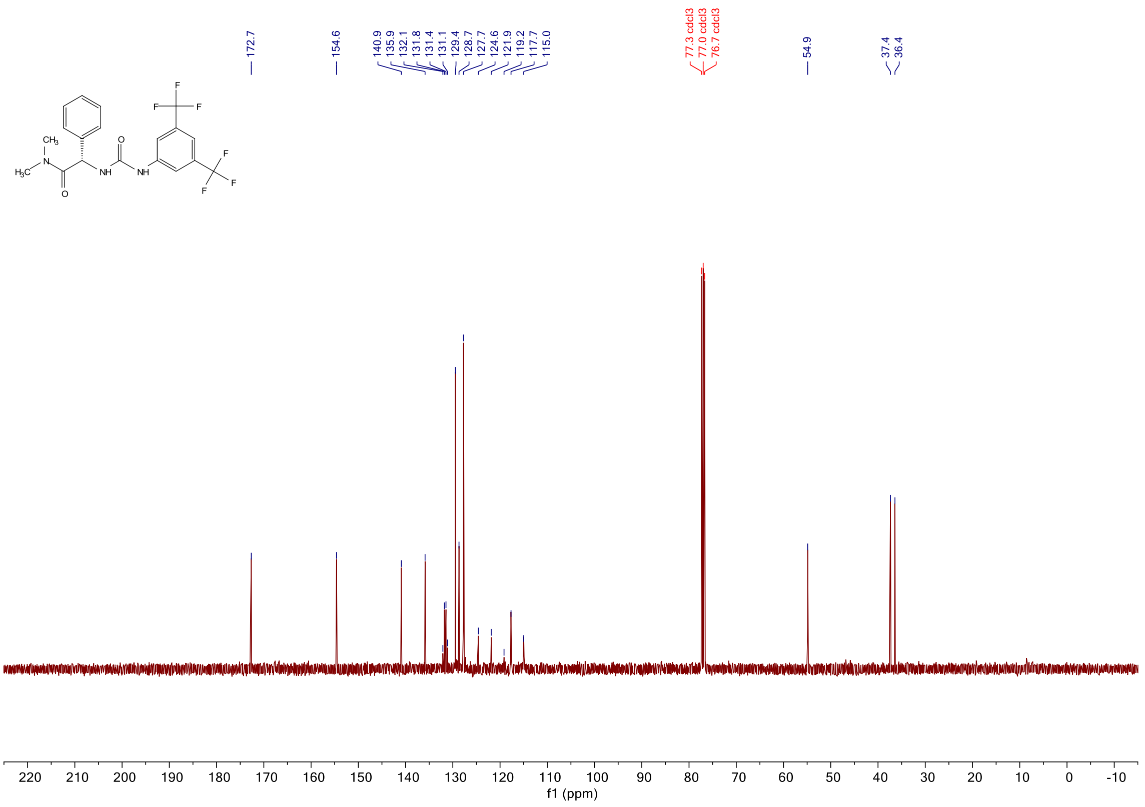
172.7
154.6
140.9
135.9
132.1
131.8
131.4
131.1
129.4
128.7
127.7
124.6
121.9
119.2
117.7
115.0
77.3 cdcl3
77.0 cdcl3
76.7 cdcl3
54.9
37.4
36.4
f1 (ppm)

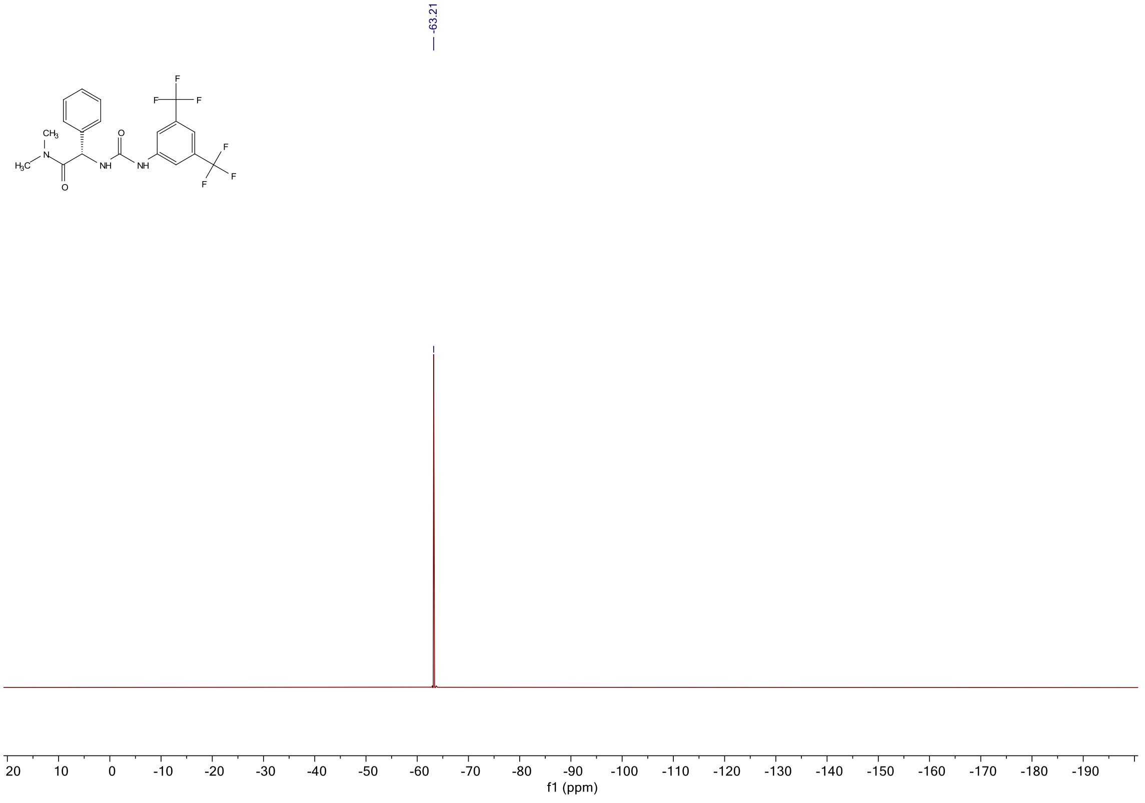
-63.21
f1 (ppm)

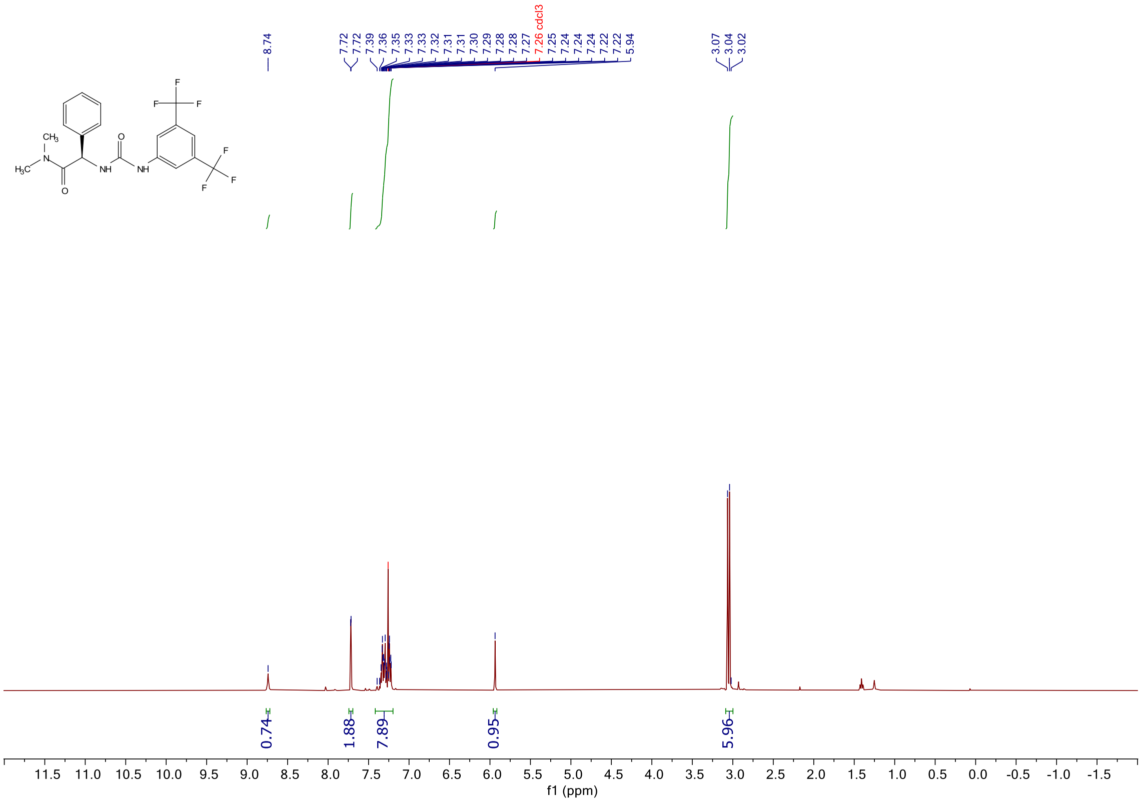

8.74
7.72
7.72
7.39
7.36
7.35
7.33
7.33
7.32
7.31
7.31
7.30
7.29
7.28
7.28
7.27
7.26 cdcl3
7.25
7.24
7.24
7.24
7.24
7.22
7.22
5.94
3.07
3.04
3.02
0.74
1.88
7.89
0.95
5.96
11.5 11.0 10.5 10.0 9.5 9.0 8.5 8.0 7.5 7.0 6.5 6.0 5.5 5.0 4.5 4.0 3.5 3.0 2.5 2.0 1.5 1.0 0.5 0.0 -0.5 -1.0 -1.5
f1 (ppm)

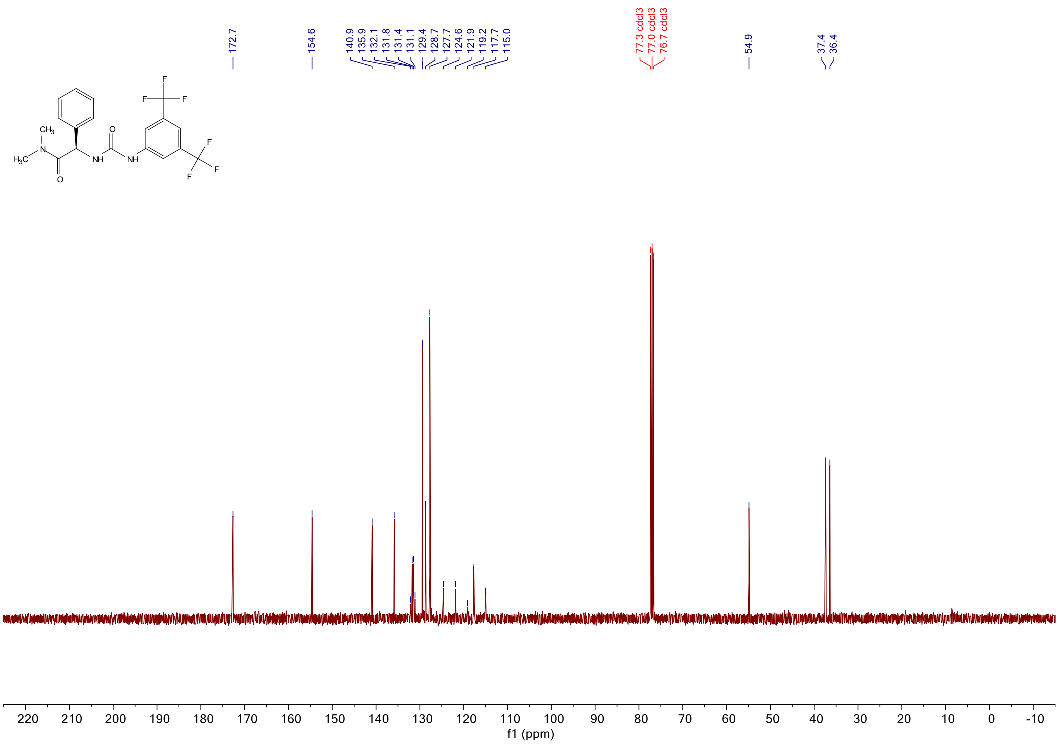

172.7
154.6
140.9
135.9
132.1
131.8
131.4
131.1
129.4
128.7
127.7
124.6
121.9
119.2
117.7
115.0
77.3 cdcl3
77.0 cdcl3
76.7 cdcl3
54.9
37.4
36.4
220 210 200 190 180 170 160 150 140 130 120 110 100 90 80 70 60 50 40 30 20 10 0 -10
f1 (ppm)

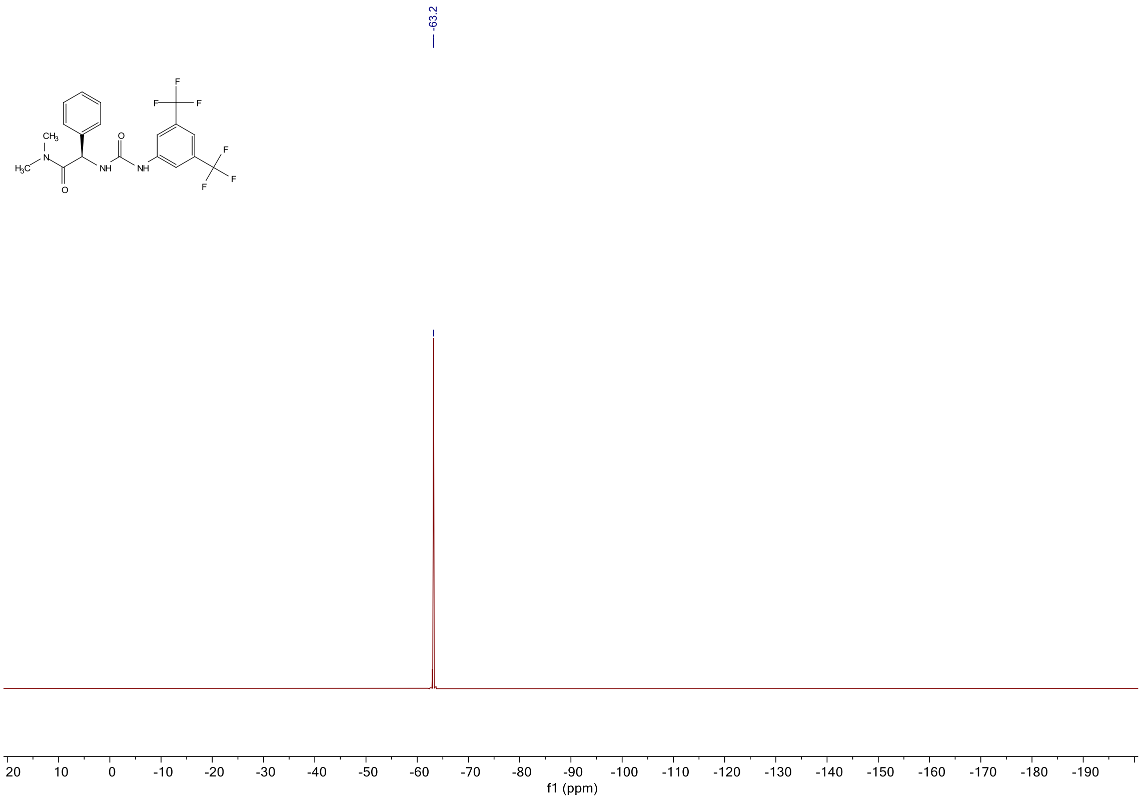

-63.2
F
F
F
F
F
F
CH3
H3C
N
O
NH
O
NH
20 10 0 -10 -20 -30 -40 -50 -60 -70 -80 -90 -100 -110 -120 -130 -140 -150 -160 -170 -180 -190
f1 (ppm)

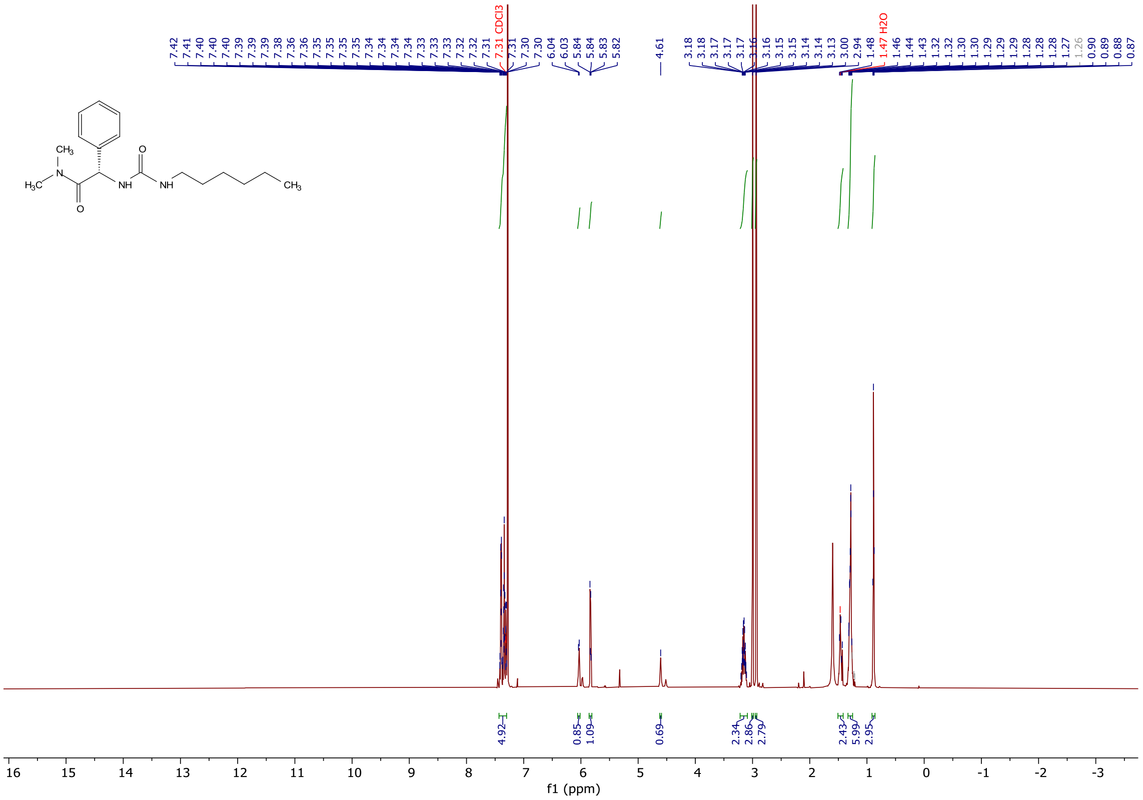

7.42 7.41 7.40 7.40 7.40 7.39 7.39 7.38 7.36 7.36 7.35 7.35 7.35 7.34 7.34 7.34 7.33 7.33 7.33 7.32 7.31 7.31 CDCl3 7.31 7.30 7.30 6.04 6.03 5.84 5.84 5.83 5.82 4.61 3.18 3.18 3.17 3.17 3.16 3.15 3.15 3.14 3.14 3.13 3.00 2.94 1.48 1.47 H2O 1.46 1.44 1.43 1.32 1.32 1.30 1.29 1.29 1.28 1.28 1.28 1.27 1.26 0.90 0.89 0.88 0.87
CH3
H3C
N
O
NH
O
NH
CH3
4.92
0.85
1.09
0.69
2.34
2.86
2.79
2.43
5.99
2.95
16 15 14 13 12 11 10 9 8 7 6 5 4 3 2 1 0 -1 -2 -3
f1 (ppm)

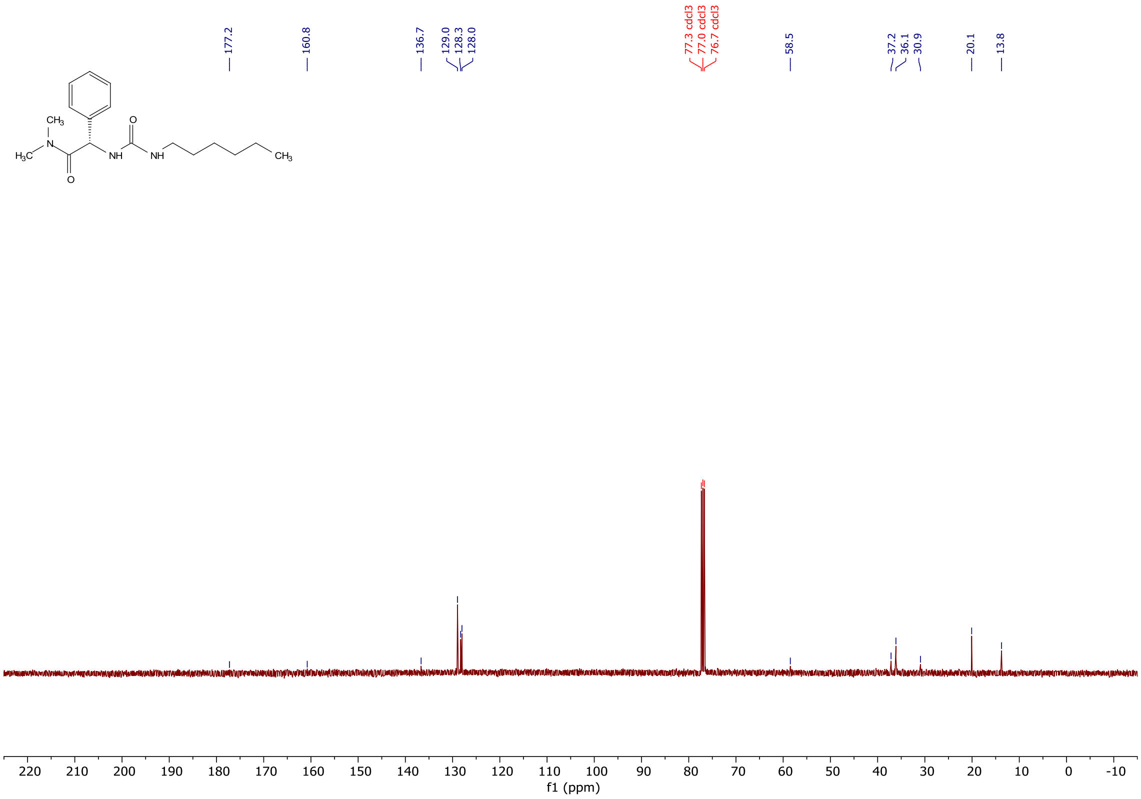
CH3
H3C
N
O
NH
O
NH
CH3
177.2
160.8
136.7
129.0
128.3
128.0
77.3 cdcl3
77.0 cdcl3
76.7 cdcl3
58.5
37.2
36.1
30.9
20.1
13.8
f1 (ppm)

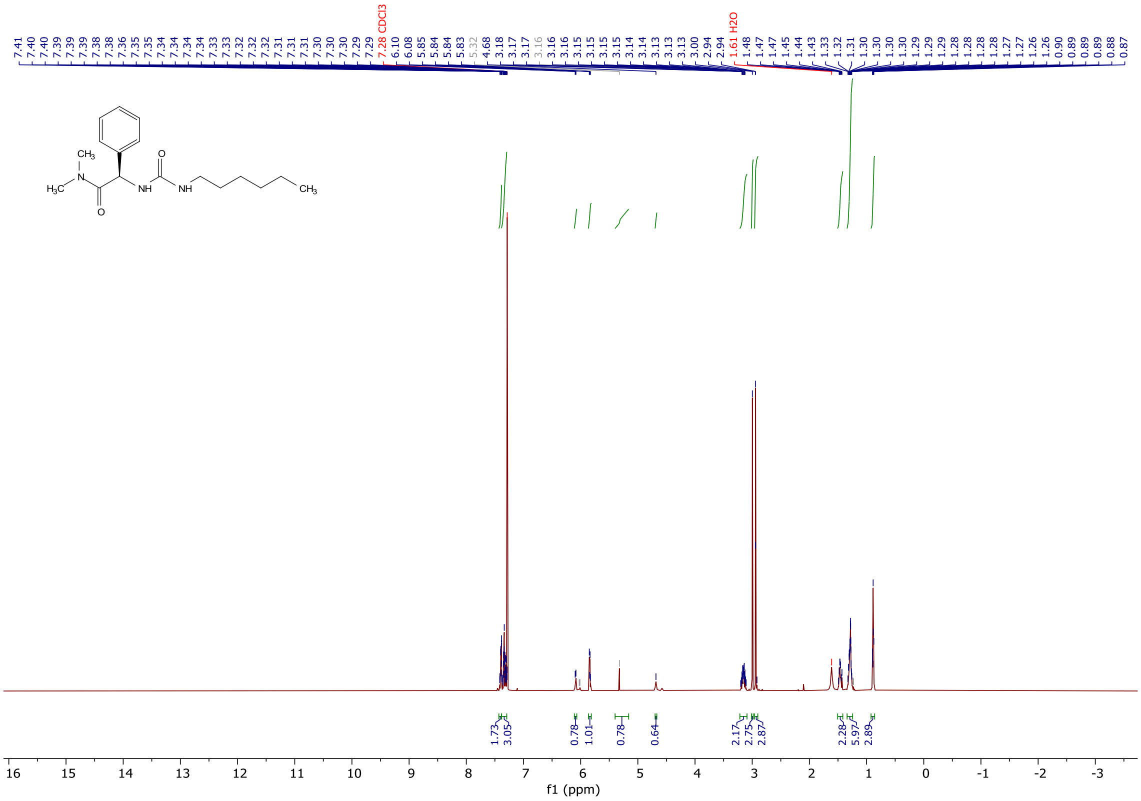
CH3
H3C
N
O
NH
O
NH
CH3
7.28 CDCl3
1.61 H2O
1.73
3.05
0.78
1.01
0.78
0.64
2.17
2.75
2.87
2.28
5.97
2.89
f1 (ppm)

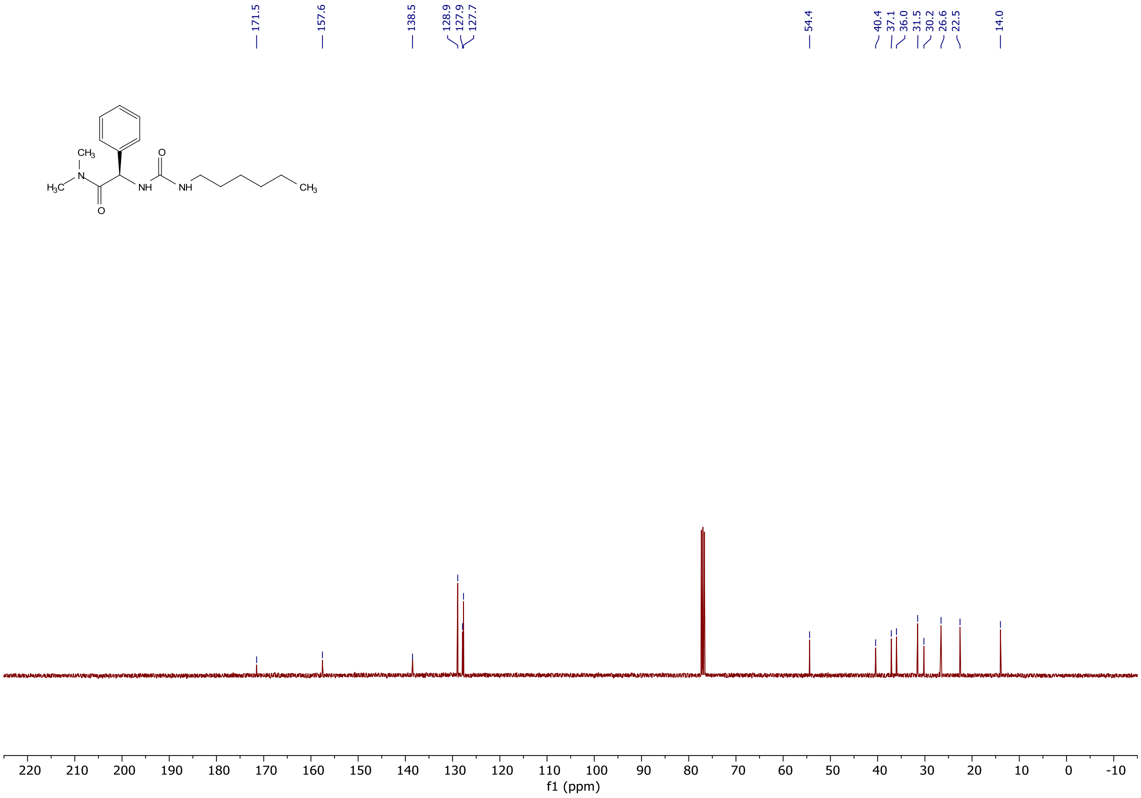
171.5
157.6
138.5
128.9
127.9
127.7
54.4
40.4
37.1
36.0
31.5
30.2
26.6
22.5
14.0
f1 (ppm)

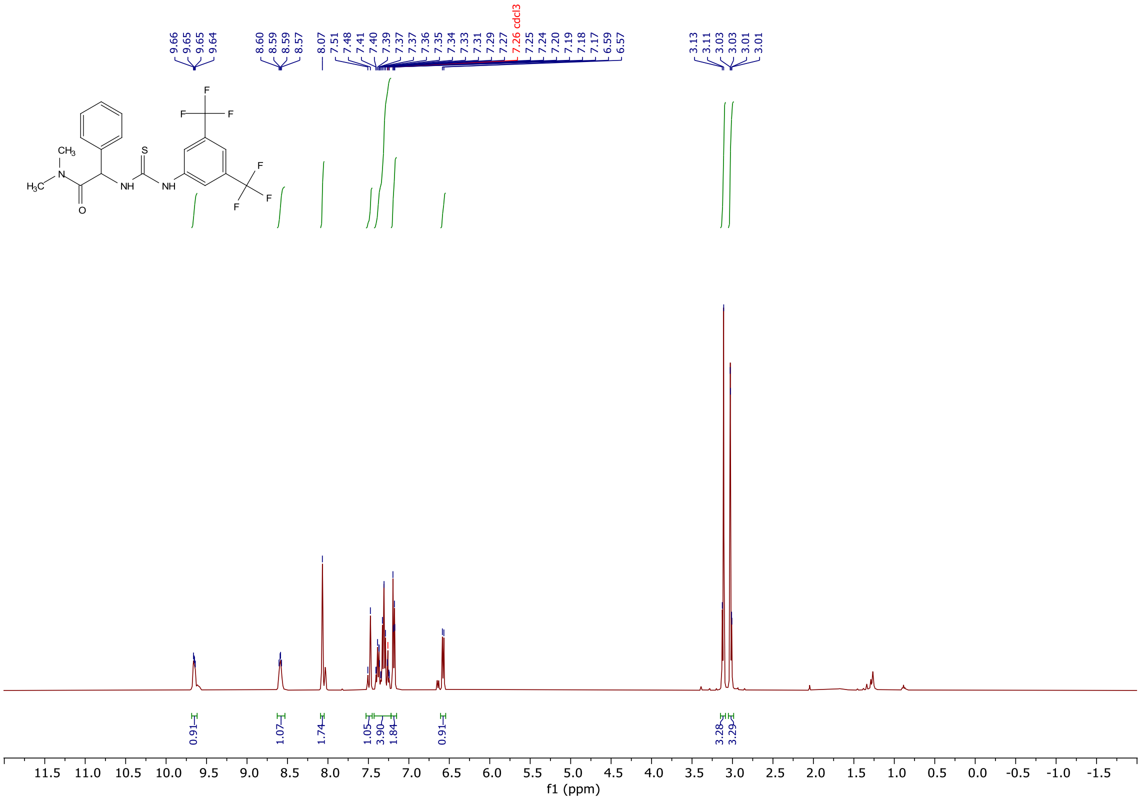
9.66
9.65
9.65
9.64
8.60
8.59
8.59
8.57
8.07
7.51
7.48
7.41
7.40
7.39
7.37
7.37
7.36
7.35
7.34
7.33
7.31
7.29
7.27
7.26 cdcl3
7.25
7.24
7.20
7.19
7.18
7.17
6.59
6.57
3.13
3.11
3.03
3.03
3.01
3.01
0.91
1.07
1.74
1.05
3.90
1.84
0.91
3.28
3.29
f1 (ppm)

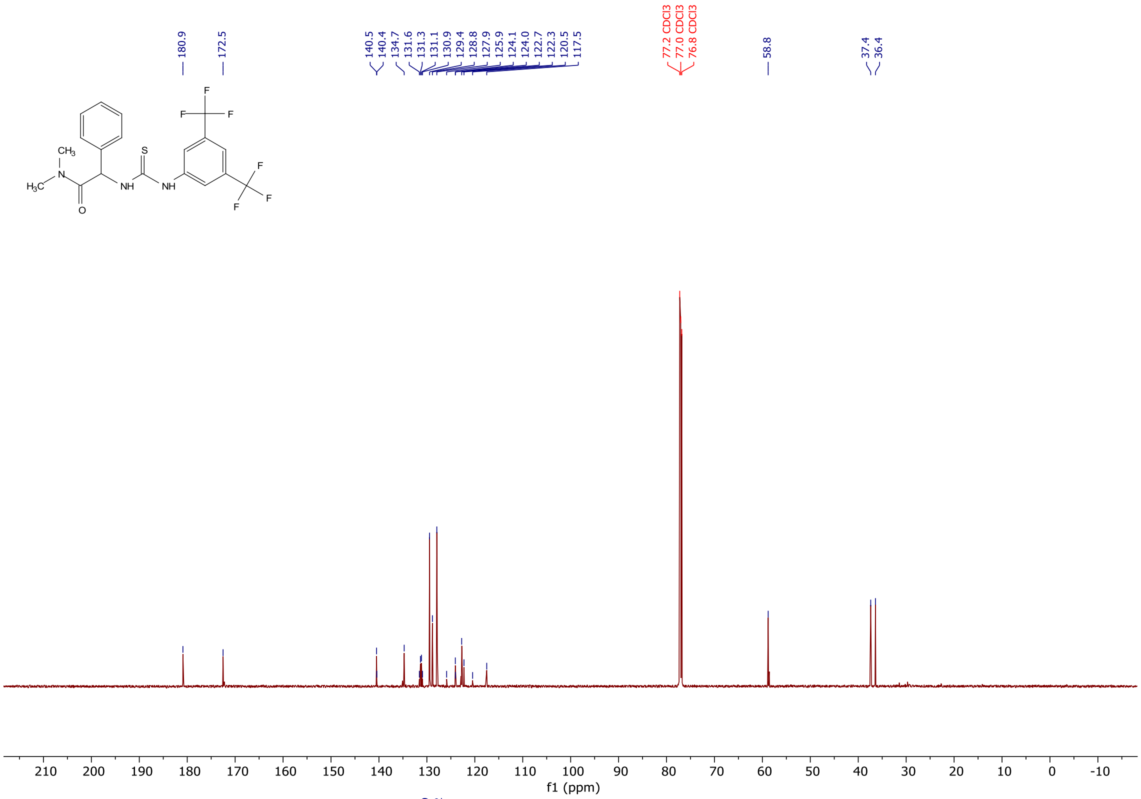
180.9
172.5
140.5
140.4
134.7
131.6
131.3
131.1
130.9
129.4
128.8
127.9
125.9
124.1
124.0
122.7
122.3
120.5
117.5
77.2 CDCl3
77.0 CDCl3
76.8 CDCl3
58.8
37.4
36.4
f1 (ppm)

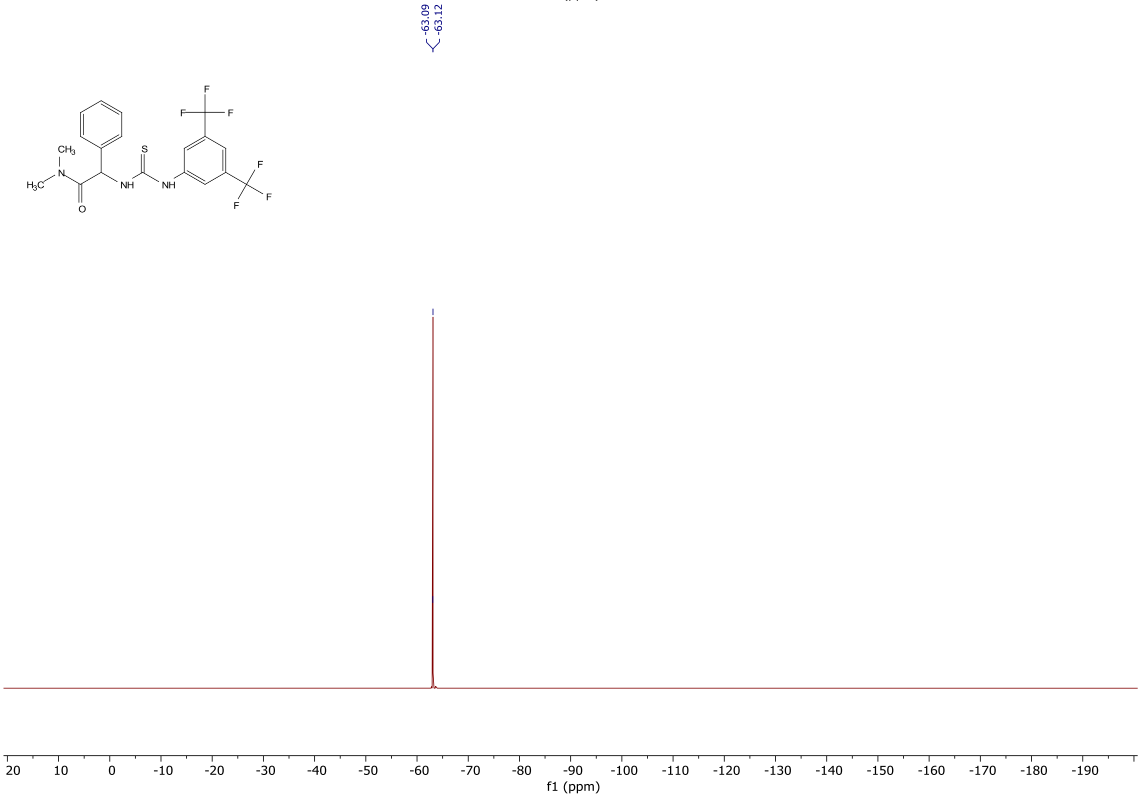
-63.09
-63.12
f1 (ppm)

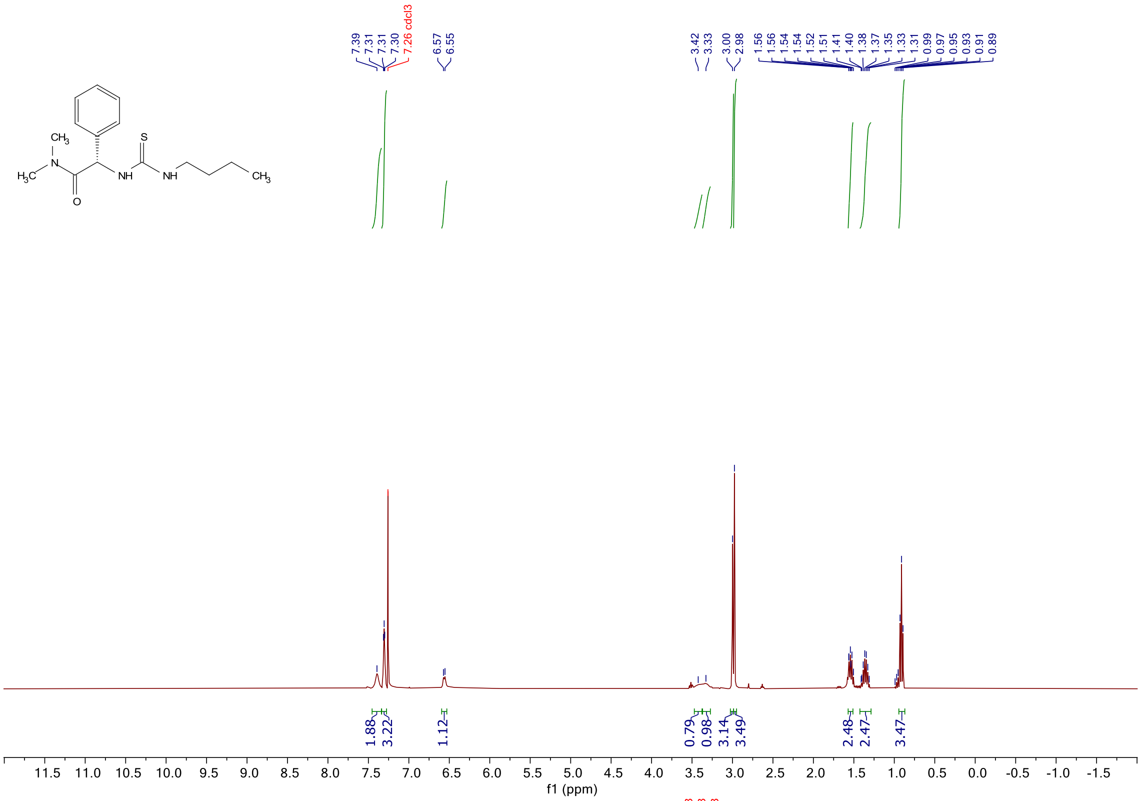
7.39
7.31
7.31
7.30
7.26 cdcl3
6.57
6.55
3.42
3.33
3.00
2.98
1.56
1.56
1.54
1.54
1.52
1.51
1.41
1.40
1.38
1.37
1.35
1.33
1.31
0.99
0.97
0.95
0.93
0.91
0.89
CH3
H3C
N
O
NH
S
NH
CH3
1.88
3.22
1.12
0.79
0.98
3.14
3.49
2.48
2.47
3.47
11.5 11.0 10.5 10.0 9.5 9.0 8.5 8.0 7.5 7.0 6.5 6.0 5.5 5.0 4.5 4.0 3.5 3.0 2.5 2.0 1.5 1.0 0.5 0.0 -0.5 -1.0 -1.5
f1 (ppm)

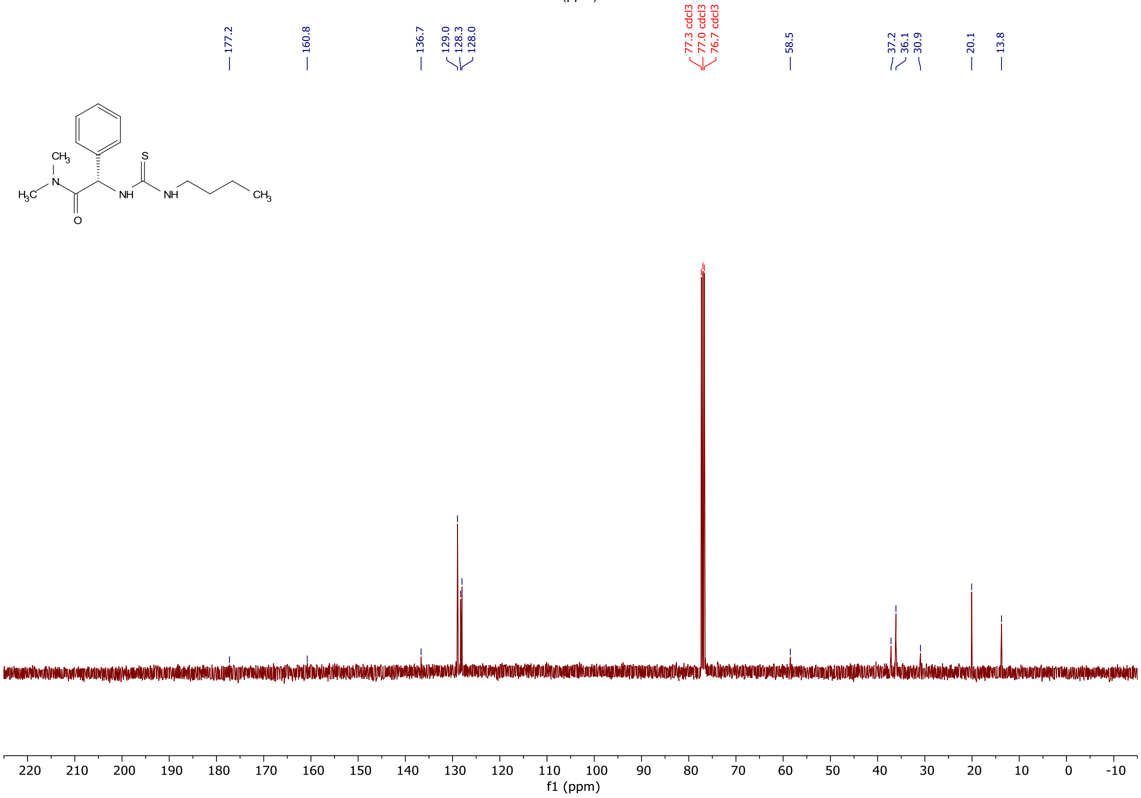
177.2
160.8
136.7
129.0
128.3
128.0
77.3 cdcl3
77.0 cdcl3
76.7 cdcl3
58.5
37.2
36.1
30.9
20.1
13.8
CH3
H3C
N
O
NH
S
NH
CH3
220 210 200 190 180 170 160 150 140 130 120 110 100 90 80 70 60 50 40 30 20 10 0 -10
f1 (ppm)

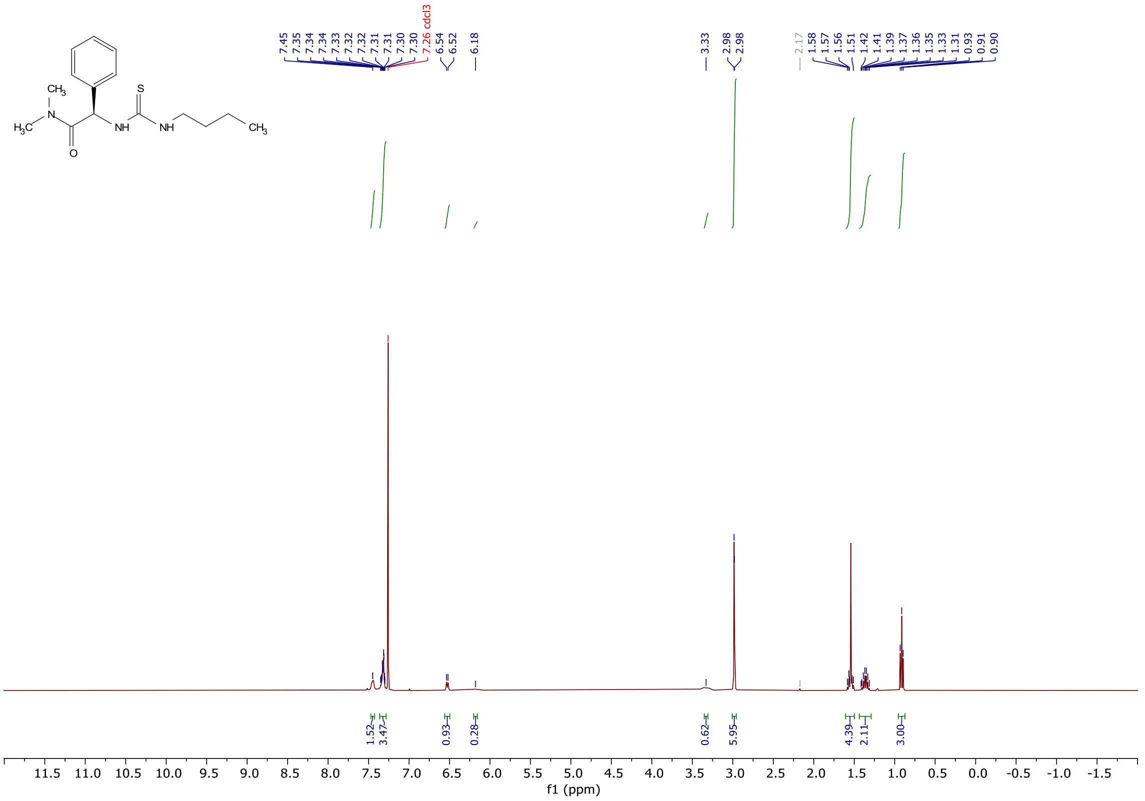
7.45
7.35
7.34
7.34
7.33
7.33
7.32
7.32
7.31
7.31
7.30
7.30
7.26 cdcl3
6.54
6.52
6.18
3.33
2.98
2.98
2.17
1.58
1.57
1.56
1.51
1.42
1.41
1.39
1.37
1.36
1.35
1.33
1.31
0.93
0.91
0.90
1.52
3.47
0.93
0.28
0.62
5.95
4.39
2.11
3.00
f1 (ppm)

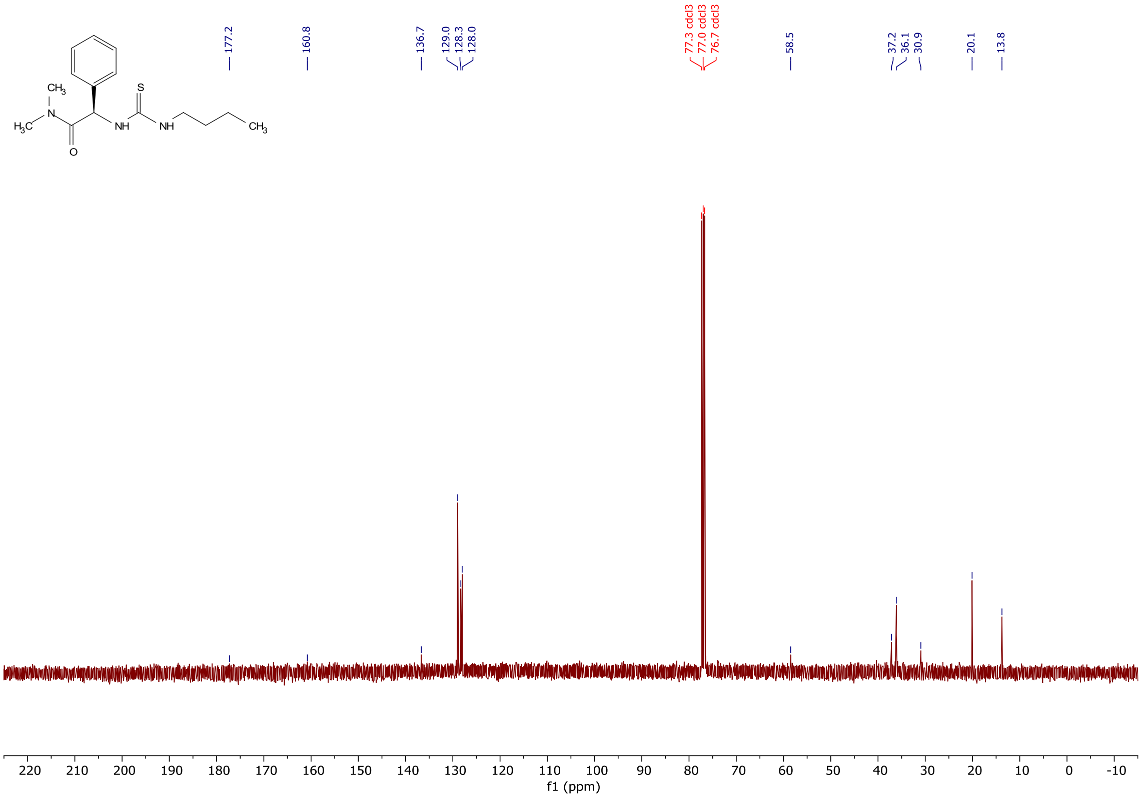
177.2
160.8
136.7
129.0
128.3
128.0
77.3 cdcl3
77.0 cdcl3
76.7 cdcl3
58.5
37.2
36.1
30.9
20.1
13.8
f1 (ppm)

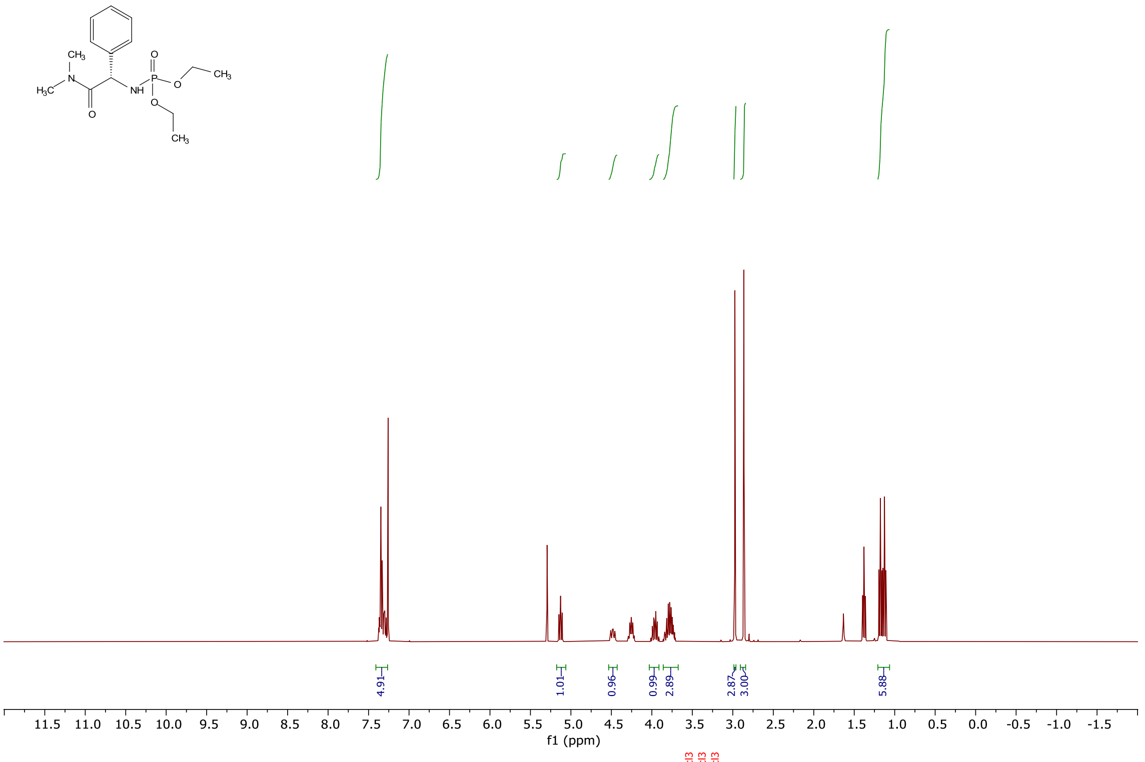
4.91
1.01
0.96
0.99
2.89
2.87
3.00
5.88
f1 (ppm)

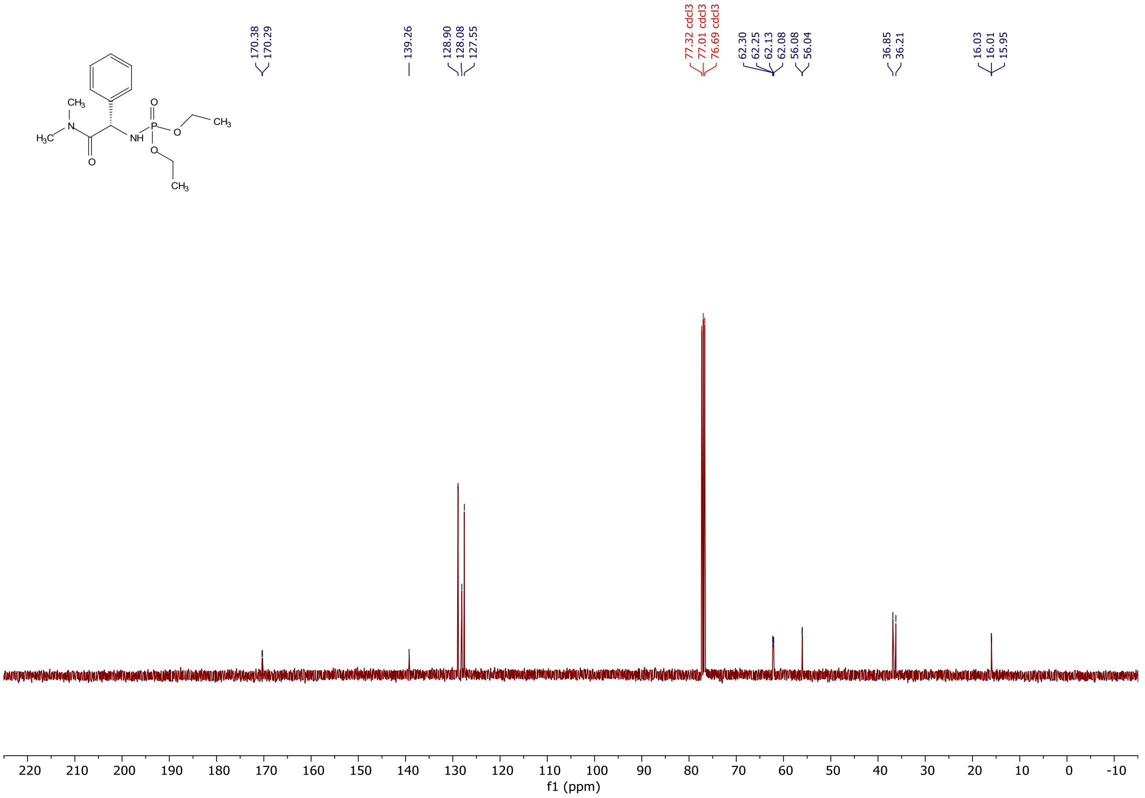
170.38
170.29
139.26
128.90
128.08
127.55
77.32 cdcl3
77.01 cdcl3
76.69 cdcl3
62.30
62.25
62.13
62.08
56.08
56.04
36.85
36.21
16.03
16.01
15.95
f1 (ppm)

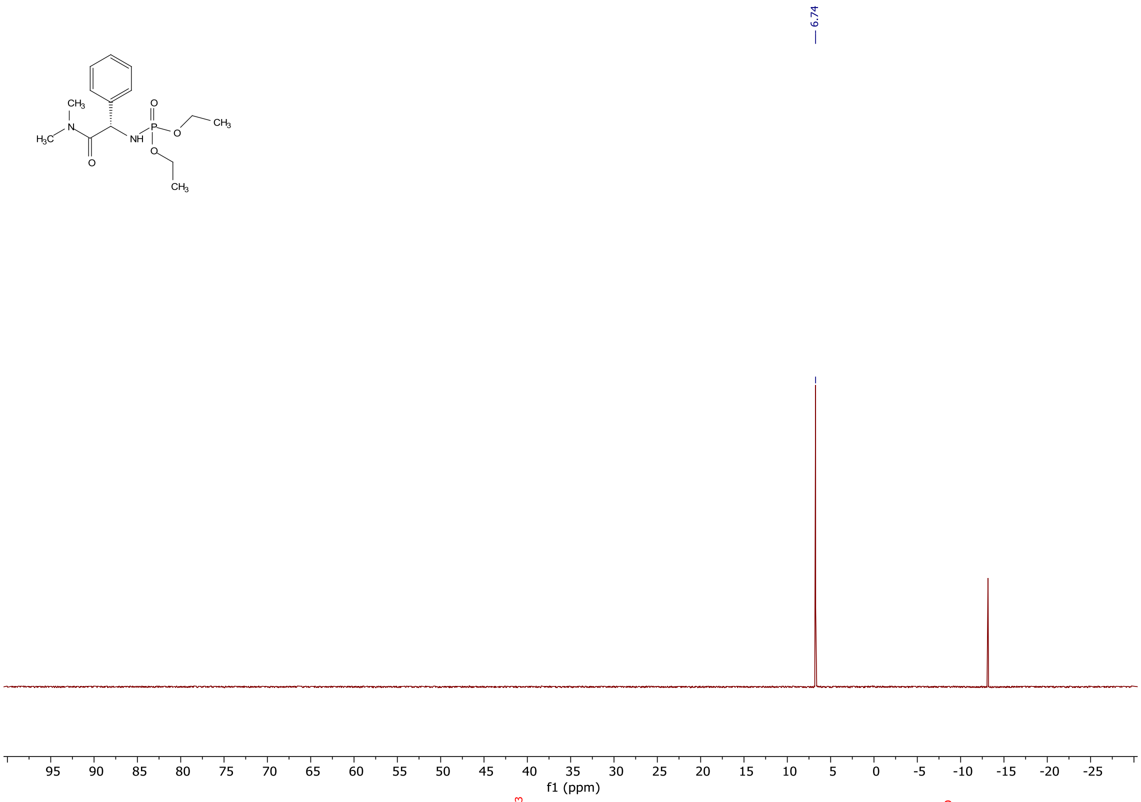

6.74
95 90 85 80 75 70 65 60 55 50 45 40 35 30 25 20 15 10 5 0 -5 -10 -15 -20 -25
f1 (ppm)

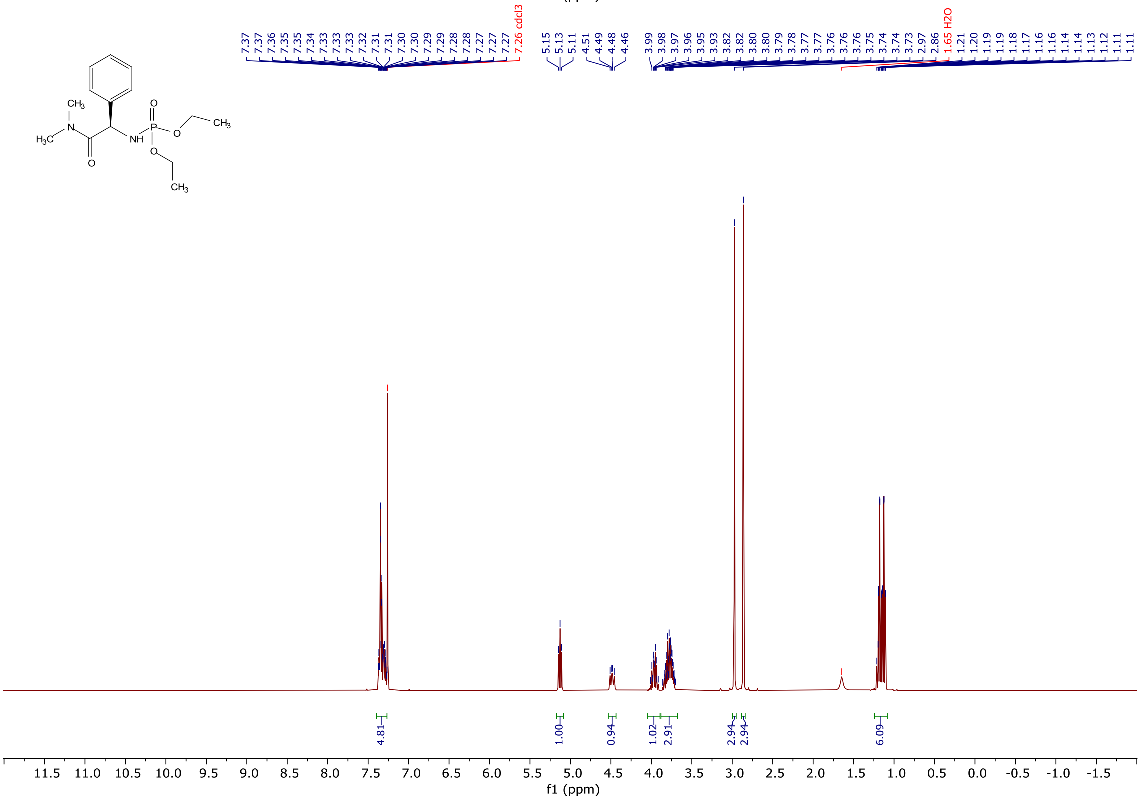

7.37 7.37 7.36 7.35 7.35 7.34 7.33 7.33 7.33 7.32 7.31 7.31 7.30 7.29 7.29 7.28 7.28 7.27 7.27 7.27 7.26 cdcl3
5.15 5.13 5.11 4.51 4.49 4.48 4.46
3.99 3.98 3.97 3.96 3.95 3.93 3.82 3.82 3.80 3.79 3.78 3.77 3.77 3.76 3.76 3.75 3.74 3.74 3.73 2.97 2.86 1.65 H2O 1.21 1.20 1.19 1.19 1.18 1.17 1.16 1.16 1.14 1.14 1.13 1.13 1.12 1.11 1.11
4.81 1.00 0.94 1.02 2.91 2.94 2.94 6.09
11.5 11.0 10.5 10.0 9.5 9.0 8.5 8.0 7.5 7.0 6.5 6.0 5.5 5.0 4.5 4.0 3.5 3.0 2.5 2.0 1.5 1.0 0.5 0.0 -0.5 -1.0 -1.5
f1 (ppm)

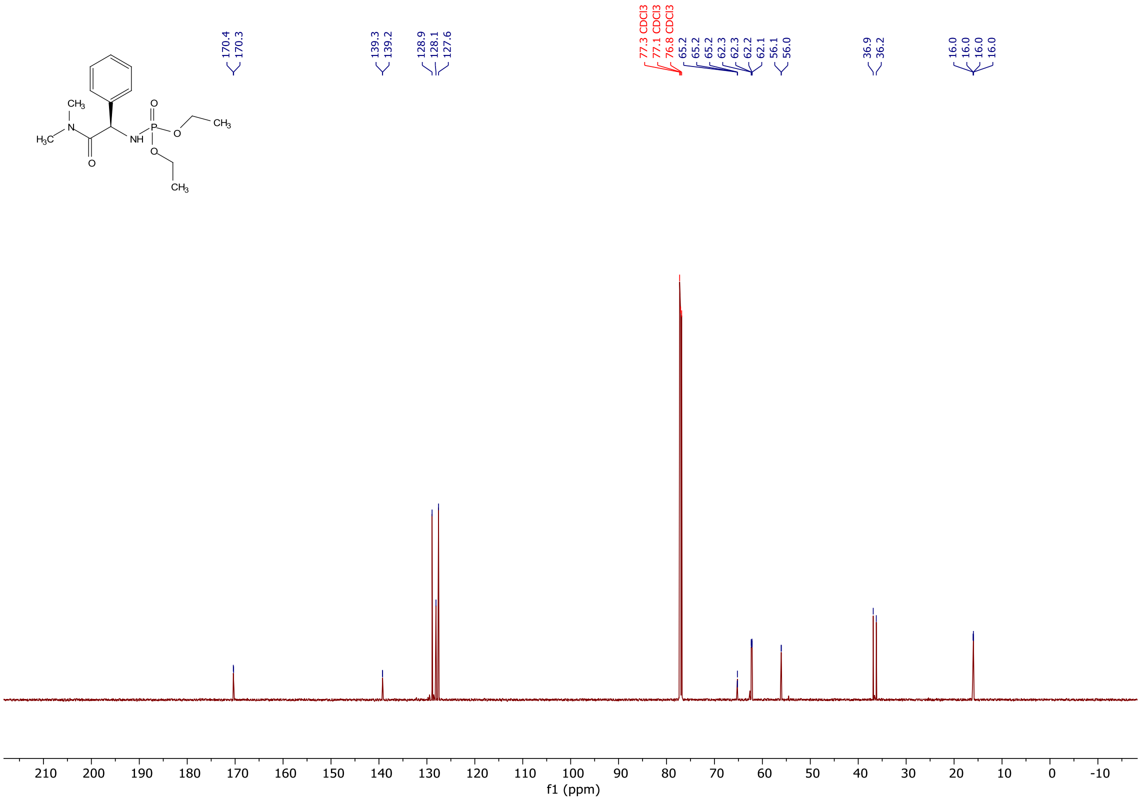
170.4
170.3
139.3
139.2
128.9
128.1
127.6
77.3 CDCl3
77.1 CDCl3
76.8 CDCl3
65.2
65.2
65.2
62.3
62.3
62.2
62.1
56.1
56.0
36.9
36.2
16.0
16.0
16.0
16.0
f1 (ppm)

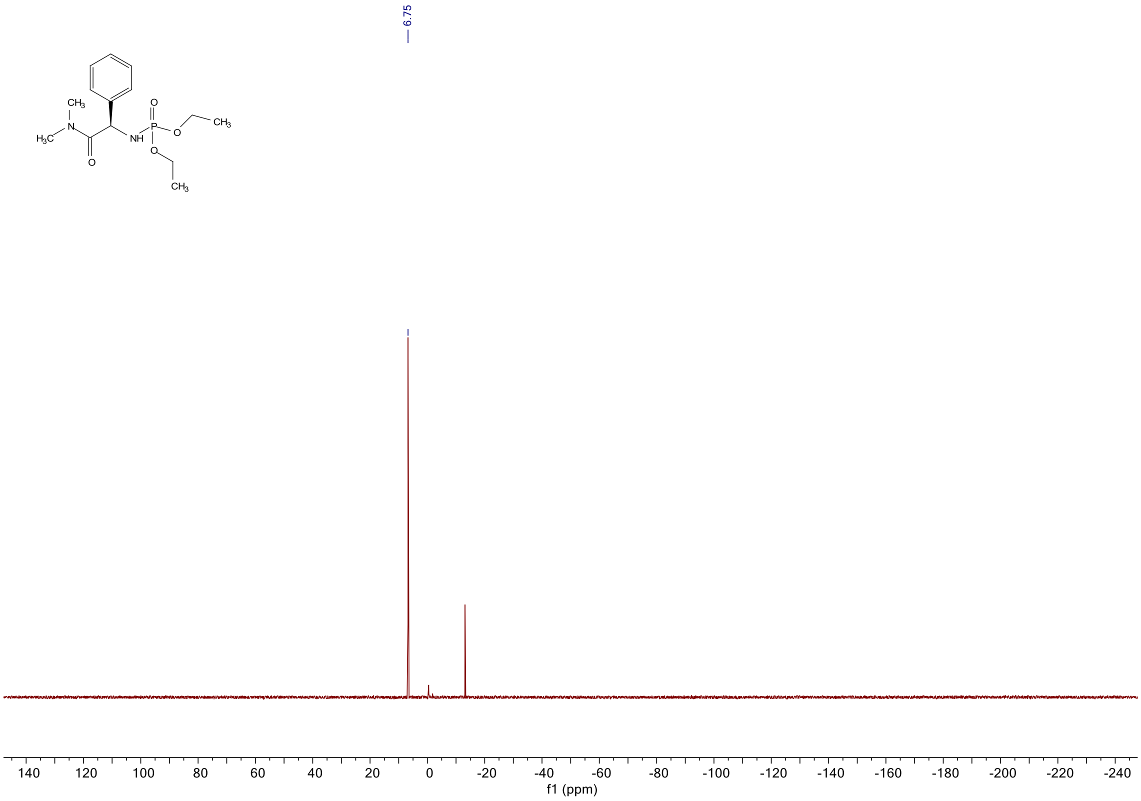
6.75
f1 (ppm)

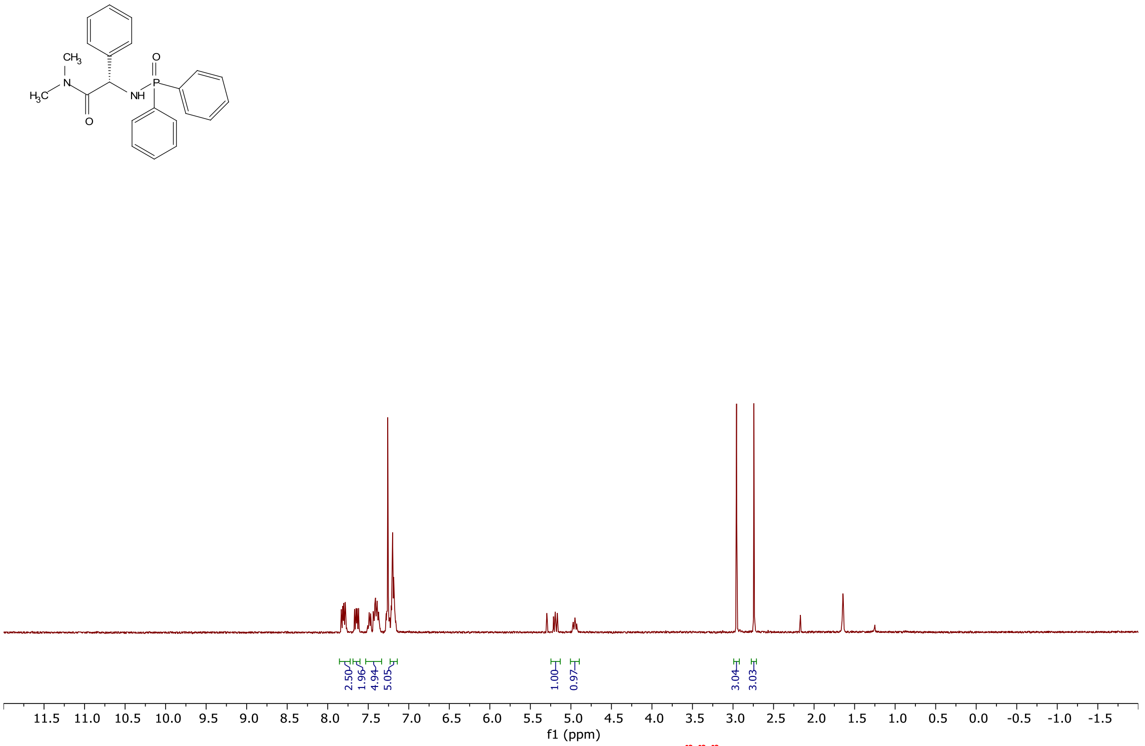
CH3
N
H3C
O
NH
P
O
2.50
1.96
4.94
5.05
1.00
0.97
3.04
3.03
11.5 11.0 10.5 10.0 9.5 9.0 8.5 8.0 7.5 7.0 6.5 6.0 5.5 5.0 4.5 4.0 3.5 3.0 2.5 2.0 1.5 1.0 0.5 0.0 -0.5 -1.0 -1.5
f1 (ppm)

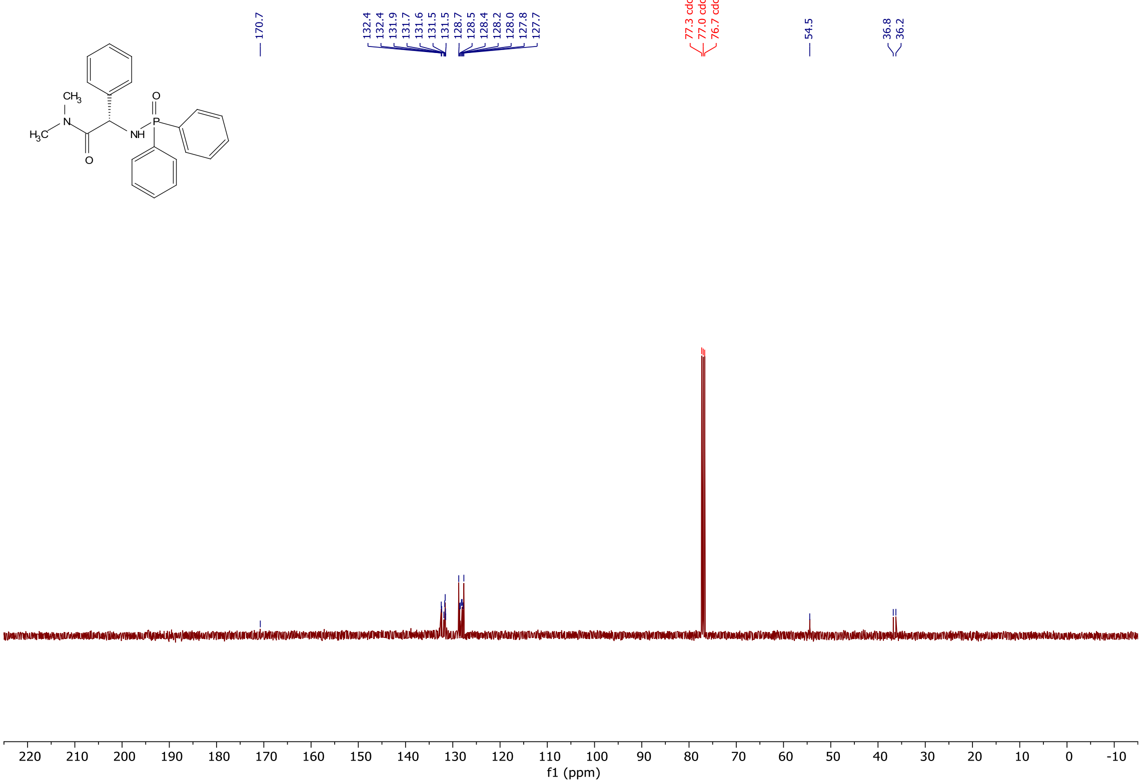
170.7
132.4
132.4
131.9
131.7
131.6
131.5
131.5
128.7
128.5
128.4
128.2
128.0
127.8
127.7
77.3 cdcl3
77.0 cdcl3
76.7 cdcl3
54.5
36.8
36.2
CH3
N
H3C
O
NH
P
O
220 210 200 190 180 170 160 150 140 130 120 110 100 90 80 70 60 50 40 30 20 10 0 -10
f1 (ppm)

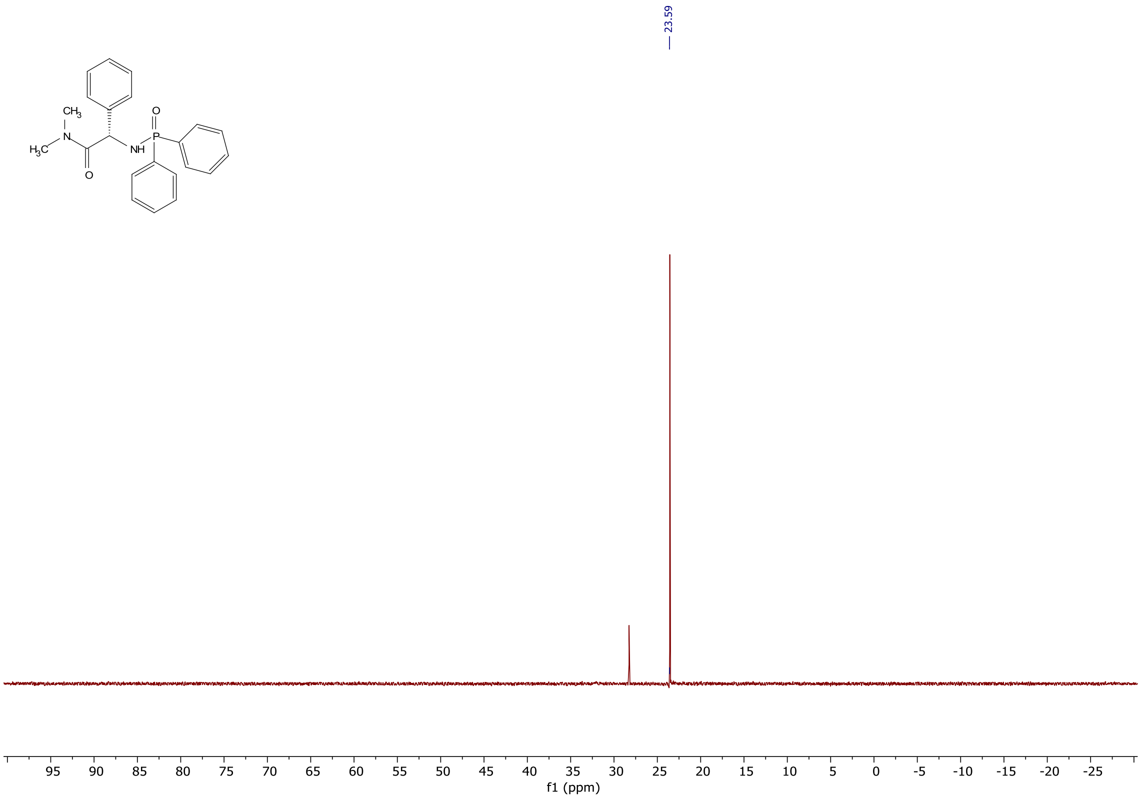

23.59
95 90 85 80 75 70 65 60 55 50 45 40 35 30 25 20 15 10 5 0 -5 -10 -15 -20 -25
f1 (ppm)

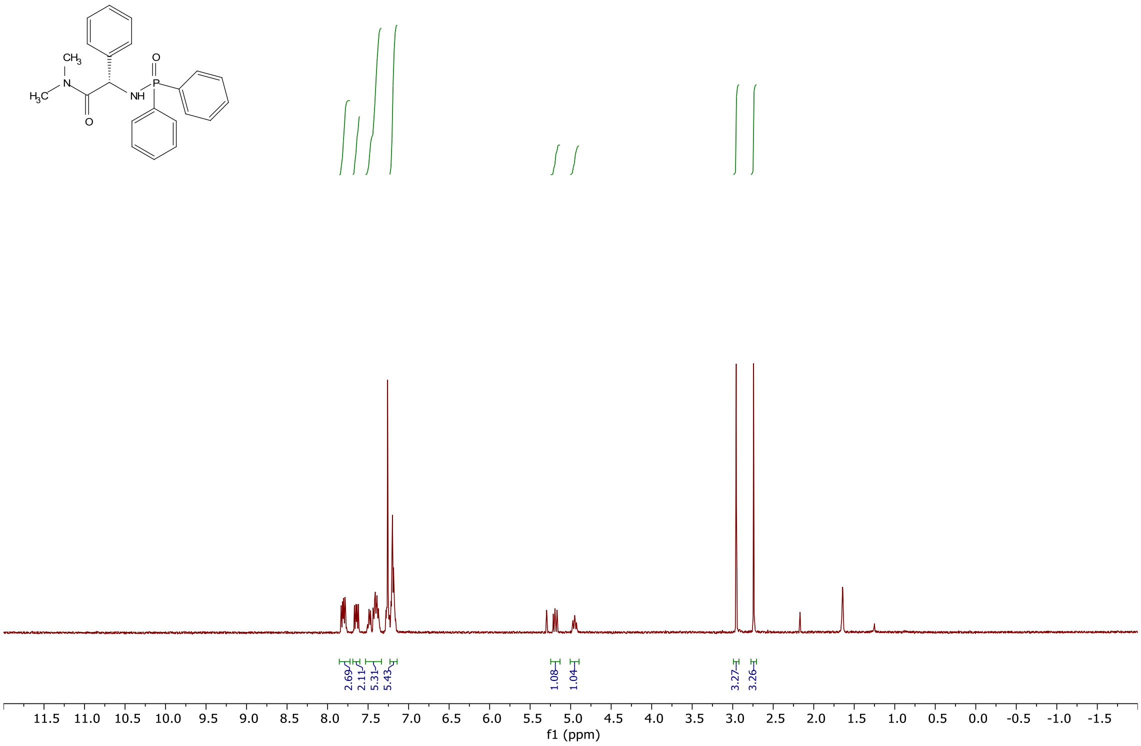

2.69
2.11
5.31
5.43
1.08
1.04
3.27
3.26
11.5 11.0 10.5 10.0 9.5 9.0 8.5 8.0 7.5 7.0 6.5 6.0 5.5 5.0 4.5 4.0 3.5 3.0 2.5 2.0 1.5 1.0 0.5 0.0 -0.5 -1.0 -1.5
f1 (ppm)

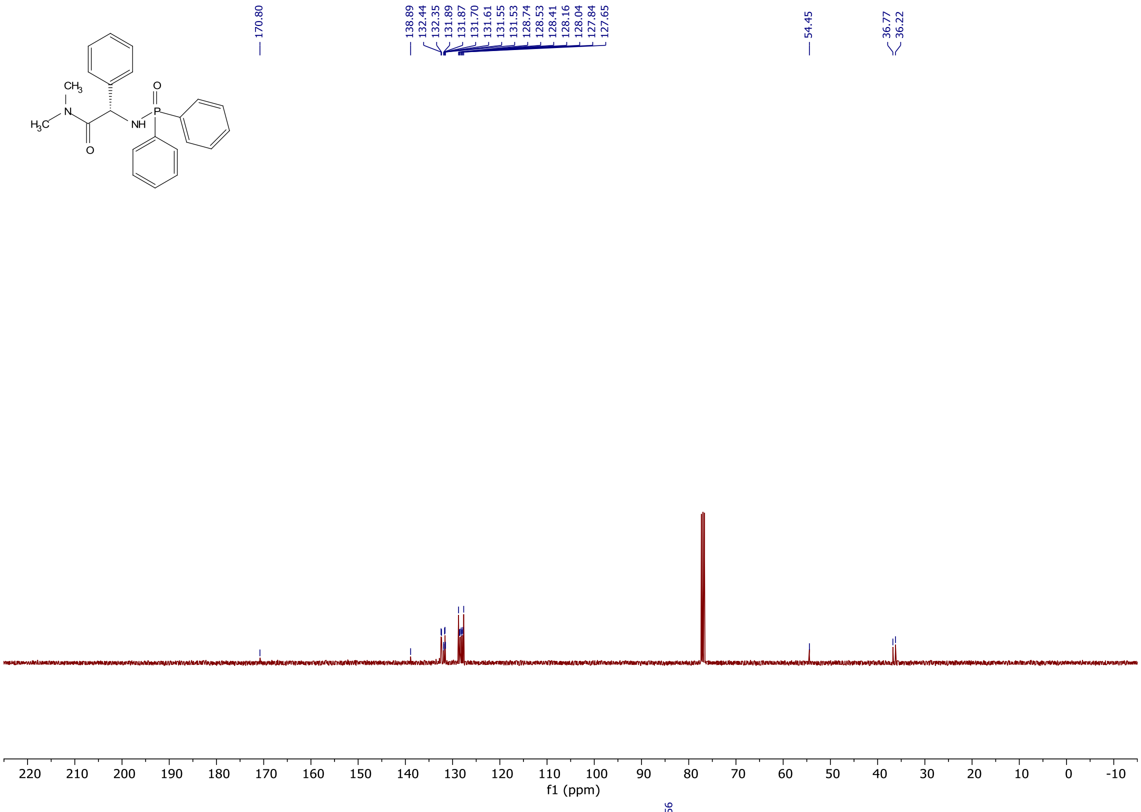
170.80
138.89
132.44
132.35
131.89
131.87
131.70
131.61
131.55
131.53
128.74
128.53
128.41
128.16
128.04
127.84
127.65
54.45
36.77
36.22
f1 (ppm)

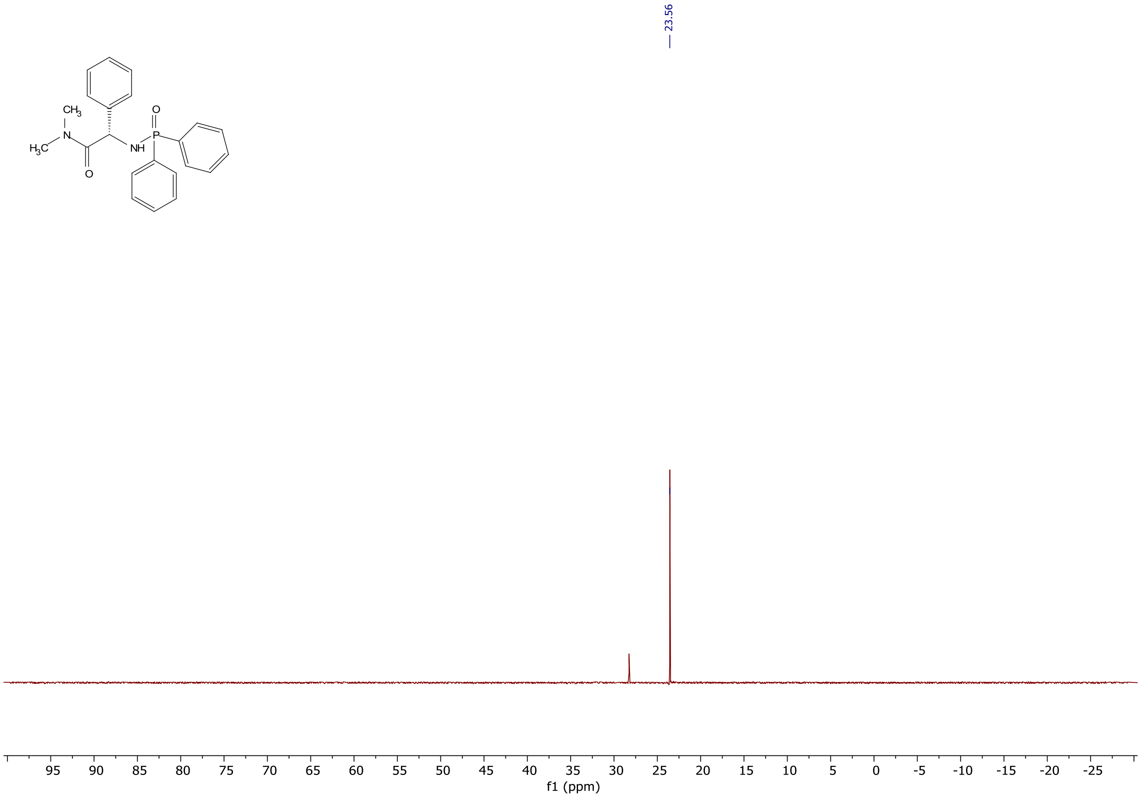
23.56
f1 (ppm)

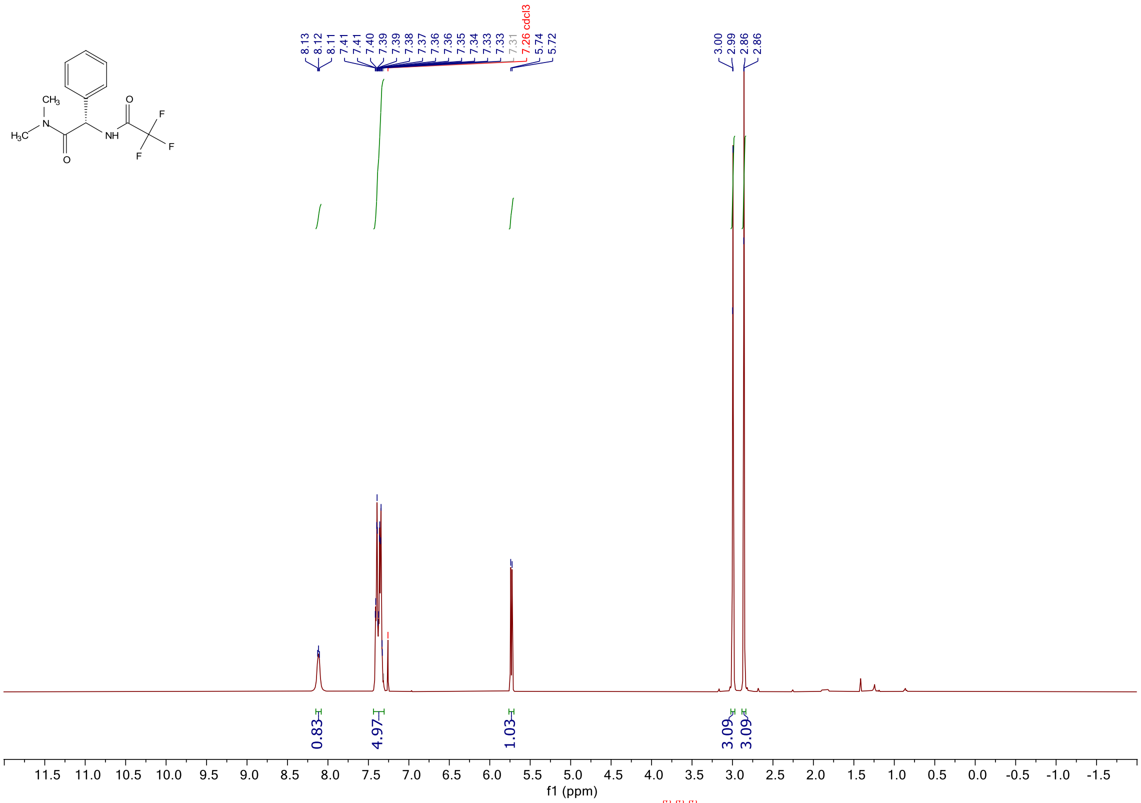
CH3
H3C
N
O
NH
O
F
F
F
8.13
8.12
8.11
7.41
7.41
7.40
7.39
7.39
7.39
7.38
7.37
7.36
7.36
7.35
7.34
7.33
7.33
7.31
7.26 cdcl3
5.74
5.72
3.00
2.99
2.86
2.86
0.83
4.97
1.03
3.09
3.09
f1 (ppm)

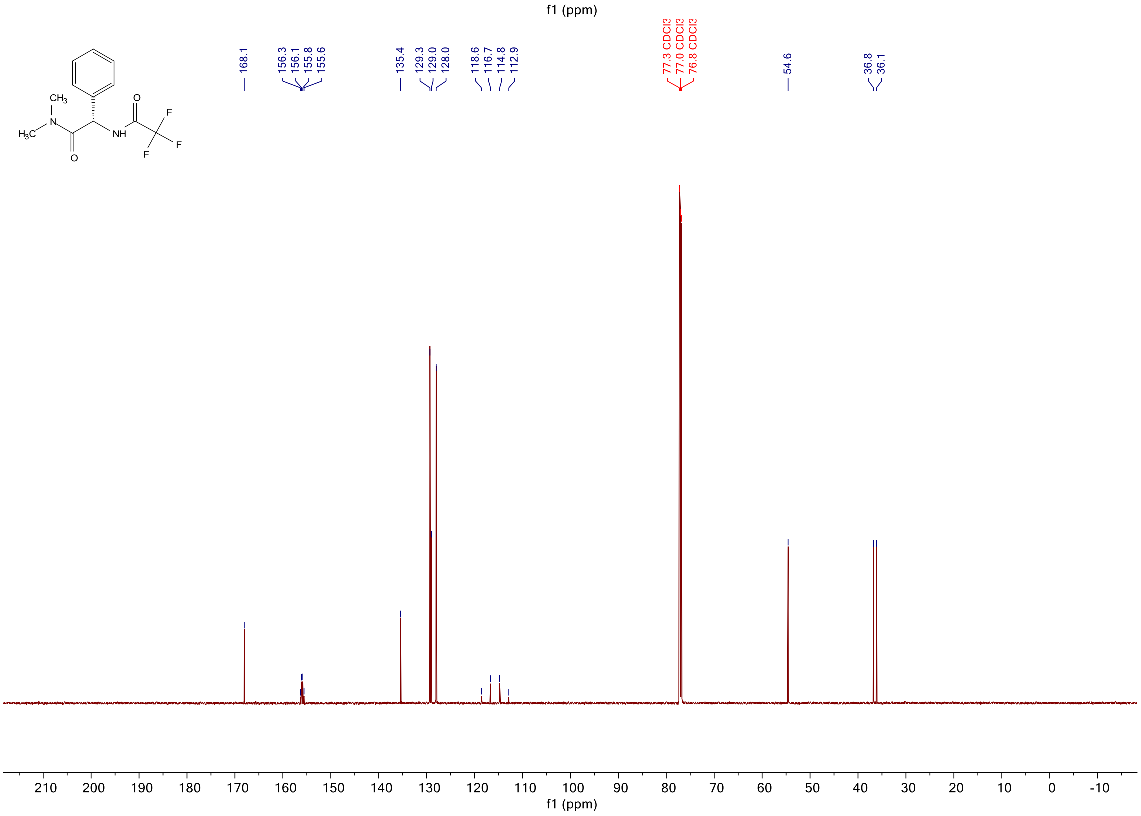
CH3
H3C
N
O
NH
O
F
F
F
168.1
156.3
156.1
155.8
155.6
135.4
129.3
129.0
128.0
118.6
116.7
114.8
112.9
77.3 CDCl3
77.0 CDCl3
76.8 CDCl3
54.6
36.8
36.1
f1 (ppm)

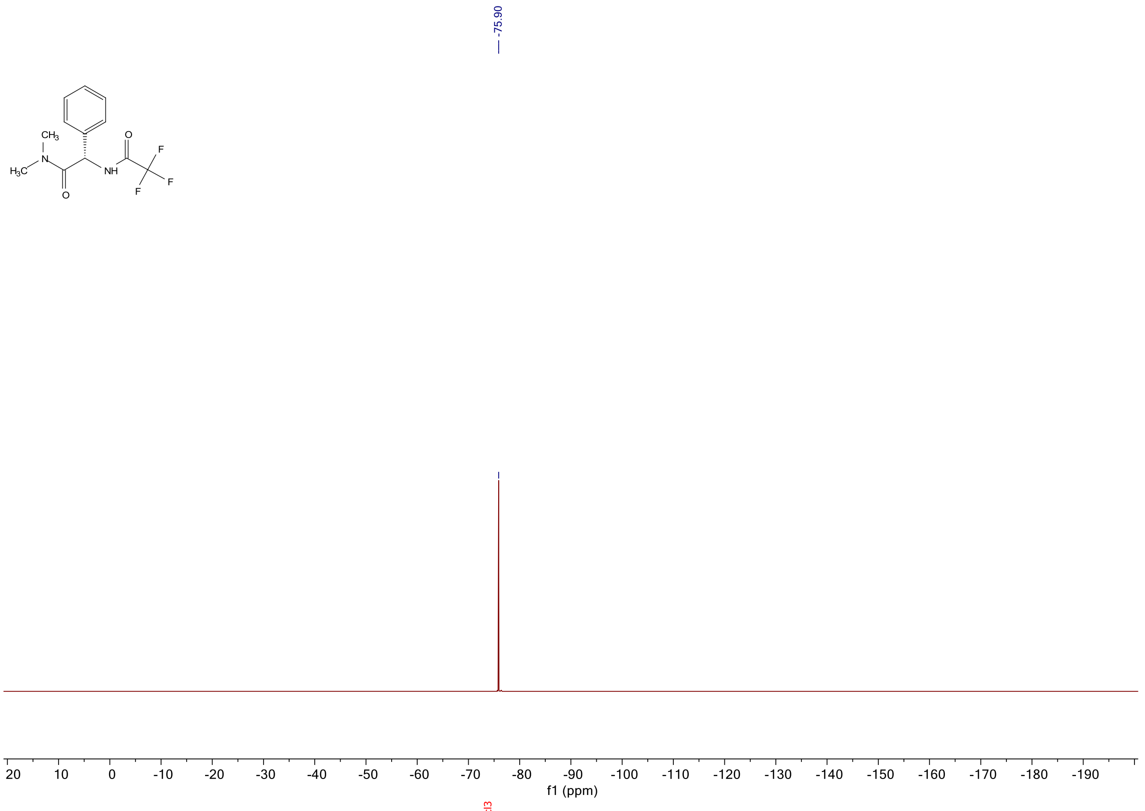

-75.90
CH3
H3C
N
O
NH
O
F
F
F
f1 (ppm)

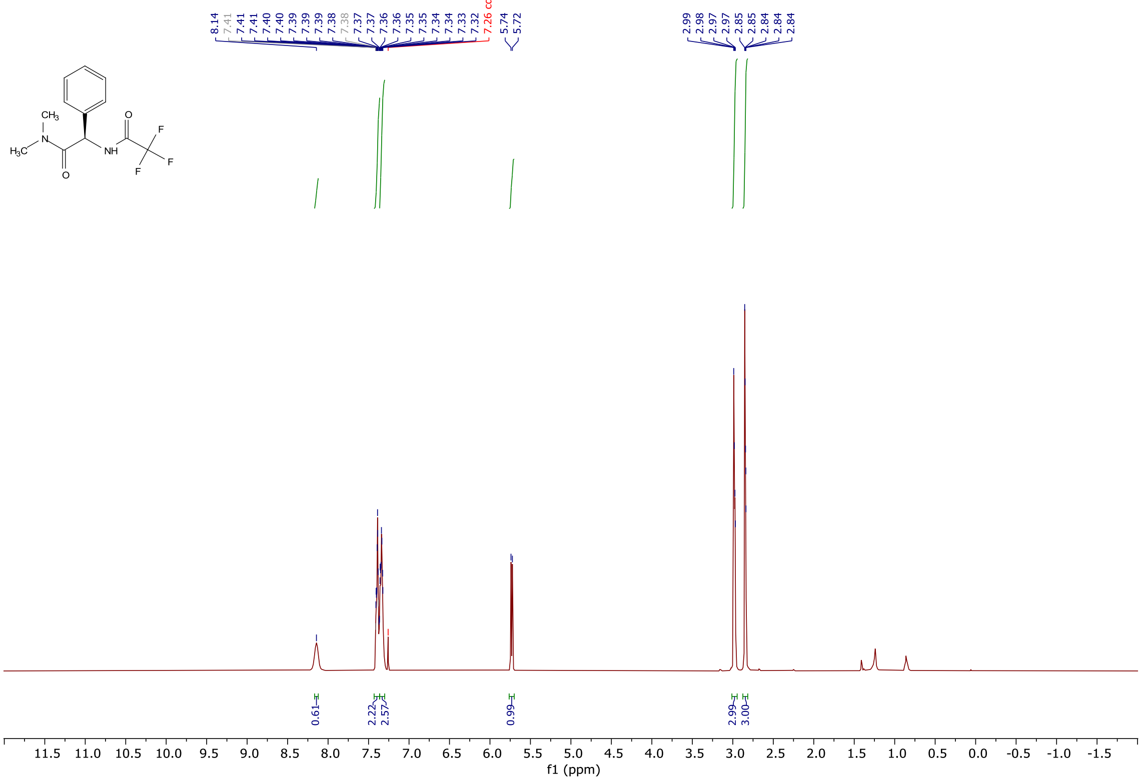

8.14
7.41
7.41
7.41
7.40
7.39
7.39
7.39
7.38
7.38
7.37
7.37
7.36
7.36
7.35
7.35
7.34
7.34
7.33
7.32
7.26 cdcl3
5.74
5.72
2.99
2.98
2.97
2.97
2.85
2.85
2.84
2.84
2.84
CH3
H3C
N
O
NH
O
F
F
F
0.61
2.22
2.57
0.99
2.99
3.00
f1 (ppm)

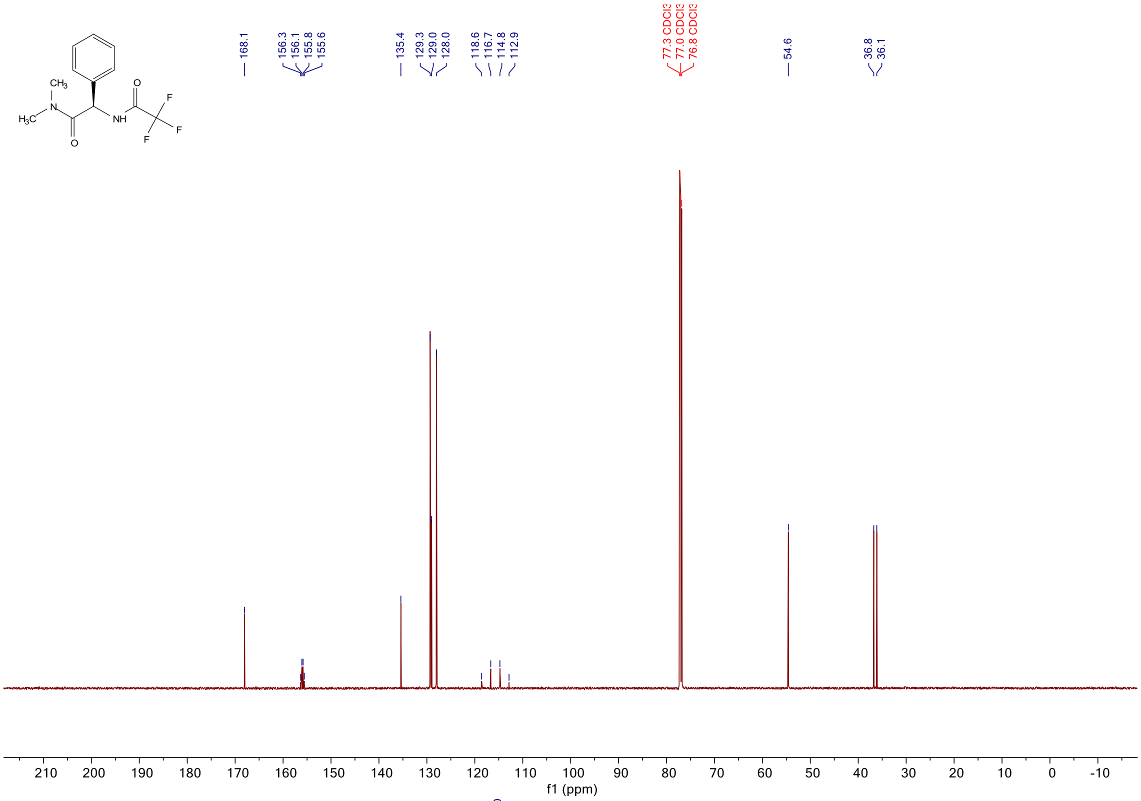

168.1
156.3
156.1
155.8
155.6
135.4
129.3
129.0
128.0
118.6
116.7
114.8
112.9
77.3 CDCl3
77.0 CDCl3
76.8 CDCl3
54.6
36.8
36.1
f1 (ppm)

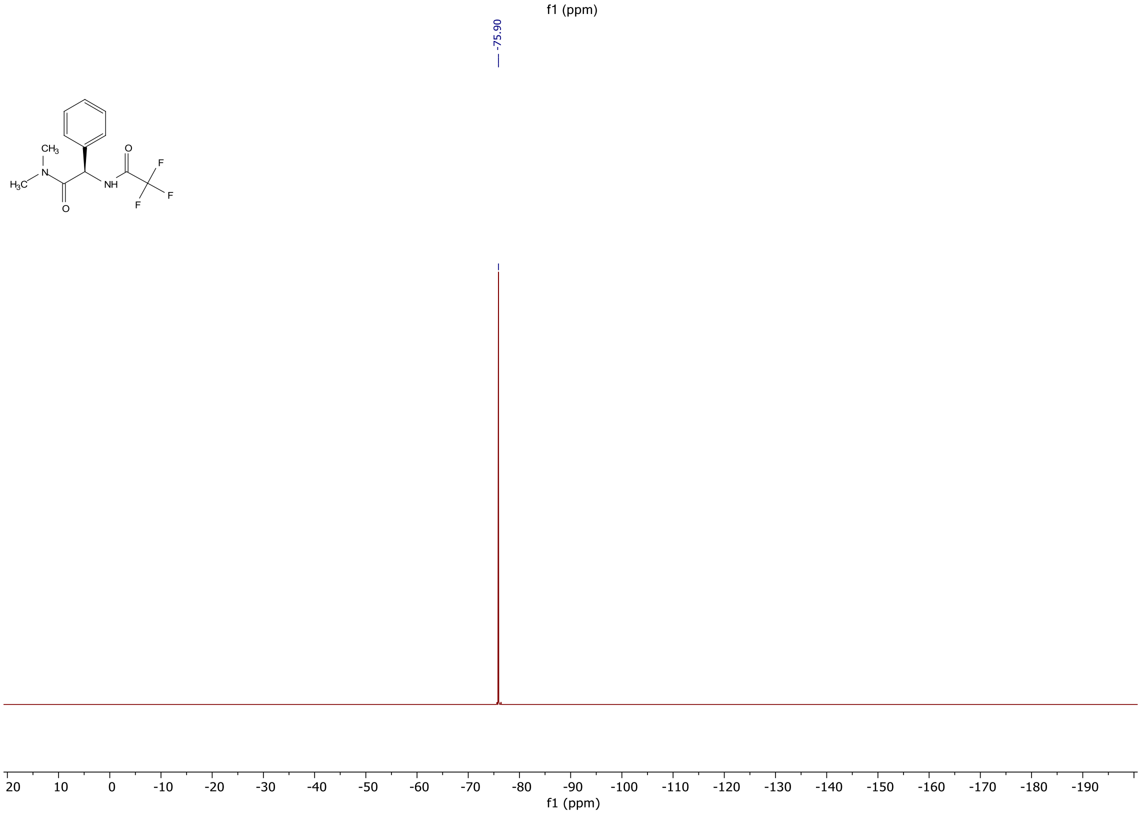

-75.90
f1 (ppm)

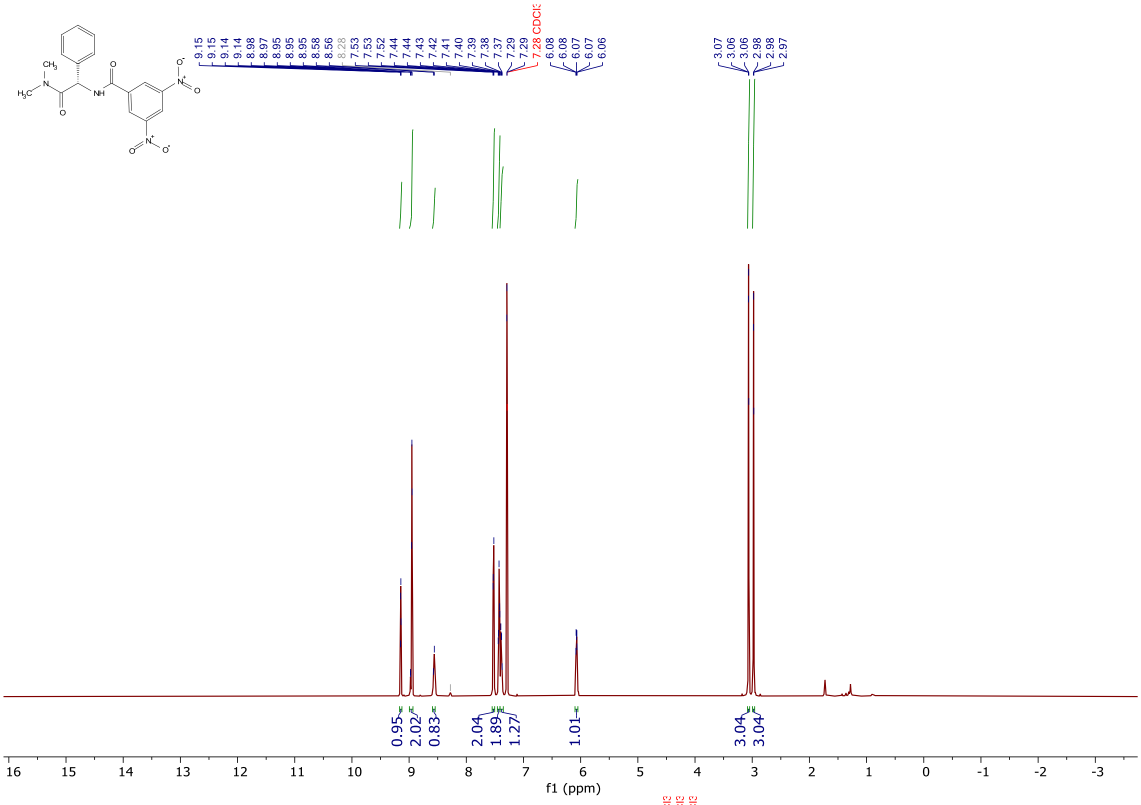
9.15 9.15 9.14 9.14 8.98 8.97 8.95 8.95 8.95 8.58 8.56 8.28 7.53 7.53 7.52 7.44 7.44 7.43 7.42 7.41 7.40 7.39 7.38 7.37 7.29 7.29
7.28 CDCl3
6.08 6.08 6.07 6.07 6.06
3.07 3.06 3.06 2.98 2.98 2.97
0.95 2.02 0.83 2.04 1.89 1.27 1.01 3.04 3.04
f1 (ppm)

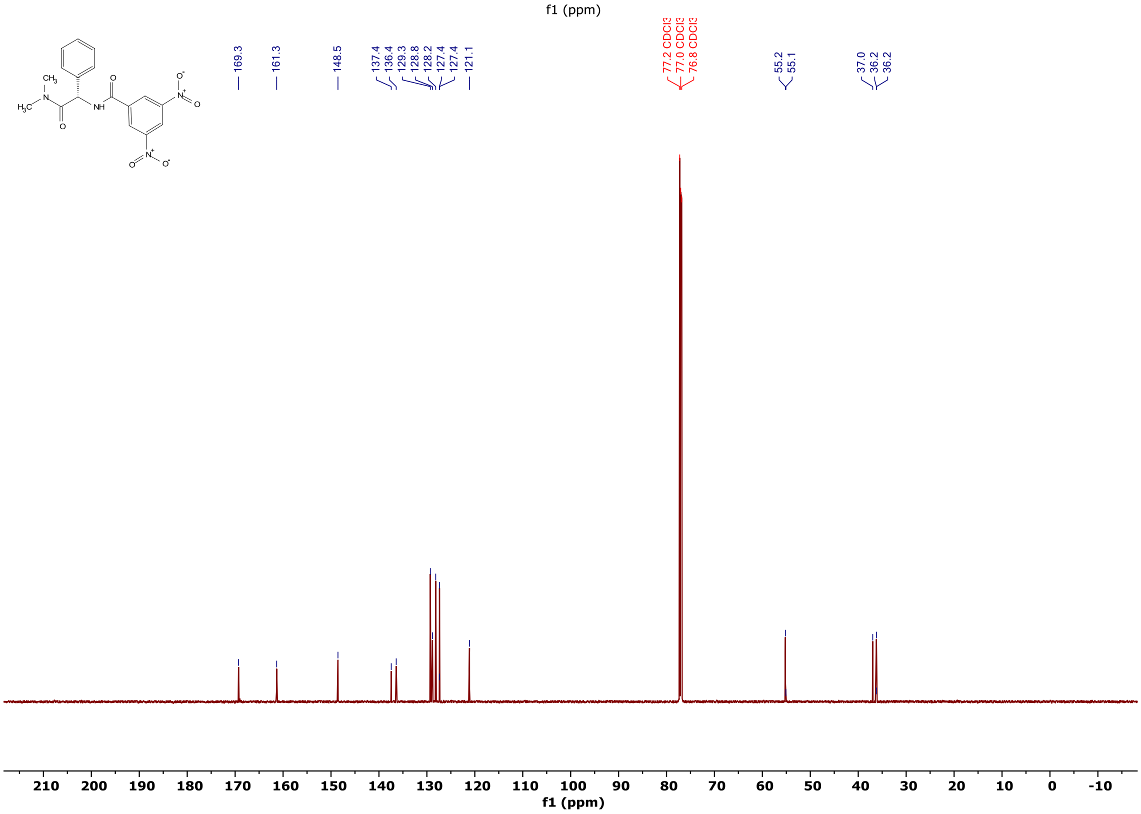
169.3
161.3
148.5
137.4 136.4 129.3 128.8 128.2 127.4 121.1
77.2 CDCl3 77.0 CDCl3 76.8 CDCl3
55.2 55.1
37.0 36.2 36.2
f1 (ppm)

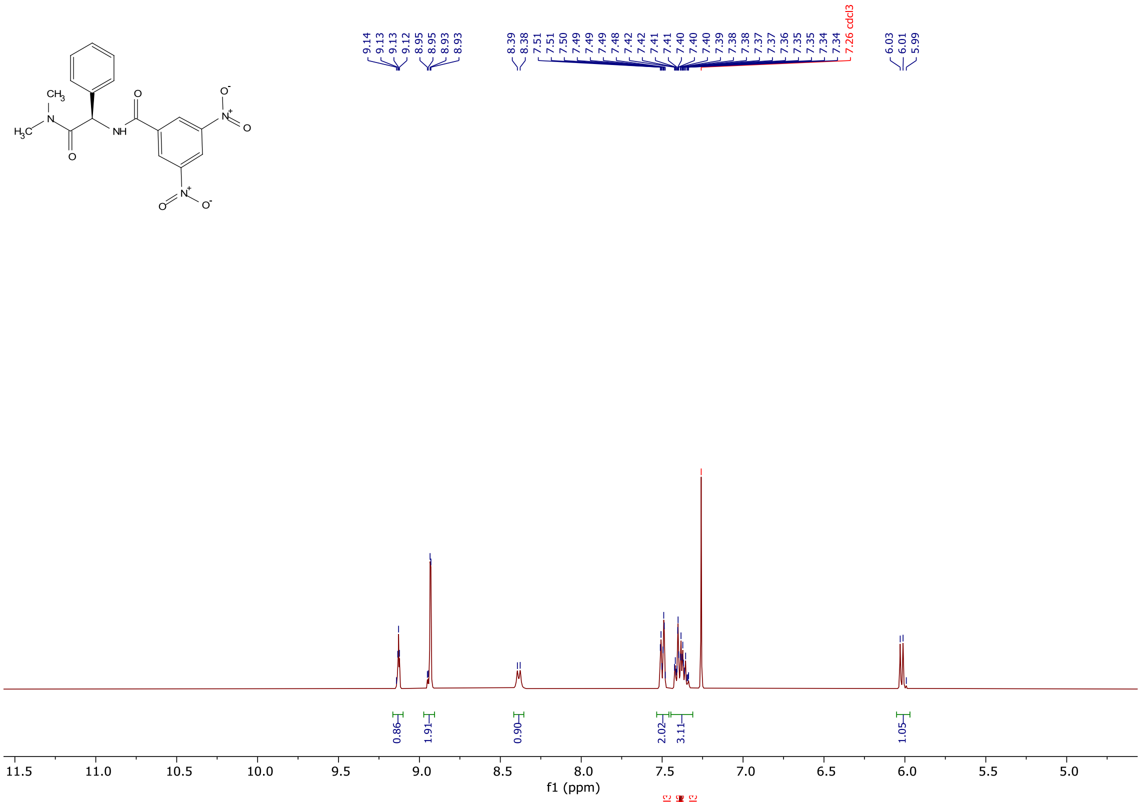

CH3
H3C
N
O
NH
O
N+
O-
O
N+
O
O-
9.14
9.13
9.13
9.12
8.95
8.95
8.93
8.93
8.39
8.38
7.51
7.51
7.50
7.49
7.49
7.49
7.48
7.42
7.42
7.41
7.41
7.40
7.40
7.40
7.39
7.38
7.38
7.37
7.37
7.36
7.35
7.35
7.34
7.34
7.26 cdcl3
6.03
6.01
5.99
0.86
1.91
0.90
2.02
3.11
1.05
11.5
11.0
10.5
10.0
9.5
9.0
8.5
8.0
7.5
7.0
6.5
6.0
5.5
5.0
f1 (ppm)

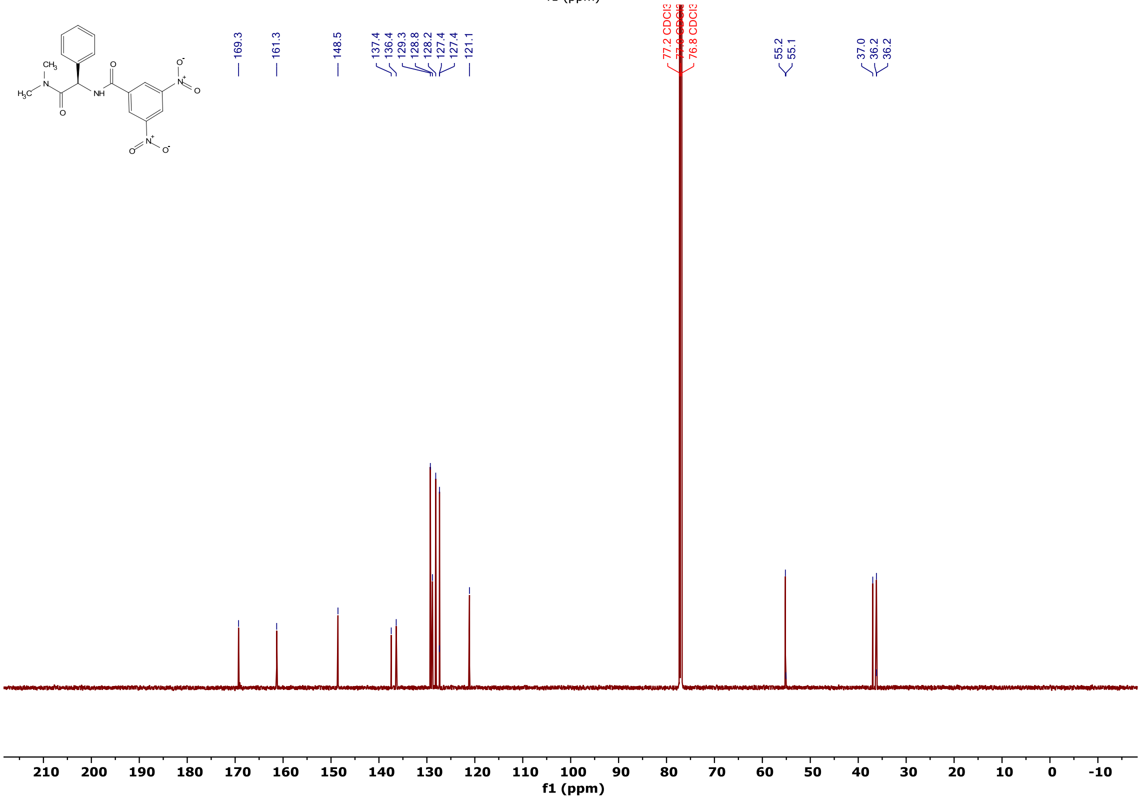

CH3
H3C
N
O
NH
O
N+
O-
O
N+
O
O-
169.3
161.3
148.5
137.4
136.4
129.3
128.8
128.2
127.4
127.4
121.1
77.2 CDCl3
76.8 CDCl3
55.2
55.1
37.0
36.2
36.2
210
200
190
180
170
160
150
140
130
120
110
100
90
80
70
60
50
40
30
20
10
0
-10
f1 (ppm)

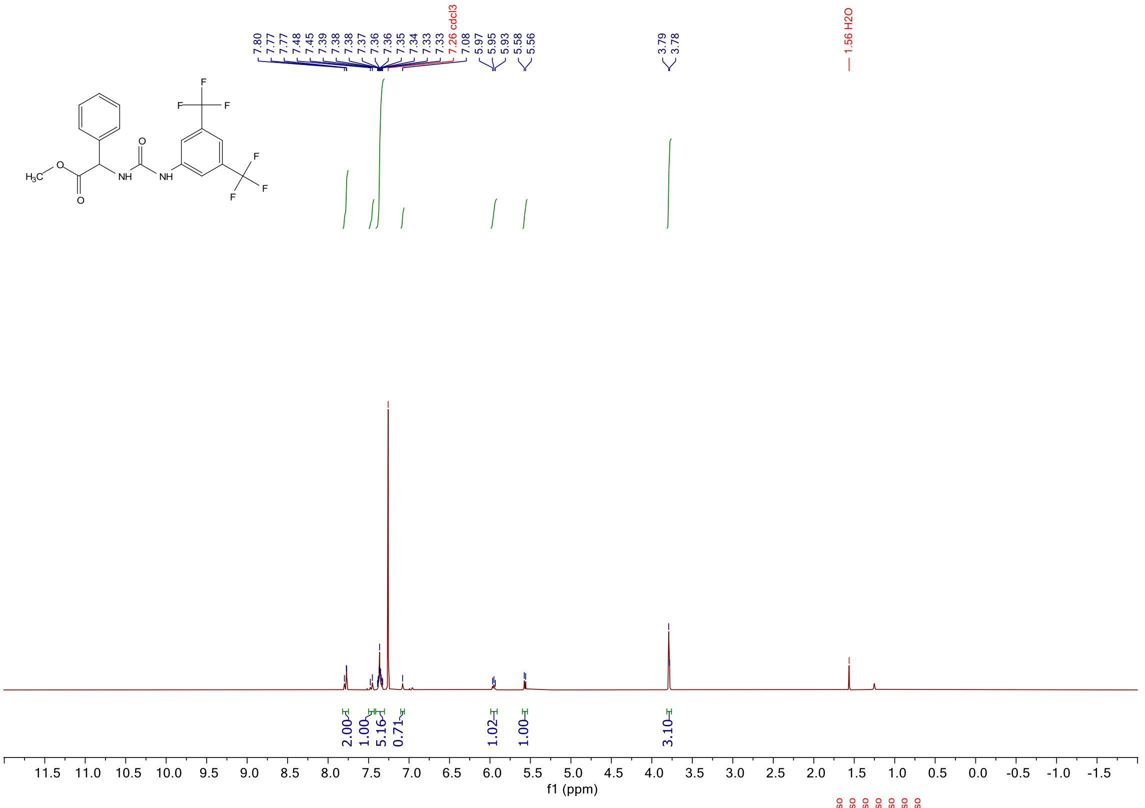

7.80
7.77
7.77
7.48
7.45
7.39
7.38
7.38
7.37
7.36
7.36
7.35
7.34
7.33
7.33
7.26 cdcl3
7.08
5.97
5.95
5.93
5.58
5.56
3.79
3.78
1.56 H2O
2.00
1.00
5.16
0.71
1.02
1.00
3.10
f1 (ppm)

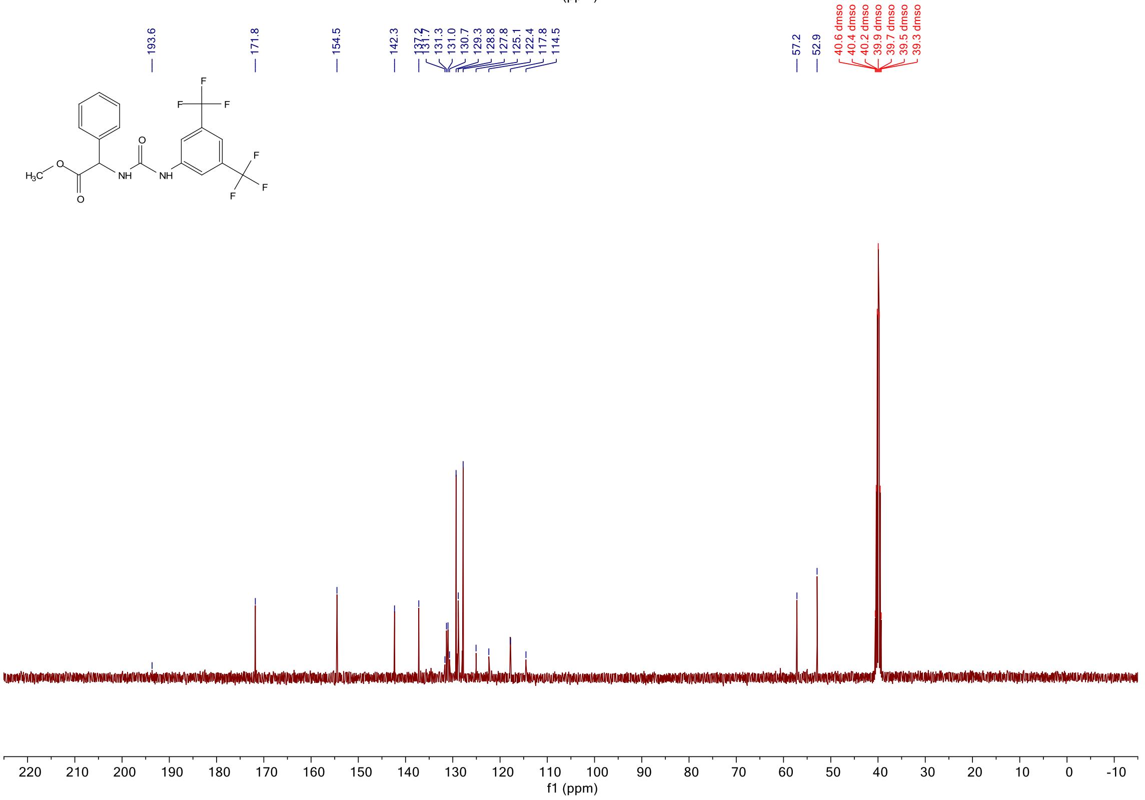

193.6
171.8
154.5
142.3
137.2
131.7
131.3
131.0
130.7
129.3
128.8
127.8
125.1
122.4
117.8
114.5
57.2
52.9
40.6 dmso
40.4 dmso
40.2 dmso
39.9 dmso
39.7 dmso
39.5 dmso
39.3 dmso
f1 (ppm)

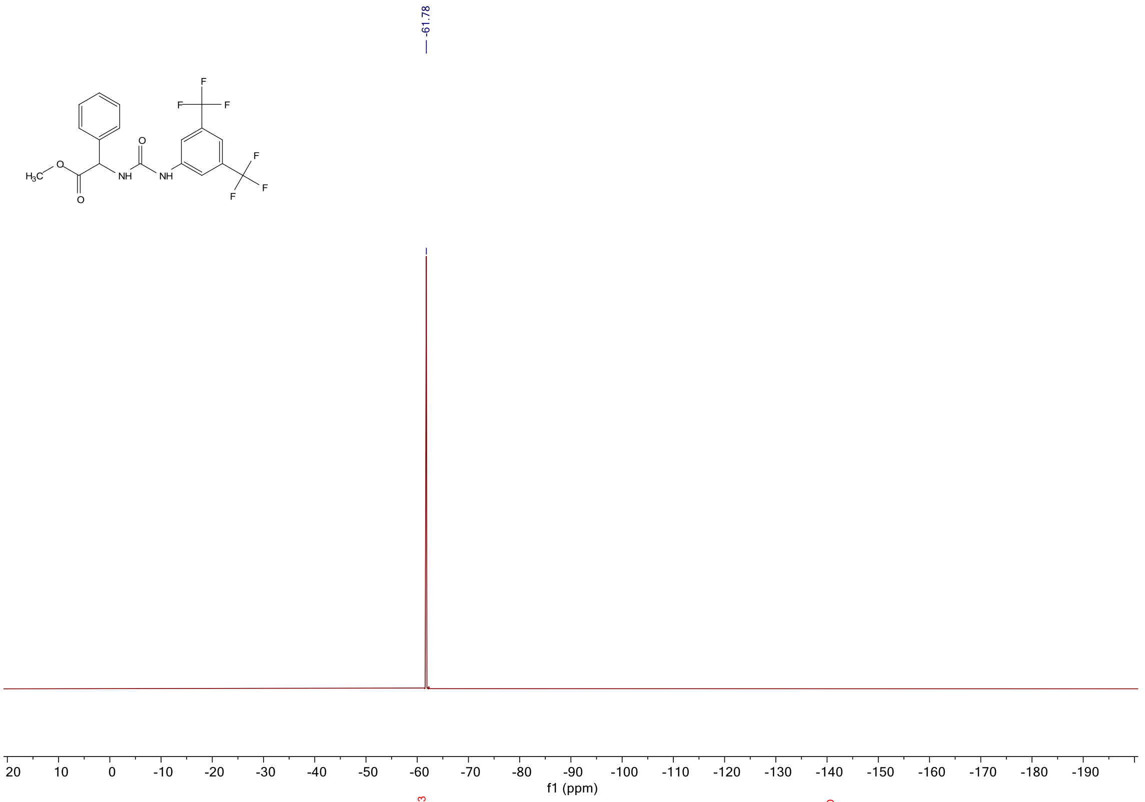
-61.78
H3C O NH NH O O F F F F F F
20 10 0 -10 -20 -30 -40 -50 -60 -70 -80 -90 -100 -110 -120 -130 -140 -150 -160 -170 -180 -190
f1 (ppm)

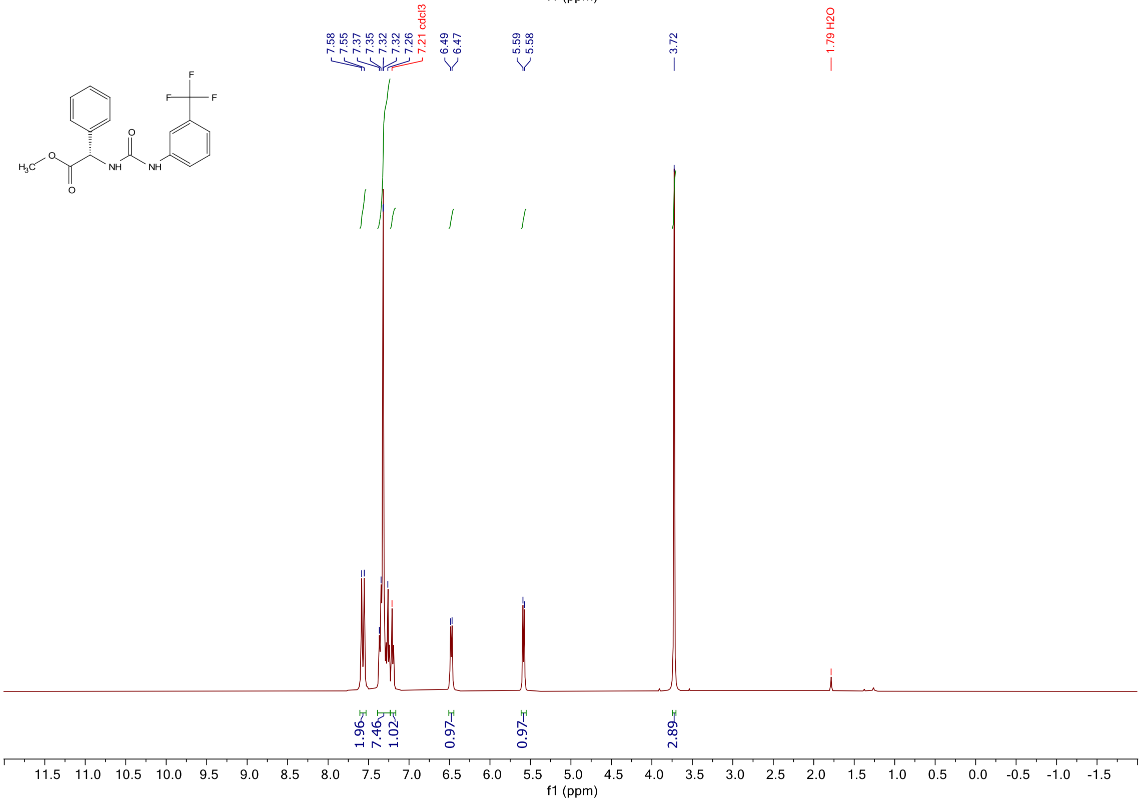
7.58
7.55
7.37
7.35
7.32
7.32
7.26
7.21 cdcl3
6.49
6.47
5.59
5.58
3.72
1.79 H2O
H3C O NH NH O O F F F
1.96
7.46
1.02
0.97
0.97
2.89
11.5 11.0 10.5 10.0 9.5 9.0 8.5 8.0 7.5 7.0 6.5 6.0 5.5 5.0 4.5 4.0 3.5 3.0 2.5 2.0 1.5 1.0 0.5 0.0 -0.5 -1.0 -1.5
f1 (ppm)

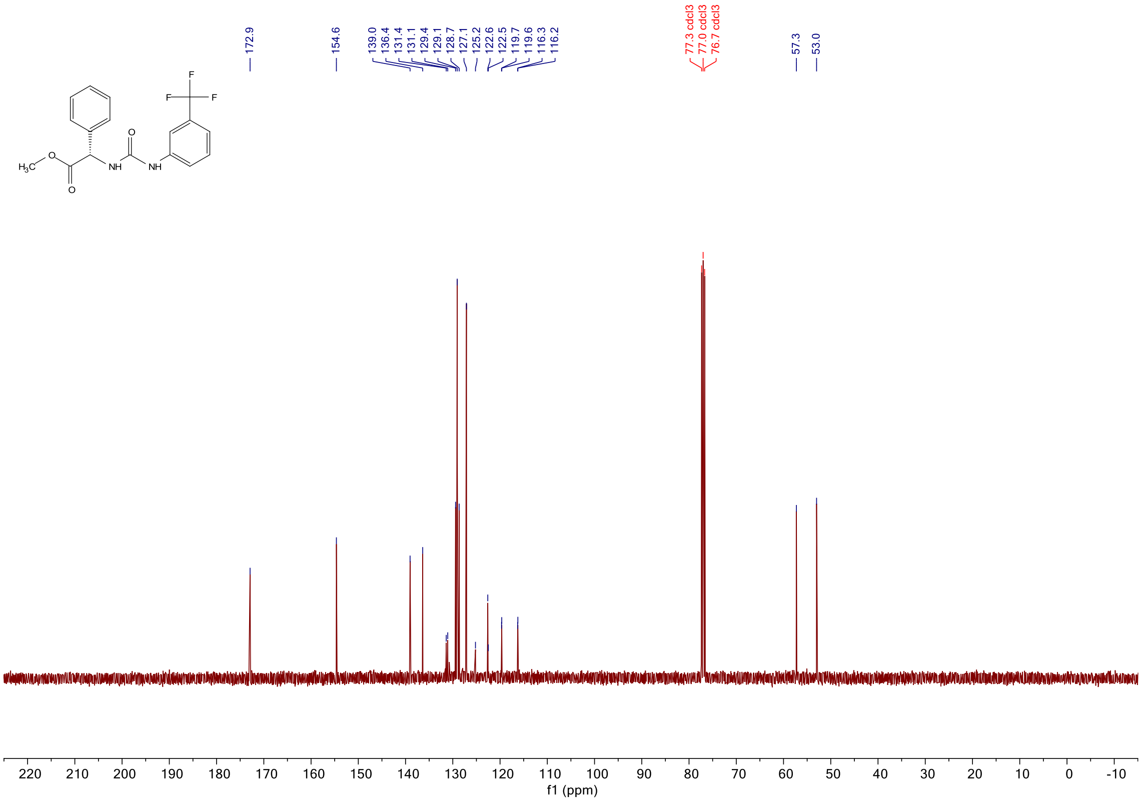

172.9
154.6
139.0
136.4
131.4
131.1
129.4
129.1
128.7
127.1
125.2
122.6
122.5
119.7
119.6
116.3
116.2
77.3 cdcl3
77.0 cdcl3
76.7 cdcl3
57.3
53.0
F
F
F
O
H3C
O
NH
NH
O
220 210 200 190 180 170 160 150 140 130 120 110 100 90 80 70 60 50 40 30 20 10 0 -10
f1 (ppm)

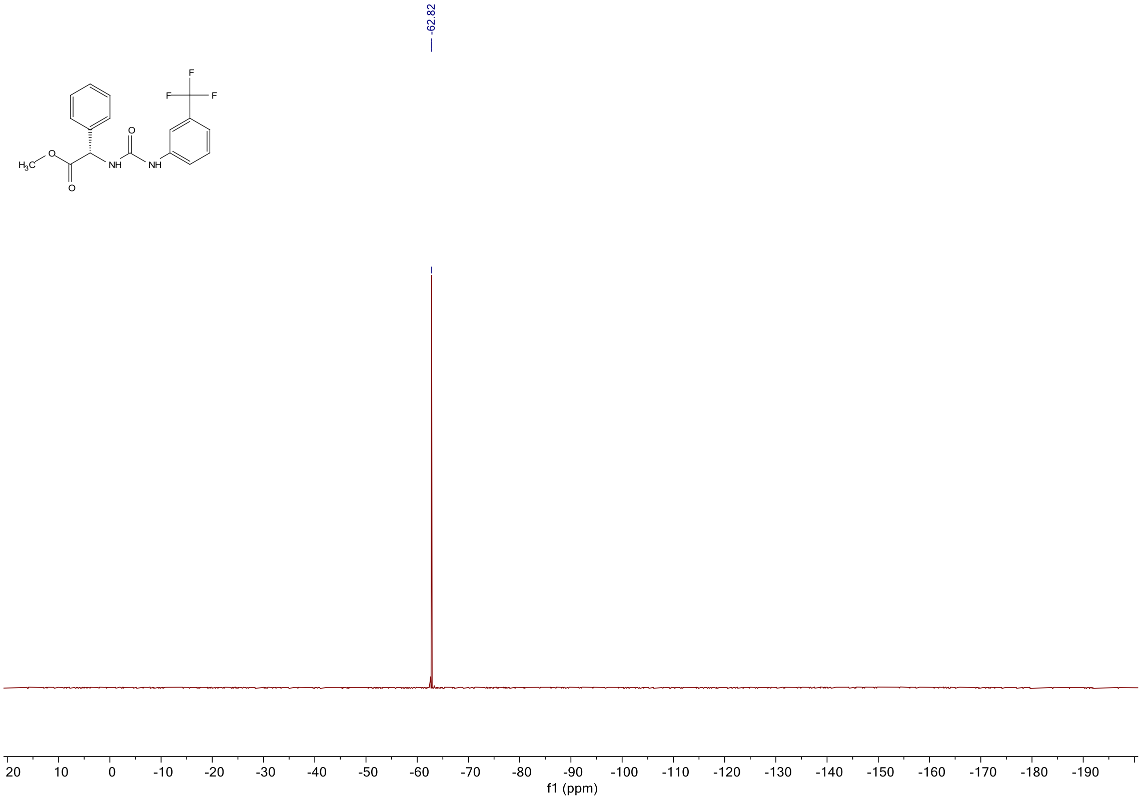

-62.82
F
F
F
O
H3C
O
NH
NH
O
20 10 0 -10 -20 -30 -40 -50 -60 -70 -80 -90 -100 -110 -120 -130 -140 -150 -160 -170 -180 -190
f1 (ppm)

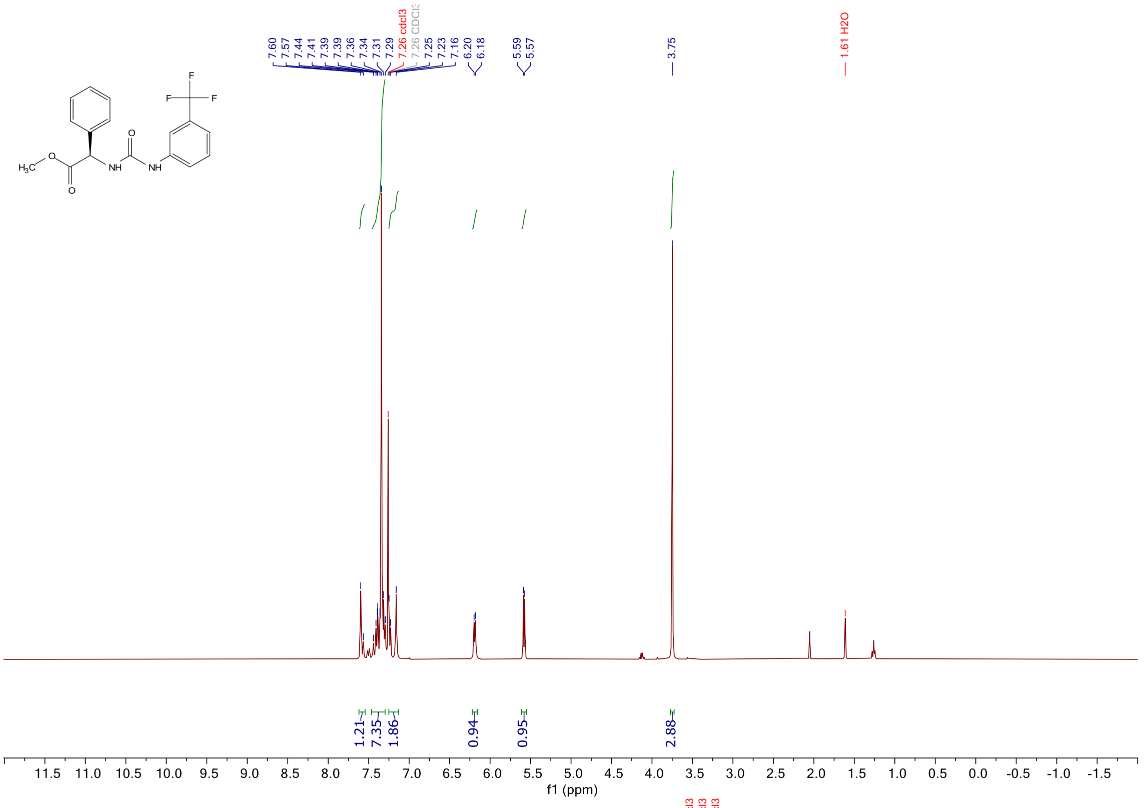
7.60
7.57
7.44
7.41
7.39
7.39
7.36
7.34
7.31
7.29
7.26 cdcl3
7.26 CDCl3
7.25
7.23
7.16
6.20
6.18
5.59
5.57
3.75
1.61 H2O
F
F
F
O
H3C
O
NH
NH
O
1.21
7.35
1.86
0.94
0.95
2.88
f1 (ppm)

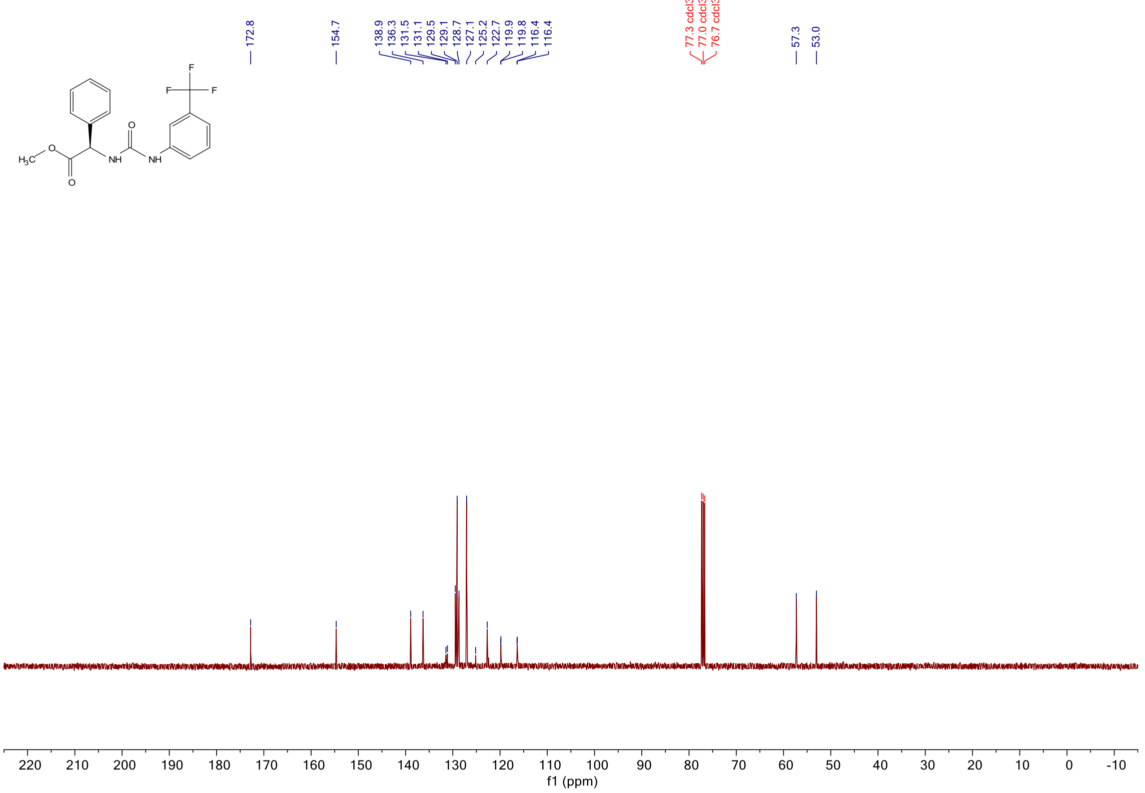
172.8
154.7
138.9
136.3
131.5
131.1
129.5
129.1
128.7
127.1
125.2
122.7
119.9
119.8
116.4
116.4
77.3 cdcl3
77.0 cdcl3
76.7 cdcl3
57.3
53.0
F
F
F
O
H3C
O
NH
NH
O
f1 (ppm)

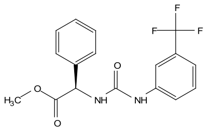

F
F
F
H3C
O
O
NH
O
NH

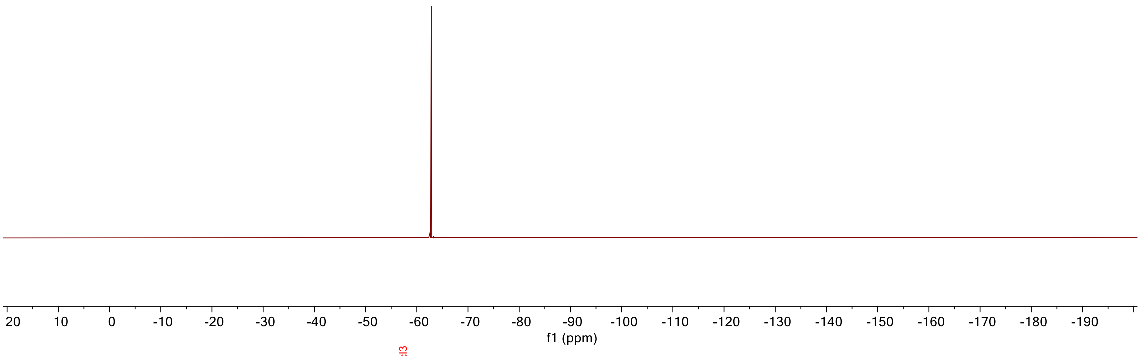

20 10 0 -10 -20 -30 -40 -50 -60 -70 -80 -90 -100 -110 -120 -130 -140 -150 -160 -170 -180 -190
f1 (ppm)

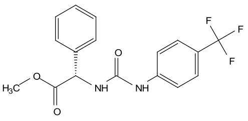

F
F
F
H3C
O
O
NH
O
NH

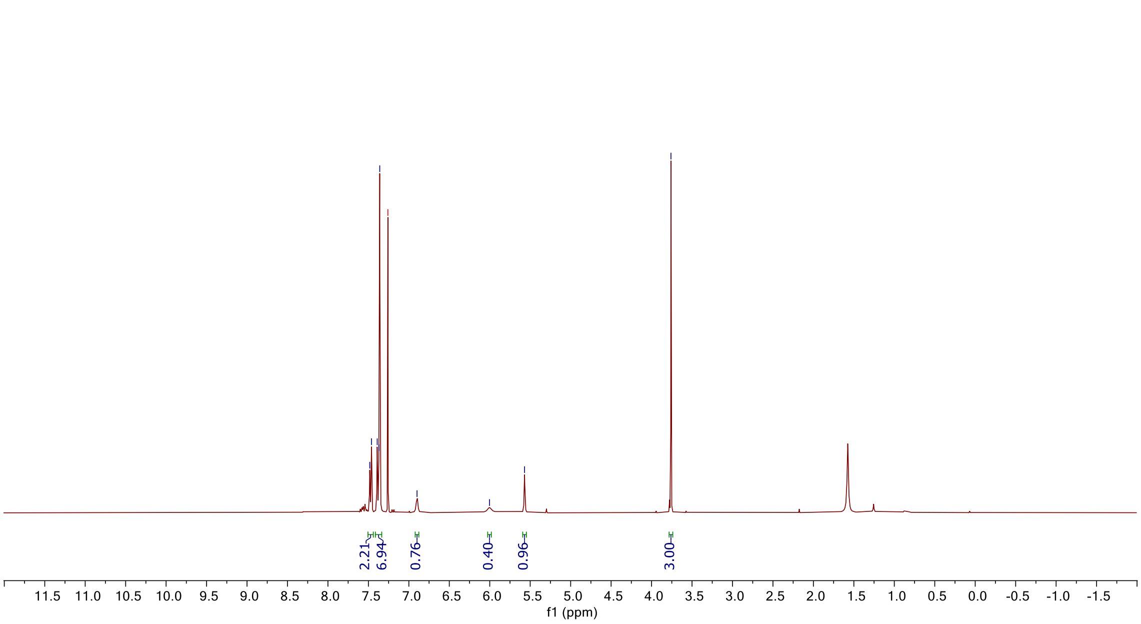

7.48
7.46
7.39
7.37
7.36
7.26 cdcl3
6.90
6.00
5.57
3.76
2.21
6.94
0.76
0.40
0.96
3.00
11.5 11.0 10.5 10.0 9.5 9.0 8.5 8.0 7.5 7.0 6.5 6.0 5.5 5.0 4.5 4.0 3.5 3.0 2.5 2.0 1.5 1.0 0.5 0.0 -0.5 -1.0 -1.5
f1 (ppm)

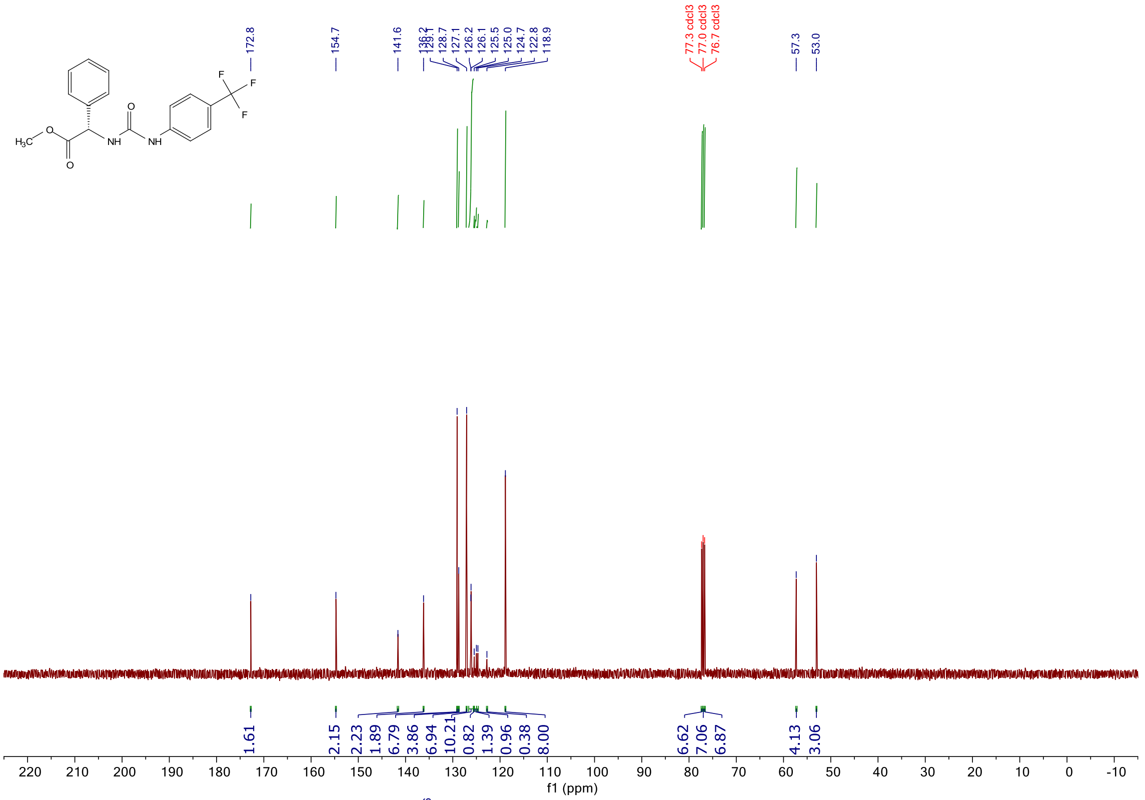

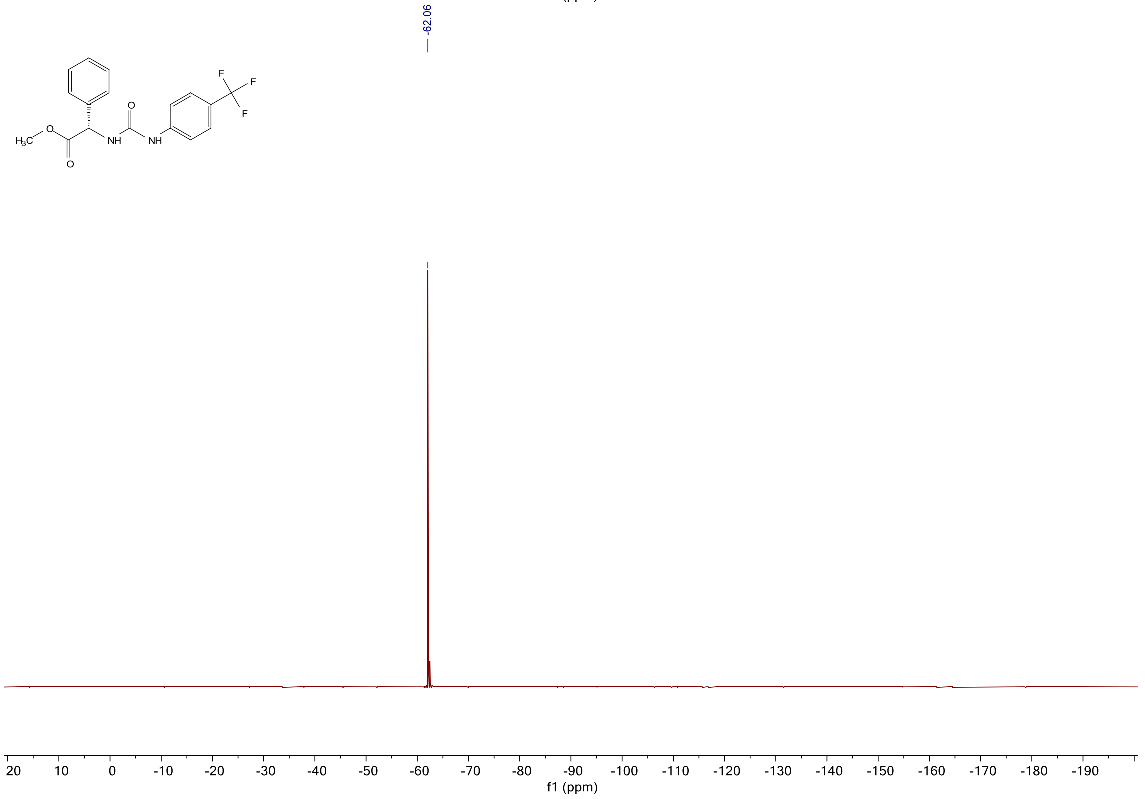

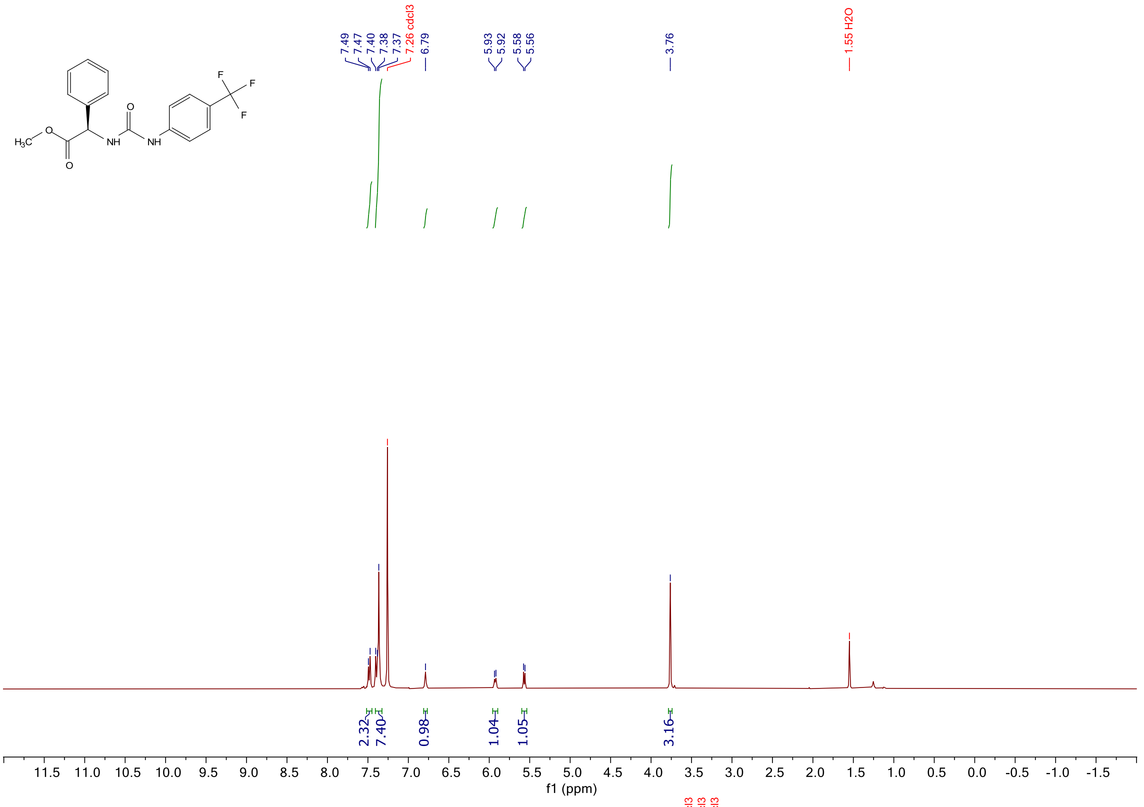
7.49
7.47
7.40
7.38
7.37
7.26 cdcl3
6.79
5.93
5.92
5.58
5.56
3.76
1.55 H2O
2.32
7.40
0.98
1.04
1.05
3.16
f1 (ppm)

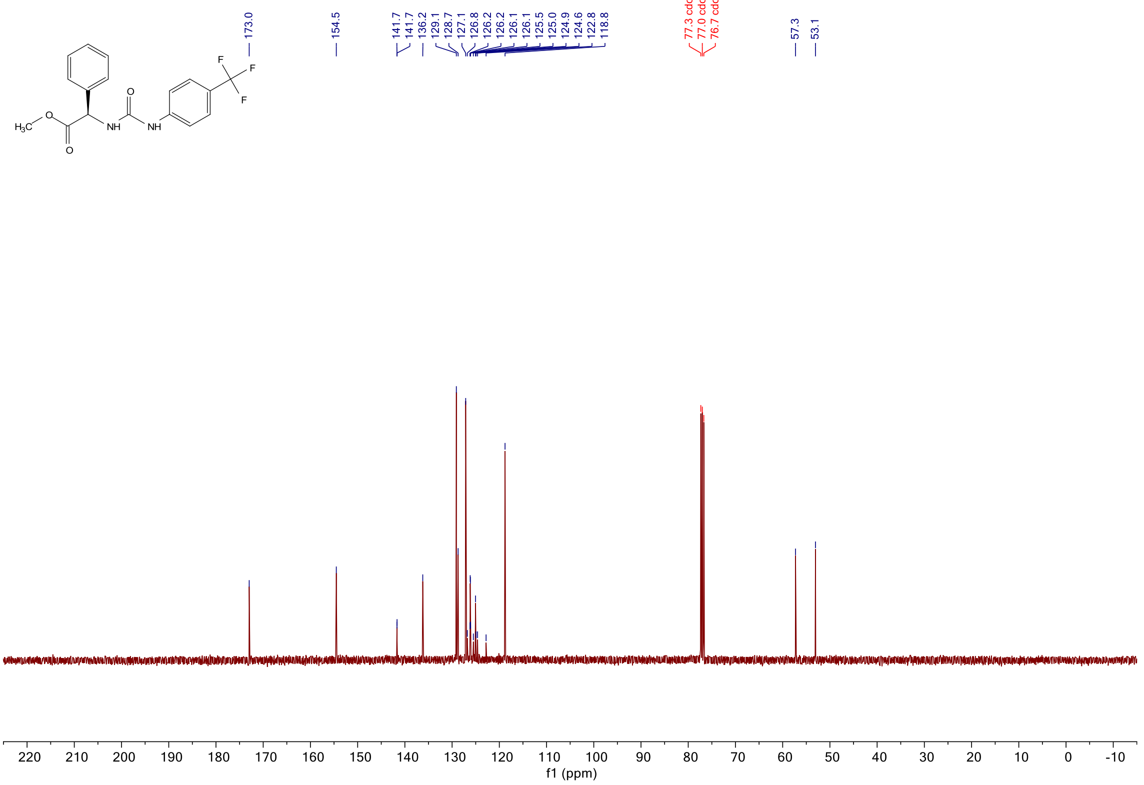
173.0
154.5
141.7
141.7
136.2
129.1
128.7
127.1
126.8
126.2
126.2
126.1
126.1
125.5
125.0
124.9
124.6
122.8
118.8
77.3 cdcl3
77.0 cdcl3
76.7 cdcl3
57.3
53.1
f1 (ppm)

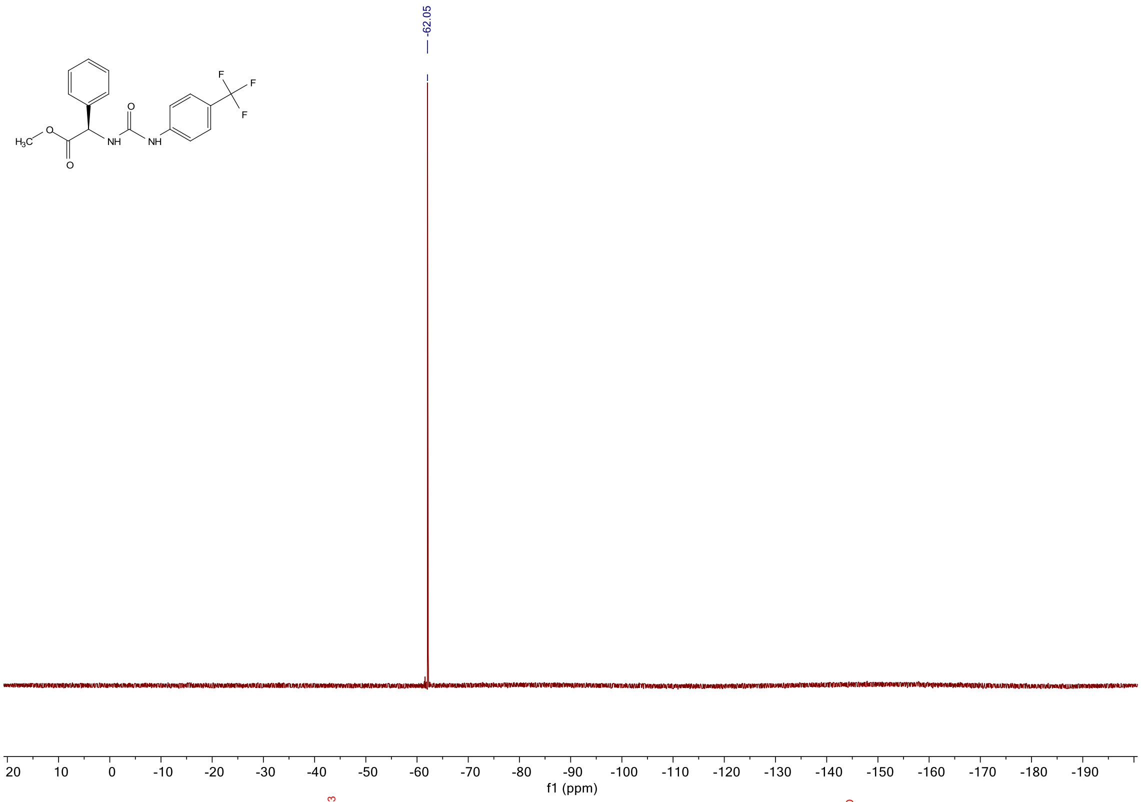

-62.05
f1 (ppm)

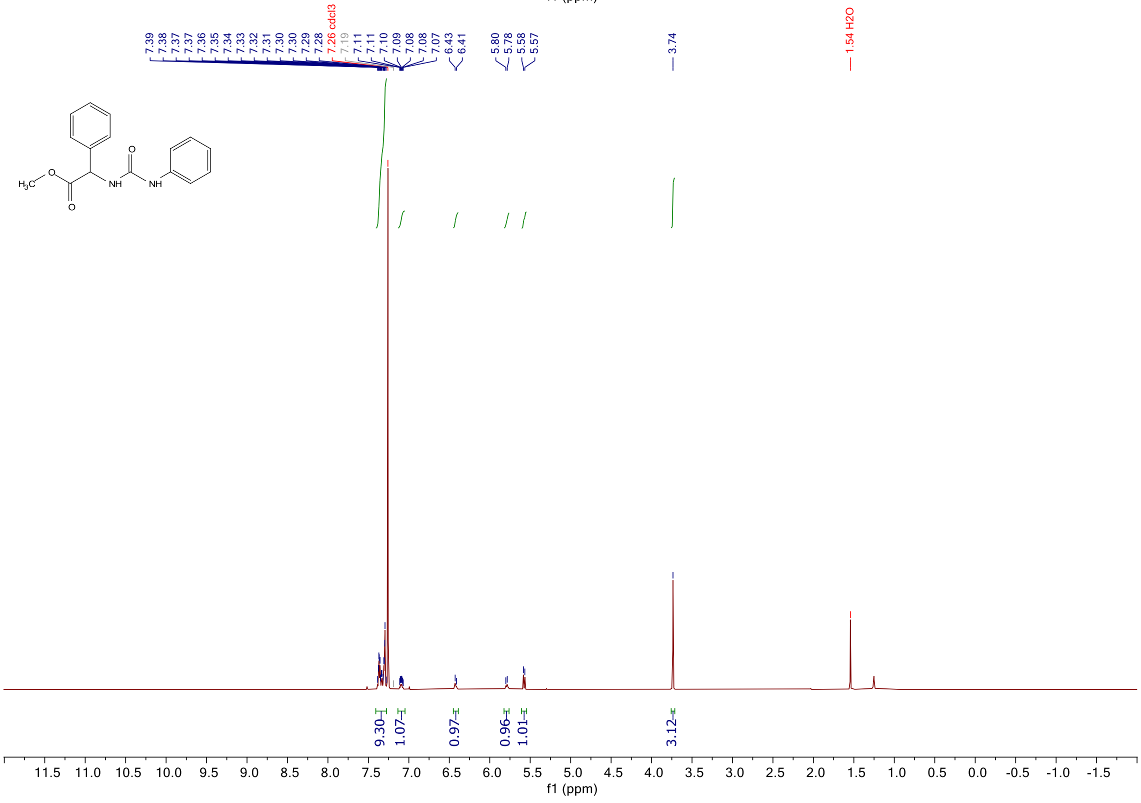

7.39 7.38 7.37 7.37 7.36 7.35 7.34 7.33 7.32 7.31 7.30 7.30 7.29 7.28 7.26 cdcl3 7.19 7.11 7.11 7.10 7.09 7.08 7.08 7.07 6.43 6.41 5.80 5.78 5.58 5.57 3.74 1.54 H2O
9.30 1.07 0.97 0.96 1.01 3.12
f1 (ppm)

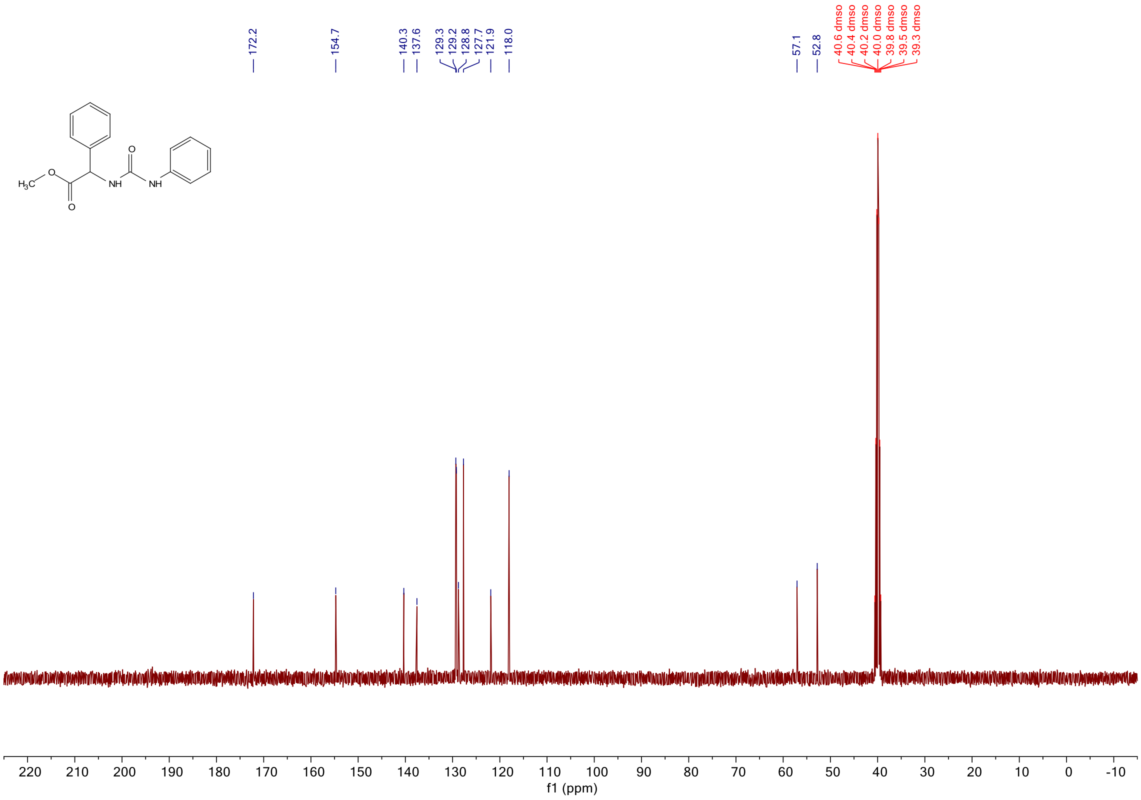
172.2
154.7
140.3
137.6
129.3
129.2
128.8
127.7
121.9
118.0
57.1
52.8
40.6 dmso
40.4 dmso
40.2 dmso
40.0 dmso
39.8 dmso
39.5 dmso
39.3 dmso
H3C O NH NH O O
f1 (ppm)

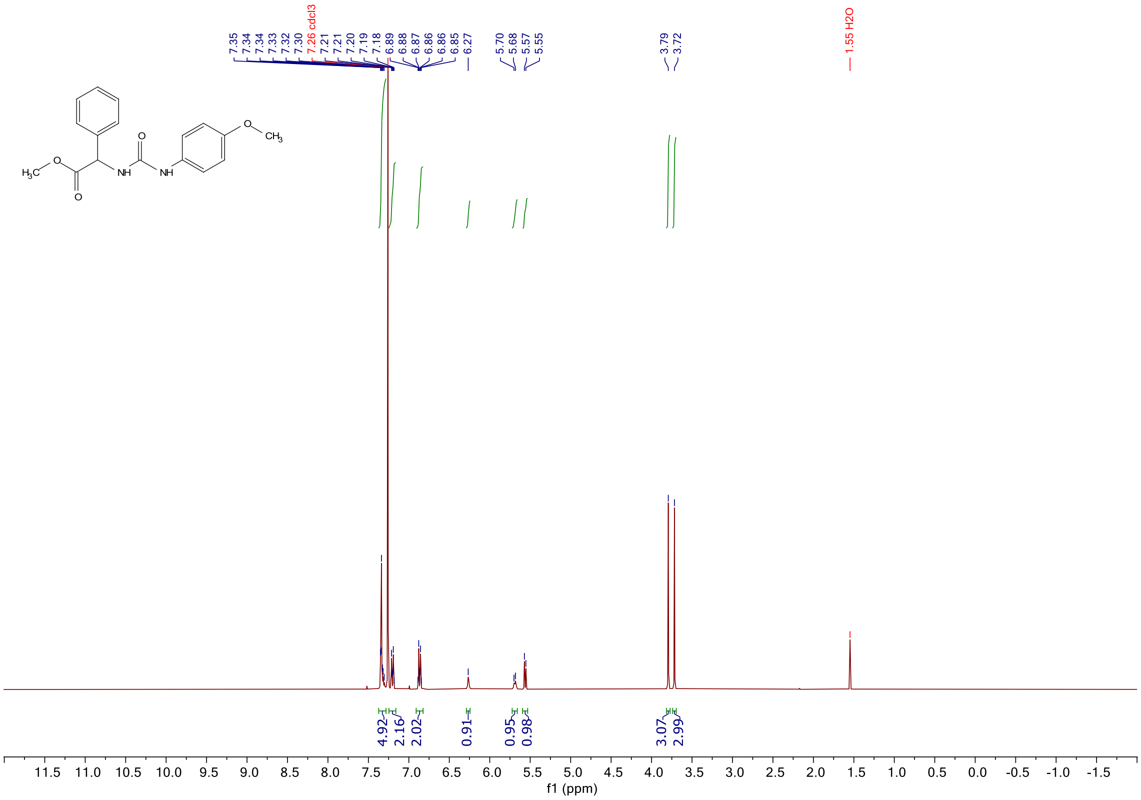
7.35
7.34
7.34
7.33
7.32
7.30
7.26 cdcl3
7.21
7.21
7.20
7.19
7.18
6.89
6.88
6.87
6.86
6.85
6.27
5.70
5.68
5.57
5.55
3.79
3.72
1.55 H2O
H3C O NH NH O O O CH3
4.92
2.16
2.02
0.91
0.95
0.98
3.07
2.99
f1 (ppm)

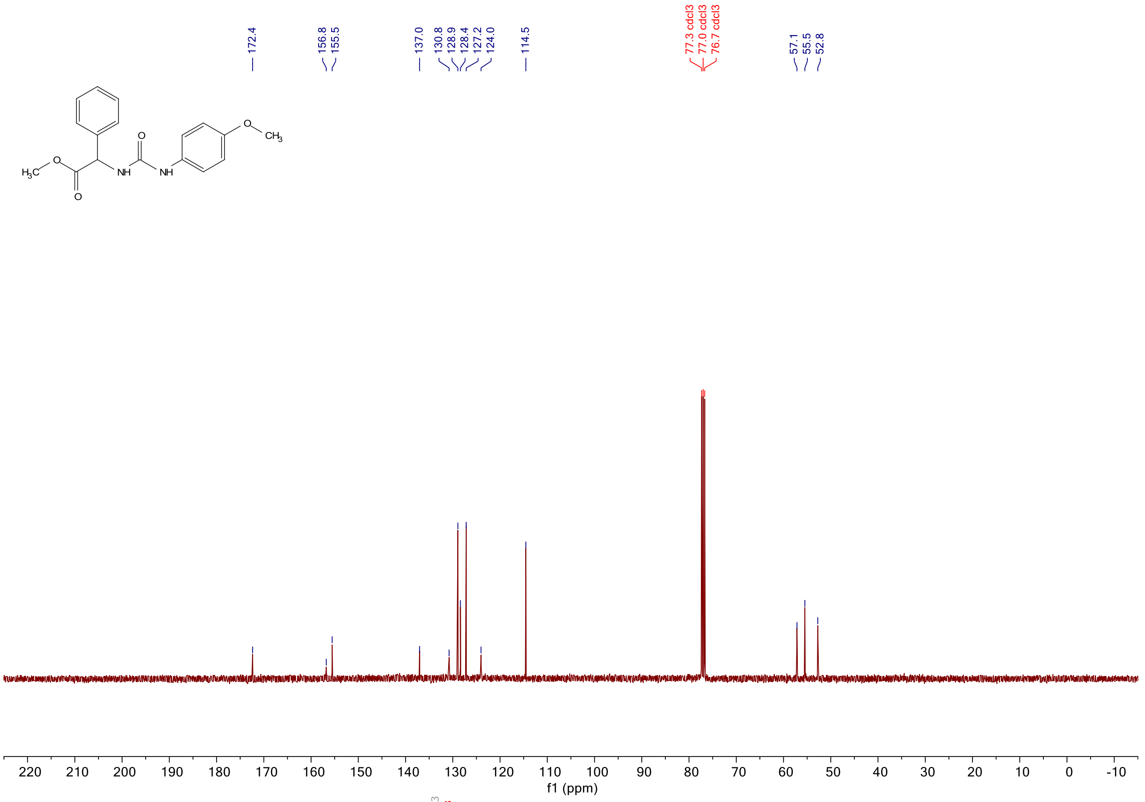
172.4
156.8
155.5
137.0
130.8
128.9
128.4
127.2
124.0
114.5
77.3 cdcl3
77.0 cdcl3
76.7 cdcl3
57.1
55.5
52.8
f1 (ppm)

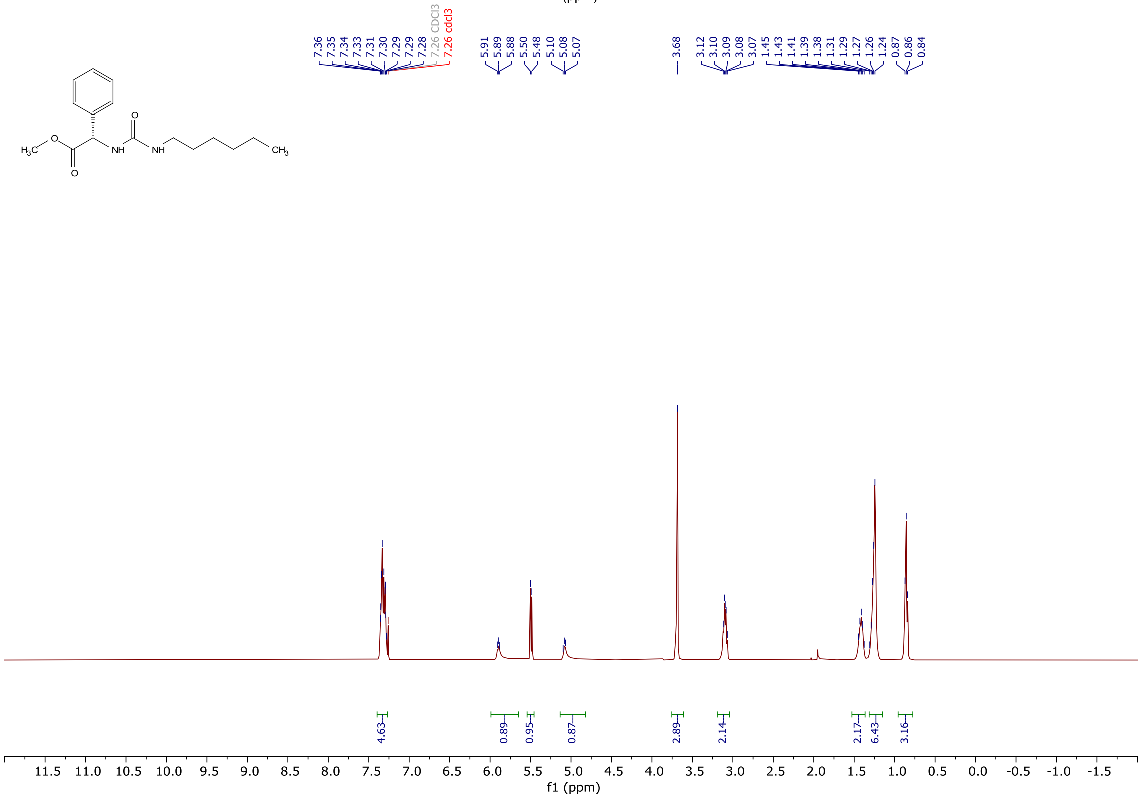
7.36
7.35
7.34
7.33
7.31
7.30
7.29
7.29
7.28
7.26 CDCl3
7.26 cdcl3
5.91
5.89
5.88
5.50
5.48
5.10
5.08
5.07
3.68
3.12
3.10
3.09
3.08
3.07
1.45
1.43
1.41
1.39
1.38
1.31
1.29
1.27
1.26
1.24
0.87
0.86
0.84
4.63
0.89
0.95
0.87
2.89
2.14
2.17
6.43
3.16
f1 (ppm)

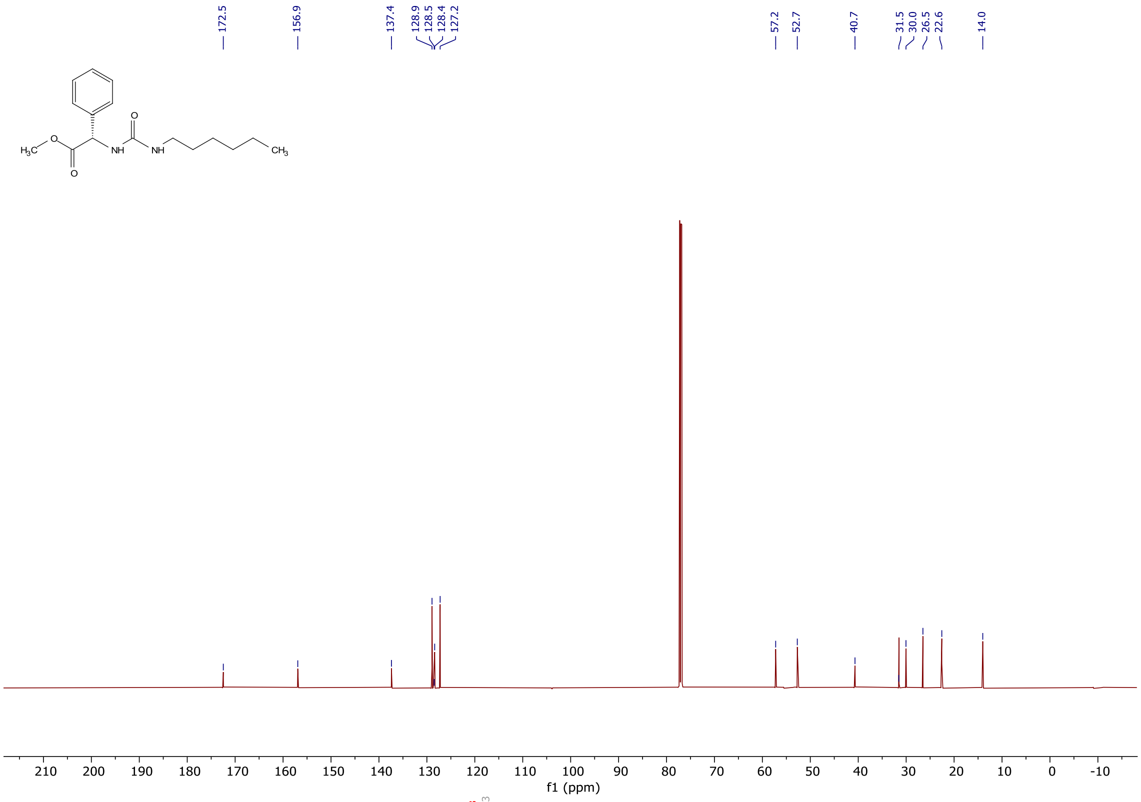
172.5
156.9
137.4
128.9
128.5
128.4
127.2
57.2
52.7
40.7
31.5
30.0
26.5
22.6
14.0
H_3C
O
NH
NH
CH_3
210 200 190 180 170 160 150 140 130 120 110 100 90 80 70 60 50 40 30 20 10 0 -10
f1 (ppm)

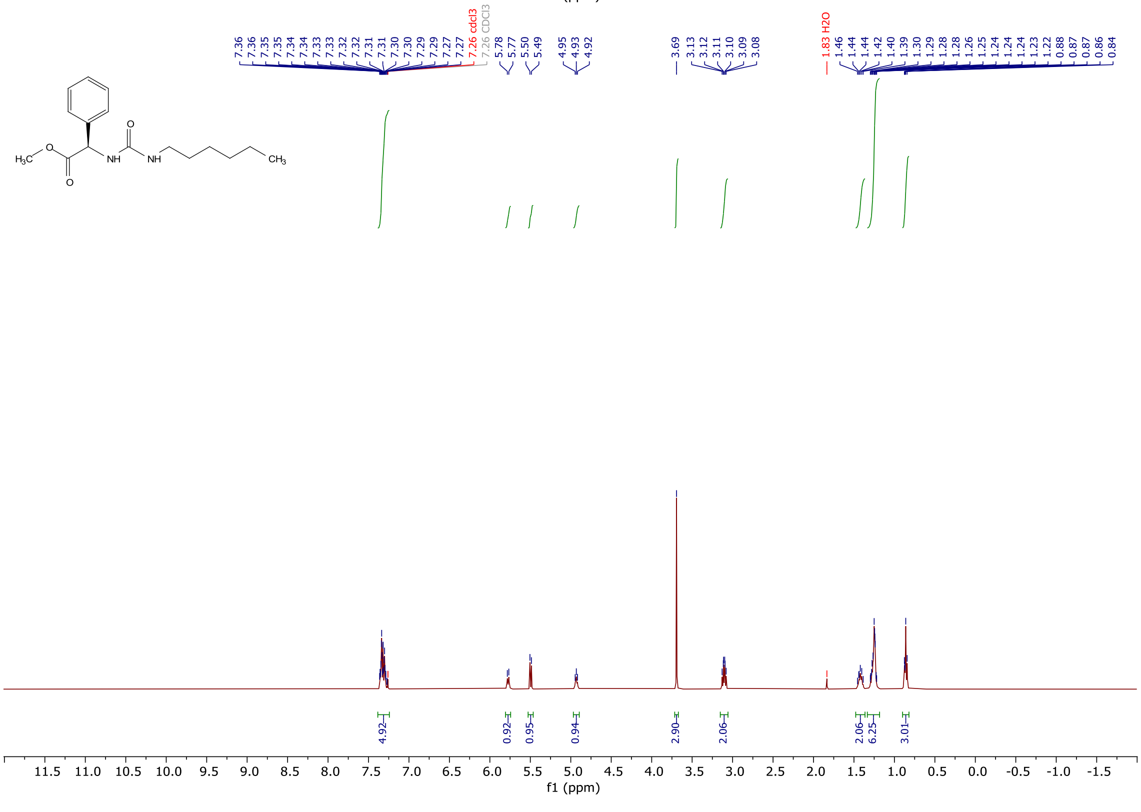
7.36 7.36 7.35 7.34 7.34 7.33 7.33 7.32 7.32 7.31 7.31 7.30 7.30 7.29 7.29 7.27 7.27
7.26 cdcl3
7.26 CDCl3
5.78 5.77 5.50 5.49
4.95 4.93 4.92
3.69
3.13 3.12 3.11 3.10 3.09 3.08
1.83 H2O
1.46 1.44 1.44 1.42 1.40 1.39 1.30 1.29 1.28 1.28 1.26 1.25 1.24 1.24 1.24 1.23 1.22 0.88 0.87 0.87 0.86 0.84
H_3C
O
NH
NH
CH_3
4.92
0.92
0.95
0.94
2.90
2.06
2.06
6.25
3.01
11.5 11.0 10.5 10.0 9.5 9.0 8.5 8.0 7.5 7.0 6.5 6.0 5.5 5.0 4.5 4.0 3.5 3.0 2.5 2.0 1.5 1.0 0.5 0.0 -0.5 -1.0 -1.5
f1 (ppm)

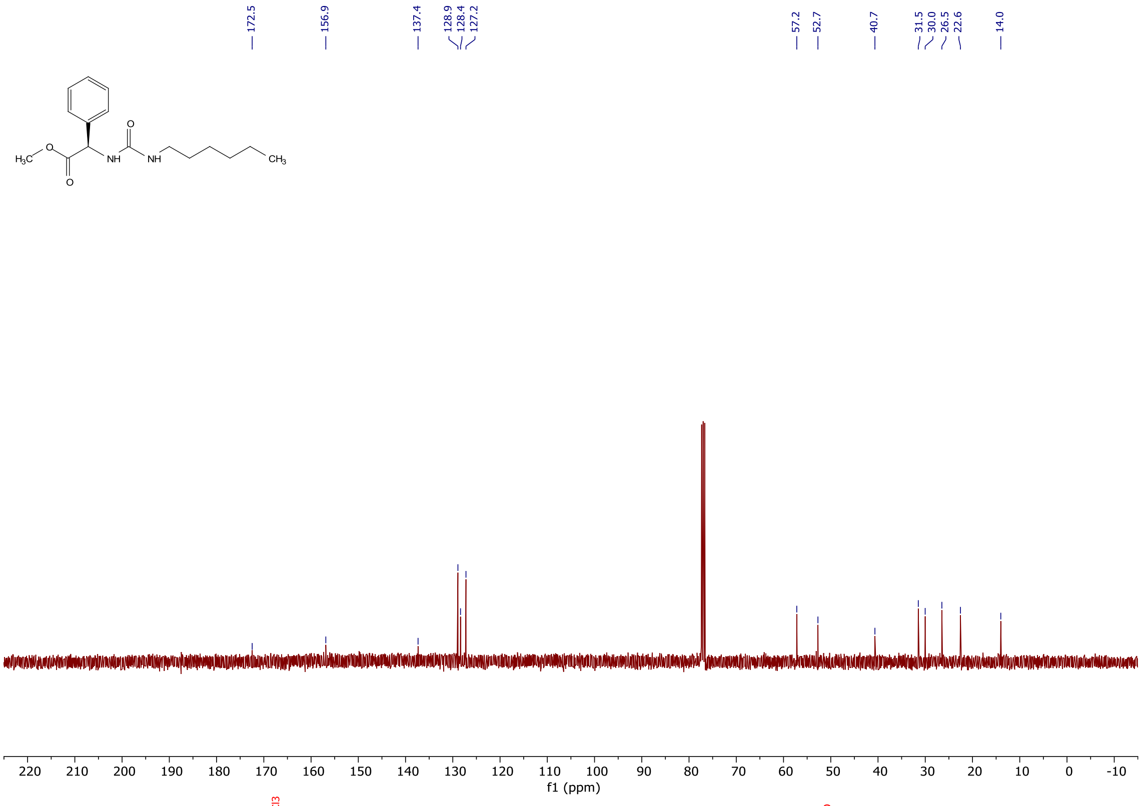
172.5
156.9
137.4
128.9
128.4
127.2
57.2
52.7
40.7
31.5
30.0
26.5
22.6
14.0
O
H_3C
O
NH
NH
CH_3
O
f1 (ppm)

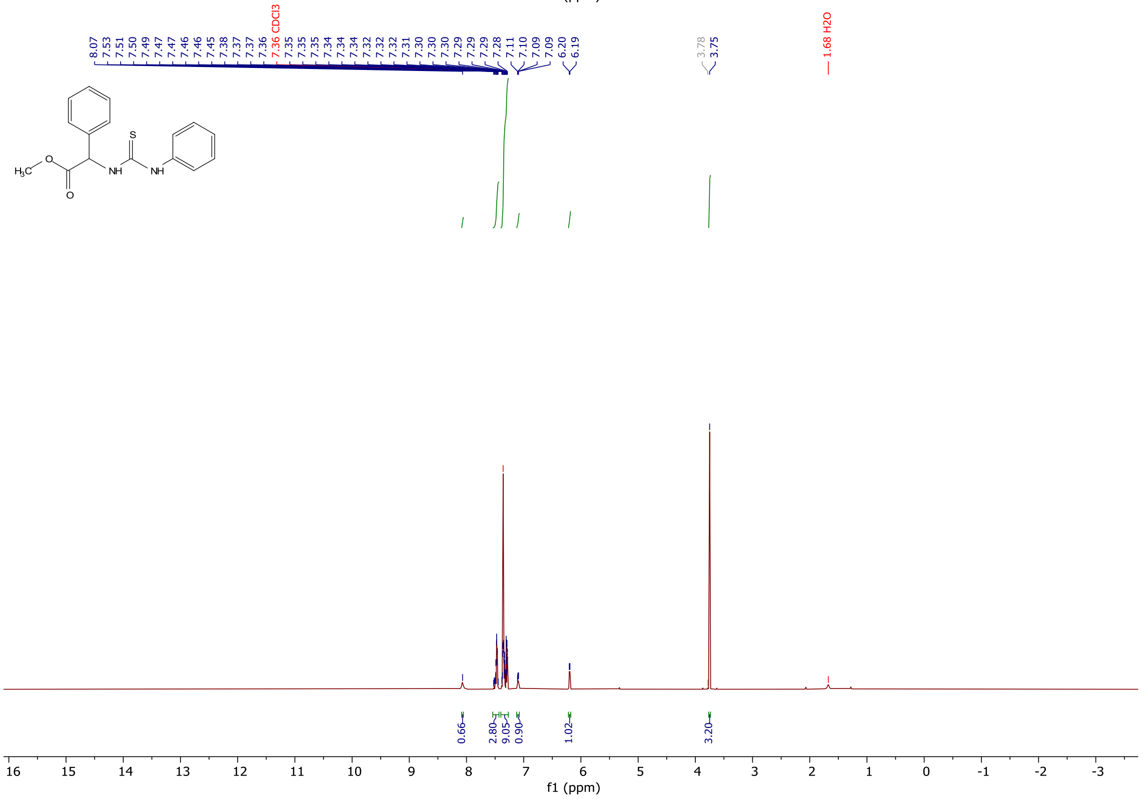
8.07
7.53
7.51
7.50
7.49
7.47
7.47
7.46
7.46
7.45
7.38
7.37
7.37
7.36
7.36 CDCl3
7.35
7.35
7.35
7.34
7.34
7.34
7.32
7.32
7.32
7.31
7.30
7.30
7.30
7.29
7.29
7.29
7.28
7.11
7.10
7.09
7.09
6.20
6.19
3.78
3.75
1.68 H2O
S
H_3C
O
NH
NH
O
0.66
2.80
9.05
0.90
1.02
3.20
f1 (ppm)

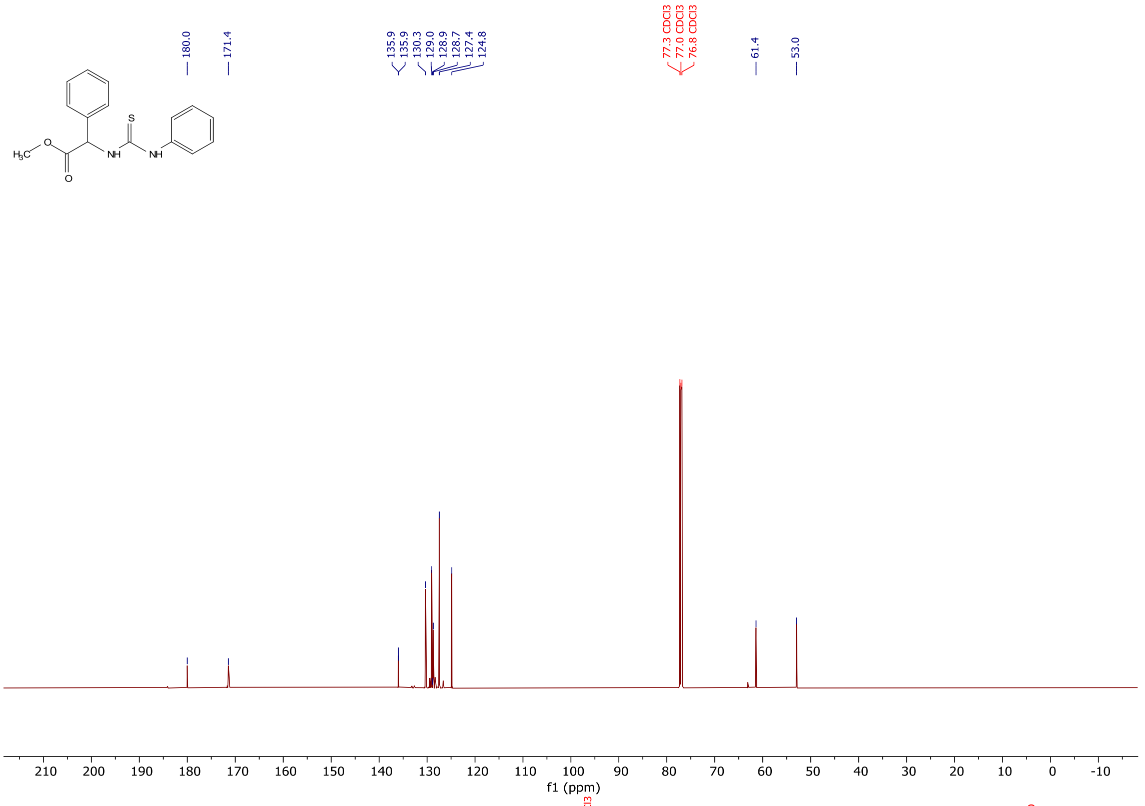
180.0
171.4
135.9
135.9
130.3
129.0
128.9
128.7
127.4
124.8
77.3 CDCl3
77.0 CDCl3
76.8 CDCl3
61.4
53.0
f1 (ppm)

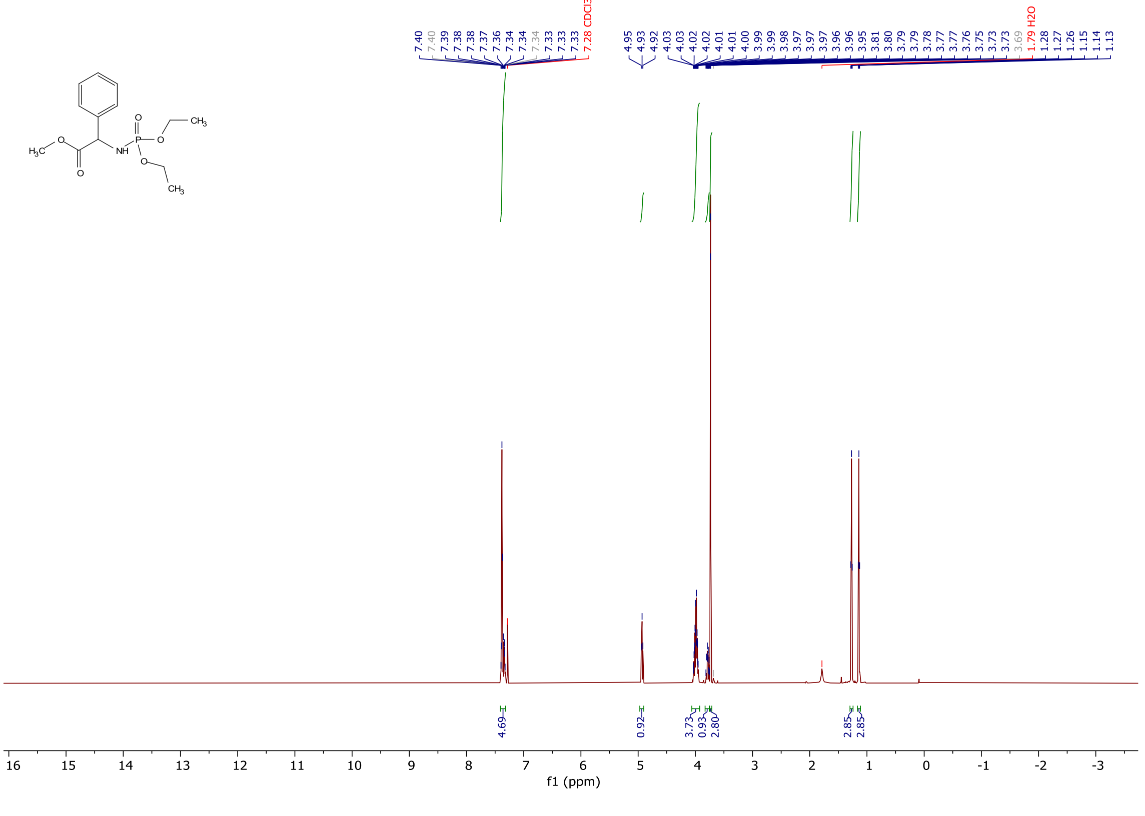
7.28 CDCl3
1.79 H2O
4.69
0.92
3.73
0.93
2.80
2.85
2.85
f1 (ppm)

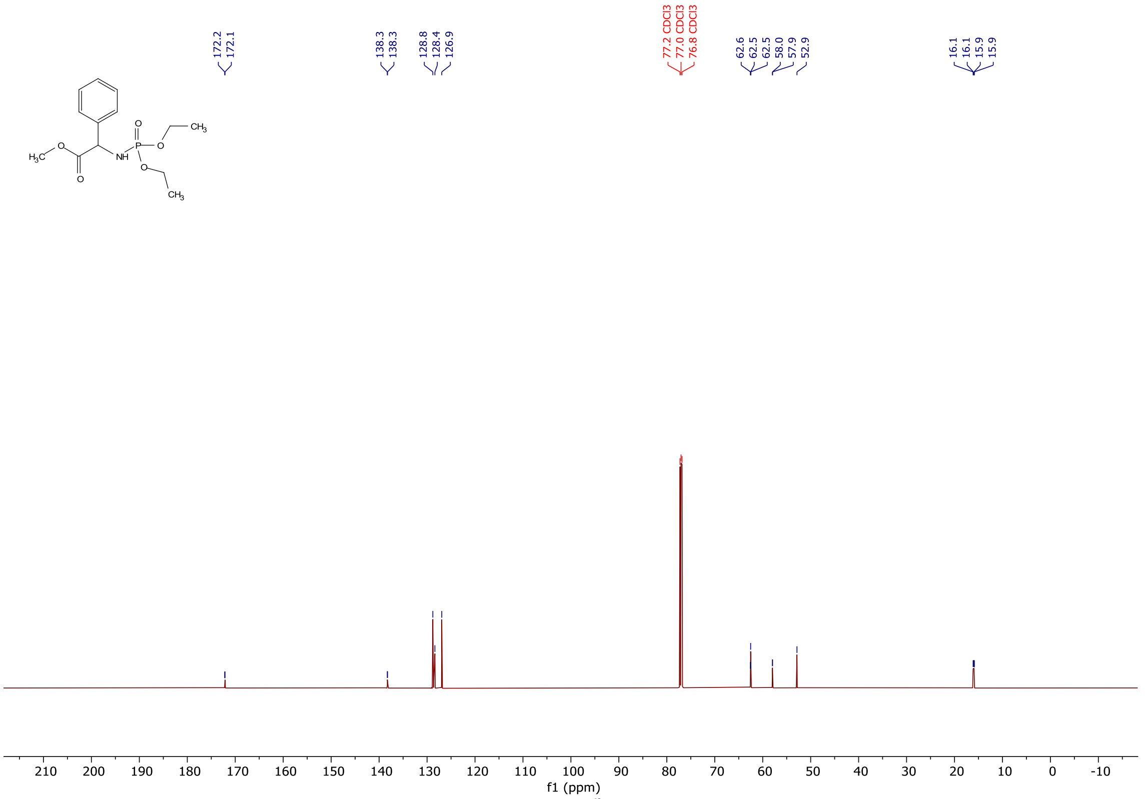
172.2
172.1
138.3
138.3
128.8
128.4
126.9
77.2 CDCl3
77.0 CDCl3
76.8 CDCl3
62.6
62.5
62.5
58.0
57.9
52.9
16.1
16.1
15.9
15.9
f1 (ppm)

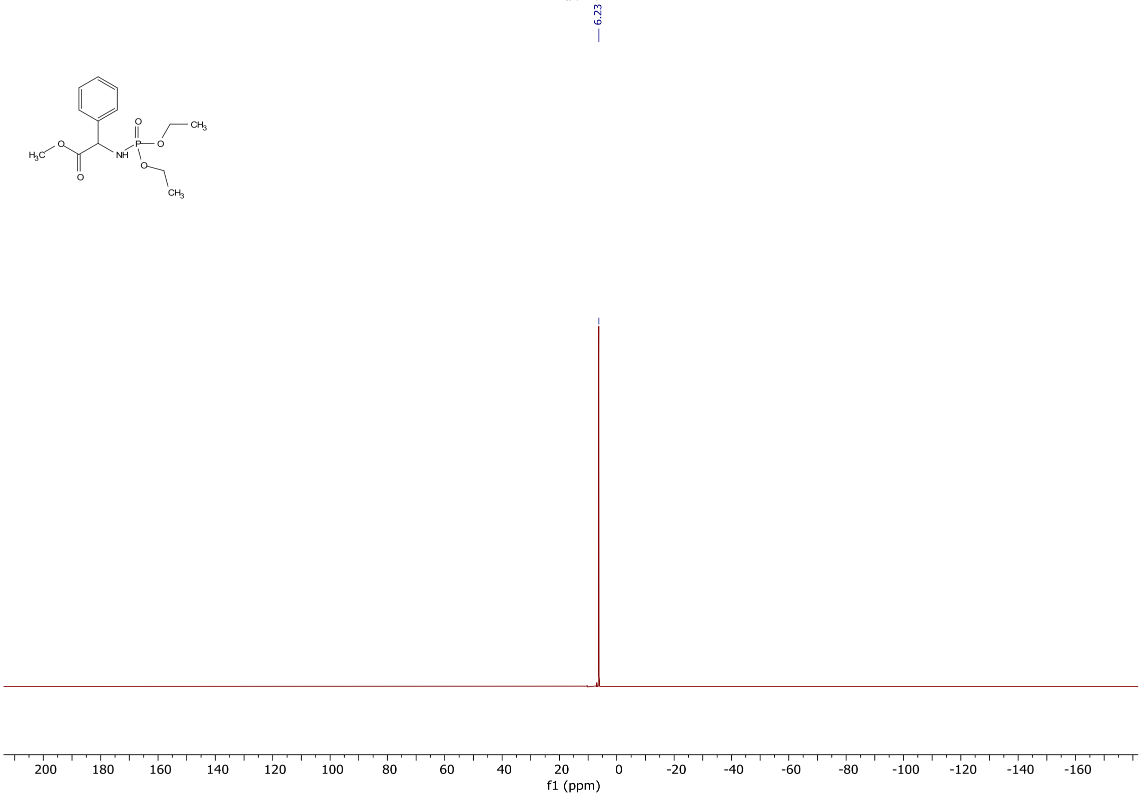
6.23
f1 (ppm)

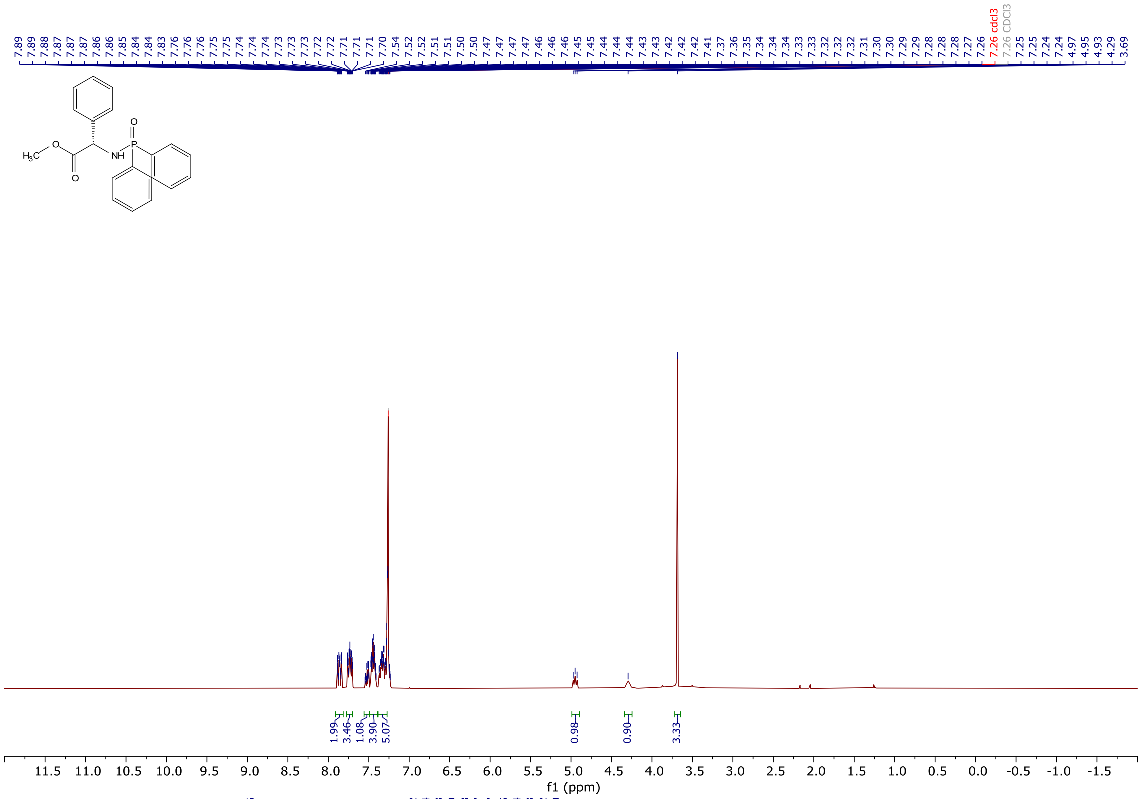
7.89
7.89
7.88
7.87
7.87
7.86
7.86
7.85
7.84
7.84
7.83
7.76
7.76
7.76
7.75
7.75
7.74
7.74
7.74
7.73
7.73
7.73
7.72
7.72
7.71
7.71
7.71
7.70
7.54
7.52
7.52
7.51
7.51
7.50
7.50
7.47
7.47
7.47
7.46
7.46
7.45
7.45
7.44
7.44
7.44
7.43
7.43
7.42
7.42
7.41
7.37
7.36
7.35
7.34
7.34
7.33
7.33
7.32
7.32
7.31
7.30
7.30
7.29
7.29
7.28
7.28
7.28
7.27
7.26 cdcl3
7.26 CDCl3
7.25
7.25
7.24
7.24
4.97
4.95
4.93
4.29
3.69
H3C
O
O
NH
P
O
1.99
3.46
1.08
3.90
5.07
0.98
0.90
3.33
11.5 11.0 10.5 10.0 9.5 9.0 8.5 8.0 7.5 7.0 6.5 6.0 5.5 5.0 4.5 4.0 3.5 3.0 2.5 2.0 1.5 1.0 0.5 0.0 -0.5 -1.0 -1.5
f1 (ppm)

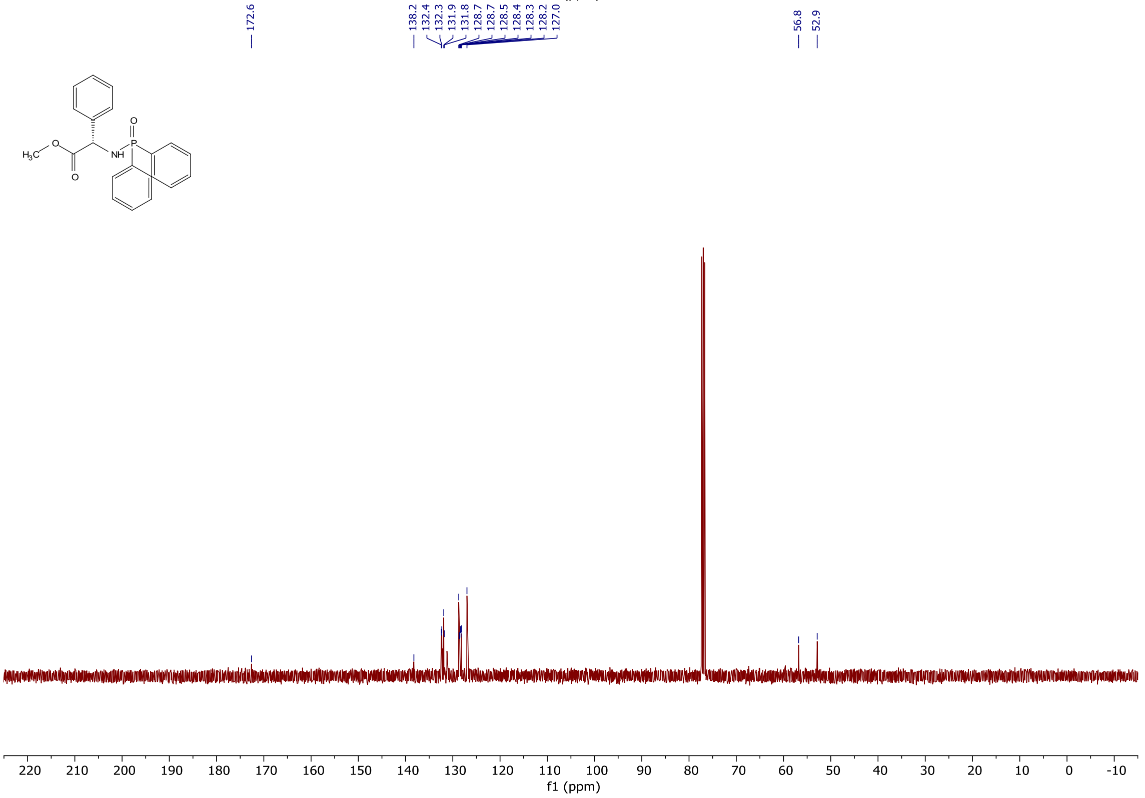
172.6
138.2
132.4
132.3
131.9
131.8
128.7
128.7
128.5
128.4
128.3
128.2
127.0
56.8
52.9
H3C
O
O
NH
P
O
220 210 200 190 180 170 160 150 140 130 120 110 100 90 80 70 60 50 40 30 20 10 0 -10
f1 (ppm)

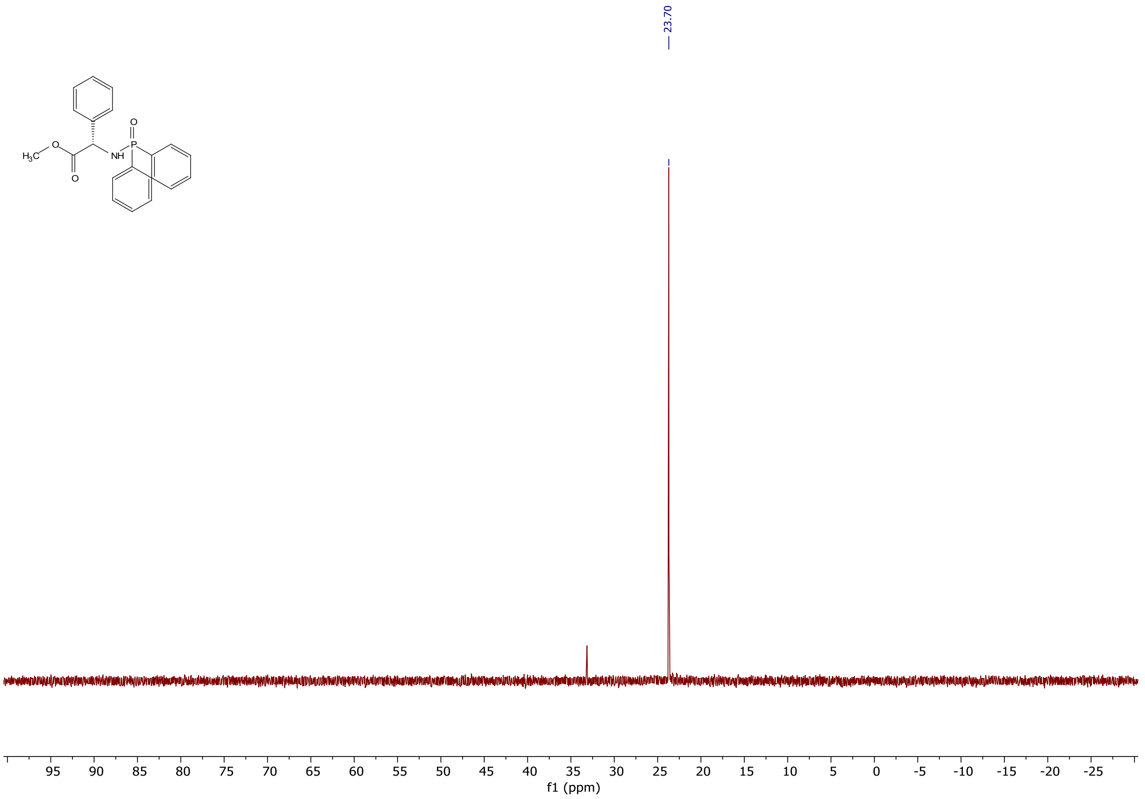

23.70
H_3C
O
NH
P
O
95 90 85 80 75 70 65 60 55 50 45 40 35 30 25 20 15 10 5 0 -5 -10 -15 -20 -25
f1 (ppm)

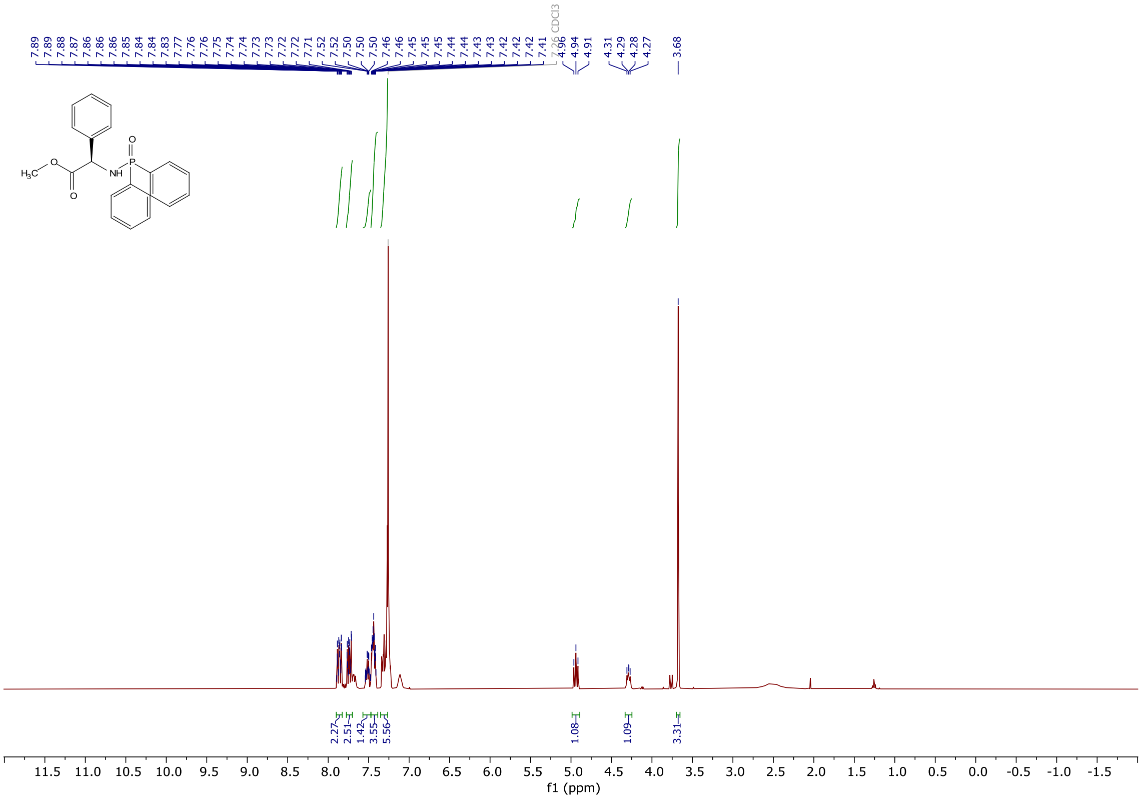

7.89 7.89 7.88 7.87 7.86 7.86 7.86 7.85 7.84 7.84 7.83 7.77 7.76 7.76 7.75 7.74 7.74 7.73 7.73 7.72 7.72 7.71 7.52 7.52 7.50 7.50 7.46 7.45 7.45 7.45 7.44 7.44 7.43 7.43 7.42 7.42 7.41
7.26 CDCl3
4.96 4.94 4.91
4.31 4.29 4.28 4.27
3.68
H_3C
O
NH
P
O
2.27 2.51 1.42 3.55 5.56
1.08
1.09
3.31
11.5 11.0 10.5 10.0 9.5 9.0 8.5 8.0 7.5 7.0 6.5 6.0 5.5 5.0 4.5 4.0 3.5 3.0 2.5 2.0 1.5 1.0 0.5 0.0 -0.5 -1.0 -1.5
f1 (ppm)

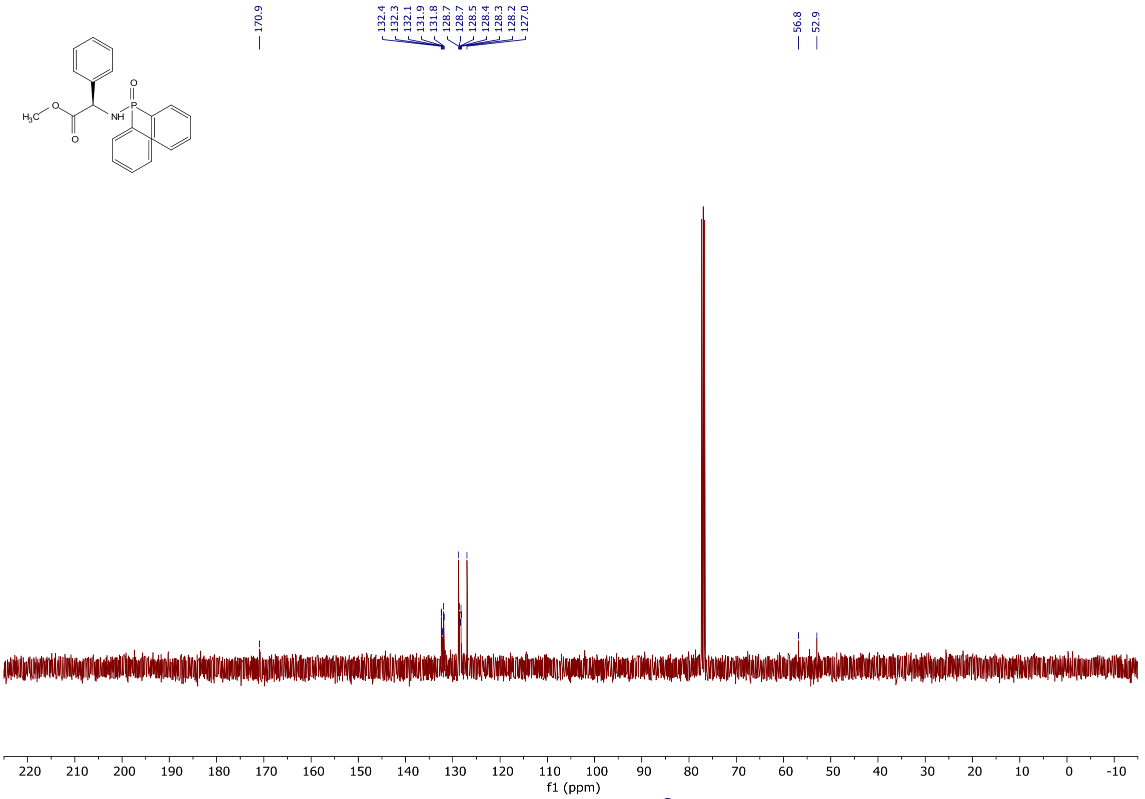
170.9
132.4
132.3
132.1
131.9
131.8
128.7
128.7
128.5
128.4
128.3
128.2
127.0
56.8
52.9
H3C
O
O
NH
P
O
220 210 200 190 180 170 160 150 140 130 120 110 100 90 80 70 60 50 40 30 20 10 0 -10
f1 (ppm)

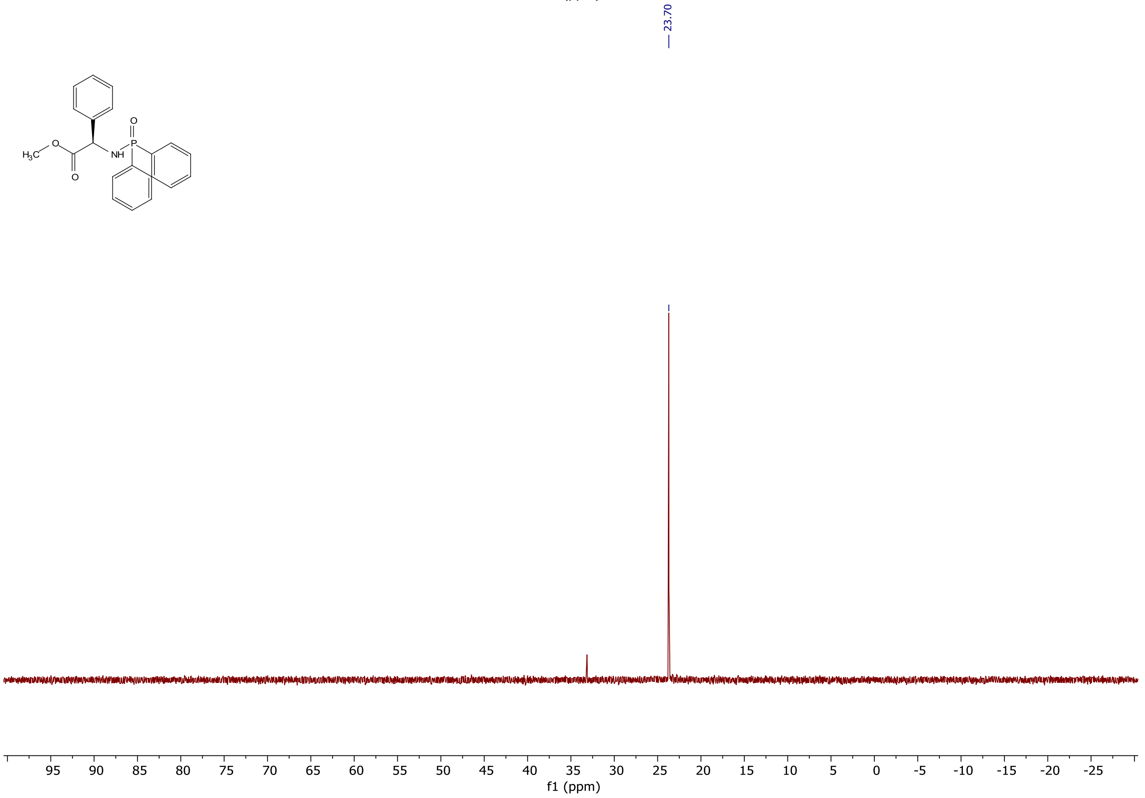
23.70
H3C
O
O
NH
P
O
95 90 85 80 75 70 65 60 55 50 45 40 35 30 25 20 15 10 5 0 -5 -10 -15 -20 -25
f1 (ppm)

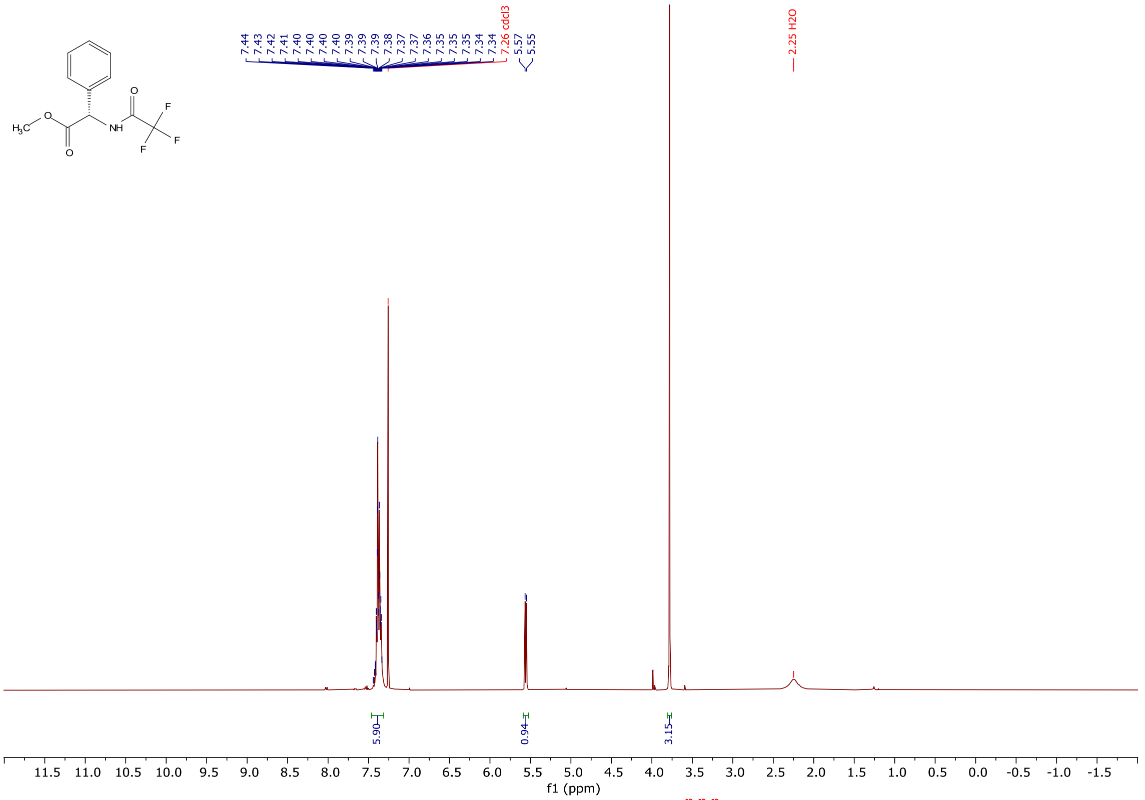
7.44
7.43
7.42
7.41
7.40
7.40
7.40
7.40
7.39
7.39
7.39
7.38
7.37
7.37
7.36
7.35
7.35
7.35
7.34
7.34
7.26 cdcl3
5.57
5.55
2.25 H2O
5.90
0.94
3.15
f1 (ppm)

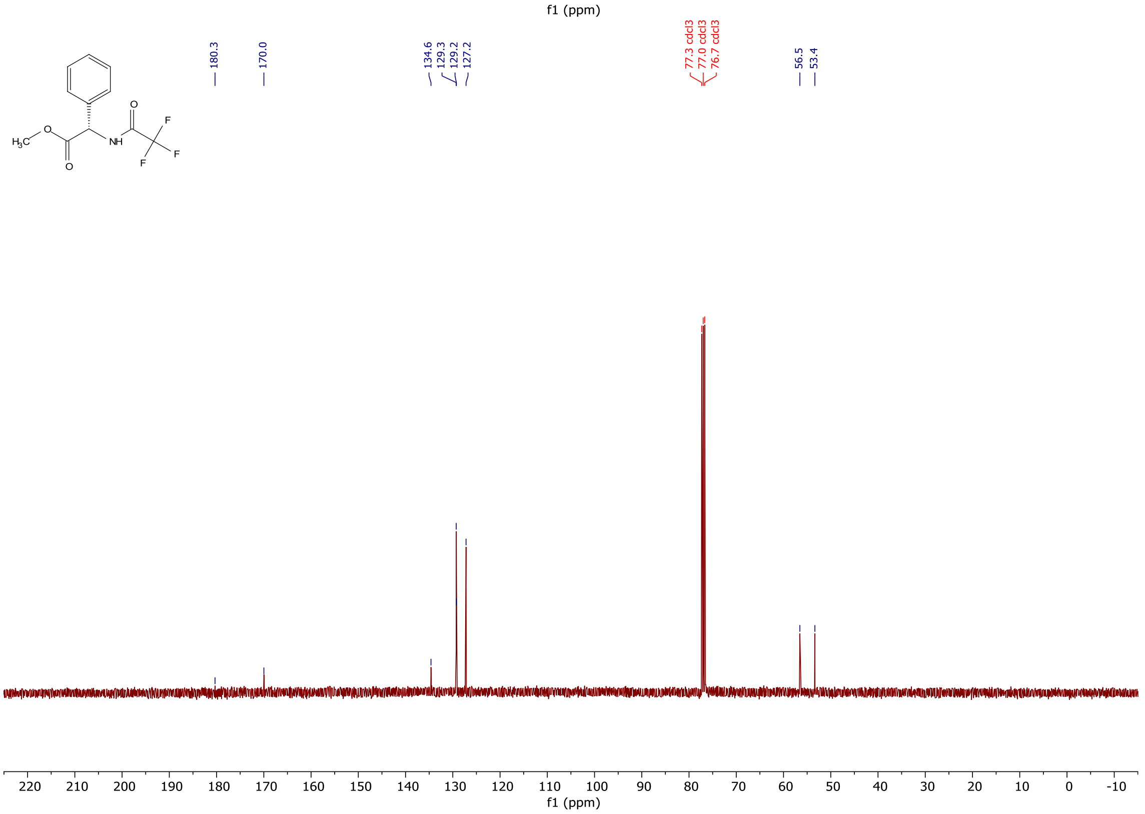
180.3
170.0
134.6
129.3
129.2
127.2
77.3 cdcl3
77.0 cdcl3
76.7 cdcl3
56.5
53.4
f1 (ppm)

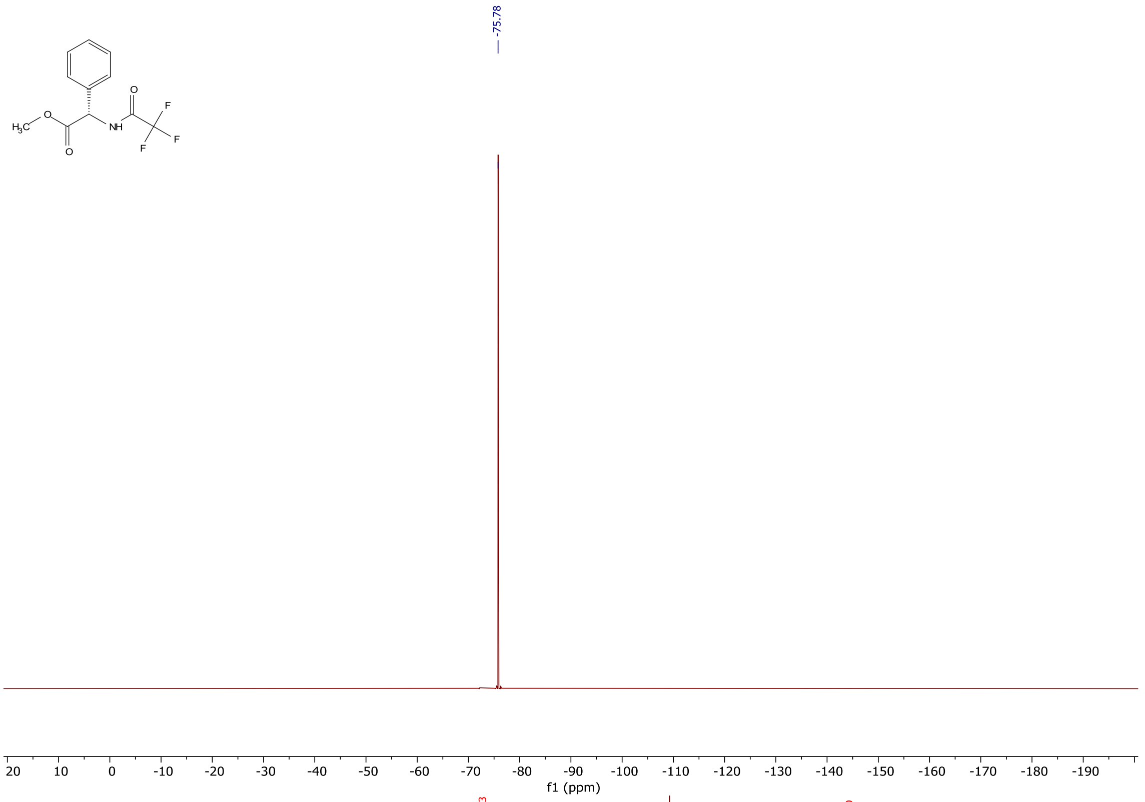
-75.78
H3C O NH O F F F O
20 10 0 -10 -20 -30 -40 -50 -60 -70 -80 -90 -100 -110 -120 -130 -140 -150 -160 -170 -180 -190
f1 (ppm)

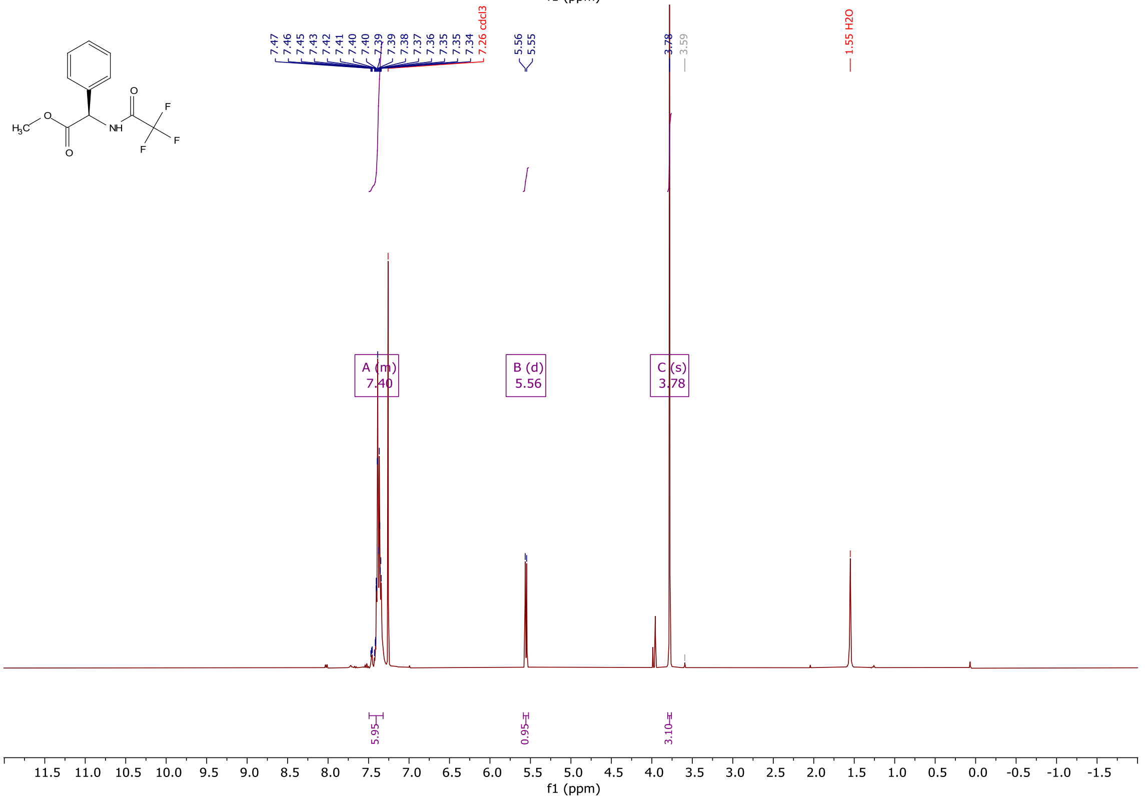
7.47 7.46 7.45 7.43 7.42 7.41 7.40 7.40 7.39 7.39 7.38 7.37 7.36 7.35 7.35 7.34
7.26 cdcl3
5.56 5.55
3.78
3.59
1.55 H2O
H3C O NH O F F F O
A (m) 7.40
B (d) 5.56
C (s) 3.78
5.95
0.95
3.10
11.5 11.0 10.5 10.0 9.5 9.0 8.5 8.0 7.5 7.0 6.5 6.0 5.5 5.0 4.5 4.0 3.5 3.0 2.5 2.0 1.5 1.0 0.5 0.0 -0.5 -1.0 -1.5
f1 (ppm)

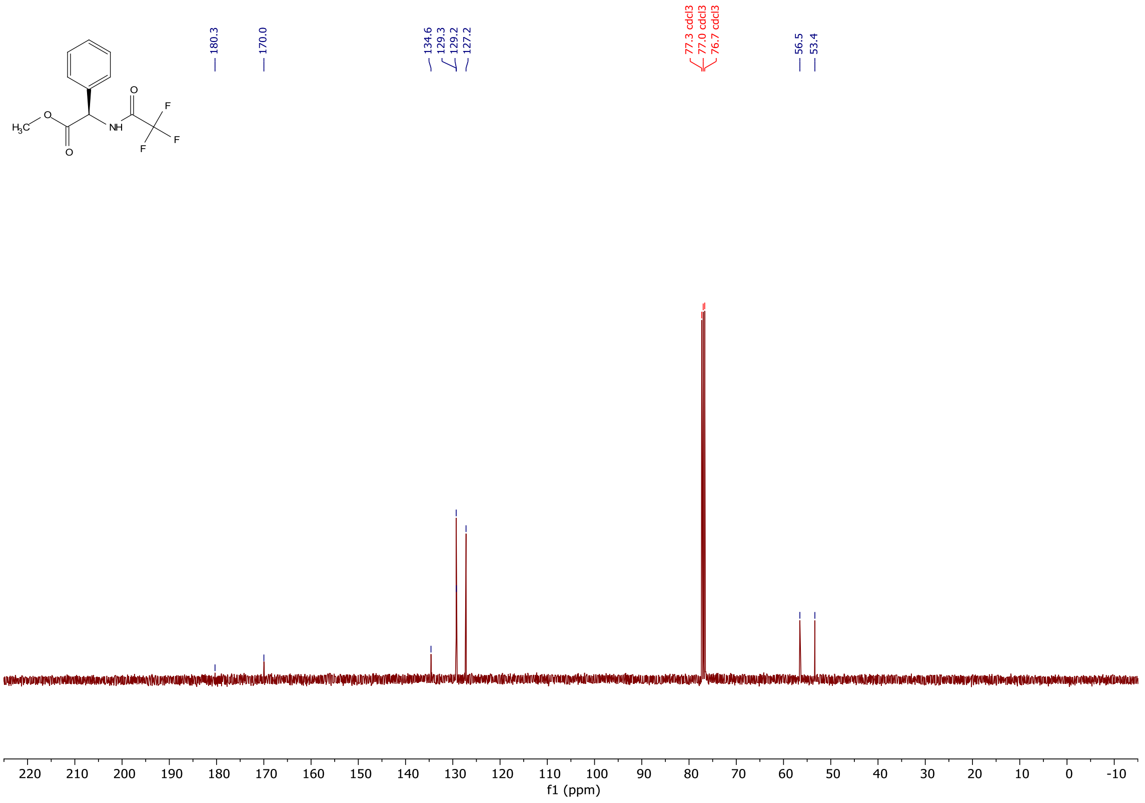
180.3
170.0
134.6
129.3
129.2
127.2
77.3 cdcl3
77.0 cdcl3
76.7 cdcl3
56.5
53.4
f1 (ppm)

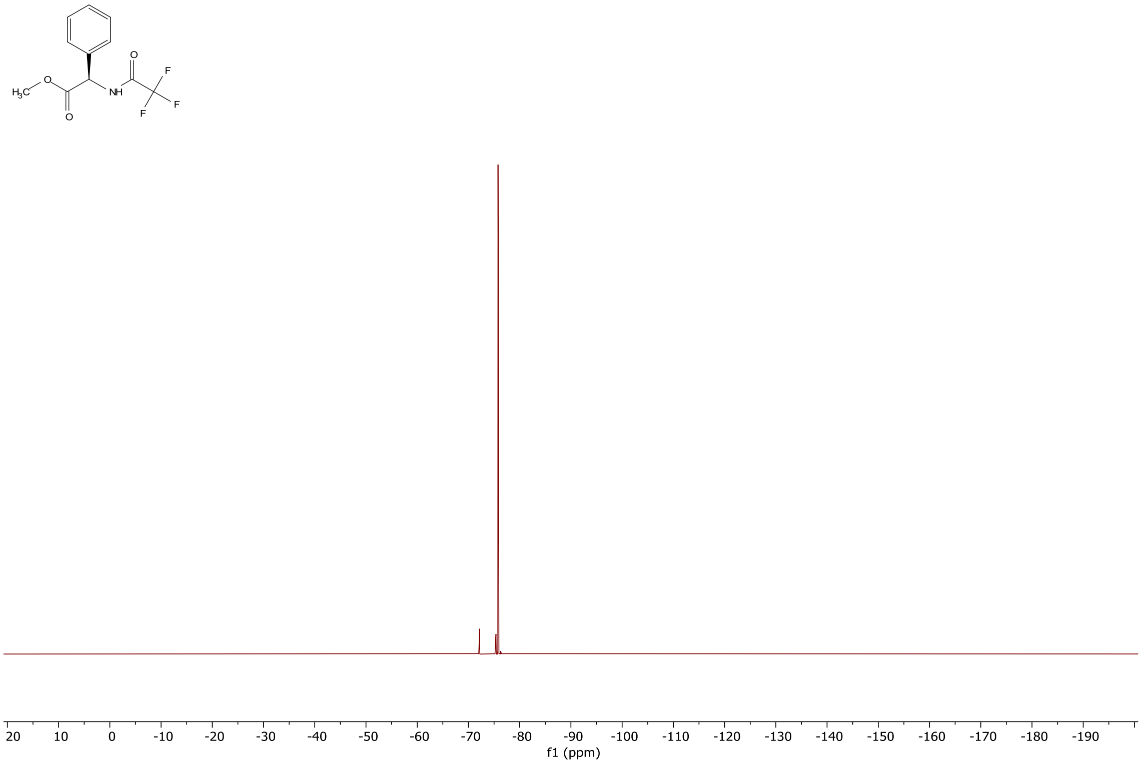
f1 (ppm)

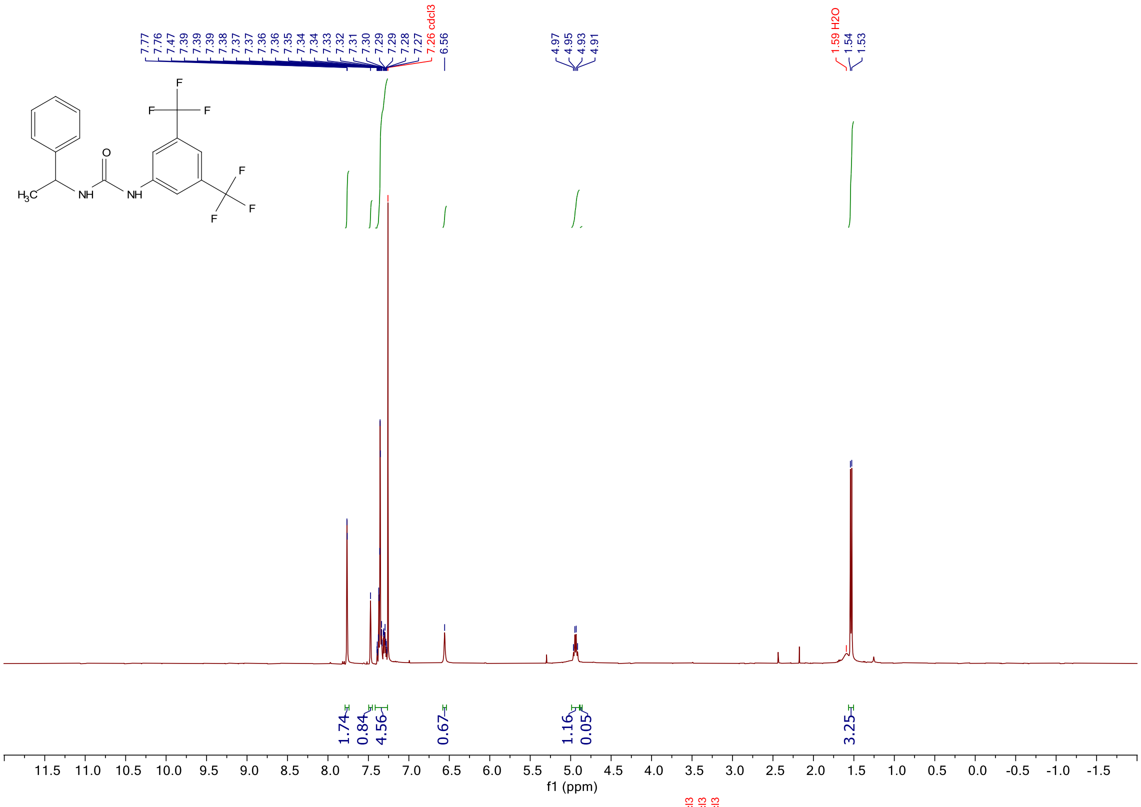
7.77
7.76
7.47
7.39
7.39
7.39
7.38
7.37
7.37
7.36
7.36
7.35
7.34
7.34
7.33
7.32
7.31
7.30
7.29
7.29
7.28
7.27
7.26 cdcl3
6.56
4.97
4.95
4.93
4.91
1.59 H2O
1.54
1.53
1.74
0.84
4.56
0.67
1.16
0.05
3.25
f1 (ppm)

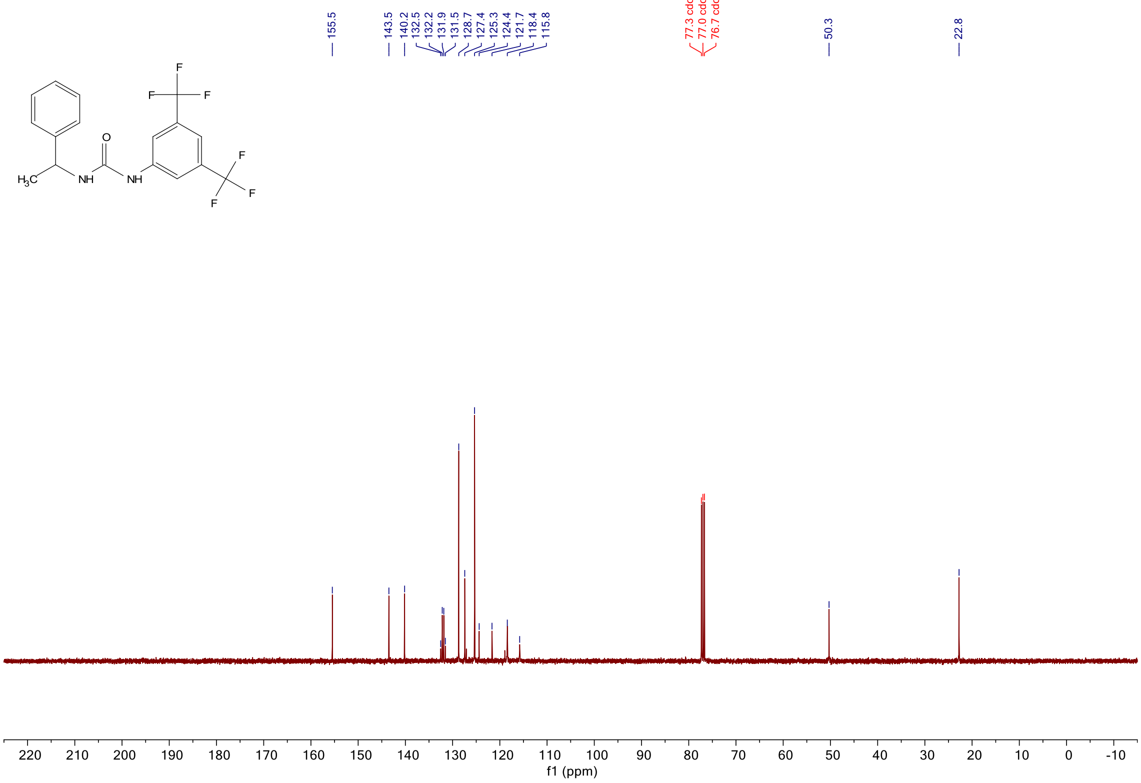
155.5
143.5
140.2
132.5
132.2
131.9
131.5
128.7
127.4
125.3
124.4
121.7
118.4
115.8
77.3 cdcl3
77.0 cdcl3
76.7 cdcl3
50.3
22.8
f1 (ppm)

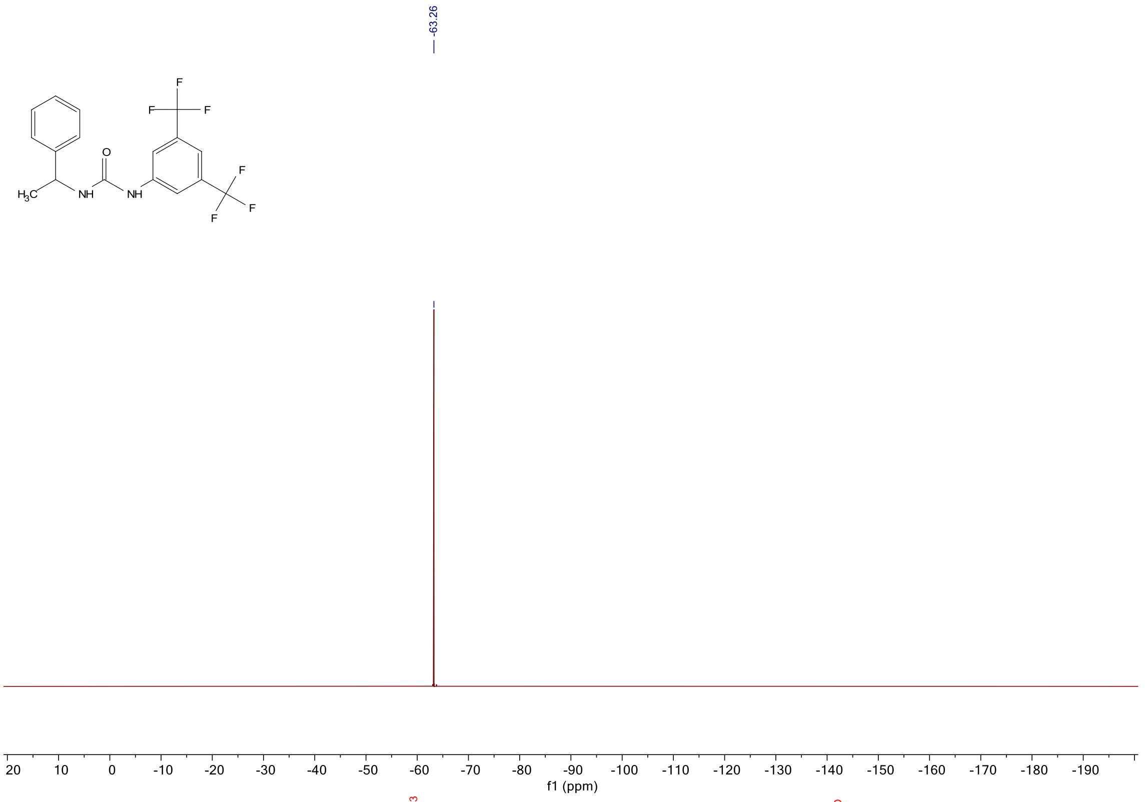
-63.26
f1 (ppm)

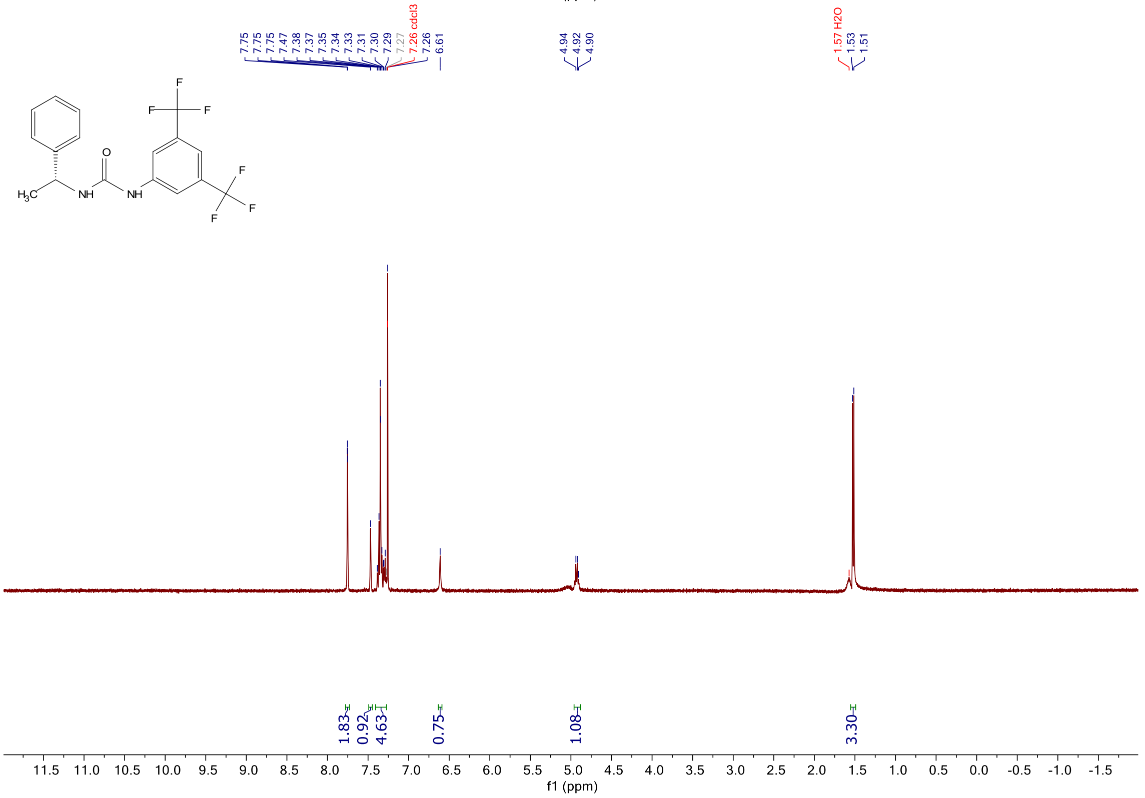
7.75
7.75
7.75
7.47
7.38
7.37
7.35
7.34
7.33
7.31
7.30
7.29
7.27
7.26 cdcl3
7.26
6.61
4.94
4.92
4.90
1.57 H2O
1.53
1.51
1.83
0.92
4.63
0.75
1.08
3.30
f1 (ppm)

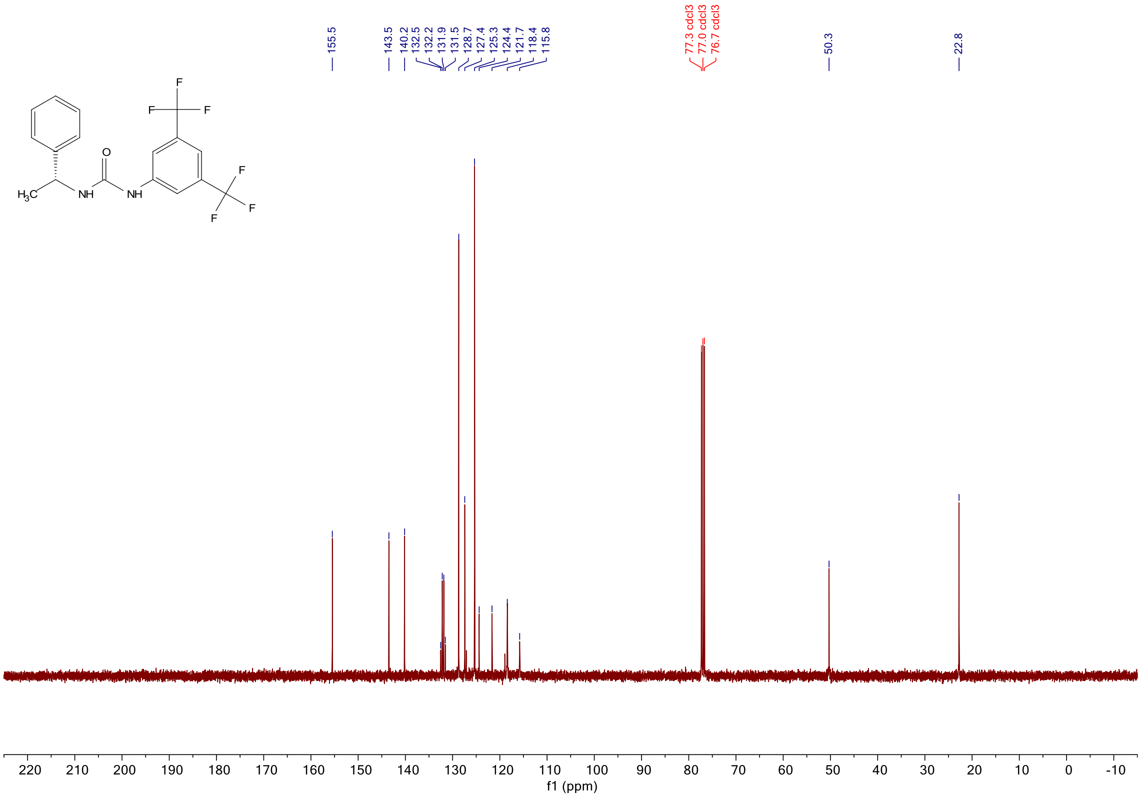
155.5
143.5
140.2
132.5
132.2
131.9
131.5
128.7
127.4
125.3
124.4
121.7
118.4
115.8
77.3 cdcl3
77.0 cdcl3
76.7 cdcl3
50.3
22.8
f1 (ppm)

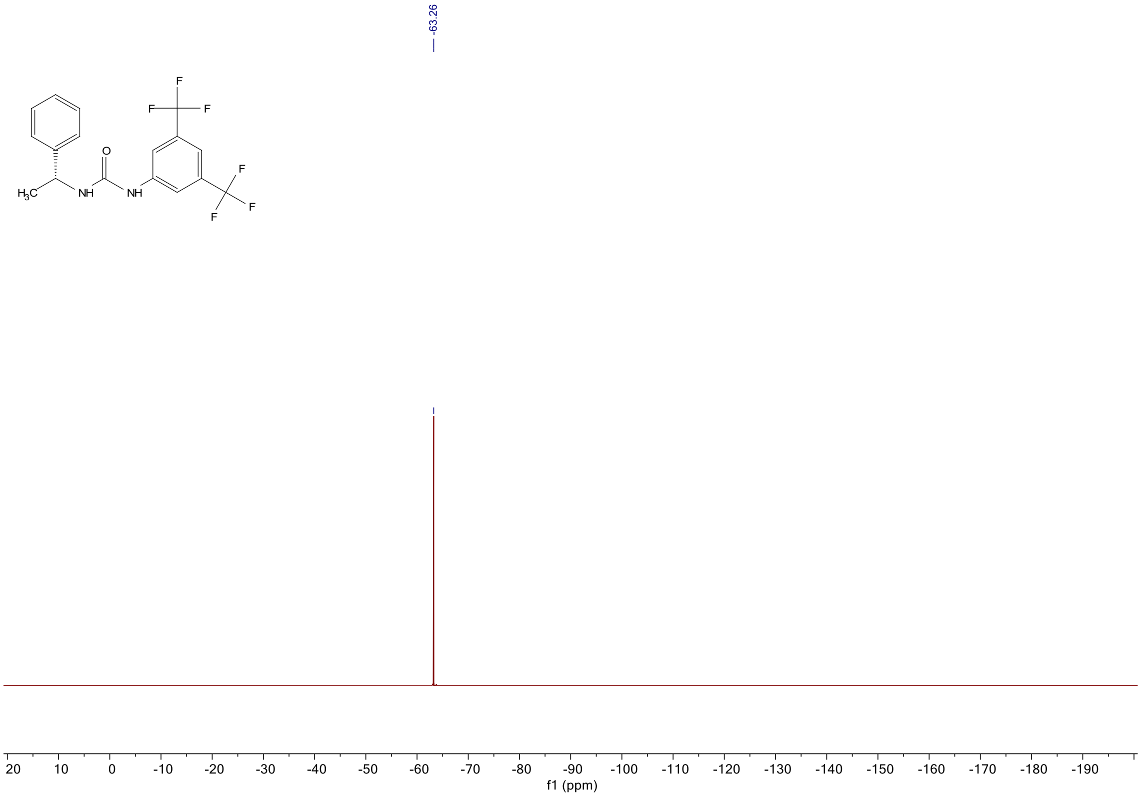
-63.26
f1 (ppm)

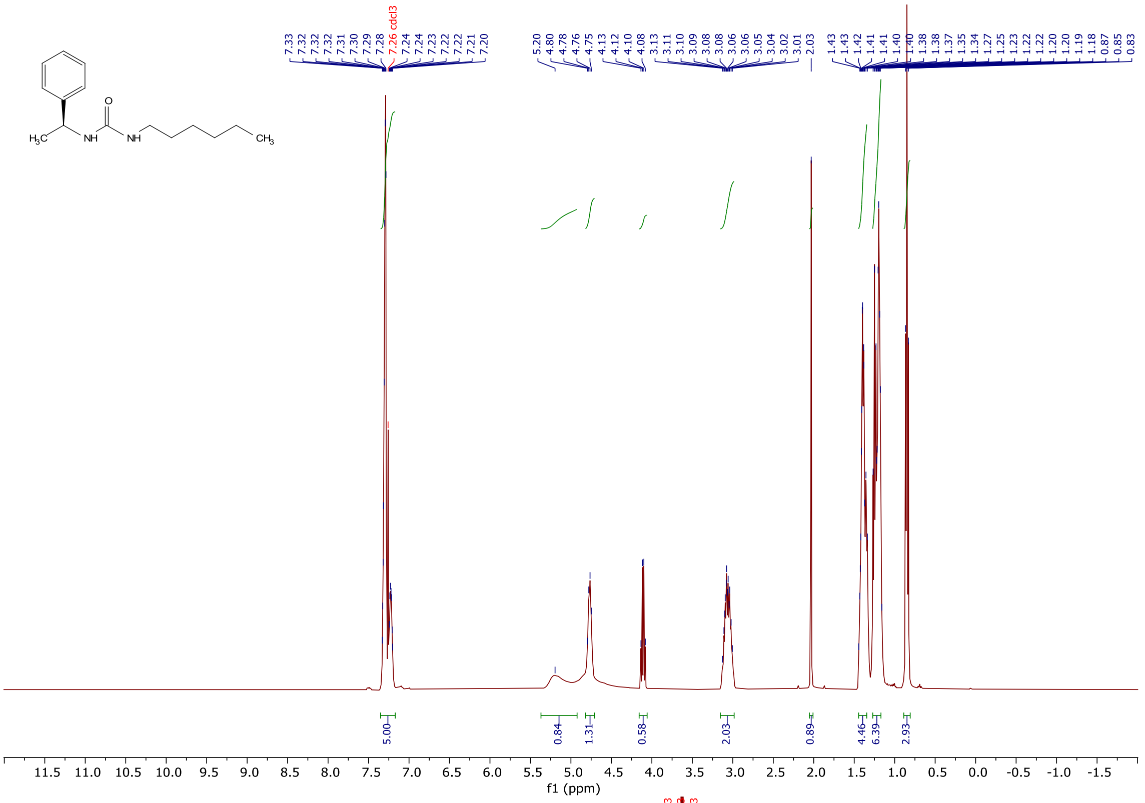

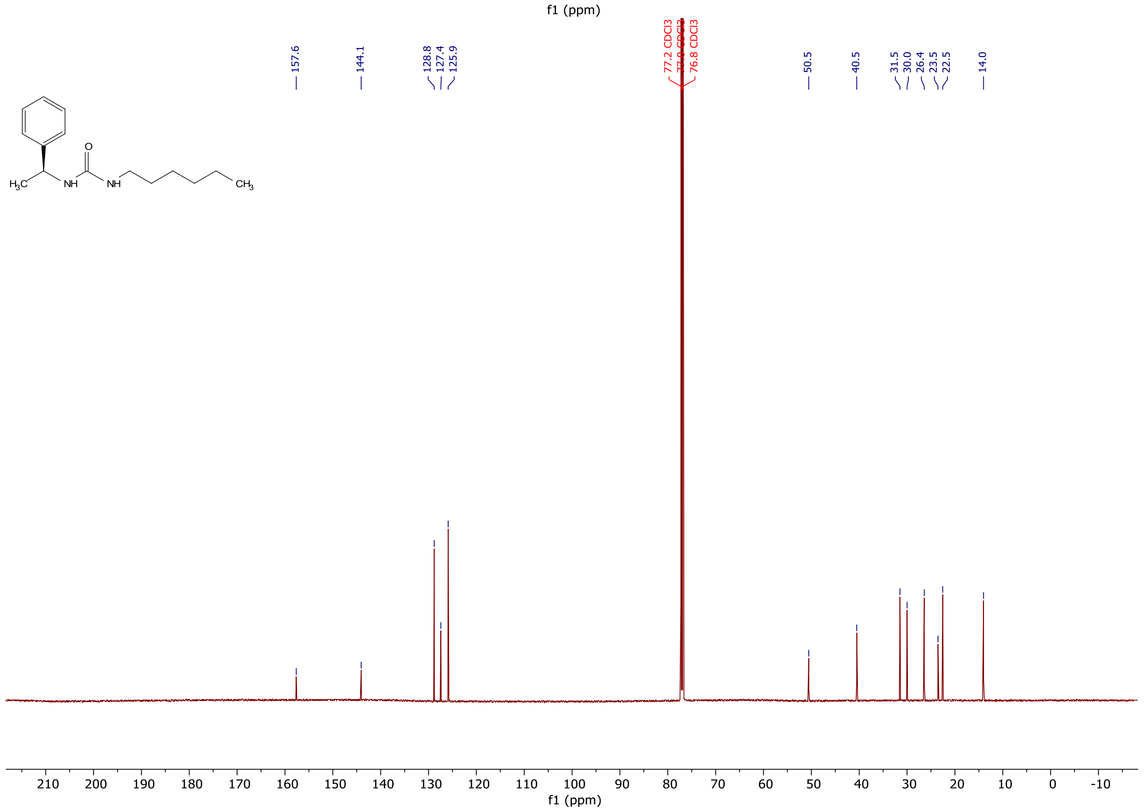

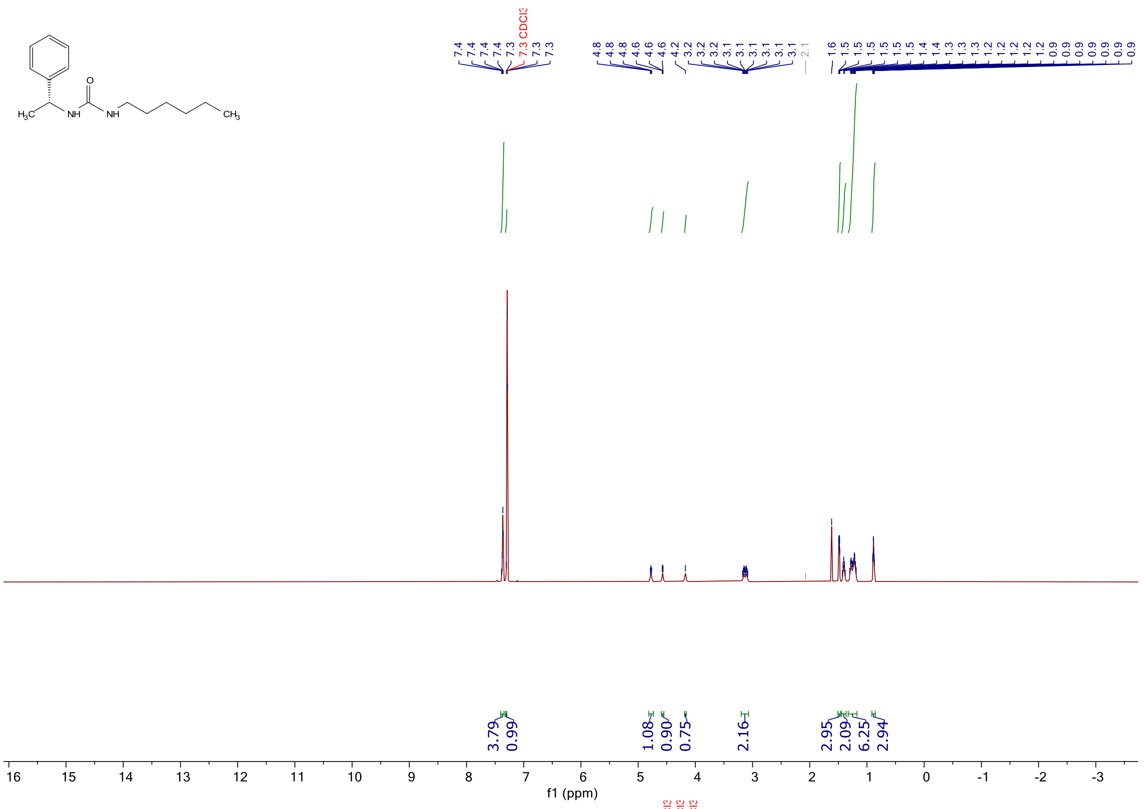

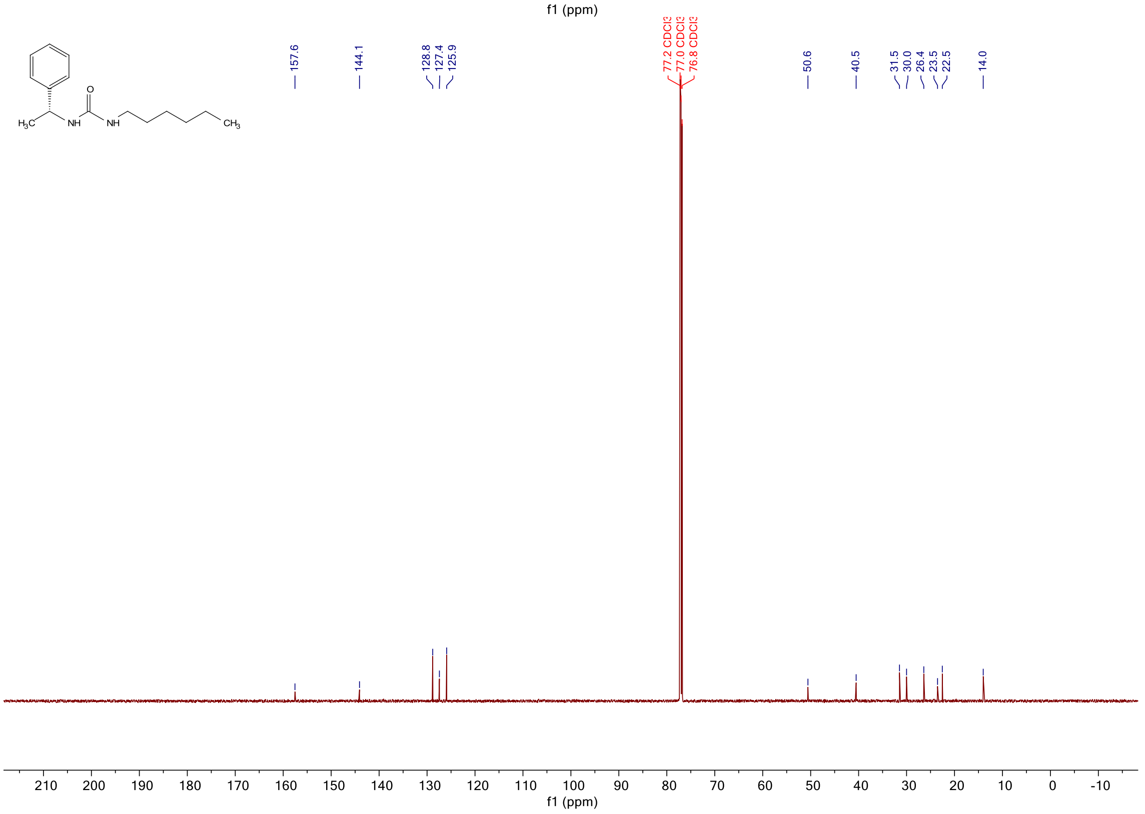

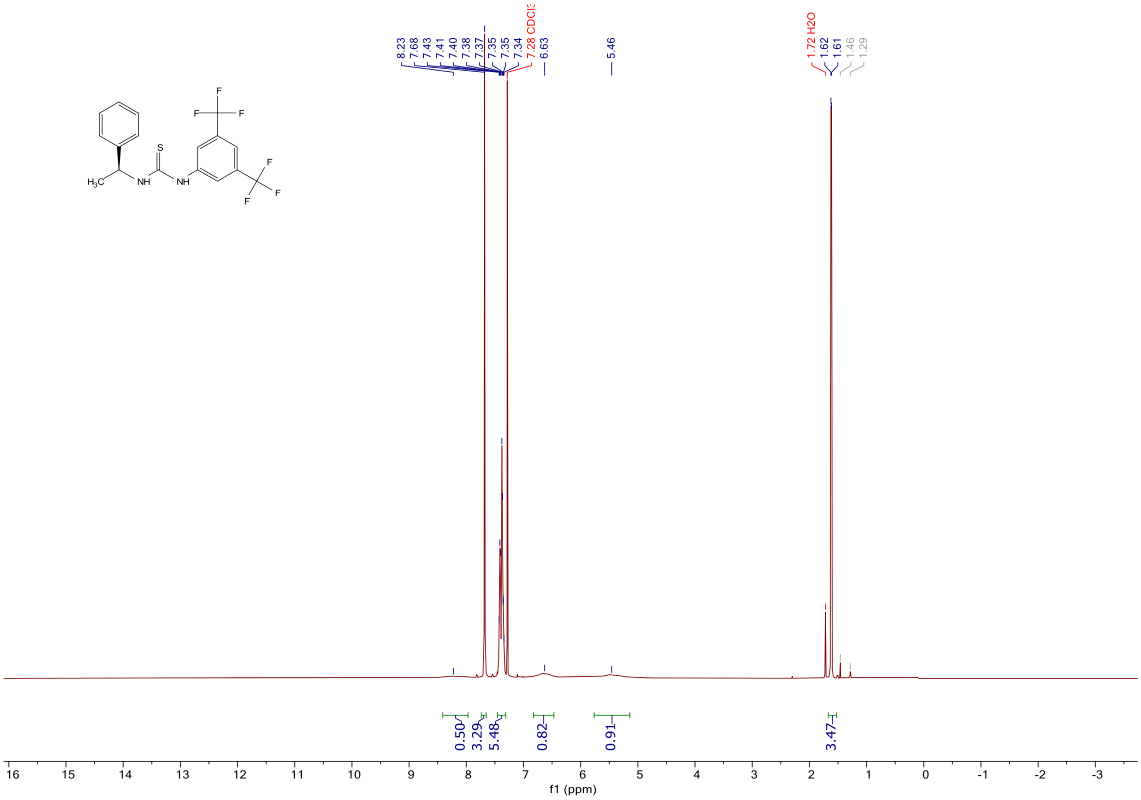

8.23
7.68
7.43
7.41
7.40
7.38
7.37
7.35
7.35
7.34
7.28 CDCl3
6.63
5.46
1.72 H2O
1.62
1.61
1.46
1.29
0.50
3.29
5.48
0.82
0.91
3.47
f1 (ppm)

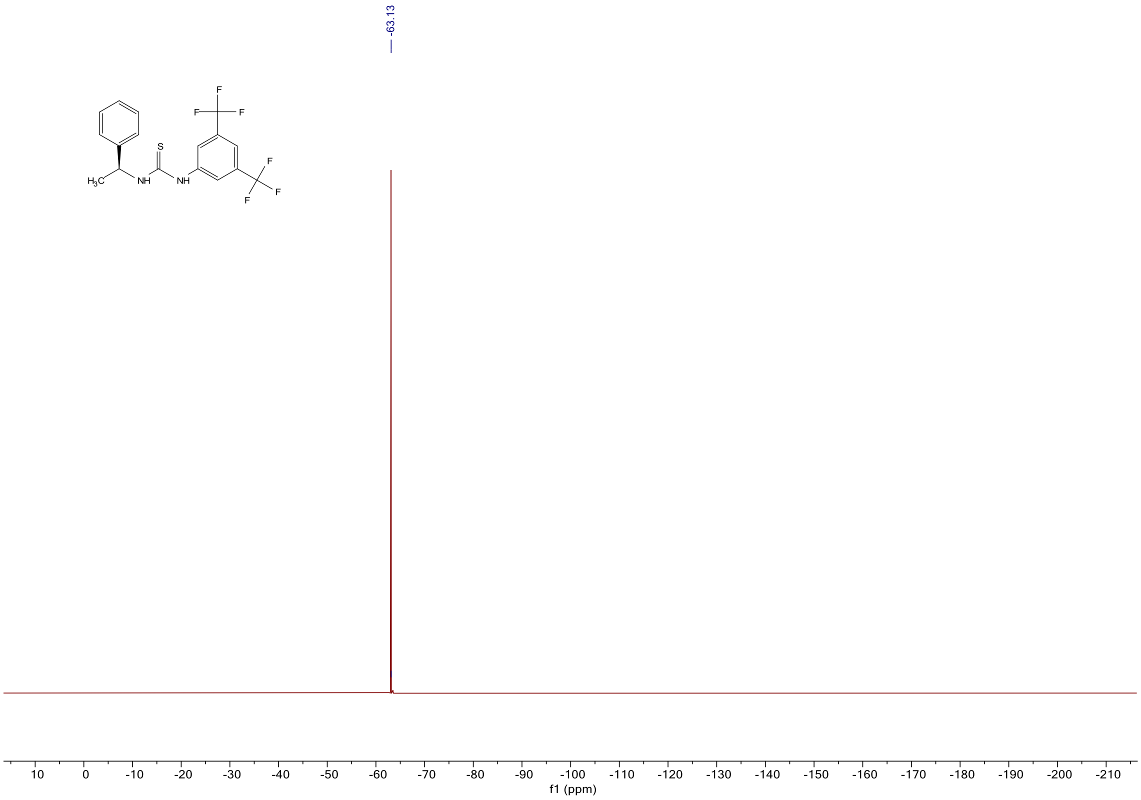

-63.13
f1 (ppm)

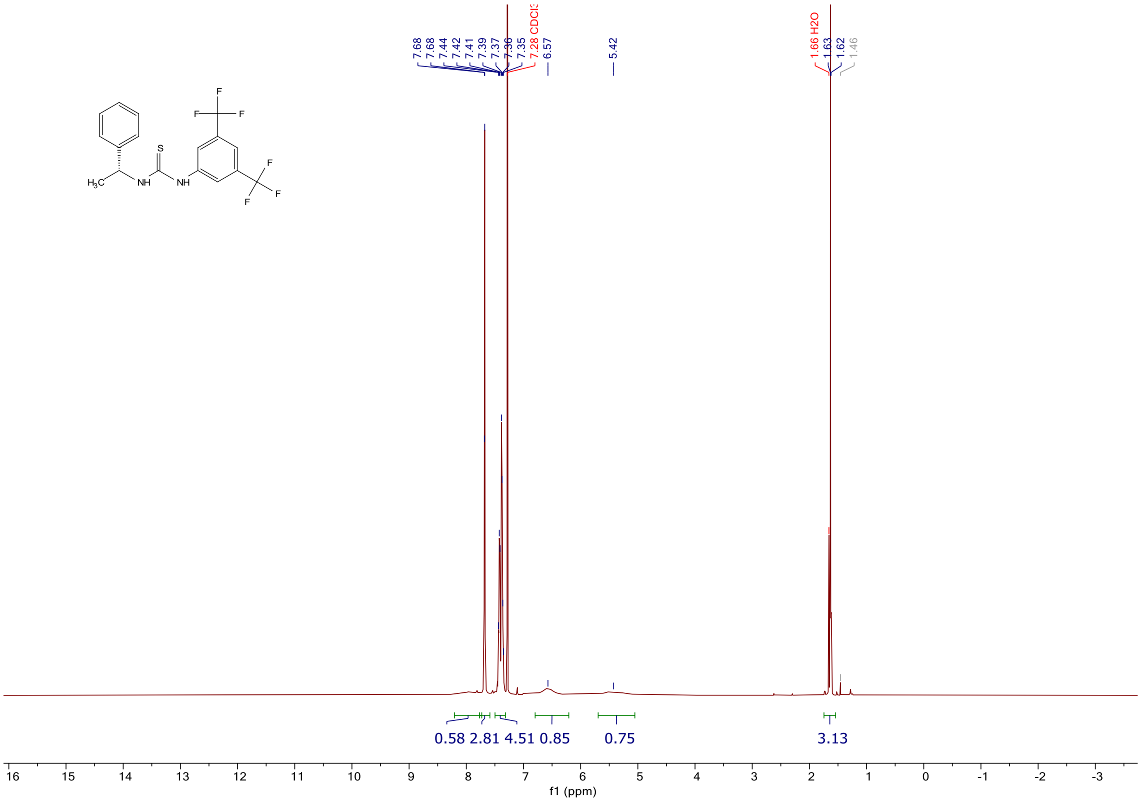
7.68
7.68
7.44
7.42
7.41
7.39
7.37
7.36
7.35
7.28 CDCl3
6.57
5.42
1.66 H2O
1.63
1.62
1.46
0.58 2.81 4.51 0.85
0.75
3.13
f1 (ppm)

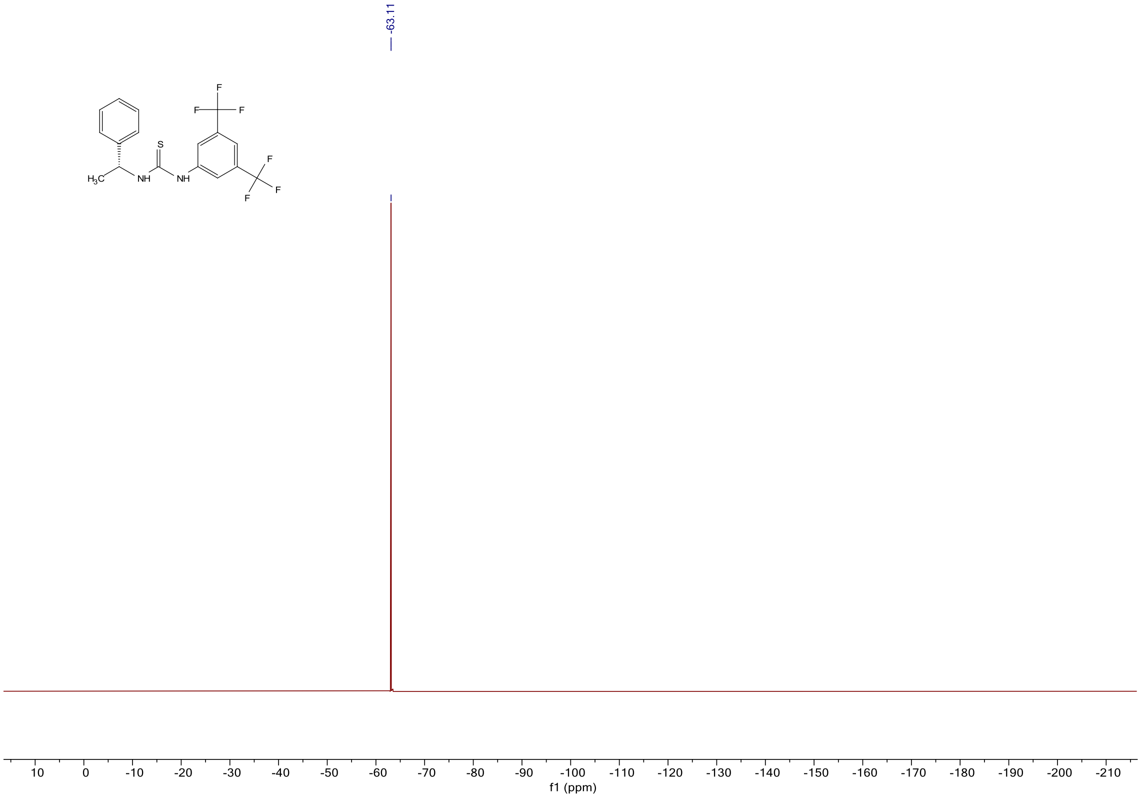
-63.11
f1 (ppm)

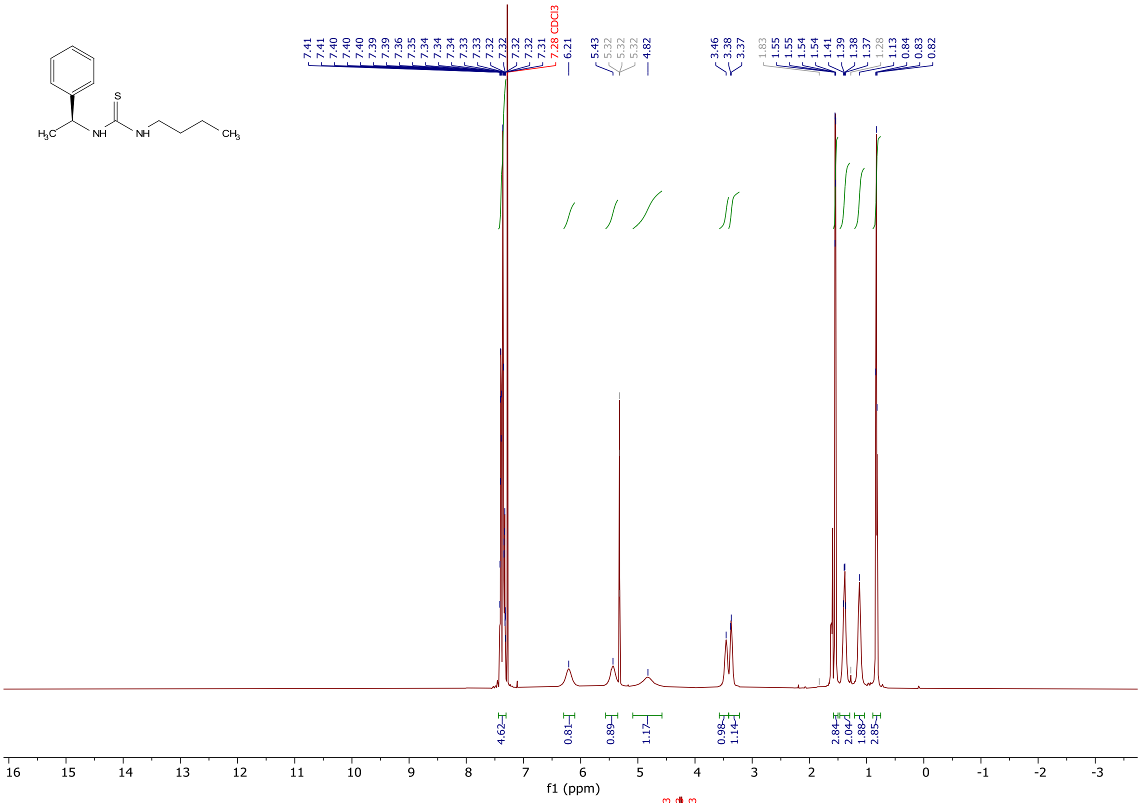
S
H3C
NH
NH
CH3
7.41 7.41 7.40 7.40 7.40 7.39 7.39 7.36 7.35 7.34 7.34 7.34 7.33 7.33 7.32 7.32 7.32 7.32 7.32 7.31
7.28 CDCl3
6.21
5.43 5.32 5.32 5.32
4.82
3.46 3.38 3.37
1.83 1.55 1.55 1.54 1.54 1.41 1.39 1.38 1.37 1.28 1.13 0.84 0.83 0.82
4.62 0.81 0.89 1.17 0.98 1.14 2.84 2.04 1.88 2.85
16 15 14 13 12 11 10 9 8 7 6 5 4 3 2 1 0 -1 -2 -3
f1 (ppm)

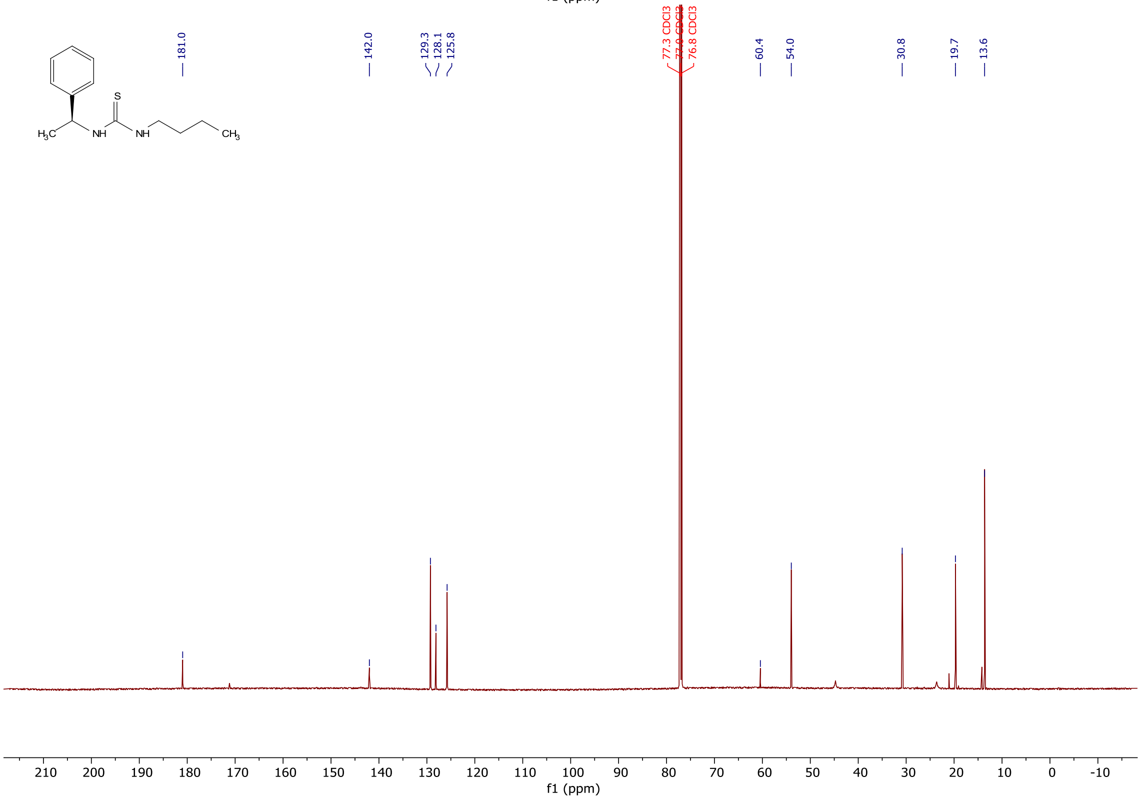
S
H3C
NH
NH
CH3
181.0
142.0
129.3 128.1 125.8
77.3 CDCl3
77.0 CDCl3
76.8 CDCl3
60.4
54.0
30.8
19.7
13.6
210 200 190 180 170 160 150 140 130 120 110 100 90 80 70 60 50 40 30 20 10 0 -10
f1 (ppm)

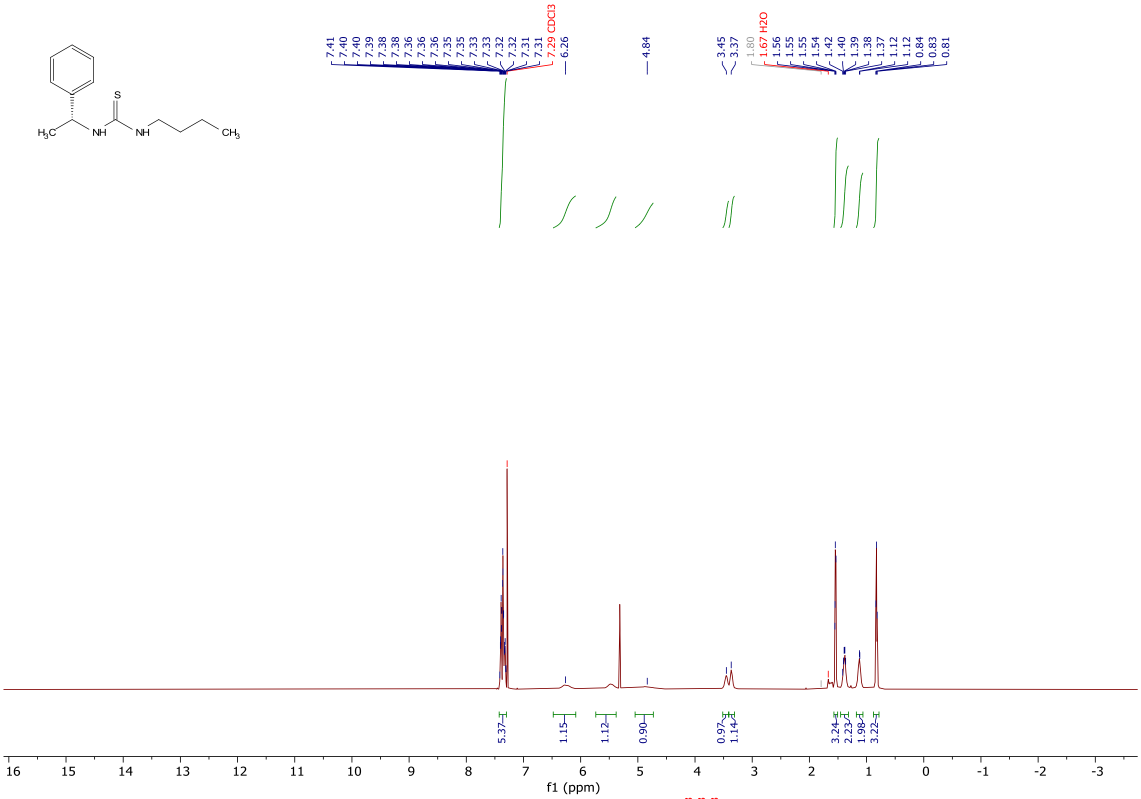

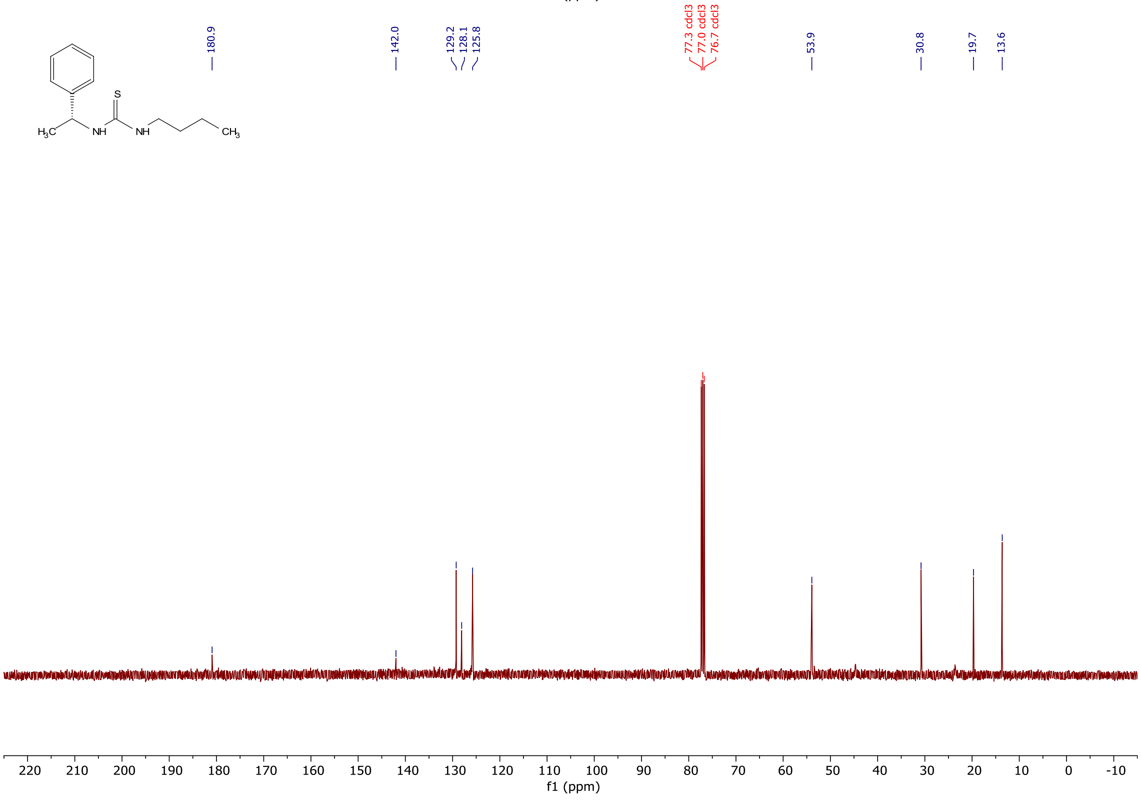

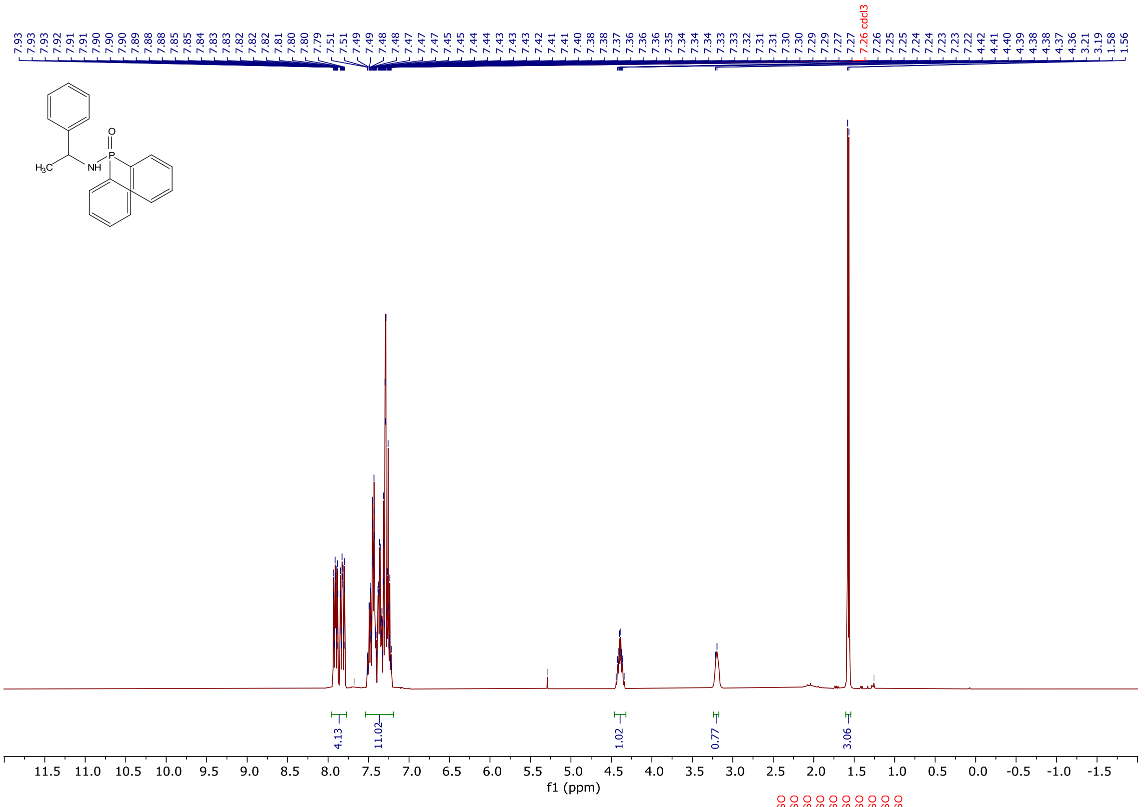

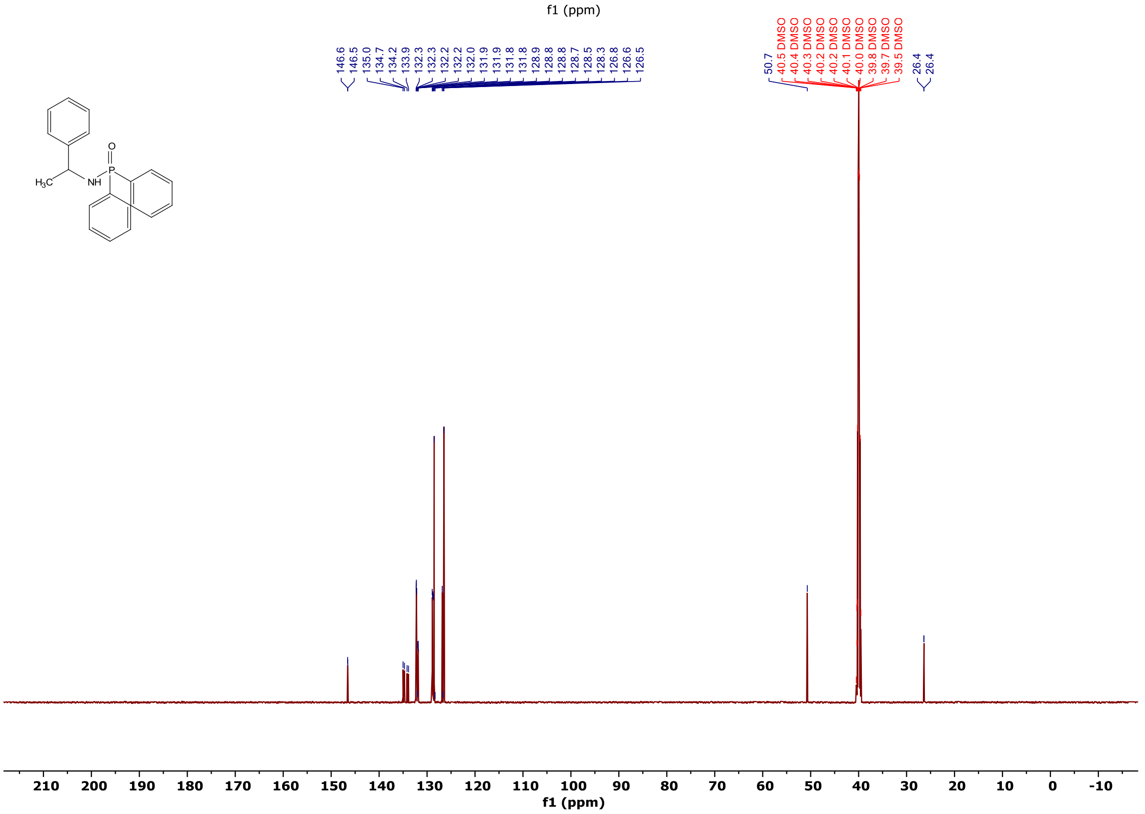

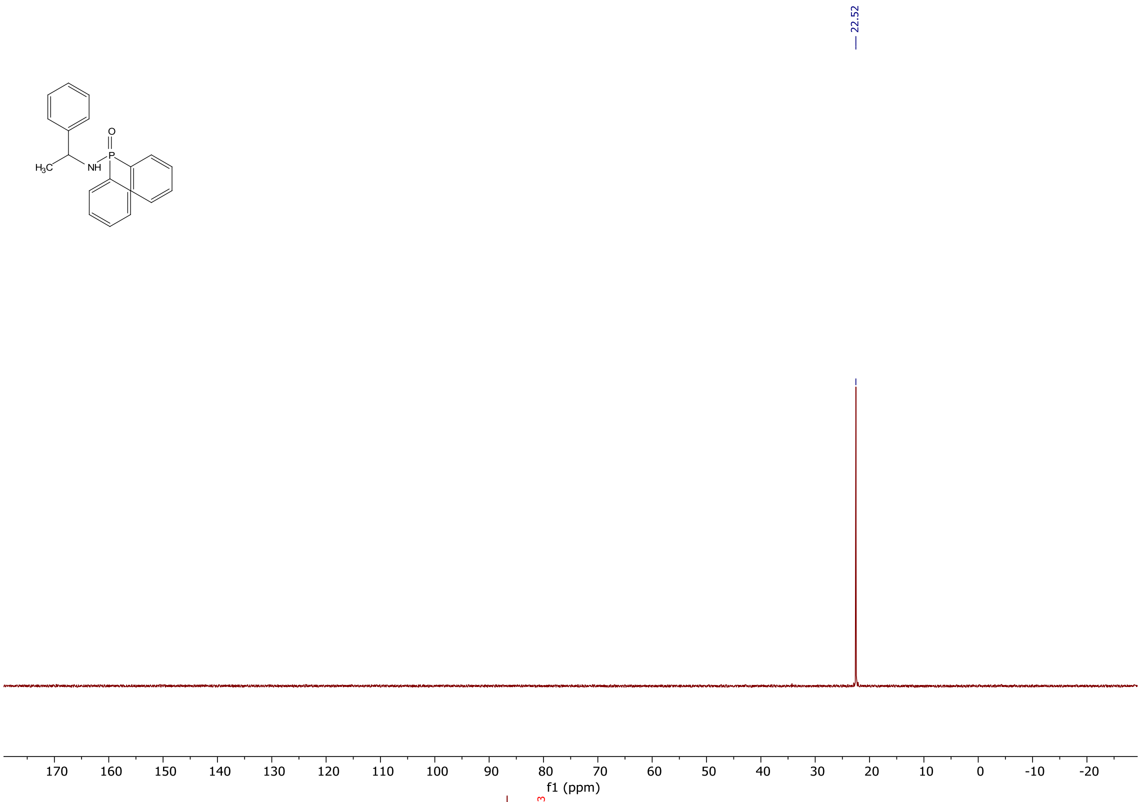
22.52
H3C
NH
P
O
170 160 150 140 130 120 110 100 90 80 70 60 50 40 30 20 10 0 -10 -20
f1 (ppm)

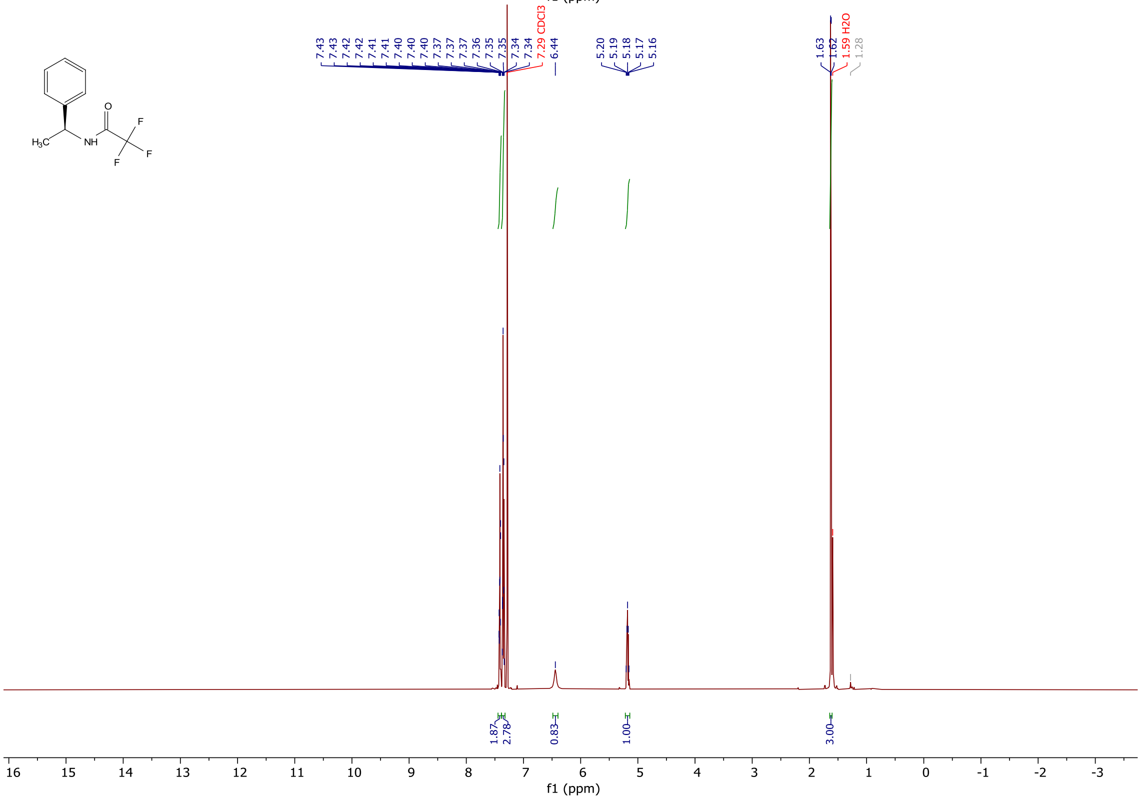
7.43 7.43 7.42 7.41 7.41 7.40 7.40 7.40 7.37 7.37 7.37 7.36 7.35 7.35 7.34 7.34
7.29 CDCl3
6.44
5.20 5.19 5.18 5.17 5.16
1.63 1.62
1.59 H2O
1.28
H3C
NH
O
F
F
F
1.87 2.78 0.83 1.00 3.00
16 15 14 13 12 11 10 9 8 7 6 5 4 3 2 1 0 -1 -2 -3
f1 (ppm)

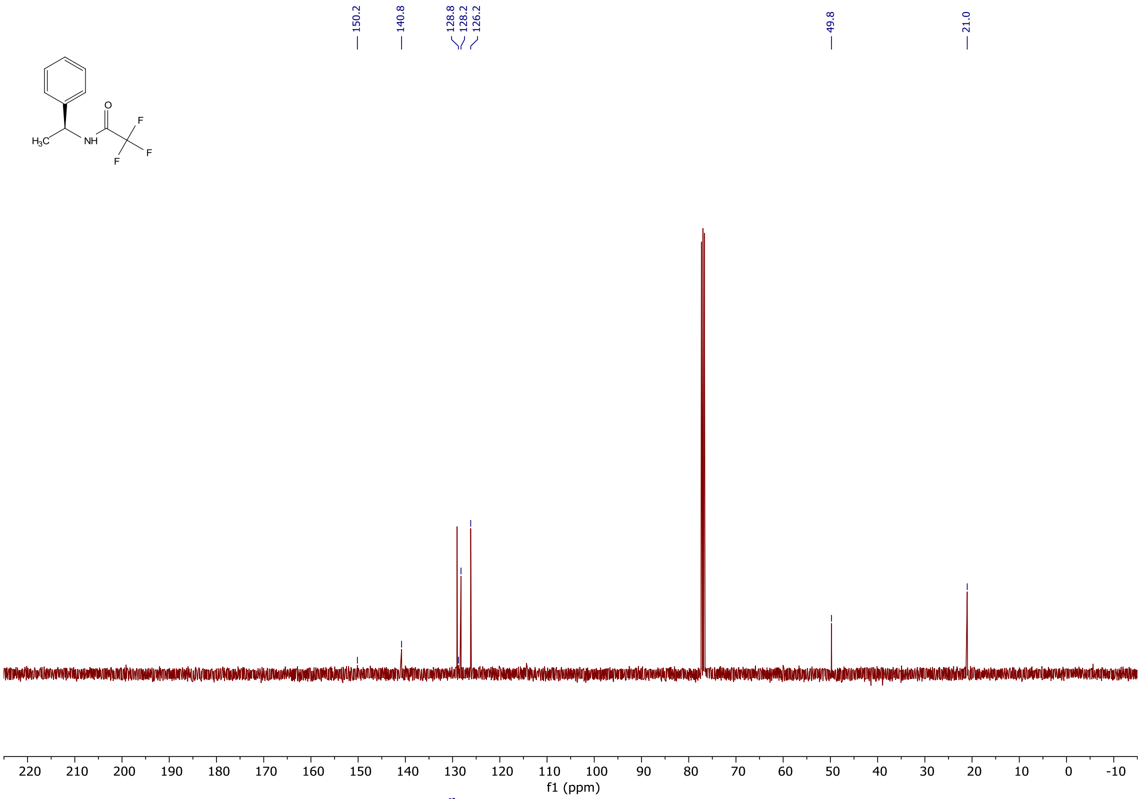
150.2
140.8
128.8
128.2
126.2
49.8
21.0
O
F
F
F
NH
H3C
220 210 200 190 180 170 160 150 140 130 120 110 100 90 80 70 60 50 40 30 20 10 0 -10
f1 (ppm)

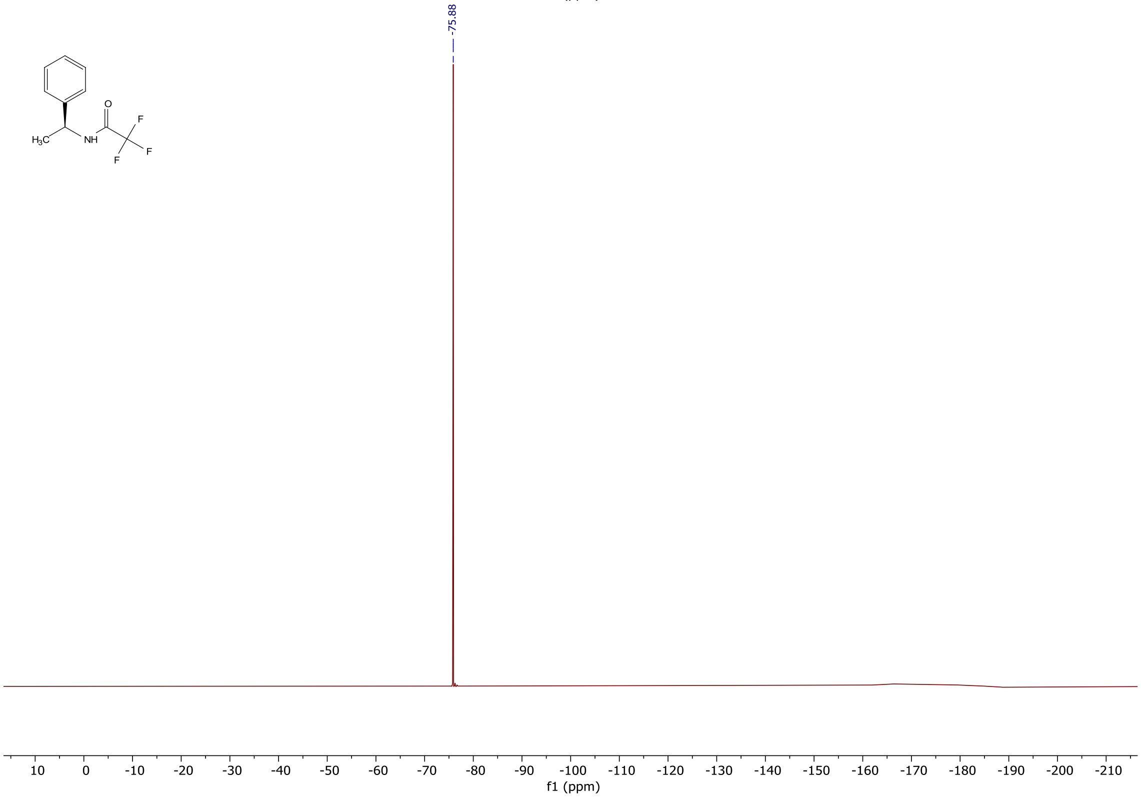
-75.88
O
F
F
F
NH
H3C
10 0 -10 -20 -30 -40 -50 -60 -70 -80 -90 -100 -110 -120 -130 -140 -150 -160 -170 -180 -190 -200 -210
f1 (ppm)

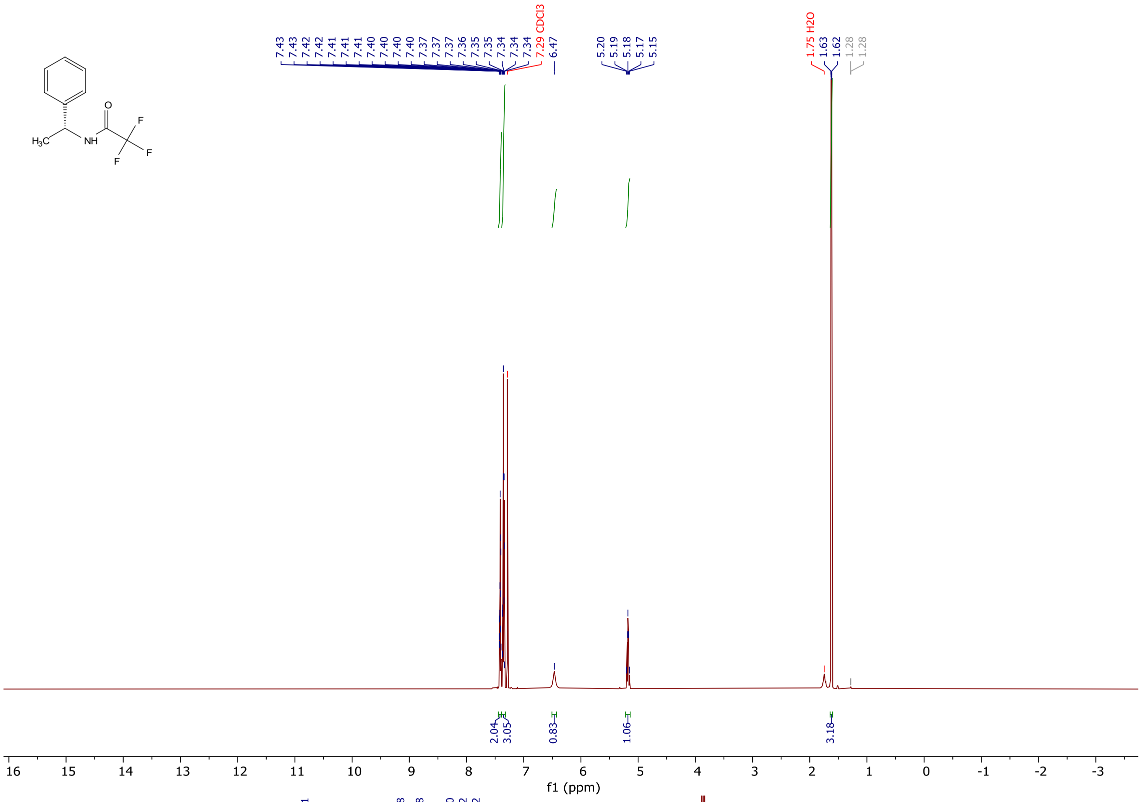
7.43
7.43
7.42
7.42
7.41
7.41
7.40
7.40
7.40
7.40
7.37
7.37
7.37
7.36
7.35
7.35
7.34
7.34
7.34
7.29 CDCl3
6.47
5.20
5.19
5.18
5.17
5.15
1.75 H2O
1.63
1.62
1.28
1.28
H3C
NH
O
F
F
F
2.04
3.05
0.83
1.06
3.18
f1 (ppm)

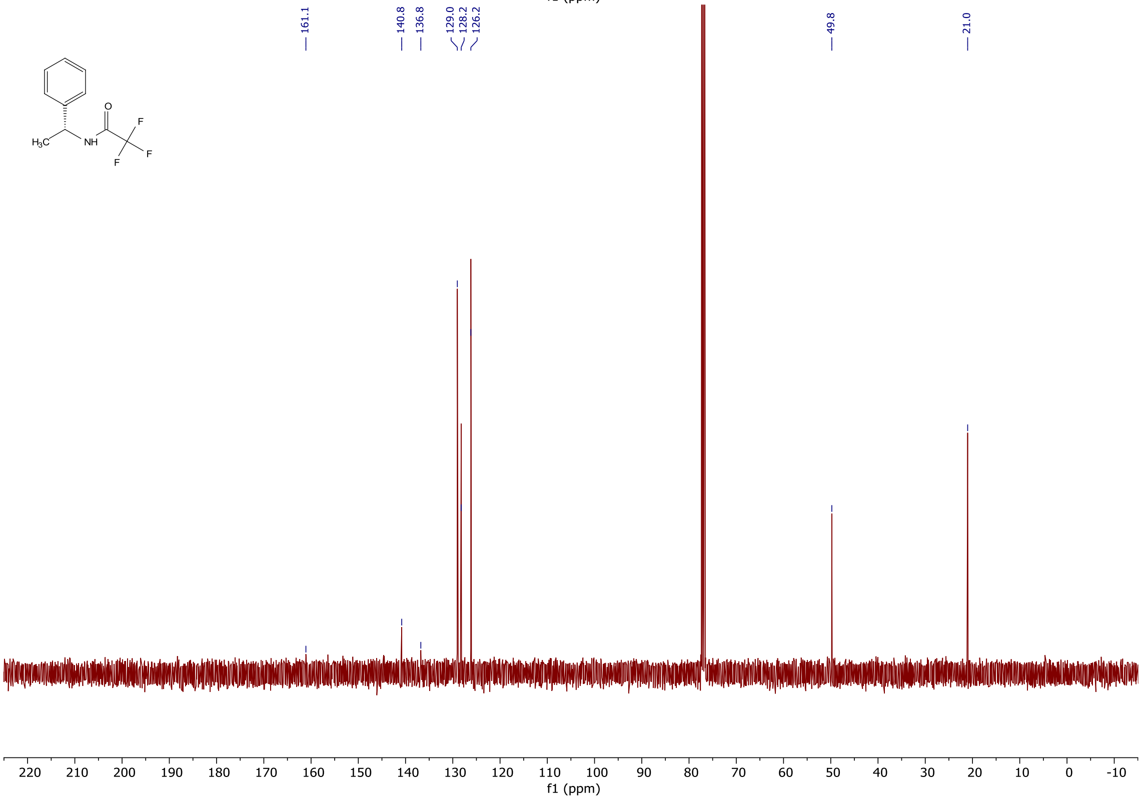
161.1
140.8
136.8
129.0
128.2
126.2
49.8
21.0
H3C
NH
O
F
F
F
f1 (ppm)

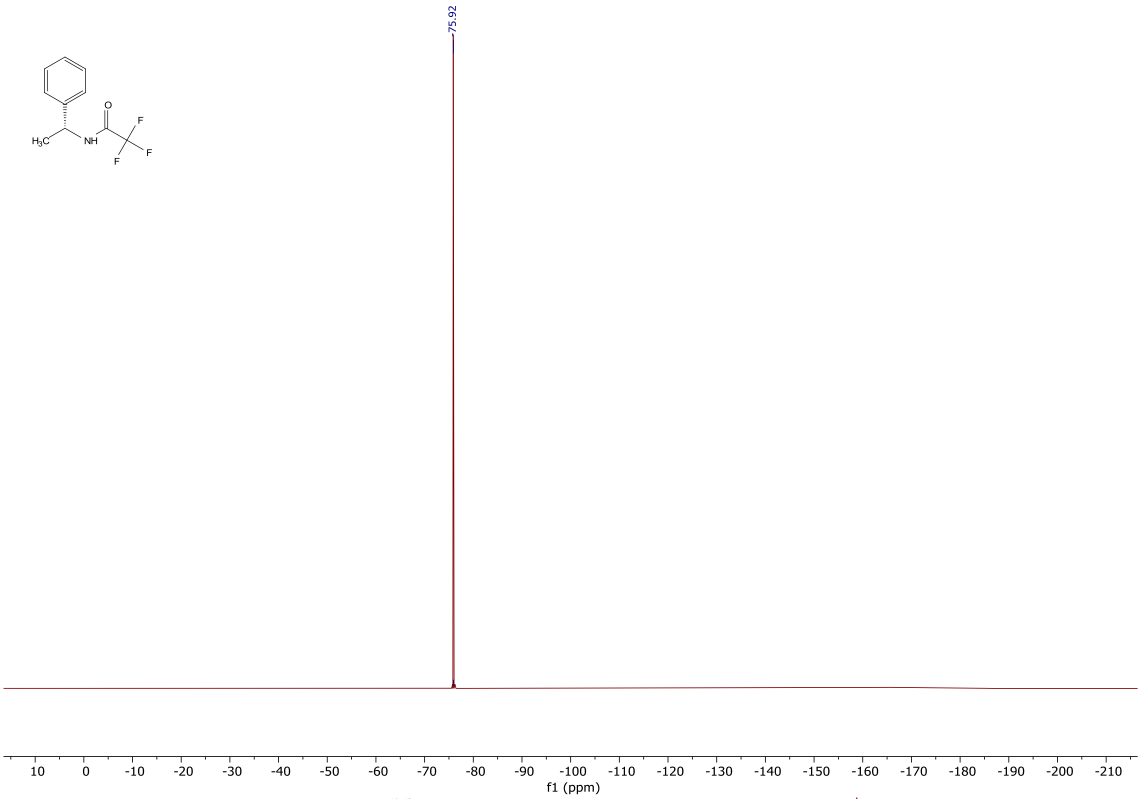

-75.92
H3C
NH
O
F
F
F
10 0 -10 -20 -30 -40 -50 -60 -70 -80 -90 -100 -110 -120 -130 -140 -150 -160 -170 -180 -190 -200 -210
f1 (ppm)

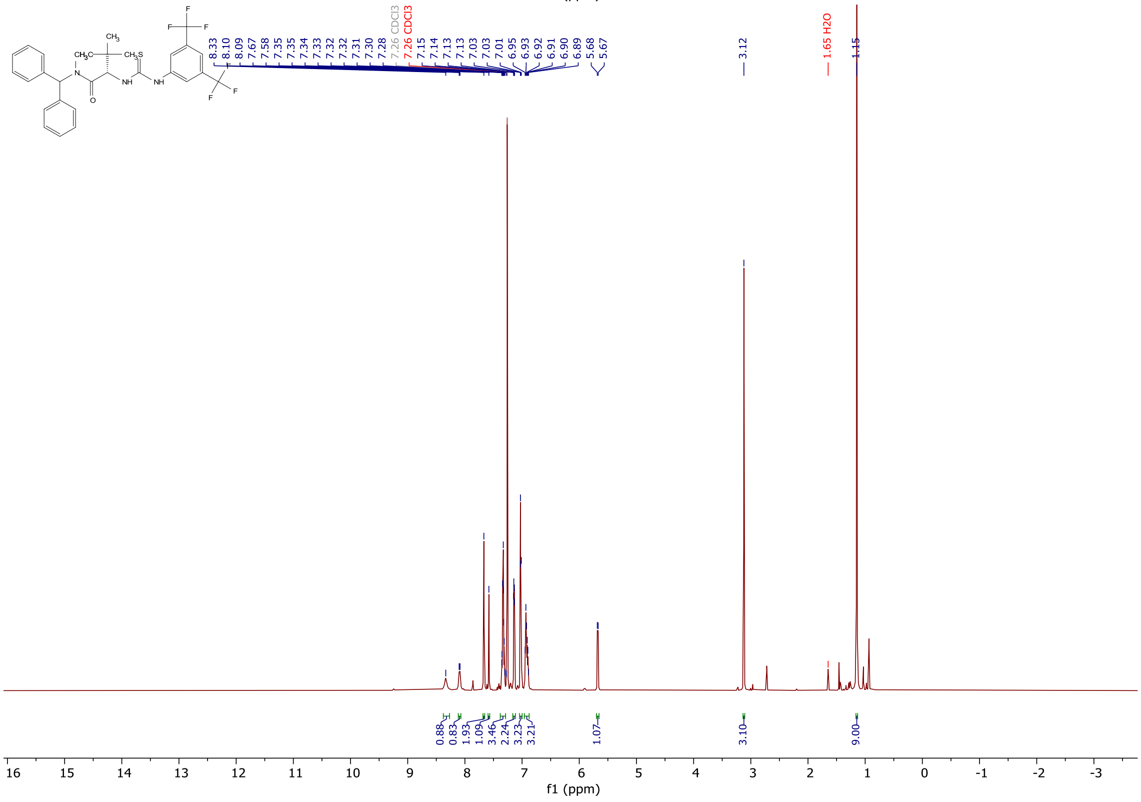

8.33 8.10 8.09 7.67 7.58 7.35 7.35 7.34 7.33 7.32 7.32 7.31 7.30 7.28
7.26 CDCl3
7.26 CDCl3
7.15 7.14 7.13 7.13 7.13 7.03 7.03 7.01 6.95 6.93 6.92 6.91 6.90 6.89 5.68 5.67
3.12
1.65 H2O
1.15
CH3
CH3
CH3
S
N
NH
NH
O
F
F
F
0.88 0.83 1.93 1.09 3.46 2.24 3.23 3.21 1.07 3.10 9.00
16 15 14 13 12 11 10 9 8 7 6 5 4 3 2 1 0 -1 -2 -3
f1 (ppm)

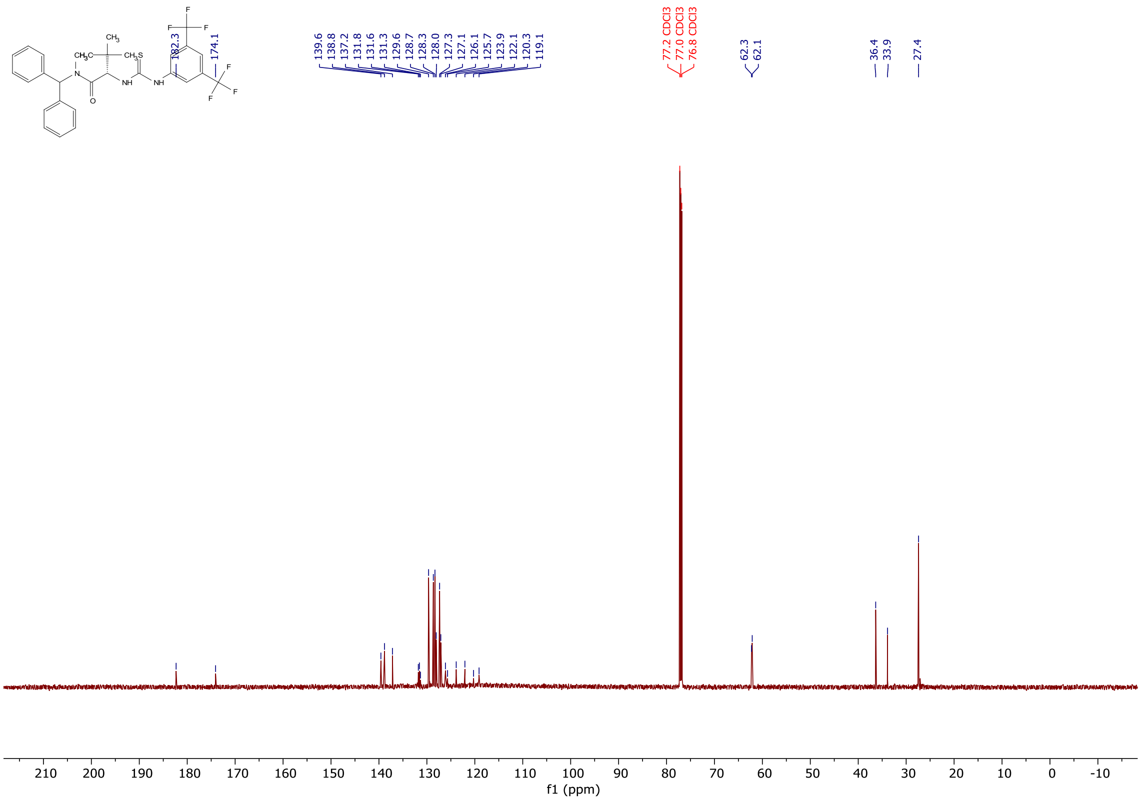
182.3
174.1
139.6
138.8
137.2
131.8
131.6
131.3
129.6
128.7
128.3
128.0
127.3
127.1
126.1
125.7
123.9
122.1
120.3
119.1
77.2 CDCl3
77.0 CDCl3
76.8 CDCl3
62.3
62.1
36.4
33.9
27.4
f1 (ppm)

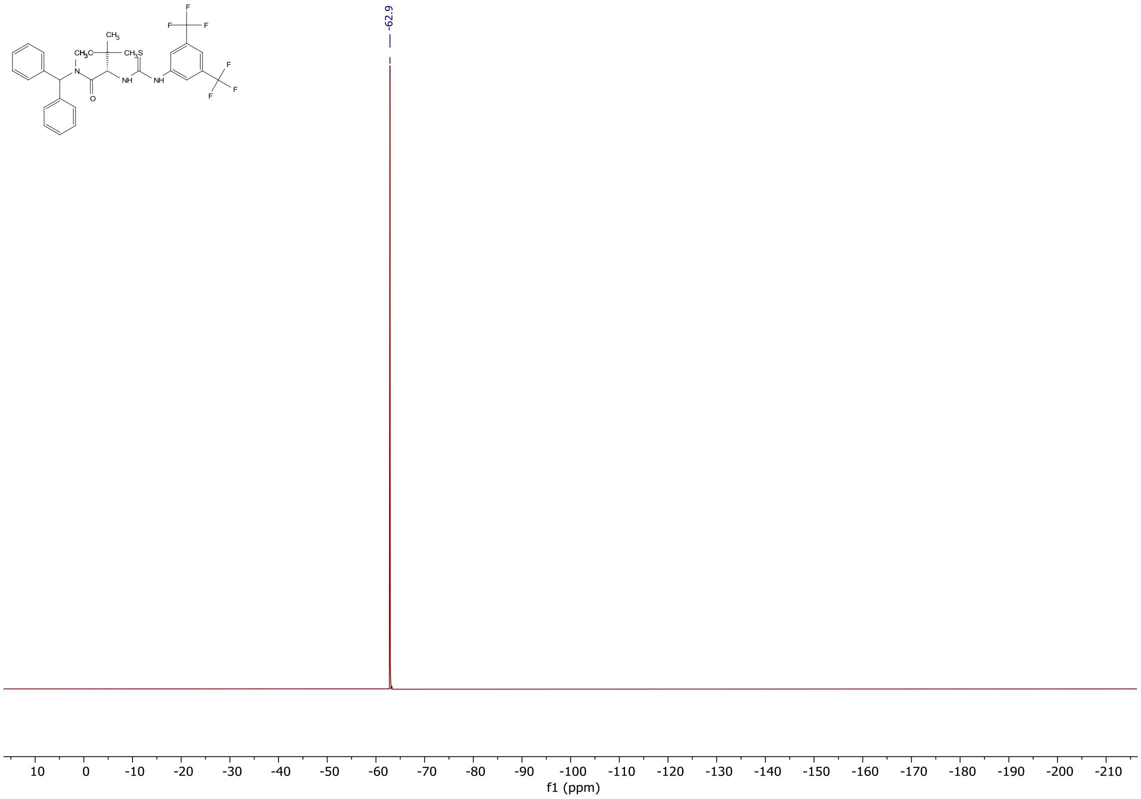
-62.9
f1 (ppm)

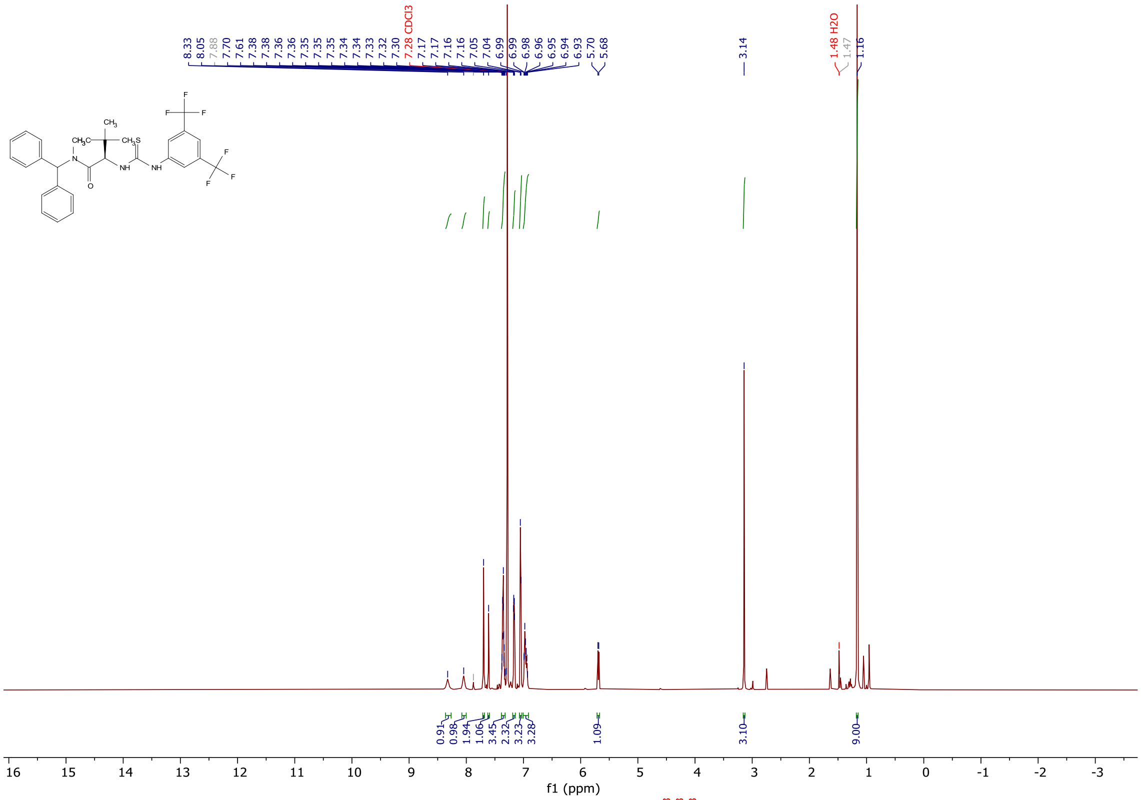

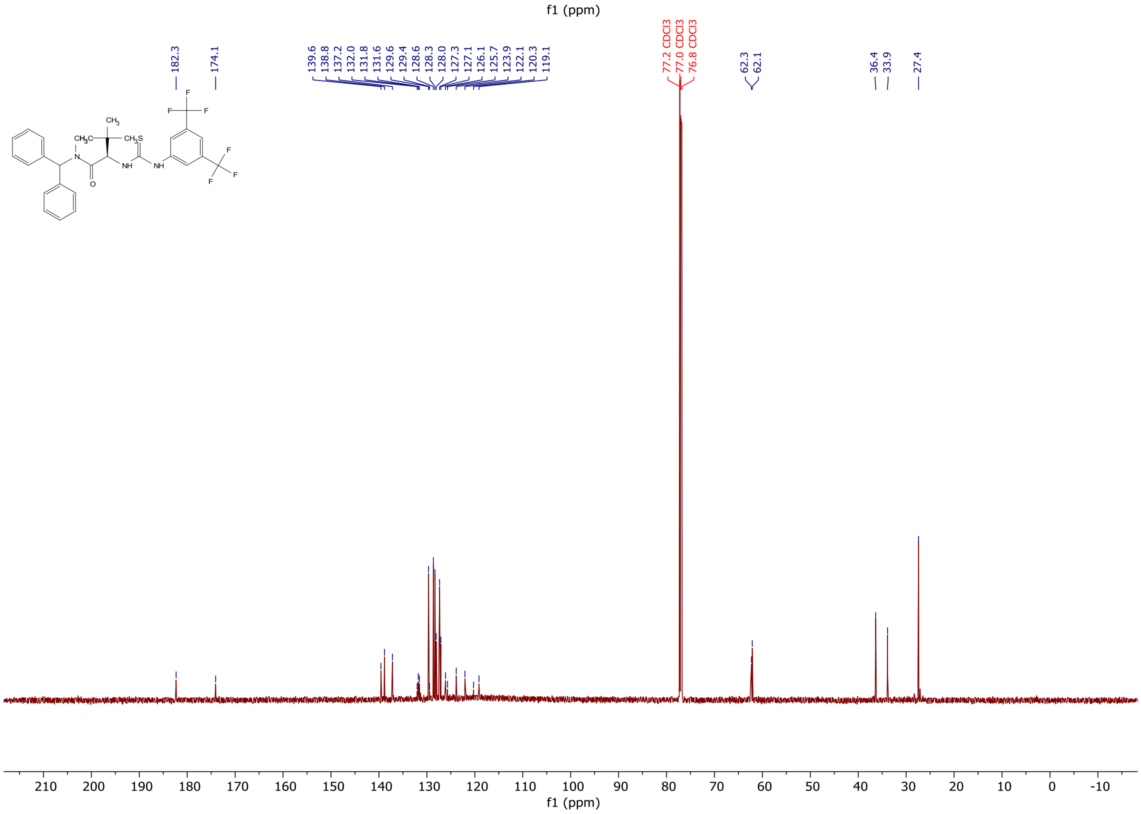

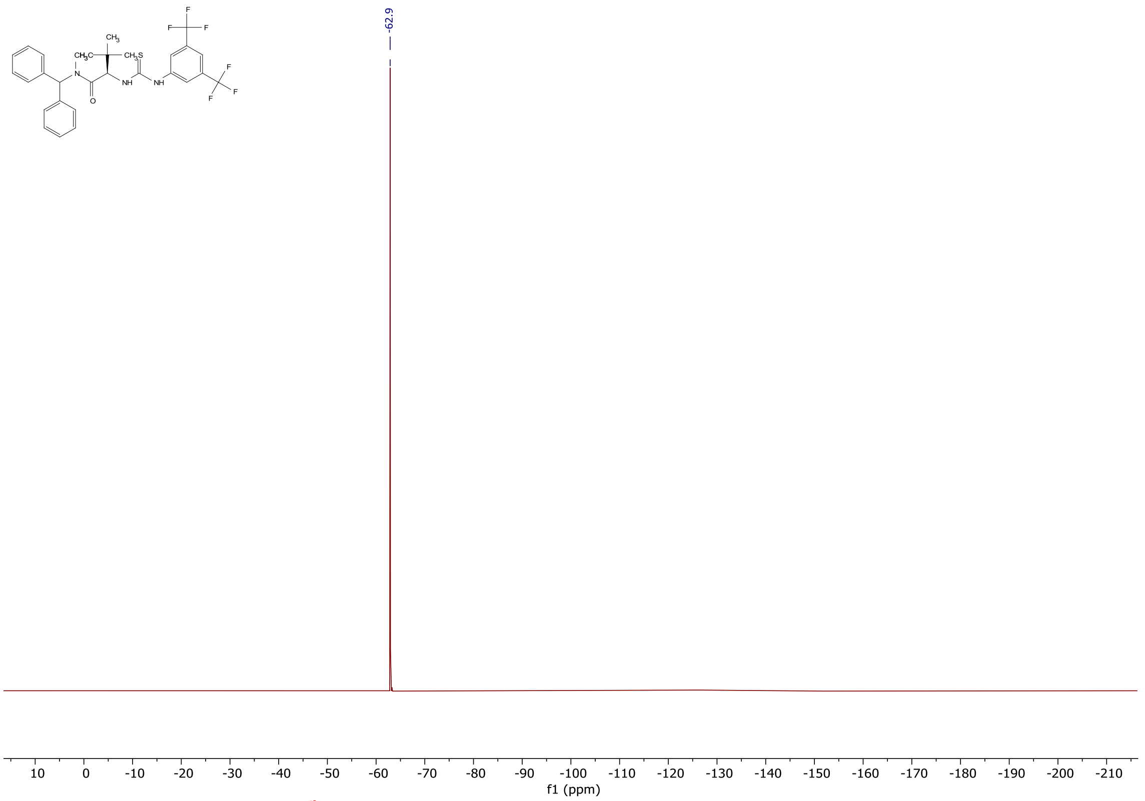

-62.9
10 0 -10 -20 -30 -40 -50 -60 -70 -80 -90 -100 -110 -120 -130 -140 -150 -160 -170 -180 -190 -200 -210
f1 (ppm)

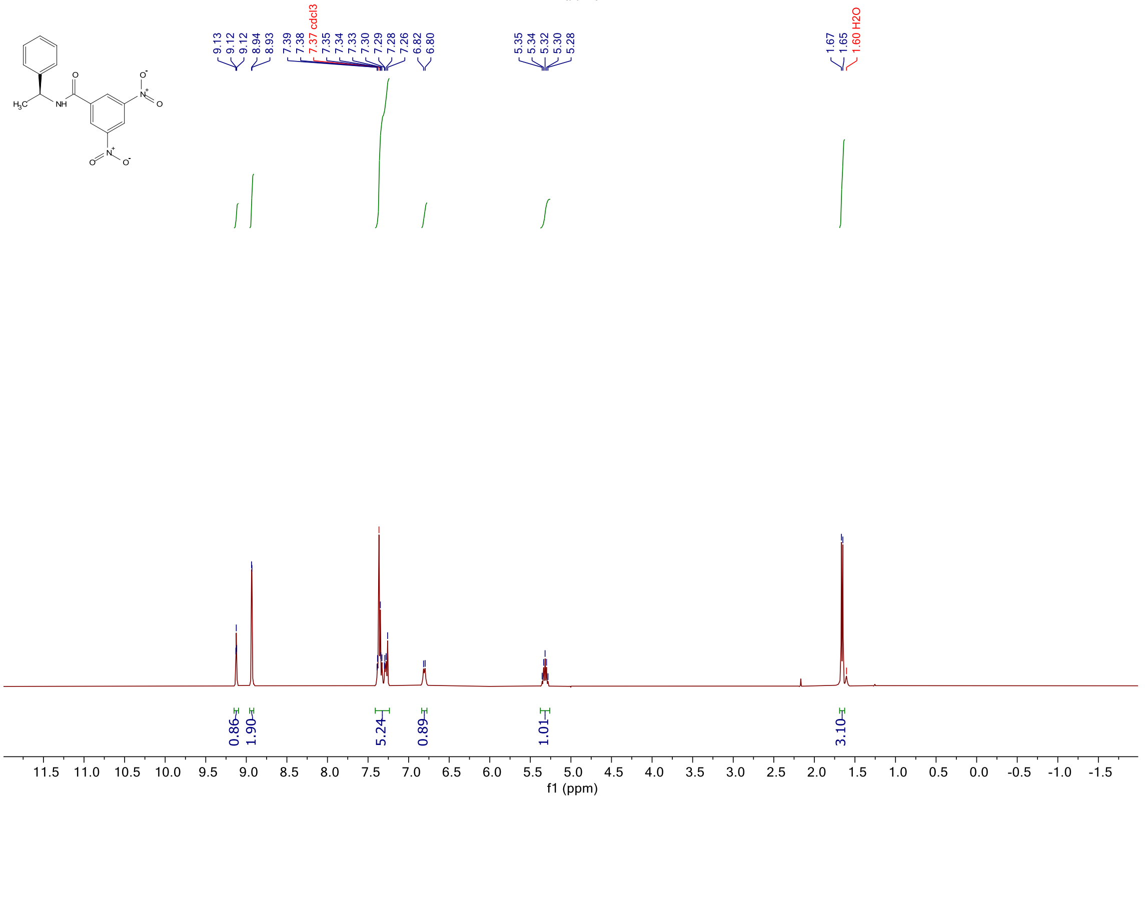

9.13 9.12 9.12 8.94 8.93 7.39 7.38 7.37 cdcl3 7.35 7.34 7.33 7.30 7.29 7.28 7.26 6.82 6.80
5.35 5.34 5.32 5.30 5.28
1.67 1.65 1.60 H2O
0.86 1.90 5.24 0.89 1.01 3.10
11.5 11.0 10.5 10.0 9.5 9.0 8.5 8.0 7.5 7.0 6.5 6.0 5.5 5.0 4.5 4.0 3.5 3.0 2.5 2.0 1.5 1.0 0.5 0.0 -0.5 -1.0 -1.5
f1 (ppm)

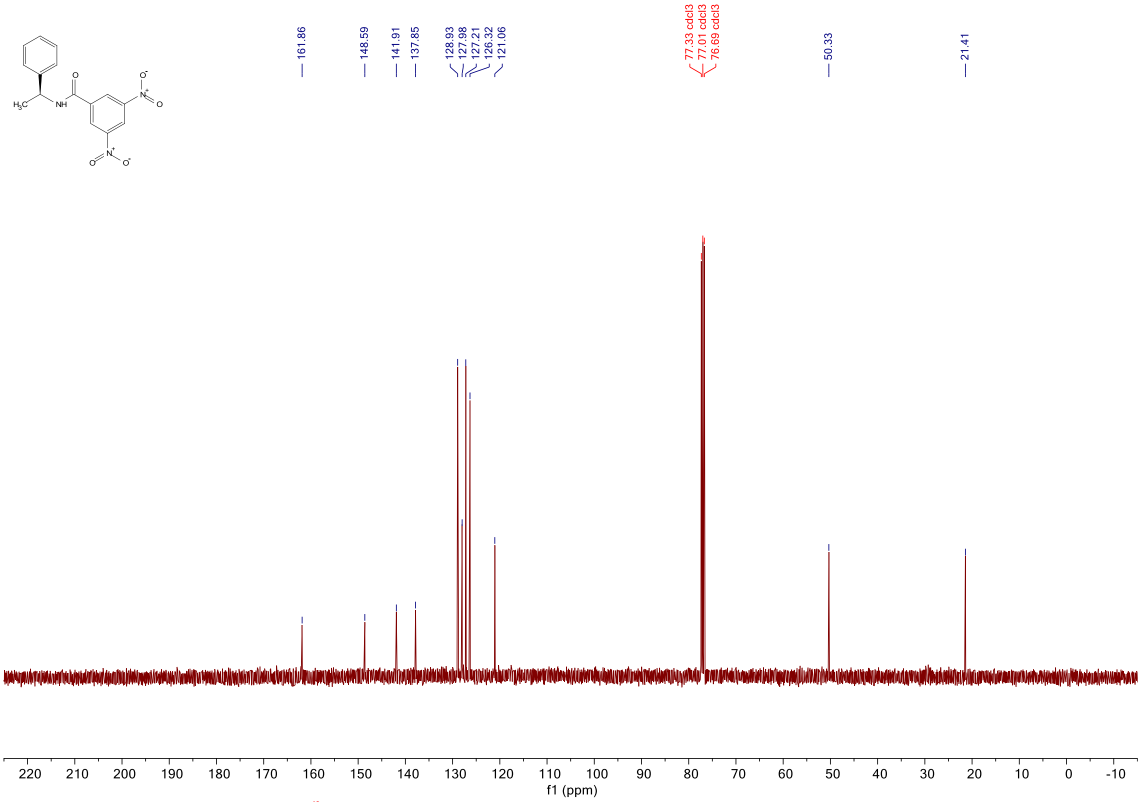
H3C
NH
O
N+
O-
O
161.86
148.59
141.91
137.85
128.93
127.98
127.21
126.32
121.06
77.33 cdcl3
77.01 cdcl3
76.69 cdcl3
50.33
21.41
220 210 200 190 180 170 160 150 140 130 120 110 100 90 80 70 60 50 40 30 20 10 0 -10
f1 (ppm)

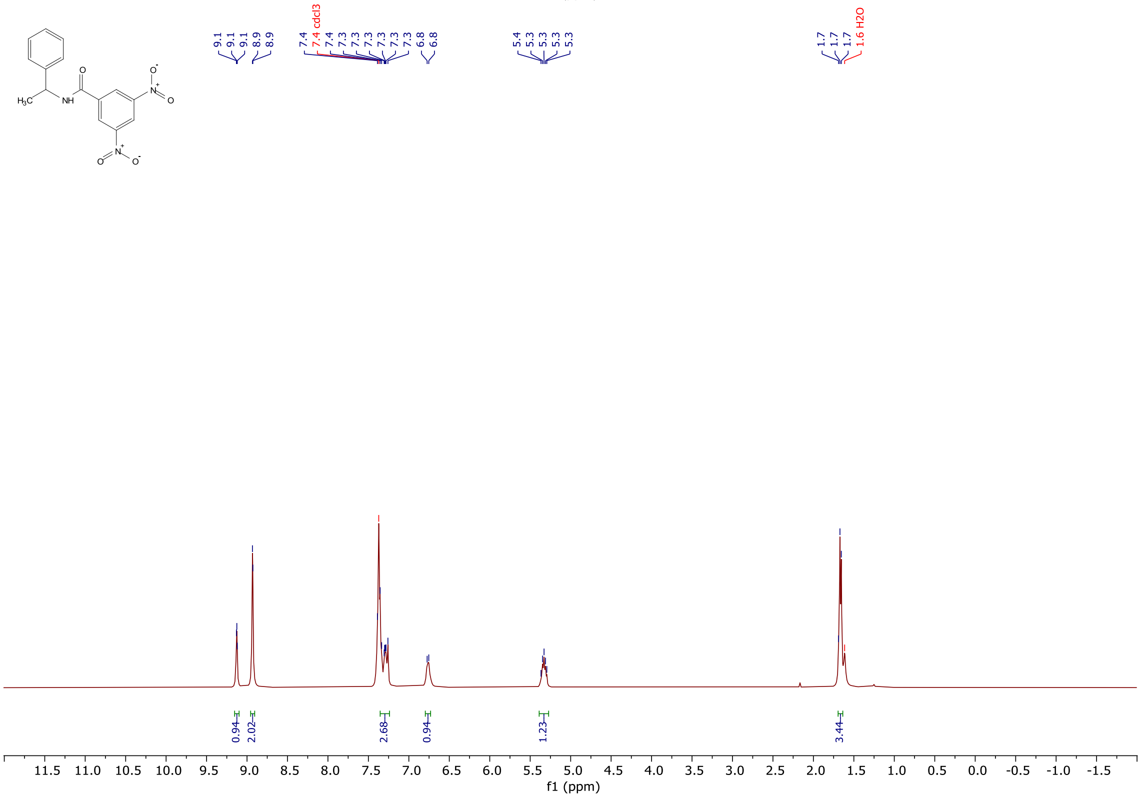
H3C
NH
O
N+
O-
O
9.1
9.1
9.1
8.9
8.9
7.4
7.4 cdcl3
7.4
7.3
7.3
7.3
7.3
7.3
7.3
6.8
6.8
5.4
5.3
5.3
5.3
5.3
1.7
1.7
1.7
1.6 H2O
0.94
2.02
2.68
0.94
1.23
3.44
11.5 11.0 10.5 10.0 9.5 9.0 8.5 8.0 7.5 7.0 6.5 6.0 5.5 5.0 4.5 4.0 3.5 3.0 2.5 2.0 1.5 1.0 0.5 0.0 -0.5 -1.0 -1.5
f1 (ppm)

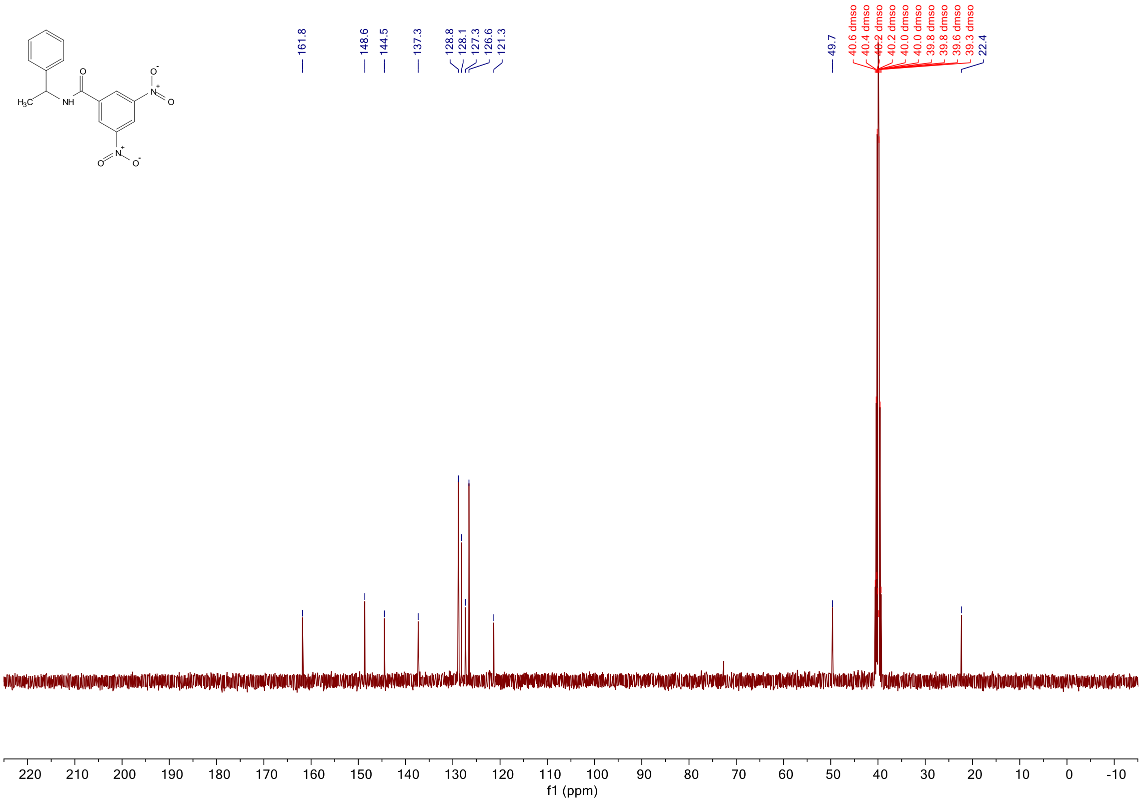
H$_3$C
NH
O
161.8
148.6
144.5
137.3
128.8
128.1
127.3
126.6
121.3
49.7
40.6 dmso
40.4 dmso
40.2 dmso
40.2 dmso
40.0 dmso
40.0 dmso
39.8 dmso
39.8 dmso
39.6 dmso
39.3 dmso
22.4
220 210 200 190 180 170 160 150 140 130 120 110 100 90 80 70 60 50 40 30 20 10 0 -10
f1 (ppm)

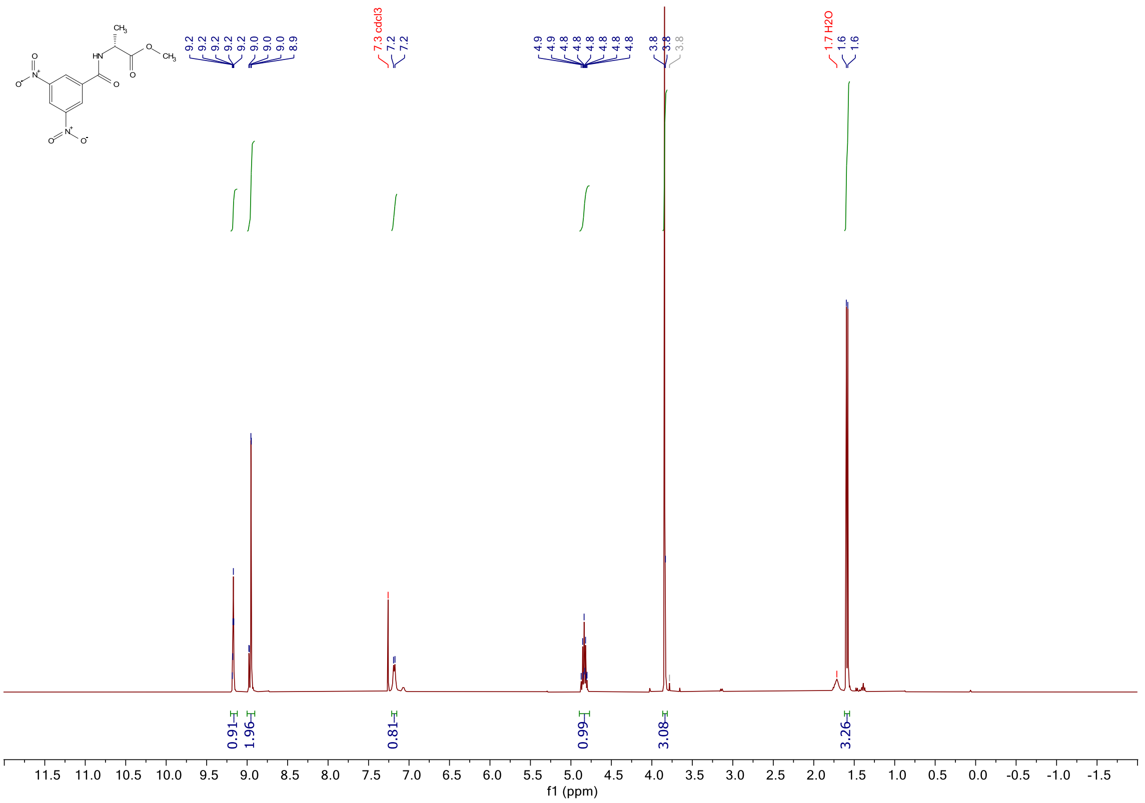
CH$_3$
HN
O
9.2
9.2
9.2
9.2
9.2
9.0
9.0
9.0
8.9
7.3 cdcl3
7.2
7.2
4.9
4.9
4.8
4.8
4.8
4.8
4.8
4.8
3.8
3.8
3.8
1.7 H2O
1.6
1.6
0.91
1.96
0.81
0.99
3.08
3.26
11.5 11.0 10.5 10.0 9.5 9.0 8.5 8.0 7.5 7.0 6.5 6.0 5.5 5.0 4.5 4.0 3.5 3.0 2.5 2.0 1.5 1.0 0.5 0.0 -0.5 -1.0 -1.5
f1 (ppm)

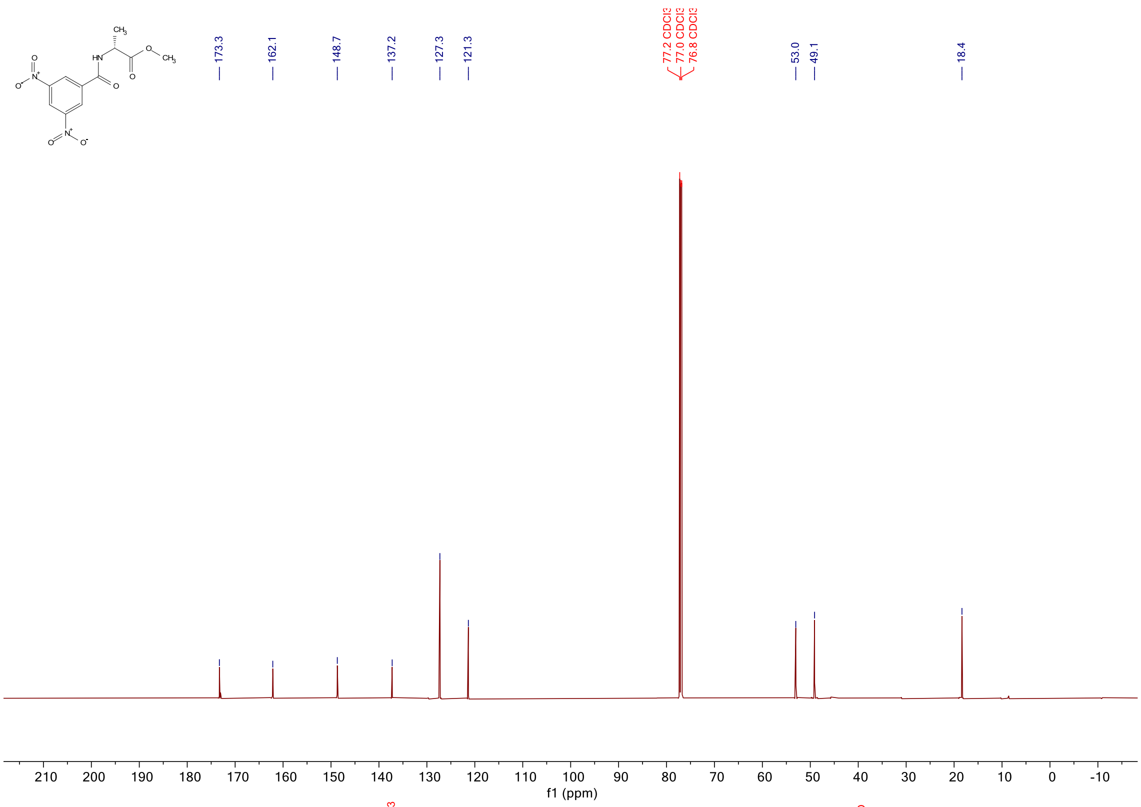

173.3
162.1
148.7
137.2
127.3
121.3
77.2 CDCl3
77.0 CDCl3
76.8 CDCl3
53.0
49.1
18.4
f1 (ppm)

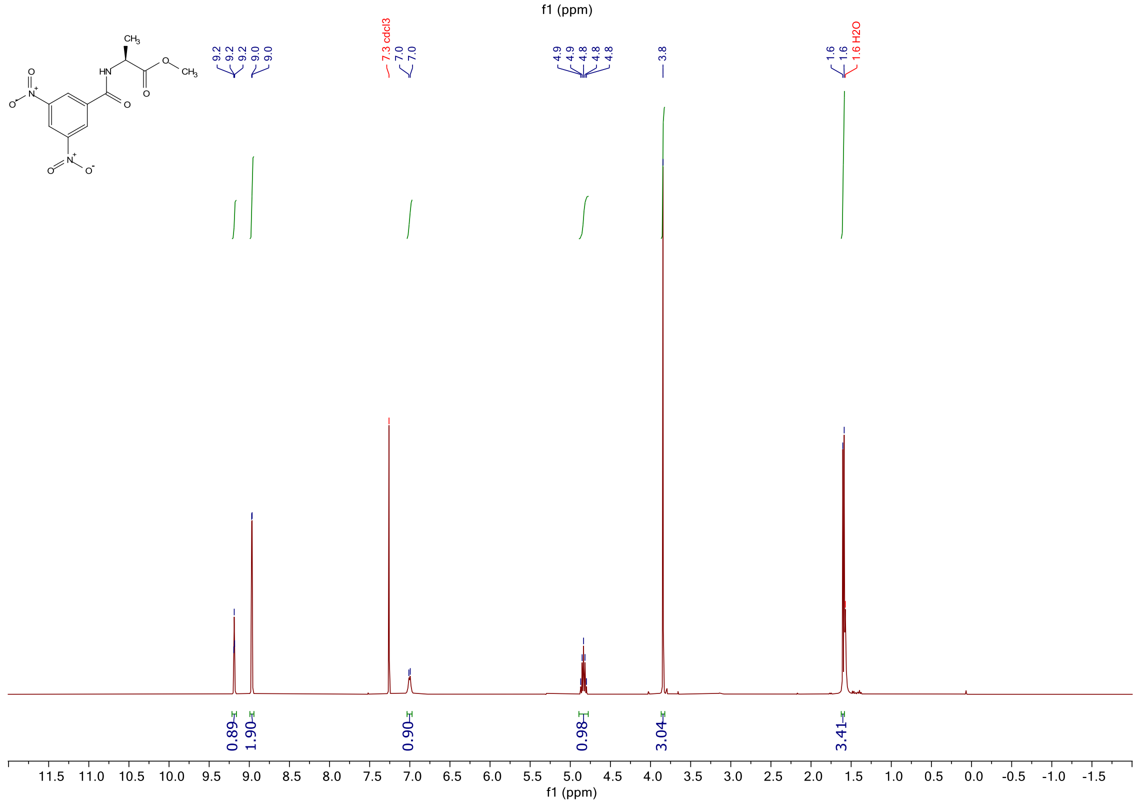

9.2
9.2
9.2
9.0
9.0
7.3 cdcl3
7.0
7.0
4.9
4.9
4.8
4.8
4.8
3.8
1.6
1.6
1.6 H2O
0.89
1.90
0.90
0.98
3.04
3.41
f1 (ppm)

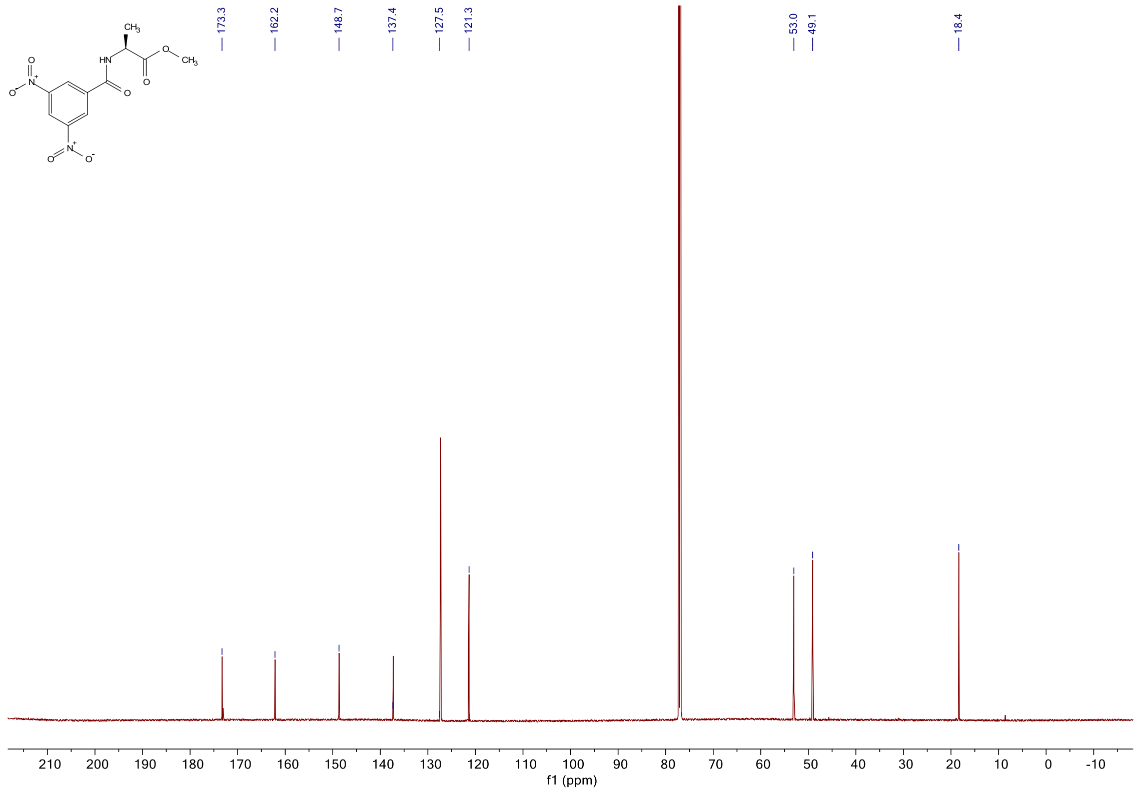
173.3
162.2
148.7
137.4
127.5
121.3
53.0
49.1
18.4
f1 (ppm)

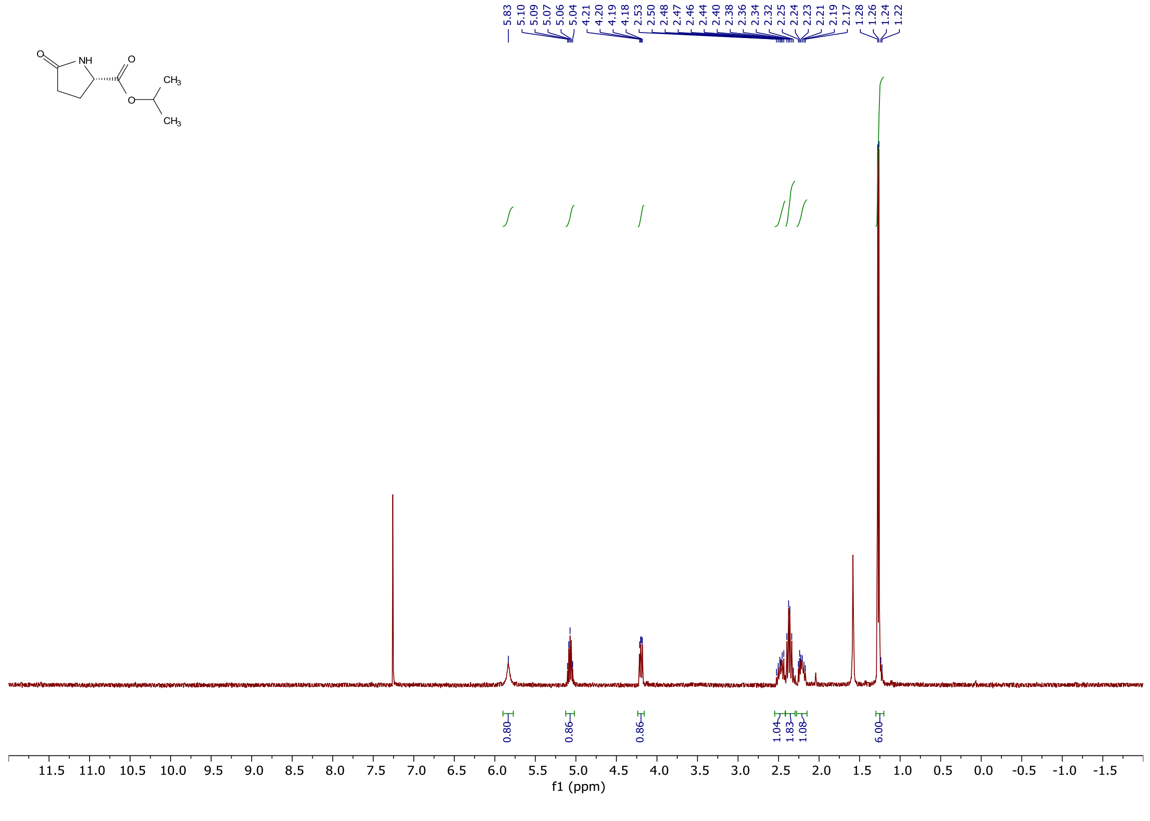
5.83
5.10
5.09
5.07
5.06
5.04
4.21
4.20
4.19
4.18
2.53
2.50
2.48
2.47
2.46
2.44
2.40
2.38
2.36
2.34
2.32
2.25
2.24
2.23
2.21
2.19
2.17
1.28
1.26
1.24
1.22
0.80
0.86
0.86
1.04
1.83
1.08
6.00
f1 (ppm)

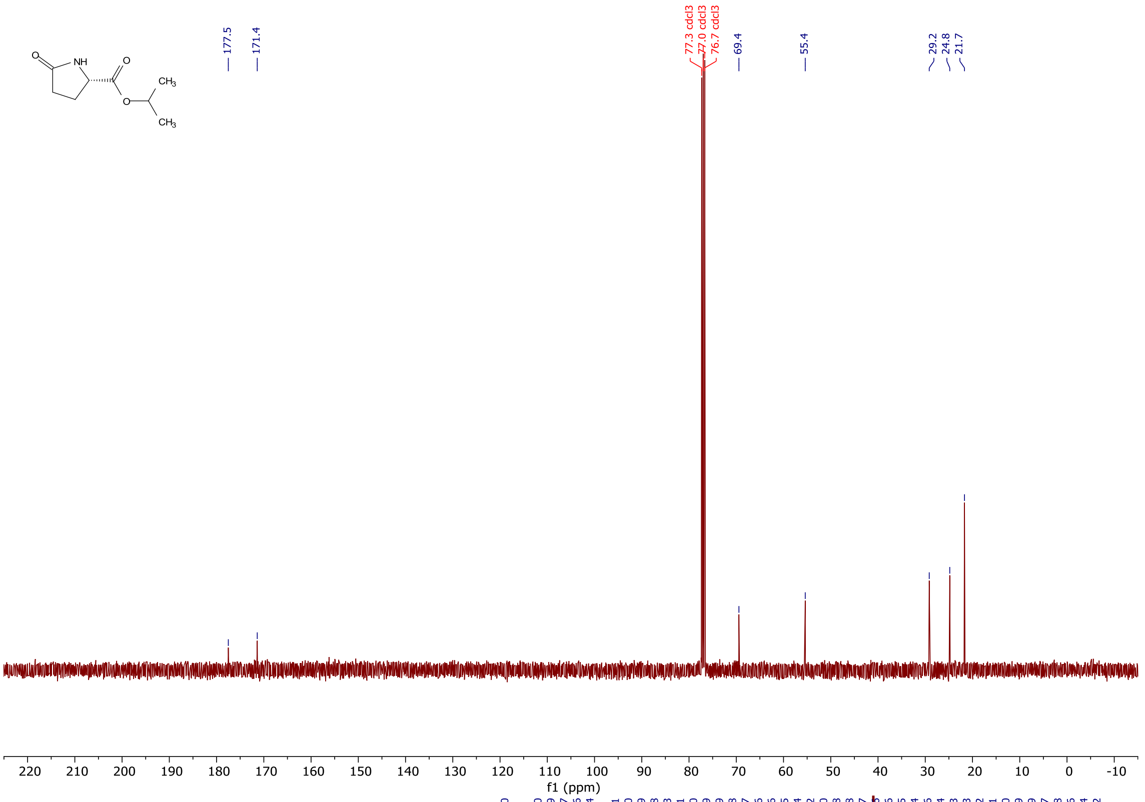
NH
O
O
CH3
O
CH3
177.5
171.4
77.3 cdcl3
77.0 cdcl3
76.7 cdcl3
69.4
55.4
29.2
24.8
21.7
220 210 200 190 180 170 160 150 140 130 120 110 100 90 80 70 60 50 40 30 20 10 0 -10
f1 (ppm)

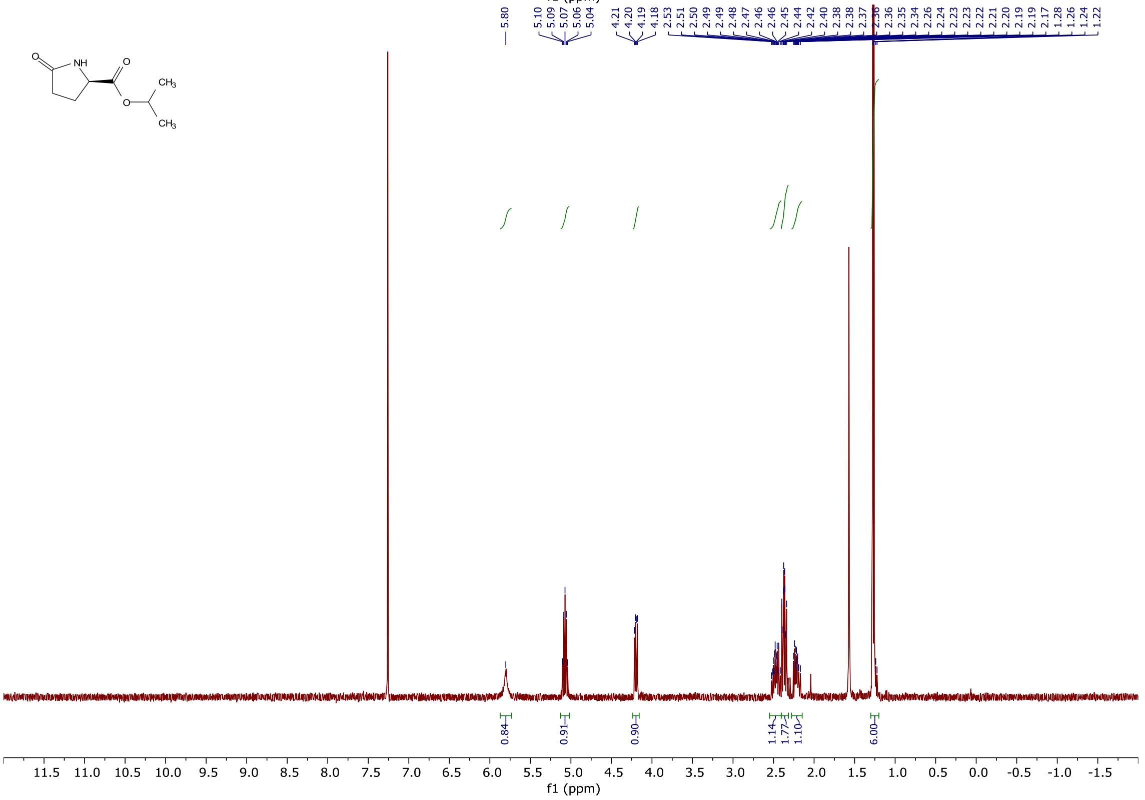
NH
O
O
CH3
O
CH3
5.80
5.10
5.09
5.07
5.06
5.04
4.21
4.20
4.19
4.18
2.53
2.51
2.50
2.49
2.49
2.48
2.47
2.46
2.46
2.45
2.44
2.42
2.40
2.38
2.38
2.37
2.36
2.36
2.35
2.34
2.26
2.24
2.24
2.23
2.23
2.22
2.21
2.20
2.19
2.19
2.17
1.28
1.26
1.24
1.22
0.84
0.91
0.90
1.14
1.77
1.10
6.00
11.5 11.0 10.5 10.0 9.5 9.0 8.5 8.0 7.5 7.0 6.5 6.0 5.5 5.0 4.5 4.0 3.5 3.0 2.5 2.0 1.5 1.0 0.5 0.0 -0.5 -1.0 -1.5
f1 (ppm)

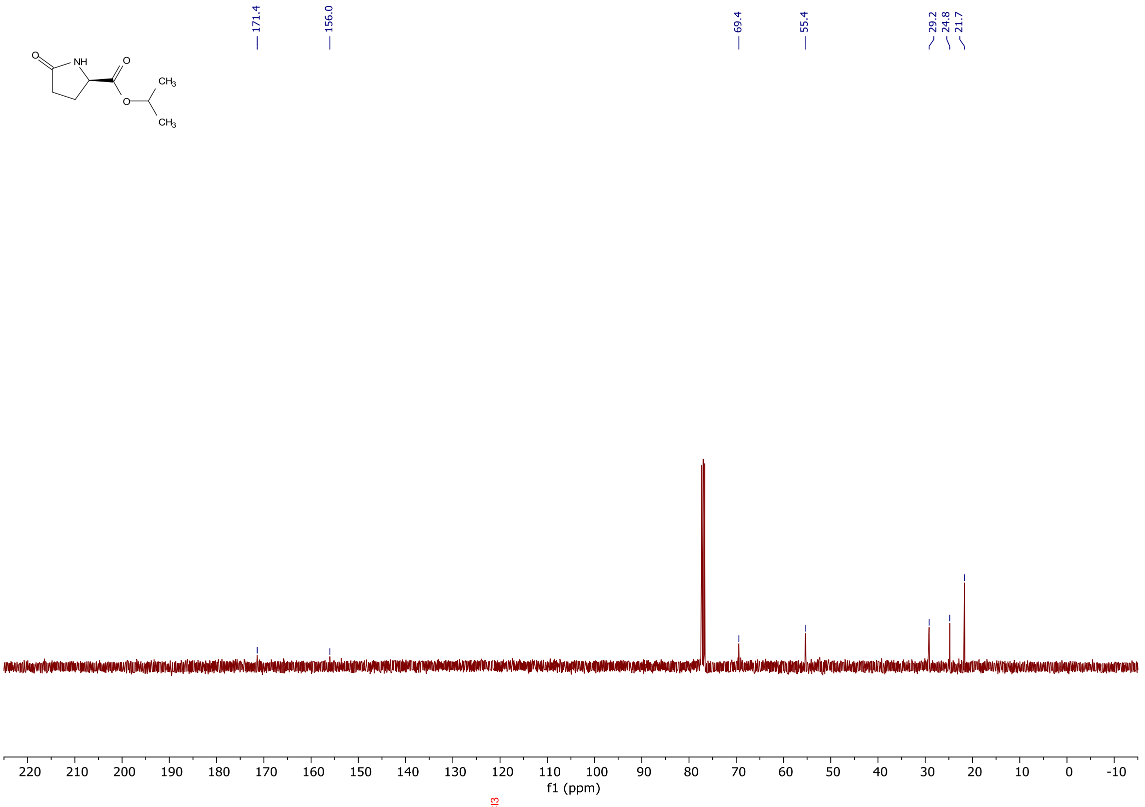
O
NH
O
CH3
O
CH3
171.4
156.0
69.4
55.4
29.2
24.8
21.7
220 210 200 190 180 170 160 150 140 130 120 110 100 90 80 70 60 50 40 30 20 10 0 -10
f1 (ppm)

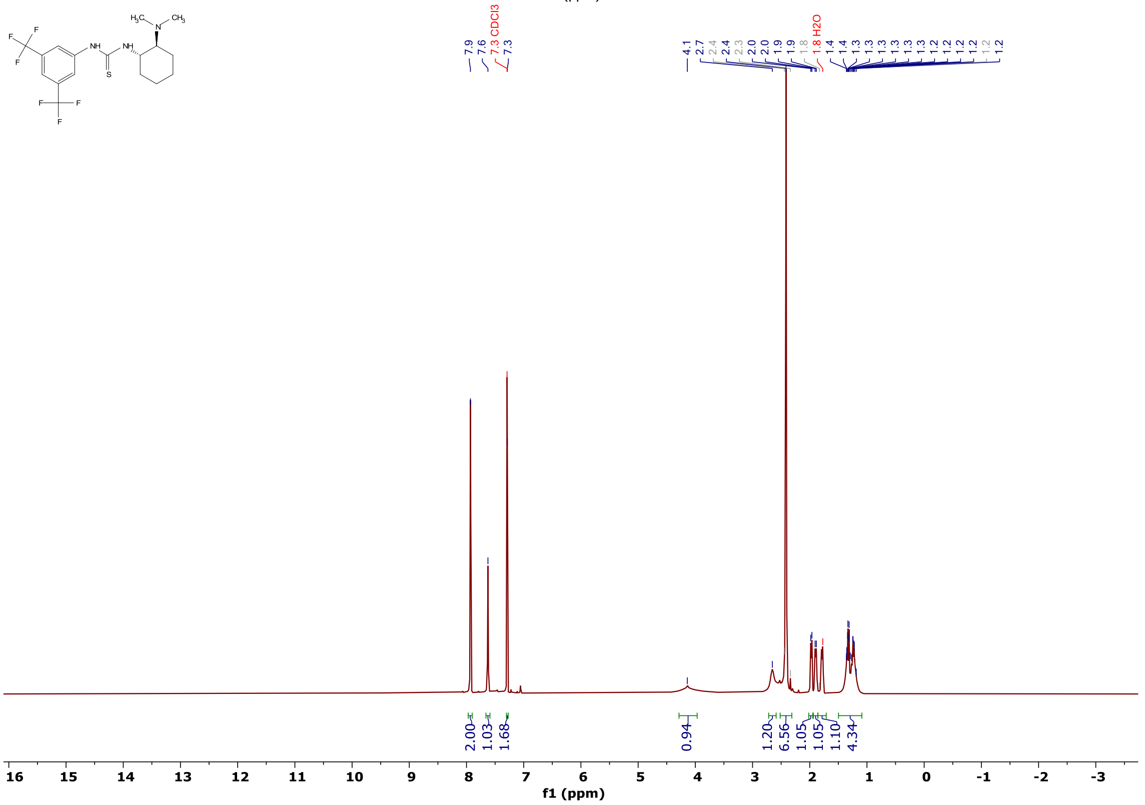
H3C
N
CH3
F
F
F
NH
NH
S
F
F
F
7.9
7.6
7.3 CDCl3
7.3
4.1
2.7
2.4
2.4
2.3
2.0
2.0
1.9
1.9
1.8
1.8 H2O
1.4
1.4
1.3
1.3
1.3
1.3
1.3
1.3
1.2
1.2
1.2
1.2
1.2
1.2
2.00
1.03
1.68
0.94
1.20
6.56
1.05
1.05
1.10
4.34
16 15 14 13 12 11 10 9 8 7 6 5 4 3 2 1 0 -1 -2 -3
f1 (ppm)

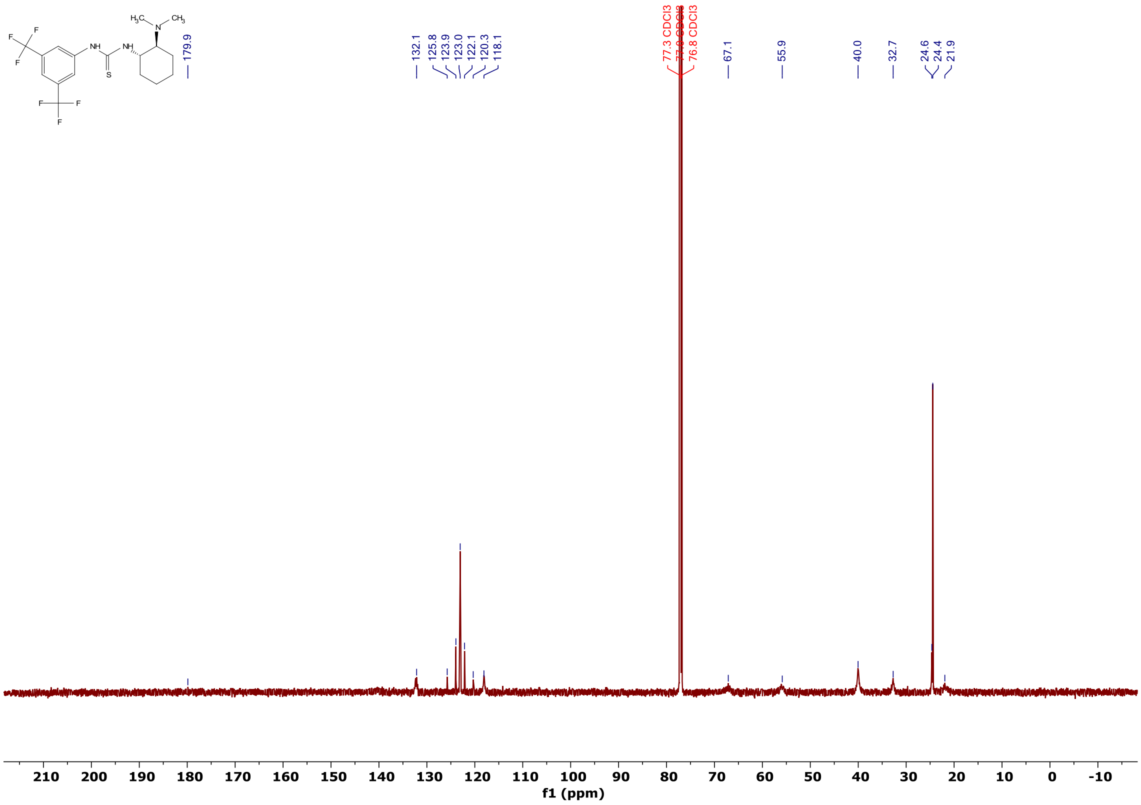
179.9
132.1
125.8
123.9
123.0
122.1
120.3
118.1
77.3 CDCl3
76.8 CDCl3
67.1
55.9
40.0
32.7
24.6
24.4
21.9
f1 (ppm)

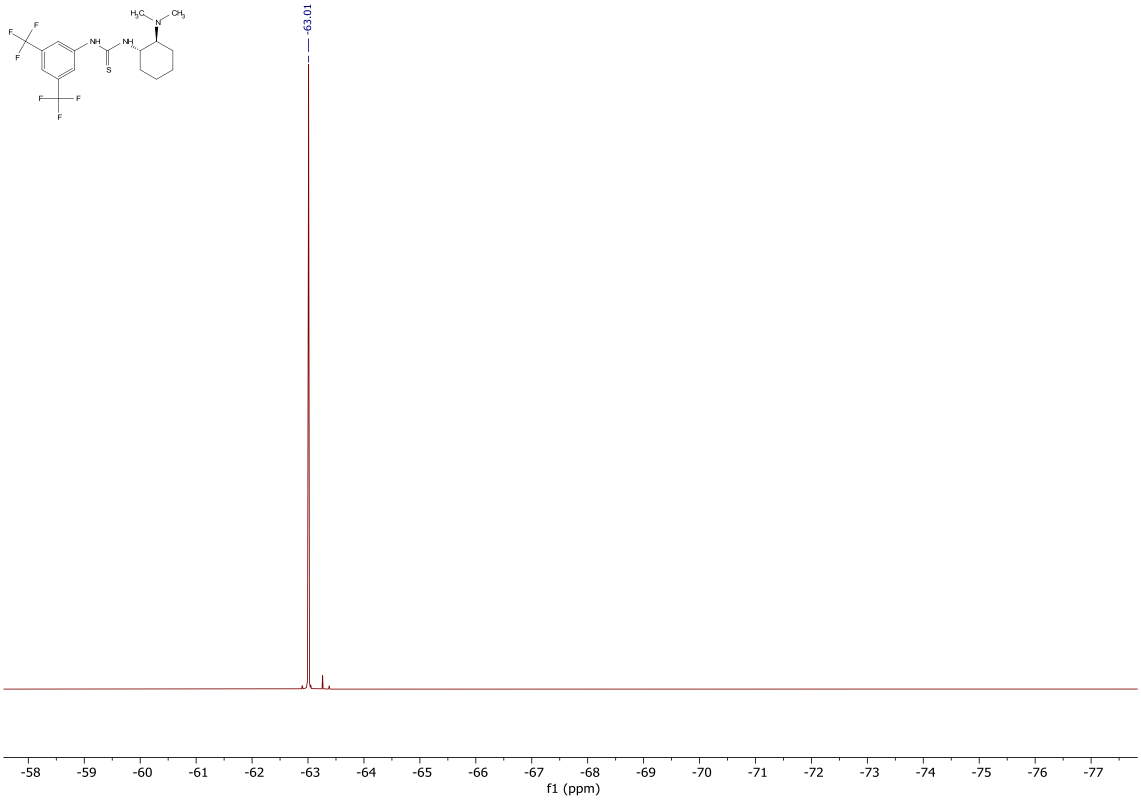
-63.01
f1 (ppm)

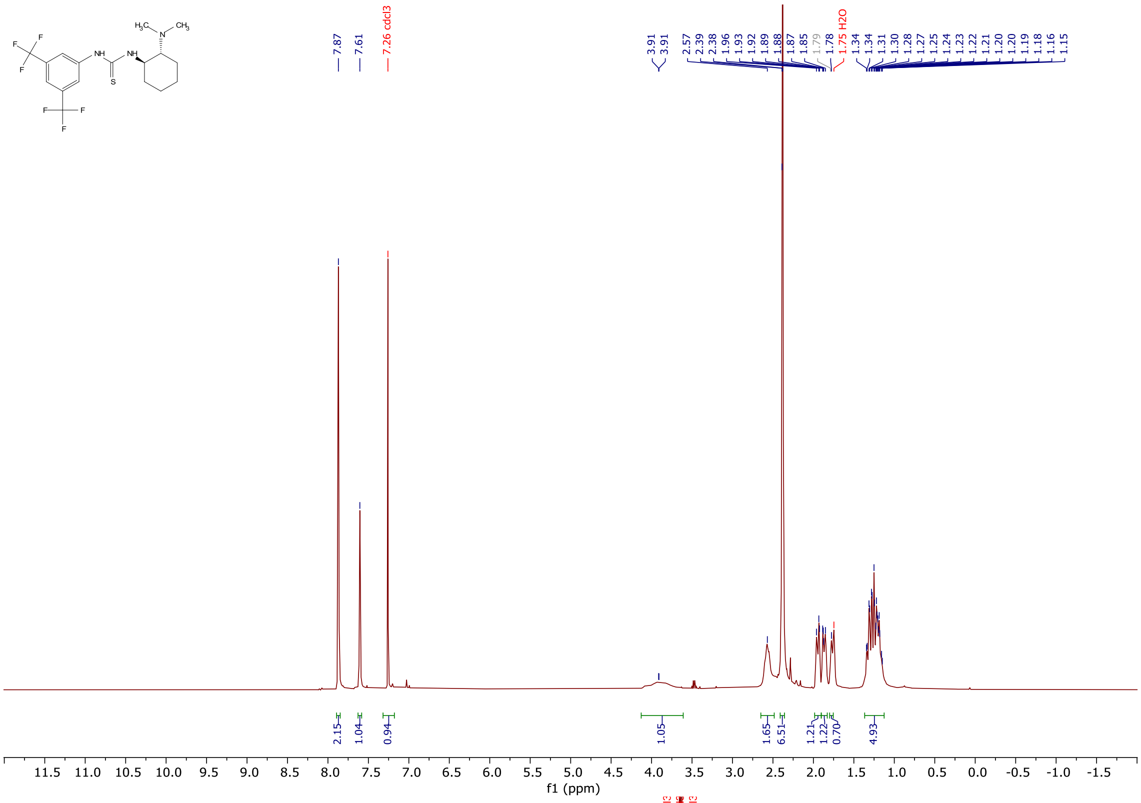
7.87
7.61
7.26 cdcl3
3.91
3.91
2.57
2.39
2.38
1.96
1.93
1.92
1.89
1.88
1.87
1.85
1.79
1.78
1.75 H2O
1.34
1.34
1.31
1.30
1.28
1.27
1.25
1.24
1.23
1.22
1.21
1.20
1.20
1.19
1.18
1.16
1.15
2.15
1.04
0.94
1.05
1.65
6.51
1.21
1.22
0.70
4.93
f1 (ppm)

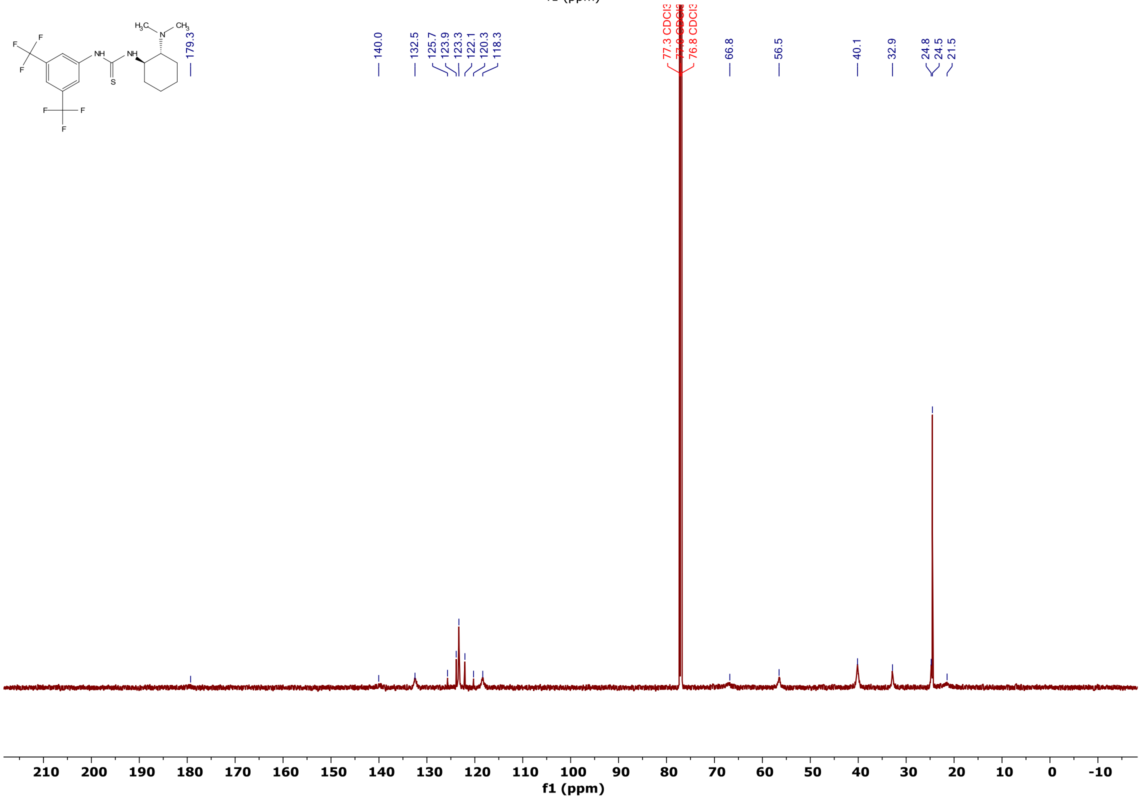
179.3
140.0
132.5
125.7
123.9
123.3
122.1
120.3
118.3
77.3 CDCl3
76.8 CDCl3
66.8
56.5
40.1
32.9
24.8
24.5
21.5
f1 (ppm)

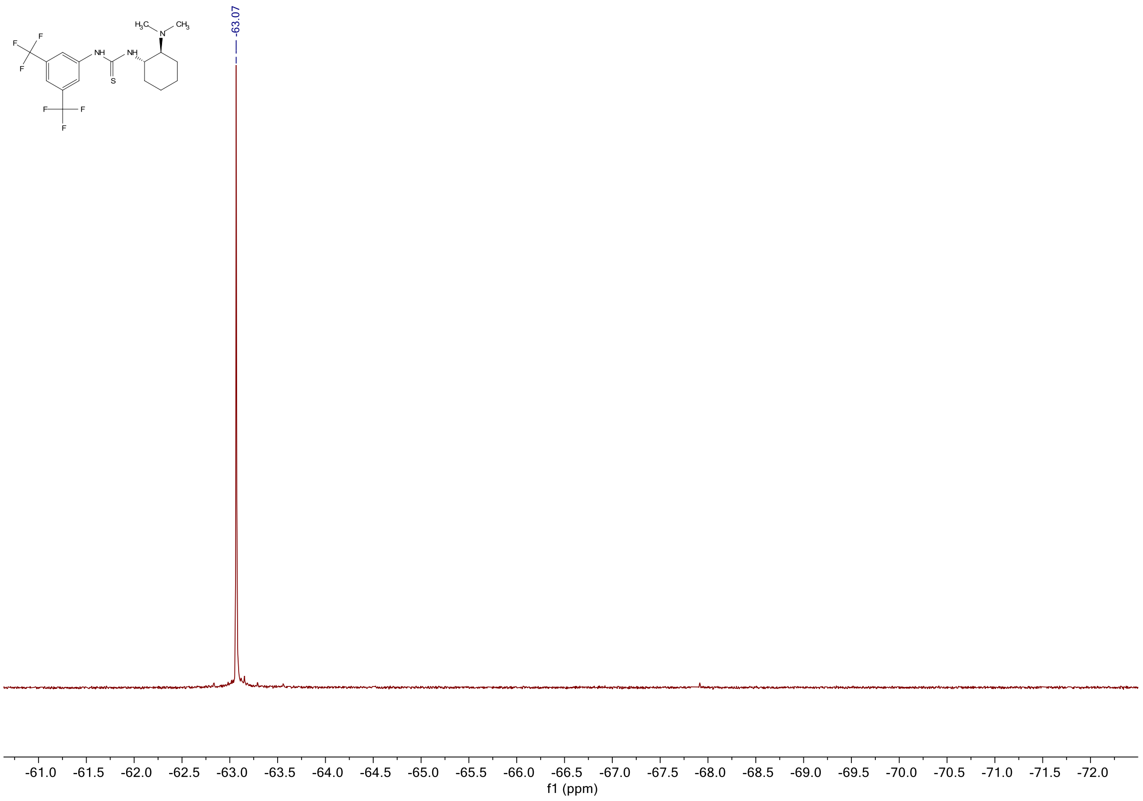
-63.07
-61.0 -61.5 -62.0 -62.5 -63.0 -63.5 -64.0 -64.5 -65.0 -65.5 -66.0 -66.5 -67.0 -67.5 -68.0 -68.5 -69.0 -69.5 -70.0 -70.5 -71.0 -71.5 -72.0
f1 (ppm)

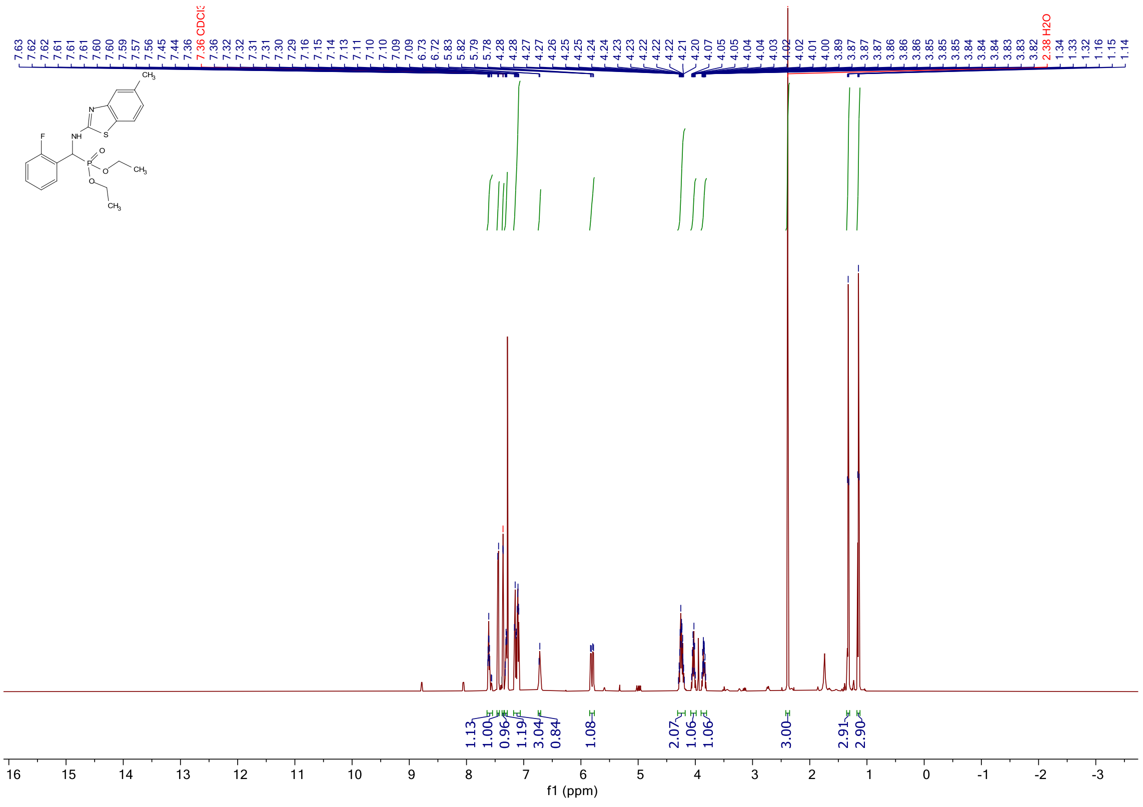
7.63 7.62 7.62 7.61 7.61 7.60 7.60 7.59 7.57 7.56 7.45 7.44 7.36 7.36 CDCl3 7.36 7.32 7.32 7.31 7.30 7.29 7.16 7.15 7.14 7.13 7.11 7.10 7.09 7.09 6.73 6.72 5.83 5.82 5.79 5.78 4.28 4.28 4.27 4.26 4.25 4.25 4.24 4.24 4.23 4.23 4.22 4.22 4.22 4.21 4.20 4.07 4.05 4.05 4.04 4.04 4.03 4.02 4.02 4.01 4.00 3.89 3.87 3.87 3.87 3.86 3.86 3.86 3.85 3.85 3.84 3.84 3.84 3.83 3.83 3.82 2.38 H2O 1.34 1.33 1.32 1.16 1.15 1.14
1.13 1.00 0.96 1.19 3.04 0.84 1.08 2.07 1.06 1.06 3.00 2.91 2.90
16 15 14 13 12 11 10 9 8 7 6 5 4 3 2 1 0 -1 -2 -3
f1 (ppm)

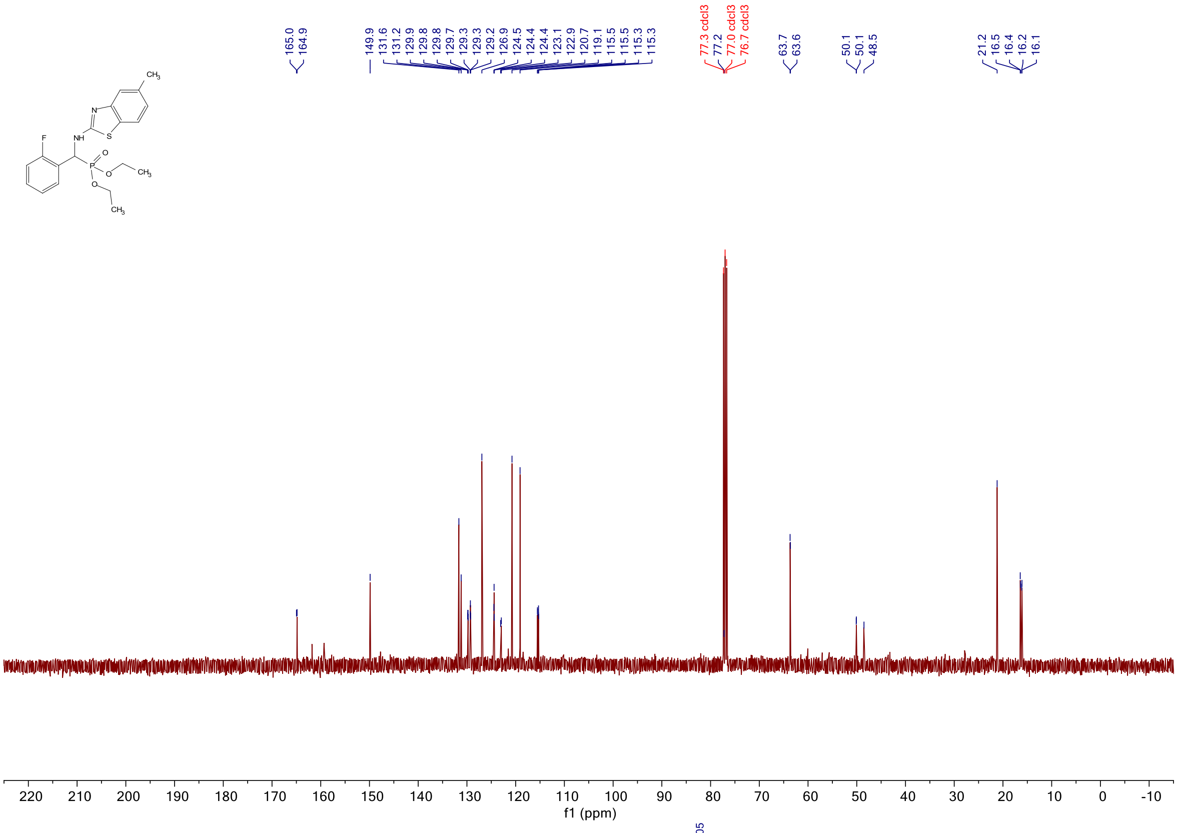
165.0
164.9
149.9
131.6
131.2
129.9
129.8
129.8
129.7
129.3
129.3
129.2
126.9
124.5
124.4
124.4
123.1
122.9
120.7
119.1
115.5
115.5
115.3
115.3
77.3 cdcl3
77.2
77.0 cdcl3
76.7 cdcl3
63.7
63.6
50.1
50.1
48.5
21.2
16.5
16.4
16.2
16.1
CH3
N
S
NH
F
P
O
O
CH3
O
CH3
f1 (ppm)

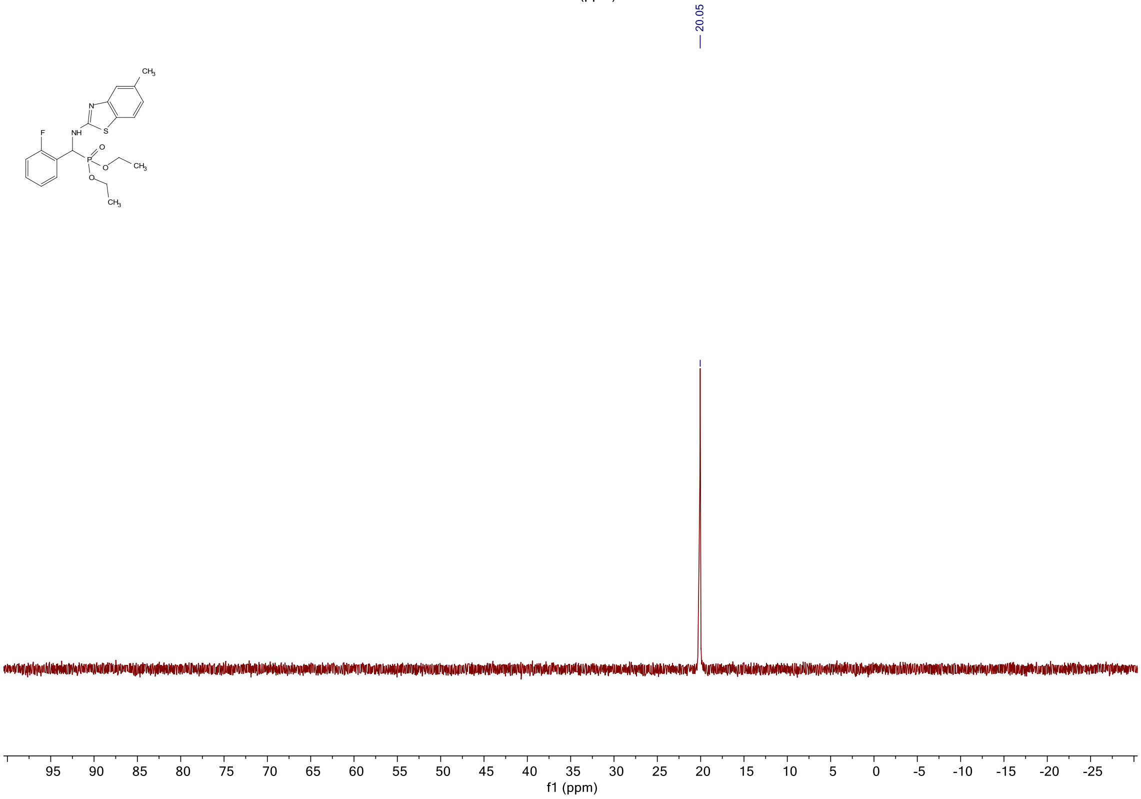
20.05
CH3
N
S
NH
F
P
O
O
CH3
O
CH3
f1 (ppm)

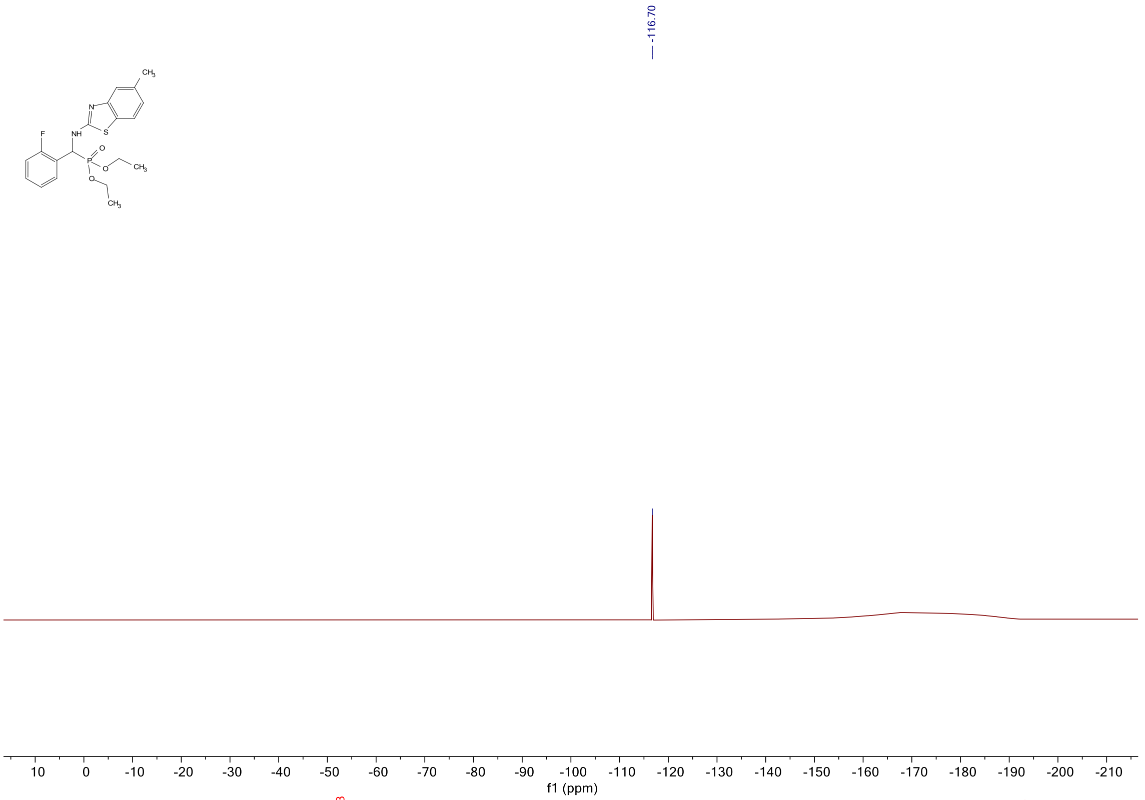

-116.70
f1 (ppm)

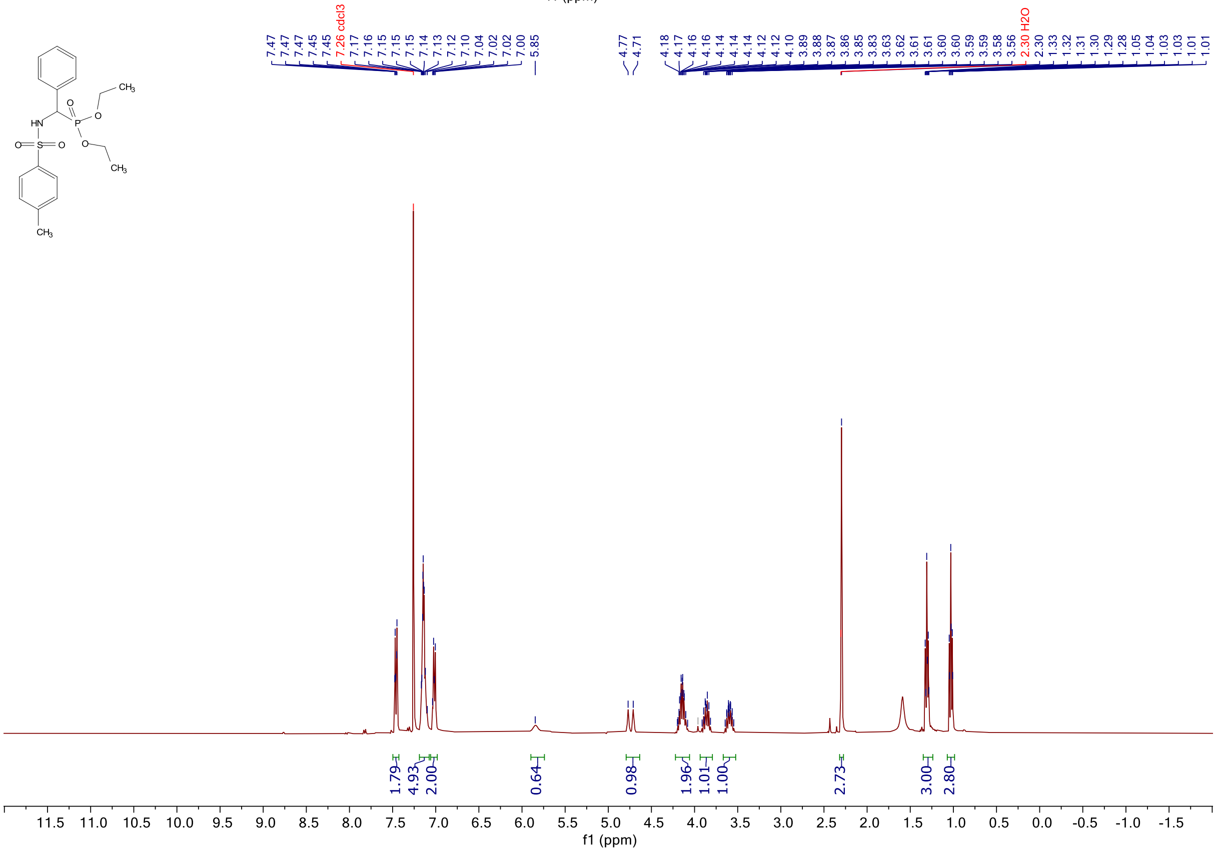

7.47 7.47 7.47 7.45 7.45 7.26 cdcl3 7.17 7.16 7.15 7.15 7.15 7.14 7.13 7.12 7.10 7.04 7.02 7.02 7.00 5.85
4.77 4.71
4.18 4.17 4.16 4.16 4.14 4.14 4.14 4.12 4.12 4.10 3.89 3.88 3.87 3.86 3.85 3.83 3.63 3.62 3.61 3.61 3.60 3.60 3.59 3.59 3.58 3.56 2.30 H2O 2.30 1.33 1.32 1.31 1.30 1.29 1.28 1.05 1.04 1.03 1.03 1.01 1.01
1.79 4.93 2.00 0.64 0.98 1.96 1.01 1.00 2.73 3.00 2.80
f1 (ppm)

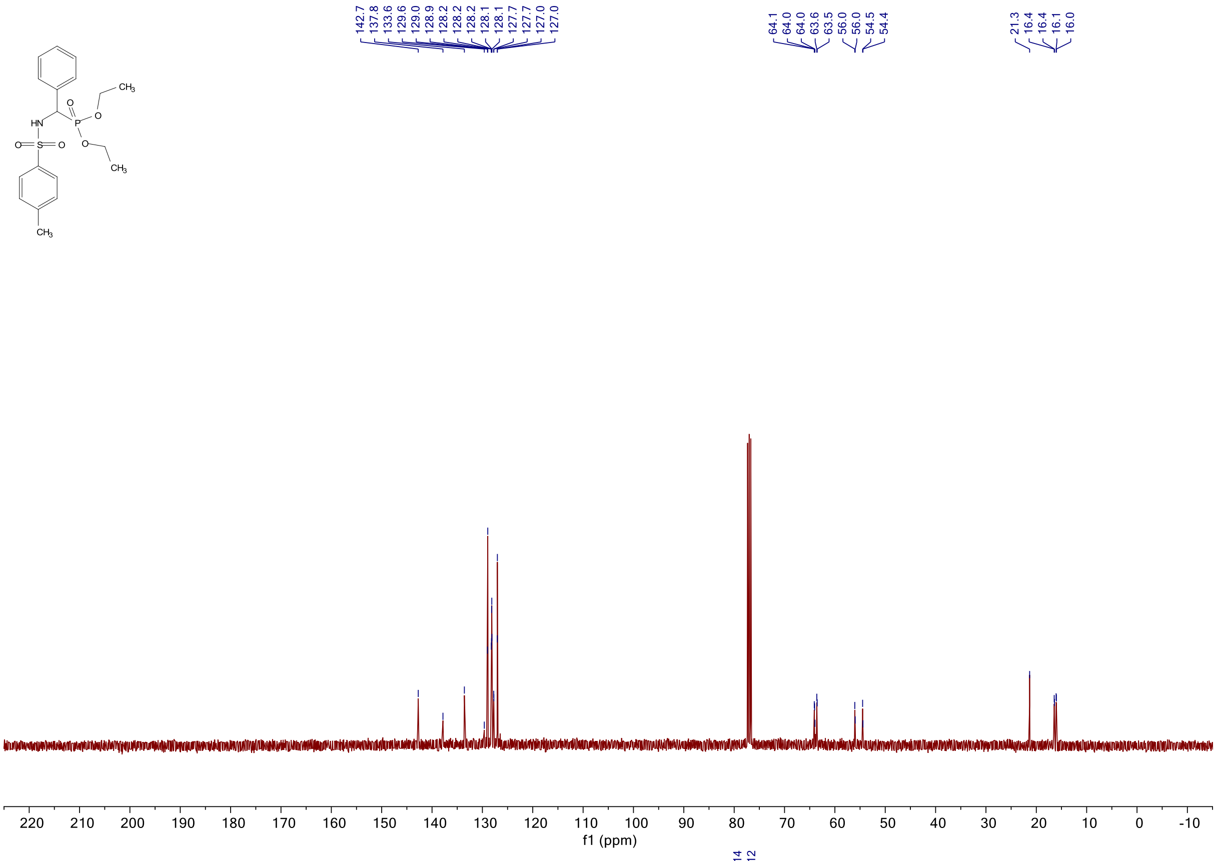
142.7
137.8
133.6
129.6
129.0
128.9
128.2
128.2
128.2
128.1
128.1
127.7
127.7
127.0
127.0
64.1
64.0
64.0
63.6
63.5
56.0
56.0
54.5
54.4
21.3
16.4
16.4
16.1
16.0
f1 (ppm)

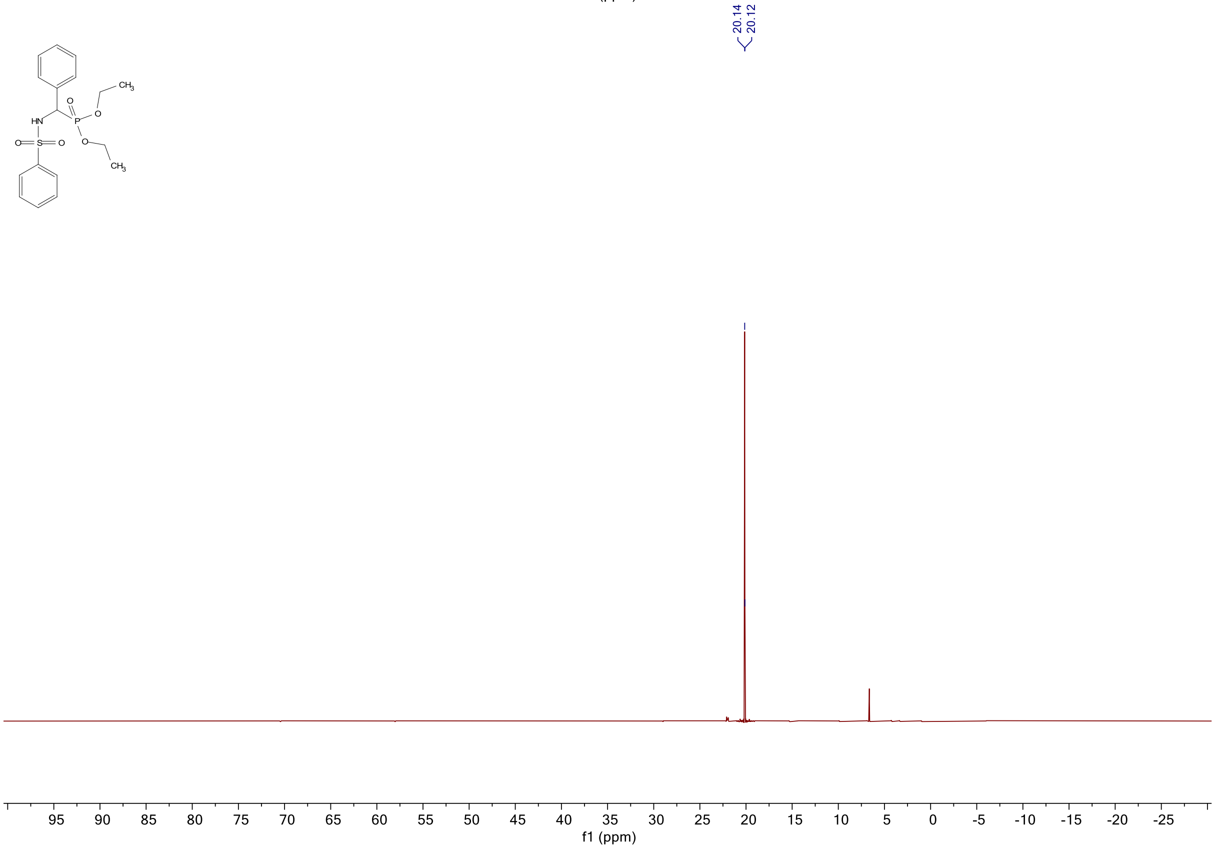
20.14
20.12
f1 (ppm)

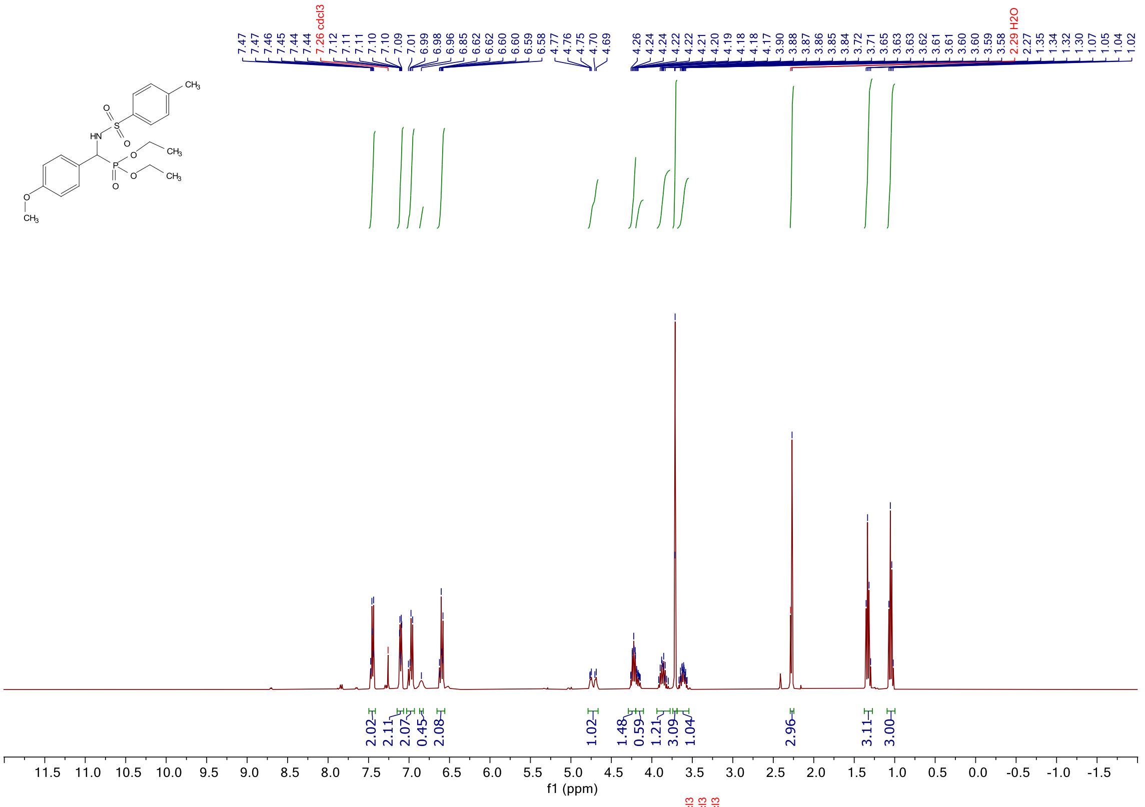

CH3
HN
O
S
O
P
O
O
CH3
CH3
O
CH3
7.47 7.47 7.46 7.45 7.44 7.44 7.26 cdcl3 7.12 7.11 7.10 7.10 7.09 7.01 6.99 6.98 6.96 6.85 6.62 6.62 6.60 6.60 6.59 6.58 4.77 4.76 4.75 4.70 4.69 4.26 4.24 4.24 4.22 4.22 4.21 4.20 4.19 4.18 4.18 4.17 3.90 3.88 3.87 3.86 3.85 3.84 3.72 3.71 3.65 3.63 3.63 3.62 3.61 3.61 3.60 3.60 3.59 3.58 2.29 H2O 2.27 1.35 1.34 1.32 1.30 1.07 1.05 1.04 1.02
2.02 2.11 2.07 0.45 2.08 1.02 1.48 0.59 1.21 3.09 1.04 2.96 3.11 3.00
11.5 11.0 10.5 10.0 9.5 9.0 8.5 8.0 7.5 7.0 6.5 6.0 5.5 5.0 4.5 4.0 3.5 3.0 2.5 2.0 1.5 1.0 0.5 0.0 -0.5 -1.0 -1.5
f1 (ppm)

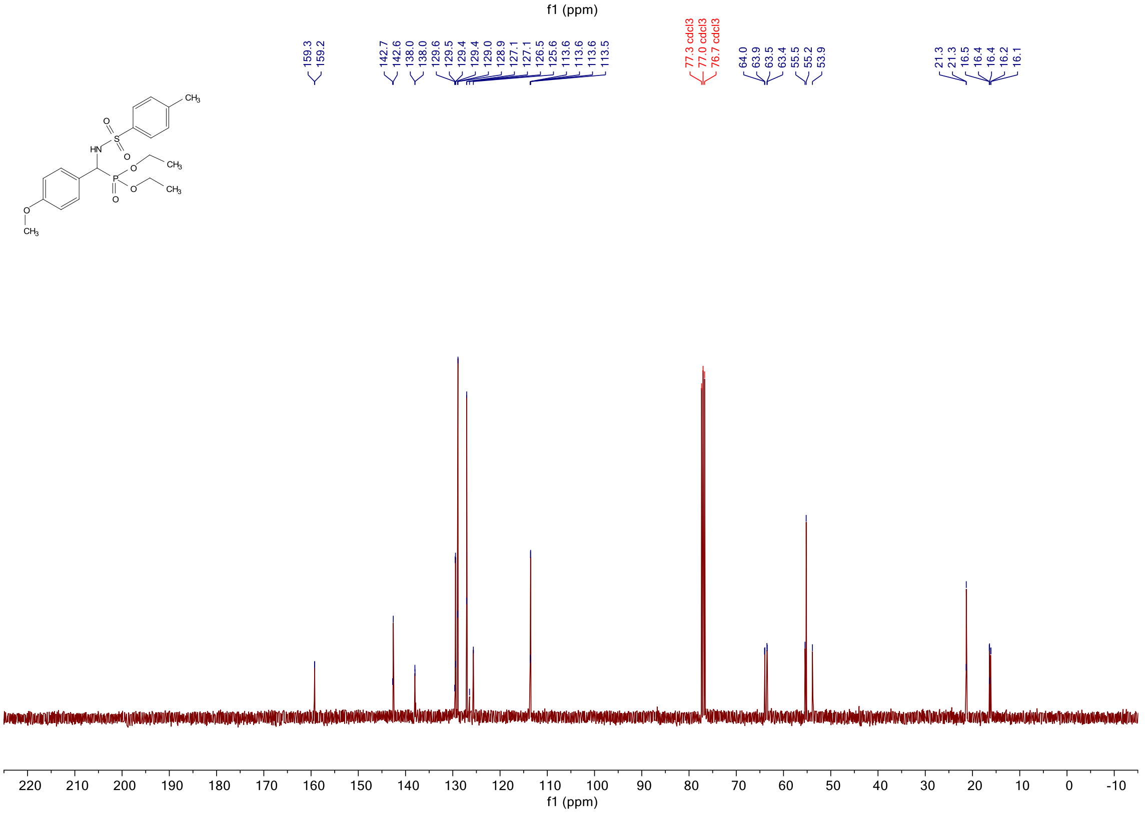

159.3 159.2 142.7 142.6 138.0 138.0 129.6 129.5 129.4 129.4 129.0 128.9 127.1 127.1 126.5 125.6 113.6 113.6 113.6 113.5 77.3 cdcl3 77.0 cdcl3 76.7 cdcl3 64.0 63.9 63.5 63.4 55.5 55.2 53.9 21.3 21.3 16.5 16.4 16.4 16.2 16.1
220 210 200 190 180 170 160 150 140 130 120 110 100 90 80 70 60 50 40 30 20 10 0 -10
f1 (ppm)

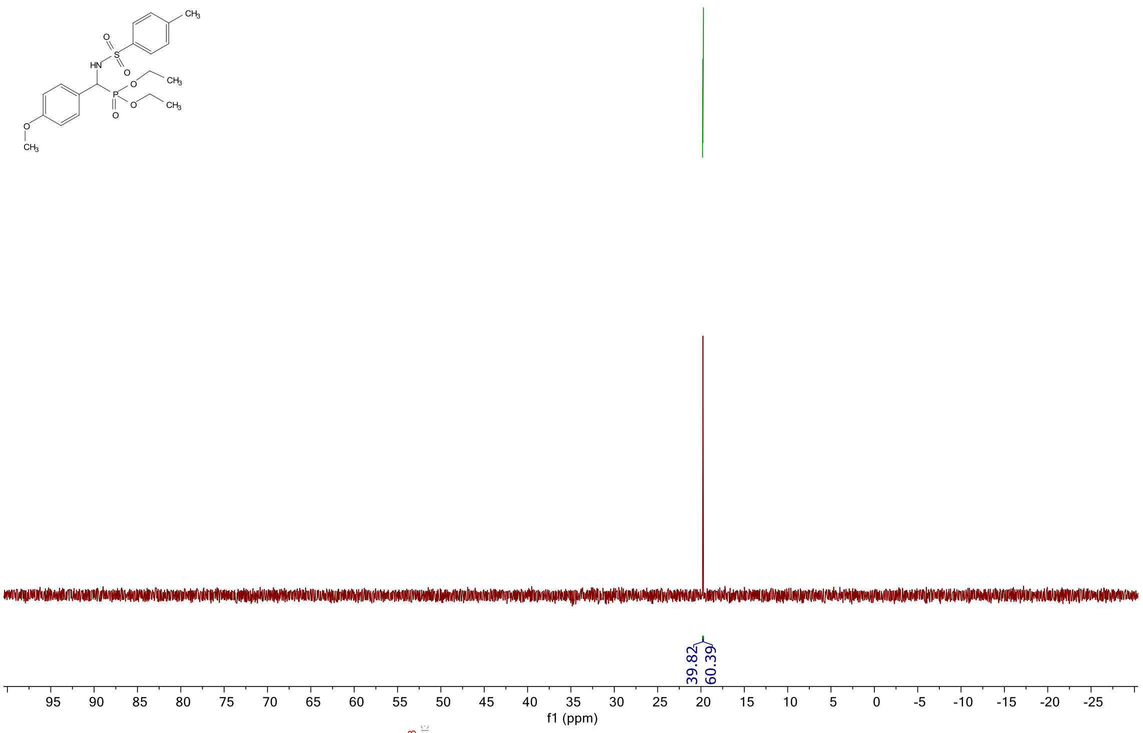

CH_3
HN
O
S
O
P
O
CH_3
O
CH_3
O
CH_3
39.82
60.39
95 90 85 80 75 70 65 60 55 50 45 40 35 30 25 20 15 10 5 0 -5 -10 -15 -20 -25
f1 (ppm)

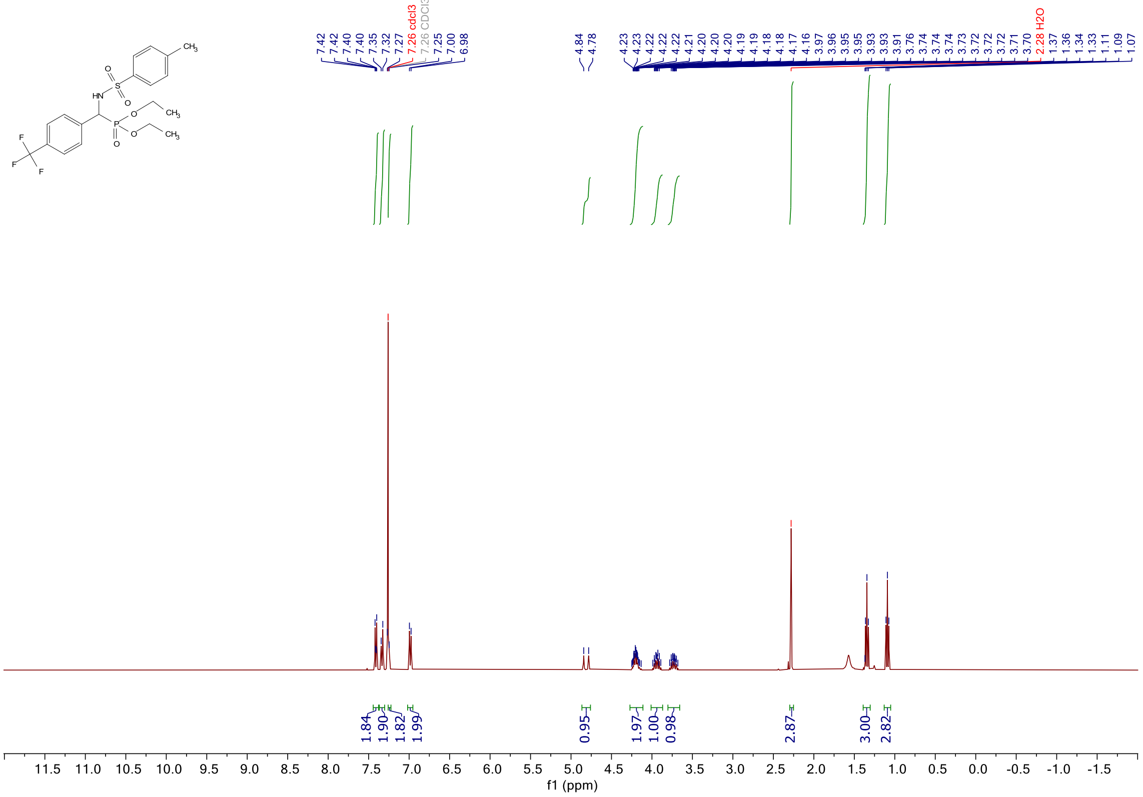

CH_3
HN
O
S
O
P
O
CH_3
O
CH_3
F
F
F
7.42 7.42 7.40 7.35 7.32 7.27 7.26 cdcl3 7.26 CDCl3 7.25 7.00 6.98
4.84 4.78
4.23 4.23 4.22 4.22 4.22 4.21 4.20 4.20 4.20 4.19 4.19 4.18 4.18 4.17 4.16 3.97 3.96 3.95 3.95 3.93 3.93 3.91 3.76 3.74 3.74 3.74 3.73 3.72 3.72 3.72 3.71 3.70 2.28 H2O 1.37 1.36 1.34 1.33 1.11 1.09 1.07
1.84 1.90 1.82 1.99
0.95
1.97 1.00 0.98
2.87
3.00 2.82
11.5 11.0 10.5 10.0 9.5 9.0 8.5 8.0 7.5 7.0 6.5 6.0 5.5 5.0 4.5 4.0 3.5 3.0 2.5 2.0 1.5 1.0 0.5 0.0 -0.5 -1.0 -1.5
f1 (ppm)

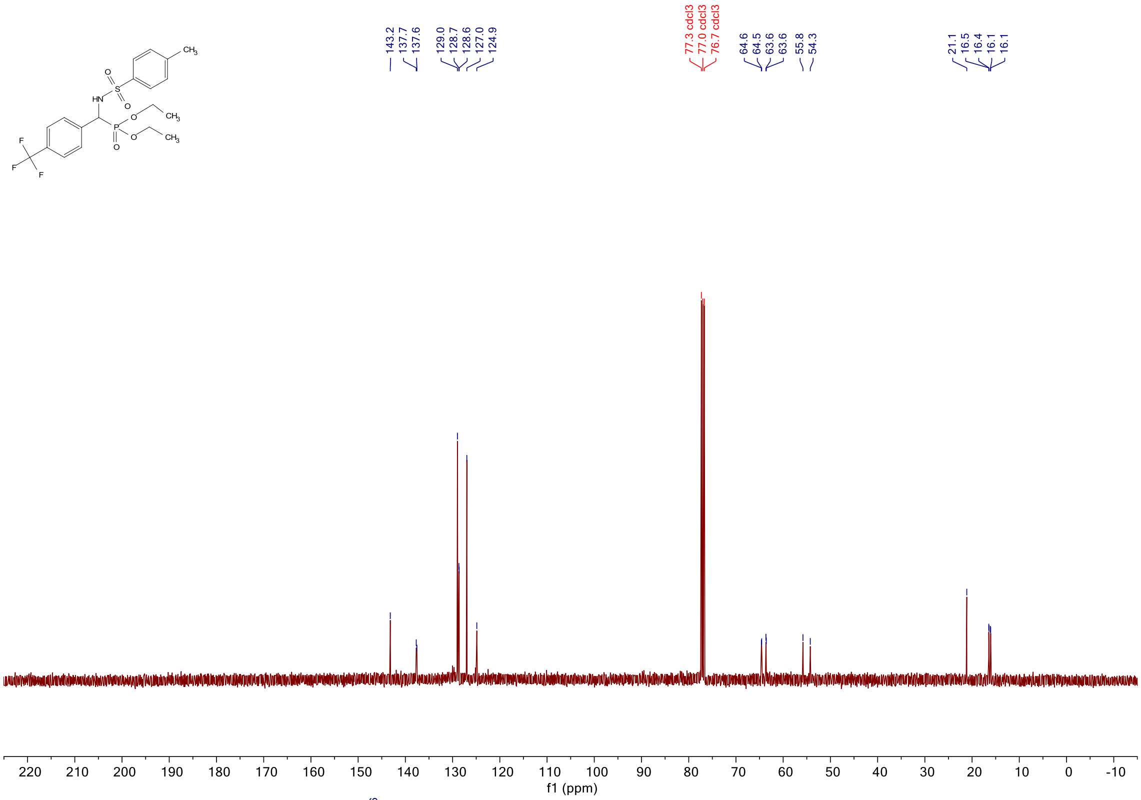
143.2
137.7
137.6
129.0
128.7
128.6
127.0
124.9
77.3 cdcl3
77.0 cdcl3
76.7 cdcl3
64.6
64.5
63.6
63.6
55.8
54.3
21.1
16.5
16.4
16.1
16.1
f1 (ppm)

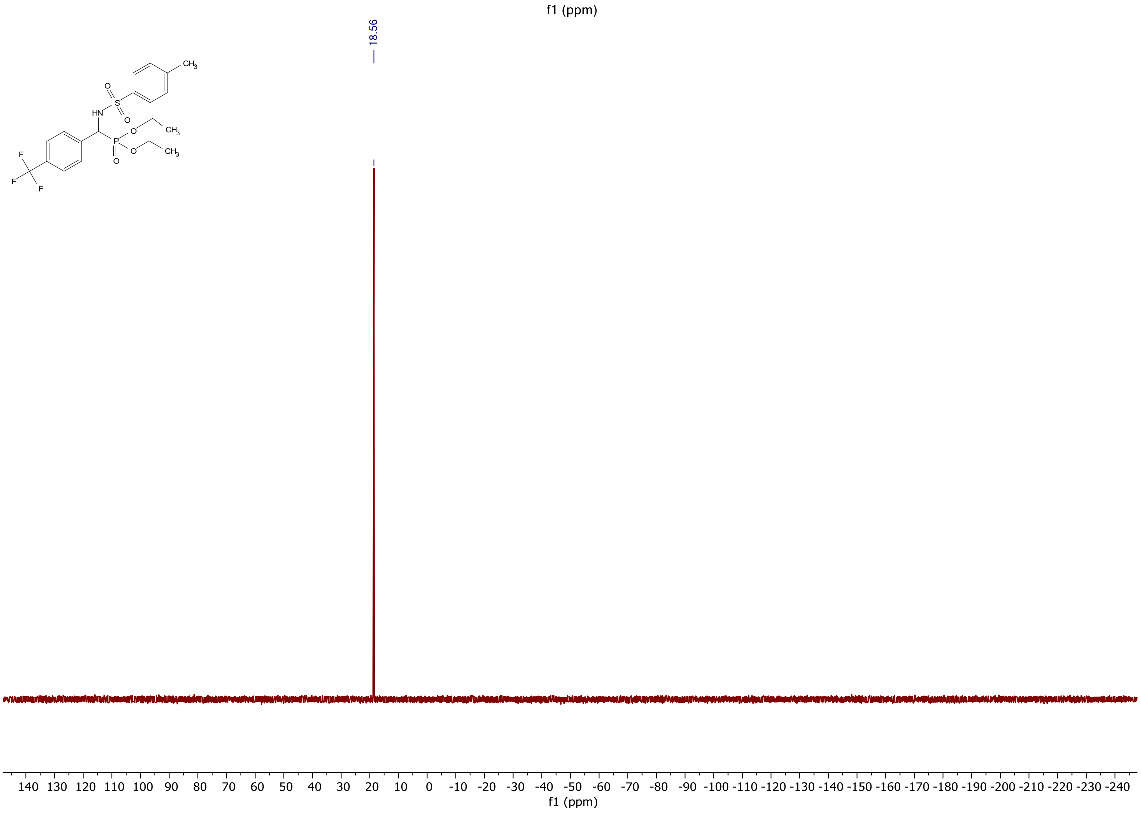
18.56
f1 (ppm)

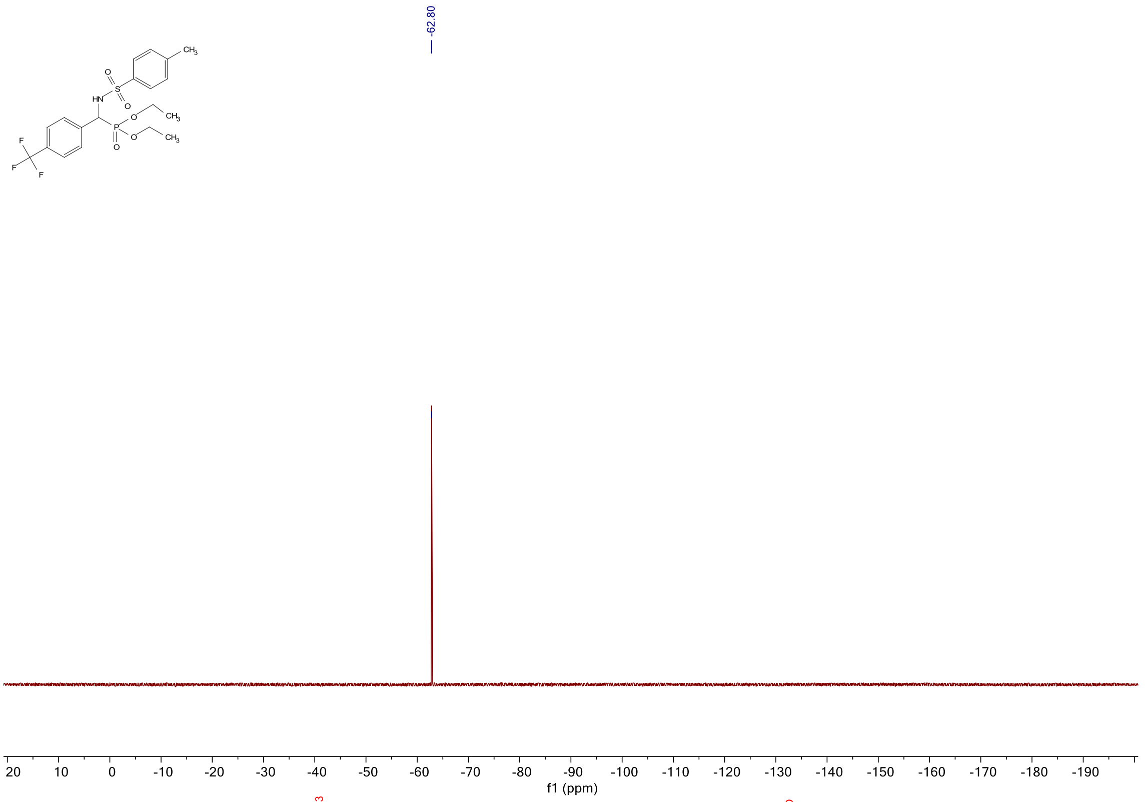

-62.80
20 10 0 -10 -20 -30 -40 -50 -60 -70 -80 -90 -100 -110 -120 -130 -140 -150 -160 -170 -180 -190
f1 (ppm)

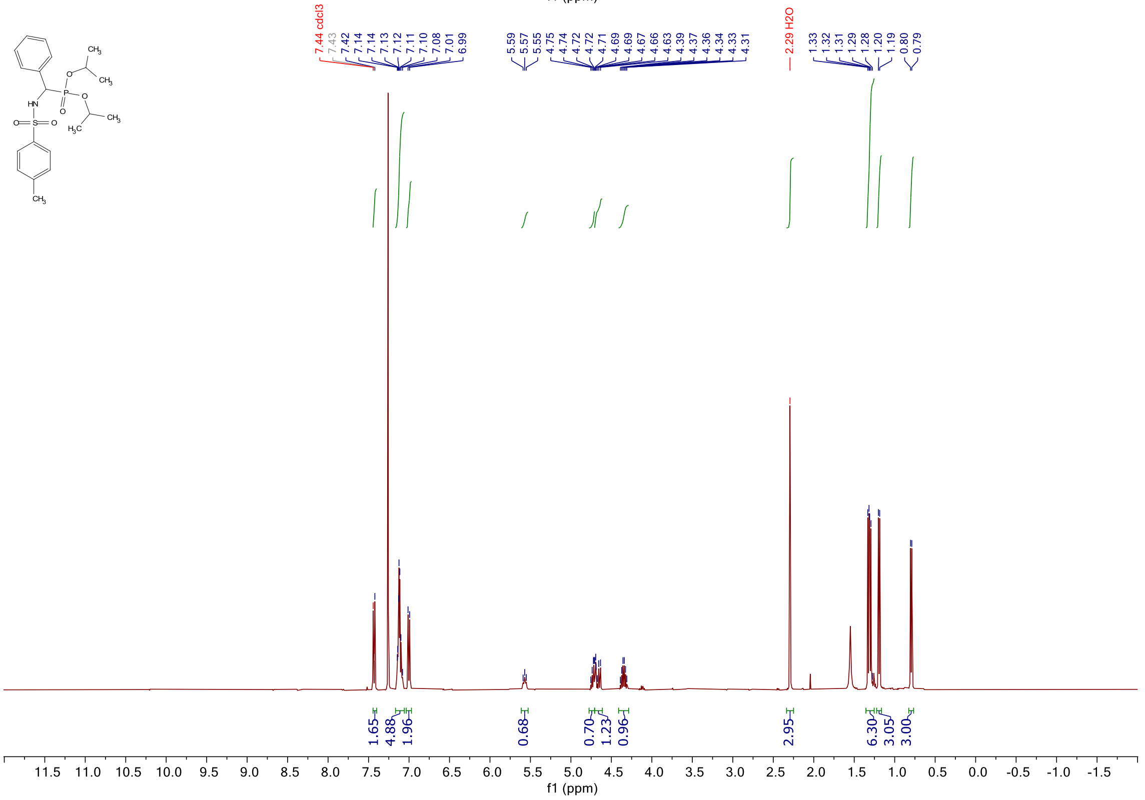

7.44 cdcl3
7.43
7.42
7.14
7.13
7.12
7.11
7.10
7.08
7.01
6.99
5.59
5.57
5.55
4.75
4.74
4.72
4.72
4.71
4.69
4.69
4.67
4.66
4.63
4.39
4.37
4.36
4.34
4.33
4.31
2.29 H2O
1.33
1.32
1.31
1.29
1.28
1.20
1.19
0.80
0.79
1.65
4.88
1.96
0.68
0.70
1.23
0.96
2.95
6.30
3.05
3.00
11.5 11.0 10.5 10.0 9.5 9.0 8.5 8.0 7.5 7.0 6.5 6.0 5.5 5.0 4.5 4.0 3.5 3.0 2.5 2.0 1.5 1.0 0.5 0.0 -0.5 -1.0 -1.5
f1 (ppm)

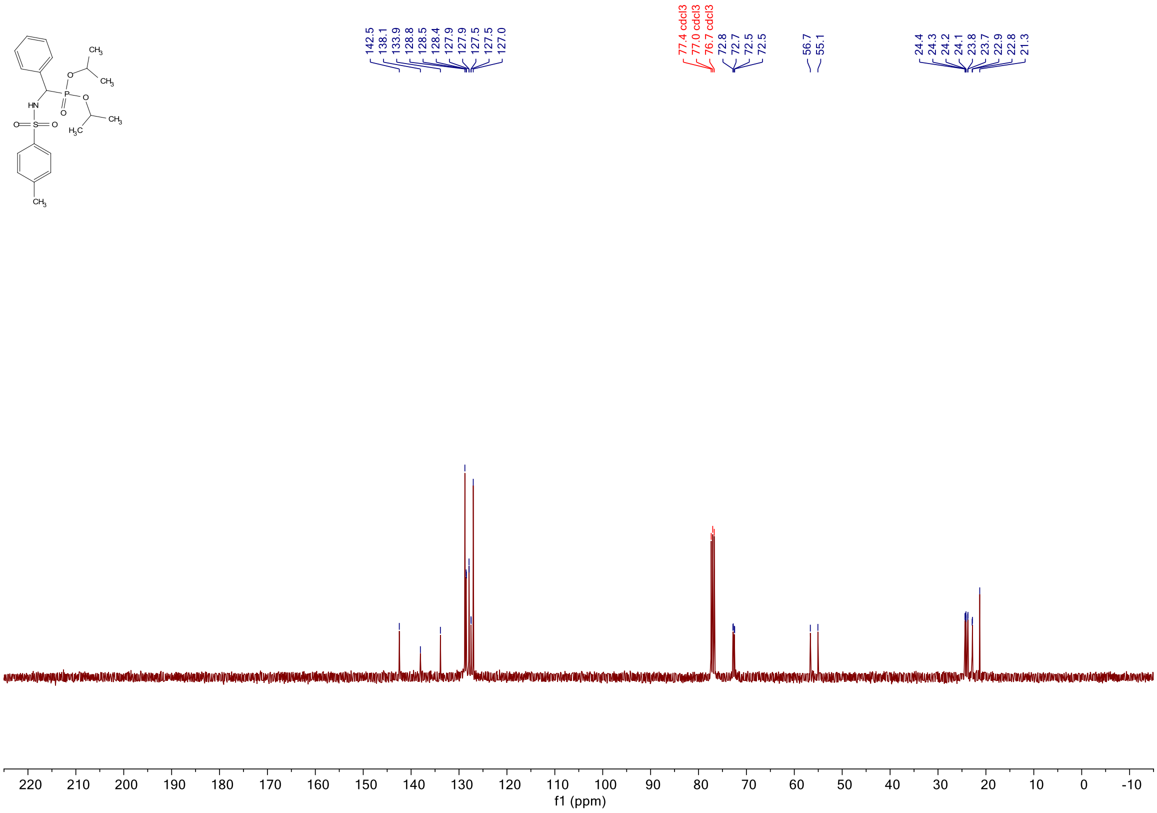
142.5
138.1
133.9
128.8
128.5
128.4
127.9
127.9
127.5
127.5
127.0
77.4 cdcl3
77.0 cdcl3
76.7 cdcl3
72.8
72.7
72.5
72.5
56.7
55.1
24.4
24.3
24.2
24.1
23.8
23.7
22.9
22.8
21.3
HN
O
P
O
O
CH3
CH3
H3C
CH3
O=S=O
CH3
220 210 200 190 180 170 160 150 140 130 120 110 100 90 80 70 60 50 40 30 20 10 0 -10
f1 (ppm)

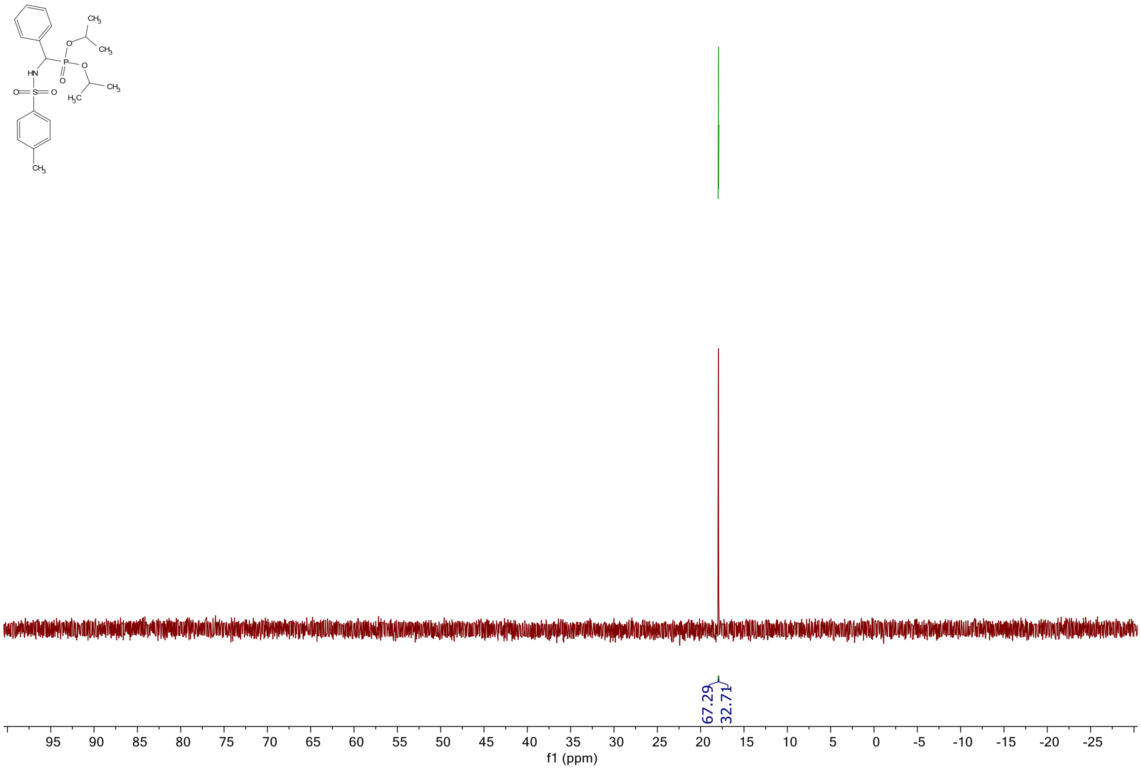
HN
O
P
O
O
CH3
CH3
H3C
CH3
O=S=O
CH3
67.29
32.71
95 90 85 80 75 70 65 60 55 50 45 40 35 30 25 20 15 10 5 0 -5 -10 -15 -20 -25
f1 (ppm)

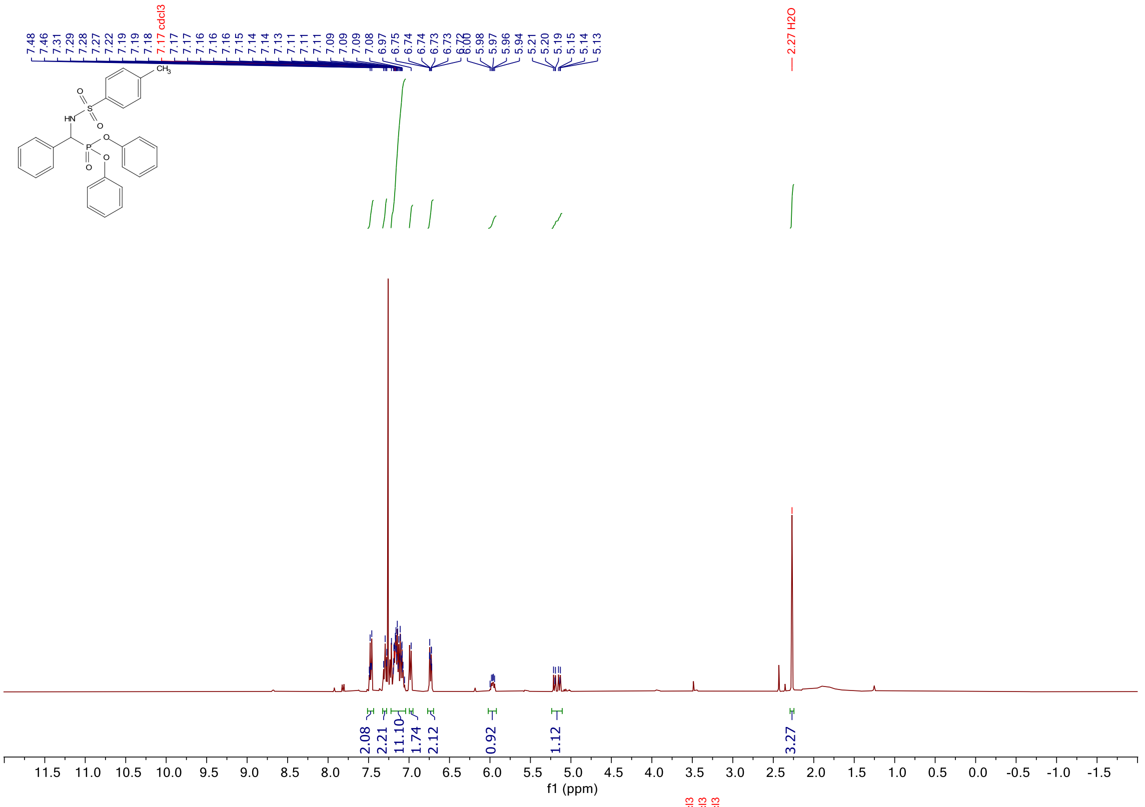
7.48
7.46
7.31
7.29
7.28
7.27
7.22
7.19
7.19
7.18
7.17 cdcl3
7.17
7.17
7.16
7.16
7.16
7.15
7.14
7.13
7.11
7.11
7.11
7.09
7.09
7.09
7.08
6.97
6.75
6.74
6.74
6.73
6.73
6.72
6.00
5.98
5.97
5.96
5.94
5.21
5.20
5.19
5.15
5.14
5.13
2.27 H2O
2.08
2.21
11.10
1.74
2.12
0.92
1.12
3.27
f1 (ppm)

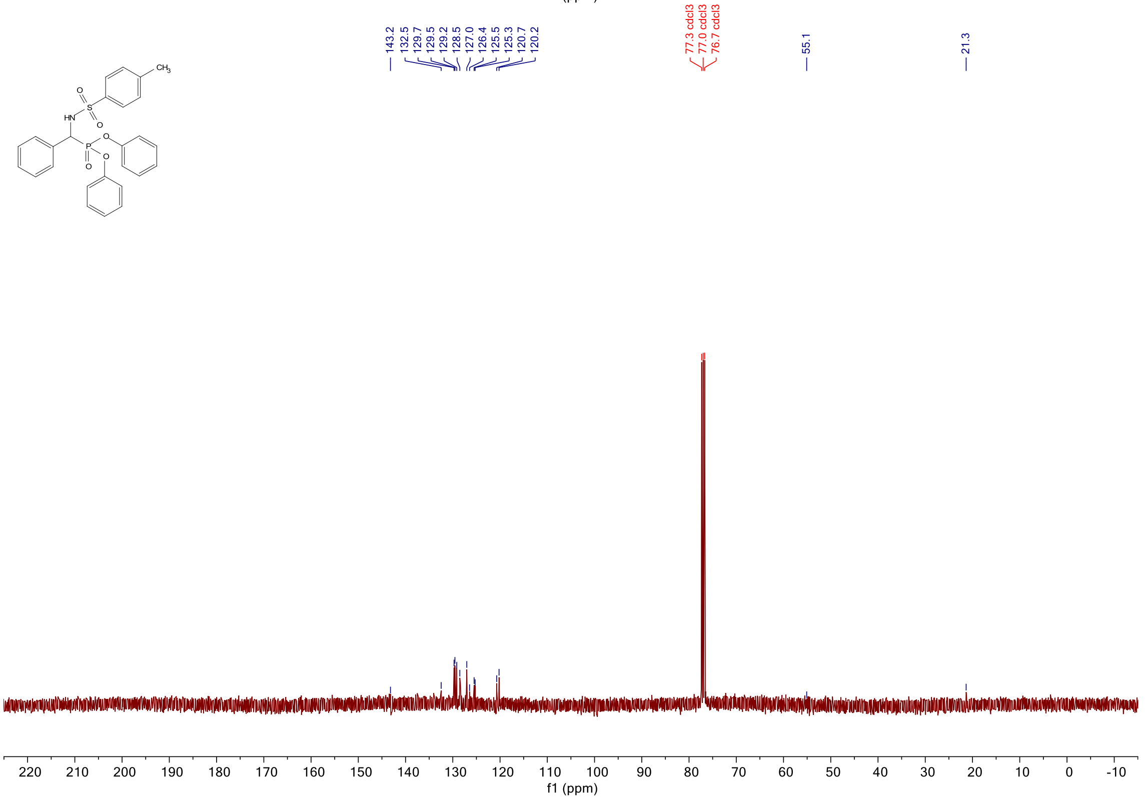
143.2
132.5
129.7
129.5
129.2
128.5
127.0
126.4
125.5
125.3
120.7
120.2
77.3 cdcl3
77.0 cdcl3
76.7 cdcl3
55.1
21.3
f1 (ppm)

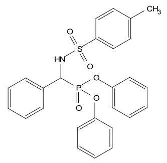

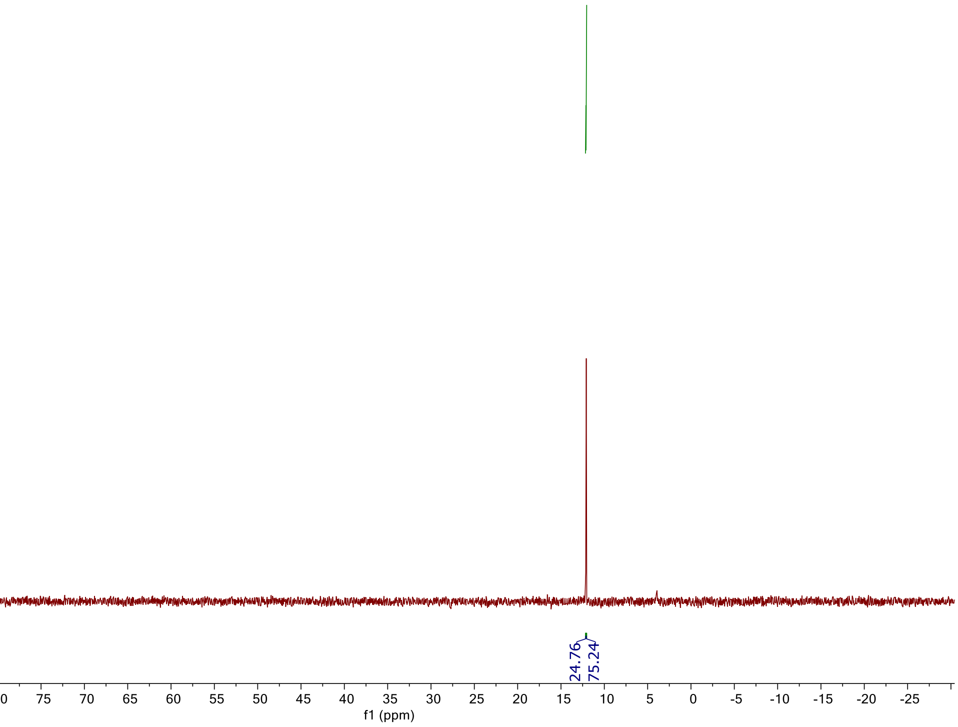

24.76
75.24
95 90 85 80 75 70 65 60 55 50 45 40 35 30 25 20 15 10 5 0 -5 -10 -15 -20 -25
f1 (ppm)

11. References

(1) Evans, R.; Dal Poggetto, G.; Nilsson, M.; Morris, G. A. Improving the Interpretation of Small Molecule Diffusion Coefficients. *Anal Chem* **2018**, *90* (6), 3987-3994.
(2) Dašková, V.; Buter, J.; Schoonen, A. K.; Lutz, M.; de Vries, F.; Feringa, B. L. Chiral Amplification of Phosphoramidates of Amines and Amino Acids in Water. *Angew. Chem. Int. Ed.* **2021**, *60* (20), 11120-11126.
(3) Wiecko, M.; Girnt, D.; Rastätter, M.; Panda, T. K.; Roesky, P. W. Zirconium complexes having a chiral phosphanylamide in the co-ordination sphere. *Dalton Trans.* **2005**, (12), 2147-2150.
(4) Storch, G.; Haas, M.; Trapp, O. Attracting Enantiomers: Chiral Analytes That Are Simultaneously Shift Reagents Allow Rapid Screening of Enantiomeric Ratios by NMR Spectroscopy. *Chem. Eur. J.* **2017**, *23* (23), 5414-5418.
(5) Weck, C.; Nauha, E.; Gruber, T. Does the Exception Prove the Rule? A Comparative Study of Supramolecular Synthons in a Series of Lactam Esters. *Cryst. Growth Des.* **2019**, *19* (5), 2899-2911.
(6) Li, W.; Wang, Y.; Xu, D. Asymmetric Synthesis of α-Amino Phosphonates by Using Cinchona Alkaloid-Based Chiral Phase Transfer Catalyst. *Eur. J. Org. Chem.* **2018**, *2018* (39), 5422-5426.
(7) Yan, Z.; Wu, B.; Gao, X.; Chen, M.-W.; Zhou, Y.-G. Enantioselective Synthesis of α-Amino Phosphonates via Pd-Catalyzed Asymmetric Hydrogenation. *Org. Lett.* **2016**, *18* (4), 692-695.
(8) Li, L.; Song, B.-A.; Bhadury, P. S.; Zhang, Y.-P.; Hu, D.-Y.; Yang, S. Enantioselective Synthesis of β-Amino Esters Bearing a Benzothiazole Moiety via a Mannich-Type Reaction Catalyzed by a Cinchona Alkaloid Derivative. *Eur. J. Org. Chem.* **2011**, *2011* (25), 4743-4746.
(9) Nogales, D. F.; Ma, J.-S.; Lightner, D. A. Self-association of dipyrrinones observed by 2d-noe nmr and dimerization constants calculated from 1h-nmr chemical shifts. *Tetrahedron* **1993**, *49* (12), 2361-2372.
(10) Thordarson, P. Determining association constants from titration experiments in supramolecular chemistry. *Chem. Soc. Rev.* **2011**, *40* (3), 1305-1323.
(11) Nakao, Y.; Sugeta, H.; Kyogoku, Y. Intermolecular Hydrogen Bonding of Enantiomers of Pantolactone Studied by Infrared and 1H-NMR Spectroscopy. *Bull. Chem. Soc. Jpn.* **1985**, *58* (6), 1767-1771.
(12) Szakács, Z.; Sánta, Z.; Lomoschitz, A.; Szántay, C. Self-induced recognition of enantiomers (SIRE) and its application in chiral NMR analysis. *TrAC, Trends Anal. Chem.* **2018**, *109*, 180-197.
(13) Smallcombe, S. H.; Patt, S. L.; Keifer, P. A. WET Solvent Suppression and Its Applications to LC NMR and High-Resolution NMR Spectroscopy. *J. Magn. Reso.* **1995**, *117* (2), 295-303.
(14) Storer, M. C.; Hunter, C. A. The surface site interaction point approach to non-covalent interactions. *Chem. Soc. Rev.* **2022**, *51* (24), 10064-10082, 10.1039/D2CS00701K.
(15) Dašková, V.; Padín, D.; Feringa, B. L. Turning Enantiomeric Relationships into Diastereomeric Ones: Self-Resolving α-Ureidophosphonates and Their Organocatalytic Enantioselective Synthesis. *J. Am. Chem. Soc.* **2022**, *144* (51), 23603-23613.
(16) Kudzin, Z. H.; uczak, J. A Facile Conversion of Aminoalkanephosphonic Acids Into O,O-Dialkyl N-Acylaminoalkanephosphonate Derivatives. *Synthesis* **1995**, *1995* (05), 509-511.
(17) Hu, L. a.; Wang, Y.-Z.; Xu, L.; Yin, Q.; Zhang, X. Highly Enantioselective Synthesis of N-Unprotected Unnatural α-Amino Acid Derivatives by Ruthenium-Catalyzed Direct Asymmetric Reductive Amination. *Angew. Chem. Int. Ed.* **2022**, *61* (25), e202202552.
(18) Kriis, K.; Martõnov, H.; Miller, A.; Erkman, K.; Järving, I.; Kaasik, M.; Kanger, T. Multifunctional Catalysts in the Asymmetric Mannich Reaction of Malononitrile with N-Phosphinoylimines: Coactivation by Halogen Bonding versus Hydrogen Bonding. *J. Org. Chem.* **2022**, *87* (11), 7422-7435
(19) Czerwiński, P. J.; Furman, B. Overcoming inaccessibility of fluorinated imines – synthesis of functionalized amines from readily available fluoroacetamides. *Chem. Comm.* **2019**, *55* (64), 9436-9439, 10.1039/C9CC04111G.
(20) Balzano, F.; Iuliano, A.; Uccello-Barretta, G.; Zullo, V. Renewable Resources for Enantiodiscrimination: Chiral Solvating Agents for NMR Spectroscopy from Isomannide and Isosorbide. *J. Org. Chem.* **2022**, *87* (19), 12698-12709.
(21) Cuřínová, P.; Dračínský, M.; Jakubec, M.; Tlustý, M.; Janků, K.; Izák, P.; Holakovský, R. Enantioselective complexation of 1-phenylethanol with chiral compounds bearing urea moiety. *Chirality* **2018**, *30* (6), 798-806.

(22) Puccetti, F.; Rinesch, T.; Suljić, S.; Rahimi, K.; Herrmann, A.; Bolm, C. NMR in operando monitoring of mechanochemically accelerated sublimations. *Chem* **2023**, *9* (5), 1318-1332.
(23) Zhou, S.; Fleischer, S.; Junge, K.; Das, S.; Addis, D.; Beller, M. Enantioselective Synthesis of Amines: General, Efficient Iron-Catalyzed Asymmetric Transfer Hydrogenation of Imines. *Angewandte Chemie International Edition* **2010**, *49* (44), 8121-8125. DOI: https://doi.org/10.1002/anie.201002456.
(24) Huang, S.-H.; Bai, Z.-W.; Feng, J.-W. Chiral self-discrimination of the enantiomers of α-phenylethylamine derivatives in proton NMR. *Magn. Reson. Chem.* **2009**, *47* (5), 423-427.
(25) Munagala, G.; Yempalla, K. R.; Aithagani, S. K.; Kalia, N. P.; Ali, F.; Ali, I.; Rajput, V. S.; Rani, C.; Chib, R.; Mehra, R.; et al. Synthesis and biological evaluation of substituted N-alkylphenyl-3,5-dinitrobenzamide analogs as anti-TB agents. *MedChemComm* **2014**, *5* (4), 521-527, 10.1039/C3MD00366C.
(26) Zuend, S. J.; Coughlin, M. P.; Lalonde, M. P.; Jacobsen, E. N. Scaleable catalytic asymmetric Strecker syntheses of unnatural α-amino acids. *Nature* **2009**, *461* (7266), 968-970.
(27) Besson, M.; Delbecq, F.; Gallezot, P.; Neto, S.; Pinel, C. Asymmetric synthesis of 2-methyl cyclohexane carboxylic acids by heterogeneous catalysis: mechanistic aspects. *Chem.* **2000**, *6* (6), 949-958.
(28) Okino, T.; Hoashi, Y.; Takemoto, Y. Enantioselective Michael Reaction of Malonates to Nitroolefins Catalyzed by Bifunctional Organocatalysts. *J. Am. Chem. Soc.* **2003**, *125* (42), 12672-12673.
(29) Ghorai, M. K.; Das, S.; Das, K.; Kumar, A. Stereoselective synthesis of activated 2-arylazetidines via imino-aldol reaction. *Org. Biomol. Chem.* **2015**, *13* (34), 9042-9049.
(30) Pettersen, D.; Marcolini, M.; Bernardi, L.; Fini, F.; Herrera, R. P.; Sgarzani, V.; Ricci, A. Direct Access to Enantiomerically Enriched α-Amino Phosphonic Acid Derivatives by Organocatalytic Asymmetric Hydrophosphonylation of Imines. *J. Org. Chem.* **2006**, *71* (16), 6269-6272.
(31) Vicario, J.; Ezpeleta, J. M.; Palacios, F. Asymmetric Cyanation of α-Ketiminophosphonates Catalyzed by Cinchona Alkaloids: Enantioselective Synthesis of Tetrasubstituted α-Aminophosphonic Acid Derivatives from Trisubstituted α-Aminophosphonates. *Adv. Synth. Catal.* **2012**, *354* (14-15), 2641-2647.
(32) Bhagat, S.; Supriya, M.; Pathak, S.; Sriram, D.; Chakraborti, A. K. α-Sulfonamidophosphonates as new anti-mycobacterial chemotypes: Design, development of synthetic methodology, and biological evaluation. *Bioorg. Chem.* **2019**, *82*, 246-252.
